# Oral and middle ear delivery of otitis media standard of care antibiotics, but not biofilm-targeted antibodies, alter chinchilla nasopharyngeal and fecal microbiomes

**DOI:** 10.1038/s41522-024-00481-0

**Published:** 2024-02-03

**Authors:** Audrey F. Duff, Joseph A. Jurcisek, Nikola Kurbatfinski, Tendy Chiang, Steven D. Goodman, Lauren O. Bakaletz, Michael T. Bailey

**Affiliations:** 1 Center for Microbial Pathogenesis, Abigail Wexner Research Institute at Nationwide Children’s Hospital, Columbus, OH USA; 2 Department of Otolaryngology at Nationwide Children’s Hospital, Columbus, OH USA; 3 Department of Pediatrics, The Ohio State University, Wexner Medical Center, Columbus, OH USA; 4 Oral and Gastrointestinal Microbiology Research Affinity Group, Abigail Wexner Research Institute at Nationwide Children’s Hospital, Columbus, OH USA

**Keywords:** Microbiome, Microbiota

## Abstract

Otitis media (OM) is one of the most globally pervasive pediatric conditions. Translocation of nasopharynx-resident opportunistic pathogens like nontypeable *Haemophilus influenzae* (NTHi) assimilates into polymicrobial middle ear biofilms, which promote OM pathogenesis and substantially diminish antibiotic efficacy. Oral or tympanostomy tube (TT)-delivered antibiotics remain the standard of care (SOC) despite consequences including secondary infection, dysbiosis, and antimicrobial resistance. Monoclonal antibodies (mAb) against two biofilm-associated structural proteins, NTHi-specific type IV pilus PilA (anti-rsPilA) and protective tip-region epitopes of NTHi integration host factor (anti-tip-chimer), were previously shown to disrupt biofilms and restore antibiotic sensitivity in vitro. However, the additional criterion for clinical relevance includes the absence of consequential microbiome alterations. Here, nine chinchilla cohorts (*n* = 3/cohort) without disease were established to evaluate whether TT delivery of mAbs disrupted nasopharyngeal or fecal microbiomes relative to SOC-OM antibiotics. Cohort treatments included a 7d regimen of oral amoxicillin-clavulanate (AC) or 2d regimen of TT-delivered mAb, AC, Trimethoprim-sulfamethoxazole (TS), ofloxacin, or saline. Fecal and nasopharyngeal lavage (NPL) samples were collected before and several days post treatment (DPT) for 16S sequencing. While antibiotic-treated cohorts displayed beta-diversity shifts (PERMANOVA, *P* < 0.05) and reductions in alpha diversity (*q* < 0.20) relative to baseline, mAb antibodies failed to affect diversity, indicating maintenance of a eubiotic state. Taxonomic and longitudinal analyses showed blooms in opportunistic pathogens (ANCOM) and greater magnitudes of compositional change (*P* < 0.05) following broad-spectrum antibiotic but not mAb treatments. Collectively, results showed broad-spectrum antibiotics induced significant fecal and nasopharyngeal microbiome disruption regardless of delivery route. Excitingly, biofilm-targeting antibodies had little effect on fecal and nasopharyngeal microbiomes.

## Introduction

Otitis media (OM), an inflammatory infection of the middle ear, is one of the most prevalent pediatric diseases worldwide with upwards of 80–90% of children under 5 years of age experiencing at least one episode^1,2^. The umbrella of OM encompasses a spectrum of clinical subtypes that include acute symptomatic OM (AOM), asymptomatic OM with effusion (OME), and chronic suppurative otitis media (CSOM) which are characterized by the presentation of symptoms, pathogenesis, and recurrence^3,4^. Surgical insertion of tympanostomy tubes (TT), a therapeutic intervention to permit middle ear ventilation while still permitting administration of topical antibiotics, can also introduce a risk of infection by translocated bacteria and subsequent post-tympanostomy tube otorrhea (PTTO) in over half of recipient children within a year of placement^5^. The global burden of OM is estimated to be 709 million AOM cases and 31 million CSOM cases with 51% and 22.5%, respectively, occurring in children under five^6^. Consequences of OM infections can include ear discomfort and fluid discharge, eardrum perforation, vestibular problems, and hearing impairment which exert negative influences on development, behavior, academic performance, and quality of life^7^. In the U.S. alone, the spectrum of OM clinical entities represents an annual healthcare burden of approximately $4 billion, not inclusive of indirect costs accrued by caregivers^8,9^.

Viral upper respiratory tract infections (URTI) by pathogens like respiratory syncytial virus, rhinovirus, influenza, and adenoviruses are among the most notorious predisposing factors for OM infections^10–12^. The collective sequelae of URTI—compromised airway defenses, negative middle ear pressure, and inflammation, obstruction, and dysfunction of the Eustachian tube—permit translocation of nasopharynx secretions and commensal bacteria into the sterile middle ear space^13^. Single-species or polymicrobial biofilms formed by aggregates of bacteria and adherent to middle ear mucosa both contribute to OM pathogenesis and confer substantial recalcitrance to antimicrobials as reviewed by Silva and Sillankorva^14^. Furthermore, the high cell density in biofilms has been shown to promote horizontal gene transfer of antimicrobial resistance (AMR)^15^. Despite mechanistic understanding of biofilm resistance to antimicrobials^16,17^ and the inability of antibiotics to reach concentrations capable of eradicating biofilms in the middle ear^18^, broad-spectrum antibiotics remain the standard of care in pediatric OM due to their short-term efficacy^19^. This practice unquestionably contributes to rapid, global emergence of antibiotic-resistant bacteria^20^, as well as microbiome dysbiosis to the potential detriment of immune development and overall health^4^.

Although the shift in microbiology is quite complex and not described in full here, certain selective pressures such as decades of pneumococcal conjugate vaccination (PCV) and widespread antibiotic use have played a role in shifting the microbiology of OM, once dominated by group A *Streptococcus*, to a predominant trio of nasopharynx-resident opportunistic otopathogens: *Streptococcus pneumoniae*, *Moraxella catarrhalis*, and nontypeable *Haemophilus influenzae* (NTHi)^21^. Currently, NTHi is attributed to half of all bacterial AOM cases and considered the primary cause of recurrent and chronic OM^21^. However, all three species have demonstrated coexistence in polymicrobial biofilms throughout OM disease progression^22^. More worrisome still, this trio of otopathogens exhibit concerning AMR profiles to antibiotics routinely used in OM treatment^23–25^. In addition to AMR and minimal efficacy of antibiotics on OM biofilm-resident bacteria, well-documented side effects of broad-spectrum antibiotics, including secondary infections, diarrhea, rash outbreaks, and disruption of the microbiome, present an added level of concern^4,26^. Per these considerations, demand for the development of alternative, sustainable strategies to combat OM pathogenesis and best minimize or eliminate these adverse outcomes are imperative.

While preventative strategies remain an ideal goal, therapeutic interventions for existing chronic or recurrent OM infections, such as CSOM or PTTO, are of equal importance. We have demonstrated that monoclonal antibodies (mAb) against two unique NTHi biofilm-associated determinants either induces NTHI to disperse from a biofilm or significantly disrupts biofilm structural elements to cause collapse of NTHi and polymicrobial biofilm matrices, both of which result in subsequent release of resident bacteria from their protective fortress^27–33^. Furthermore, these newly released (NRel) bacteria are phenotypically distinct from planktonic or biofilm-resident counterparts, susceptible to clearance by host immunity, and, importantly, demonstrate a four to eightfold increase in antibiotic sensitivity relative to planktonic states (free living)^29,31^. The first mAb, anti-rsPilA, targets the majority subunit of the NTHi Type IV pilus (PilA) which has critical roles in adherence, motility, competence, and biofilm formation^34,35^. The second mAb, anti-tip-chimer peptide, targets protective epitopes within the α- & β-tip domains of the bacterial DNA-binding DNABII protein integration host factor (IHF) produced by NTHi which encodes a critical biofilm structural element required for integration of extracellular DNA (eDNA) into biofilm matrices^32^. Both anti-rsPilA^27,36^ and anti-tip-chimer peptide^30,36^ have proven efficacious in inducing the NRel state and subsequent resolution of experimental NTHi-induced OM in vivo. We envision clinical application of these mAb technologies in biofilm-mediated infections, such as CSOM or PTTO, to facilitate eradication by host immune effectors and a now-efficacious shortened regimen of lower-dosage antibiotics if necessary. While targeted clearance of recalcitrant middle ear biofilms is the mechanistic crux of these potential therapeutics, an absence of significant microbiome dysbiosis is a critical prerequisite for success of these technologies in pediatric clinical settings. Moreover, anti-tip-chimer is derived from the DNABII protein family which has widespread roles in biofilm architecture^37^, therefore, potential collateral damage to commensal biofilm-forming bacteria associated with healthy microbiotas must be investigated.

**Fig. 1:**
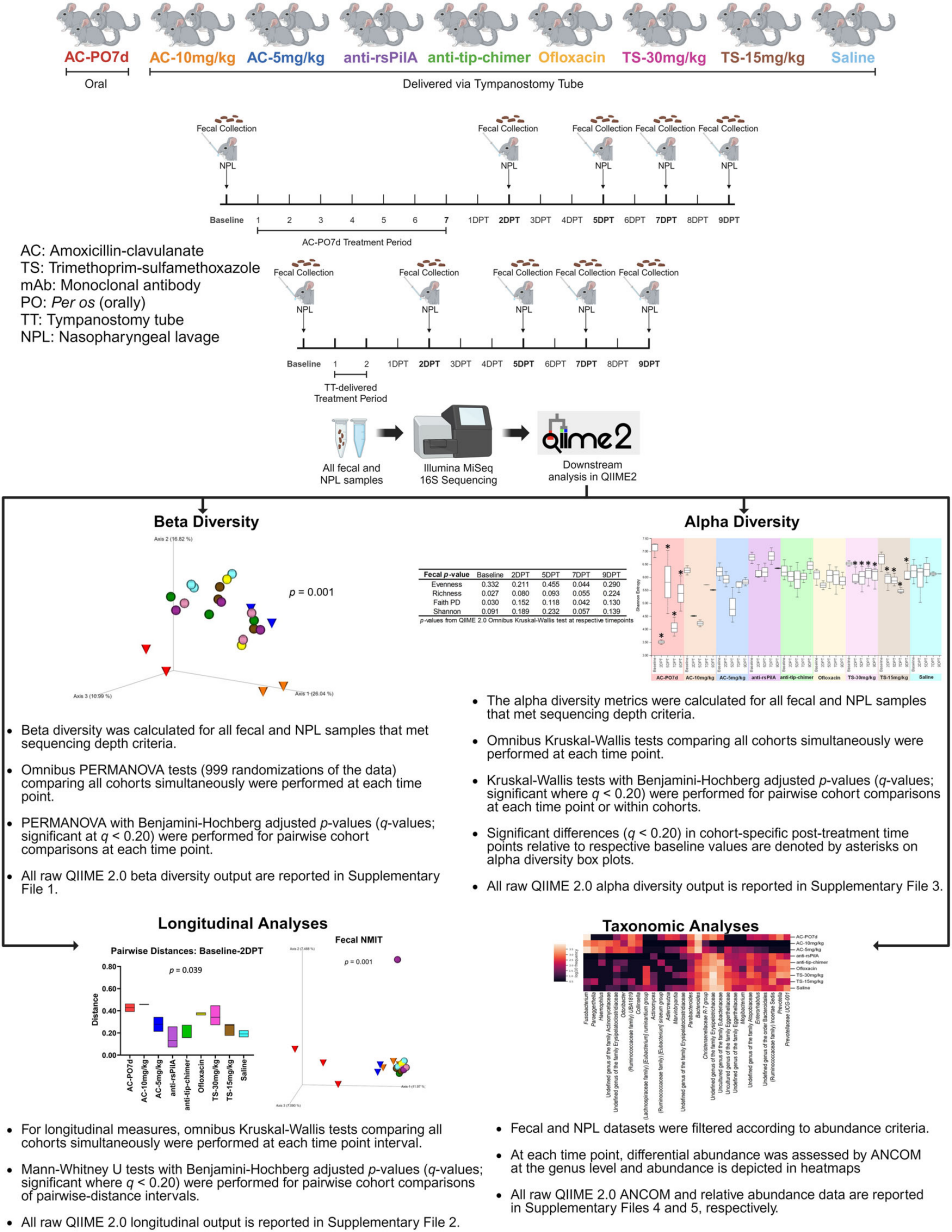
Experimental design schematic. Representation of experimental design and subsequent microbiome analyses. Created with BioRender.com.

We have previously shown that antibodies produced after subcutaneous and transcutaneous immunization with tip-chimer peptide do not alter the intestinal microbiome of chinchillas^4^. However, effects of clinically relevant TT delivery of anti-rsPilA and anti-tip-chimer mAbs on the microbiome have not yet been evaluated. To address this knowledge gap, nasopharyngeal lavage (NPL) and fecal samples were collected from chinchillas before and intermittently after antibiotic or mAb treatment to assess both nasal and gastrointestinal microbiome changes as fluids present in the middle ear can drain into the nasopharynx and subsequently the gut by way of the Eustachian tube and swallowing, respectively. Chinchillas were assigned to one of nine cohorts after TT insertion. One cohort received a week of twice daily oral amoxicillin-clavulanate (AC), reflective of standard OM care in children, and a positive control for antibiotic-mediated microbiome disruption. Alternatively and specifically toward the goal to develop a shorter-term yet more effective therapy for recalcitrant OM, other cohorts received a 2-day regimen of anti-rsPilA, anti-tip-chimer, AC, trimethoprim-sulfamethoxazole (TS), ofloxacin, or saline delivered via TT twice daily. For a summary of experimental design and analyses, see Fig. 1. As expected, oral AC treatment significantly impacted fecal diversity and differential abundance, in addition to unexpectedly analogous effects on NPL microbiomes. Shifts in the microbiome were also observed following TT delivery of AC, TS, and ofloxacin. Importantly, however, anti-rsPilA and anti-tip-chimer treatment did not significantly alter the microbiome, and instead, appeared similar to saline controls. These findings provide intriguing evidence of the effect of oral antibiotic administration on more proximal microbial environments like the nasopharynx, but more importantly, demonstrate that our anti-biofilm antibodies can be safely administered to healthy chinchillas without significant adverse effects on either fecal or nasopharyngeal microbiomes.

## Results

### 16S rRNA gene sequencing

In total, 126 fecal and 126 NPL samples were available and submitted for 16S sequencing and returned a total of 2,448,804 high-quality sequences (19,435 ± 1303) and 1,505,762 high-quality sequences (11,951 ± 1131), respectively. After amplicon sequence variant (ASV) prevalence filtering and rarefaction, diversity analyses included 117 fecal samples comprised of 2,268,884 reads (19,393 ± 1192) and 1101 total ASVs. The 120 NPL samples comprised of 1,055,094 reads (8793 ± 698) and 453 total ASVs. Differential abundance analyses (without rarefaction) included 123 fecal samples comprising 1,938,547 reads (15,761 ± 944) and 382 total ASVs. The 124 NPL samples comprised of 836,105 reads (6743 ± 550) and 140 total ASVs. Cohort fecal and NPL sample sizes across time points are detailed in Tables 1 and 2, respectively.

**Table 1:** Cohort sample sizes across time points for fecal diversity or differential abundance analyses.

Fecal diversity analysis	Baseline	2DPT	5DPT	7DPT	9DPT	Fecal differential abundance analysis	Baseline	2DPT	5DPT	7DPT	9DPT
AC-PO7d	3	2	3	3	2	AC-PO7d	3	2	3	3	2
AC-10 mg/kg	2	2	2	1	2	AC-10 mg/kg	2	3	2	1	2
AC-5 mg/kg	2	2	3	3	3	AC-5 mg/kg	2	3	3	3	3
Anti-rsPilA	3	3	2	2	2	Anti-rsPilA	3	3	2	2	2
Anti-tip-chimer	3	3	3	2	3	Anti-tip-chimer	3	3	3	3	3
Ofloxacin	3	2	3	3	3	Ofloxacin	3	3	3	3	3
TS-30 mg/kg	3	3	3	3	3	TS-30 mg/kg	3	3	3	3	3
TS-15 mg/kg	3	3	3	3	3	TS-15 mg/kg	3	3	3	3	3
Saline	3	3	3	2	1	Saline	3	3	3	3	2

All cohorts started with three chinchillas. Decreases in cohort size were due to failure to collect a sample, humane endpoint euthanasia or mortality, or rarefaction criteria (diversity analyses).

**Table 2:** Cohort sample sizes across time points for nasopharyngeal lavage diversity or differential abundance analyses.

NPL diversity analysis	Baseline	2DPT	5DPT	7DPT	9DPT	NPL differential abundance analysis	Baseline	2DPT	5DPT	7DPT	9DPT
AC-PO7d	3	3	3	2	2	AC-PO7d	3	3	3	2	2
AC-10 mg/kg	2	2	2	1	2	AC-10 mg/kg	3	3	2	2	2
AC-5 mg/kg	3	3	3	3	3	AC-5 mg/kg	3	3	3	3	3
Anti-rsPilA	3	3	2	2	2	Anti-rsPilA	3	3	2	2	2
Anti-tip-chimer	3	3	3	2	3	Anti-tip-chimer	3	3	3	3	3
Ofloxacin	3	3	3	3	2	Ofloxacin	3	3	3	3	2
TS-30 mg/kg	3	3	3	2	3	TS-30 mg/kg	3	3	3	2	3
TS-15 mg/kg	3	3	3	3	3	TS-15 mg/kg	3	3	3	3	3
Saline	3	3	3	3	2	Saline	3	3	3	3	2

All cohorts started with three chinchillas. Decreases in cohort size were due to failure to collect a sample, humane endpoint euthanasia or mortality, or rarefaction criteria (diversity analyses).

### Fecal beta diversity

**Fig. 2:**
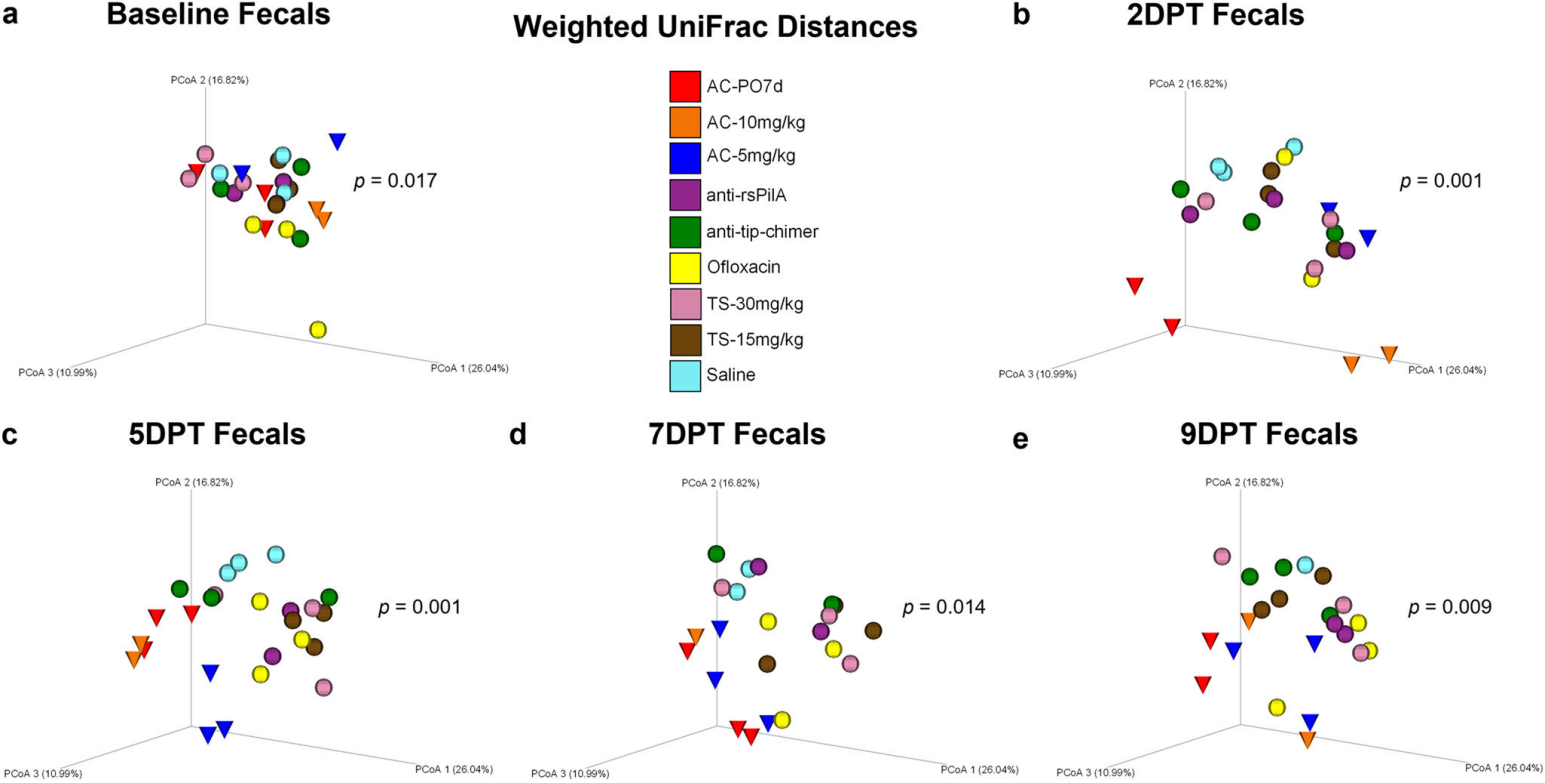
Fecal weighted UniFrac beta diversity. Principal coordinate analysis plots of weighted UniFrac distance matrices for fecal samples from all treatments at **a** baseline, **b** 2DPT, **c** 5DPT, **d** 7DPT, and **e** 9DPT. Differences in distances between treatments were analyzed in QIIME 2.0 by PERMANOVA with 999 randomizations of the data. Omnibus test PERMANOVA *P* values that tested for differences between the nine cohorts at each specified time point are displayed on respective graphs (significant where *P* < 0.05). Individual chinchillas are colored according to cohort treatment as depicted in the legend. Chinchillas in AC-treated cohorts are depicted as cones to emphasize separation from other cohorts which are depicted as spheres. Sample sizes are reported in Table 1.

Beta diversity, a measure of microbiome composition similarity or dissimilarity of two communities, was assessed using weighted UniFrac distances to compare all nine cohorts. Omnibus PERMANOVA tests were performed at each time point which compared all cohorts simultaneously to determine if there were differences between one or more of the nine cohorts, and *P* values from these tests are reported on respective principal coordinate analysis (PCoA) plots (Fig. 2). In addition, pairwise cohort comparisons were performed with permutational multivariate analysis of variance (PERMANOVA) and Benjamini–Hochberg correction for multiple testing to test for differences between each cohort relative to the others. Pairwise comparisons are reported as *q*-values (significant where *q* < 0.20) and are detailed in Supplementary File 1.

Baseline weighted UniFrac distances revealed fecal microbial composition differed between cohorts (*P* = 0.017; Fig. 2a) prior to the administration of treatments. While unique microbial communities across individuals are not uncommon, conventional beta diversity metrics were supplemented with longitudinal analyses to account for intraindividual variability over time and more precisely delineate treatment-specific effects on the microbiome. Despite baseline differences, antibiotic effects on beta diversity became evident at posttreatment time points, and differences in fecal weighted UniFrac distances were noted at 2DPT (*P* = 0.001), 5DPT (*P* = 0.001), 7DPT (*P* = 0.014) and 9DPT (*P* = 0.009; Fig. 2b–e**)**. As expected, an oral regimen of AC had dynamically disruptive effects on the gut microbiome per clear separation and clustering of AC-PO7d (red cones) away from TT-delivered treatments starting at 2DPT and persisting through 9DPT (Fig. 2). Separation and clustering of AC-10 mg/kg and AC-5 mg/kg (blue and orange cones, respectively) away from other TT-treated cohorts between 2DPT and 9DPT further delineated far-reaching impacts of broad-spectrum antibiotics and demonstrated that similar trends in microbiome disruption replicated whether AC was delivered orally or via TT (Fig. 2). Conversely, minimal changes in fecal beta diversity of mAb-treated cohorts (purple and green spheres) were observed across the experimental period which instead remained consistently clustered in the upper right quadrant of each weighted UniFrac PCoA. Pairwise comparisons of fecal weighted UniFrac distances between each cohort at posttreatment time points revealed AC-PO7d was significantly different from each of the eight TT-treated cohorts at 5DPT, including both TT-delivered AC cohorts, which conveyed the difference in magnitude of gut microbial disruption expected between oral and TT delivery of a broad-spectrum antibiotic (Benjamini–Hochberg adjusted *P* value of *q* < 0.20; Supplementary File 1). However, AC-10 mg/kg and AC-5 mg/kg were also different (*q* < 0.20) relative to all other TT-treated cohorts at 5DPT except anti-rsPilA. Importantly, neither mAb was significantly different from saline at any time point with the exception of 5DPT anti-rsPilA (*q* = 0.196; Supplementary File 1). Trends in posttreatment significance and separation of AC cohorts were similarly replicated in supplementary beta diversity metrics, Jaccard, Bray–Curtis, and unweighted UniFrac (Supplementary Figs. 1–3, respectively). Individual PCoA plots displaying cumulative changes across all time points for beta diversity metrics are available in Supplementary Fig. 4.

### Fecal longitudinal beta diversity analyses

**Fig. 3:**
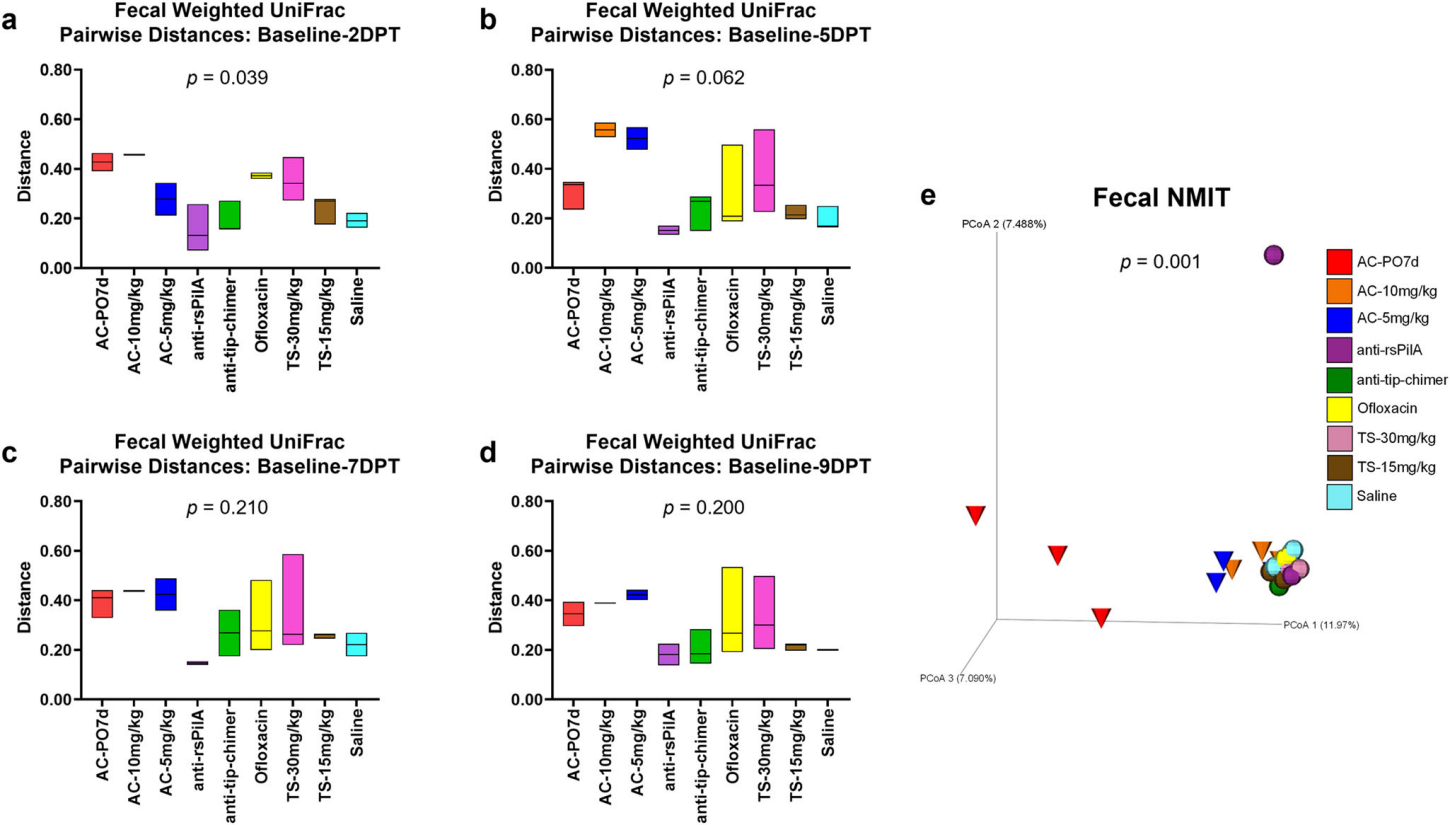
Fecal longitudinal beta diversity analyses. Longitudinal analysis of changes in fecal weighted UniFrac beta diversity pairwise-distances across posttreatment intervals **a** baseline—2DPT, **b** baseline—5DPT, **c** baseline—7DPT, and **d** baseline—9DPT. Omnibus tests for differences between any of the nine cohorts for each interval were performed with Kruskal–Wallis tests in QIIME 2.0 (*P* < 0.05) and *P* values for each interval are displayed on respective graphs. Floating bar charts denote minimum, maximum, and median values for pairwise-distances within cohorts between the respective time point intervals. Chinchillas without values for both time points in the assessed interval were not included in analysis. **e** Principal coordinate analysis plot of fecal nonparametric microbial interdependence test (NMIT) distances at the genus level for samples from all treatments. Individual cones (AC cohorts) or spheres (all other cohorts) represent the longitudinal change in microbial composition of each chinchilla (*n* = 27) across the experimental period. Differences in NMIT distances between treatments were analyzed in QIIME 2.0 by PERMANOVA with 999 randomizations of the data (*P* < 0.05) with *P* values displayed on respective graphs. Cohorts are colored according to treatment as denoted in the legend.

Sampling the microbiome of a single individual repeatedly over an experimental period, or longitudinally, provides valuable insight into overall microbiome stability as well as response and recovery from various treatments^38^. The longitudinal sample collection implemented in these studies, which consisted of a pre-treatment and four posttreatment time points, permitted a more thorough evaluation of the magnitude of change within an individual chinchilla’s microbiome in response to the treatment received. In addition, longitudinal analyses were useful to elucidate treatment effects that may have been influenced or otherwise masked by baseline differences between cohorts. All longitudinal analyses were evaluated by omnibus Kruskal–Wallis tests comparing all cohorts simultaneously to determine if there were differences between one or more of the nine cohorts, and *P* values from these tests are reported on respective graphs or PCoA plots (Fig. 3). Pairwise cohort comparisons with Mann–Whitney *U* tests and Benjamini–Hochberg correction for multiple testing were used to test for differences between each cohort relative to the others. Longitudinal pairwise comparisons are reported as *q*-values (significant where *q* < 0.20) and are detailed in Supplementary File 2.

For each chinchilla, pairwise-distances, a measure of longitudinal changes in beta diversity distances in an individual, were calculated to assess the magnitude of change between paired samples (from the same chinchilla) collected at baseline and each posttreatment time point for a total of four pairwise-distance intervals (i.e., baseline—2DPT, baseline—5DPT, baseline—7DPT, baseline—9DPT). Intervals with greater deviations from zero represented greater amounts of change within a treatment relative to baseline values. Magnitudes of change across intervals, attributable to antibiotic treatment effects, could then be compared between cohorts. In accordance with fecal beta diversity at isolated time points, weighted UniFrac pairwise-distances revealed that the magnitude of change over the baseline—2DPT interval significantly differed across cohorts (*P* = 0.039). Expectedly, AC-PO7d and AC-10 mg/kg had numerically higher values over this interval, indicative of more prominent changes in the microbiome relative to other treatments. Interestingly, effects of ofloxacin and TS-30 mg/kg that were not as apparent in weighted UniFrac PCoAs also became evident. Most importantly however, both anti-rsPilA and anti-tip-chimer displayed some of the lowest values over the baseline—2DPT interval and were most similar to saline (Fig. 3a). A reduction in pairwise-distance values over the baseline—9DPT interval would suggest a return to baseline microbiome equilibrium if prior shifts had occurred, however, consistently high pairwise-distance values were maintained across all intervals in AC cohorts and again emphasized lasting effects of broad-spectrum antibiotic usage on the gut microbiota. In contrast, anti-rsPilA and anti-tip-chimer fecal pairwise-distances remained consistent, low, and comparable to saline across all intervals (Fig. 3a–d). No pairwise comparisons between cohort pairwise-distances were significant (Supplementary File 2).

A nonparametric microbial interdependence test (NMIT) was used to longitudinally compare similarity across individuals as a function of temporal microbial composition. Pairwise correlations of genus-level taxa composition within each individual over time were used to compute between-subject distances, and ultimately, generate a single compressed value representative of each chinchilla’s longitudinal microbial interdependence. Similar clustering patterns suggest similar microbiome trajectories—or nature of change—over the experimental period, whereas distinct clustering suggests unique microbiome trajectories. Fecal NMIT definitively demonstrated that AC significantly altered microbial interdependence networks unique relative to TT-delivered antibiotics (*P* = 0.001) with especially remarkable separation of AC-PO7d from all cohorts. While oral AC effects were predictably more intense, lesser but still notable separation of both TT-delivered AC cohorts were similarly observed. The separation of the single anti-rsPilA (purple) sphere represents an individual chinchilla that showed signs of a suspected health problem (later confirmed) that led to its eventual removal from the study by 5DPT. Overall, non-AC cohorts, including mAbs, clustered tightly together and suggested a notably more minimal, or similar type, of overall change in the fecal microbiome (Fig. 3e).

### Nasopharyngeal lavage beta diversity

**Fig. 4:**
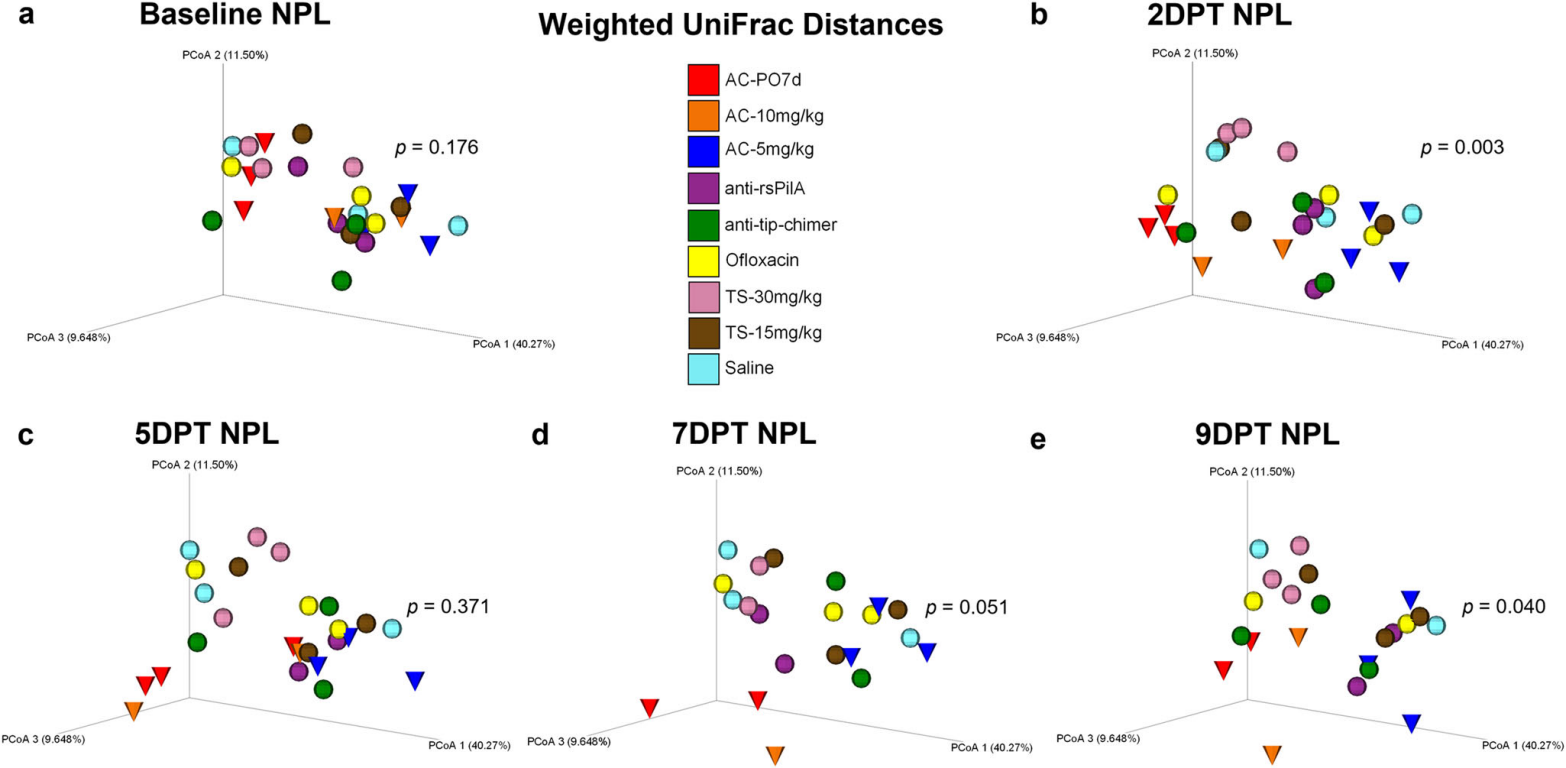
Nasopharyngeal lavage weighted UniFrac beta diversity. Principal coordinate analysis plots of weighted UniFrac distance matrices for NPL samples from all treatments at **a** baseline, **b** 2DPT, **c** 5DPT, **d** 7DPT, and **e** 9DPT. Differences in distances between treatments were analyzed in QIIME 2.0 by PERMANOVA with 999 randomizations of the data. Omnibus test PERMANOVA *P* values that tested for differences between the nine cohorts at each specified time point are displayed on respective graphs (significant where *P* < 0.05). Individual chinchillas are colored according to cohort treatment as depicted in the legend. Chinchillas in AC-treated cohorts are depicted as cones to emphasize separation from other cohorts which are depicted as spheres. Sample sizes are reported in Table 2.

Beta diversity in NPL samples was analyzed and is presented in a manner identical to that specified for fecal sample beta diversity analysis above. No differences in NPL microbiomes were observed at baseline. However, as in feces, NPL samples exhibited omnibus differences in 2DPT (*P* = 0.003) and 9DPT (*P* = 0.040) weighted UniFrac distances (Fig. 4b, e). Little change in temporal NPL clustering patterns were observed for any cohort except AC-PO7d and AC-10 mg/kg (red and orange cones, respectively) which demonstrated noticeably unique clustering at each time point (Fig. 4). Clustering of AC-5 mg/kg, which separated less distinctly than other AC cohorts, suggested a dose-dependent effect of TT-delivered AC on the nasopharyngeal microbiome. Although pairwise comparisons between cohorts did not significantly differ (Supplementary File 1), distinct clustering patterns and omnibus significance suggest that oral administration of AC disrupted the superiorly located nasopharyngeal microbiome and that all three AC treatments exerted prolonged effects on NPL beta diversity. Similar trends in significance were observed for supplemental beta diversity metrics, Jaccard and Bray–Curtis, but not unweighted UniFrac (Supplementary Figs. 5–7, respectively). Cumulative changes in NPL beta diversity metrics are provided in Supplementary Fig. 8. In general, weighted UniFrac changes appeared to be less extreme in nasopharyngeal microbiomes than observed in feces. More importantly, however, neither mAb-treated cohort exhibited notable shifts in NPL weighted UniFrac clustering over the experimental period, recapitulated fecal findings, and showed a lack of mAb-associated microbiome disruption.

### Nasopharyngeal lavage longitudinal beta diversity analyses

**Fig. 5:**
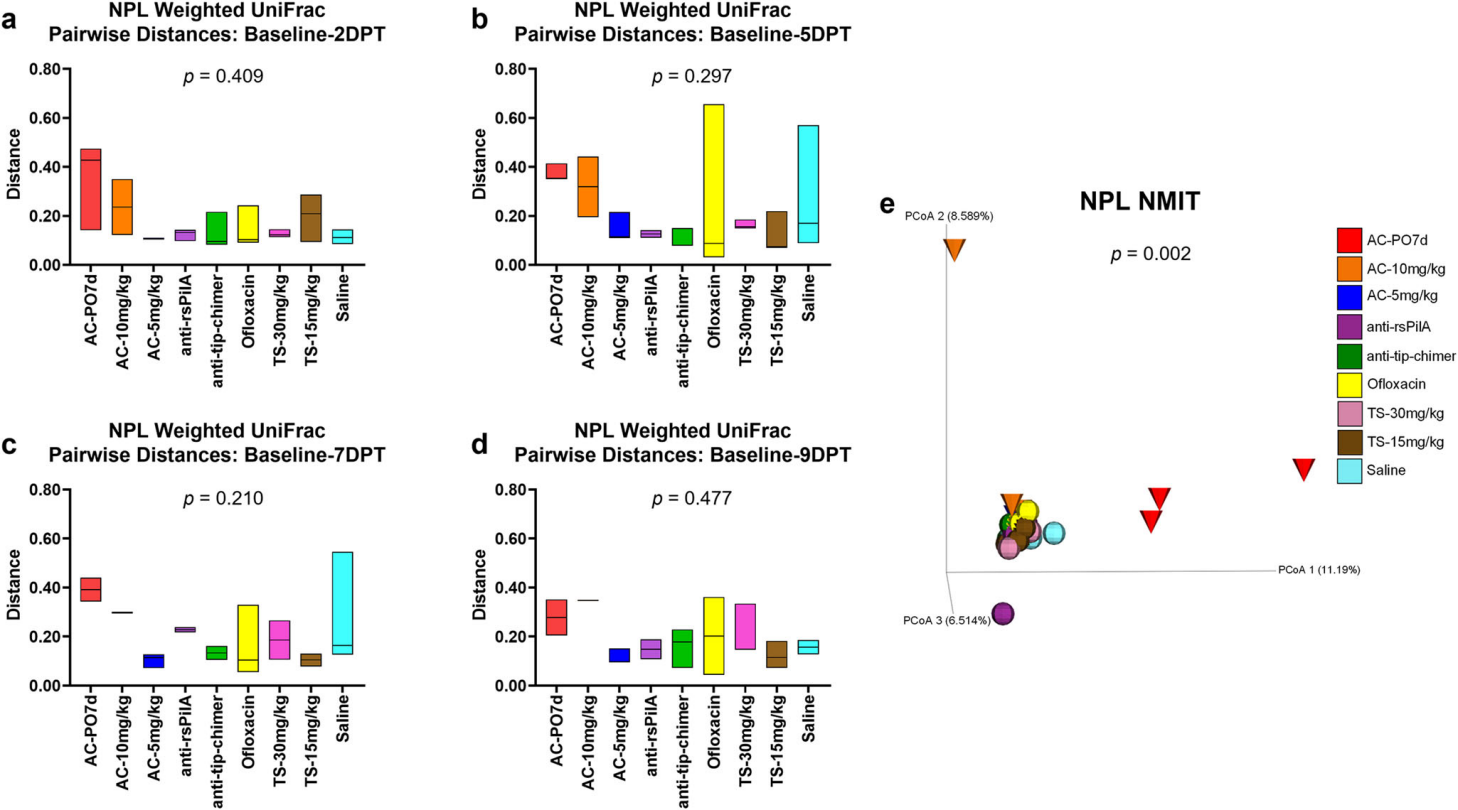
Nasopharyngeal lavage longitudinal beta diversity analyses. Longitudinal analysis of changes in NPL weighted UniFrac beta diversity pairwise-distances across posttreatment intervals **a** baseline—2DPT, **b** baseline—5DPT, **c** baseline—7DPT, and **d** baseline—9DPT. Omnibus tests for differences between any of the nine cohorts for each interval were performed with Kruskal–Wallis tests in QIIME 2.0 (*P* < 0.05) and *P* values for each interval are displayed on respective graphs. Floating bar charts denote minimum, maximum, and median values for pairwise-distances within cohorts between the respective time point intervals. Chinchillas without values for both time points in the assessed interval were not included in the analysis. **e** Principal coordinate analysis plot of NPL nonparametric microbial interdependence test (NMIT) distances at the genus level for samples from all treatments. Individual cones (AC cohorts) or spheres (all other cohorts) represent the longitudinal change in the microbial composition of each chinchilla (*n* = 27) across the experimental period. Differences in NMIT distances between treatments were analyzed in QIIME 2.0 by PERMANOVA with 999 randomizations of the data (*P* < 0.05) with *P* values displayed on respective graphs. Cohorts are colored according to treatment as denoted in the legend.

Longitudinal analyses performed on NPL samples were analyzed and presented in a manner identical to that specified for longitudinal fecal sample analyses above. Weighted UniFrac pairwise-distances in NPL samples were numerically greater for AC-treated cohorts, similarl to fecal samples, but no differences were detected via omnibus tests or through pairwise comparisons between cohorts (Fig. 5a–d and Supplementary File 2, respectively). Overall, anti-rsPilA and anti-tip-chimer exhibited low and overall stable pairwise-distances across the intervals, indicating minimal changes from baseline. Longitudinal NMIT analysis of NPL samples (Fig. 5e) showed the same distinctly clustered nasopharyngeal microbiome trajectory for AC-PO7d as observed in fecal samples, but the separation of AC-10 mg/kg and AC-5 mg/kg was less apparent in the nasopharynx. The separation of a single anti-rsPilA purple sphere and AC-10 mg/kg orange cone represent individuals that had suspected health problems or were found as mortalities leading to removal from the study by 5DPT. Overall, AC was most disruptive to nasopharyngeal microbiomes, whereas mAbs did not lead to significant microbiome disruption relative to baseline compositions.

### Fecal alpha diversity

**Fig. 6:**
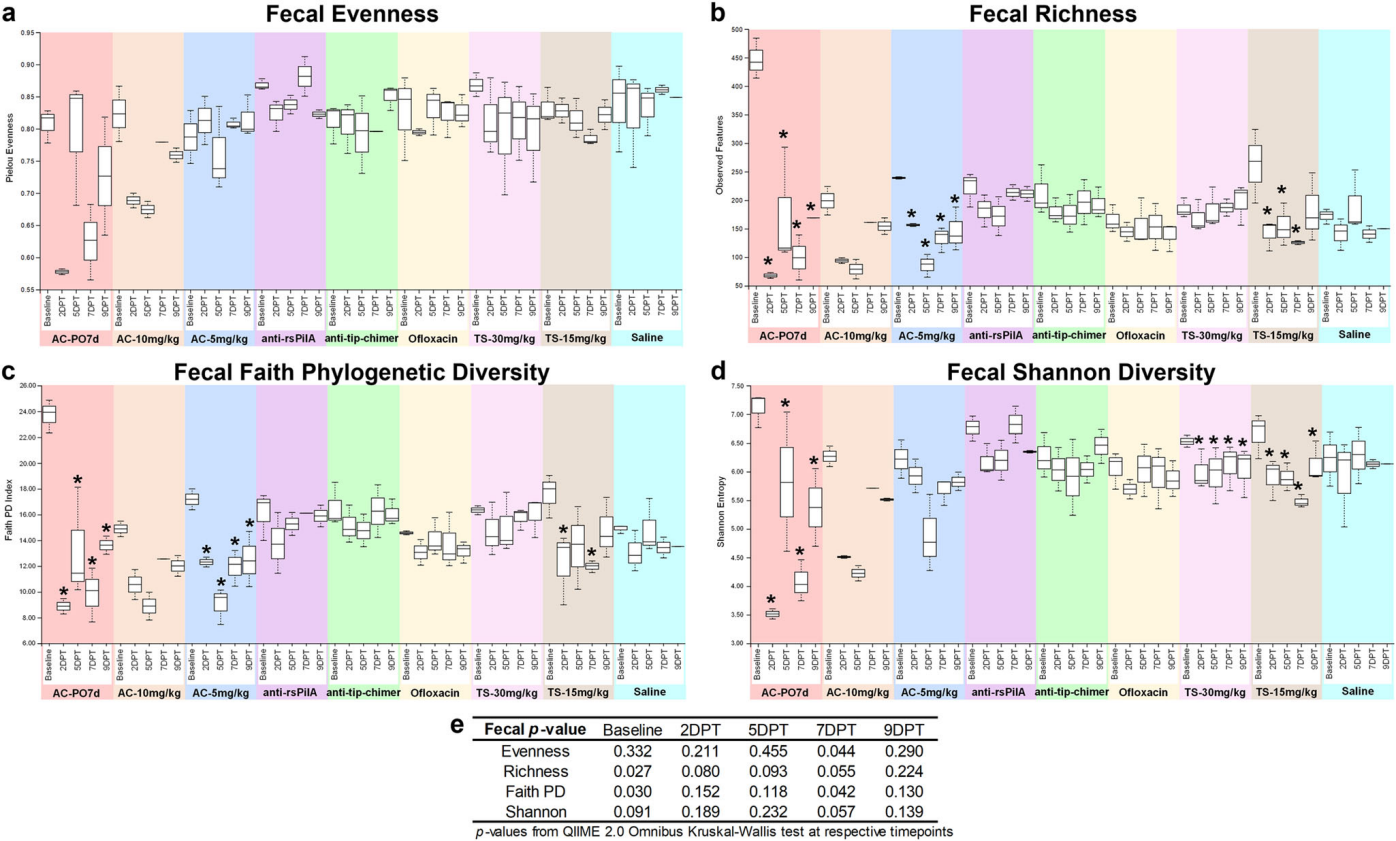
Fecal alpha Diversity. Measures of alpha diversity within fecal samples based on **a** evenness, **b** richness, **c** Faith phylogenetic diversity, and **d** Shannon diversity. **e** Omnibus tests for differences between any of the nine cohorts at each time point were performed with the Kruskal–Wallis tests in QIIME 2.0 (significant where *P* < 0.05). Values are represented as box and whisker plots where boxes denote interquartile range and upper and lower whiskers denote minimum and maximum, respectively. Each plot is organized by time point within the treatment. Asterisks denote significant pairwise comparisons (*q* < 0.20) between posttreatment time points and respective baseline values within individual cohorts. Sample sizes are reported in Table 1.

Alpha diversity, a measure of microbiome composition within an individual sample, was assessed in each chinchilla across the sampled time points with measures of evenness, richness, Faith phylogenetic diversity (Faith PD), and Shannon diversity (Fig. 6). Omnibus test differences were analyzed with Kruskal–Wallis tests at each time point to determine whether any of the nine cohorts differed at a given time point. Pairwise comparisons between cohorts were assessed with Kruskal–Wallis tests and Benjamini–Hochberg correction for multiple testing to determine which specific cohorts differed from one another at each time point. Finally, pairwise comparisons were made within each cohort to assess whether alpha diversity within an individual cohort at 2DPT, 5DPT, 7DPT, or 9DPT differed from that same cohort’s baseline value. All alpha diversity pairwise comparisons are reported as *q*-values (significant where *q* < 0.20) and are detailed in Supplementary File 3.

Omnibus test differences at baseline were observed for baseline fecal alpha richness (*P* = 0.027) and Faith PD (*P* = 0.030; Fig. 6e), however, differences more likely to be attributed to treatment were noted for 7DPT evenness (*P* = 0.044) and Faith PD (*P* = 0.042; Fig. 6e). Excitingly, alpha diversity in mAb-treated samples remained relatively consistent through 9DPT and exhibited minimal changes (Fig. 6a–d). Differences in pairwise comparisons between cohorts at posttreatment time points were only observed for fecal evenness and Shannon diversity at 7DPT (*q* < 0.20; Supplementary File 3) and were significantly lower in AC-PO7d relative to all cohorts except AC-10 mg/kg. Notably, when compared against saline, neither monoclonal antibody significantly differed.

It is not uncommon to observe variations in microbiome composition across healthy individuals of any species; however, due to baseline differences observed here that could potentially confound comparisons of treatment-mediated effects across cohorts, pairwise comparisons of alpha diversity were performed within each cohort for additional statistical perspective. Within-cohort comparisons allowed a more direct assessment of independent treatment effects and microbiome changes respective to that cohort’s baseline. Comprehensive fecal pairwise comparisons between all combinations of sampling time points within a cohort are available in Supplementary Table 1, and significant pairwise comparisons made specifically between a cohort’s posttreatment time points and respective baseline are represented as asterisks on fecal alpha diversity figures (Fig. 6a–d). Treatment-associated decreases in alpha diversity most consistently replicated in AC-PO7d, which had significantly lower (*q* < 0.20) fecal richness, Faith PD, and Shannon diversity at all time points relative to baseline (Fig. 6b–d). The same reduction (*q* < 0.20) in fecal alpha diversity across all time points relative to baseline was observed in AC-5 mg/kg richness and Faith PD (Fig. 6b, c), and in Shannon Diversity for both TS cohorts (Fig. 6d). In addition, relative to baseline, TS-15 mg/kg exhibited decreases (*q* < 0.20) in richness (2DPT, 5DPT, and 7DPT; Fig. 6b) and Faith PD (2DPT and 7DPT; Fig. 6c). These results demonstrated TT delivery of AC and TS could disrupt the gut microbiome as observed with oral AC, despite the more distant and somewhat less direct path between the middle ear and the gut. Moreover, mAb cohorts remained statistically unchanged relative to respective baselines for all fecal alpha diversity metrics.

Pairwise-differences, which measure longitudinal changes in alpha diversity, revealed a significant omnibus test difference for fecal richness over the baseline-5DPT interval (*P* = 0.039; Supplementary File 2). While direct pairwise comparisons between cohorts were insignificant over this interval (*q* > 0.20), an average of 274 features in AC-PO7d, 120 features in AC-10 mg/kg, 162 features in AC-5 mg/kg, and 107 features in TS-15 mg/kg were lost relative to respective baseline richness versus an average relative loss of less than 68 features in all remaining cohorts including mAbs (Supplementary File 2). Collectively, fecal alpha diversity results indicate that oral and TT delivery of AC have significantly disruptive effects on the gut microbiome. In addition, TS, an OM-relevant antibiotic administered to those with penicillin allergies, can also alter the fecal microbiome. As opposed to these standard-of-care antibiotics, neither mAb lead to changes in fecal alpha diversity.

### Nasopharyngeal lavage alpha diversity

**Fig. 7:**
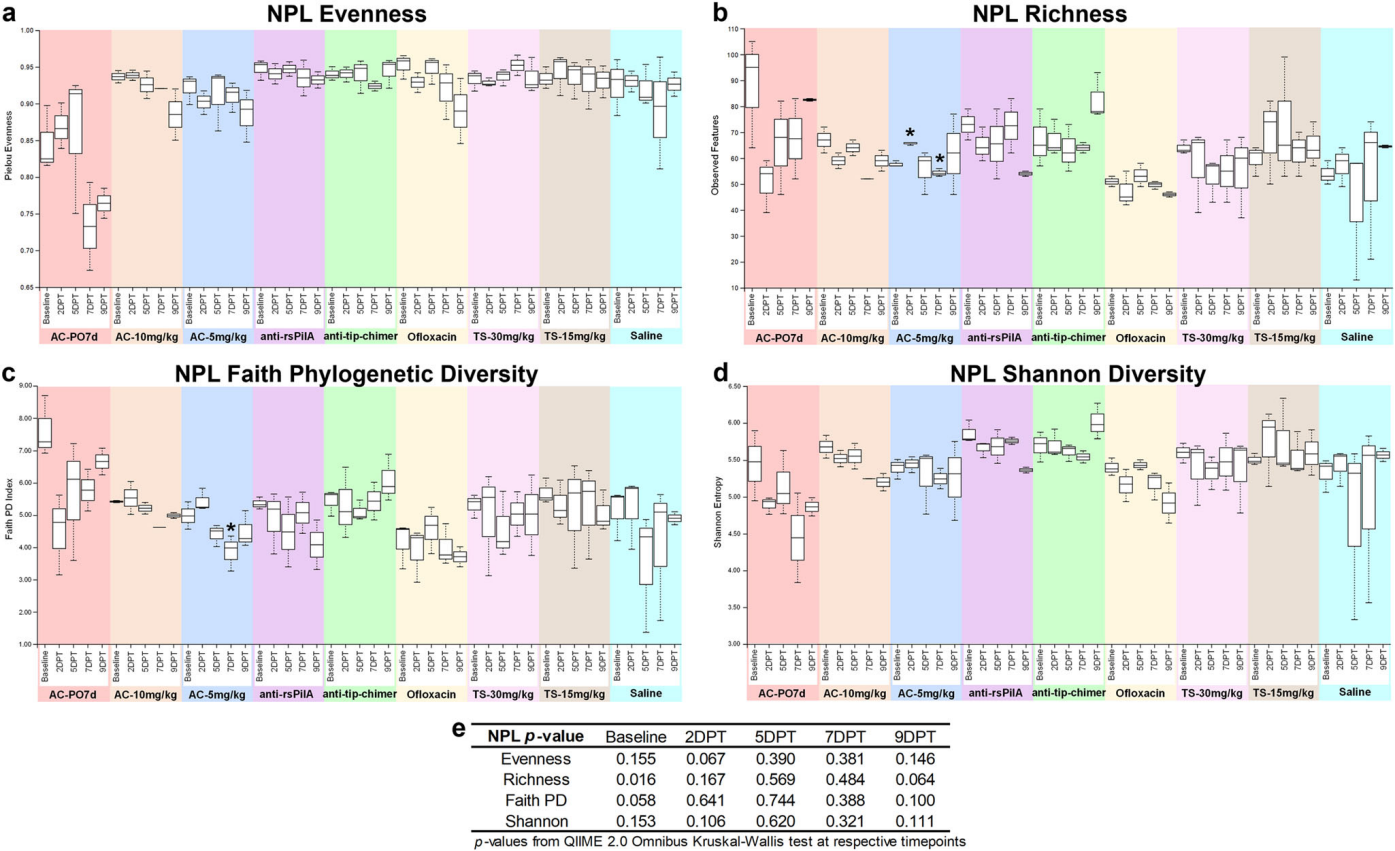
Nasopharyngeal lavage alpha diversity. Measures of alpha diversity within NPL samples based on **a** evenness, **b** richness, **c** Faith phylogenetic diversity, and **d** Shannon diversity. **e** Omnibus tests for differences between any of the nine cohorts at each time point were performed with the Kruskal–Wallis tests in QIIME 2.0 (significant where *P* < 0.05). Values are represented as box and whisker plots where boxes denote interquartile range and upper and lower whiskers minimum and maximum, respectively. Each plot is organized by time point within the treatment. Asterisks denote significant pairwise comparisons (*q* < 0.20) between posttreatment time points and respective baseline values within individual cohorts. Sample sizes are reported in Table 2.

Alpha diversity in NPL samples was analyzed and is presented in a manner identical to that specified for fecal sample alpha diversity analysis above. Baseline differences in NPL alpha diversity were limited to richness (*P* = 0.016), and no omnibus test differences were observed at posttreatment time points. Pairwise comparisons between all cohorts are reported in Supplementary File 3. Comprehensive NPL pairwise comparisons between all combinations of sampling time points within a cohort are available in Supplementary Table 2, and significant pairwise comparisons made specifically between a cohort’s posttreatment time points and respective baseline are represented as asterisks on NPL alpha diversity figures (Fig. 7a–d**)**. Surprisingly, all pairwise comparisons of AC-PO7d relative to baseline were insignificant, likely due to intraindividual nasopharyngeal microbiome variability that was observed even in saline controls (Fig. 7). Within-cohort pairwise comparisons relative to baseline were only significant for AC-5 mg/kg which had increased richness at 2DPT (*q* = 0.116; Fig. 7b) followed by reductions in 7DPT richness and Faith PD (*q* < 0.20; Fig. 7b, c). The diversity of mAb cohorts did not differ from the baseline at any point and remained relatively consistent (Fig. 7a–d). No differences were observed for NPL longitudinal pairwise-differences (Supplementary File 2).

### Taxonomic abundances in fecal samples

**Fig. 8:**
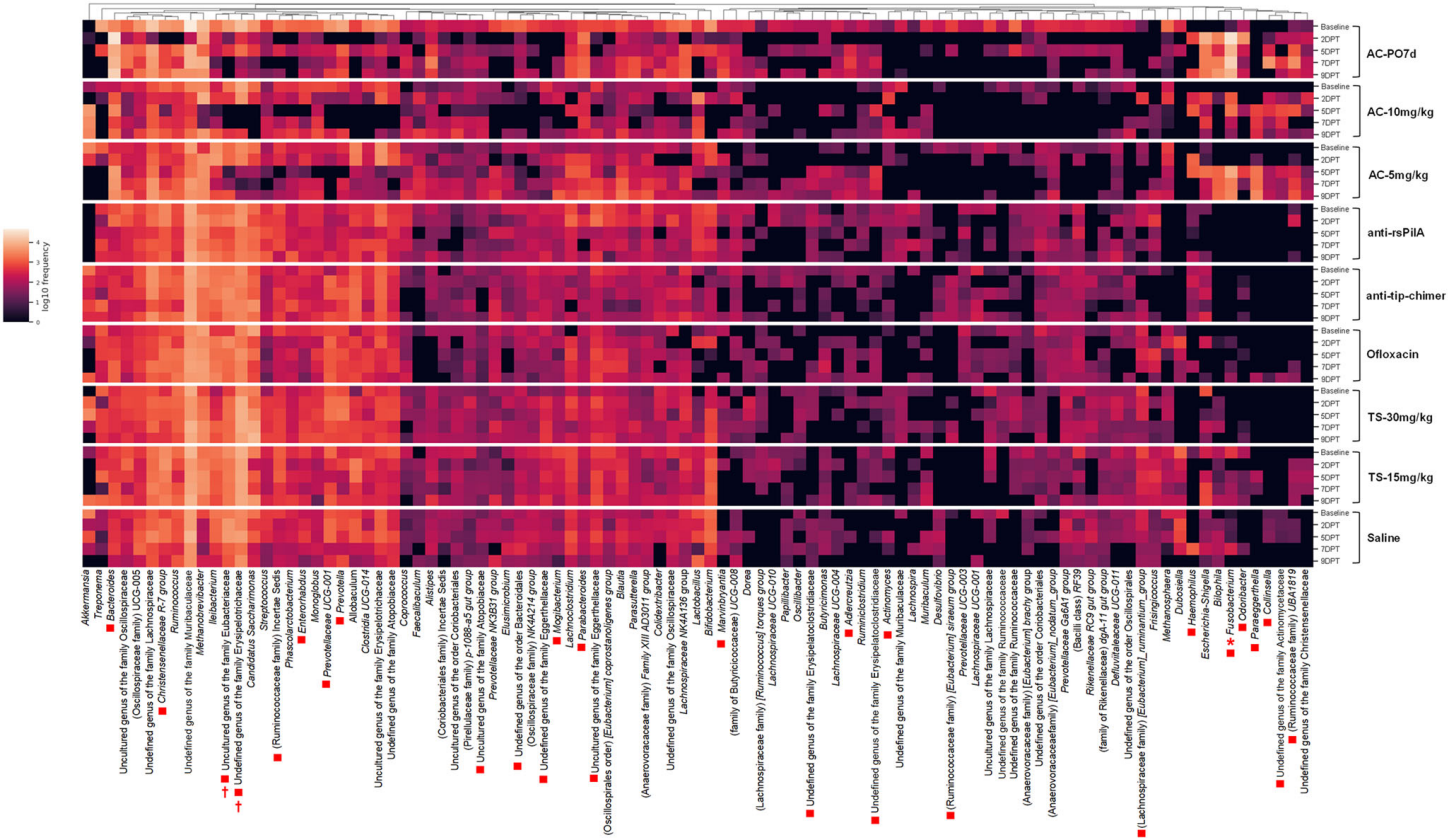
Fecal taxa frequency at the genus level. Heatmap denotes the mean log10 frequency of genus-level taxa observed in fecal samples at each time point, within treatment. Darker boxes denote lower prevalence, and lighter boxes denote greater prevalence. Significant taxa per ANCOM at 2DPT (*), 5DPT (■), and 7DPT (†) are depicted by specified symbols. No significant differences were observed for baseline or 9DPT genera. Sample sizes are reported in Table 1.

To assess the effects of antibiotics or mAb on microbiome community makeup, differential abundance was analyzed with ANCOM which compared all nine cohorts simultaneously at the genus level across time points. All ANCOM output is available in Supplementary File 4. Heatmaps were generated depicting the observed frequency (total ASV counts) of all classified fecal genera (Fig. 8) with significant features denoted by asterisks (2DPT), squares (5DPT), or crosses (7DPT). No fecal genera were significant at baseline or 9DPT. Heatmaps for each posttreatment time point detailing only significant features are available in Supplementary Fig. 9. For both fecal and NPL samples, ASV counts and relative abundances within cohorts are provided in Supplementary File 5.

At 2DPT, *Fusobacterium* was the only differentially abundant genus observed in fecal samples with an approximately tenfold increase in AC-PO7d (relative abundance: 34.98%) and enrichment in AC-10 mg/kg (3.33%) relative to other cohorts (0–0.03%). Differences in *Fusobacterium* persisted through 5DPT with AC-PO7d (16.13%), AC-10 mg/kg (10.53%), and AC-5 mg/kg (20.78%) having greater abundance than other cohorts (<0.05%). Similarly, *Bacteroides* was significantly elevated in all three AC cohorts by 5DPT (12.59–17.55%) compared with others (<1.90%; Fig. 8). Additional notable fecal genera significant at 5DPT included C*hristensenellaceae R-7 group* and unreported genera of Erysipelotrichaceae and Eubacteriaceae which had lower abundances in AC cohorts (0.08–1.15%; 0–2.80%; and 0–0.71%, respectively) than non-AC cohorts (2.39–6.11%; 6.35–19.81%; and 2.85–10.13%, respectively). Interestingly, *Paraeggerthella* and *Haemophilus* were different at 5DPT and were specifically enriched in TT-delivered AC-10 mg/kg (3.72% and 5.70%, respectively) and AC-5 mg/kg (5.44% and 3.31%, respectively) whereas these taxa were present at less than 0.25% in all other cohorts. Other significant genera at 5DPT were found at very low abundance across all cohorts (on average 0.66% relative abundance) and are detailed in Supplementary Fig. 9b. At 7DPT, undefined genera of Erysipelotrichaceae and Eubacteriaceae remained significantly lower in all AC-treated cohorts (0.11–0.06%) while other cohorts harbored 3.13–8.74% of undefined Eubacteriaceae and 7.80–17.98% undefined Erysipelotrichaceae, the latter of which was particularly prominent (>17%) in TS cohorts (Fig. 8).

### Taxonomic abundances in NPL samples

**Fig. 9:**
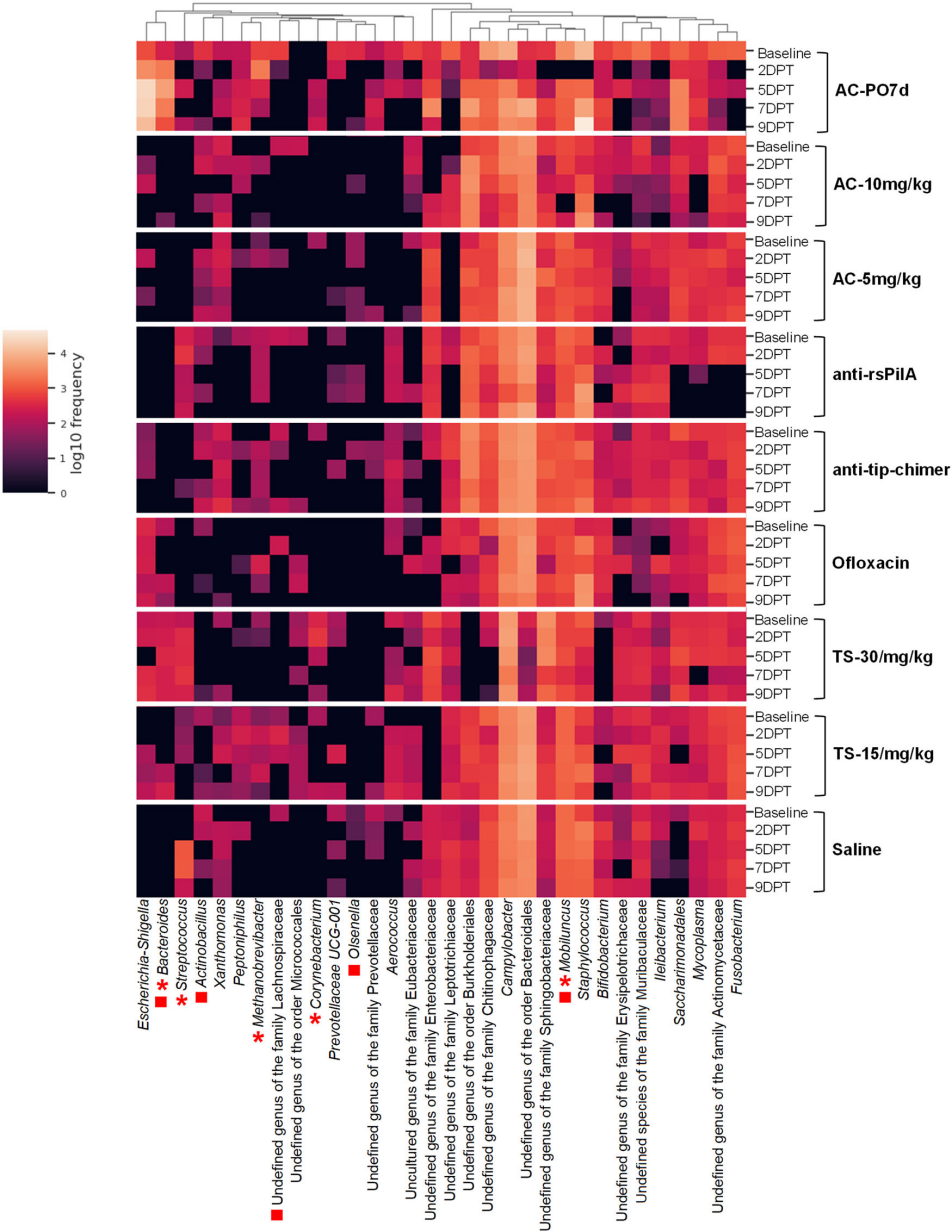
Nasopharyngeal lavage taxa frequency at the genus level. Heatmap denotes the mean log10 frequency of genus-level taxa observed in NPL samples at each time point, within the treatment. Darker boxes denote lower prevalence, and lighter boxes denote greater prevalence. Significant taxa per ANCOM at 2DPT (*), 5DPT (■), and 7DPT (†) are depicted by specified symbols. No significant differences were observed for baseline or 9DPT genera. Sample sizes are reported in Table 2.

As in fecal samples, ANCOM analysis was used to assess taxonomic abundance in NPL samples. In 2DPT NPL samples, *Bacteroides* were significantly more abundant in AC-PO7d (21.00%) and absent from all other cohorts except TS-30 mg/kg (1.74%). *Methanobrevibacter* was also significantly higher in AC-PO7d (21.69%) relative to other cohorts (0–1.34%), while *Mobiluncus* was absent in AC-PO7d (0%) but detectable in all other TT-treated cohorts (4.22–10.02%). Differences in both *Bacteroides* and *Mobiluncus* persisted at 5DPT with *Bacteroides* exclusively found in AC-PO7d (12.16%) and TS-30 mg/kg (2.48%), and *Mobiluncus* less abundant in AC-treated cohorts (2.35–3.90%) relative to other TT-treated cohorts (5.19–11.53%; Fig. 9). Other significant 5DPT genera were driven by differences in abundances between 0 and 1% in comparative cohorts and are detailed in Supplementary Fig. 9e. No significant ANCOM genus-level differences were detected at 7DPT or 9DPT.

For both fecal and NPL taxonomic abundance, additional heatmaps were generated with hierarchical clustering (not organized by time point within cohort as in Figs. 8 and 9) so that cohort time points that exhibited similar frequency of taxa would be grouped by dendrogram branches and located closer together on the y-axis. Dendrogram branches clearly delineated differences between post-baseline AC-PO7d time points and all other treatments in both sample types (Supplementary Figs. 10 and 11). In fecal samples particularly, there were also clear similarities and dendrogram grouping of post-baseline AC treatments, both oral (2DPT onward) and TT-delivered (5DPT onward) which were separated from all other treatments. Moreover, both mAb treatments clustered together regardless of time point (Supplementary Fig. 10). Collectively, these findings further support our conclusions of significant microbiome disruption associated with AC treatment in contrast to minimal influences attributed to mAb treatments.

## Discussion

Antibiotics are the most frequently prescribed drugs in children^39^ with an estimated 20-50% of these prescriptions reflecting inappropriate or unnecessary use^40^. Furthermore, OM is a leading cause of primary care visits with 80% of consultations resulting in prescriptions of broad-spectrum antibiotics^41^. Not only does this practice contribute to the global epidemic of continually emerging antibiotic-resistant bacteria, but significantly disrupts microbiome equilibrium to the detriment of recipient health^4,24^. A 2019 CDC report estimated that AMR contributed to an annual excess of 2.8 million infections, 35,000 deaths, and $13.65 billion in direct medical costs within the U.S. alone^42^. Though importance of antibiotic treatment in many cases of OM cannot be denied^7,43^, neither can staggering rates of AMR in key OM otopathogens. Over 95% of today’s *M*. *catarrhalis* strains express β-lactamases^23^ which significantly contribute to recalcitrance of polymicrobial OM biofilms^22^. In 2022, Gavrilovici et al.^24^ reported 82.85% and 91.42% of *S. pneumoniae* isolated from children with AOM were multidrug or TS-resistant, respectively, and 23.5% of *H. influenzae* were ampicillin-resistant. Another study from Taiwan found pediatric OM middle ear fluid harbored NTHi strains resistant to ampicillin (80.3%), AC (18%), and TS (68.9%)^44^. Similarly sobering increases in NTHi resistance profiles have been reported globally^44–48^, although levels of resistance tend to vary geographically and likely reflect differences in healthcare accessibility, PCV vaccination rates, and region-specific lifestyle or risks^24^.

Concerns surrounding AMR are substantially compounded by the frequent tendency of these OM-relevant bacteria to aggregate into sophisticated and recalcitrant-to-treatment polymicrobial biofilms with markedly increased antibiotic tolerance^49,50^. Therefore, in conditions such as CSOM^4^, OME^4^, and PTTO^51^ wherein chronic and recalcitrant biofilms are hallmarks of disease, biofilm-targeted therapeutics in lieu of antibiotic treatment could directly address disease etiology with fewer adverse effects. To this end, our mAbs were designed to target either an essential adhesin/motility factor or an integral biofilm structural component, both of which elicit release of formerly biofilm-resident bacteria with a distinct NRel phenotype into the environmental milieu. Biofilm dissemination by anti-rsPilA is mediated through disruption of NTHi adherence and induction of quorum sensing mechanisms that result in NTHi twitching out of associated biofilm matrices and eventual biofilm dispersion^52^. Alternatively, anti-tip-chimer peptide targets IHF (a DNABII protein) within the surrounding extracellular milieu and drives an equilibrium shift prompting release of biofilm matrix-bound IHF and subsequent collapse of the biofilm’s eDNA scaffolding^52^. When evaluated against single and multi-genera biofilms in vitro, Jurcisek et al.^53^ concluded that anti-rsPilA acted specifically on NTHi and NTHi co-partnered biofilms, whereas anti-tip-chimer peptide disrupted all single and multi-genera biofilms. Per these findings, we investigated whether mAb treatment might evoke collateral damage in healthy nasopharyngeal or intestinal microbiomes in vivo when administered via a clinically relevant route of delivery (e.g., via a surgically inserted TT or a tympanic membrane perforation as occurs from severe OM). Excitingly, results presented here showed no evidence that our mAbs induced significant microbiome alterations as opposed to markedly contrasted results in cohorts that received antibiotics representative of OM standard of care. Of note, only a single concentration of mAb (20 µg over the course of treatment, equivalent to ~0.03 mg/kg) was tested in this study based on our previously demonstrated findings that delivery of 5–10 µg of mAb per ear in the chinchilla host was very efficacious against a controlled challenge dose-mediated infection with a single human pathogen^30,32,33,54,55^. Importantly, in contrast to chinchillas used in this study which lacked ongoing active infection, OM infections in humans would be attributed to a much greater, likely polymicrobial, bacterial load in the middle ear that would presumably necessitate greater doses of mAb for clinical use. This is currently being investigated with a dose-escalation study in our ongoing human trials (NCT 05629741) wherein healthy volunteers will be receiving between 2.5 and 30 mg/kg based on pre-clinical data.

While AC is not typically administered directly into the middle ear, two TT-delivered AC cohorts were incorporated here as pilot cohorts for future studies because fluids placed into the middle ear can drain into the nasopharynx via the Eustachian tube where they are then swallowed, as occurs with oral delivery. As previously mentioned, our mAbs mediate release of NRel bacteria that exhibit increased susceptibility to antibiotic-mediated killing even over free-living cultured bacteria thus permitting significantly lower doses and shorter regimens of antibiotics to clear infections. Preliminary findings show that these mAb-released NRel are particularly susceptible to AC^52^, therefore, TT-delivered cohorts were included to assess dosages at which AC could be delivered subsequent to mAb treatment to aid in the clearance of NRel without microbiome disruption during in vivo OM challenge. As demonstrated by our results, the dose of TT-delivered AC requires further optimization, as just 2 days of 5 mg/kg was sufficient to induce significant nasopharyngeal and gut microbiome disruption. Other antibiotics included in this study, ofloxacin and TS, represent standard-of-care antibiotics in the treatment of OM which are routinely administered via TT or orally, respectively. However, as with AC, TT delivery of TS was piloted here in preparation for future studies. Relative to AC cohorts, our results showed ofloxacin and TS caused significant but more tempered effects on nasopharyngeal and gut microbiome disturbances. This outcome was not entirely unexpected due to the refinement of optimal doses and concentrations of these drugs to optimize host tolerance, drug efficacy, and minimize previously characterized antibiotic-mediated side effects^56,57^. Of paramount importance, however, there is no evidence that administration of mAbs resulted in changes in microbial diversity, composition, or overall homeostasis and further, have been shown to be highly effective in three distinct pre-clinical animal models of human disease wherein no additional antibiotics were delivered^30,33,54,58–60^.

Beyond concerns of AMR, pediatric antibiotic use and resultant microbiome disruption are associated risk factors for multiple health complications that include allergies and asthma^61^, juvenile idiopathic arthritis^62^, obesity^63^, inflammatory intestinal disorders^62,64^, and psychiatric or behavioral conditions^65,66^. Differences in alpha and beta diversity metrics denote that perturbations in diversity of antibiotic-treated cohorts were driven not only by losses but also uneven representation of select microbiome constituents over time^67,68^. Indeed, fecal samples recovered after 7 days of oral AC had a predominance of *Bacteroides* and *Fusobacterium* post treatment that persisted after cessation of antibiotics and mimicked our previous findings of antibiotic-mediated chinchilla microbiome disruption^4^. Increases in *Bacteroides*, *Escherichia–Shigella*, and *Fusobacterium* have previously been reported in adults treated with AC, and have been linked to the enrichment of certain AMR genes and potential opportunistic infections^69,70^. The decrease in fecal Erysipelotrichaceae observed here is also consistent with previous reports in humans treated with amoxicillin^71^. Although the biological importance of this decrease is not clear, lower abundance of this family has been associated with chronic or new onset of intestinal inflammation in humans^72^. Although none of the animals in this study were experimentally infected, *Haemophilus* was enriched in 2DPT fecal microbiomes of TT-delivered AC cohorts. While expected in NPL rather than fecal samples, increased intestinal prevalence of *Haemophilus* has been correlated with psychosis in patients with schizophrenia^73^ and Crohn’s disease in patients with periodontal disease (presumably due to swallowing of oral-resident *Haemophilus* into the gut)^74^. Lastly, *Christensenellaceae* has been associated with metabolic health and negatively correlated with visceral fat mass in humans^75^, therefore, the reductions in this genera in AC cohorts offers support towards previously described associations between antibiotic use and obesity risk. Notably, mAbs did not alter the prevalence of any of these genera. Nevertheless, despite valuable insights obtained from rodent microbiome models and similarities in bacterial taxa observed between rodent and human systems, it is worth noting that human and rodent microbiomes also exhibit important differences due to diet, anatomy, physiology, and genetics^76^. Therefore, these findings will need further verification in humans in the future.

We were also able to glean valuable insights into effects of TT delivery of OM-relevant antibiotics on the nasopharyngeal microbiome, a topic that remains relatively unexplored. Oral antibiotics have previously been shown to alter nasopharyngeal microbiome diversity, but studies generally included combined effects of antibiotics with intranasal steroids or rinses^77,78^. Here, we have demonstrated that oral AC alone is a sufficient driver of nasopharyngeal microbiome disruption, and as in feces, oral and TT-delivered AC were capable of altering NPL diversity. Moreover, while it is known that middle ear fluids and debris are swallowed by way of the Eustachian tube^79^ and eventually reach the gut, we appear to be among the first to provide evidence that TT delivery of antibiotics into the middle ear can significantly impact both fecal and nasopharyngeal microbiome composition. Additionally, 7 days of oral AC resulted blooms in *Bacteroides*, and *Methanobrevibacter* at 2DPT. *Methanobrevibacter*, particularly, is an archaea genus considered challenging to treat due to β-lactam and macrolide resistance and is implicated in refractory sinusitis and proinflammatory responses in human intestinal dendritic cells^80,81^. Although not significant at the genus level due to the required small sample sizes imposed on USDA-protected species and conservative nature of ANCOM, blooms in NPL *Escherichia–Shigella* in oral AC-treated chinchillas occurred at 2DPT ( + 30.42% relative to baseline) and even more so at 5DPT (+ 53.63% relative to baseline; Supplementary File 5). In addition to concerns with AMR and opportunistic intestinal infection, elevated abundance of nasopharyngeal *Escherichia–Shigella* has been associated with multiple allergic diseases^82^ and chronic rhinosinusitis (CRS)^83^. Despite restrictions on sample sizes, multiple paired samples and longitudinal analyses have generated strong evidence to support claims that upon delivery to humans, it is unlikely that our mAbs will evoke collateral damage in either gastrointestinal or nasopharyngeal microbiomes and further, imply that neither of our mAbs would contribute to the plausible public and personal health concerns surrounding widespread broad-spectrum antibiotic use.

While our observed findings hold great promise for future clinical applications of our mAb technologies, several limitations of our studies must be acknowledged. First, small hindgut fermenting rodents, including chinchillas, are prone to antimicrobial toxicity from oral antibiotics such as beta-lactams, macrolides, and lincosamides^84^. Therefore, despite prolific and often unproblematic use in human patients, the use of oral AC in this study could have put chinchillas at risk for undesirable health outcomes. However, given the clinical relevance which necessitated inclusion of this treatment cohort, the dose and timeline of AC administration used in this study was carefully optimized to minimize animal distress and morbidity based on previous work with this antibiotic in the chinchilla host^4^. An additional limitation is that the composition and subsequent changes observed in the chinchilla fecal and nasopharyngeal microbiomes may not be completely equivocal to those to be expected in humans. For example, the nasal microbiome of humans is predominated by the phyla Firmicutes, Actinobacteriota, Proteobacteria, and Bacteroidota^85,86^, and while our relative abundance data showed that these phyla were among the top most abundant, Campylobacterota was also amongst the most prevalent and ranked as the second most abundant phylum in the nasopharyngeal microbiota of chinchillas at baseline. Moreover, the fecal microbiota of chinchillas at baseline was predominated by Firmicutes and Bacteroidota which is the same profile observed in humans where these two phyla make up approximately 90% of the gut microbiota^87^. At deeper levels of taxonomic classification, however, up to 85% of bacterial genera have been observed to differ between humans and rodents^76^. Although some extent of dissimilarity between models and the system modeled is to be expected, the primary aim of this work was to test whether our mAb technologies would alter an intact microbiome; our analyses do not support this possibility.

In children, perturbations in the microbiome can pose significant consequences due to the implications of the microbiome on organ development, immune maturation, and neurocognitive development^4,88^. Given a predominant prevalence in pediatric populations, OM acts as a major determinant of antibiotic exposure in children which could predispose for health ramifications later in life, especially in children with chronic OM infections. Here, we have generated promising evidence that our two distinctly targeted anti-biofilm mAbs can be administered directly into the middle ear without collateral damage to either nasopharyngeal or gut microbiomes and have brought these alternative therapeutic technologies one step closer to broadly reaching clinical application. Disease induction was strategically omitted from the present study to limit confounding variables, but future in vivo investigations are outlined to test the reproducibility of these results in the context of OM infectious challenge given these highly encouraging results.

## Methods

### Animal housing and handling

Twenty-seven healthy male and female adult chinchillas (*Chinchilla lanigera*; mean body weight 690 g) were purchased from Rauscher’s Chinchilla Ranch, LLC (LaRue, OH) and were undisturbed in the vivarium for 3 days to permit acclimation. All chinchillas were housed on autoclaved corn cob bedding and provided ad libitum access to fresh timothy hay, Mazuri chinchilla diet (catalog number 0001471; PMI Nutrition International LLC), and autoclaved water. Water bottles were changed every 2 days, and bedding was changed weekly. After acclimation, chinchillas were weighed, then divided into nine cohorts (n = 3/cohort) so that mean body weights were approximately equal. One day after baseline collections (described below), chinchillas were anesthetized by intramuscular injection of 10 mg/kg ketamine and 2 mg/kg xylazine, dual myringotomies were performed, and ventilation tube grommets (C-flex I.D. 1.27 mm, Medtronic XOmed®, Inc., Jacksonville, FL) were inserted into the tympanic membrane (TM) of both ears to mimic TT placement in children with OM. Prior to myringotomy and subsequent treatments, a video otoscope was used to confirm TM were healthy and free of inflammation. Upon conclusion of the experiment, chinchillas were humanely euthanized by intracardial injection of 1 ml Euthasol. This study was performed in accordance with federal, state, and institutional guidelines, under protocol #01304AR, approved by the Nationwide Children’s Hospital Institutional Animal Care and Use Committee.

### Fecal and nasopharyngeal lavage sample collections

Fecal and NPL samples were collected from chinchillas prior to treatment as a baseline and 2-, 5-, 7-, and 9 days post treatment (DPT) relative to the final dose administered. Briefly, alert animals were individually placed into clean, empty cages without bedding for fecal collections. Fecal pellets were collected into sterile microcentrifuge tubes with sterile forceps, weighed, and snap-frozen over the vapor phase of liquid nitrogen. Animals were then anesthetized, and NPL was performed by passive inhalation of 500 µl of sterile pyrogen-free saline delivered as 5–10 single microliter droplets at a time into one naris. With a sterile 1 mL syringe, fluid was collected from the contralateral naris as it was exhaled, transferred into sterile microcentrifuge tubes, volume recorded, and immediately snap-frozen. All samples were stored at −80 °C until further analysis. We were unable to collect samples from all chinchillas at all designated time points for the following reasons: 1 chinchilla in the positive-control cohort (AC-PO7d) did not produce a fecal sample on 1 occasion (2DPT) and was lost prior to completion of the study due to an adverse reaction to anesthesia (8DPT). Three additional chinchillas were lost due to either an unrelated bacterial infection (1 in saline cohort at 7DPT and 1 in AC-10 mg/kg cohort at 5DPT) or due to labyrinthitis (1 in anti-rsPilA cohort at 5DPT).

### Antibiotic and antibody preparation

Amoxicillin and clavulanate potassium (NDC 60432-065-75; Morton Grove Pharmaceuticals, Inc., Morton Grove, IL) and trimethoprim (Sigma-Aldrich, St. Louis, MO) in combination with sulfamethoxazole (MP Biomedicals, Irvine, CA) were suspended in sterile, pyrogen-free water and stored at 4 °C. Ofloxacin (Akorn, Inc., Forest, IL) was available as a ready-made 0.3% solution and was stored at room temperature according to manufacturer’s instructions. Sterile, pyrogen-free saline served as the negative control for antibiotic delivery. A murine monoclonal antibody directed against the immunoprotective α- & β-tip domains of the DNABII protein, IHF, as expressed by NTHi^30^ and anti-rsPilA murine monoclonal antibody directed against a recombinant soluble form of PilA, the majority subunit of the type IV pilus (T4P) expressed by NTHi^53^ were used (custom preparations by Rockland Immunochemicals Inc, Philadelphia, PA). Antibody treatments were diluted in sterile, pyrogen-free saline to a final concentration of 20 µg/mL.

### Treatment delivery

To represent the standard first-line treatment of children with acute OM^13^ and positive control for antibiotic-mediated gut microbiome disruption^4^, 10 mg/kg AC was delivered orally (PO) to alert animals in a twice daily divided dose (e.g., two PO doses of 5 mg/kg AC) for 7 days and a total of 14 administrations. Whereas children are commonly prescribed 10 days of oral AC, for this study the oral dosing timeline was selected to minimize incidence and duration of animal distress previously noted with prolonged oral AC treatment (significantly decreased gastrointestinal motility, altered character of fecal pellets, and huddling) while still inducing observable perturbations in the intestinal microbiome as we had reported earlier^4^. All TT treatments were administered in 125 µL volumes into each ear to equate to five drops of 0.3% ofloxacin, the standard-of-care treatment for CSOM and PTTO^7^. To promote treatment delivery to the middle ear, alert chinchillas were held so that heads could be tilted 45°. Treatments were delivered by a P200 micropipettor and held in position for 15 s to allow for entry of treatments into the middle ear space. After delivery, heads were returned to a neutral position for 2 min to promote fluid absorption by the middle ear mucosa and also limit potential passage into the nasopharynx via the Eustachian tube. Delivery of treatments through the TT and into the middle ear space was confirmed by video otoscopy after each administration as indicated by a lack of fluid pooled within the auditory canal and/or up against the tympanic membrane. Treatments were administered twice daily in divided doses and split between ears, for 2 consecutive days. For example, chinchillas in the AC-10 mg/kg cohort were administered a 125 µL volume of treatment into each ear for a final concentration of 5 mg/kg (2.5 mg/kg/ear) in the morning and repeated that same evening for a total daily dose of 10 mg/kg. The nine cohorts are summarized as follows: (1) AC-PO7d (positive control) (5 mg/kg BID orally—7 days), (2) AC-10 mg/kg (5 mg/kg BID via TT—2 days), (3) AC-5 mg/kg (2.5 mg/kg BID via TT—2 days), (4) anti-rsPilA (10 µg/day; 5 µg BID via TT—2 days), (5) anti-tip-chimer (10 µg/day; 5 µg BID via TT—2 days), (6) Ofloxacin (125 µL 0.3% solution BID via TT—2 days), (7) TS-30 mg/kg (15 mg/kg BID via TT—2 days), (8) TS-15 mg/kg (7.5 mg/kg BID via TT—2 days), and (9) Saline (125 µL BID via TT—2 days).

### DNA extraction and 16S rRNA gene sequencing

Approximately 100–300 µL NPL fluid or 1–3 fecal pellets, dependent on available or successfully collected sample, were used for DNA extraction with the QIAamp DNA Mini Kit (Cat. No. 51306; Qiagen, Hilden, Germany) per the manufacturer’s instructions with slight modifications to improve bacterial cell lysis. Samples were incubated for 45 min at 37 °C in lysozyme-mutanolysin buffer (pH 8.0) containing 22 mg/mL lysozyme, 0.1 U/mL mutanolysin, 20 mM TrisHCL, 1.2% Triton-x (Sigma-Aldrich, St. Louis, MO), and 2 mM EDTA (Thermo Fisher Scientific, Waltham, MA), followed by homogenization for 150 s with 0.1 mm zirconia beads on the Mini-Beadbeater-16 (BioSpec Products Inc., Bartlesville, OK). After bead beating, samples were incubated at 95 °C for 5 min with InhibitEx Buffer (Qiagen, Hilden, Germany), then incubated at 70 °C for 10 min with Proteinase K and Buffer AL. The QIAamp DNA Mini Kit isolation protocol was then followed, beginning with the ethanol step. DNA was quantified with the Qubit 2.0 Fluorometer (Life Technologies, Carlsbad, CA) using the dsDNA Broad Range Assay Kit for fecal samples or dsDNA High Sensitivity Assay Kit for NPL samples based on expected DNA yields per sample type. All DNA extracts were stored at −20 °C prior to submission for 16S rRNA gene sequencing. DNA was submitted to the Genomic Services Core at the Institute for Genomic Medicine at Nationwide Children’s Hospital (Columbus, OH) for library preparation and high-throughput sequencing. Paired-end (300 bp forward and reverse) sequences of the V4 hypervariable region of the 16S rRNA gene with primers 515F-806R were generated by Illumina MiSeq. Fecal and NPL samples from AC-PO7d were submitted as a single batch prior to other cohorts, which were submitted later in a split batch of fecal or NPL samples. In addition, upon initial sequencing, 8 of 13 fecal samples from AC-PO7d had a read count of zero (likely a consequence of prolonged oral AC) and were therefore re-sequenced using a greater amount of template DNA.

### 16S sequencing data analysis

Fecal and NPL sequences were analyzed independently with Quantitative Insights Into Microbial Ecology (QIIME) 2.0^89^, and DADA2^90^ was used for downstream amplicon processing, denoising, and quality control. After reads were imported, identical DADA2 trimming parameters were applied for batched data respective to sample type to control for batch effects and facilitate merging of like-sample datasets. To achieve an average quality score of at least 20, sequences were truncated from the 3’ end to 290 nt (NPL forward reads) and 240 nt (NPL reverse reads) or 250 nt (fecal forward reads) and 220nt (fecal reverse reads), and the first 20nt were trimmed from the 5’ end of both forward and reverse reads. Sequences that did not meet quality control criteria were discarded. Taxonomy was assigned using a trained classifier constructed from the SILVAv138.99 ribosomal RNA database^91,92^, and features (DADA2-classified ASVs) denoted as “Eukaryota”, “Unassigned”, “Chloroplast”, “Mitochondria”, not annotated beyond the phylum level, and not present in at least 5 samples were filtered from the dataset. Compositional diversity metrics were assessed at a sequencing depth of 9200 (fecal) or 4170 (NPL) sequences per sample following the core-metrics-phylogenetic QIIME 2.0 pipeline, and sequences with fewer reads were omitted from diversity analyses. Evenness, richness (number of observed species), Faith PD^93^, and Shannon diversity^94^ were calculated to measure changes in alpha diversity. To measure beta diversity changes, weighted and unweighted UniFrac^95,96^, Jaccard^67^, and Bray–Curtis^68^ distance matrices were constructed and visualized as three-dimensional principal coordinate analysis plots using the EMPeror software package^97^. The QIIME 2.0 longitudinal plugin^38^ was used to investigate temporal changes in the aforementioned diversity measures while controlling for intraindividual variation across time points. Longitudinal pairwise-distance and pairwise-difference comparisons were made between baseline and subsequent time points to assess within and between-treatment changes over respective intervals. Chinchillas without values for time points in the interval were dropped from the analysis. Longitudinal changes in the microbiome at the genus level were assessed by NMIT^98^. Pairwise correlations of features within an individual were used to compute between-subject distances across time points to compare between-treatment versus within-treatment differences. Differences were summarized into a single data point per individual and visualized with EMPeror. For differential abundance testing, no rarefaction was performed, unlike in diversity analyses. However, fecal and NPL datasets were further filtered to retain only features with a minimum abundance of 0.10% in at least 10% of samples and drop samples with fewer than 1000 total reads to minimize noise, sequencing artifacts, and computation time. For visualization, genus-level taxonomy was collapsed across individuals within a treatment and used to create heatmaps denoting observed abundance (averaged across treatment replicates) for the five time points.

### Statistical analysis

All diversity statistics were computed in QIIME 2.0 through respective plugins. Sequence read counts are reported as mean ± standard error. Data are represented as results from Omnibus tests (comparison of all cohorts to test whether differences existed between one or more cohorts) or pairwise comparisons (comparison of individual cohorts against specified time points or cohorts). Omnibus tests and pairwise comparisons of alpha diversity metrics were analyzed with Kruskal–Wallis tests. Differences in beta diversity distance and NMIT matrices were analyzed by permutational multivariate analysis of variance (PERMANOVA) with 999 randomizations of the data, and *P* values from Omnibus PERMANOVA tests were reported on respective PCoA plots. Beta diversity was further analyzed by permutational multivariate analysis of dispersion (PERMDISP) in order to assure dispersion differences were not driving significance (Supplementary File 1). Longitudinal pairwise-distances and pairwise-differences used Kruskal–Wallis for omnibus tests, and Mann–Whitney *U* tests for pairwise comparisons between treatments. A value of *P* < 0.05 was used to denote significance for all omnibus tests, while pairwise comparisons between cohorts or time points, as specified, were accepted as significant for Benjamini–Hochberg^99^ adjusted *P* values (*q*) of *q* < 0.20 to account for multiple testing false discovery rates. Significant differences in differential abundance between treatments at each individual time point were assessed using analysis of the composition of microbiomes (ANCOM)^100^.

### Reporting summary

Further information on research design is available in the Nature Research Reporting Summary linked to this article.

### Supplementary information

Supplementary Information

Reporting Summary

## Data Availability

The datasets generated and analyzed during the current study are available in the SRA repository under BioProject accession number PRJNA989545.
