# Supplementary material for: Oral and middle ear delivery of otitis media standard of care antibiotics, but not biofilm-targeted antibodies, alter chinchilla nasopharyngeal and fecal microbiomes
Source: NPJ Biofilms Microbiomes. 2024 Feb 3;10:10. doi: 10.1038/s41522-024-00481-0 (PMC10838340; doi:10.1038/s41522-024-00481-0)
Supplement: Supplementary file 1 — Supplementary Information [file 41522_2024_481_MOESM1_ESM.pdf]

Supplementary Table 1. Fecal alpha diversity—Pairwise comparisons within treatment.

	Time point 1	Time point 2	AC-PO7d p-value	AC-PO7d q-value	AC-10mg/kg p-value	AC-10mg/kg q-value	AC-5mg/kg p-value	AC-5mg/kg q-value	anti-rsPilA p-value	anti-rsPilA q-value	anti-tip-chimer p-value	anti-tip-chimer q-value	Ofloxacin p-value	Ofloxacin q-value	TS-30mg/kg p-value	TS-30mg/kg q-value	TS-15mg/kg p-value	TS-15mg/kg q-value	Saline p-value	Saline q-value
Evenness	Baseline	2DPT	0.083	0.253	0.121	0.243	0.439	0.705	0.050	0.208	0.827	0.827	0.564	0.827	0.275	0.688	0.827	0.827	0.827	1.000
	Baseline	5DPT	0.513	0.564	0.121	0.243	0.564	0.705	0.083	0.208	0.827	0.827	0.827	0.827	0.275	0.688	0.275	0.459	0.827	1.000
	Baseline	7DPT	0.050	0.253	0.221	0.245	1.000	1.000	1.000	1.000	0.564	0.805	0.513	0.827	0.127	0.633	0.050	0.248	1.000	1.000
	Baseline	9DPT	0.564	0.564	0.121	0.243	0.564	0.705	0.083	0.208	0.127	0.317	0.827	0.827	0.127	0.633	0.827	0.827	0.655	1.000
	2DPT	5DPT	0.083	0.253	0.439	0.439	0.248	0.705	0.564	0.626	0.827	0.827	0.248	0.827	0.827	0.827	0.275	0.459	0.513	1.000
	2DPT	7DPT	0.564	0.564	0.221	0.245	1.000	1.000	0.083	0.208	0.564	0.805	0.564	0.827	0.827	0.827	0.050	0.248	1.000	1.000
	2DPT	9DPT	0.121	0.253	0.121	0.243	0.564	0.705	0.564	0.626	0.127	0.317	0.083	0.827	0.827	0.827	0.513	0.732	0.655	1.000
	5DPT	7DPT	0.127	0.253	0.221	0.245	0.513	0.705	0.439	0.626	0.564	0.805	0.275	0.827	0.827	0.827	0.127	0.317	0.248	1.000
	5DPT	9DPT	0.248	0.355	0.121	0.243	0.275	0.705	0.439	0.626	0.127	0.317	0.827	0.827	0.827	0.827	0.827	0.827	0.655	1.000
	7DPT	9DPT	0.248	0.355	0.221	0.245	0.513	0.705	0.121	0.243	0.083	0.317	0.827	0.827	0.513	0.827	0.127	0.317	0.221	1.000
Richness	Baseline	2DPT	0.083	0.139	0.121	0.243	0.121	0.152	0.127	0.564	0.275	0.805	0.564	0.919	0.275	0.641	0.050	0.165	0.127	0.359
	Baseline	5DPT	0.050	0.139	0.121	0.243	0.083	0.119	0.248	0.564	0.275	0.805	0.513	0.919	0.513	0.641	0.077	0.191	1.000	1.000
	Baseline	7DPT	0.050	0.139	0.221	0.276	0.083	0.119	0.564	0.564	0.564	0.805	0.827	0.919	0.827	0.827	0.050	0.165	0.083	0.359
	Baseline	9DPT	0.076	0.139	0.121	0.243	0.083	0.119	0.564	0.564	0.513	0.805	0.275	0.919	0.513	0.641	0.127	0.253	0.180	0.359
	2DPT	5DPT	0.083	0.139	0.439	0.487	0.083	0.119	0.564	0.564	0.827	1.000	0.564	0.919	0.275	0.641	0.827	0.827	0.275	0.459
	2DPT	7DPT	0.564	0.564	0.221	0.276	0.083	0.119	0.248	0.564	1.000	1.000	1.000	1.000	0.275	0.641	0.513	0.570	1.000	1.000
	2DPT	9DPT	0.102	0.146	0.121	0.243	0.564	0.626	0.248	0.564	0.513	0.805	0.564	0.919	0.127	0.641	0.275	0.459	0.655	0.935
	5DPT	7DPT	0.275	0.344	0.221	0.276	0.050	0.119	0.439	0.564	0.564	0.805	0.827	0.919	0.513	0.641	0.513	0.570	0.083	0.359
	5DPT	9DPT	0.554	0.564	0.121	0.243	0.050	0.119	0.439	0.564	0.513	0.805	0.827	0.919	0.827	0.827	0.513	0.570	0.180	0.359
	7DPT	9DPT	0.076	0.139	1.000	1.000	0.827	0.827	0.439	0.564	1.000	1.000	0.658	0.919	0.513	0.641	0.050	0.165	1.000	1.000
Faith PD	Baseline	2DPT	0.083	0.139	0.121	0.303	0.121	0.173	0.127	0.705	0.275	0.688	0.083	0.416	0.513	0.641	0.050	0.165	0.127	0.599
	Baseline	5DPT	0.050	0.139	0.121	0.303	0.083	0.139	0.564	0.705	0.275	0.688	0.513	0.940	0.513	0.641	0.127	0.253	0.513	0.818
	Baseline	7DPT	0.050	0.139	0.221	0.315	0.083	0.139	0.564	0.705	0.564	0.705	0.513	0.940	0.275	0.641	0.050	0.165	0.083	0.599
	Baseline	9DPT	0.083	0.139	0.121	0.303	0.083	0.139	0.564	0.705	0.827	0.919	0.050	0.416	0.513	0.641	0.127	0.253	0.180	0.599
	2DPT	5DPT	0.083	0.139	0.439	0.487	0.083	0.139	0.248	0.705	0.513	0.705	0.564	0.940	0.827	0.827	0.513	0.513	0.275	0.688
	2DPT	7DPT	0.564	0.564	0.221	0.315	1.000	1.000	0.564	0.705	0.564	0.705	1.000	1.000	0.513	0.641	0.513	0.513	1.000	1.000
	2DPT	9DPT	0.121	0.173	0.439	0.487	1.000	1.000	0.248	0.705	0.275	0.688	1.000	1.000	0.827	0.827	0.275	0.459	0.655	0.818
	5DPT	7DPT	0.275	0.344	0.221	0.315	0.050	0.139	1.000	1.000	0.564	0.705	0.827	1.000	0.513	0.641	0.513	0.513	0.564	0.818
	5DPT	9DPT	0.564	0.564	0.121	0.303	0.050	0.139	0.439	0.705	0.275	0.688	0.513	0.940	0.513	0.641	0.513	0.513	0.655	0.818
	7DPT	9DPT	0.083	0.139	1.000	1.000	0.827	1.000	1.000	1.000	1.000	1.000	0.827	1.000	0.513	0.641	0.050	0.165	1.000	1.000
Shannon	Baseline	2DPT	0.083	0.119	0.121	0.202	0.439	0.627	0.050	0.278	0.513	0.805	0.248	0.827	0.050	0.124	0.050	0.099	0.513	0.818
	Baseline	5DPT	0.127	0.141	0.121	0.202	0.083	0.208	0.248	0.496	0.513	0.805	0.827	0.827	0.050	0.124	0.050	0.099	0.513	0.818
	Baseline	7DPT	0.050	0.119	0.221	0.221	0.083	0.208	1.000	1.000	0.564	0.805	0.827	0.827	0.050	0.124	0.050	0.099	0.564	0.818
	Baseline	9DPT	0.083	0.119	0.121	0.202	0.248	0.414	0.083	0.278	0.513	0.805	0.513	0.827	0.050	0.124	0.127	0.181	0.655	0.818
	2DPT	5DPT	0.083	0.119	0.121	0.202	0.083	0.208	1.000	1.000	0.827	1.000	0.248	0.827	0.827	0.827	0.827	0.827	0.513	0.818
	2DPT	7DPT	0.083	0.119	0.221	0.221	0.564	0.705	0.083	0.278	1.000	1.000	0.564	0.827	0.827	0.827	0.127	0.181	1.000	1.000
	2DPT	9DPT	0.121	0.141	0.121	0.202	1.000	1.000	0.564	0.805	0.127	0.805	0.564	0.827	0.827	0.827	0.827	0.827	0.655	0.818
	5DPT	7DPT	0.050	0.119	0.221	0.221	0.127	0.253	0.439	0.731	1.000	1.000	0.827	0.827	0.513	0.827	0.050	0.099	0.564	0.818
	5DPT	9DPT	1.000	1.000	0.121	0.202	0.050	0.208	1.000	1.000	0.275	0.805	0.827	0.827	0.827	0.827	0.275	0.344	0.655	0.818
	7DPT	9DPT	0.083	0.119	0.221	0.221	0.827	0.919	0.121	0.303	0.248	0.805	0.827	0.827	0.513	0.827	0.050	0.099	1.000	1.000

Each cohort column contains *p*-values and *q*-values representative of pairwise comparisons performed exclusively within each of the nine cohorts over the specified intervals. Unadjusted *p*-value from pairwise Kruskal-Wallis pairwise tests; *q*-value: Benjamini-Hochberg adjusted *p*-value. Statistical analyses were performed in QIIME 2.0. Significant difference within treatment over specified intervals where $q < 0.20$.

Supplementary Table 2. Nasopharyngeal lavage alpha diversity—Pairwise comparisons within treatment.

	Time point 1	Time point 2	AC-PO7d p-value	AC-PO7d q-value	AC-10mg/kg p-value	AC-10mg/kg q-value	AC-5mg/kg p-value	AC-5mg/kg q-value	anti-rsPilA p-value	anti-rsPilA q-value	anti-tip-chimer p-value	anti-tip-chimer q-value	Ofloxacin p-value	Ofloxacin q-value	TS-30mg/kg p-value	TS-30mg/kg q-value	TS-15mg/kg p-value	TS-15mg/kg q-value	Saline p-value	Saline q-value
Evenness	Baseline	2DPT	0.275	0.393	0.439	0.487	0.275	0.641	0.513	1.000	0.827	0.827	0.127	0.459	0.513	0.805	0.513	0.827	0.827	0.919
	Baseline	5DPT	0.513	0.570	0.439	0.487	0.827	0.827	1.000	1.000	0.827	0.827	0.513	0.626	0.513	0.805	0.827	0.827	0.827	0.919
	Baseline	7DPT	0.083	0.208	0.221	0.441	0.275	0.641	1.000	1.000	0.083	0.805	0.127	0.459	0.248	0.805	0.827	0.827	0.827	0.919
	Baseline	9DPT	0.083	0.208	0.121	0.441	0.127	0.641	0.248	1.000	0.513	0.805	0.248	0.459	0.827	0.827	0.827	0.827	1.000	1.000
	2DPT	5DPT	0.513	0.570	0.439	0.487	0.513	0.641	0.564	1.000	0.827	0.827	0.275	0.459	0.513	0.805	0.275	0.827	0.513	0.919
	2DPT	7DPT	0.083	0.208	0.221	0.441	0.513	0.641	1.000	1.000	0.248	0.805	0.827	0.827	0.083	0.805	0.513	0.827	0.513	0.919
	2DPT	9DPT	0.083	0.208	0.121	0.441	0.827	0.827	0.564	1.000	0.513	0.805	0.564	0.626	0.827	0.827	0.275	0.827	0.564	0.919
	5DPT	7DPT	0.248	0.393	1.000	1.000	0.513	0.641	1.000	1.000	0.564	0.805	0.275	0.459	0.564	0.805	0.827	0.827	0.513	0.919
	5DPT	9DPT	0.248	0.393	0.439	0.487	0.275	0.641	0.439	1.000	0.513	0.805	0.248	0.459	0.827	0.827	0.827	0.827	0.564	0.919
	7DPT	9DPT	1.000	1.000	0.221	0.441	0.513	0.641	1.000	1.000	0.248	0.805	0.564	0.626	0.248	0.805	0.827	0.827	0.564	0.919
Richness	Baseline	2DPT	0.050	0.416	0.221	0.441	0.043	0.116	0.127	0.317	0.827	0.919	0.513	0.570	0.827	1.000	0.513	0.827	0.658	0.731
	Baseline	5DPT	0.275	0.459	0.439	0.487	0.653	0.653	0.767	1.000	0.513	0.854	0.500	0.570	0.050	0.495	0.376	0.827	0.825	0.825
	Baseline	7DPT	0.248	0.459	0.221	0.441	0.046	0.116	1.000	1.000	1.000	1.000	0.376	0.570	0.767	1.000	0.500	0.827	0.513	0.705
	Baseline	9DPT	0.564	0.626	0.439	0.487	0.507	0.570	0.083	0.317	0.275	0.688	0.083	0.278	0.513	1.000	0.513	0.827	0.083	0.416
	2DPT	5DPT	0.275	0.459	0.439	0.487	0.046	0.116	1.000	1.000	0.376	0.752	0.275	0.550	0.513	1.000	0.827	0.827	0.268	0.537
	2DPT	7DPT	0.564	0.626	0.221	0.441	0.046	0.116	0.564	0.940	0.767	0.919	0.513	0.570	1.000	1.000	0.513	0.827	0.513	0.705
	2DPT	9DPT	0.083	0.416	1.000	1.000	0.507	0.570	0.083	0.317	0.050	0.248	0.767	0.767	0.658	1.000	0.658	0.827	0.139	0.462
	5DPT	7DPT	0.564	0.626	0.221	0.441	0.513	0.570	0.439	0.877	0.767	0.919	0.275	0.550	0.767	1.000	0.658	0.827	0.268	0.537
	5DPT	9DPT	0.139	0.459	0.439	0.487	0.500	0.570	1.000	1.000	0.050	0.248	0.083	0.278	0.513	1.000	0.827	0.827	0.076	0.416
	7DPT	9DPT	0.683	0.683	0.221	0.441	0.513	0.570	0.121	0.317	0.083	0.278	0.083	0.278	1.000	1.000	0.827	0.827	0.564	0.705
Faith PD	Baseline	2DPT	0.050	0.278	1.000	1.000	0.275	0.306	0.513	0.626	0.827	1.000	0.275	0.550	0.827	1.000	0.275	0.827	0.513	0.705
	Baseline	5DPT	0.127	0.317	0.439	0.548	0.127	0.211	0.564	0.626	0.127	0.633	0.275	0.550	0.513	1.000	0.827	0.827	0.275	0.550
	Baseline	7DPT	0.083	0.278	0.221	0.441	0.050	0.124	1.000	1.000	1.000	1.000	0.827	0.827	1.000	1.000	0.827	0.827	0.827	0.919
	Baseline	9DPT	0.248	0.393	0.121	0.441	0.275	0.306	0.083	0.626	0.275	0.688	0.564	0.705	0.827	1.000	0.275	0.827	0.564	0.705
	2DPT	5DPT	0.275	0.393	1.000	1.000	0.050	0.124	0.564	0.626	0.827	1.000	0.275	0.550	0.827	1.000	0.827	0.827	0.275	0.550
	2DPT	7DPT	0.248	0.393	0.221	0.441	0.050	0.124	0.564	0.626	1.000	1.000	0.827	0.827	1.000	1.000	0.827	0.827	0.275	0.550
	2DPT	9DPT	0.083	0.278	0.439	0.548	0.050	0.124	0.248	0.626	0.275	0.688	0.564	0.705	0.827	1.000	0.513	0.827	0.564	0.705
	5DPT	7DPT	1.000	1.000	0.221	0.441	0.127	0.211	0.439	0.626	1.000	1.000	0.275	0.550	0.564	1.000	0.827	0.827	0.275	0.550
	5DPT	9DPT	0.564	0.626	0.439	0.548	0.827	0.827	0.439	0.626	0.127	0.633	0.248	0.550	0.827	1.000	0.827	0.827	0.248	0.550
	7DPT	9DPT	0.439	0.548	0.221	0.441	0.275	0.306	0.439	0.626	0.564	1.000	0.564	0.705	1.000	1.000	0.827	0.827	1.000	1.000
Shannon	Baseline	2DPT	0.127	0.550	0.439	0.548	0.513	0.827	0.050	0.243	0.827	0.827	0.127	0.211	0.513	0.940	0.513	0.827	0.275	0.805
	Baseline	5DPT	0.513	0.805	0.439	0.548	0.513	0.827	0.564	0.705	0.513	0.705	0.827	0.827	0.127	0.940	0.827	0.827	0.827	0.919
	Baseline	7DPT	0.248	0.550	0.221	0.368	0.275	0.827	0.564	0.705	0.248	0.414	0.127	0.211	1.000	1.000	0.513	0.827	0.513	0.805
	Baseline	9DPT	0.248	0.550	0.121	0.368	0.827	0.827	0.083	0.243	0.127	0.317	0.083	0.211	0.827	1.000	0.827	0.827	0.248	0.805
	2DPT	5DPT	0.275	0.550	1.000	1.000	0.827	0.827	1.000	1.000	0.827	0.827	0.127	0.211	0.513	0.940	0.827	0.827	0.513	0.805
	2DPT	7DPT	1.000	1.000	0.221	0.368	0.127	0.827	0.564	0.705	0.564	0.705	0.827	0.827	0.564	0.940	0.513	0.827	0.827	0.919
	2DPT	9DPT	1.000	1.000	0.121	0.368	0.513	0.827	0.083	0.243	0.127	0.317	0.564	0.705	0.827	1.000	0.513	0.827	0.564	0.805
	5DPT	7DPT	0.564	0.805	0.221	0.368	0.513	0.827	1.000	1.000	0.248	0.414	0.050	0.211	1.000	1.000	0.275	0.827	0.513	0.805
	5DPT	9DPT	0.248	0.550	0.121	0.368	0.827	0.827	0.121	0.243	0.050	0.317	0.083	0.211	0.513	0.940	0.827	0.827	0.248	0.805
	7DPT	9DPT	1.000	1.000	1.000	1.000	0.827	0.827	0.121	0.243	0.083	0.317	0.248	0.355	0.564	0.940	0.827	0.827	1.000	1.000

Each cohort column contains *p*-values and *q*-values representative of pairwise comparisons performed exclusively within each of the nine cohorts over the specified intervals. Unadjusted *p*-value from pairwise Kruskal-Wallis pairwise tests; *q*-value: Benjamini-Hochberg adjusted *p*-value. Statistical analyses were performed in QIIME 2.0. Significant difference within treatment over specified intervals where $q < 0.20$.

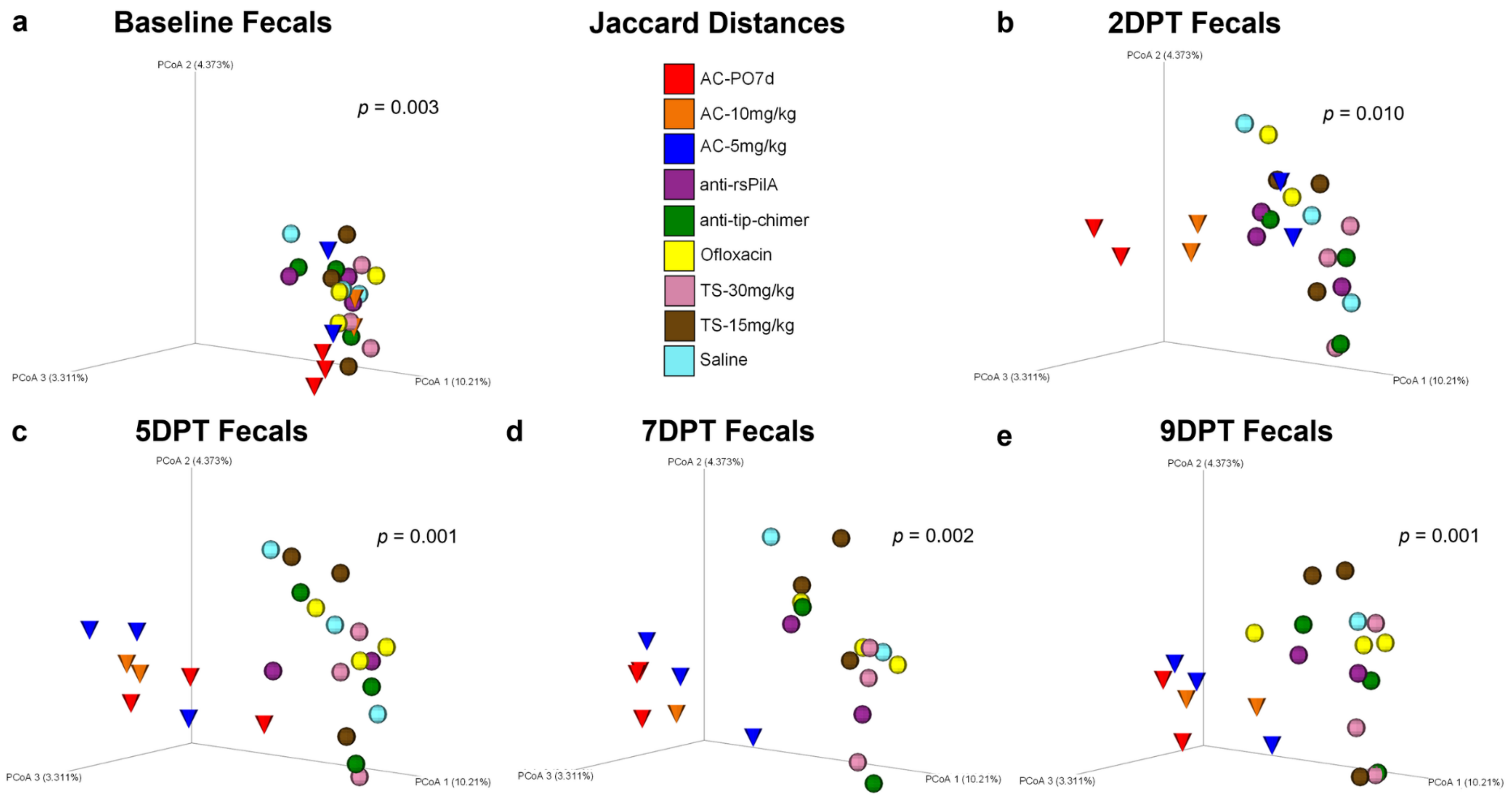

Supplementary Figure 1. Fecal Jaccard beta diversity. Principal coordinate analysis plots of Jaccard distance matrices for fecal samples from all treatments at (a) baseline, (b) 2DPT, (c) 5DPT, (d) 7DPT, and (e) 9DPT. Differences in distances between treatments were analyzed in QIIME 2.0 by PERMANOVA with 999 randomizations of the data. Omnibus test PERMANOVA *p*-values that tested for differences between the nine cohorts at each specified time point are displayed on respective graphs (significant where $p < 0.05$). Individual chinchillas are colored according to cohort treatment as depicted in the legend. Chinchillas in AC-treated cohorts are depicted as cones to emphasize separation from other cohorts which are depicted as spheres. Sample sizes are reported in Table 1.

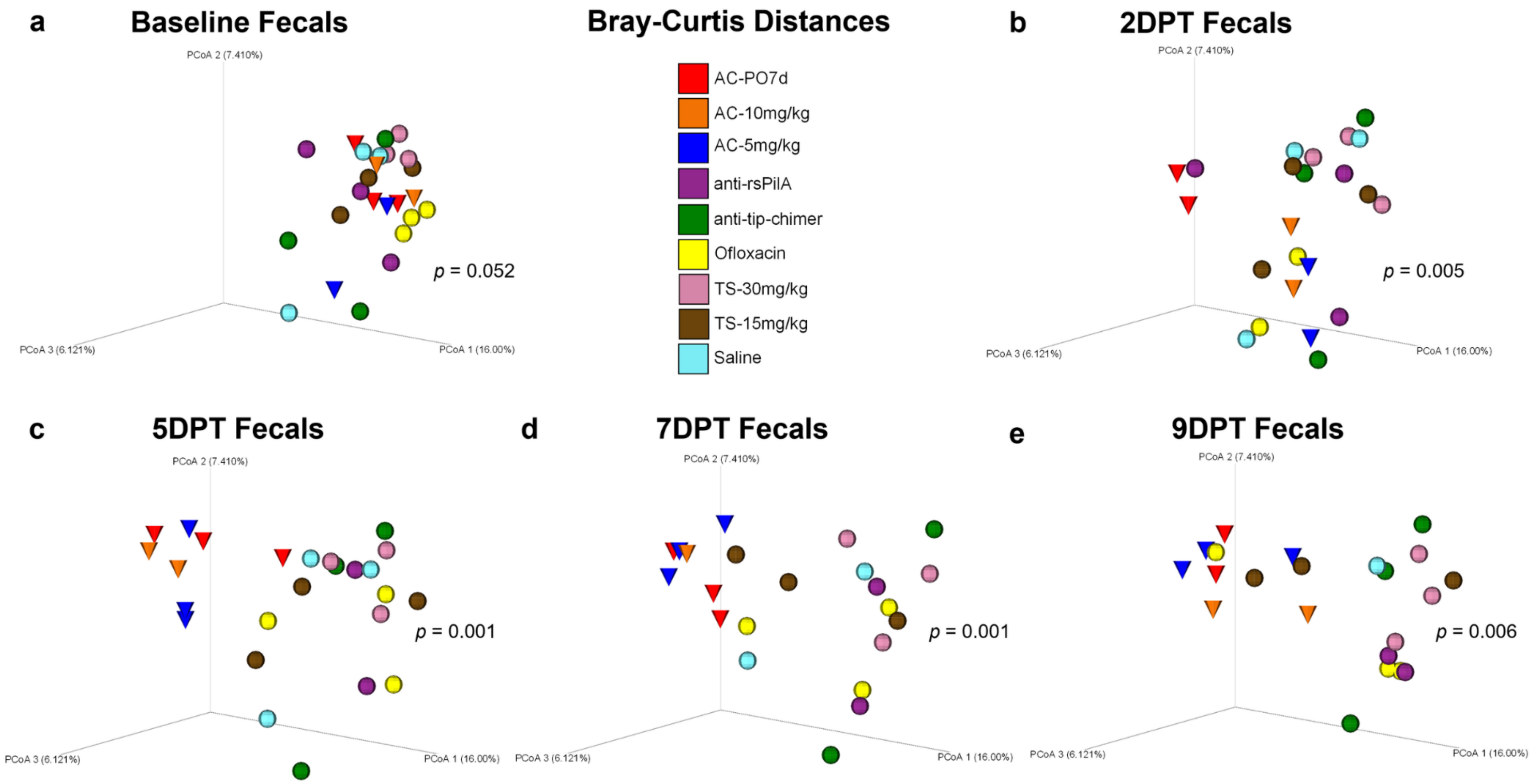

Supplementary Figure 2. Fecal Bray-Curtis beta diversity. Principal coordinate analysis plots of Bray-Curtis dissimilarity for fecal samples from all treatments at (a) baseline, (b) 2DPT, (c) 5DPT, (d) 7DPT, and (e) 9DPT. Differences in distances between treatments were analyzed in QIIME 2.0 by PERMANOVA with 999 randomizations of the data. Omnibus test PERMANOVA *p*-values that tested for differences between the nine cohorts at each specified time point are displayed on respective graphs (significant where $p < 0.05$). Individual chinchillas are colored according to cohort treatment as depicted in the legend. Chinchillas in AC-treated cohorts are depicted as cones to emphasize separation from other cohorts which are depicted as spheres. Sample sizes are reported in Table 1.

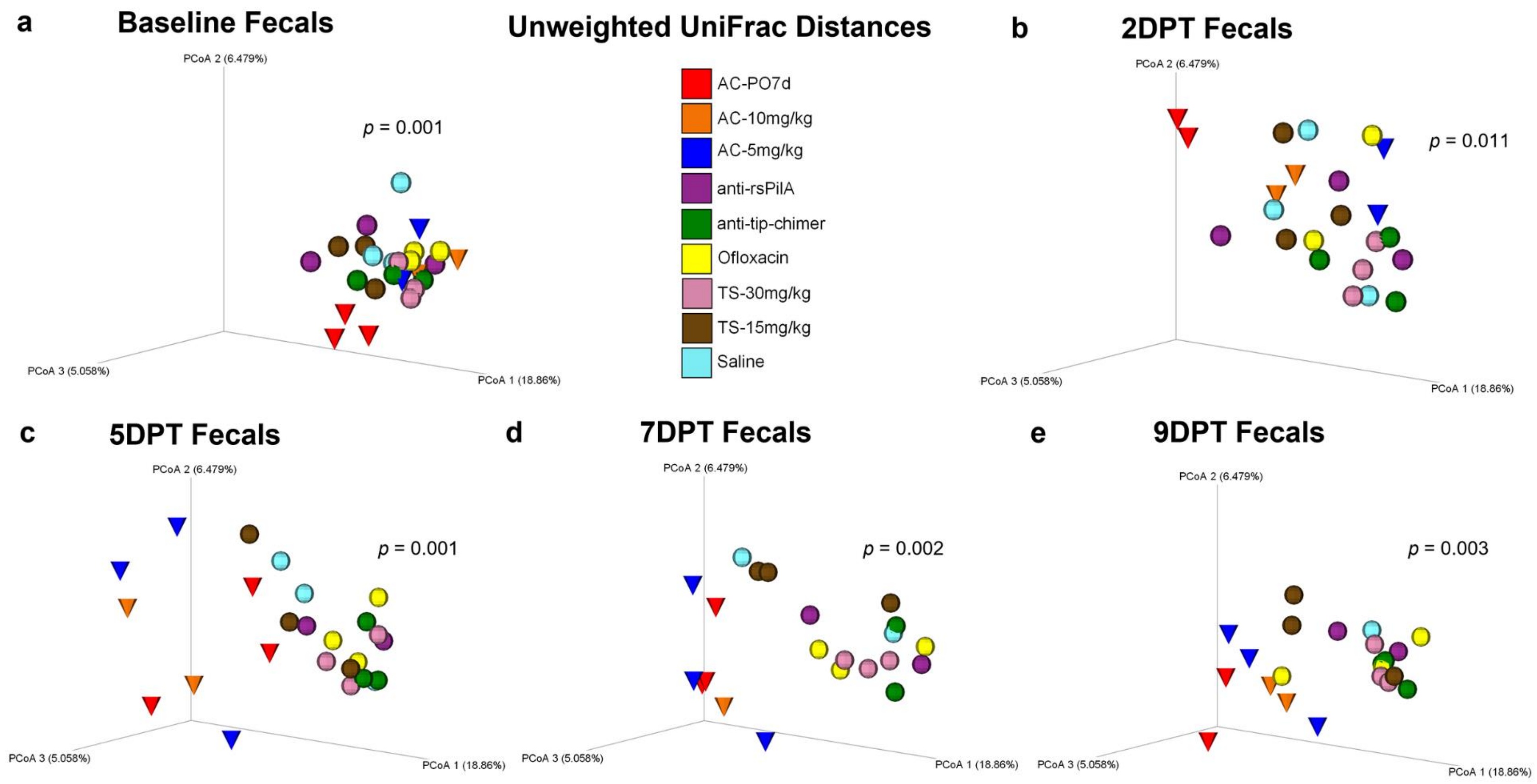

Supplementary Figure 3. Fecal unweighted UniFrac beta diversity. Principal coordinate analysis plots of unweighted UniFrac distance matrices for fecal samples from all treatments at (a) baseline, (b) 2DPT, (c) 5DPT, (d) 7DPT, and (e) 9DPT. Differences in distances between treatments were analyzed in QIIME 2.0 by PERMANOVA with 999 randomizations of the data. Omnibus test PERMANOVA *p*-values that tested for differences between the nine cohorts at each specified time point are displayed on respective graphs (significant where $p < 0.05$). Individual chinchillas are colored according to cohort treatment as depicted in the legend. Chinchillas in AC-treated cohorts are depicted as cones to emphasize separation from other cohorts which are depicted as spheres. Sample sizes are reported in Table 1.

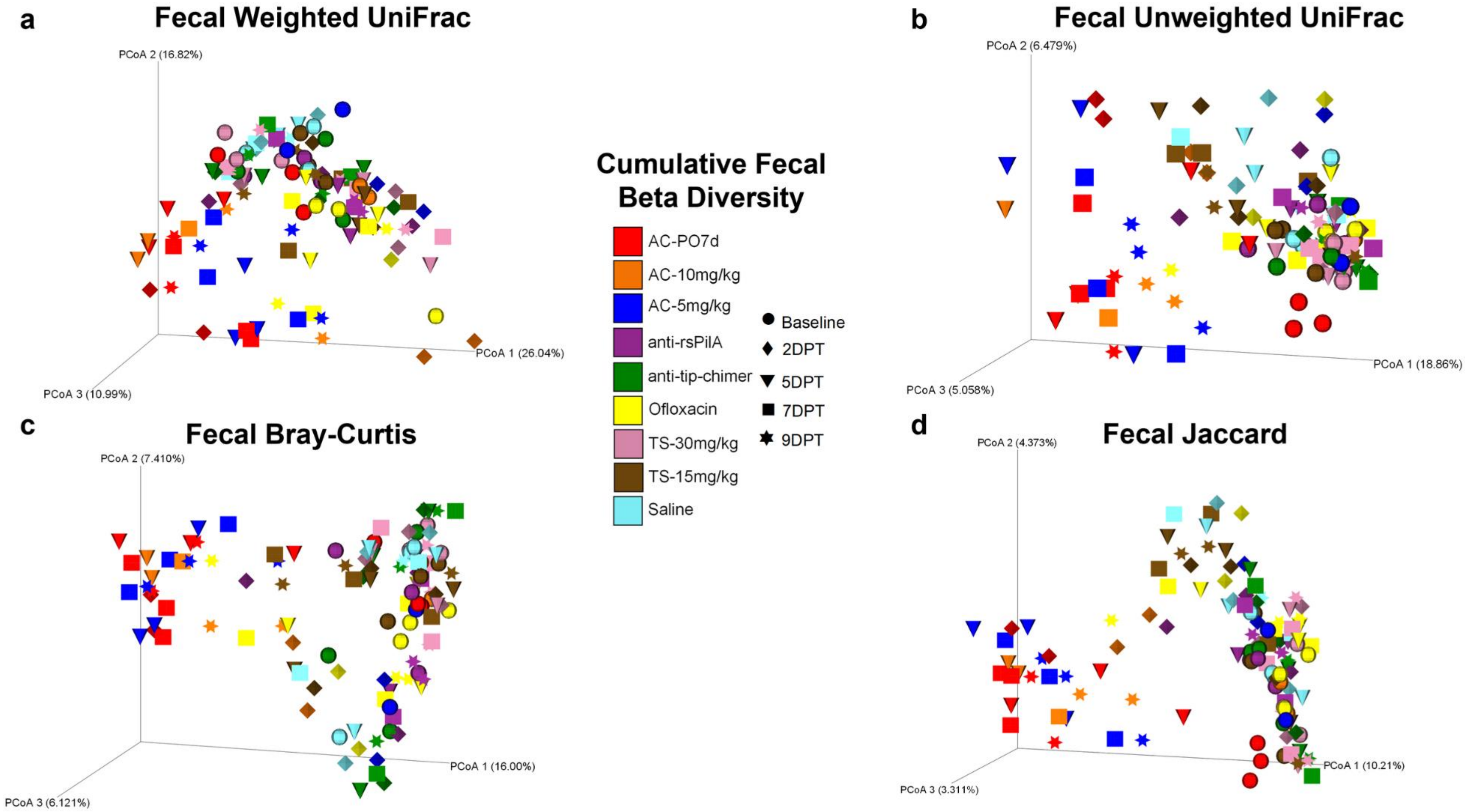

Supplementary Figure 4. Cumulative changes in fecal beta diversity. Principal coordinate analysis plots of fecal (a) weighted UniFrac, (b) unweighted UniFrac, (c) Bray-Curtis and (d) Jaccard distance matrices depicting all time points. No statistical analysis performed. Individual chinchillas are represented by shapes depicting baseline(●), 2DPT(♦), 5DPT(▼), 7DPT(■), 9DPT(✱) and colored according to cohort treatment as depicted in the legend. Sample sizes are reported in Table 1.

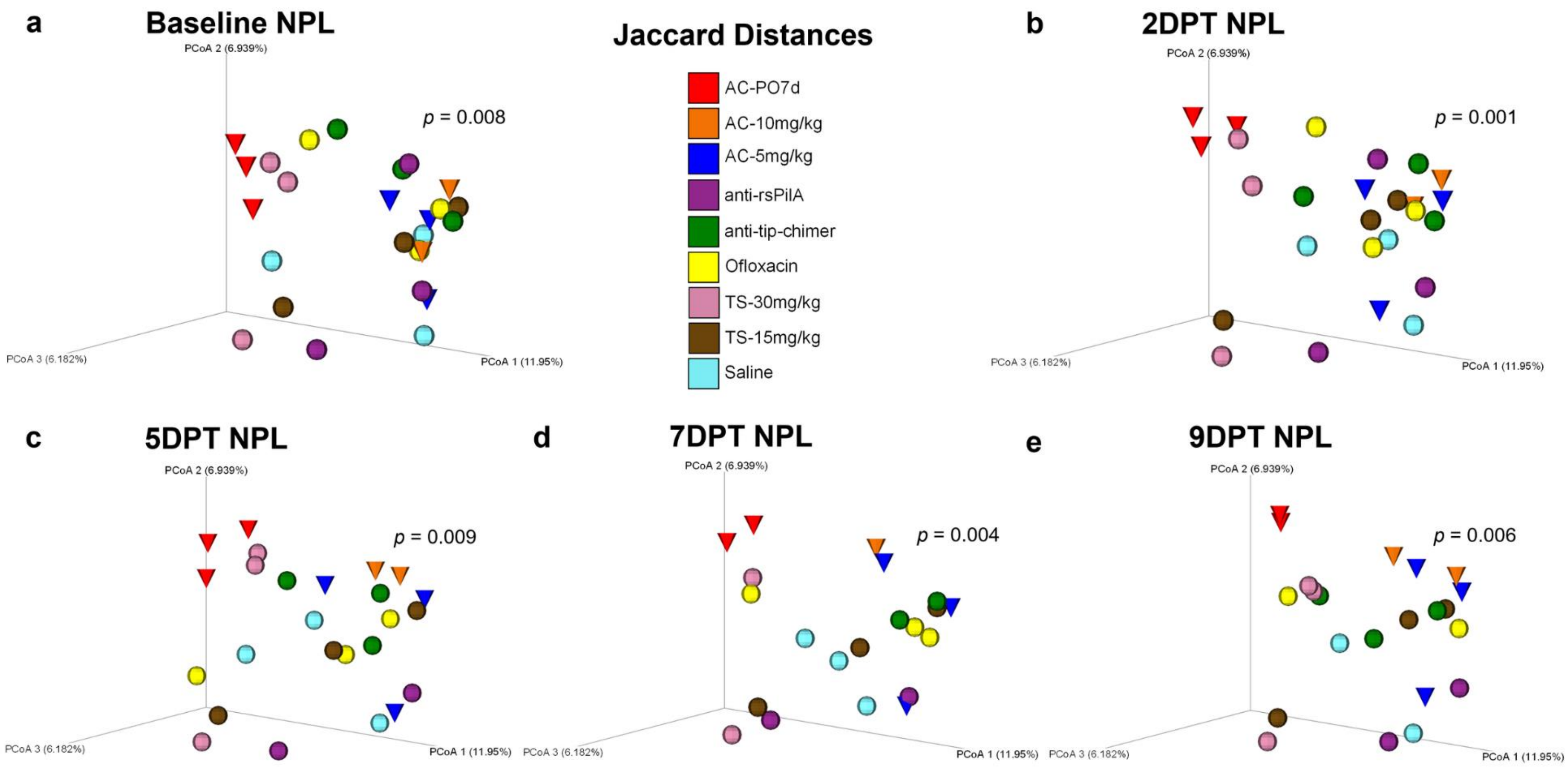

Supplementary Figure 5. Nasopharyngeal lavage Jaccard beta diversity. Principal coordinate analysis plots of Jaccard distance matrices for NPL samples from all treatments at (a) baseline, (b) 2DPT, (c) 5DPT, (d) 7DPT, and (e) 9DPT. Differences in distances between treatments were analyzed in QIIME 2.0 by PERMANOVA with 999 randomizations of the data. Omnibus test PERMANOVA *p*-values that tested for differences between the nine cohorts at each specified time point are displayed on respective graphs (significant where $p < 0.05$). Individual chinchillas are colored according to cohort treatment as depicted in the legend. Chinchillas in AC-treated cohorts are depicted as cones to emphasize separation from other cohorts which are depicted as spheres. Sample sizes are reported in Table 2.

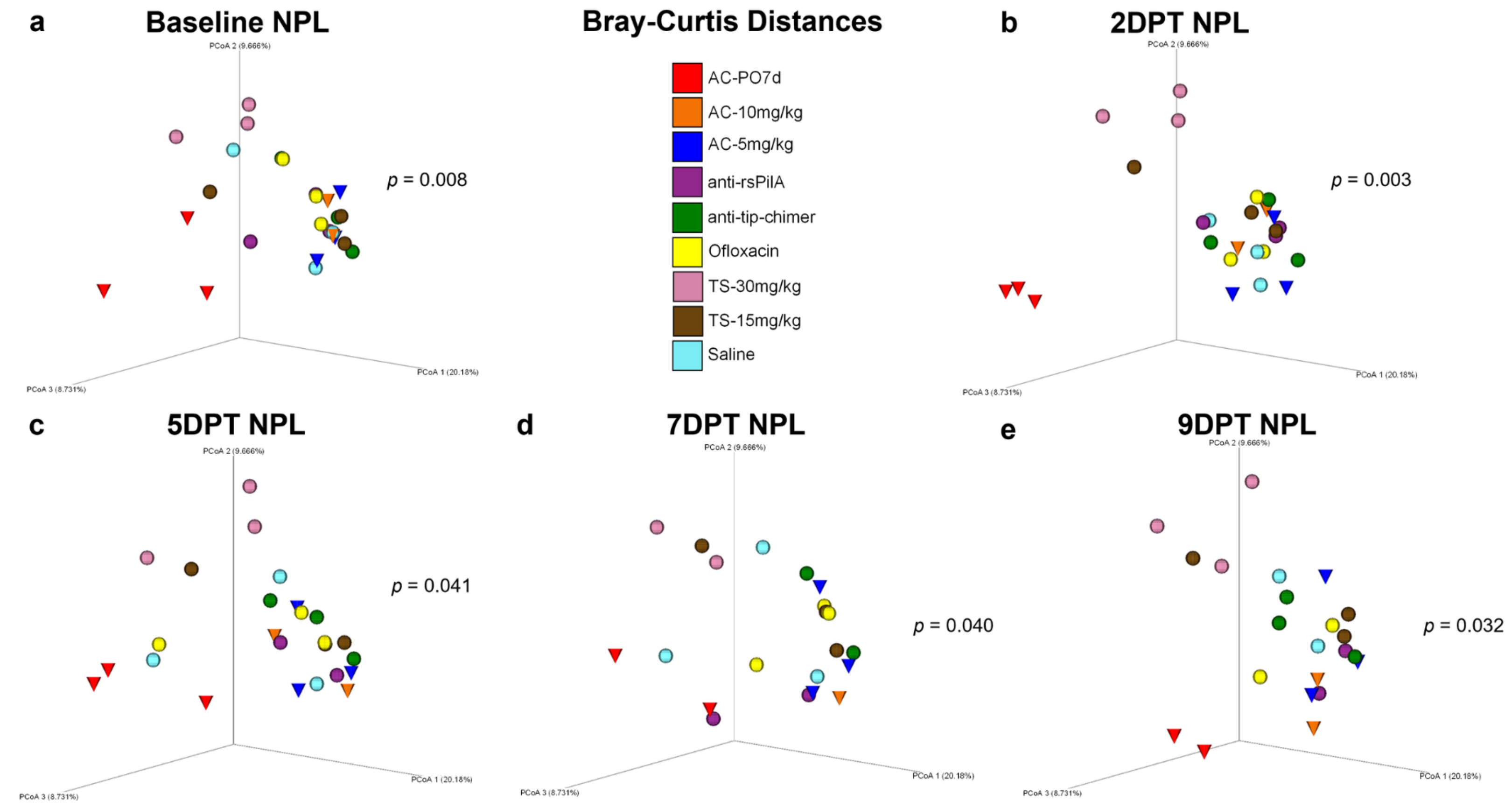

Supplementary Figure 6. Nasopharyngeal lavage Bray-Curtis beta diversity. Principal coordinate analysis plots of Bray-Curtis dissimilarity for NPL samples from all treatments at (a) baseline, (b) 2DPT, (c) 5DPT, (d) 7DPT, and (e) 9DPT. Differences in distances between treatments were analyzed in QIIME 2.0 by PERMANOVA with 999 randomizations of the data. Omnibus test PERMANOVA *p*-values that tested for differences between the nine cohorts at each specified time point are displayed on respective graphs (significant where $p < 0.05$). Individual chinchillas are colored according to cohort treatment as depicted in the legend. Chinchillas in AC-treated cohorts are depicted as cones to emphasize separation from other cohorts which are depicted as spheres. Sample sizes are reported in Table 2.

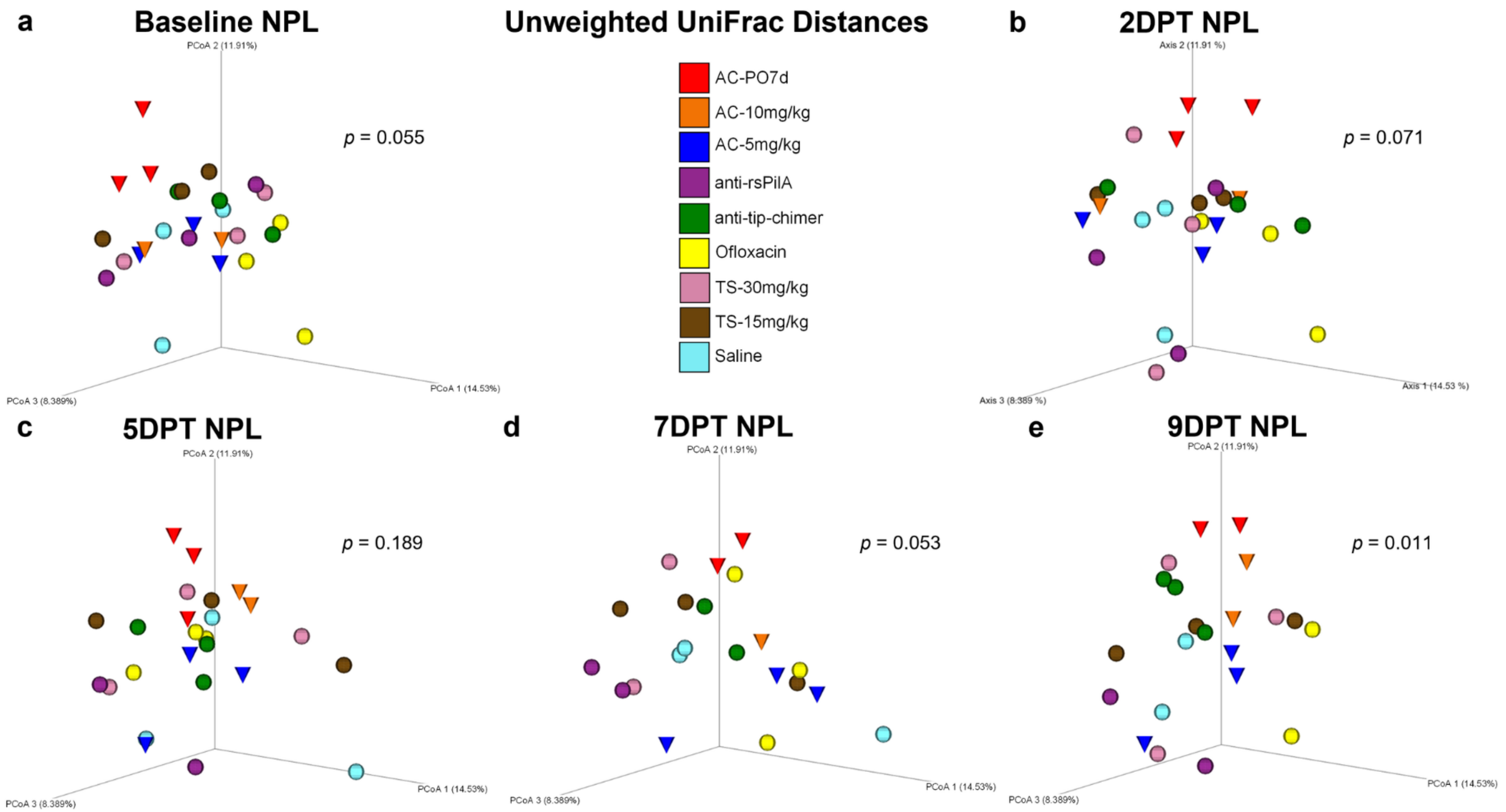

Supplementary Figure 7. Nasopharyngeal lavage unweighted UniFrac beta diversity. Principal coordinate analysis plots of unweighted UniFrac distance matrices for NPL samples from all treatments at (a) baseline, (b) 2DPT, (c) 5DPT, (d) 7DPT, and (e) 9DPT. Differences in distances between treatments were analyzed in QIIME 2.0 by PERMANOVA with 999 randomizations of the data. Omnibus test PERMANOVA *p*-values that tested for differences between the nine cohorts at each specified time point are displayed on respective graphs (significant where $p < 0.05$). Individual chinchillas are colored according to cohort treatment as depicted in the legend. Chinchillas in AC-treated cohorts are depicted as cones to emphasize separation from other cohorts which are depicted as spheres. Sample sizes are reported in Table 2.

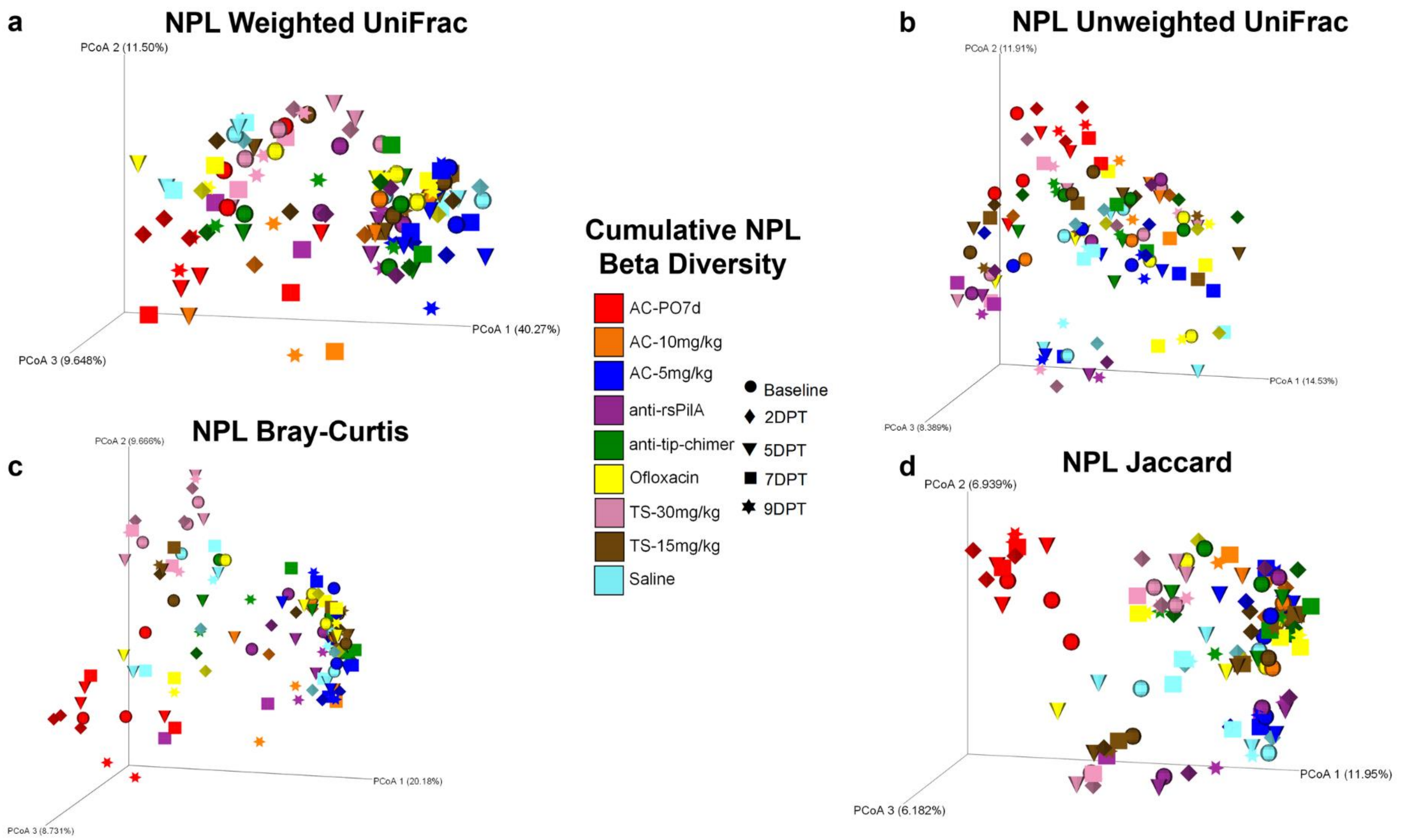

Supplementary Figure 8. Cumulative changes in nasopharyngeal lavage beta diversity. Principal coordinate analysis plots of NPL (a) weighted UniFrac, (b) unweighted UniFrac, (c) Bray-Curtis and (d) Jaccard distance matrices depicting all time points. No statistical analysis performed. Individual chinchillas are represented by shapes depicting baseline(●), 2DPT(♦), 5DPT(▼), 7DPT(■), 9DPT(✸) and colored according to cohort as depicted in the legend. Sample sizes are reported in Table 2.

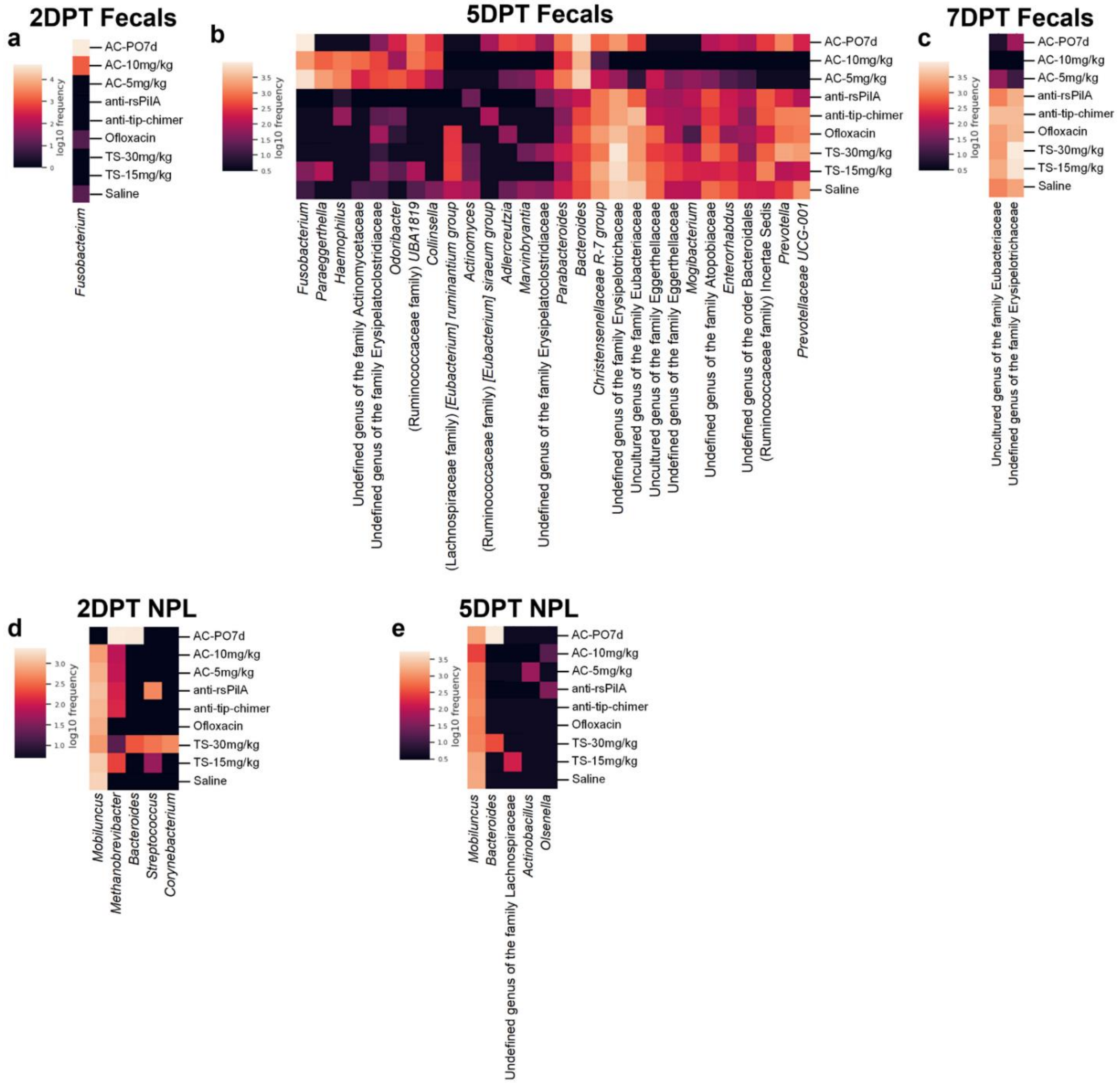

Supplementary Figure 9. Significant fecal and nasopharyngeal lavage taxa. Heatmaps denote the mean log10 frequency of significant fecal genus-level taxa at (a) 2DPT, (b) 5DPT, (c) 7DPT and significant NPL genus-level taxa at (d) 2DPT and (e) 5DPT per ANCOM analysis. Darker boxes denote lower prevalence, and lighter boxes denote greater prevalence.

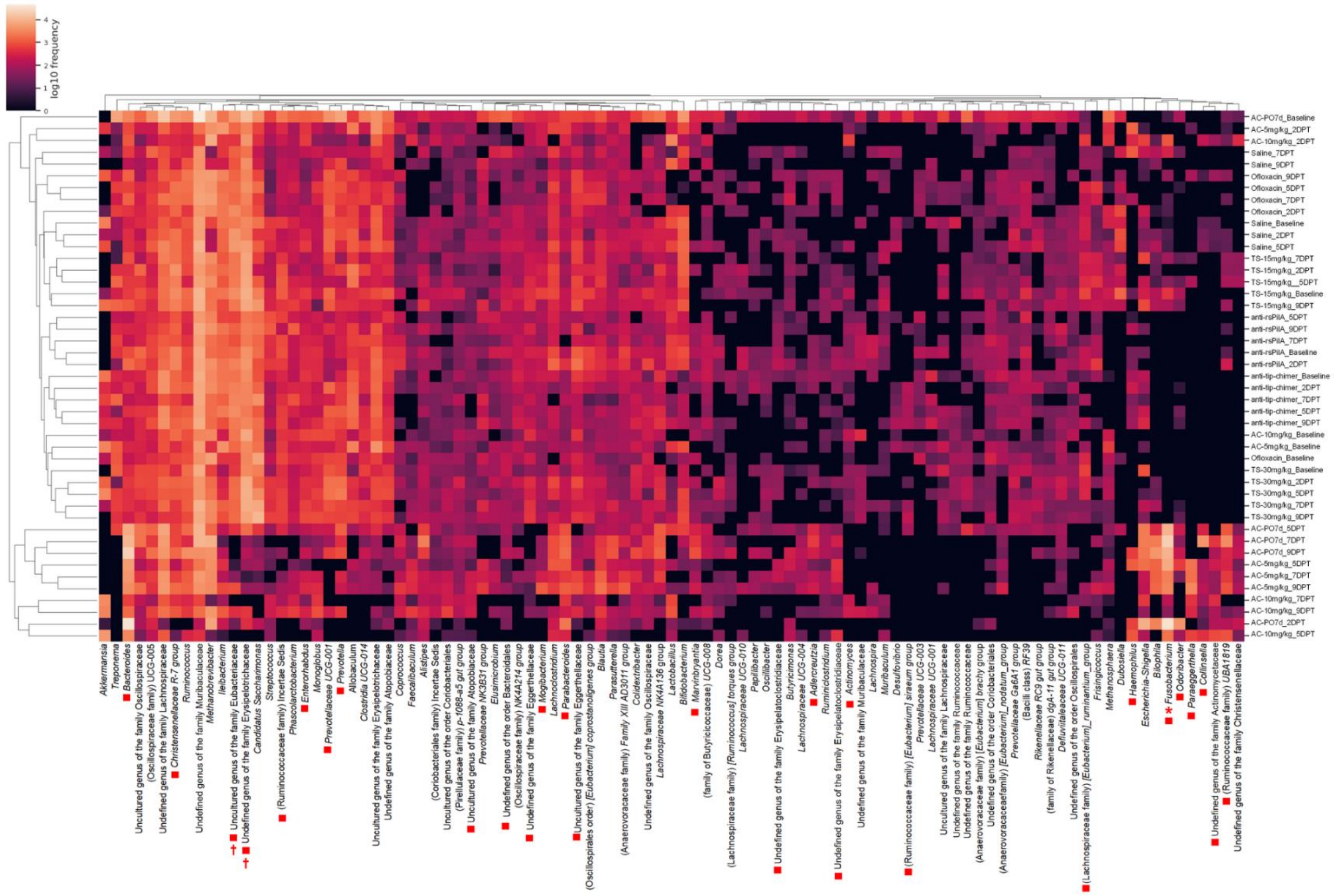

Supplementary Figure 10. Fecal taxa frequency at the genus level. Heatmap hierarchal clustering the mean log10 frequency of genus level taxa observed in fecal samples at each time point, within treatment. Treatments that are grouped by dendrogram branches and located closer together on the y-axis exhibited similar frequency of taxa. Darker boxes denote lower prevalence, and lighter boxes denote greater prevalence. Significant taxa per ANCOM at 2DPT (*), 5DPT (■), and 7DPT (†) are depicted by specified symbols. No significant differences were observed for 9DPT genera. Sample sizes are reported in Table 1.

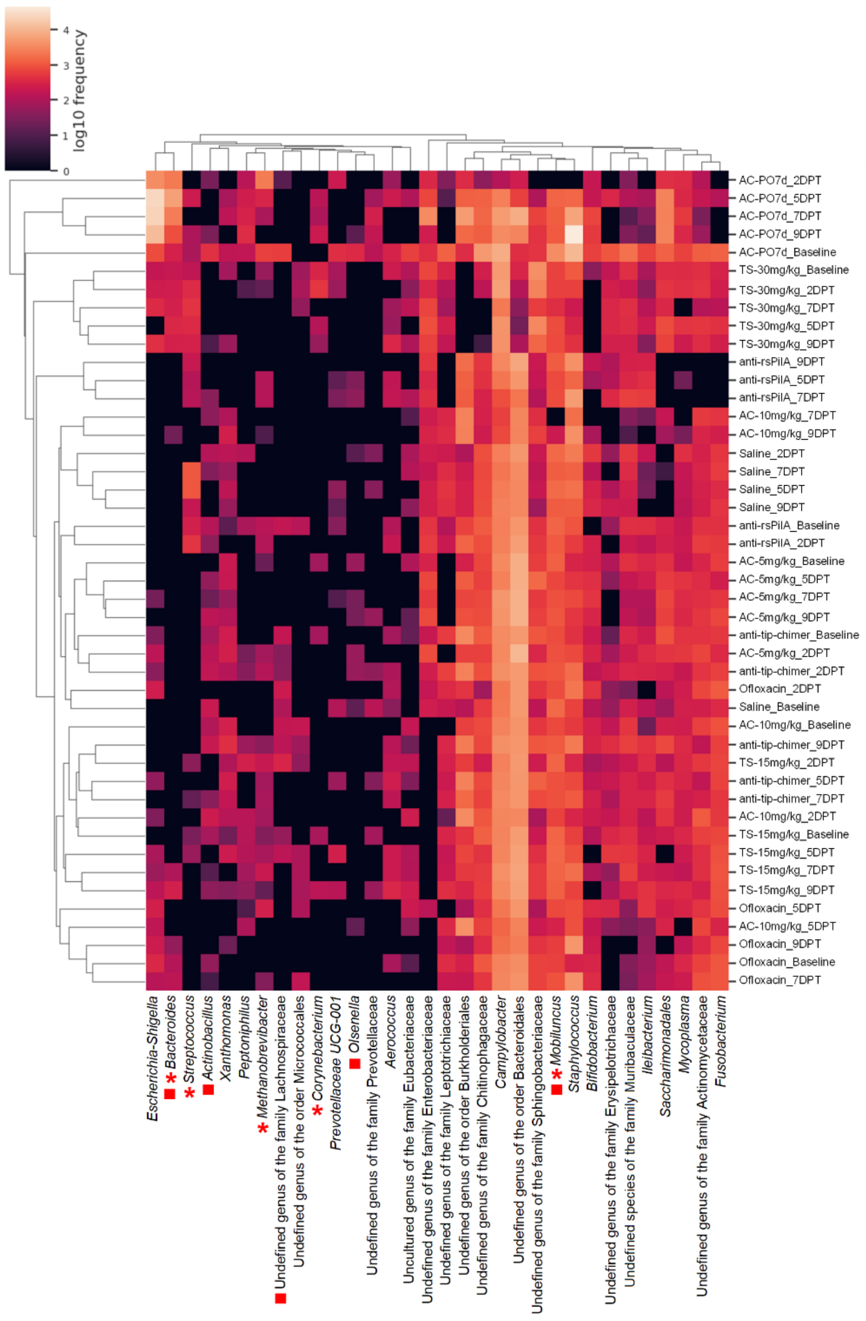

Supplementary Figure 11. Nasopharyngeal lavage taxa frequency at the genus level. Heatmap with hierarchical clustering denotes the mean log10 frequency of genus level taxa observed in NPL samples at each time point, within treatment. Treatments that are grouped by dendrogram branches and located closer together on the y-axis exhibited similar frequency of taxa. Darker boxes denote lower prevalence, and lighter boxes denote greater prevalence. Significant taxa per ANCOM at 2DPT (*), 5DPT (■), and 7DPT (†) are depicted by specified symbols. No significant differences were observed for 9DPT genera. Sample sizes are reported in Table 2.

Supplementary File 1
Beta Diversity Output

Fecal Weighted UniFrac-Baseline

PERMANOVA Pairwise Comparisons (999 permutations)

Group 1	Group 2	Sample size	Permutations	pseudo-F	p-value	q-value
AC-PO7d	Ofloxacin	6	999	2.354	0.079	0.339
AC-PO7d	Saline	6	999	1.250	0.314	0.595
AC-PO7d	TS-30mg/kg	6	999	1.112	0.418	0.602
AC-PO7d	TS-15mg/kg	6	999	2.214	0.099	0.339
AC-PO7d	anti-tip-chimer	6	999	1.552	0.206	0.428
AC-PO7d	anti-rsPilA	6	999	1.153	0.488	0.663
AC-5mg/kg	AC-PO7d	5	999	1.929	0.110	0.339
AC-5mg/kg	AC-10mg/kg	4	999	1.217	0.343	0.602
AC-5mg/kg	Ofloxacin	5	999	1.831	0.196	0.428
AC-5mg/kg	Saline	5	999	0.409	0.892	0.944
AC-5mg/kg	TS-30mg/kg	5	999	1.589	0.214	0.428
AC-5mg/kg	TS-15mg/kg	5	999	1.055	0.397	0.602
AC-5mg/kg	anti-tip-chimer	5	999	0.757	0.824	0.927
AC-5mg/kg	anti-rsPilA	5	999	0.850	0.516	0.663
AC-10mg/kg	AC-PO7d	5	999	3.173	0.091	0.339
AC-10mg/kg	Ofloxacin	5	999	1.063	0.416	0.602
AC-10mg/kg	Saline	5	999	1.224	0.364	0.602
AC-10mg/kg	TS-30mg/kg	5	999	4.233	0.100	0.339
AC-10mg/kg	TS-15mg/kg	5	999	1.570	0.205	0.428
AC-10mg/kg	anti-tip-chimer	5	999	1.275	0.393	0.602
AC-10mg/kg	anti-rsPilA	5	999	1.373	0.199	0.428
Ofloxacin	Saline	6	999	2.114	0.108	0.339
Ofloxacin	TS-30mg/kg	6	999	2.455	0.081	0.339
Ofloxacin	TS-15mg/kg	6	999	2.495	0.106	0.339
Ofloxacin	anti-tip-chimer	6	999	2.227	0.113	0.339
Ofloxacin	anti-rsPilA	6	999	2.252	0.104	0.339
Saline	TS-30mg/kg	6	999	1.065	0.508	0.663
Saline	TS-15mg/kg	6	999	0.742	1.000	1.000
Saline	anti-tip-chimer	6	999	0.776	0.698	0.866
Saline	anti-rsPilA	6	999	0.591	0.890	0.944
TS-30mg/kg	anti-tip-chimer	6	999	1.891	0.099	0.339
TS-30mg/kg	anti-rsPilA	6	999	1.592	0.196	0.428
TS-15mg/kg	TS-30mg/kg	6	999	2.453	0.102	0.339
TS-15mg/kg	anti-tip-chimer	6	999	0.836	0.783	0.927
TS-15mg/kg	anti-rsPilA	6	999	0.453	1.000	1.000
anti-tip-chimer	anti-rsPilA	6	999	0.555	0.803	0.927

Supplementary File 1
Beta Diversity Output

Fecal Weighted UniFrac-2DPT

PERMANOVA Pairwise Comparisons (999 permutations)

Group 1	Group 2	Sample size	Permutations	pseudo-F	p-value	q-value
AC-PO7d	Ofloxacin	4	999	5.379	0.350	0.548
AC-PO7d	Saline	5	999	9.592	0.113	0.370
AC-PO7d	TS-30mg/kg	5	999	5.078	0.104	0.370
AC-PO7d	TS-15mg/kg	5	999	8.194	0.086	0.370
AC-PO7d	anti-tip-chimer	5	999	5.340	0.093	0.370
AC-PO7d	anti-rsPilA	5	999	5.229	0.107	0.370
AC-5mg/kg	AC-PO7d	4	999	27.027	0.342	0.548
AC-5mg/kg	AC-10mg/kg	4	999	11.459	0.322	0.548
AC-5mg/kg	Ofloxacin	4	999	1.398	0.331	0.548
AC-5mg/kg	Saline	5	999	5.035	0.105	0.370
AC-5mg/kg	TS-30mg/kg	5	999	1.273	0.292	0.548
AC-5mg/kg	TS-15mg/kg	5	999	2.056	0.178	0.504
AC-5mg/kg	anti-tip-chimer	5	999	2.179	0.196	0.504
AC-5mg/kg	anti-rsPilA	5	999	1.646	0.193	0.504
AC-10mg/kg	AC-PO7d	4	999	20.814	0.309	0.548
AC-10mg/kg	Ofloxacin	4	999	3.611	0.293	0.548
AC-10mg/kg	Saline	5	999	13.210	0.107	0.370
AC-10mg/kg	TS-30mg/kg	5	999	4.465	0.104	0.370
AC-10mg/kg	TS-15mg/kg	5	999	8.879	0.105	0.370
AC-10mg/kg	anti-tip-chimer	5	999	6.888	0.099	0.370
AC-10mg/kg	anti-rsPilA	5	999	5.712	0.101	0.370
Ofloxacin	Saline	5	999	0.954	0.511	0.736
Ofloxacin	TS-30mg/kg	5	999	0.400	1.000	1.000
Ofloxacin	TS-15mg/kg	5	999	0.965	0.589	0.785
Ofloxacin	anti-tip-chimer	5	999	0.342	0.811	0.951
Ofloxacin	anti-rsPilA	5	999	0.402	0.915	0.969
Saline	TS-30mg/kg	6	999	1.485	0.283	0.548
Saline	TS-15mg/kg	6	999	1.623	0.286	0.548
Saline	anti-tip-chimer	6	999	0.919	0.587	0.785
Saline	anti-rsPilA	6	999	0.898	0.703	0.873
TS-30mg/kg	anti-tip-chimer	6	999	0.359	1.000	1.000
TS-30mg/kg	anti-rsPilA	6	999	0.492	0.913	0.969
TS-15mg/kg	TS-30mg/kg	6	999	0.656	0.819	0.951
TS-15mg/kg	anti-tip-chimer	6	999	1.043	0.495	0.736
TS-15mg/kg	anti-rsPilA	6	999	0.549	0.897	0.969
anti-tip-chimer	anti-rsPilA	6	999	0.488	0.698	0.873

Supplementary File 1
Beta Diversity Output

Fecal Weighted UniFrac-5DPT

PERMANOVA Pairwise Comparisons (999 permutations)

Group 1	Group 2	Sample size	Permutations	pseudo-F	p-value	q-value
AC-PO7d	Ofloxacin	6	999	5.014	0.109	0.196
AC-PO7d	Saline	6	999	3.928	0.089	0.196
AC-PO7d	TS-30mg/kg	6	999	3.174	0.091	0.196
AC-PO7d	TS-15mg/kg	6	999	6.389	0.104	0.196
AC-PO7d	anti-tip-chimer	6	999	1.968	0.118	0.196
AC-PO7d	anti-rsPilA	5	999	4.217	0.094	0.196
AC-5mg/kg	AC-PO7d	6	999	2.416	0.095	0.196
AC-5mg/kg	AC-10mg/kg	5	999	5.673	0.102	0.196
AC-5mg/kg	Ofloxacin	6	999	3.799	0.111	0.196
AC-5mg/kg	Saline	6	999	7.404	0.117	0.196
AC-5mg/kg	TS-30mg/kg	6	999	2.691	0.096	0.196
AC-5mg/kg	TS-15mg/kg	6	999	4.107	0.102	0.196
AC-5mg/kg	anti-tip-chimer	6	999	2.915	0.116	0.196
AC-5mg/kg	anti-rsPilA	5	999	2.876	0.216	0.338
AC-10mg/kg	AC-PO7d	5	999	4.728	0.105	0.196
AC-10mg/kg	Ofloxacin	5	999	11.376	0.106	0.196
AC-10mg/kg	Saline	5	999	11.260	0.103	0.196
AC-10mg/kg	TS-30mg/kg	5	999	4.167	0.102	0.196
AC-10mg/kg	TS-15mg/kg	5	999	11.942	0.091	0.196
AC-10mg/kg	anti-tip-chimer	5	999	4.390	0.109	0.196
AC-10mg/kg	anti-rsPilA	4	999	10.074	0.312	0.433
Ofloxacin	Saline	6	999	3.630	0.120	0.196
Ofloxacin	TS-30mg/kg	6	999	0.502	0.892	0.934
Ofloxacin	TS-15mg/kg	6	999	1.280	0.314	0.433
Ofloxacin	anti-tip-chimer	6	999	1.005	0.499	0.639
Ofloxacin	anti-rsPilA	5	999	0.505	0.795	0.894
Saline	TS-30mg/kg	6	999	1.631	0.325	0.433
Saline	TS-15mg/kg	6	999	3.137	0.104	0.196
Saline	anti-tip-chimer	6	999	0.823	0.515	0.639
Saline	anti-rsPilA	5	999	2.673	0.103	0.196
TS-30mg/kg	anti-tip-chimer	6	999	0.575	0.897	0.934
TS-30mg/kg	anti-rsPilA	5	999	0.319	0.908	0.934
TS-15mg/kg	TS-30mg/kg	6	999	0.421	1.000	1.000
TS-15mg/kg	anti-tip-chimer	6	999	1.426	0.315	0.433
TS-15mg/kg	anti-rsPilA	5	999	0.585	0.714	0.834
anti-tip-chimer	anti-rsPilA	5	999	0.844	0.718	0.834

Supplementary File 1
Beta Diversity Output

Fecal Weighted UniFrac-7DPT

PERMANOVA Pairwise Comparisons (999 permutations)

Group 1	Group 2	Sample size	Permutations	pseudo-F	p-value	q-value
AC-PO7d	Ofloxacin	6	999	2.388	0.095	0.410
AC-PO7d	Saline	5	999	2.412	0.182	0.471
AC-PO7d	TS-30mg/kg	6	999	2.338	0.089	0.410
AC-PO7d	TS-15mg/kg	6	999	2.719	0.114	0.410
AC-PO7d	anti-tip-chimer	5	999	2.581	0.102	0.410
AC-PO7d	anti-rsPilA	5	999	2.485	0.109	0.410
AC-5mg/kg	AC-PO7d	6	999	1.141	0.391	0.503
AC-5mg/kg	AC-10mg/kg	4	999	2.025	0.233	0.471
AC-5mg/kg	Ofloxacin	6	999	2.213	0.179	0.471
AC-5mg/kg	Saline	5	999	2.555	0.102	0.410
AC-5mg/kg	TS-30mg/kg	6	999	1.986	0.186	0.471
AC-5mg/kg	TS-15mg/kg	6	999	3.104	0.095	0.410
AC-5mg/kg	anti-tip-chimer	5	999	2.801	0.106	0.410
AC-5mg/kg	anti-rsPilA	5	999	2.516	0.087	0.410
AC-10mg/kg	AC-PO7d	4	999	1.010	0.505	0.607
AC-10mg/kg	Ofloxacin	4	999	3.171	0.265	0.471
AC-10mg/kg	Saline	3	999	2.811	0.328	0.471
AC-10mg/kg	TS-30mg/kg	4	999	1.463	0.506	0.607
AC-10mg/kg	TS-15mg/kg	4	999	3.619	0.264	0.471
AC-10mg/kg	anti-tip-chimer	3	999	1.506	0.333	0.471
AC-10mg/kg	anti-rsPilA	3	999	2.952	0.340	0.471
Ofloxacin	Saline	5	999	2.731	0.097	0.410
Ofloxacin	TS-30mg/kg	6	999	0.637	0.692	0.755
Ofloxacin	TS-15mg/kg	6	999	1.152	0.391	0.503
Ofloxacin	anti-tip-chimer	5	999	1.592	0.304	0.471
Ofloxacin	anti-rsPilA	5	999	1.345	0.320	0.471
Saline	TS-30mg/kg	5	999	1.241	0.311	0.471
Saline	TS-15mg/kg	5	999	2.534	0.222	0.471
Saline	anti-tip-chimer	4	999	1.142	0.670	0.755
Saline	anti-rsPilA	4	999	1.418	0.331	0.471
TS-30mg/kg	anti-tip-chimer	5	999	0.185	0.800	0.825
TS-30mg/kg	anti-rsPilA	5	999	0.351	1.000	1.000
TS-15mg/kg	TS-30mg/kg	6	999	0.517	0.802	0.825
TS-15mg/kg	anti-tip-chimer	5	999	1.361	0.187	0.471
TS-15mg/kg	anti-rsPilA	5	999	1.066	0.309	0.471
anti-tip-chimer	anti-rsPilA	4	999	0.585	0.672	0.755

Supplementary File 1
Beta Diversity Output

Fecal Weighted UniFrac-9DPT

PERMANOVA Pairwise Comparisons (999 permutations)

Group 1	Group 2	Sample size	Permutations	pseudo-F	p-value	q-value
AC-PO7d	Ofloxacin	5	999	4.105	0.234	0.468
AC-PO7d	Saline	3	999	2.374	0.326	0.521
AC-PO7d	TS-30mg/kg	5	999	3.258	0.098	0.448
AC-PO7d	TS-15mg/kg	5	999	2.097	0.089	0.448
AC-PO7d	anti-tip-chimer	5	999	3.834	0.108	0.448
AC-PO7d	anti-rsPilA	4	999	6.652	0.345	0.521
AC-5mg/kg	AC-PO7d	5	999	1.872	0.219	0.464
AC-5mg/kg	AC-10mg/kg	5	999	1.382	0.510	0.633
AC-5mg/kg	Ofloxacin	6	999	2.159	0.186	0.461
AC-5mg/kg	Saline	4	999	1.561	0.264	0.500
AC-5mg/kg	TS-30mg/kg	6	999	2.519	0.086	0.448
AC-5mg/kg	TS-15mg/kg	6	999	1.872	0.084	0.448
AC-5mg/kg	anti-tip-chimer	6	999	2.411	0.107	0.448
AC-5mg/kg	anti-rsPilA	5	999	2.885	0.112	0.448
AC-10mg/kg	AC-PO7d	4	999	2.448	0.347	0.521
AC-10mg/kg	Ofloxacin	5	999	2.288	0.183	0.461
AC-10mg/kg	Saline	3	999	1.718	0.667	0.784
AC-10mg/kg	TS-30mg/kg	5	999	2.014	0.127	0.457
AC-10mg/kg	TS-15mg/kg	5	999	1.594	0.191	0.461
AC-10mg/kg	anti-tip-chimer	5	999	2.875	0.091	0.448
AC-10mg/kg	anti-rsPilA	4	999	3.500	0.319	0.521
Ofloxacin	Saline	4	999	1.470	0.491	0.633
Ofloxacin	TS-30mg/kg	6	999	1.805	0.183	0.461
Ofloxacin	TS-15mg/kg	6	999	1.540	0.192	0.461
Ofloxacin	anti-tip-chimer	6	999	2.262	0.097	0.448
Ofloxacin	anti-rsPilA	5	999	1.192	0.505	0.633
Saline	TS-30mg/kg	4	999	0.676	0.739	0.831
Saline	TS-15mg/kg	4	999	0.406	1.000	1.000
Saline	anti-tip-chimer	4	999	0.790	1.000	1.000
Saline	anti-rsPilA	3	999	3.585	0.344	0.521
TS-30mg/kg	anti-tip-chimer	6	999	1.182	0.392	0.547
TS-30mg/kg	anti-rsPilA	5	999	0.728	0.806	0.879
TS-15mg/kg	TS-30mg/kg	6	999	0.604	0.675	0.784
TS-15mg/kg	anti-tip-chimer	6	999	0.601	0.898	0.951
TS-15mg/kg	anti-rsPilA	5	999	0.977	0.395	0.547
anti-tip-chimer	anti-rsPilA	5	999	1.969	0.218	0.464

Supplementary File 1
Beta Diversity Output

Fecal Unweighted UniFrac-Baseline

PERMANOVA Pairwise Comparisons (999 permutations)

Group 1	Group 2	Sample size	Permutations	pseudo-F	p-value	q-value
AC-PO7d	Ofloxacin	6	999	2.835	0.103	0.248
AC-PO7d	Saline	6	999	2.539	0.109	0.248
AC-PO7d	TS-30mg/kg	6	999	2.570	0.119	0.252
AC-PO7d	TS-15mg/kg	6	999	1.749	0.093	0.248
AC-PO7d	anti-tip-chimer	6	999	2.094	0.103	0.248
AC-PO7d	anti-rsPilA	6	999	1.789	0.102	0.248
AC-5mg/kg	AC-PO7d	5	999	1.852	0.082	0.248
AC-5mg/kg	AC-10mg/kg	4	999	1.270	0.333	0.500
AC-5mg/kg	Ofloxacin	5	999	0.951	0.701	0.788
AC-5mg/kg	Saline	5	999	0.943	0.486	0.661
AC-5mg/kg	TS-30mg/kg	5	999	1.025	0.496	0.661
AC-5mg/kg	TS-15mg/kg	5	999	1.203	0.305	0.477
AC-5mg/kg	anti-tip-chimer	5	999	0.890	0.908	0.908
AC-5mg/kg	anti-rsPilA	5	999	0.949	0.596	0.742
AC-10mg/kg	AC-PO7d	5	999	2.954	0.110	0.248
AC-10mg/kg	Ofloxacin	5	999	0.957	0.620	0.744
AC-10mg/kg	Saline	5	999	1.273	0.088	0.248
AC-10mg/kg	TS-30mg/kg	5	999	1.446	0.099	0.248
AC-10mg/kg	TS-15mg/kg	5	999	1.572	0.103	0.248
AC-10mg/kg	anti-tip-chimer	5	999	1.208	0.222	0.381
AC-10mg/kg	anti-rsPilA	5	999	1.197	0.195	0.369
Ofloxacin	Saline	6	999	0.838	0.805	0.852
Ofloxacin	TS-30mg/kg	6	999	1.232	0.099	0.248
Ofloxacin	TS-15mg/kg	6	999	1.691	0.091	0.248
Ofloxacin	anti-tip-chimer	6	999	1.172	0.101	0.248
Ofloxacin	anti-rsPilA	6	999	1.398	0.108	0.248
Saline	TS-30mg/kg	6	999	1.099	0.272	0.445
Saline	TS-15mg/kg	6	999	1.398	0.093	0.248
Saline	anti-tip-chimer	6	999	1.126	0.212	0.381
Saline	anti-rsPilA	6	999	1.161	0.406	0.585
TS-30mg/kg	anti-tip-chimer	6	999	0.970	0.722	0.788
TS-30mg/kg	anti-rsPilA	6	999	1.090	0.174	0.348
TS-15mg/kg	TS-30mg/kg	6	999	1.399	0.101	0.248
TS-15mg/kg	anti-tip-chimer	6	999	0.989	0.598	0.742
TS-15mg/kg	anti-rsPilA	6	999	0.826	0.907	0.908
anti-tip-chimer	anti-rsPilA	6	999	0.915	0.641	0.744

Supplementary File 1
Beta Diversity Output

Fecal Unweighted UniFrac-2DPT

PERMANOVA Pairwise Comparisons (999 permutations)

Group 1	Group 2	Sample size	Permutations	pseudo-F	p-value	q-value
AC-PO7d	Ofloxacin	4	999	1.967	0.323	0.639
AC-PO7d	Saline	5	999	2.255	0.108	0.508
AC-PO7d	TS-30mg/kg	5	999	3.780	0.096	0.508
AC-PO7d	TS-15mg/kg	5	999	1.602	0.203	0.609
AC-PO7d	anti-tip-chimer	5	999	3.521	0.127	0.508
AC-PO7d	anti-rsPilA	5	999	2.358	0.100	0.508
AC-5mg/kg	AC-PO7d	4	999	3.267	0.320	0.639
AC-5mg/kg	AC-10mg/kg	4	999	1.824	0.330	0.639
AC-5mg/kg	Ofloxacin	4	999	0.815	0.649	0.831
AC-5mg/kg	Saline	5	999	1.091	0.406	0.639
AC-5mg/kg	TS-30mg/kg	5	999	1.458	0.089	0.508
AC-5mg/kg	TS-15mg/kg	5	999	0.912	0.596	0.816
AC-5mg/kg	anti-tip-chimer	5	999	1.520	0.172	0.609
AC-5mg/kg	anti-rsPilA	5	999	0.835	0.716	0.831
AC-10mg/kg	AC-PO7d	4	999	1.464	0.348	0.639
AC-10mg/kg	Ofloxacin	4	999	1.280	0.348	0.639
AC-10mg/kg	Saline	5	999	1.680	0.102	0.508
AC-10mg/kg	TS-30mg/kg	5	999	2.561	0.106	0.508
AC-10mg/kg	TS-15mg/kg	5	999	1.206	0.401	0.639
AC-10mg/kg	anti-tip-chimer	5	999	2.402	0.113	0.508
AC-10mg/kg	anti-rsPilA	5	999	1.669	0.111	0.508
Ofloxacin	Saline	5	999	0.734	0.612	0.816
Ofloxacin	TS-30mg/kg	5	999	1.042	0.401	0.639
Ofloxacin	TS-15mg/kg	5	999	0.671	1.000	1.000
Ofloxacin	anti-tip-chimer	5	999	1.021	0.704	0.831
Ofloxacin	anti-rsPilA	5	999	0.664	1.000	1.000
Saline	TS-30mg/kg	6	999	1.271	0.193	0.609
Saline	TS-15mg/kg	6	999	0.833	0.818	0.920
Saline	anti-tip-chimer	6	999	1.021	0.488	0.732
Saline	anti-rsPilA	6	999	0.719	0.891	0.972
TS-30mg/kg	anti-tip-chimer	6	999	1.033	0.408	0.639
TS-30mg/kg	anti-rsPilA	6	999	1.050	0.390	0.639
TS-15mg/kg	TS-30mg/kg	6	999	0.952	0.695	0.831
TS-15mg/kg	anti-tip-chimer	6	999	1.046	0.302	0.639
TS-15mg/kg	anti-rsPilA	6	999	0.680	1.000	1.000
anti-tip-chimer	anti-rsPilA	6	999	0.889	0.600	0.816

Supplementary File 1
Beta Diversity Output

Fecal Unweighted UniFrac-5DPT

PERMANOVA Pairwise Comparisons (999 permutations)

Group 1	Group 2	Sample size	Permutations	pseudo-F	p-value	q-value
AC-PO7d	Ofloxacin	6	999	1.885	0.120	0.332
AC-PO7d	Saline	6	999	1.482	0.105	0.332
AC-PO7d	TS-30mg/kg	6	999	1.623	0.091	0.332
AC-PO7d	TS-15mg/kg	6	999	1.213	0.305	0.451
AC-PO7d	anti-tip-chimer	6	999	2.098	0.087	0.332
AC-PO7d	anti-rsPilA	5	999	1.273	0.237	0.388
AC-5mg/kg	AC-PO7d	6	999	1.259	0.415	0.553
AC-5mg/kg	AC-10mg/kg	5	999	0.690	0.804	0.911
AC-5mg/kg	Ofloxacin	6	999	2.680	0.099	0.332
AC-5mg/kg	Saline	6	999	2.091	0.100	0.332
AC-5mg/kg	TS-30mg/kg	6	999	2.484	0.092	0.332
AC-5mg/kg	TS-15mg/kg	6	999	1.615	0.185	0.380
AC-5mg/kg	anti-tip-chimer	6	999	3.012	0.099	0.332
AC-5mg/kg	anti-rsPilA	5	999	1.839	0.188	0.380
AC-10mg/kg	AC-PO7d	5	999	1.797	0.199	0.380
AC-10mg/kg	Ofloxacin	5	999	4.065	0.094	0.332
AC-10mg/kg	Saline	5	999	3.363	0.096	0.332
AC-10mg/kg	TS-30mg/kg	5	999	3.683	0.099	0.332
AC-10mg/kg	TS-15mg/kg	5	999	2.646	0.103	0.332
AC-10mg/kg	anti-tip-chimer	5	999	4.481	0.113	0.332
AC-10mg/kg	anti-rsPilA	4	999	3.029	0.313	0.451
Ofloxacin	Saline	6	999	1.154	0.211	0.380
Ofloxacin	TS-30mg/kg	6	999	1.112	0.188	0.380
Ofloxacin	TS-15mg/kg	6	999	1.156	0.302	0.451
Ofloxacin	anti-tip-chimer	6	999	1.303	0.202	0.380
Ofloxacin	anti-rsPilA	5	999	1.031	0.386	0.534
Saline	TS-30mg/kg	6	999	0.873	0.812	0.911
Saline	TS-15mg/kg	6	999	0.711	0.911	0.911
Saline	anti-tip-chimer	6	999	1.291	0.196	0.380
Saline	anti-rsPilA	5	999	0.692	0.910	0.911
TS-30mg/kg	anti-tip-chimer	6	999	0.955	0.600	0.771
TS-30mg/kg	anti-rsPilA	5	999	0.702	0.890	0.911
TS-15mg/kg	TS-30mg/kg	6	999	0.828	0.801	0.911
TS-15mg/kg	anti-tip-chimer	6	999	1.424	0.222	0.381
TS-15mg/kg	anti-rsPilA	5	999	0.765	0.710	0.881
anti-tip-chimer	anti-rsPilA	5	999	0.850	0.892	0.911

Supplementary File 1
Beta Diversity Output

Fecal Unweighted UniFrac-7DPT

PERMANOVA Pairwise Comparisons (999 permutations)

Group 1	Group 2	Sample size	Permutations	pseudo-F	p-value	q-value
AC-PO7d	Ofloxacin	6	999	2.897	0.090	0.367
AC-PO7d	Saline	5	999	2.094	0.086	0.367
AC-PO7d	TS-30mg/kg	6	999	3.631	0.112	0.367
AC-PO7d	TS-15mg/kg	6	999	2.454	0.097	0.367
AC-PO7d	anti-tip-chimer	5	999	3.260	0.106	0.367
AC-PO7d	anti-rsPilA	5	999	2.887	0.098	0.367
AC-5mg/kg	AC-PO7d	6	999	1.274	0.328	0.467
AC-5mg/kg	AC-10mg/kg	4	999	0.904	0.759	0.804
AC-5mg/kg	Ofloxacin	6	999	2.174	0.096	0.367
AC-5mg/kg	Saline	5	999	1.473	0.085	0.367
AC-5mg/kg	TS-30mg/kg	6	999	2.528	0.097	0.367
AC-5mg/kg	TS-15mg/kg	6	999	1.566	0.129	0.387
AC-5mg/kg	anti-tip-chimer	5	999	2.494	0.092	0.367
AC-5mg/kg	anti-rsPilA	5	999	2.122	0.106	0.367
AC-10mg/kg	AC-PO7d	4	999	1.199	0.496	0.616
AC-10mg/kg	Ofloxacin	4	999	1.912	0.243	0.467
AC-10mg/kg	Saline	3	999	1.400	0.352	0.469
AC-10mg/kg	TS-30mg/kg	4	999	2.249	0.277	0.467
AC-10mg/kg	TS-15mg/kg	4	999	1.686	0.250	0.467
AC-10mg/kg	anti-tip-chimer	3	999	2.336	0.330	0.467
AC-10mg/kg	anti-rsPilA	3	999	1.770	0.324	0.467
Ofloxacin	Saline	5	999	1.049	0.394	0.507
Ofloxacin	TS-30mg/kg	6	999	1.169	0.214	0.453
Ofloxacin	TS-15mg/kg	6	999	1.571	0.199	0.453
Ofloxacin	anti-tip-chimer	5	999	1.086	0.293	0.467
Ofloxacin	anti-rsPilA	5	999	1.169	0.185	0.453
Saline	TS-30mg/kg	5	999	0.925	0.688	0.751
Saline	TS-15mg/kg	5	999	0.809	0.597	0.716
Saline	anti-tip-chimer	4	999	1.103	0.337	0.467
Saline	anti-rsPilA	4	999	0.881	0.657	0.751
TS-30mg/kg	anti-tip-chimer	5	999	0.791	0.903	0.903
TS-30mg/kg	anti-rsPilA	5	999	0.872	0.899	0.903
TS-15mg/kg	TS-30mg/kg	6	999	1.386	0.195	0.453
TS-15mg/kg	anti-tip-chimer	5	999	1.805	0.203	0.453
TS-15mg/kg	anti-rsPilA	5	999	1.384	0.282	0.467
anti-tip-chimer	anti-rsPilA	4	999	0.819	0.669	0.751

Supplementary File 1
Beta Diversity Output

Fecal Unweighted UniFrac-9DPT

PERMANOVA Pairwise Comparisons (999 permutations)

Group 1	Group 2	Sample size	Permutations	pseudo-F	p-value	q-value
AC-PO7d	Ofloxacin	5	999	1.781	0.186	0.497
AC-PO7d	Saline	3	999	1.434	0.346	0.548
AC-PO7d	TS-30mg/kg	5	999	2.759	0.114	0.373
AC-PO7d	TS-15mg/kg	5	999	1.450	0.113	0.373
AC-PO7d	anti-tip-chimer	5	999	2.700	0.073	0.373
AC-PO7d	anti-rsPilA	4	999	1.744	0.350	0.548
AC-5mg/kg	AC-PO7d	5	999	0.947	0.409	0.566
AC-5mg/kg	AC-10mg/kg	5	999	1.092	0.607	0.728
AC-5mg/kg	Ofloxacin	6	999	1.732	0.201	0.497
AC-5mg/kg	Saline	4	999	1.373	0.237	0.519
AC-5mg/kg	TS-30mg/kg	6	999	2.456	0.083	0.373
AC-5mg/kg	TS-15mg/kg	6	999	1.626	0.108	0.373
AC-5mg/kg	anti-tip-chimer	6	999	2.705	0.100	0.373
AC-5mg/kg	anti-rsPilA	5	999	1.621	0.097	0.373
AC-10mg/kg	AC-PO7d	4	999	1.263	0.339	0.548
AC-10mg/kg	Ofloxacin	5	999	1.890	0.207	0.497
AC-10mg/kg	Saline	3	999	1.987	0.346	0.548
AC-10mg/kg	TS-30mg/kg	5	999	3.151	0.100	0.373
AC-10mg/kg	TS-15mg/kg	5	999	1.734	0.112	0.373
AC-10mg/kg	anti-tip-chimer	5	999	3.007	0.098	0.373
AC-10mg/kg	anti-rsPilA	4	999	2.028	0.338	0.548
Ofloxacin	Saline	4	999	0.919	0.505	0.673
Ofloxacin	TS-30mg/kg	6	999	1.411	0.096	0.373
Ofloxacin	TS-15mg/kg	6	999	0.874	0.585	0.726
Ofloxacin	anti-tip-chimer	6	999	1.062	0.530	0.681
Ofloxacin	anti-rsPilA	5	999	0.969	0.676	0.785
Saline	TS-30mg/kg	4	999	1.507	0.261	0.522
Saline	TS-15mg/kg	4	999	0.911	0.738	0.830
Saline	anti-tip-chimer	4	999	1.550	0.245	0.519
Saline	anti-rsPilA	3	999	0.955	1.000	1.000
TS-30mg/kg	anti-tip-chimer	6	999	1.190	0.191	0.497
TS-30mg/kg	anti-rsPilA	5	999	0.811	0.904	0.967
TS-15mg/kg	TS-30mg/kg	6	999	0.988	0.369	0.554
TS-15mg/kg	anti-tip-chimer	6	999	1.062	0.394	0.566
TS-15mg/kg	anti-rsPilA	5	999	0.721	0.913	0.967
anti-tip-chimer	anti-rsPilA	5	999	0.836	1.000	1.000

Supplementary File 1
Beta Diversity Output

Fecal Bray-Curtis-Baseline

PERMANOVA Pairwise Comparisons (999 permutations)

Group 1	Group 2	Sample size	Permutations	pseudo-F	p-value	q-value
AC-PO7d	Ofloxacin	6	999	1.496	0.106	0.345
AC-PO7d	Saline	6	999	1.079	0.299	0.461
AC-PO7d	TS-30mg/kg	6	999	1.175	0.289	0.461
AC-PO7d	TS-15mg/kg	6	999	1.423	0.095	0.345
AC-PO7d	anti-tip-chimer	6	999	1.240	0.093	0.345
AC-PO7d	anti-rsPilA	6	999	1.115	0.299	0.461
AC-5mg/kg	AC-PO7d	5	999	1.354	0.201	0.449
AC-5mg/kg	AC-10mg/kg	4	999	1.294	0.331	0.477
AC-5mg/kg	Ofloxacin	5	999	1.484	0.097	0.345
AC-5mg/kg	Saline	5	999	0.801	0.798	0.890
AC-5mg/kg	TS-30mg/kg	5	999	1.346	0.102	0.345
AC-5mg/kg	TS-15mg/kg	5	999	1.442	0.099	0.345
AC-5mg/kg	anti-tip-chimer	5	999	0.822	0.902	0.902
AC-5mg/kg	anti-rsPilA	5	999	0.991	0.590	0.752
AC-10mg/kg	AC-PO7d	5	999	1.469	0.093	0.345
AC-10mg/kg	Ofloxacin	5	999	1.202	0.190	0.449
AC-10mg/kg	Saline	5	999	0.961	0.606	0.752
AC-10mg/kg	TS-30mg/kg	5	999	1.306	0.115	0.345
AC-10mg/kg	TS-15mg/kg	5	999	1.063	0.299	0.461
AC-10mg/kg	anti-tip-chimer	5	999	0.918	0.486	0.648
AC-10mg/kg	anti-rsPilA	5	999	0.888	0.816	0.890
Ofloxacin	Saline	6	999	1.275	0.198	0.449
Ofloxacin	TS-30mg/kg	6	999	1.318	0.209	0.449
Ofloxacin	TS-15mg/kg	6	999	1.759	0.082	0.345
Ofloxacin	anti-tip-chimer	6	999	1.351	0.113	0.345
Ofloxacin	anti-rsPilA	6	999	1.288	0.240	0.461
Saline	TS-30mg/kg	6	999	0.899	0.802	0.890
Saline	TS-15mg/kg	6	999	1.100	0.287	0.461
Saline	anti-tip-chimer	6	999	0.983	0.382	0.529
Saline	anti-rsPilA	6	999	0.824	0.893	0.902
TS-30mg/kg	anti-tip-chimer	6	999	1.312	0.099	0.345
TS-30mg/kg	anti-rsPilA	6	999	1.087	0.212	0.449
TS-15mg/kg	TS-30mg/kg	6	999	1.192	0.092	0.345
TS-15mg/kg	anti-tip-chimer	6	999	1.155	0.307	0.461
TS-15mg/kg	anti-rsPilA	6	999	0.866	0.887	0.902
anti-tip-chimer	anti-rsPilA	6	999	0.857	0.789	0.890

Supplementary File 1
Beta Diversity Output

Fecal Bray-Curtis-2DPT

PERMANOVA Pairwise Comparisons (999 permutations)

Group 1	Group 2	Sample size	Permutations	pseudo-F	p-value	q-value
AC-PO7d	Ofloxacin	4	999	2.936	0.340	0.650
AC-PO7d	Saline	5	999	3.722	0.100	0.414
AC-PO7d	TS-30mg/kg	5	999	3.472	0.113	0.414
AC-PO7d	TS-15mg/kg	5	999	3.499	0.115	0.414
AC-PO7d	anti-tip-chimer	5	999	2.931	0.090	0.414
AC-PO7d	anti-rsPilA	5	999	2.896	0.080	0.414
AC-5mg/kg	AC-PO7d	4	999	6.279	0.343	0.650
AC-5mg/kg	AC-10mg/kg	4	999	3.996	0.340	0.650
AC-5mg/kg	Ofloxacin	4	999	1.033	0.658	0.877
AC-5mg/kg	Saline	5	999	1.556	0.209	0.579
AC-5mg/kg	TS-30mg/kg	5	999	1.571	0.194	0.579
AC-5mg/kg	TS-15mg/kg	5	999	1.439	0.176	0.576
AC-5mg/kg	anti-tip-chimer	5	999	1.158	0.289	0.650
AC-5mg/kg	anti-rsPilA	5	999	1.033	0.494	0.847
AC-10mg/kg	AC-PO7d	4	999	3.935	0.335	0.650
AC-10mg/kg	Ofloxacin	4	999	1.761	0.322	0.650
AC-10mg/kg	Saline	5	999	2.847	0.112	0.414
AC-10mg/kg	TS-30mg/kg	5	999	2.466	0.101	0.414
AC-10mg/kg	TS-15mg/kg	5	999	2.519	0.084	0.414
AC-10mg/kg	anti-tip-chimer	5	999	2.116	0.104	0.414
AC-10mg/kg	anti-rsPilA	5	999	1.988	0.102	0.414
Ofloxacin	Saline	5	999	0.576	0.601	0.847
Ofloxacin	TS-30mg/kg	5	999	1.022	0.527	0.847
Ofloxacin	TS-15mg/kg	5	999	0.872	0.612	0.847
Ofloxacin	anti-tip-chimer	5	999	0.661	0.886	0.927
Ofloxacin	anti-rsPilA	5	999	0.735	1.000	1.000
Saline	TS-30mg/kg	6	999	0.961	0.607	0.847
Saline	TS-15mg/kg	6	999	1.027	0.386	0.695
Saline	anti-tip-chimer	6	999	0.769	0.588	0.847
Saline	anti-rsPilA	6	999	0.922	0.703	0.889
TS-30mg/kg	anti-tip-chimer	6	999	0.873	0.795	0.914
TS-30mg/kg	anti-rsPilA	6	999	0.863	0.812	0.914
TS-15mg/kg	TS-30mg/kg	6	999	0.797	0.888	0.927
TS-15mg/kg	anti-tip-chimer	6	999	0.999	0.808	0.914
TS-15mg/kg	anti-rsPilA	6	999	0.720	0.901	0.927
anti-tip-chimer	anti-rsPilA	6	999	0.811	0.716	0.889

Supplementary File 1
Beta Diversity Output

Fecal Bray-Curtis-5DPT

PERMANOVA Pairwise Comparisons (999 permutations)

Group 1	Group 2	Sample size	Permutations	pseudo-F	p-value	q-value
AC-PO7d	Ofloxacin	6	999	3.099	0.103	0.226
AC-PO7d	Saline	6	999	3.228	0.091	0.226
AC-PO7d	TS-30mg/kg	6	999	2.804	0.102	0.226
AC-PO7d	TS-15mg/kg	6	999	2.841	0.092	0.226
AC-PO7d	anti-tip-chimer	6	999	2.141	0.098	0.226
AC-PO7d	anti-rsPilA	5	999	2.312	0.099	0.226
AC-5mg/kg	AC-PO7d	6	999	1.439	0.187	0.337
AC-5mg/kg	AC-10mg/kg	5	999	0.988	0.487	0.605
AC-5mg/kg	Ofloxacin	6	999	2.172	0.098	0.226
AC-5mg/kg	Saline	6	999	2.798	0.105	0.226
AC-5mg/kg	TS-30mg/kg	6	999	2.407	0.101	0.226
AC-5mg/kg	TS-15mg/kg	6	999	2.047	0.127	0.241
AC-5mg/kg	anti-tip-chimer	6	999	1.964	0.105	0.226
AC-5mg/kg	anti-rsPilA	5	999	1.542	0.311	0.477
AC-10mg/kg	AC-PO7d	5	999	1.782	0.096	0.226
AC-10mg/kg	Ofloxacin	5	999	2.615	0.097	0.226
AC-10mg/kg	Saline	5	999	2.659	0.113	0.226
AC-10mg/kg	TS-30mg/kg	5	999	2.246	0.107	0.226
AC-10mg/kg	TS-15mg/kg	5	999	2.318	0.091	0.226
AC-10mg/kg	anti-tip-chimer	5	999	1.945	0.096	0.226
AC-10mg/kg	anti-rsPilA	4	999	1.814	0.318	0.477
Ofloxacin	Saline	6	999	1.840	0.110	0.226
Ofloxacin	TS-30mg/kg	6	999	1.263	0.098	0.226
Ofloxacin	TS-15mg/kg	6	999	1.160	0.203	0.348
Ofloxacin	anti-tip-chimer	6	999	1.048	0.293	0.477
Ofloxacin	anti-rsPilA	5	999	0.895	0.692	0.830
Saline	TS-30mg/kg	6	999	1.140	0.418	0.579
Saline	TS-15mg/kg	6	999	1.069	0.392	0.564
Saline	anti-tip-chimer	6	999	0.930	0.470	0.605
Saline	anti-rsPilA	5	999	0.879	0.892	0.955
TS-30mg/kg	anti-tip-chimer	6	999	0.887	0.902	0.955
TS-30mg/kg	anti-rsPilA	5	999	0.771	1.000	1.000
TS-15mg/kg	TS-30mg/kg	6	999	0.863	0.796	0.924
TS-15mg/kg	anti-tip-chimer	6	999	1.025	0.487	0.605
TS-15mg/kg	anti-rsPilA	5	999	0.635	1.000	1.000
anti-tip-chimer	anti-rsPilA	5	999	0.680	0.892	0.955

Supplementary File 1
Beta Diversity Output

Fecal Bray-Curtis-7DPT

PERMANOVA Pairwise Comparisons (999 permutations)

Group 1	Group 2	Sample size	Permutations	pseudo-F	p-value	q-value
AC-PO7d	Ofloxacin	6	999	2.562	0.103	0.275
AC-PO7d	Saline	5	999	2.715	0.119	0.275
AC-PO7d	TS-30mg/kg	6	999	2.925	0.113	0.275
AC-PO7d	TS-15mg/kg	6	999	3.225	0.105	0.275
AC-PO7d	anti-tip-chimer	5	999	2.558	0.101	0.275
AC-PO7d	anti-rsPilA	5	999	2.649	0.100	0.275
AC-5mg/kg	AC-PO7d	6	999	1.326	0.102	0.275
AC-5mg/kg	AC-10mg/kg	4	999	1.107	0.492	0.613
AC-5mg/kg	Ofloxacin	6	999	1.871	0.101	0.275
AC-5mg/kg	Saline	5	999	1.812	0.114	0.275
AC-5mg/kg	TS-30mg/kg	6	999	2.164	0.096	0.275
AC-5mg/kg	TS-15mg/kg	6	999	2.018	0.092	0.275
AC-5mg/kg	anti-tip-chimer	5	999	1.832	0.110	0.275
AC-5mg/kg	anti-rsPilA	5	999	1.858	0.122	0.275
AC-10mg/kg	AC-PO7d	4	999	1.741	0.246	0.426
AC-10mg/kg	Ofloxacin	4	999	2.013	0.268	0.426
AC-10mg/kg	Saline	3	999	1.684	0.339	0.469
AC-10mg/kg	TS-30mg/kg	4	999	1.959	0.261	0.426
AC-10mg/kg	TS-15mg/kg	4	999	2.128	0.272	0.426
AC-10mg/kg	anti-tip-chimer	3	999	1.532	0.338	0.469
AC-10mg/kg	anti-rsPilA	3	999	1.696	0.322	0.469
Ofloxacin	Saline	5	999	1.617	0.087	0.275
Ofloxacin	TS-30mg/kg	6	999	1.311	0.105	0.275
Ofloxacin	TS-15mg/kg	6	999	1.642	0.206	0.394
Ofloxacin	anti-tip-chimer	5	999	1.294	0.100	0.275
Ofloxacin	anti-rsPilA	5	999	1.142	0.208	0.394
Saline	TS-30mg/kg	5	999	0.995	0.710	0.775
Saline	TS-15mg/kg	5	999	0.998	0.673	0.757
Saline	anti-tip-chimer	4	999	0.973	0.669	0.757
Saline	anti-rsPilA	4	999	0.929	0.659	0.757
TS-30mg/kg	anti-tip-chimer	5	999	0.707	0.888	0.929
TS-30mg/kg	anti-rsPilA	5	999	0.864	0.903	0.929
TS-15mg/kg	TS-30mg/kg	6	999	1.021	0.485	0.613
TS-15mg/kg	anti-tip-chimer	5	999	1.293	0.195	0.394
TS-15mg/kg	anti-rsPilA	5	999	1.114	0.494	0.613
anti-tip-chimer	anti-rsPilA	4	999	0.708	1.000	1.000

Supplementary File 1
Beta Diversity Output

Fecal Bray-Curtis-9DPT

PERMANOVA Pairwise Comparisons (999 permutations)

Group 1	Group 2	Sample size	Permutations	pseudo-F	p-value	q-value
AC-PO7d	Ofloxacin	5	999	1.529	0.203	0.542
AC-PO7d	Saline	3	999	1.173	0.322	0.542
AC-PO7d	TS-30mg/kg	5	999	2.198	0.097	0.374
AC-PO7d	TS-15mg/kg	5	999	1.564	0.103	0.374
AC-PO7d	anti-tip-chimer	5	999	1.796	0.090	0.374
AC-PO7d	anti-rsPilA	4	999	2.203	0.334	0.542
AC-5mg/kg	AC-PO7d	5	999	0.872	0.905	0.958
AC-5mg/kg	AC-10mg/kg	5	999	1.128	0.283	0.542
AC-5mg/kg	Ofloxacin	6	999	1.285	0.297	0.542
AC-5mg/kg	Saline	4	999	1.249	0.252	0.542
AC-5mg/kg	TS-30mg/kg	6	999	2.220	0.097	0.374
AC-5mg/kg	TS-15mg/kg	6	999	1.565	0.101	0.374
AC-5mg/kg	anti-tip-chimer	6	999	1.848	0.104	0.374
AC-5mg/kg	anti-rsPilA	5	999	1.997	0.103	0.374
AC-10mg/kg	AC-PO7d	4	999	1.104	0.346	0.542
AC-10mg/kg	Ofloxacin	5	999	1.190	0.315	0.542
AC-10mg/kg	Saline	3	999	1.120	0.344	0.542
AC-10mg/kg	TS-30mg/kg	5	999	1.962	0.098	0.374
AC-10mg/kg	TS-15mg/kg	5	999	1.349	0.104	0.374
AC-10mg/kg	anti-tip-chimer	5	999	1.713	0.101	0.374
AC-10mg/kg	anti-rsPilA	4	999	1.707	0.327	0.542
Ofloxacin	Saline	4	999	1.076	0.482	0.563
Ofloxacin	TS-30mg/kg	6	999	1.470	0.200	0.542
Ofloxacin	TS-15mg/kg	6	999	0.977	0.508	0.563
Ofloxacin	anti-tip-chimer	6	999	1.096	0.297	0.542
Ofloxacin	anti-rsPilA	5	999	1.122	0.516	0.563
Saline	TS-30mg/kg	4	999	1.092	0.512	0.563
Saline	TS-15mg/kg	4	999	0.738	1.000	1.000
Saline	anti-tip-chimer	4	999	0.953	1.000	1.000
Saline	anti-rsPilA	3	999	2.066	0.325	0.542
TS-30mg/kg	anti-tip-chimer	6	999	1.007	0.514	0.563
TS-30mg/kg	anti-rsPilA	5	999	1.031	0.494	0.563
TS-15mg/kg	TS-30mg/kg	6	999	0.984	0.512	0.563
TS-15mg/kg	anti-tip-chimer	6	999	1.047	0.499	0.563
TS-15mg/kg	anti-rsPilA	5	999	1.042	0.415	0.563
anti-tip-chimer	anti-rsPilA	5	999	1.012	0.484	0.563

Supplementary File 1
Beta Diversity Output

Fecal Jaccard-Baseline

PERMANOVA Pairwise Comparisons (999 permutations)

Group 1	Group 2	Sample size	Permutations	pseudo-F	p-value	q-value
AC-PO7d	Ofloxacin	6	999	1.972	0.089	0.347
AC-PO7d	Saline	6	999	1.826	0.092	0.347
AC-PO7d	TS-30mg/kg	6	999	1.883	0.104	0.347
AC-PO7d	TS-15mg/kg	6	999	1.486	0.095	0.347
AC-PO7d	anti-tip-chimer	6	999	1.616	0.104	0.347
AC-PO7d	anti-rsPilA	6	999	1.459	0.093	0.347
AC-5mg/kg	AC-PO7d	5	999	1.555	0.106	0.347
AC-5mg/kg	AC-10mg/kg	4	999	1.131	0.340	0.726
AC-5mg/kg	Ofloxacin	5	999	1.016	0.507	0.830
AC-5mg/kg	Saline	5	999	0.895	0.893	0.974
AC-5mg/kg	TS-30mg/kg	5	999	0.989	0.714	0.889
AC-5mg/kg	TS-15mg/kg	5	999	1.016	0.499	0.830
AC-5mg/kg	anti-tip-chimer	5	999	0.851	1.000	1.000
AC-5mg/kg	anti-rsPilA	5	999	0.907	1.000	1.000
AC-10mg/kg	AC-PO7d	5	999	1.937	0.121	0.363
AC-10mg/kg	Ofloxacin	5	999	0.996	0.545	0.853
AC-10mg/kg	Saline	5	999	1.058	0.302	0.726
AC-10mg/kg	TS-30mg/kg	5	999	1.189	0.189	0.523
AC-10mg/kg	TS-15mg/kg	5	999	1.170	0.098	0.347
AC-10mg/kg	anti-tip-chimer	5	999	0.992	0.693	0.889
AC-10mg/kg	anti-rsPilA	5	999	0.954	0.799	0.930
Ofloxacin	Saline	6	999	0.983	0.676	0.889
Ofloxacin	TS-30mg/kg	6	999	1.029	0.584	0.876
Ofloxacin	TS-15mg/kg	6	999	1.306	0.103	0.347
Ofloxacin	anti-tip-chimer	6	999	1.040	0.492	0.830
Ofloxacin	anti-rsPilA	6	999	1.006	0.501	0.830
Saline	TS-30mg/kg	6	999	0.977	0.716	0.889
Saline	TS-15mg/kg	6	999	1.160	0.104	0.347
Saline	anti-tip-chimer	6	999	1.054	0.314	0.726
Saline	anti-rsPilA	6	999	0.959	0.471	0.830
TS-30mg/kg	anti-tip-chimer	6	999	1.063	0.343	0.726
TS-30mg/kg	anti-rsPilA	6	999	0.964	0.707	0.889
TS-15mg/kg	TS-30mg/kg	6	999	1.128	0.101	0.347
TS-15mg/kg	anti-tip-chimer	6	999	0.958	0.801	0.930
TS-15mg/kg	anti-rsPilA	6	999	0.855	1.000	1.000
anti-tip-chimer	anti-rsPilA	6	999	0.879	0.879	0.974

Supplementary File 1
Beta Diversity Output

Fecal Jaccard-2DPT

PERMANOVA Pairwise Comparisons (999 permutations)

Group 1	Group 2	Sample size	Permutations	pseudo-F	p-value	q-value
AC-PO7d	Ofloxacin	4	999	1.594	0.319	0.688
AC-PO7d	Saline	5	999	1.660	0.096	0.424
AC-PO7d	TS-30mg/kg	5	999	1.969	0.118	0.425
AC-PO7d	TS-15mg/kg	5	999	1.537	0.101	0.424
AC-PO7d	anti-tip-chimer	5	999	1.872	0.106	0.424
AC-PO7d	anti-rsPilA	5	999	1.639	0.084	0.424
AC-5mg/kg	AC-PO7d	4	999	2.032	0.332	0.688
AC-5mg/kg	AC-10mg/kg	4	999	1.607	0.344	0.688
AC-5mg/kg	Ofloxacin	4	999	1.009	0.685	0.778
AC-5mg/kg	Saline	5	999	0.998	0.477	0.778
AC-5mg/kg	TS-30mg/kg	5	999	1.128	0.212	0.651
AC-5mg/kg	TS-15mg/kg	5	999	1.011	0.492	0.778
AC-5mg/kg	anti-tip-chimer	5	999	1.184	0.217	0.651
AC-5mg/kg	anti-rsPilA	5	999	0.933	0.764	0.798
AC-10mg/kg	AC-PO7d	4	999	1.270	0.335	0.688
AC-10mg/kg	Ofloxacin	4	999	1.339	0.325	0.688
AC-10mg/kg	Saline	5	999	1.396	0.099	0.424
AC-10mg/kg	TS-30mg/kg	5	999	1.645	0.105	0.424
AC-10mg/kg	TS-15mg/kg	5	999	1.318	0.092	0.424
AC-10mg/kg	anti-tip-chimer	5	999	1.611	0.097	0.424
AC-10mg/kg	anti-rsPilA	5	999	1.393	0.100	0.424
Ofloxacin	Saline	5	999	0.713	0.568	0.778
Ofloxacin	TS-30mg/kg	5	999	1.070	0.315	0.688
Ofloxacin	TS-15mg/kg	5	999	0.932	0.776	0.798
Ofloxacin	anti-tip-chimer	5	999	0.996	0.606	0.778
Ofloxacin	anti-rsPilA	5	999	0.921	0.579	0.778
Saline	TS-30mg/kg	6	999	0.978	0.705	0.778
Saline	TS-15mg/kg	6	999	0.923	0.672	0.778
Saline	anti-tip-chimer	6	999	0.960	0.585	0.778
Saline	anti-rsPilA	6	999	0.958	0.683	0.778
TS-30mg/kg	anti-tip-chimer	6	999	0.996	0.508	0.778
TS-30mg/kg	anti-rsPilA	6	999	0.937	0.713	0.778
TS-15mg/kg	TS-30mg/kg	6	999	0.876	0.904	0.904
TS-15mg/kg	anti-tip-chimer	6	999	1.001	0.382	0.724
TS-15mg/kg	anti-rsPilA	6	999	0.955	0.574	0.778
anti-tip-chimer	anti-rsPilA	6	999	1.010	0.482	0.778

Supplementary File 1
Beta Diversity Output

Fecal Jaccard-5DPT

PERMANOVA Pairwise Comparisons (999 permutations)

Group 1	Group 2	Sample size	Permutations	pseudo-F	p-value	q-value
AC-PO7d	Ofloxacin	6	999	1.837	0.090	0.224
AC-PO7d	Saline	6	999	1.676	0.102	0.224
AC-PO7d	TS-30mg/kg	6	999	1.718	0.098	0.224
AC-PO7d	TS-15mg/kg	6	999	1.491	0.112	0.224
AC-PO7d	anti-tip-chimer	6	999	1.771	0.104	0.224
AC-PO7d	anti-rsPilA	5	999	1.494	0.183	0.315
AC-5mg/kg	AC-PO7d	6	999	1.395	0.111	0.224
AC-5mg/kg	AC-10mg/kg	5	999	0.751	0.910	0.936
AC-5mg/kg	Ofloxacin	6	999	1.842	0.104	0.224
AC-5mg/kg	Saline	6	999	1.641	0.103	0.224
AC-5mg/kg	TS-30mg/kg	6	999	1.850	0.082	0.224
AC-5mg/kg	TS-15mg/kg	6	999	1.425	0.089	0.224
AC-5mg/kg	anti-tip-chimer	6	999	2.004	0.112	0.224
AC-5mg/kg	anti-rsPilA	5	999	1.519	0.092	0.224
AC-10mg/kg	AC-PO7d	5	999	1.473	0.088	0.224
AC-10mg/kg	Ofloxacin	5	999	1.739	0.097	0.224
AC-10mg/kg	Saline	5	999	1.618	0.077	0.224
AC-10mg/kg	TS-30mg/kg	5	999	1.749	0.119	0.225
AC-10mg/kg	TS-15mg/kg	5	999	1.414	0.094	0.224
AC-10mg/kg	anti-tip-chimer	5	999	1.890	0.083	0.224
AC-10mg/kg	anti-rsPilA	4	999	1.464	0.332	0.478
Ofloxacin	Saline	6	999	1.122	0.193	0.316
Ofloxacin	TS-30mg/kg	6	999	1.094	0.184	0.315
Ofloxacin	TS-15mg/kg	6	999	1.047	0.401	0.555
Ofloxacin	anti-tip-chimer	6	999	1.106	0.094	0.224
Ofloxacin	anti-rsPilA	5	999	0.993	0.584	0.779
Saline	TS-30mg/kg	6	999	0.942	0.780	0.928
Saline	TS-15mg/kg	6	999	0.793	1.000	1.000
Saline	anti-tip-chimer	6	999	1.078	0.316	0.474
Saline	anti-rsPilA	5	999	0.856	0.899	0.936
TS-30mg/kg	anti-tip-chimer	6	999	1.006	0.698	0.897
TS-30mg/kg	anti-rsPilA	5	999	0.864	0.907	0.936
TS-15mg/kg	TS-30mg/kg	6	999	0.866	0.796	0.928
TS-15mg/kg	anti-tip-chimer	6	999	1.108	0.203	0.318
TS-15mg/kg	anti-rsPilA	5	999	0.839	0.890	0.936
anti-tip-chimer	anti-rsPilA	5	999	0.978	0.799	0.928

Supplementary File 1
Beta Diversity Output

Fecal Jaccard-7DPT

PERMANOVA Pairwise Comparisons (999 permutations)

Group 1	Group 2	Sample size	Permutations	pseudo-F	p-value	q-value
AC-PO7d	Ofloxacin	6	999	1.932	0.110	0.360
AC-PO7d	Saline	5	999	1.759	0.099	0.360
AC-PO7d	TS-30mg/kg	6	999	2.184	0.107	0.360
AC-PO7d	TS-15mg/kg	6	999	1.785	0.104	0.360
AC-PO7d	anti-tip-chimer	5	999	2.068	0.120	0.360
AC-PO7d	anti-rsPilA	5	999	1.931	0.099	0.360
AC-5mg/kg	AC-PO7d	6	999	1.064	0.179	0.441
AC-5mg/kg	AC-10mg/kg	4	999	1.025	0.775	0.797
AC-5mg/kg	Ofloxacin	6	999	1.551	0.086	0.360
AC-5mg/kg	Saline	5	999	1.407	0.104	0.360
AC-5mg/kg	TS-30mg/kg	6	999	1.740	0.086	0.360
AC-5mg/kg	TS-15mg/kg	6	999	1.363	0.094	0.360
AC-5mg/kg	anti-tip-chimer	5	999	1.721	0.102	0.360
AC-5mg/kg	anti-rsPilA	5	999	1.652	0.103	0.360
AC-10mg/kg	AC-PO7d	4	999	1.156	0.262	0.441
AC-10mg/kg	Ofloxacin	4	999	1.389	0.235	0.441
AC-10mg/kg	Saline	3	999	1.286	0.331	0.441
AC-10mg/kg	TS-30mg/kg	4	999	1.397	0.227	0.441
AC-10mg/kg	TS-15mg/kg	4	999	1.263	0.238	0.441
AC-10mg/kg	anti-tip-chimer	3	999	1.424	0.355	0.456
AC-10mg/kg	anti-rsPilA	3	999	1.371	0.326	0.441
Ofloxacin	Saline	5	999	1.040	0.324	0.441
Ofloxacin	TS-30mg/kg	6	999	1.095	0.196	0.441
Ofloxacin	TS-15mg/kg	6	999	1.070	0.211	0.441
Ofloxacin	anti-tip-chimer	5	999	1.022	0.305	0.441
Ofloxacin	anti-rsPilA	5	999	1.080	0.295	0.441
Saline	TS-30mg/kg	5	999	1.023	0.529	0.635
Saline	TS-15mg/kg	5	999	0.915	0.904	0.904
Saline	anti-tip-chimer	4	999	1.073	0.661	0.721
Saline	anti-rsPilA	4	999	1.057	0.653	0.721
TS-30mg/kg	anti-tip-chimer	5	999	0.918	0.708	0.750
TS-30mg/kg	anti-rsPilA	5	999	0.997	0.414	0.514
TS-15mg/kg	TS-30mg/kg	6	999	1.016	0.315	0.441
TS-15mg/kg	anti-tip-chimer	5	999	1.263	0.198	0.441
TS-15mg/kg	anti-rsPilA	5	999	1.110	0.306	0.441
anti-tip-chimer	anti-rsPilA	4	999	0.972	0.641	0.721

Supplementary File 1
Beta Diversity Output

Fecal Jaccard-9DPT

PERMANOVA Pairwise Comparisons (999 permutations)

Group 1	Group 2	Sample size	Permutations	pseudo-F	p-value	q-value
AC-PO7d	Ofloxacin	5	999	1.511	0.176	0.474
AC-PO7d	Saline	3	999	1.447	0.335	0.511
AC-PO7d	TS-30mg/kg	5	999	2.042	0.093	0.421
AC-PO7d	TS-15mg/kg	5	999	1.341	0.113	0.421
AC-PO7d	anti-tip-chimer	5	999	1.818	0.103	0.421
AC-PO7d	anti-rsPilA	4	999	1.613	0.349	0.511
AC-5mg/kg	AC-PO7d	5	999	0.964	0.395	0.527
AC-5mg/kg	AC-10mg/kg	5	999	1.173	0.200	0.474
AC-5mg/kg	Ofloxacin	6	999	1.441	0.197	0.474
AC-5mg/kg	Saline	4	999	1.273	0.234	0.474
AC-5mg/kg	TS-30mg/kg	6	999	1.835	0.101	0.421
AC-5mg/kg	TS-15mg/kg	6	999	1.331	0.095	0.421
AC-5mg/kg	anti-tip-chimer	6	999	1.756	0.117	0.421
AC-5mg/kg	anti-rsPilA	5	999	1.554	0.098	0.421
AC-10mg/kg	AC-PO7d	4	999	1.329	0.355	0.511
AC-10mg/kg	Ofloxacin	5	999	1.509	0.206	0.474
AC-10mg/kg	Saline	3	999	1.499	0.347	0.511
AC-10mg/kg	TS-30mg/kg	5	999	1.843	0.090	0.421
AC-10mg/kg	TS-15mg/kg	5	999	1.294	0.211	0.474
AC-10mg/kg	anti-tip-chimer	5	999	1.727	0.098	0.421
AC-10mg/kg	anti-rsPilA	4	999	1.533	0.336	0.511
Ofloxacin	Saline	4	999	0.927	0.527	0.612
Ofloxacin	TS-30mg/kg	6	999	1.315	0.100	0.421
Ofloxacin	TS-15mg/kg	6	999	0.982	0.380	0.526
Ofloxacin	anti-tip-chimer	6	999	1.076	0.188	0.474
Ofloxacin	anti-rsPilA	5	999	1.001	0.599	0.672
Saline	TS-30mg/kg	4	999	1.131	0.250	0.474
Saline	TS-15mg/kg	4	999	0.872	1.000	1.000
Saline	anti-tip-chimer	4	999	1.075	0.247	0.474
Saline	anti-rsPilA	3	999	0.992	0.662	0.701
TS-30mg/kg	anti-tip-chimer	6	999	1.022	0.419	0.533
TS-30mg/kg	anti-rsPilA	5	999	0.940	0.616	0.672
TS-15mg/kg	TS-30mg/kg	6	999	0.993	0.429	0.533
TS-15mg/kg	anti-tip-chimer	6	999	1.089	0.295	0.511
TS-15mg/kg	anti-rsPilA	5	999	0.926	0.499	0.599
anti-tip-chimer	anti-rsPilA	5	999	0.895	0.775	0.797

Supplementary File 1
Beta Diversity Output

Fecal Weighted UniFrac PERMDISP-Baseline

Pairwise Permdisp Results (999 permutations)

Group 1	Group 2	Sample size	Permutations	F-value	p-value	q-value
A.C (10mg.kg PO 7d)	Ofloxacin	6	999	0.758769474	0.607	0.836
A.C (10mg.kg PO 7d)	Saline	6	999	1.071540209	0.429	0.810
A.C (10mg.kg PO 7d)	T.S (15mg.kg @12h)	6	999	0.006327996	1.000	1.000
A.C (10mg.kg PO 7d)	T.S (7.5mg.kg @12h)	6	999	0.151482857	0.682	0.836
A.C (10mg.kg PO 7d)	anti-IHF	6	999	0.674164911	0.272	0.810
A.C (10mg.kg PO 7d)	anti-rsPilA	6	999	0.162877902	0.658	0.836
A.C (2.5mg.kg BID)	A.C (10mg.kg PO 7d)	5	999	1.571448627	0.409	0.810
A.C (2.5mg.kg BID)	A.C (5mg.kg BID)	4	999	inf	0.677	0.836
A.C (2.5mg.kg BID)	Ofloxacin	5	999	0.046689845	0.792	0.891
A.C (2.5mg.kg BID)	Saline	5	999	0.031834252	0.911	0.937
A.C (2.5mg.kg BID)	T.S (15mg.kg @12h)	5	999	2.548508206	0.457	0.810
A.C (2.5mg.kg BID)	T.S (7.5mg.kg @12h)	5	999	11.35147013	0.109	0.810
A.C (2.5mg.kg BID)	anti-IHF	5	999	6.396727223	0.215	0.810
A.C (2.5mg.kg BID)	anti-rsPilA	5	999	0.640046373	0.495	0.810
A.C (5mg.kg BID)	A.C (10mg.kg PO 7d)	5	999	0.88249429	0.373	0.810
A.C (5mg.kg BID)	Ofloxacin	5	999	1.24E+00	0.720	0.836
A.C (5mg.kg BID)	Saline	5	999	4.864948562	0.212	0.810
A.C (5mg.kg BID)	T.S (15mg.kg @12h)	5	999	1.898797029	0.333	0.810
A.C (5mg.kg BID)	T.S (7.5mg.kg @12h)	5	999	1.656475968	0.310	0.810
A.C (5mg.kg BID)	anti-IHF	5	999	45.10549502	0.097	0.810
A.C (5mg.kg BID)	anti-rsPilA	5	999	1.873244417	0.490	0.810
Ofloxacin	Saline	6	999	0.117112108	0.910	0.937
Ofloxacin	T.S (15mg.kg @12h)	6	999	0.743640796	0.424	0.810
Ofloxacin	T.S (7.5mg.kg @12h)	6	999	1.203482613	0.314	0.810
Ofloxacin	anti-IHF	6	999	0.351755368	0.689	0.836
Ofloxacin	anti-rsPilA	6	999	0.425958016	0.577	0.836
Saline	T.S (15mg.kg @12h)	6	999	1.230425083	0.483	0.810
Saline	T.S (7.5mg.kg @12h)	6	999	3.158853494	0.200	0.810
Saline	anti-IHF	6	999	0.350787395	0.643	0.836
Saline	anti-rsPilA	6	999	0.370909111	0.456	0.810
T.S (15mg.kg @12h)	anti-IHF	6	999	0.925310944	0.276	0.810
T.S (15mg.kg @12h)	anti-rsPilA	6	999	0.144139935	0.711	0.836
T.S (7.5mg.kg @12h)	T.S (15mg.kg @12h)	6	999	0.365669262	0.376	0.810
T.S (7.5mg.kg @12h)	anti-IHF	6	999	5.837283529	0.041	0.810
T.S (7.5mg.kg @12h)	anti-rsPilA	6	999	0.810350686	0.312	0.810
anti-IHF	anti-rsPilA	6	999	0.067526766	0.897	0.937

Omnibus PERMDISP Test

p-value 0.462

Supplementary File 1
Beta Diversity Output

Fecal Weighted UniFrac PERMDISP-2DPT

Pairwise Permdisp Results (999 permutations)

Group 1	Group 2	Sample size	Permutations	F-value	p-value	q-value
A.C (10mg.kg PO 7d)	Ofloxacin	4	999	inf	1.000	1.000
A.C (10mg.kg PO 7d)	Saline	5	999	0.507159051	0.188	0.631
A.C (10mg.kg PO 7d)	T.S (15mg.kg @12h)	5	999	3.006889816	0.121	0.631
A.C (10mg.kg PO 7d)	T.S (7.5mg.kg @12h)	5	999	2.335205471	0.111	0.631
A.C (10mg.kg PO 7d)	anti-IHF	5	999	1.891970047	0.094	0.631
A.C (10mg.kg PO 7d)	anti-rsPilA	5	999	1.501940928	0.093	0.631
A.C (2.5mg.kg BID)	A.C (10mg.kg PO 7d)	4	999	inf	1.000	1.000
A.C (2.5mg.kg BID)	A.C (5mg.kg BID)	4	999	inf	1.000	1.000
A.C (2.5mg.kg BID)	Ofloxacin	4	999	inf	1.000	1.000
A.C (2.5mg.kg BID)	Saline	5	999	0.998424618	0.198	0.631
A.C (2.5mg.kg BID)	T.S (15mg.kg @12h)	5	999	4.161790334	0.306	0.734
A.C (2.5mg.kg BID)	T.S (7.5mg.kg @12h)	5	999	3.96793933	0.194	0.631
A.C (2.5mg.kg BID)	anti-IHF	5	999	2.81369581	0.203	0.631
A.C (2.5mg.kg BID)	anti-rsPilA	5	999	2.192583734	0.544	1.000
A.C (5mg.kg BID)	A.C (10mg.kg PO 7d)	4	999	inf	1.000	1.000
A.C (5mg.kg BID)	Ofloxacin	4	999	1.55E+16	0.828	1.000
A.C (5mg.kg BID)	Saline	5	999	0.112425633	0.681	1.000
A.C (5mg.kg BID)	T.S (15mg.kg @12h)	5	999	1.775058785	0.209	0.631
A.C (5mg.kg BID)	T.S (7.5mg.kg @12h)	5	999	0.844972864	0.105	0.631
A.C (5mg.kg BID)	anti-IHF	5	999	0.958700608	0.181	0.631
A.C (5mg.kg BID)	anti-rsPilA	5	999	0.793010659	0.228	0.631
Ofloxacin	Saline	5	999	0.562837662	0.701	1.000
Ofloxacin	T.S (15mg.kg @12h)	5	999	0.030674151	0.897	1.000
Ofloxacin	T.S (7.5mg.kg @12h)	5	999	0.696819808	0.595	1.000
Ofloxacin	anti-IHF	5	999	0.026430769	0.903	1.000
Ofloxacin	anti-rsPilA	5	999	0.005555074	0.903	1.000
Saline	T.S (15mg.kg @12h)	6	999	0.741444368	0.223	0.631
Saline	T.S (7.5mg.kg @12h)	6	999	0.065760728	0.727	1.000
Saline	anti-IHF	6	999	0.310573073	0.509	1.000
Saline	anti-rsPilA	6	999	0.326724511	0.531	1.000
T.S (15mg.kg @12h)	anti-IHF	6	999	0.094991433	0.722	1.000
T.S (15mg.kg @12h)	anti-rsPilA	6	999	0.047834002	0.700	1.000
T.S (7.5mg.kg @12h)	T.S (15mg.kg @12h)	6	999	0.611827623	0.259	0.666
T.S (7.5mg.kg @12h)	anti-IHF	6	999	0.169744616	0.493	1.000
T.S (7.5mg.kg @12h)	anti-rsPilA	6	999	0.189391403	0.672	1.000
anti-IHF	anti-rsPilA	6	999	0.003844466	0.912	1.000

Omnibus PERMDISP Test

p-value 0.505

Supplementary File 1
Beta Diversity Output

Fecal Weighted UniFrac PERMDISP-5DPT

Pairwise Permdisp Results (999 permutations)

Group 1	Group 2	Sample size	Permutations	F-value	p-value	q-value
A.C (10mg.kg PO 7d)	Ofloxacin	6	999	0.002209627	1.000	1.000
A.C (10mg.kg PO 7d)	Saline	6	999	2.023509174	0.040	0.396
A.C (10mg.kg PO 7d)	T.S (15mg.kg @12h)	6	999	1.038429693	0.295	0.542
A.C (10mg.kg PO 7d)	T.S (7.5mg.kg @12h)	6	999	0.003566655	0.905	0.987
A.C (10mg.kg PO 7d)	anti-IHF	6	999	0.870363504	0.499	0.781
A.C (10mg.kg PO 7d)	anti-rsPilA	5	999	0.173254346	0.784	0.941
A.C (2.5mg.kg BID)	A.C (10mg.kg PO 7d)	6	999	0.007840996	0.626	0.901
A.C (2.5mg.kg BID)	A.C (5mg.kg BID)	5	999	1.023424678	0.104	0.396
A.C (2.5mg.kg BID)	Ofloxacin	6	999	0.012188812	0.766	0.941
A.C (2.5mg.kg BID)	Saline	6	999	0.540094699	0.041	0.396
A.C (2.5mg.kg BID)	T.S (15mg.kg @12h)	6	999	0.718335513	0.254	0.542
A.C (2.5mg.kg BID)	T.S (7.5mg.kg @12h)	6	999	0.013271205	0.903	0.987
A.C (2.5mg.kg BID)	anti-IHF	6	999	0.430831462	0.374	0.641
A.C (2.5mg.kg BID)	anti-rsPilA	5	999	0.047053419	0.689	0.919
A.C (5mg.kg BID)	A.C (10mg.kg PO 7d)	5	999	7.334921751	0.124	0.406
A.C (5mg.kg BID)	Ofloxacin	5	999	1.55E+01	0.110	0.396
A.C (5mg.kg BID)	Saline	5	999	1.570719485	0.093	0.396
A.C (5mg.kg BID)	T.S (15mg.kg @12h)	5	999	1.853474325	0.099	0.396
A.C (5mg.kg BID)	T.S (7.5mg.kg @12h)	5	999	1.768918879	0.091	0.396
A.C (5mg.kg BID)	anti-IHF	5	999	2.640757399	0.093	0.396
A.C (5mg.kg BID)	anti-rsPilA	4	999	inf	1.000	1.000
Ofloxacin	Saline	6	999	2.666408093	0.085	0.396
Ofloxacin	T.S (15mg.kg @12h)	6	999	1.085521165	0.199	0.518
Ofloxacin	T.S (7.5mg.kg @12h)	6	999	0.001294501	1.000	1.000
Ofloxacin	anti-IHF	6	999	0.950644072	0.297	0.542
Ofloxacin	anti-rsPilA	5	999	0.301600607	0.594	0.891
Saline	T.S (15mg.kg @12h)	6	999	2.005067092	0.178	0.518
Saline	T.S (7.5mg.kg @12h)	6	999	0.706054812	0.216	0.518
Saline	anti-IHF	6	999	2.363958283	0.103	0.396
Saline	anti-rsPilA	5	999	1.350495129	0.684	0.919
T.S (15mg.kg @12h)	anti-IHF	6	999	0.120285161	0.752	0.941
T.S (15mg.kg @12h)	anti-rsPilA	5	999	0.794197547	0.301	0.542
T.S (7.5mg.kg @12h)	T.S (15mg.kg @12h)	6	999	0.980449104	0.402	0.658
T.S (7.5mg.kg @12h)	anti-IHF	6	999	0.749126423	0.212	0.518
T.S (7.5mg.kg @12h)	anti-rsPilA	5	999	0.025746008	0.902	0.987
anti-IHF	anti-rsPilA	5	999	0.795418126	0.301	0.542

**Omnibus PERMDISP Test**	
**p-value**	0.183

Supplementary File 1
Beta Diversity Output

Fecal Weighted UniFrac PERMDISP-7DPT

Pairwise Permdisp Results (999 permutations)

Group 1	Group 2	Sample size	Permutations	F-value	p-value	q-value
A.C (10mg.kg PO 7d)	Ofloxacin	6	999	2.293636209	0.056	1.000
A.C (10mg.kg PO 7d)	Saline	5	999	12.1994291	0.224	1.000
A.C (10mg.kg PO 7d)	T.S (15mg.kg @12h)	6	999	0.000101625	1.000	1.000
A.C (10mg.kg PO 7d)	T.S (7.5mg.kg @12h)	6	999	3.657412731	0.123	1.000
A.C (10mg.kg PO 7d)	anti-IHF	5	999	0.810816131	0.706	1.000
A.C (10mg.kg PO 7d)	anti-rsPilA	5	999	8.619022496	0.217	1.000
A.C (2.5mg.kg BID)	A.C (10mg.kg PO 7d)	6	999	1.97003925	0.225	1.000
A.C (2.5mg.kg BID)	A.C (5mg.kg BID)	4	999	3.226530795	0.736	1.000
A.C (2.5mg.kg BID)	Ofloxacin	6	999	0.005432835	0.917	1.000
A.C (2.5mg.kg BID)	Saline	5	999	0.678892769	0.496	1.000
A.C (2.5mg.kg BID)	T.S (15mg.kg @12h)	6	999	0.372428695	0.588	1.000
A.C (2.5mg.kg BID)	T.S (7.5mg.kg @12h)	6	999	0.002632871	0.847	1.000
A.C (2.5mg.kg BID)	anti-IHF	5	999	0.530079095	0.712	1.000
A.C (2.5mg.kg BID)	anti-rsPilA	5	999	0.240594022	0.469	1.000
A.C (5mg.kg BID)	A.C (10mg.kg PO 7d)	4	999	19.06617128	0.261	1.000
A.C (5mg.kg BID)	Ofloxacin	4	999	5.03E+00	0.538	1.000
A.C (5mg.kg BID)	Saline	3	999	inf	1.000	1.000
A.C (5mg.kg BID)	T.S (15mg.kg @12h)	4	999	1.075521648	1.000	1.000
A.C (5mg.kg BID)	T.S (7.5mg.kg @12h)	4	999	7.263394224	0.256	1.000
A.C (5mg.kg BID)	anti-IHF	3	999	inf	1.000	1.000
A.C (5mg.kg BID)	anti-rsPilA	3	999	inf	1.000	1.000
Ofloxacin	Saline	5	999	1.192217988	0.411	1.000
Ofloxacin	T.S (15mg.kg @12h)	6	999	0.345995293	0.548	1.000
Ofloxacin	T.S (7.5mg.kg @12h)	6	999	0.022234019	0.757	1.000
Ofloxacin	anti-IHF	5	999	0.63282286	0.516	1.000
Ofloxacin	anti-rsPilA	5	999	0.47112791	0.629	1.000
Saline	T.S (15mg.kg @12h)	5	999	0.694622859	0.703	1.000
Saline	T.S (7.5mg.kg @12h)	5	999	1.40466357	0.193	1.000
Saline	anti-IHF	4	999	inf	1.000	1.000
Saline	anti-rsPilA	4	999	1.32717E+16	1.000	1.000
T.S (15mg.kg @12h)	anti-IHF	5	999	0.048761611	0.772	1.000
T.S (15mg.kg @12h)	anti-rsPilA	5	999	0.492552467	0.702	1.000
T.S (7.5mg.kg @12h)	T.S (15mg.kg @12h)	6	999	0.433806318	0.598	1.000
T.S (7.5mg.kg @12h)	anti-IHF	5	999	1.401576799	0.691	1.000
T.S (7.5mg.kg @12h)	anti-rsPilA	5	999	0.457243987	0.690	1.000
anti-IHF	anti-rsPilA	4	999	inf	1.000	1.000

**Omnibus PERMDISP Test**	
**p-value**	0.603

Supplementary File 1
Beta Diversity Output

Fecal Weighted UniFrac PERMDISP-9DPT

Pairwise Permdisp Results (999 permutations)

Group 1	Group 2	Sample size	Permutations	F-value	p-value	q-value
A.C (10mg.kg PO 7d)	Ofloxacin	5	999	0.01039443	0.897	1.000
A.C (10mg.kg PO 7d)	Saline	3	999	inf	1.000	1.000
A.C (10mg.kg PO 7d)	T.S (15mg.kg @12h)	5	999	1.623793963	0.305	1.000
A.C (10mg.kg PO 7d)	T.S (7.5mg.kg @12h)	5	999	0.630912477	0.585	1.000
A.C (10mg.kg PO 7d)	anti-IHF	5	999	1.61445E-06	1.000	1.000
A.C (10mg.kg PO 7d)	anti-rsPilA	4	999	inf	1.000	1.000
A.C (2.5mg.kg BID)	A.C (10mg.kg PO 7d)	5	999	0.866386239	0.488	1.000
A.C (2.5mg.kg BID)	A.C (5mg.kg BID)	5	999	0.159635085	0.628	1.000
A.C (2.5mg.kg BID)	Ofloxacin	6	999	0.301042166	0.293	1.000
A.C (2.5mg.kg BID)	Saline	4	999	5.779361846	1.000	1.000
A.C (2.5mg.kg BID)	T.S (15mg.kg @12h)	6	999	0.469217989	0.492	1.000
A.C (2.5mg.kg BID)	T.S (7.5mg.kg @12h)	6	999	0.03492604	0.809	1.000
A.C (2.5mg.kg BID)	anti-IHF	6	999	1.006404389	0.288	1.000
A.C (2.5mg.kg BID)	anti-rsPilA	5	999	4.222981183	0.188	1.000
A.C (5mg.kg BID)	A.C (10mg.kg PO 7d)	4	999	inf	0.851	1.000
A.C (5mg.kg BID)	Ofloxacin	5	999	5.91E-02	0.780	1.000
A.C (5mg.kg BID)	Saline	3	999	inf	1.000	1.000
A.C (5mg.kg BID)	T.S (15mg.kg @12h)	5	999	0.844689528	0.389	1.000
A.C (5mg.kg BID)	T.S (7.5mg.kg @12h)	5	999	0.196600672	0.699	1.000
A.C (5mg.kg BID)	anti-IHF	5	999	0.639372418	0.569	1.000
A.C (5mg.kg BID)	anti-rsPilA	4	999	inf	1.000	1.000
Ofloxacin	Saline	4	999	1.55692685	1.000	1.000
Ofloxacin	T.S (15mg.kg @12h)	6	999	1.106655527	0.507	1.000
Ofloxacin	T.S (7.5mg.kg @12h)	6	999	0.39088379	0.618	1.000
Ofloxacin	anti-IHF	6	999	0.014454294	0.858	1.000
Ofloxacin	anti-rsPilA	5	999	0.703643462	0.781	1.000
Saline	T.S (15mg.kg @12h)	4	999	4.157749566	0.515	1.000
Saline	T.S (7.5mg.kg @12h)	4	999	2.939419035	0.743	1.000
Saline	anti-IHF	4	999	7.527483015	1.000	1.000
Saline	anti-rsPilA	3	999	inf	1.000	1.000
T.S (15mg.kg @12h)	anti-IHF	6	999	2.259456636	0.428	1.000
T.S (15mg.kg @12h)	anti-rsPilA	5	999	4.131888157	0.288	1.000
T.S (7.5mg.kg @12h)	T.S (15mg.kg @12h)	6	999	0.182014124	0.536	1.000
T.S (7.5mg.kg @12h)	anti-IHF	6	999	0.880931609	0.155	1.000
T.S (7.5mg.kg @12h)	anti-rsPilA	5	999	2.382464918	0.110	1.000
anti-IHF	anti-rsPilA	5	999	2.92837205	0.108	1.000

Omnibus PERMDISP Test
p-value 0.32

Supplementary File 1
Beta Diversity Output

Fecal Unweighted UniFrac PERMDISP-Baseline

Pairwise Permdisp Results (999 permutations)

Group 1	Group 2	Sample size	Permutations	F-value	p-value	q-value
A.C (10mg.kg PO 7d)	Ofloxacin	6	999	4.687879714	0.100	0.420
A.C (10mg.kg PO 7d)	Saline	6	999	5.649437818	0.060	0.420
A.C (10mg.kg PO 7d)	T.S (15mg.kg @12h)	6	999	2.665698051	0.054	0.420
A.C (10mg.kg PO 7d)	T.S (7.5mg.kg @12h)	6	999	7.677486309	0.046	0.420
A.C (10mg.kg PO 7d)	anti-IHF	6	999	12.03005503	0.044	0.420
A.C (10mg.kg PO 7d)	anti-rsPilA	6	999	18.42639451	0.099	0.420
A.C (2.5mg.kg BID)	A.C (10mg.kg PO 7d)	5	999	0.692703894	0.573	0.838
A.C (2.5mg.kg BID)	A.C (5mg.kg BID)	4	999	inf	0.673	0.838
A.C (2.5mg.kg BID)	Ofloxacin	5	999	2.121742493	0.693	0.838
A.C (2.5mg.kg BID)	Saline	5	999	2.696984515	0.573	0.838
A.C (2.5mg.kg BID)	T.S (15mg.kg @12h)	5	999	0.940809646	0.895	0.959
A.C (2.5mg.kg BID)	T.S (7.5mg.kg @12h)	5	999	4.289494786	0.373	0.822
A.C (2.5mg.kg BID)	anti-IHF	5	999	10.60777286	0.411	0.822
A.C (2.5mg.kg BID)	anti-rsPilA	5	999	12.09569961	0.203	0.609
A.C (5mg.kg BID)	A.C (10mg.kg PO 7d)	5	999	2.106899867	0.402	0.822
A.C (5mg.kg BID)	Ofloxacin	5	999	1.26E+00	0.694	0.838
A.C (5mg.kg BID)	Saline	5	999	1.759018132	0.612	0.838
A.C (5mg.kg BID)	T.S (15mg.kg @12h)	5	999	0.334213202	0.799	0.928
A.C (5mg.kg BID)	T.S (7.5mg.kg @12h)	5	999	2.528855742	0.297	0.764
A.C (5mg.kg BID)	anti-IHF	5	999	6.173183186	0.099	0.420
A.C (5mg.kg BID)	anti-rsPilA	5	999	9.26233651	0.292	0.764
Ofloxacin	Saline	6	999	0.060994585	0.648	0.838
Ofloxacin	T.S (15mg.kg @12h)	6	999	0.383756932	0.592	0.838
Ofloxacin	T.S (7.5mg.kg @12h)	6	999	0.000404553	0.958	0.985
Ofloxacin	anti-IHF	6	999	0.00337947	0.906	0.959
Ofloxacin	anti-rsPilA	6	999	1.548013313	0.199	0.609
Saline	T.S (15mg.kg @12h)	6	999	0.747149838	0.378	0.822
Saline	T.S (7.5mg.kg @12h)	6	999	0.092159321	0.857	0.959
Saline	anti-IHF	6	999	0.137074868	0.698	0.838
Saline	anti-rsPilA	6	999	0.857690981	0.501	0.838
T.S (15mg.kg @12h)	anti-IHF	6	999	0.617216794	0.605	0.838
T.S (15mg.kg @12h)	anti-rsPilA	6	999	4.314556537	0.105	0.420
T.S (7.5mg.kg @12h)	T.S (15mg.kg @12h)	6	999	0.515927089	0.480	0.838
T.S (7.5mg.kg @12h)	anti-IHF	6	999	0.002256867	1.000	1.000
T.S (7.5mg.kg @12h)	anti-rsPilA	6	999	2.368429917	0.061	0.420
anti-IHF	anti-rsPilA	6	999	3.429106784	0.183	0.609

Omnibus PERMDISP Test

p-value 0.036

Supplementary File 1
Beta Diversity Output

Fecal Unweighted UniFrac PERMDISP-2DPT

Pairwise Permdisp Results (999 permutations)

Group 1	Group 2	Sample size	Permutations	F-value	p-value	q-value
A.C (10mg.kg PO 7d)	Ofloxacin	4	999	inf	1.000	1.000
A.C (10mg.kg PO 7d)	Saline	5	999	4.171474492	0.101	0.909
A.C (10mg.kg PO 7d)	T.S (15mg.kg @12h)	5	999	3.994844474	0.090	0.909
A.C (10mg.kg PO 7d)	T.S (7.5mg.kg @12h)	5	999	2.823277521	0.294	0.916
A.C (10mg.kg PO 7d)	anti-IHF	5	999	0.719509771	0.314	0.916
A.C (10mg.kg PO 7d)	anti-rsPilA	5	999	2.213397593	0.187	0.916
A.C (2.5mg.kg BID)	A.C (10mg.kg PO 7d)	4	999	inf	1.000	1.000
A.C (2.5mg.kg BID)	A.C (5mg.kg BID)	4	999	-1.23701E+16	1.000	1.000
A.C (2.5mg.kg BID)	Ofloxacin	4	999	inf	1.000	1.000
A.C (2.5mg.kg BID)	Saline	5	999	7.135124169	0.208	0.916
A.C (2.5mg.kg BID)	T.S (15mg.kg @12h)	5	999	17.51099601	0.214	0.916
A.C (2.5mg.kg BID)	T.S (7.5mg.kg @12h)	5	999	4.417260894	0.090	0.909
A.C (2.5mg.kg BID)	anti-IHF	5	999	1.652878659	0.400	0.916
A.C (2.5mg.kg BID)	anti-rsPilA	5	999	3.720237903	0.217	0.916
A.C (5mg.kg BID)	A.C (10mg.kg PO 7d)	4	999	inf	1.000	1.000
A.C (5mg.kg BID)	Ofloxacin	4	999	inf	1.000	1.000
A.C (5mg.kg BID)	Saline	5	999	3.152625971	0.384	0.916
A.C (5mg.kg BID)	T.S (15mg.kg @12h)	5	999	1.146956278	0.404	0.916
A.C (5mg.kg BID)	T.S (7.5mg.kg @12h)	5	999	2.254143106	0.407	0.916
A.C (5mg.kg BID)	anti-IHF	5	999	0.439031291	0.688	0.940
A.C (5mg.kg BID)	anti-rsPilA	5	999	1.691456344	0.582	0.940
Ofloxacin	Saline	5	999	1.120865292	0.512	0.940
Ofloxacin	T.S (15mg.kg @12h)	5	999	2.019977116	0.577	0.940
Ofloxacin	T.S (7.5mg.kg @12h)	5	999	1.042139408	0.705	0.940
Ofloxacin	anti-IHF	5	999	0.026861836	1.000	1.000
Ofloxacin	anti-rsPilA	5	999	0.636346006	0.694	0.940
Saline	T.S (15mg.kg @12h)	6	999	3.31506579	0.333	0.916
Saline	T.S (7.5mg.kg @12h)	6	999	0.111345019	0.901	1.000
Saline	anti-IHF	6	999	0.368266934	0.473	0.940
Saline	anti-rsPilA	6	999	0.003387285	1.000	1.000
T.S (15mg.kg @12h)	anti-IHF	6	999	0.322048987	0.563	0.940
T.S (15mg.kg @12h)	anti-rsPilA	6	999	1.88347051	0.304	0.916
T.S (7.5mg.kg @12h)	T.S (15mg.kg @12h)	6	999	2.694387285	0.100	0.909
T.S (7.5mg.kg @12h)	anti-IHF	6	999	0.643558859	0.491	0.940
T.S (7.5mg.kg @12h)	anti-rsPilA	6	999	0.058232267	0.704	0.940
anti-IHF	anti-rsPilA	6	999	0.330570186	0.644	0.940

Omnibus PERMDISP Test

p-value 0.248

Supplementary File 1
Beta Diversity Output

Fecal Unweighted UniFrac PERMDISP-5DPT

Pairwise Permdisp Results (999 permutations)

Group 1	Group 2	Sample size	Permutations	F-value	p-value	q-value
A.C (10mg.kg PO 7d)	Ofloxacin	6	999	0.761306447	0.491	0.981
A.C (10mg.kg PO 7d)	Saline	6	999	0.175092364	0.894	0.981
A.C (10mg.kg PO 7d)	T.S (15mg.kg @12h)	6	999	0.347183264	0.716	0.981
A.C (10mg.kg PO 7d)	T.S (7.5mg.kg @12h)	6	999	0.055981893	0.789	0.981
A.C (10mg.kg PO 7d)	anti-IHF	6	999	0.597959908	0.373	0.981
A.C (10mg.kg PO 7d)	anti-rsPilA	5	999	0.579621124	0.603	0.981
A.C (2.5mg.kg BID)	A.C (10mg.kg PO 7d)	6	999	0.024146887	0.699	0.981
A.C (2.5mg.kg BID)	A.C (5mg.kg BID)	5	999	1.202104727	0.506	0.981
A.C (2.5mg.kg BID)	Ofloxacin	6	999	0.735795055	0.276	0.981
A.C (2.5mg.kg BID)	Saline	6	999	0.263512035	0.523	0.981
A.C (2.5mg.kg BID)	T.S (15mg.kg @12h)	6	999	0.418243831	0.302	0.981
A.C (2.5mg.kg BID)	T.S (7.5mg.kg @12h)	6	999	0.127668024	0.714	0.981
A.C (2.5mg.kg BID)	anti-IHF	6	999	0.618780586	0.101	0.778
A.C (2.5mg.kg BID)	anti-rsPilA	5	999	0.529858455	0.534	0.981
A.C (5mg.kg BID)	A.C (10mg.kg PO 7d)	5	999	1.556574964	0.200	0.981
A.C (5mg.kg BID)	Ofloxacin	5	999	1.60E+01	0.101	0.778
A.C (5mg.kg BID)	Saline	5	999	5.42093341	0.096	0.778
A.C (5mg.kg BID)	T.S (15mg.kg @12h)	5	999	3.630173895	0.108	0.778
A.C (5mg.kg BID)	T.S (7.5mg.kg @12h)	5	999	0.964205464	0.399	0.981
A.C (5mg.kg BID)	anti-IHF	5	999	4.31934073	0.102	0.778
A.C (5mg.kg BID)	anti-rsPilA	4	999	inf	1.000	1.000
Ofloxacin	Saline	6	999	1.104476707	0.266	0.981
Ofloxacin	T.S (15mg.kg @12h)	6	999	0.344074187	0.316	0.981
Ofloxacin	T.S (7.5mg.kg @12h)	6	999	0.28853896	0.763	0.981
Ofloxacin	anti-IHF	6	999	0.049928289	0.552	0.981
Ofloxacin	anti-rsPilA	5	999	0.31091103	1.000	1.000
Saline	T.S (15mg.kg @12h)	6	999	0.111858345	0.823	0.981
Saline	T.S (7.5mg.kg @12h)	6	999	0.011002873	0.896	0.981
Saline	anti-IHF	6	999	0.537690248	0.386	0.981
Saline	anti-rsPilA	5	999	1.145789789	0.535	0.981
T.S (15mg.kg @12h)	anti-IHF	6	999	0.121324798	0.386	0.981
T.S (15mg.kg @12h)	anti-rsPilA	5	999	0.463225413	0.592	0.981
T.S (7.5mg.kg @12h)	T.S (15mg.kg @12h)	6	999	0.07613626	1.000	1.000
T.S (7.5mg.kg @12h)	anti-IHF	6	999	0.201944603	0.893	0.981
T.S (7.5mg.kg @12h)	anti-rsPilA	5	999	0.248469705	0.704	0.981
anti-IHF	anti-rsPilA	5	999	0.206462185	0.899	0.981

Omnibus PERMDISP Test

p-value 0.666

Supplementary File 1
Beta Diversity Output

Fecal Unweighted UniFrac PERMDISP-7DPT

Pairwise Permdisp Results (999 permutations)

Group 1	Group 2	Sample size	Permutations	F-value	p-value	q-value
A.C (10mg.kg PO 7d)	Ofloxacin	6	999	0.000393495	1.000	1.000
A.C (10mg.kg PO 7d)	Saline	5	999	0.023651455	0.921	1.000
A.C (10mg.kg PO 7d)	T.S (15mg.kg @12h)	6	999	0.188869142	0.295	1.000
A.C (10mg.kg PO 7d)	T.S (7.5mg.kg @12h)	6	999	0.014439765	0.768	1.000
A.C (10mg.kg PO 7d)	anti-IHF	5	999	1.99369786	0.093	1.000
A.C (10mg.kg PO 7d)	anti-rsPilA	5	999	0.595377194	0.528	1.000
A.C (2.5mg.kg BID)	A.C (10mg.kg PO 7d)	6	999	0.047687798	0.563	1.000
A.C (2.5mg.kg BID)	A.C (5mg.kg BID)	4	999	14.75105555	1.000	1.000
A.C (2.5mg.kg BID)	Ofloxacin	6	999	0.067032076	0.351	1.000
A.C (2.5mg.kg BID)	Saline	5	999	0.117009831	0.894	1.000
A.C (2.5mg.kg BID)	T.S (15mg.kg @12h)	6	999	0.394960822	0.190	1.000
A.C (2.5mg.kg BID)	T.S (7.5mg.kg @12h)	6	999	0.0954339	0.460	1.000
A.C (2.5mg.kg BID)	anti-IHF	5	999	1.850439067	0.196	1.000
A.C (2.5mg.kg BID)	anti-rsPilA	5	999	0.709063153	0.421	1.000
A.C (5mg.kg BID)	A.C (10mg.kg PO 7d)	4	999	20.90520577	0.771	1.000
A.C (5mg.kg BID)	Ofloxacin	4	999	3.89E+01	0.242	1.000
A.C (5mg.kg BID)	Saline	3	999	inf	0.670	1.000
A.C (5mg.kg BID)	T.S (15mg.kg @12h)	4	999	140.4585513	0.256	1.000
A.C (5mg.kg BID)	T.S (7.5mg.kg @12h)	4	999	13.4227416	0.742	1.000
A.C (5mg.kg BID)	anti-IHF	3	999	inf	1.000	1.000
A.C (5mg.kg BID)	anti-rsPilA	3	999	inf	1.000	1.000
Ofloxacin	Saline	5	999	0.033988287	1.000	1.000
Ofloxacin	T.S (15mg.kg @12h)	6	999	0.287333576	0.576	1.000
Ofloxacin	T.S (7.5mg.kg @12h)	6	999	0.013455303	0.909	1.000
Ofloxacin	anti-IHF	5	999	3.631566656	0.414	1.000
Ofloxacin	anti-rsPilA	5	999	1.060037045	0.593	1.000
Saline	T.S (15mg.kg @12h)	5	999	0.312386292	0.886	1.000
Saline	T.S (7.5mg.kg @12h)	5	999	3.10532E-05	1.000	1.000
Saline	anti-IHF	4	999	-1.11833E+16	1.000	1.000
Saline	anti-rsPilA	4	999	inf	1.000	1.000
T.S (15mg.kg @12h)	anti-IHF	5	999	8.272957881	0.388	1.000
T.S (15mg.kg @12h)	anti-rsPilA	5	999	1.272118554	0.695	1.000
T.S (7.5mg.kg @12h)	T.S (15mg.kg @12h)	6	999	0.045814438	0.797	1.000
T.S (7.5mg.kg @12h)	anti-IHF	5	999	1.071478317	0.394	1.000
T.S (7.5mg.kg @12h)	anti-rsPilA	5	999	0.26124082	0.793	1.000
anti-IHF	anti-rsPilA	4	999	inf	1.000	1.000

Omnibus PERMDISP Test

p-value 0.131

Supplementary File 1
Beta Diversity Output

Fecal Unweighted UniFrac PERMDISP-9DPT

Pairwise Permdisp Results (999 permutations)

Group 1	Group 2	Sample size	Permutations	F-value	p-value	q-value
A.C (10mg.kg PO 7d)	Ofloxacin	5	999	0.230366872	0.678	1.000
A.C (10mg.kg PO 7d)	Saline	3	999	inf	1.000	1.000
A.C (10mg.kg PO 7d)	T.S (15mg.kg @12h)	5	999	0.457064087	0.382	1.000
A.C (10mg.kg PO 7d)	T.S (7.5mg.kg @12h)	5	999	0.291791206	0.806	1.000
A.C (10mg.kg PO 7d)	anti-IHF	5	999	3.82618769	0.378	1.000
A.C (10mg.kg PO 7d)	anti-rsPilA	4	999	inf	1.000	1.000
A.C (2.5mg.kg BID)	A.C (10mg.kg PO 7d)	5	999	0.194491072	0.814	1.000
A.C (2.5mg.kg BID)	A.C (5mg.kg BID)	5	999	1.337917299	0.501	1.000
A.C (2.5mg.kg BID)	Ofloxacin	6	999	0.001126588	1.000	1.000
A.C (2.5mg.kg BID)	Saline	4	999	9.139138654	0.750	1.000
A.C (2.5mg.kg BID)	T.S (15mg.kg @12h)	6	999	0.752464066	0.205	1.000
A.C (2.5mg.kg BID)	T.S (7.5mg.kg @12h)	6	999	0.004605656	0.939	1.000
A.C (2.5mg.kg BID)	anti-IHF	6	999	1.052642795	0.096	1.000
A.C (2.5mg.kg BID)	anti-rsPilA	5	999	0.647367237	0.721	1.000
A.C (5mg.kg BID)	A.C (10mg.kg PO 7d)	4	999	inf	0.655	1.000
A.C (5mg.kg BID)	Ofloxacin	5	999	1.44E+00	0.182	1.000
A.C (5mg.kg BID)	Saline	3	999	inf	1.000	1.000
A.C (5mg.kg BID)	T.S (15mg.kg @12h)	5	999	0.982544738	0.314	1.000
A.C (5mg.kg BID)	T.S (7.5mg.kg @12h)	5	999	1.671757453	0.308	1.000
A.C (5mg.kg BID)	anti-IHF	5	999	3.429474486	0.091	1.000
A.C (5mg.kg BID)	anti-rsPilA	4	999	inf	1.000	1.000
Ofloxacin	Saline	4	999	9.371400979	1.000	1.000
Ofloxacin	T.S (15mg.kg @12h)	6	999	0.836527093	0.601	1.000
Ofloxacin	T.S (7.5mg.kg @12h)	6	999	0.001130832	1.000	1.000
Ofloxacin	anti-IHF	6	999	1.160902612	0.408	1.000
Ofloxacin	anti-rsPilA	5	999	0.714490249	0.679	1.000
Saline	T.S (15mg.kg @12h)	4	999	35.32293183	0.483	1.000
Saline	T.S (7.5mg.kg @12h)	4	999	10.42727247	1.000	1.000
Saline	anti-IHF	4	999	177.1027631	0.263	1.000
Saline	anti-rsPilA	3	999	inf	1.000	1.000
T.S (15mg.kg @12h)	anti-IHF	6	999	0.045510572	0.810	1.000
T.S (15mg.kg @12h)	anti-rsPilA	5	999	0.029229244	1.000	1.000
T.S (7.5mg.kg @12h)	T.S (15mg.kg @12h)	6	999	0.989512964	0.306	1.000
T.S (7.5mg.kg @12h)	anti-IHF	6	999	1.377956898	0.102	1.000
T.S (7.5mg.kg @12h)	anti-rsPilA	5	999	0.851213723	0.601	1.000
anti-IHF	anti-rsPilA	5	999	0.000463487	1.000	1.000

Omnibus PERMDISP Test

p-value 0.556

Supplementary File 1
Beta Diversity Output

Fecal Bray-Curtis PERMDISP-Baseline

Pairwise Permdisp Results (999 permutations)

Group 1	Group 2	Sample size	Permutations	F-value	p-value	q-value
A.C (10mg.kg PO 7d)	Ofloxacin	6	999	0.17814082	0.662	0.804
A.C (10mg.kg PO 7d)	Saline	6	999	2.049267268	0.281	0.597
A.C (10mg.kg PO 7d)	T.S (15mg.kg @12h)	6	999	0.287127755	0.663	0.804
A.C (10mg.kg PO 7d)	T.S (7.5mg.kg @12h)	6	999	0.012835064	1.000	1.000
A.C (10mg.kg PO 7d)	anti-IHF	6	999	1.470479936	0.187	0.594
A.C (10mg.kg PO 7d)	anti-rsPilA	6	999	1.033937805	0.461	0.722
A.C (2.5mg.kg BID)	A.C (10mg.kg PO 7d)	5	999	0.043214054	1.000	1.000
A.C (2.5mg.kg BID)	A.C (5mg.kg BID)	4	999	inf	1.000	1.000
A.C (2.5mg.kg BID)	Ofloxacin	5	999	5.09582741	0.198	0.594
A.C (2.5mg.kg BID)	Saline	5	999	6.614098908	0.216	0.597
A.C (2.5mg.kg BID)	T.S (15mg.kg @12h)	5	999	1.373070499	0.682	0.804
A.C (2.5mg.kg BID)	T.S (7.5mg.kg @12h)	5	999	0.358657558	0.692	0.804
A.C (2.5mg.kg BID)	anti-IHF	5	999	4.029235132	0.315	0.597
A.C (2.5mg.kg BID)	anti-rsPilA	5	999	6.705282399	0.095	0.495
A.C (5mg.kg BID)	A.C (10mg.kg PO 7d)	5	999	0.390298877	0.804	0.881
A.C (5mg.kg BID)	Ofloxacin	5	999	1.59E+01	0.102	0.495
A.C (5mg.kg BID)	Saline	5	999	10.83512791	0.110	0.495
A.C (5mg.kg BID)	T.S (15mg.kg @12h)	5	999	3.535379356	0.287	0.597
A.C (5mg.kg BID)	T.S (7.5mg.kg @12h)	5	999	7.32759816	0.608	0.804
A.C (5mg.kg BID)	anti-IHF	5	999	6.935213676	0.182	0.594
A.C (5mg.kg BID)	anti-rsPilA	5	999	13.02443275	0.193	0.594
Ofloxacin	Saline	6	999	3.783514155	0.107	0.495
Ofloxacin	T.S (15mg.kg @12h)	6	999	0.087007773	0.638	0.804
Ofloxacin	T.S (7.5mg.kg @12h)	6	999	3.107635316	0.091	0.495
Ofloxacin	anti-IHF	6	999	2.086076445	0.285	0.597
Ofloxacin	anti-rsPilA	6	999	1.959175378	0.293	0.597
Saline	T.S (15mg.kg @12h)	6	999	1.564239124	0.408	0.668
Saline	T.S (7.5mg.kg @12h)	6	999	8.36456435	0.108	0.495
Saline	anti-IHF	6	999	0.050078554	0.808	0.881
Saline	anti-rsPilA	6	999	0.614444049	0.299	0.597
T.S (15mg.kg @12h)	anti-IHF	6	999	0.883365436	0.405	0.668
T.S (15mg.kg @12h)	anti-rsPilA	6	999	0.442874581	0.606	0.804
T.S (7.5mg.kg @12h)	T.S (15mg.kg @12h)	6	999	1.410500362	0.392	0.668
T.S (7.5mg.kg @12h)	anti-IHF	6	999	5.125612879	0.054	0.495
T.S (7.5mg.kg @12h)	anti-rsPilA	6	999	7.144458047	0.096	0.495
anti-IHF	anti-rsPilA	6	999	0.21023194	0.604	0.804

Omnibus PERMDISP Test

p-value 0.085

Supplementary File 1
Beta Diversity Output

Fecal Bray-Curtis PERMDISP-2DPT

Pairwise Permdisp Results (999 permutations)

Group 1	Group 2	Sample size	Permutations	F-value	p-value	q-value
A.C (10mg.kg PO 7d)	Ofloxacin	4	999	inf	1.000	1.000
A.C (10mg.kg PO 7d)	Saline	5	999	6.030578034	0.088	0.424
A.C (10mg.kg PO 7d)	T.S (15mg.kg @12h)	5	999	43.43576296	0.106	0.424
A.C (10mg.kg PO 7d)	T.S (7.5mg.kg @12h)	5	999	4.92210171	0.105	0.424
A.C (10mg.kg PO 7d)	anti-IHF	5	999	50.34260539	0.097	0.424
A.C (10mg.kg PO 7d)	anti-rsPilA	5	999	7.510531891	0.130	0.468
A.C (2.5mg.kg BID)	A.C (10mg.kg PO 7d)	4	999	inf	1.000	1.000
A.C (2.5mg.kg BID)	A.C (5mg.kg BID)	4	999	inf	0.828	1.000
A.C (2.5mg.kg BID)	Ofloxacin	4	999	inf	0.816	1.000
A.C (2.5mg.kg BID)	Saline	5	999	7.291590367	0.098	0.424
A.C (2.5mg.kg BID)	T.S (15mg.kg @12h)	5	999	52.36981022	0.291	0.656
A.C (2.5mg.kg BID)	T.S (7.5mg.kg @12h)	5	999	6.016646419	0.184	0.526
A.C (2.5mg.kg BID)	anti-IHF	5	999	58.73867273	0.190	0.526
A.C (2.5mg.kg BID)	anti-rsPilA	5	999	8.874283057	0.086	0.424
A.C (5mg.kg BID)	A.C (10mg.kg PO 7d)	4	999	inf	1.000	1.000
A.C (5mg.kg BID)	Ofloxacin	4	999	inf	1.000	1.000
A.C (5mg.kg BID)	Saline	5	999	5.433963786	0.102	0.424
A.C (5mg.kg BID)	T.S (15mg.kg @12h)	5	999	39.20400269	0.104	0.424
A.C (5mg.kg BID)	T.S (7.5mg.kg @12h)	5	999	4.406539587	0.211	0.543
A.C (5mg.kg BID)	anti-IHF	5	999	46.31279776	0.103	0.424
A.C (5mg.kg BID)	anti-rsPilA	5	999	6.859281596	0.310	0.656
Ofloxacin	Saline	5	999	0.139092349	0.592	0.969
Ofloxacin	T.S (15mg.kg @12h)	5	999	1.18424122	0.621	0.972
Ofloxacin	T.S (7.5mg.kg @12h)	5	999	0.049835865	0.902	1.000
Ofloxacin	anti-IHF	5	999	5.066079545	0.498	0.907
Ofloxacin	anti-rsPilA	5	999	0.504080634	0.504	0.907
Saline	T.S (15mg.kg @12h)	6	999	0.002213864	1.000	1.000
Saline	T.S (7.5mg.kg @12h)	6	999	0.015617584	0.794	1.000
Saline	anti-IHF	6	999	0.497543551	0.155	0.507
Saline	anti-rsPilA	6	999	0.10252513	1.000	1.000
T.S (15mg.kg @12h)	anti-IHF	6	999	1.568895335	0.308	0.656
T.S (15mg.kg @12h)	anti-rsPilA	6	999	0.139512311	0.808	1.000
T.S (7.5mg.kg @12h)	T.S (15mg.kg @12h)	6	999	0.043267404	0.805	1.000
T.S (7.5mg.kg @12h)	anti-IHF	6	999	0.705860446	0.484	0.907
T.S (7.5mg.kg @12h)	anti-rsPilA	6	999	0.190855606	0.583	0.969
anti-IHF	anti-rsPilA	6	999	0.077246348	0.702	1.000

Omnibus PERMDISP Test

p-value 0.023

Supplementary File 1
Beta Diversity Output

Fecal Bray-Curtis PERMDISP-5DPT

Pairwise Permdisp Results (999 permutations)

Group 1	Group 2	Sample size	Permutations	F-value	p-value	q-value
A.C (10mg.kg PO 7d)	Ofloxacin	6	999	0.247706963	0.114	0.518
A.C (10mg.kg PO 7d)	Saline	6	999	0.209289826	0.300	0.820
A.C (10mg.kg PO 7d)	T.S (15mg.kg @12h)	6	999	0.734518351	0.112	0.518
A.C (10mg.kg PO 7d)	T.S (7.5mg.kg @12h)	6	999	0.541489293	0.112	0.518
A.C (10mg.kg PO 7d)	anti-IHF	6	999	2.2479519	0.095	0.518
A.C (10mg.kg PO 7d)	anti-rsPilA	5	999	0.236831688	0.394	0.820
A.C (2.5mg.kg BID)	A.C (10mg.kg PO 7d)	6	999	0.381246322	0.631	0.962
A.C (2.5mg.kg BID)	A.C (5mg.kg BID)	5	999	0.023903558	0.909	0.962
A.C (2.5mg.kg BID)	Ofloxacin	6	999	0.141742051	0.476	0.902
A.C (2.5mg.kg BID)	Saline	6	999	0.173963753	0.382	0.820
A.C (2.5mg.kg BID)	T.S (15mg.kg @12h)	6	999	0.019193021	0.757	0.962
A.C (2.5mg.kg BID)	T.S (7.5mg.kg @12h)	6	999	0.040573444	0.903	0.962
A.C (2.5mg.kg BID)	anti-IHF	6	999	0.074731724	0.372	0.820
A.C (2.5mg.kg BID)	anti-rsPilA	5	999	0.063205038	0.797	0.962
A.C (5mg.kg BID)	A.C (10mg.kg PO 7d)	5	999	0.400517521	0.610	0.962
A.C (5mg.kg BID)	Ofloxacin	5	999	3.39E-01	0.402	0.820
A.C (5mg.kg BID)	Saline	5	999	0.807888159	0.398	0.820
A.C (5mg.kg BID)	T.S (15mg.kg @12h)	5	999	0.040320069	0.728	0.962
A.C (5mg.kg BID)	T.S (7.5mg.kg @12h)	5	999	0.000722658	0.909	0.962
A.C (5mg.kg BID)	anti-IHF	5	999	6.281755165	0.095	0.518
A.C (5mg.kg BID)	anti-rsPilA	4	999	inf	1.000	1.000
Ofloxacin	Saline	6	999	0.01340868	0.783	0.962
Ofloxacin	T.S (15mg.kg @12h)	6	999	0.539336735	0.410	0.820
Ofloxacin	T.S (7.5mg.kg @12h)	6	999	0.219629045	0.718	0.962
Ofloxacin	anti-IHF	6	999	5.222076172	0.054	0.518
Ofloxacin	anti-rsPilA	5	999	0.038558194	0.889	0.962
Saline	T.S (15mg.kg @12h)	6	999	0.918283073	0.372	0.820
Saline	T.S (7.5mg.kg @12h)	6	999	0.386351505	0.602	0.962
Saline	anti-IHF	6	999	8.287728615	0.115	0.518
Saline	anti-rsPilA	5	999	0.160186345	0.897	0.962
T.S (15mg.kg @12h)	anti-IHF	6	999	2.459282871	0.302	0.820
T.S (15mg.kg @12h)	anti-rsPilA	5	999	0.404367396	0.698	0.962
T.S (7.5mg.kg @12h)	T.S (15mg.kg @12h)	6	999	0.034699477	0.946	0.973
T.S (7.5mg.kg @12h)	anti-IHF	6	999	2.271051771	0.222	0.820
T.S (7.5mg.kg @12h)	anti-rsPilA	5	999	0.096416094	0.804	0.962
anti-IHF	anti-rsPilA	5	999	10.01385541	0.114	0.518

Omnibus PERMDISP Test

p-value 0.779

Supplementary File 1
Beta Diversity Output

Fecal Bray-Curtis PERMDISP-7DPT

Pairwise Permdisp Results (999 permutations)

Group 1	Group 2	Sample size	Permutations	F-value	p-value	q-value
A.C (10mg.kg PO 7d)	Ofloxacin	6	999	0.001320945	1.000	1.000
A.C (10mg.kg PO 7d)	Saline	5	999	2.266281728	0.305	1.000
A.C (10mg.kg PO 7d)	T.S (15mg.kg @12h)	6	999	0.022399724	0.598	1.000
A.C (10mg.kg PO 7d)	T.S (7.5mg.kg @12h)	6	999	0.048283201	0.090	1.000
A.C (10mg.kg PO 7d)	anti-IHF	5	999	0.267208499	0.402	1.000
A.C (10mg.kg PO 7d)	anti-rsPilA	5	999	4.332101146	0.095	1.000
A.C (2.5mg.kg BID)	A.C (10mg.kg PO 7d)	6	999	0.805348916	0.383	1.000
A.C (2.5mg.kg BID)	A.C (5mg.kg BID)	4	999	17.11446169	1.000	1.000
A.C (2.5mg.kg BID)	Ofloxacin	6	999	0.573310055	0.297	1.000
A.C (2.5mg.kg BID)	Saline	5	999	1.319980944	0.518	1.000
A.C (2.5mg.kg BID)	T.S (15mg.kg @12h)	6	999	0.211820206	0.311	1.000
A.C (2.5mg.kg BID)	T.S (7.5mg.kg @12h)	6	999	0.505460542	0.101	1.000
A.C (2.5mg.kg BID)	anti-IHF	5	999	0.754994862	0.682	1.000
A.C (2.5mg.kg BID)	anti-rsPilA	5	999	1.721469352	0.571	1.000
A.C (5mg.kg BID)	A.C (10mg.kg PO 7d)	4	999	167.9223469	0.251	1.000
A.C (5mg.kg BID)	Ofloxacin	4	999	2.16E+01	0.279	1.000
A.C (5mg.kg BID)	Saline	3	999	inf	1.000	1.000
A.C (5mg.kg BID)	T.S (15mg.kg @12h)	4	999	8.964205309	0.495	1.000
A.C (5mg.kg BID)	T.S (7.5mg.kg @12h)	4	999	5.739933286	0.500	1.000
A.C (5mg.kg BID)	anti-IHF	3	999	inf	1.000	1.000
A.C (5mg.kg BID)	anti-rsPilA	3	999	inf	1.000	1.000
Ofloxacin	Saline	5	999	0.262726796	0.723	1.000
Ofloxacin	T.S (15mg.kg @12h)	6	999	0.022673477	0.902	1.000
Ofloxacin	T.S (7.5mg.kg @12h)	6	999	0.032005365	0.708	1.000
Ofloxacin	anti-IHF	5	999	0.024446505	1.000	1.000
Ofloxacin	anti-rsPilA	5	999	0.518572223	0.794	1.000
Saline	T.S (15mg.kg @12h)	5	999	0.209529567	0.777	1.000
Saline	T.S (7.5mg.kg @12h)	5	999	0.013915626	0.905	1.000
Saline	anti-IHF	4	999	inf	1.000	1.000
Saline	anti-rsPilA	4	999	inf	1.000	1.000
T.S (15mg.kg @12h)	anti-IHF	5	999	0.055321704	0.900	1.000
T.S (15mg.kg @12h)	anti-rsPilA	5	999	0.345047283	0.599	1.000
T.S (7.5mg.kg @12h)	T.S (15mg.kg @12h)	6	999	0.07275279	0.795	1.000
T.S (7.5mg.kg @12h)	anti-IHF	5	999	0.005377103	1.000	1.000
T.S (7.5mg.kg @12h)	anti-rsPilA	5	999	0.052634747	0.790	1.000
anti-IHF	anti-rsPilA	4	999	inf	1.000	1.000

Omnibus PERMDISP Test

p-value 0.695

Supplementary File 1
Beta Diversity Output

Fecal Bray-Curtis PERMDISP-9DPT

Pairwise Permdisp Results (999 permutations)

Group 1	Group 2	Sample size	Permutations	F-value	p-value	q-value
A.C (10mg.kg PO 7d)	Ofloxacin	5	999	0.004663819	0.902	1.000
A.C (10mg.kg PO 7d)	Saline	3	999	inf	1.000	1.000
A.C (10mg.kg PO 7d)	T.S (15mg.kg @12h)	5	999	1.074512105	0.417	1.000
A.C (10mg.kg PO 7d)	T.S (7.5mg.kg @12h)	5	999	0.039519236	0.826	1.000
A.C (10mg.kg PO 7d)	anti-IHF	5	999	0.035739085	0.899	1.000
A.C (10mg.kg PO 7d)	anti-rsPilA	4	999	inf	1.000	1.000
A.C (2.5mg.kg BID)	A.C (10mg.kg PO 7d)	5	999	0.688978785	0.591	1.000
A.C (2.5mg.kg BID)	A.C (5mg.kg BID)	5	999	1.327467741	0.685	1.000
A.C (2.5mg.kg BID)	Ofloxacin	6	999	0.167025736	0.320	1.000
A.C (2.5mg.kg BID)	Saline	4	999	34.17441979	0.763	1.000
A.C (2.5mg.kg BID)	T.S (15mg.kg @12h)	6	999	2.533045511	0.095	1.000
A.C (2.5mg.kg BID)	T.S (7.5mg.kg @12h)	6	999	0.161583662	0.397	1.000
A.C (2.5mg.kg BID)	anti-IHF	6	999	0.905723549	0.091	1.000
A.C (2.5mg.kg BID)	anti-rsPilA	5	999	15.99088806	0.209	1.000
A.C (5mg.kg BID)	A.C (10mg.kg PO 7d)	4	999	inf	1.000	1.000
A.C (5mg.kg BID)	Ofloxacin	5	999	1.16E-03	1.000	1.000
A.C (5mg.kg BID)	Saline	3	999	inf	1.000	1.000
A.C (5mg.kg BID)	T.S (15mg.kg @12h)	5	999	0.218170787	0.587	1.000
A.C (5mg.kg BID)	T.S (7.5mg.kg @12h)	5	999	0.179099601	0.795	1.000
A.C (5mg.kg BID)	anti-IHF	5	999	1.515294657	0.403	1.000
A.C (5mg.kg BID)	anti-rsPilA	4	999	inf	0.830	1.000
Ofloxacin	Saline	4	999	2.703090339	1.000	1.000
Ofloxacin	T.S (15mg.kg @12h)	6	999	0.022519336	1.000	1.000
Ofloxacin	T.S (7.5mg.kg @12h)	6	999	0.034877162	0.813	1.000
Ofloxacin	anti-IHF	6	999	0.012457776	1.000	1.000
Ofloxacin	anti-rsPilA	5	999	0.881983339	1.000	1.000
Saline	T.S (15mg.kg @12h)	4	999	76.02562538	0.512	1.000
Saline	T.S (7.5mg.kg @12h)	4	999	14.65164599	1.000	1.000
Saline	anti-IHF	4	999	299.2384227	0.753	1.000
Saline	anti-rsPilA	3	999	inf	1.000	1.000
T.S (15mg.kg @12h)	anti-IHF	6	999	1.667510585	0.160	1.000
T.S (15mg.kg @12h)	anti-rsPilA	5	999	20.84793651	0.294	1.000
T.S (7.5mg.kg @12h)	T.S (15mg.kg @12h)	6	999	0.531993496	0.293	1.000
T.S (7.5mg.kg @12h)	anti-IHF	6	999	0.039801381	0.302	1.000
T.S (7.5mg.kg @12h)	anti-rsPilA	5	999	5.790935328	0.312	1.000
anti-IHF	anti-rsPilA	5	999	108.975984	0.097	1.000

Omnibus PERMDISP Test

p-value 0.793

Supplementary File 1
Beta Diversity Output

Fecal Jaccard PERMDISP-Baseline

Pairwise Permdisp Results (999 permutations)

Group 1	Group 2	Sample size	Permutations	F-value	p-value	q-value
A.C (10mg.kg PO 7d)	Ofloxacin	6	999	21.11245106	0.103	0.472
A.C (10mg.kg PO 7d)	Saline	6	999	35.66489425	0.097	0.472
A.C (10mg.kg PO 7d)	T.S (15mg.kg @12h)	6	999	31.25437927	0.114	0.472
A.C (10mg.kg PO 7d)	T.S (7.5mg.kg @12h)	6	999	18.63789609	0.101	0.472
A.C (10mg.kg PO 7d)	anti-IHF	6	999	69.82711364	0.047	0.472
A.C (10mg.kg PO 7d)	anti-rsPilA	6	999	115.1926531	0.118	0.472
A.C (2.5mg.kg BID)	A.C (10mg.kg PO 7d)	5	999	5.096310756	0.421	0.606
A.C (2.5mg.kg BID)	A.C (5mg.kg BID)	4	999	inf	1.000	1.000
A.C (2.5mg.kg BID)	Ofloxacin	5	999	8.339986486	0.364	0.606
A.C (2.5mg.kg BID)	Saline	5	999	16.30880916	0.279	0.606
A.C (2.5mg.kg BID)	T.S (15mg.kg @12h)	5	999	13.66419836	0.371	0.606
A.C (2.5mg.kg BID)	T.S (7.5mg.kg @12h)	5	999	6.936618689	0.304	0.606
A.C (2.5mg.kg BID)	anti-IHF	5	999	45.65256712	0.103	0.472
A.C (2.5mg.kg BID)	anti-rsPilA	5	999	131.2185545	0.106	0.472
A.C (5mg.kg BID)	A.C (10mg.kg PO 7d)	5	999	5.428019695	0.418	0.606
A.C (5mg.kg BID)	Ofloxacin	5	999	8.15E+00	0.322	0.606
A.C (5mg.kg BID)	Saline	5	999	15.99674381	0.309	0.606
A.C (5mg.kg BID)	T.S (15mg.kg @12h)	5	999	13.35435599	0.400	0.606
A.C (5mg.kg BID)	T.S (7.5mg.kg @12h)	5	999	6.758652472	0.794	0.886
A.C (5mg.kg BID)	anti-IHF	5	999	44.74077481	0.310	0.606
A.C (5mg.kg BID)	anti-rsPilA	5	999	128.7971095	0.207	0.606
Ofloxacin	Saline	6	999	0.234146757	0.573	0.711
Ofloxacin	T.S (15mg.kg @12h)	6	999	0.001777886	1.000	1.000
Ofloxacin	T.S (7.5mg.kg @12h)	6	999	0.121877083	0.410	0.606
Ofloxacin	anti-IHF	6	999	0.162583502	0.516	0.688
Ofloxacin	anti-rsPilA	6	999	0.574287864	0.314	0.606
Saline	T.S (15mg.kg @12h)	6	999	0.362041294	0.502	0.688
Saline	T.S (7.5mg.kg @12h)	6	999	0.769815013	0.322	0.606
Saline	anti-IHF	6	999	0.036359715	1.000	1.000
Saline	anti-rsPilA	6	999	0.032993999	0.812	0.886
T.S (15mg.kg @12h)	anti-IHF	6	999	0.304373969	0.605	0.726
T.S (15mg.kg @12h)	anti-rsPilA	6	999	1.041705871	0.317	0.606
T.S (7.5mg.kg @12h)	T.S (15mg.kg @12h)	6	999	0.122581706	0.655	0.761
T.S (7.5mg.kg @12h)	anti-IHF	6	999	0.757923937	0.383	0.606
T.S (7.5mg.kg @12h)	anti-rsPilA	6	999	1.6182957	0.114	0.472
anti-IHF	anti-rsPilA	6	999	0.369864736	0.544	0.699

Omnibus PERMDISP Test

p-value 0.009

Supplementary File 1
Beta Diversity Output

Fecal Jaccard PERMDISP-2DPT

Pairwise Permdisp Results (999 permutations)

Group 1	Group 2	Sample size	Permutations	F-value	p-value	q-value
A.C (10mg.kg PO 7d)	Ofloxacin	4	999	inf	1.000	1.000
A.C (10mg.kg PO 7d)	Saline	5	999	6.204042041	0.108	0.671
A.C (10mg.kg PO 7d)	T.S (15mg.kg @12h)	5	999	4.191690784	0.204	0.671
A.C (10mg.kg PO 7d)	T.S (7.5mg.kg @12h)	5	999	7.260004657	0.301	0.671
A.C (10mg.kg PO 7d)	anti-IHF	5	999	10.84189944	0.103	0.671
A.C (10mg.kg PO 7d)	anti-rsPilA	5	999	6.343127724	0.107	0.671
A.C (2.5mg.kg BID)	A.C (10mg.kg PO 7d)	4	999	inf	0.835	1.000
A.C (2.5mg.kg BID)	A.C (5mg.kg BID)	4	999	inf	1.000	1.000
A.C (2.5mg.kg BID)	Ofloxacin	4	999	inf	1.000	1.000
A.C (2.5mg.kg BID)	Saline	5	999	13.44674403	0.302	0.671
A.C (2.5mg.kg BID)	T.S (15mg.kg @12h)	5	999	11.60169655	0.208	0.671
A.C (2.5mg.kg BID)	T.S (7.5mg.kg @12h)	5	999	14.07058758	0.296	0.671
A.C (2.5mg.kg BID)	anti-IHF	5	999	24.13650746	0.310	0.671
A.C (2.5mg.kg BID)	anti-rsPilA	5	999	13.28689445	0.216	0.671
A.C (5mg.kg BID)	A.C (10mg.kg PO 7d)	4	999	1.74396E+16	1.000	1.000
A.C (5mg.kg BID)	Ofloxacin	4	999	inf	1.000	1.000
A.C (5mg.kg BID)	Saline	5	999	8.886126232	0.281	0.671
A.C (5mg.kg BID)	T.S (15mg.kg @12h)	5	999	6.831000683	0.096	0.671
A.C (5mg.kg BID)	T.S (7.5mg.kg @12h)	5	999	9.826889419	0.278	0.671
A.C (5mg.kg BID)	anti-IHF	5	999	15.74428583	0.083	0.671
A.C (5mg.kg BID)	anti-rsPilA	5	999	8.928409626	0.414	0.710
Ofloxacin	Saline	5	999	5.426456184	0.686	0.988
Ofloxacin	T.S (15mg.kg @12h)	5	999	3.463341374	1.000	1.000
Ofloxacin	T.S (7.5mg.kg @12h)	5	999	6.500052857	0.599	0.906
Ofloxacin	anti-IHF	5	999	9.427926819	0.397	0.710
Ofloxacin	anti-rsPilA	5	999	5.58871843	0.317	0.671
Saline	T.S (15mg.kg @12h)	6	999	0.491893942	0.284	0.671
Saline	T.S (7.5mg.kg @12h)	6	999	0.192470456	0.604	0.906
Saline	anti-IHF	6	999	0.011007717	1.000	1.000
Saline	anti-rsPilA	6	999	0.015342991	0.891	1.000
T.S (15mg.kg @12h)	anti-IHF	6	999	0.498895748	0.388	0.710
T.S (15mg.kg @12h)	anti-rsPilA	6	999	0.665868271	0.400	0.710
T.S (7.5mg.kg @12h)	T.S (15mg.kg @12h)	6	999	1.261994277	0.216	0.671
T.S (7.5mg.kg @12h)	anti-IHF	6	999	0.34991751	0.563	0.906
T.S (7.5mg.kg @12h)	anti-rsPilA	6	999	0.09785229	1.000	1.000
anti-IHF	anti-rsPilA	6	999	0.05894045	0.806	1.000

Omnibus PERMDISP Test

p-value 0.053

Supplementary File 1
Beta Diversity Output

Fecal Jaccard PERMDISP-5DPT

Pairwise Permdisp Results (999 permutations)

Group 1	Group 2	Sample size	Permutations	F-value	p-value	q-value
A.C (10mg.kg PO 7d)	Ofloxacin	6	999	0.293366701	0.107	0.800
A.C (10mg.kg PO 7d)	Saline	6	999	0.530238428	0.098	0.800
A.C (10mg.kg PO 7d)	T.S (15mg.kg @12h)	6	999	0.449464995	0.197	0.800
A.C (10mg.kg PO 7d)	T.S (7.5mg.kg @12h)	6	999	1.065521053	0.173	0.800
A.C (10mg.kg PO 7d)	anti-IHF	6	999	0.221249056	0.311	0.800
A.C (10mg.kg PO 7d)	anti-rsPilA	5	999	0.136409098	0.799	1.000
A.C (2.5mg.kg BID)	A.C (10mg.kg PO 7d)	6	999	0.385778407	0.282	0.800
A.C (2.5mg.kg BID)	A.C (5mg.kg BID)	5	999	0.540537363	0.630	0.989
A.C (2.5mg.kg BID)	Ofloxacin	6	999	0.08395746	0.322	0.800
A.C (2.5mg.kg BID)	Saline	6	999	0.046173575	0.698	0.989
A.C (2.5mg.kg BID)	T.S (15mg.kg @12h)	6	999	0.053825386	0.495	0.938
A.C (2.5mg.kg BID)	T.S (7.5mg.kg @12h)	6	999	0.030475017	0.678	0.989
A.C (2.5mg.kg BID)	anti-IHF	6	999	0.14420864	0.286	0.800
A.C (2.5mg.kg BID)	anti-rsPilA	5	999	0.777868771	0.803	1.000
A.C (5mg.kg BID)	A.C (10mg.kg PO 7d)	5	999	0.030648966	0.906	1.000
A.C (5mg.kg BID)	Ofloxacin	5	999	1.45E+00	0.387	0.800
A.C (5mg.kg BID)	Saline	5	999	13.95284394	0.195	0.800
A.C (5mg.kg BID)	T.S (15mg.kg @12h)	5	999	4.105886824	0.386	0.800
A.C (5mg.kg BID)	T.S (7.5mg.kg @12h)	5	999	2.405012505	0.714	0.989
A.C (5mg.kg BID)	anti-IHF	5	999	2.055858989	0.395	0.800
A.C (5mg.kg BID)	anti-rsPilA	4	999	inf	1.000	1.000
Ofloxacin	Saline	6	999	0.04811572	0.905	1.000
Ofloxacin	T.S (15mg.kg @12h)	6	999	0.021947323	1.000	1.000
Ofloxacin	T.S (7.5mg.kg @12h)	6	999	0.531724583	0.244	0.800
Ofloxacin	anti-IHF	6	999	0.027002059	0.900	1.000
Ofloxacin	anti-rsPilA	5	999	2.441953007	0.906	1.000
Saline	T.S (15mg.kg @12h)	6	999	0.005498274	1.000	1.000
Saline	T.S (7.5mg.kg @12h)	6	999	0.496233453	0.151	0.800
Saline	anti-IHF	6	999	0.28544666	0.400	0.800
Saline	anti-rsPilA	5	999	22.11261925	0.284	0.800
T.S (15mg.kg @12h)	anti-IHF	6	999	0.149571293	0.392	0.800
T.S (15mg.kg @12h)	anti-rsPilA	5	999	6.594296593	0.628	0.989
T.S (7.5mg.kg @12h)	T.S (15mg.kg @12h)	6	999	0.483377146	0.528	0.950
T.S (7.5mg.kg @12h)	anti-IHF	6	999	0.85166404	0.317	0.800
T.S (7.5mg.kg @12h)	anti-rsPilA	5	999	3.262338643	0.680	0.989
anti-IHF	anti-rsPilA	5	999	3.706448387	1.000	1.000

Omnibus PERMDISP Test

p-value 0.845

Supplementary File 1
Beta Diversity Output

Fecal Jaccard PERMDISP-7DPT

Pairwise Permdisp Results (999 permutations)

Group 1	Group 2	Sample size	Permutations	F-value	p-value	q-value
A.C (10mg.kg PO 7d)	Ofloxacin	6	999	0.047562243	0.597	1.000
A.C (10mg.kg PO 7d)	Saline	5	999	1.972042003	0.409	1.000
A.C (10mg.kg PO 7d)	T.S (15mg.kg @12h)	6	999	0.001495434	0.892	1.000
A.C (10mg.kg PO 7d)	T.S (7.5mg.kg @12h)	6	999	0.131563549	0.498	1.000
A.C (10mg.kg PO 7d)	anti-IHF	5	999	3.354872145	0.103	1.000
A.C (10mg.kg PO 7d)	anti-rsPilA	5	999	3.191581209	0.290	1.000
A.C (2.5mg.kg BID)	A.C (10mg.kg PO 7d)	6	999	0.02951131	0.471	1.000
A.C (2.5mg.kg BID)	A.C (5mg.kg BID)	4	999	39.60477692	1.000	1.000
A.C (2.5mg.kg BID)	Ofloxacin	6	999	0.000121484	0.941	1.000
A.C (2.5mg.kg BID)	Saline	5	999	1.317446393	0.609	1.000
A.C (2.5mg.kg BID)	T.S (15mg.kg @12h)	6	999	0.02426476	0.682	1.000
A.C (2.5mg.kg BID)	T.S (7.5mg.kg @12h)	6	999	0.006826138	0.843	1.000
A.C (2.5mg.kg BID)	anti-IHF	5	999	2.09560177	0.491	1.000
A.C (2.5mg.kg BID)	anti-rsPilA	5	999	2.004929468	0.504	1.000
A.C (5mg.kg BID)	A.C (10mg.kg PO 7d)	4	999	77.79774951	0.753	1.000
A.C (5mg.kg BID)	Ofloxacin	4	999	7.10E+01	0.519	1.000
A.C (5mg.kg BID)	Saline	3	999	inf	1.000	1.000
A.C (5mg.kg BID)	T.S (15mg.kg @12h)	4	999	139.5240699	0.270	1.000
A.C (5mg.kg BID)	T.S (7.5mg.kg @12h)	4	999	209.1150831	0.511	1.000
A.C (5mg.kg BID)	anti-IHF	3	999	inf	1.000	1.000
A.C (5mg.kg BID)	anti-rsPilA	3	999	inf	1.000	1.000
Ofloxacin	Saline	5	999	2.400394608	0.706	1.000
Ofloxacin	T.S (15mg.kg @12h)	6	999	0.043103152	1.000	1.000
Ofloxacin	T.S (7.5mg.kg @12h)	6	999	0.007779483	0.737	1.000
Ofloxacin	anti-IHF	5	999	3.803657886	0.614	1.000
Ofloxacin	anti-rsPilA	5	999	3.640271455	0.804	1.000
Saline	T.S (15mg.kg @12h)	5	999	3.707365612	0.493	1.000
Saline	T.S (7.5mg.kg @12h)	5	999	7.71788098	0.712	1.000
Saline	anti-IHF	4	999	inf	1.000	1.000
Saline	anti-rsPilA	4	999	inf	1.000	1.000
T.S (15mg.kg @12h)	anti-IHF	5	999	6.23087894	0.586	1.000
T.S (15mg.kg @12h)	anti-rsPilA	5	999	5.933602476	0.509	1.000
T.S (7.5mg.kg @12h)	T.S (15mg.kg @12h)	6	999	0.151347563	0.587	1.000
T.S (7.5mg.kg @12h)	anti-IHF	5	999	11.98640538	0.089	1.000
T.S (7.5mg.kg @12h)	anti-rsPilA	5	999	11.49140769	0.289	1.000
anti-IHF	anti-rsPilA	4	999	inf	1.000	1.000

Omnibus PERMDISP Test

p-value 0.095

Supplementary File 1
Beta Diversity Output

Fecal Jaccard PERMDISP-9DPT

Pairwise Permdisp Results (999 permutations)

Group 1	Group 2	Sample size	Permutations	F-value	p-value	q-value
A.C (10mg.kg PO 7d)	Ofloxacin	5	999	1.886119104	0.516	1.000
A.C (10mg.kg PO 7d)	Saline	3	999	inf	1.000	1.000
A.C (10mg.kg PO 7d)	T.S (15mg.kg @12h)	5	999	1.52306292	0.412	1.000
A.C (10mg.kg PO 7d)	T.S (7.5mg.kg @12h)	5	999	3.773318831	0.403	1.000
A.C (10mg.kg PO 7d)	anti-IHF	5	999	18.28552449	0.101	1.000
A.C (10mg.kg PO 7d)	anti-rsPilA	4	999	inf	1.000	1.000
A.C (2.5mg.kg BID)	A.C (10mg.kg PO 7d)	5	999	1.5678515	0.505	1.000
A.C (2.5mg.kg BID)	A.C (5mg.kg BID)	5	999	2.054214584	0.510	1.000
A.C (2.5mg.kg BID)	Ofloxacin	6	999	0.03220719	0.602	1.000
A.C (2.5mg.kg BID)	Saline	4	999	21.70969548	0.747	1.000
A.C (2.5mg.kg BID)	T.S (15mg.kg @12h)	6	999	0.223989454	0.314	1.000
A.C (2.5mg.kg BID)	T.S (7.5mg.kg @12h)	6	999	0.064384668	0.593	1.000
A.C (2.5mg.kg BID)	anti-IHF	6	999	0.050113939	0.553	1.000
A.C (2.5mg.kg BID)	anti-rsPilA	5	999	1.487008977	0.694	1.000
A.C (5mg.kg BID)	A.C (10mg.kg PO 7d)	4	999	inf	1.000	1.000
A.C (5mg.kg BID)	Ofloxacin	5	999	2.39E+00	0.421	1.000
A.C (5mg.kg BID)	Saline	3	999	inf	1.000	1.000
A.C (5mg.kg BID)	T.S (15mg.kg @12h)	5	999	2.277117716	0.205	1.000
A.C (5mg.kg BID)	T.S (7.5mg.kg @12h)	5	999	4.739195029	0.294	1.000
A.C (5mg.kg BID)	anti-IHF	5	999	24.97701445	0.104	1.000
A.C (5mg.kg BID)	anti-rsPilA	4	999	inf	0.492	1.000
Ofloxacin	Saline	4	999	20.4525579	1.000	1.000
Ofloxacin	T.S (15mg.kg @12h)	6	999	0.440813571	0.656	1.000
Ofloxacin	T.S (7.5mg.kg @12h)	6	999	0.002040026	1.000	1.000
Ofloxacin	anti-IHF	6	999	0.204482777	0.956	1.000
Ofloxacin	anti-rsPilA	5	999	1.802249848	0.584	1.000
Saline	T.S (15mg.kg @12h)	4	999	44.11196349	0.738	1.000
Saline	T.S (7.5mg.kg @12h)	4	999	38.93053067	1.000	1.000
Saline	anti-IHF	4	999	327.7091158	0.762	1.000
Saline	anti-rsPilA	3	999	inf	1.000	1.000
T.S (15mg.kg @12h)	anti-IHF	6	999	0.227050996	0.523	1.000
T.S (15mg.kg @12h)	anti-rsPilA	5	999	1.402992899	1.000	1.000
T.S (7.5mg.kg @12h)	T.S (15mg.kg @12h)	6	999	0.763945682	0.203	1.000
T.S (7.5mg.kg @12h)	anti-IHF	6	999	0.46094087	0.093	1.000
T.S (7.5mg.kg @12h)	anti-rsPilA	5	999	3.610604298	0.403	1.000
anti-IHF	anti-rsPilA	5	999	17.18805574	0.423	1.000

Omnibus PERMDISP Test

p-value 0.76

Supplementary File 1
Beta Diversity Output

NPL Weighted UniFrac-Baseline

PERMANOVA Pairwise Comparisons (999 permutations)

Group 1	Group 2	Sample size	Permutations	pseudo-F	p-value	q-value
AC-PO7d	Ofloxacin	6	999	2.768	0.112	0.581
AC-PO7d	Saline	6	999	1.492	0.385	0.866
AC-PO7d	TS-30mg/kg	6	999	1.594	0.193	0.584
AC-PO7d	TS-15mg/kg	6	999	2.201	0.196	0.584
AC-PO7d	anti-tip-chimer	6	999	2.478	0.098	0.581
AC-PO7d	anti-rsPilA	6	999	1.745	0.203	0.584
AC-5mg/kg	AC-PO7d	6	999	6.045	0.113	0.581
AC-5mg/kg	AC-10mg/kg	5	999	0.706	0.810	1.000
AC-5mg/kg	Ofloxacin	6	999	2.229	0.106	0.581
AC-5mg/kg	Saline	6	999	0.697	0.610	0.878
AC-5mg/kg	TS-30mg/kg	6	999	3.866	0.101	0.581
AC-5mg/kg	TS-15mg/kg	6	999	0.814	0.781	1.000
AC-5mg/kg	anti-tip-chimer	6	999	2.140	0.082	0.581
AC-5mg/kg	anti-rsPilA	6	999	1.458	0.211	0.584
AC-10mg/kg	AC-PO7d	5	999	3.100	0.091	0.581
AC-10mg/kg	Ofloxacin	5	999	0.885	0.590	0.878
AC-10mg/kg	Saline	5	999	0.359	0.896	1.000
AC-10mg/kg	TS-30mg/kg	5	999	2.076	0.193	0.584
AC-10mg/kg	TS-15mg/kg	5	999	0.198	1.000	1.000
AC-10mg/kg	anti-tip-chimer	5	999	0.837	0.698	0.966
AC-10mg/kg	anti-rsPilA	5	999	0.494	1.000	1.000
Ofloxacin	Saline	6	999	0.570	0.606	0.878
Ofloxacin	TS-30mg/kg	6	999	1.583	0.228	0.586
Ofloxacin	TS-15mg/kg	6	999	0.297	1.000	1.000
Ofloxacin	anti-tip-chimer	6	999	0.782	0.499	0.878
Ofloxacin	anti-rsPilA	6	999	0.652	0.596	0.878
Saline	TS-30mg/kg	6	999	1.030	0.581	0.878
Saline	TS-15mg/kg	6	999	0.171	0.892	1.000
Saline	anti-tip-chimer	6	999	0.764	0.515	0.878
Saline	anti-rsPilA	6	999	0.314	1.000	1.000
TS-30mg/kg	anti-tip-chimer	6	999	1.627	0.189	0.584
TS-30mg/kg	anti-rsPilA	6	999	1.373	0.417	0.878
TS-15mg/kg	TS-30mg/kg	6	999	1.393	0.314	0.754
TS-15mg/kg	anti-tip-chimer	6	999	0.779	0.604	0.878
TS-15mg/kg	anti-rsPilA	6	999	0.203	1.000	1.000
anti-tip-chimer	anti-rsPilA	6	999	0.707	0.795	1.000

Supplementary File 1
Beta Diversity Output

NPL Weighted UniFrac-2DPT

PERMANOVA Pairwise Comparisons (999 permutations)

Group 1	Group 2	Sample size	Permutations	pseudo-F	p-value	q-value
AC-PO7d	Ofloxacin	6	999	5.596	0.121	0.335
AC-PO7d	Saline	6	999	7.078	0.109	0.335
AC-PO7d	TS-30mg/kg	6	999	8.920	0.101	0.335
AC-PO7d	TS-15mg/kg	6	999	4.482	0.104	0.335
AC-PO7d	anti-tip-chimer	6	999	6.196	0.092	0.335
AC-PO7d	anti-rsPilA	6	999	9.883	0.082	0.335
AC-5mg/kg	AC-PO7d	6	999	19.671	0.112	0.335
AC-5mg/kg	AC-10mg/kg	5	999	3.632	0.111	0.335
AC-5mg/kg	Ofloxacin	6	999	1.659	0.290	0.547
AC-5mg/kg	Saline	6	999	1.120	0.298	0.547
AC-5mg/kg	TS-30mg/kg	6	999	7.041	0.109	0.335
AC-5mg/kg	TS-15mg/kg	6	999	1.747	0.395	0.651
AC-5mg/kg	anti-tip-chimer	6	999	1.647	0.089	0.335
AC-5mg/kg	anti-rsPilA	6	999	1.413	0.281	0.547
AC-10mg/kg	AC-PO7d	5	999	6.829	0.096	0.335
AC-10mg/kg	Ofloxacin	5	999	1.061	0.398	0.651
AC-10mg/kg	Saline	5	999	1.153	0.419	0.656
AC-10mg/kg	TS-30mg/kg	5	999	2.466	0.097	0.335
AC-10mg/kg	TS-15mg/kg	5	999	0.835	0.600	0.864
AC-10mg/kg	anti-tip-chimer	5	999	0.385	0.913	0.945
AC-10mg/kg	anti-rsPilA	5	999	0.843	0.699	0.891
Ofloxacin	Saline	6	999	0.392	0.809	0.939
Ofloxacin	TS-30mg/kg	6	999	1.555	0.204	0.470
Ofloxacin	TS-15mg/kg	6	999	0.220	1.000	1.000
Ofloxacin	anti-tip-chimer	6	999	0.491	0.628	0.870
Ofloxacin	anti-rsPilA	6	999	0.581	0.798	0.939
Saline	TS-30mg/kg	6	999	1.729	0.191	0.470
Saline	TS-15mg/kg	6	999	0.329	0.919	0.945
Saline	anti-tip-chimer	6	999	0.610	0.583	0.864
Saline	anti-rsPilA	6	999	0.527	0.892	0.945
TS-30mg/kg	anti-tip-chimer	6	999	2.080	0.209	0.470
TS-30mg/kg	anti-rsPilA	6	999	3.049	0.098	0.335
TS-15mg/kg	TS-30mg/kg	6	999	1.380	0.304	0.547
TS-15mg/kg	anti-tip-chimer	6	999	0.487	0.705	0.891
TS-15mg/kg	anti-rsPilA	6	999	0.797	0.718	0.891
anti-tip-chimer	anti-rsPilA	6	999	0.379	0.901	0.945

Supplementary File 1
Beta Diversity Output

NPL Weighted UniFrac-5DPT

PERMANOVA Pairwise Comparisons (999 permutations)

Group 1	Group 2	Sample size	Permutations	pseudo-F	p-value	q-value
AC-PO7d	Ofloxacin	6	999	1.053	0.402	0.842
AC-PO7d	Saline	6	999	0.963	0.634	0.842
AC-PO7d	TS-30mg/kg	6	999	2.048	0.184	0.770
AC-PO7d	TS-15mg/kg	6	999	2.120	0.200	0.770
AC-PO7d	anti-tip-chimer	6	999	2.191	0.210	0.770
AC-PO7d	anti-rsPilA	5	999	2.541	0.202	0.770
AC-5mg/kg	AC-PO7d	6	999	4.150	0.117	0.770
AC-5mg/kg	AC-10mg/kg	5	999	2.245	0.102	0.770
AC-5mg/kg	Ofloxacin	6	999	1.539	0.183	0.770
AC-5mg/kg	Saline	6	999	1.885	0.301	0.792
AC-5mg/kg	TS-30mg/kg	6	999	2.738	0.108	0.770
AC-5mg/kg	TS-15mg/kg	6	999	1.241	0.193	0.770
AC-5mg/kg	anti-tip-chimer	6	999	1.277	0.302	0.792
AC-5mg/kg	anti-rsPilA	5	999	1.430	0.214	0.770
AC-10mg/kg	AC-PO7d	5	999	0.779	0.699	0.842
AC-10mg/kg	Ofloxacin	5	999	0.703	0.702	0.842
AC-10mg/kg	Saline	5	999	0.670	0.584	0.842
AC-10mg/kg	TS-30mg/kg	5	999	1.614	0.308	0.792
AC-10mg/kg	TS-15mg/kg	5	999	0.939	0.511	0.842
AC-10mg/kg	anti-tip-chimer	5	999	0.752	0.517	0.842
AC-10mg/kg	anti-rsPilA	4	999	1.021	0.665	0.842
Ofloxacin	Saline	6	999	0.238	1.000	1.000
Ofloxacin	TS-30mg/kg	6	999	0.680	0.506	0.842
Ofloxacin	TS-15mg/kg	6	999	0.249	0.903	0.962
Ofloxacin	anti-tip-chimer	6	999	0.726	0.599	0.842
Ofloxacin	anti-rsPilA	5	999	0.400	0.909	0.962
Saline	TS-30mg/kg	6	999	0.755	0.613	0.842
Saline	TS-15mg/kg	6	999	0.590	0.800	0.929
Saline	anti-tip-chimer	6	999	1.009	0.336	0.806
Saline	anti-rsPilA	5	999	0.803	0.500	0.842
TS-30mg/kg	anti-tip-chimer	6	999	1.170	0.581	0.842
TS-30mg/kg	anti-rsPilA	5	999	1.818	0.289	0.792
TS-15mg/kg	TS-30mg/kg	6	999	0.943	0.542	0.842
TS-15mg/kg	anti-tip-chimer	6	999	0.361	0.689	0.842
TS-15mg/kg	anti-rsPilA	5	999	0.413	0.907	0.962
anti-tip-chimer	anti-rsPilA	5	999	0.330	1.000	1.000

Supplementary File 1
Beta Diversity Output

NPL Weighted UniFrac-7DPT

PERMANOVA Pairwise Comparisons (999 permutations)

Group 1	Group 2	Sample size	Permutations	pseudo-F	p-value	q-value
AC-PO7d	Ofloxacin	5	999	2.324	0.305	0.625
AC-PO7d	Saline	5	999	1.296	0.381	0.628
AC-PO7d	TS-30mg/kg	4	999	2.033	0.305	0.625
AC-PO7d	TS-15mg/kg	5	999	3.327	0.091	0.625
AC-PO7d	anti-tip-chimer	4	999	3.586	0.303	0.625
AC-PO7d	anti-rsPilA	4	999	1.832	0.333	0.625
AC-5mg/kg	AC-PO7d	5	999	7.255	0.091	0.625
AC-5mg/kg	AC-10mg/kg	4	999	4.746	0.226	0.625
AC-5mg/kg	Ofloxacin	6	999	1.596	0.309	0.625
AC-5mg/kg	Saline	6	999	2.371	0.100	0.625
AC-5mg/kg	TS-30mg/kg	5	999	6.803	0.095	0.625
AC-5mg/kg	TS-15mg/kg	6	999	1.145	0.384	0.628
AC-5mg/kg	anti-tip-chimer	5	999	0.516	0.798	0.910
AC-5mg/kg	anti-rsPilA	5	999	7.164	0.112	0.625
AC-10mg/kg	AC-PO7d	3	999	0.771	0.667	0.840
AC-10mg/kg	Ofloxacin	4	999	1.228	0.498	0.701
AC-10mg/kg	Saline	4	999	0.993	0.493	0.701
AC-10mg/kg	TS-30mg/kg	3	999	3.624	0.325	0.625
AC-10mg/kg	TS-15mg/kg	4	999	1.687	0.506	0.701
AC-10mg/kg	anti-tip-chimer	3	999	1.685	0.673	0.840
AC-10mg/kg	anti-rsPilA	3	999	2.267	0.677	0.840
Ofloxacin	Saline	6	999	0.472	0.902	0.955
Ofloxacin	TS-30mg/kg	5	999	1.468	0.310	0.625
Ofloxacin	TS-15mg/kg	6	999	0.150	1.000	1.000
Ofloxacin	anti-tip-chimer	5	999	0.536	0.891	0.955
Ofloxacin	anti-rsPilA	5	999	1.748	0.293	0.625
Saline	TS-30mg/kg	5	999	0.618	0.809	0.910
Saline	TS-15mg/kg	6	999	0.812	0.479	0.701
Saline	anti-tip-chimer	5	999	1.163	0.309	0.625
Saline	anti-rsPilA	5	999	0.523	0.788	0.910
TS-30mg/kg	anti-tip-chimer	4	999	3.005	0.342	0.625
TS-30mg/kg	anti-rsPilA	4	999	1.649	0.347	0.625
TS-15mg/kg	TS-30mg/kg	5	999	1.590	0.272	0.625
TS-15mg/kg	anti-tip-chimer	5	999	0.330	1.000	1.000
TS-15mg/kg	anti-rsPilA	5	999	2.007	0.308	0.625
anti-tip-chimer	anti-rsPilA	4	999	3.530	0.333	0.625

Supplementary File 1
Beta Diversity Output

NPL Weighted UniFrac-9DPT

PERMANOVA Pairwise Comparisons (999 permutations)

Group 1	Group 2	Sample size	Permutations	pseudo-F	p-value	q-value
AC-PO7d	Ofloxacin	4	999	1.506	0.336	0.709
AC-PO7d	Saline	4	999	3.034	0.311	0.709
AC-PO7d	TS-30mg/kg	5	999	5.315	0.107	0.566
AC-PO7d	TS-15mg/kg	5	999	5.407	0.203	0.681
AC-PO7d	anti-tip-chimer	5	999	3.733	0.208	0.681
AC-PO7d	anti-rsPilA	4	999	11.721	0.348	0.709
AC-5mg/kg	AC-PO7d	5	999	10.522	0.105	0.566
AC-5mg/kg	AC-10mg/kg	5	999	2.874	0.102	0.566
AC-5mg/kg	Ofloxacin	5	999	1.134	0.409	0.709
AC-5mg/kg	Saline	5	999	1.391	0.408	0.709
AC-5mg/kg	TS-30mg/kg	6	999	5.026	0.096	0.566
AC-5mg/kg	TS-15mg/kg	6	999	0.793	0.815	1.000
AC-5mg/kg	anti-tip-chimer	6	999	2.292	0.110	0.566
AC-5mg/kg	anti-rsPilA	5	999	0.699	0.802	1.000
AC-10mg/kg	AC-PO7d	4	999	2.660	0.323	0.709
AC-10mg/kg	Ofloxacin	4	999	0.641	1.000	1.000
AC-10mg/kg	Saline	4	999	1.323	0.316	0.709
AC-10mg/kg	TS-30mg/kg	5	999	3.277	0.081	0.566
AC-10mg/kg	TS-15mg/kg	5	999	1.853	0.188	0.681
AC-10mg/kg	anti-tip-chimer	5	999	1.165	0.599	0.881
AC-10mg/kg	anti-rsPilA	4	999	1.655	0.673	0.897
Ofloxacin	Saline	4	999	0.139	1.000	1.000
Ofloxacin	TS-30mg/kg	5	999	1.114	0.433	0.709
Ofloxacin	TS-15mg/kg	5	999	0.278	0.892	1.000
Ofloxacin	anti-tip-chimer	5	999	0.624	0.636	0.881
Ofloxacin	anti-rsPilA	4	999	0.635	1.000	1.000
Saline	TS-30mg/kg	5	999	1.122	0.420	0.709
Saline	TS-15mg/kg	5	999	0.117	0.894	1.000
Saline	anti-tip-chimer	5	999	0.742	0.614	0.881
Saline	anti-rsPilA	4	999	0.807	1.000	1.000
TS-30mg/kg	anti-tip-chimer	6	999	1.503	0.195	0.681
TS-30mg/kg	anti-rsPilA	5	999	3.729	0.102	0.566
TS-15mg/kg	TS-30mg/kg	6	999	2.048	0.292	0.709
TS-15mg/kg	anti-tip-chimer	6	999	0.900	0.415	0.709
TS-15mg/kg	anti-rsPilA	5	999	0.502	0.894	1.000
anti-tip-chimer	anti-rsPilA	5	999	0.972	0.501	0.784

Supplementary File 1
Beta Diversity Output

NPL Unweighted UniFrac-Baseline

PERMANOVA Pairwise Comparisons (999 permutations)

Group 1	Group 2	Sample size	Permutations	pseudo-F	p-value	q-value
AC-PO7d	Ofloxacin	6	999	3.548	0.101	0.476
AC-PO7d	Saline	6	999	1.747	0.095	0.476
AC-PO7d	TS-30mg/kg	6	999	1.847	0.103	0.476
AC-PO7d	TS-15mg/kg	6	999	1.531	0.107	0.476
AC-PO7d	anti-tip-chimer	6	999	2.840	0.089	0.476
AC-PO7d	anti-rsPilA	6	999	1.756	0.119	0.476
AC-5mg/kg	AC-PO7d	6	999	2.017	0.098	0.476
AC-5mg/kg	AC-10mg/kg	5	999	1.005	0.415	0.722
AC-5mg/kg	Ofloxacin	6	999	1.321	0.210	0.584
AC-5mg/kg	Saline	6	999	1.082	0.532	0.730
AC-5mg/kg	TS-30mg/kg	6	999	0.918	0.616	0.739
AC-5mg/kg	TS-15mg/kg	6	999	0.824	0.637	0.740
AC-5mg/kg	anti-tip-chimer	6	999	1.155	0.291	0.667
AC-5mg/kg	anti-rsPilA	6	999	0.911	0.502	0.729
AC-10mg/kg	AC-PO7d	5	999	2.172	0.090	0.476
AC-10mg/kg	Ofloxacin	5	999	1.416	0.211	0.584
AC-10mg/kg	Saline	5	999	0.789	0.724	0.790
AC-10mg/kg	TS-30mg/kg	5	999	1.246	0.277	0.667
AC-10mg/kg	TS-15mg/kg	5	999	0.790	0.902	0.928
AC-10mg/kg	anti-tip-chimer	5	999	1.205	0.441	0.722
AC-10mg/kg	anti-rsPilA	5	999	0.747	0.778	0.824
Ofloxacin	Saline	6	999	1.138	0.315	0.667
Ofloxacin	TS-30mg/kg	6	999	1.171	0.408	0.722
Ofloxacin	TS-15mg/kg	6	999	2.072	0.099	0.476
Ofloxacin	anti-tip-chimer	6	999	1.346	0.191	0.584
Ofloxacin	anti-rsPilA	6	999	1.073	0.506	0.729
Saline	TS-30mg/kg	6	999	0.859	0.693	0.780
Saline	TS-15mg/kg	6	999	1.141	0.399	0.722
Saline	anti-tip-chimer	6	999	1.236	0.204	0.584
Saline	anti-rsPilA	6	999	0.722	1.000	1.000
TS-30mg/kg	anti-tip-chimer	6	999	0.997	0.588	0.730
TS-30mg/kg	anti-rsPilA	6	999	0.761	0.587	0.730
TS-15mg/kg	TS-30mg/kg	6	999	1.274	0.303	0.667
TS-15mg/kg	anti-tip-chimer	6	999	1.184	0.425	0.722
TS-15mg/kg	anti-rsPilA	6	999	0.841	0.587	0.730
anti-tip-chimer	anti-rsPilA	6	999	0.989	0.482	0.729

Supplementary File 1
Beta Diversity Output

NPL Unweighted UniFrac-2DPT

PERMANOVA Pairwise Comparisons (999 permutations)

Group 1	Group 2	Sample size	Permutations	pseudo-F	p-value	q-value
AC-PO7d	Ofloxacin	6	999	2.389	0.082	0.612
AC-PO7d	Saline	6	999	3.363	0.119	0.612
AC-PO7d	TS-30mg/kg	6	999	2.047	0.103	0.612
AC-PO7d	TS-15mg/kg	6	999	1.847	0.092	0.612
AC-PO7d	anti-tip-chimer	6	999	2.692	0.109	0.612
AC-PO7d	anti-rsPilA	6	999	2.730	0.117	0.612
AC-5mg/kg	AC-PO7d	6	999	2.636	0.107	0.612
AC-5mg/kg	AC-10mg/kg	5	999	0.586	0.917	0.994
AC-5mg/kg	Ofloxacin	6	999	1.127	0.385	0.970
AC-5mg/kg	Saline	6	999	0.668	0.690	0.994
AC-5mg/kg	TS-30mg/kg	6	999	1.163	0.327	0.970
AC-5mg/kg	TS-15mg/kg	6	999	0.584	0.939	0.994
AC-5mg/kg	anti-tip-chimer	6	999	0.801	0.527	0.994
AC-5mg/kg	anti-rsPilA	6	999	0.695	0.934	0.994
AC-10mg/kg	AC-PO7d	5	999	1.891	0.208	0.880
AC-10mg/kg	Ofloxacin	5	999	1.045	0.404	0.970
AC-10mg/kg	Saline	5	999	0.828	0.899	0.994
AC-10mg/kg	TS-30mg/kg	5	999	1.069	0.383	0.970
AC-10mg/kg	TS-15mg/kg	5	999	0.473	0.898	0.994
AC-10mg/kg	anti-tip-chimer	5	999	0.642	0.720	0.994
AC-10mg/kg	anti-rsPilA	5	999	0.635	0.698	0.994
Ofloxacin	Saline	6	999	1.286	0.314	0.970
Ofloxacin	TS-30mg/kg	6	999	1.206	0.284	0.970
Ofloxacin	TS-15mg/kg	6	999	0.795	0.582	0.994
Ofloxacin	anti-tip-chimer	6	999	0.701	0.802	0.994
Ofloxacin	anti-rsPilA	6	999	1.001	0.507	0.994
Saline	TS-30mg/kg	6	999	1.172	0.501	0.994
Saline	TS-15mg/kg	6	999	0.793	1.000	1.000
Saline	anti-tip-chimer	6	999	0.950	0.522	0.994
Saline	anti-rsPilA	6	999	0.612	0.695	0.994
TS-30mg/kg	anti-tip-chimer	6	999	1.470	0.220	0.880
TS-30mg/kg	anti-rsPilA	6	999	0.665	0.784	0.994
TS-15mg/kg	TS-30mg/kg	6	999	0.750	1.000	1.000
TS-15mg/kg	anti-tip-chimer	6	999	0.620	0.790	0.994
TS-15mg/kg	anti-rsPilA	6	999	0.605	0.898	0.994
anti-tip-chimer	anti-rsPilA	6	999	0.921	0.612	0.994

Supplementary File 1
Beta Diversity Output

NPL Unweighted UniFrac-5DPT

PERMANOVA Pairwise Comparisons (999 permutations)

Group 1	Group 2	Sample size	Permutations	pseudo-F	p-value	q-value
AC-PO7d	Ofloxacin	6	999	1.449	0.086	0.558
AC-PO7d	Saline	6	999	1.194	0.293	0.718
AC-PO7d	TS-30mg/kg	6	999	1.429	0.103	0.558
AC-PO7d	TS-15mg/kg	6	999	1.219	0.396	0.792
AC-PO7d	anti-tip-chimer	6	999	1.620	0.234	0.702
AC-PO7d	anti-rsPilA	5	999	1.325	0.299	0.718
AC-5mg/kg	AC-PO7d	6	999	2.017	0.095	0.558
AC-5mg/kg	AC-10mg/kg	5	999	1.568	0.298	0.718
AC-5mg/kg	Ofloxacin	6	999	1.482	0.115	0.558
AC-5mg/kg	Saline	6	999	0.908	0.713	0.856
AC-5mg/kg	TS-30mg/kg	6	999	1.554	0.089	0.558
AC-5mg/kg	TS-15mg/kg	6	999	0.936	0.601	0.832
AC-5mg/kg	anti-tip-chimer	6	999	1.017	0.388	0.792
AC-5mg/kg	anti-rsPilA	5	999	0.839	0.571	0.832
AC-10mg/kg	AC-PO7d	5	999	1.825	0.201	0.691
AC-10mg/kg	Ofloxacin	5	999	1.937	0.101	0.558
AC-10mg/kg	Saline	5	999	1.158	0.200	0.691
AC-10mg/kg	TS-30mg/kg	5	999	1.645	0.211	0.691
AC-10mg/kg	TS-15mg/kg	5	999	0.729	0.882	0.923
AC-10mg/kg	anti-tip-chimer	5	999	1.661	0.118	0.558
AC-10mg/kg	anti-rsPilA	4	999	2.547	0.354	0.792
Ofloxacin	Saline	6	999	1.245	0.124	0.558
Ofloxacin	TS-30mg/kg	6	999	0.878	0.696	0.856
Ofloxacin	TS-15mg/kg	6	999	0.747	0.813	0.887
Ofloxacin	anti-tip-chimer	6	999	1.232	0.424	0.803
Ofloxacin	anti-rsPilA	5	999	0.868	0.897	0.923
Saline	TS-30mg/kg	6	999	0.981	0.577	0.832
Saline	TS-15mg/kg	6	999	0.778	0.578	0.832
Saline	anti-tip-chimer	6	999	0.993	0.490	0.832
Saline	anti-rsPilA	5	999	0.528	1.000	1.000
TS-30mg/kg	anti-tip-chimer	6	999	0.849	0.705	0.856
TS-30mg/kg	anti-rsPilA	5	999	0.951	0.510	0.832
TS-15mg/kg	TS-30mg/kg	6	999	0.702	0.694	0.856
TS-15mg/kg	anti-tip-chimer	6	999	0.723	0.794	0.887
TS-15mg/kg	anti-rsPilA	5	999	0.872	0.507	0.832
anti-tip-chimer	anti-rsPilA	5	999	0.771	0.785	0.887

Supplementary File 1
Beta Diversity Output

NPL Unweighted UniFrac-7DPT

PERMANOVA Pairwise Comparisons (999 permutations)

Group 1	Group 2	Sample size	Permutations	pseudo-F	p-value	q-value
AC-PO7d	Ofloxacin	5	999	1.977	0.097	0.623
AC-PO7d	Saline	5	999	1.707	0.090	0.623
AC-PO7d	TS-30mg/kg	4	999	2.289	0.360	0.648
AC-PO7d	TS-15mg/kg	5	999	1.611	0.093	0.623
AC-PO7d	anti-tip-chimer	4	999	2.577	0.311	0.623
AC-PO7d	anti-rsPilA	4	999	2.859	0.327	0.623
AC-5mg/kg	AC-PO7d	5	999	2.347	0.093	0.623
AC-5mg/kg	AC-10mg/kg	4	999	1.164	0.758	0.897
AC-5mg/kg	Ofloxacin	6	999	1.012	0.574	0.861
AC-5mg/kg	Saline	6	999	1.134	0.305	0.623
AC-5mg/kg	TS-30mg/kg	5	999	2.348	0.092	0.623
AC-5mg/kg	TS-15mg/kg	6	999	1.496	0.205	0.623
AC-5mg/kg	anti-tip-chimer	5	999	1.654	0.112	0.623
AC-5mg/kg	anti-rsPilA	5	999	2.326	0.182	0.623
AC-10mg/kg	AC-PO7d	3	999	1.776	0.329	0.623
AC-10mg/kg	Ofloxacin	4	999	0.447	1.000	1.000
AC-10mg/kg	Saline	4	999	0.397	1.000	1.000
AC-10mg/kg	TS-30mg/kg	3	999	1.069	0.675	0.870
AC-10mg/kg	TS-15mg/kg	4	999	0.676	0.732	0.897
AC-10mg/kg	anti-tip-chimer	3	999	0.974	0.665	0.870
AC-10mg/kg	anti-rsPilA	3	999	1.900	0.327	0.623
Ofloxacin	Saline	6	999	0.658	0.898	0.980
Ofloxacin	TS-30mg/kg	5	999	1.543	0.299	0.623
Ofloxacin	TS-15mg/kg	6	999	0.544	1.000	1.000
Ofloxacin	anti-tip-chimer	5	999	1.194	0.427	0.668
Ofloxacin	anti-rsPilA	5	999	1.835	0.186	0.623
Saline	TS-30mg/kg	5	999	1.222	0.285	0.623
Saline	TS-15mg/kg	6	999	0.778	0.808	0.909
Saline	anti-tip-chimer	5	999	0.743	0.772	0.897
Saline	anti-rsPilA	5	999	1.247	0.302	0.623
TS-30mg/kg	anti-tip-chimer	4	999	2.270	0.323	0.623
TS-30mg/kg	anti-rsPilA	4	999	1.063	0.664	0.870
TS-15mg/kg	TS-30mg/kg	5	999	1.096	0.400	0.655
TS-15mg/kg	anti-tip-chimer	5	999	0.991	0.677	0.870
TS-15mg/kg	anti-rsPilA	5	999	0.932	0.389	0.655
anti-tip-chimer	anti-rsPilA	4	999	2.536	0.314	0.623

Supplementary File 1
Beta Diversity Output

NPL Unweighted UniFrac-9DPT

PERMANOVA Pairwise Comparisons (999 permutations)

Group 1	Group 2	Sample size	Permutations	pseudo-F	p-value	q-value
AC-PO7d	Ofloxacin	4	999	4.304	0.347	0.591
AC-PO7d	Saline	4	999	5.410	0.335	0.591
AC-PO7d	TS-30mg/kg	5	999	2.480	0.105	0.571
AC-PO7d	TS-15mg/kg	5	999	1.995	0.190	0.591
AC-PO7d	anti-tip-chimer	5	999	3.149	0.105	0.571
AC-PO7d	anti-rsPilA	4	999	4.731	0.337	0.591
AC-5mg/kg	AC-PO7d	5	999	3.838	0.108	0.571
AC-5mg/kg	AC-10mg/kg	5	999	1.802	0.111	0.571
AC-5mg/kg	Ofloxacin	5	999	1.495	0.209	0.591
AC-5mg/kg	Saline	5	999	1.243	0.296	0.591
AC-5mg/kg	TS-30mg/kg	6	999	0.988	0.592	0.742
AC-5mg/kg	TS-15mg/kg	6	999	0.997	0.483	0.674
AC-5mg/kg	anti-tip-chimer	6	999	1.406	0.097	0.571
AC-5mg/kg	anti-rsPilA	5	999	0.914	0.581	0.742
AC-10mg/kg	AC-PO7d	4	999	3.058	0.304	0.591
AC-10mg/kg	Ofloxacin	4	999	1.581	0.323	0.591
AC-10mg/kg	Saline	4	999	1.969	0.328	0.591
AC-10mg/kg	TS-30mg/kg	5	999	1.070	0.472	0.674
AC-10mg/kg	TS-15mg/kg	5	999	0.875	0.716	0.758
AC-10mg/kg	anti-tip-chimer	5	999	1.312	0.212	0.591
AC-10mg/kg	anti-rsPilA	4	999	2.166	0.341	0.591
Ofloxacin	Saline	4	999	1.601	0.361	0.591
Ofloxacin	TS-30mg/kg	5	999	1.136	0.487	0.674
Ofloxacin	TS-15mg/kg	5	999	0.874	0.707	0.758
Ofloxacin	anti-tip-chimer	5	999	1.802	0.096	0.571
Ofloxacin	anti-rsPilA	4	999	1.755	0.335	0.591
Saline	TS-30mg/kg	5	999	0.877	0.603	0.742
Saline	TS-15mg/kg	5	999	0.862	0.708	0.758
Saline	anti-tip-chimer	5	999	1.322	0.200	0.591
Saline	anti-rsPilA	4	999	1.362	0.328	0.591
TS-30mg/kg	anti-tip-chimer	6	999	0.755	0.899	0.899
TS-30mg/kg	anti-rsPilA	5	999	1.082	0.398	0.623
TS-15mg/kg	TS-30mg/kg	6	999	0.758	0.685	0.758
TS-15mg/kg	anti-tip-chimer	6	999	0.599	0.894	0.899
TS-15mg/kg	anti-rsPilA	5	999	0.919	0.618	0.742
anti-tip-chimer	anti-rsPilA	5	999	1.729	0.111	0.571

Supplementary File 1
Beta Diversity Output

NPL Bray-Curtis-Baseline

PERMANOVA Pairwise Comparisons (999 permutations)

Group 1	Group 2	Sample size	Permutations	pseudo-F	p-value	q-value
AC-PO7d	Ofloxacin	6	999	2.651	0.084	0.290
AC-PO7d	Saline	6	999	2.144	0.108	0.290
AC-PO7d	TS-30mg/kg	6	999	2.044	0.096	0.290
AC-PO7d	TS-15mg/kg	6	999	2.150	0.110	0.290
AC-PO7d	anti-tip-chimer	6	999	2.646	0.121	0.290
AC-PO7d	anti-rsPilA	6	999	2.305	0.104	0.290
AC-5mg/kg	AC-PO7d	6	999	3.703	0.104	0.290
AC-5mg/kg	AC-10mg/kg	5	999	0.666	0.907	1.000
AC-5mg/kg	Ofloxacin	6	999	1.157	0.301	0.637
AC-5mg/kg	Saline	6	999	0.770	0.900	1.000
AC-5mg/kg	TS-30mg/kg	6	999	3.073	0.092	0.290
AC-5mg/kg	TS-15mg/kg	6	999	0.731	1.000	1.000
AC-5mg/kg	anti-tip-chimer	6	999	1.239	0.207	0.466
AC-5mg/kg	anti-rsPilA	6	999	0.901	0.701	1.000
AC-10mg/kg	AC-PO7d	5	999	2.161	0.097	0.290
AC-10mg/kg	Ofloxacin	5	999	0.820	0.812	1.000
AC-10mg/kg	Saline	5	999	0.625	1.000	1.000
AC-10mg/kg	TS-30mg/kg	5	999	1.912	0.099	0.290
AC-10mg/kg	TS-15mg/kg	5	999	0.532	1.000	1.000
AC-10mg/kg	anti-tip-chimer	5	999	0.622	1.000	1.000
AC-10mg/kg	anti-rsPilA	5	999	0.662	0.884	1.000
Ofloxacin	Saline	6	999	0.860	0.590	1.000
Ofloxacin	TS-30mg/kg	6	999	1.720	0.105	0.290
Ofloxacin	TS-15mg/kg	6	999	0.745	0.790	1.000
Ofloxacin	anti-tip-chimer	6	999	0.764	0.522	0.940
Ofloxacin	anti-rsPilA	6	999	0.908	0.491	0.930
Saline	TS-30mg/kg	6	999	1.754	0.077	0.290
Saline	TS-15mg/kg	6	999	0.381	1.000	1.000
Saline	anti-tip-chimer	6	999	0.910	0.451	0.902
Saline	anti-rsPilA	6	999	0.638	1.000	1.000
TS-30mg/kg	anti-tip-chimer	6	999	1.836	0.119	0.290
TS-30mg/kg	anti-rsPilA	6	999	1.937	0.109	0.290
TS-15mg/kg	TS-30mg/kg	6	999	1.844	0.099	0.290
TS-15mg/kg	anti-tip-chimer	6	999	0.804	0.701	1.000
TS-15mg/kg	anti-rsPilA	6	999	0.567	1.000	1.000
anti-tip-chimer	anti-rsPilA	6	999	0.865	0.701	1.000

Supplementary File 1
Beta Diversity Output

NPL Bray-Curtis-2DPT

PERMANOVA Pairwise Comparisons (999 permutations)

Group 1	Group 2	Sample size	Permutations	pseudo-F	p-value	q-value
AC-PO7d	Ofloxacin	6	999	4.769	0.109	0.261
AC-PO7d	Saline	6	999	5.672	0.101	0.261
AC-PO7d	TS-30mg/kg	6	999	5.087	0.094	0.261
AC-PO7d	TS-15mg/kg	6	999	4.460	0.103	0.261
AC-PO7d	anti-tip-chimer	6	999	5.819	0.103	0.261
AC-PO7d	anti-rsPilA	6	999	7.094	0.091	0.261
AC-5mg/kg	AC-PO7d	6	999	8.551	0.098	0.261
AC-5mg/kg	AC-10mg/kg	5	999	2.489	0.116	0.261
AC-5mg/kg	Ofloxacin	6	999	0.954	0.586	0.742
AC-5mg/kg	Saline	6	999	1.120	0.408	0.641
AC-5mg/kg	TS-30mg/kg	6	999	3.861	0.083	0.261
AC-5mg/kg	TS-15mg/kg	6	999	1.151	0.307	0.528
AC-5mg/kg	anti-tip-chimer	6	999	1.282	0.301	0.528
AC-5mg/kg	anti-rsPilA	6	999	1.152	0.299	0.528
AC-10mg/kg	AC-PO7d	5	999	6.354	0.104	0.261
AC-10mg/kg	Ofloxacin	5	999	1.148	0.308	0.528
AC-10mg/kg	Saline	5	999	1.558	0.112	0.261
AC-10mg/kg	TS-30mg/kg	5	999	2.300	0.112	0.261
AC-10mg/kg	TS-15mg/kg	5	999	1.012	0.422	0.641
AC-10mg/kg	anti-tip-chimer	5	999	0.900	0.606	0.742
AC-10mg/kg	anti-rsPilA	5	999	1.177	0.427	0.641
Ofloxacin	Saline	6	999	0.476	0.794	0.821
Ofloxacin	TS-30mg/kg	6	999	1.672	0.097	0.261
Ofloxacin	TS-15mg/kg	6	999	0.528	1.000	1.000
Ofloxacin	anti-tip-chimer	6	999	0.506	0.798	0.821
Ofloxacin	anti-rsPilA	6	999	0.820	0.598	0.742
Saline	TS-30mg/kg	6	999	2.371	0.097	0.261
Saline	TS-15mg/kg	6	999	0.692	0.794	0.821
Saline	anti-tip-chimer	6	999	0.886	0.518	0.742
Saline	anti-rsPilA	6	999	0.991	0.618	0.742
TS-30mg/kg	anti-tip-chimer	6	999	2.117	0.095	0.261
TS-30mg/kg	anti-rsPilA	6	999	2.512	0.112	0.261
TS-15mg/kg	TS-30mg/kg	6	999	1.512	0.130	0.275
TS-15mg/kg	anti-tip-chimer	6	999	0.743	0.734	0.821
TS-15mg/kg	anti-rsPilA	6	999	0.791	0.788	0.821
anti-tip-chimer	anti-rsPilA	6	999	0.956	0.596	0.742

Supplementary File 1
Beta Diversity Output

NPL Bray-Curtis-5DPT

PERMANOVA Pairwise Comparisons (999 permutations)

Group 1	Group 2	Sample size	Permutations	pseudo-F	p-value	q-value
AC-PO7d	Ofloxacin	6	999	1.630	0.109	0.484
AC-PO7d	Saline	6	999	1.455	0.203	0.526
AC-PO7d	TS-30mg/kg	6	999	2.116	0.087	0.484
AC-PO7d	TS-15mg/kg	6	999	2.238	0.083	0.484
AC-PO7d	anti-tip-chimer	6	999	2.237	0.093	0.484
AC-PO7d	anti-rsPilA	5	999	2.158	0.099	0.484
AC-5mg/kg	AC-PO7d	6	999	2.524	0.082	0.484
AC-5mg/kg	AC-10mg/kg	5	999	1.131	0.426	0.852
AC-5mg/kg	Ofloxacin	6	999	0.893	0.798	0.962
AC-5mg/kg	Saline	6	999	1.146	0.394	0.852
AC-5mg/kg	TS-30mg/kg	6	999	2.277	0.217	0.526
AC-5mg/kg	TS-15mg/kg	6	999	0.882	0.695	0.962
AC-5mg/kg	anti-tip-chimer	6	999	0.792	0.900	0.962
AC-5mg/kg	anti-rsPilA	5	999	0.802	0.806	0.962
AC-10mg/kg	AC-PO7d	5	999	1.554	0.219	0.526
AC-10mg/kg	Ofloxacin	5	999	0.957	0.610	0.962
AC-10mg/kg	Saline	5	999	0.894	0.601	0.962
AC-10mg/kg	TS-30mg/kg	5	999	1.934	0.099	0.484
AC-10mg/kg	TS-15mg/kg	5	999	1.044	0.405	0.852
AC-10mg/kg	anti-tip-chimer	5	999	0.586	1.000	1.000
AC-10mg/kg	anti-rsPilA	4	999	1.008	0.655	0.962
Ofloxacin	Saline	6	999	0.773	0.677	0.962
Ofloxacin	TS-30mg/kg	6	999	1.462	0.203	0.526
Ofloxacin	TS-15mg/kg	6	999	0.435	1.000	1.000
Ofloxacin	anti-tip-chimer	6	999	0.804	0.597	0.962
Ofloxacin	anti-rsPilA	5	999	0.663	0.909	0.962
Saline	TS-30mg/kg	6	999	1.356	0.207	0.526
Saline	TS-15mg/kg	6	999	0.783	0.909	0.962
Saline	anti-tip-chimer	6	999	0.820	0.806	0.962
Saline	anti-rsPilA	5	999	0.823	0.723	0.962
TS-30mg/kg	anti-tip-chimer	6	999	1.633	0.098	0.484
TS-30mg/kg	anti-rsPilA	5	999	2.299	0.204	0.526
TS-15mg/kg	TS-30mg/kg	6	999	1.629	0.121	0.484
TS-15mg/kg	anti-tip-chimer	6	999	0.675	0.794	0.962
TS-15mg/kg	anti-rsPilA	5	999	0.668	0.905	0.962
anti-tip-chimer	anti-rsPilA	5	999	0.661	0.803	0.962

Supplementary File 1
Beta Diversity Output

NPL Bray-Curtis-7DPT

PERMANOVA Pairwise Comparisons (999 permutations)

Group 1	Group 2	Sample size	Permutations	pseudo-F	p-value	q-value
AC-PO7d	Ofloxacin	5	999	1.823	0.192	0.583
AC-PO7d	Saline	5	999	1.479	0.094	0.583
AC-PO7d	TS-30mg/kg	4	999	1.267	0.320	0.583
AC-PO7d	TS-15mg/kg	5	999	2.210	0.095	0.583
AC-PO7d	anti-tip-chimer	4	999	2.171	0.340	0.583
AC-PO7d	anti-rsPilA	4	999	2.319	0.329	0.583
AC-5mg/kg	AC-PO7d	5	999	3.094	0.101	0.583
AC-5mg/kg	AC-10mg/kg	4	999	1.732	0.213	0.583
AC-5mg/kg	Ofloxacin	6	999	0.780	0.915	0.998
AC-5mg/kg	Saline	6	999	1.347	0.301	0.583
AC-5mg/kg	TS-30mg/kg	5	999	2.344	0.119	0.583
AC-5mg/kg	TS-15mg/kg	6	999	1.055	0.396	0.620
AC-5mg/kg	anti-tip-chimer	5	999	0.544	0.782	0.908
AC-5mg/kg	anti-rsPilA	5	999	3.364	0.095	0.583
AC-10mg/kg	AC-PO7d	3	999	1.442	0.279	0.583
AC-10mg/kg	Ofloxacin	4	999	0.885	0.485	0.710
AC-10mg/kg	Saline	4	999	0.945	0.508	0.710
AC-10mg/kg	TS-30mg/kg	3	999	1.277	0.320	0.583
AC-10mg/kg	TS-15mg/kg	4	999	1.188	0.513	0.710
AC-10mg/kg	anti-tip-chimer	3	999	1.178	0.672	0.852
AC-10mg/kg	anti-rsPilA	3	999	2.218	0.313	0.583
Ofloxacin	Saline	6	999	0.709	0.893	0.998
Ofloxacin	TS-30mg/kg	5	999	1.240	0.380	0.620
Ofloxacin	TS-15mg/kg	6	999	0.497	1.000	1.000
Ofloxacin	anti-tip-chimer	5	999	0.470	1.000	1.000
Ofloxacin	anti-rsPilA	5	999	1.438	0.307	0.583
Saline	TS-30mg/kg	5	999	0.969	0.599	0.799
Saline	TS-15mg/kg	6	999	0.876	0.698	0.852
Saline	anti-tip-chimer	5	999	0.835	0.710	0.852
Saline	anti-rsPilA	5	999	1.419	0.194	0.583
TS-30mg/kg	anti-tip-chimer	4	999	1.346	0.333	0.583
TS-30mg/kg	anti-rsPilA	4	999	1.882	0.320	0.583
TS-15mg/kg	TS-30mg/kg	5	999	1.320	0.284	0.583
TS-15mg/kg	anti-tip-chimer	5	999	0.571	1.000	1.000
TS-15mg/kg	anti-rsPilA	5	999	2.117	0.106	0.583
anti-tip-chimer	anti-rsPilA	4	999	2.528	0.331	0.583

Supplementary File 1
Beta Diversity Output

NPL Bray-Curtis-9DPT

PERMANOVA Pairwise Comparisons (999 permutations)

Group 1	Group 2	Sample size	Permutations	pseudo-F	p-value	q-value
AC-PO7d	Ofloxacin	4	999	1.180	0.328	0.657
AC-PO7d	Saline	4	999	4.277	0.336	0.657
AC-PO7d	TS-30mg/kg	5	999	3.194	0.117	0.468
AC-PO7d	TS-15mg/kg	5	999	4.018	0.093	0.468
AC-PO7d	anti-tip-chimer	5	999	3.410	0.090	0.468
AC-PO7d	anti-rsPilA	4	999	6.657	0.344	0.657
AC-5mg/kg	AC-PO7d	5	999	6.640	0.095	0.468
AC-5mg/kg	AC-10mg/kg	5	999	1.373	0.205	0.657
AC-5mg/kg	Ofloxacin	5	999	0.897	0.491	0.803
AC-5mg/kg	Saline	5	999	1.307	0.417	0.715
AC-5mg/kg	TS-30mg/kg	6	999	2.276	0.103	0.468
AC-5mg/kg	TS-15mg/kg	6	999	0.763	1.000	1.000
AC-5mg/kg	anti-tip-chimer	6	999	1.285	0.310	0.657
AC-5mg/kg	anti-rsPilA	5	999	1.162	0.282	0.657
AC-10mg/kg	AC-PO7d	4	999	2.282	0.348	0.657
AC-10mg/kg	Ofloxacin	4	999	0.449	1.000	1.000
AC-10mg/kg	Saline	4	999	1.491	0.338	0.657
AC-10mg/kg	TS-30mg/kg	5	999	1.986	0.094	0.468
AC-10mg/kg	TS-15mg/kg	5	999	1.412	0.109	0.468
AC-10mg/kg	anti-tip-chimer	5	999	0.866	0.710	0.913
AC-10mg/kg	anti-rsPilA	4	999	1.097	0.653	0.913
Ofloxacin	Saline	4	999	0.460	1.000	1.000
Ofloxacin	TS-30mg/kg	5	999	0.997	0.596	0.894
Ofloxacin	TS-15mg/kg	5	999	0.579	1.000	1.000
Ofloxacin	anti-tip-chimer	5	999	0.586	0.787	0.977
Ofloxacin	anti-rsPilA	4	999	0.535	1.000	1.000
Saline	TS-30mg/kg	5	999	1.322	0.208	0.657
Saline	TS-15mg/kg	5	999	0.719	0.819	0.983
Saline	anti-tip-chimer	5	999	0.961	0.365	0.657
Saline	anti-rsPilA	4	999	0.971	0.667	0.913
TS-30mg/kg	anti-tip-chimer	6	999	1.404	0.099	0.468
TS-30mg/kg	anti-rsPilA	5	999	2.187	0.110	0.468
TS-15mg/kg	TS-30mg/kg	6	999	1.204	0.304	0.657
TS-15mg/kg	anti-tip-chimer	6	999	0.810	0.699	0.913
TS-15mg/kg	anti-rsPilA	5	999	0.971	0.591	0.894
anti-tip-chimer	anti-rsPilA	5	999	0.762	0.900	1.000

Supplementary File 1
Beta Diversity Output

NPL Jaccard-Baseline

PERMANOVA Pairwise Comparisons (999 permutations)

Group 1	Group 2	Sample size	Permutations	pseudo-F	p-value	q-value
AC-PO7d	Ofloxacin	6	999	2.638	0.094	0.370
AC-PO7d	Saline	6	999	2.340	0.099	0.370
AC-PO7d	TS-30mg/kg	6	999	1.968	0.091	0.370
AC-PO7d	TS-15mg/kg	6	999	2.258	0.092	0.370
AC-PO7d	anti-tip-chimer	6	999	2.783	0.111	0.370
AC-PO7d	anti-rsPilA	6	999	2.332	0.087	0.370
AC-5mg/kg	AC-PO7d	6	999	2.696	0.100	0.370
AC-5mg/kg	AC-10mg/kg	5	999	0.700	0.910	1.000
AC-5mg/kg	Ofloxacin	6	999	0.805	1.000	1.000
AC-5mg/kg	Saline	6	999	0.960	0.707	1.000
AC-5mg/kg	TS-30mg/kg	6	999	1.172	0.203	0.573
AC-5mg/kg	TS-15mg/kg	6	999	0.850	1.000	1.000
AC-5mg/kg	anti-tip-chimer	6	999	0.938	0.610	0.955
AC-5mg/kg	anti-rsPilA	6	999	0.661	1.000	1.000
AC-10mg/kg	AC-PO7d	5	999	2.200	0.113	0.370
AC-10mg/kg	Ofloxacin	5	999	0.803	1.000	1.000
AC-10mg/kg	Saline	5	999	0.876	0.908	1.000
AC-10mg/kg	TS-30mg/kg	5	999	1.209	0.207	0.573
AC-10mg/kg	TS-15mg/kg	5	999	0.609	1.000	1.000
AC-10mg/kg	anti-tip-chimer	5	999	0.856	0.904	1.000
AC-10mg/kg	anti-rsPilA	5	999	0.732	0.903	1.000
Ofloxacin	Saline	6	999	1.086	0.411	0.925
Ofloxacin	TS-30mg/kg	6	999	1.125	0.304	0.749
Ofloxacin	TS-15mg/kg	6	999	0.871	0.794	1.000
Ofloxacin	anti-tip-chimer	6	999	0.945	0.582	0.955
Ofloxacin	anti-rsPilA	6	999	0.947	0.603	0.955
Saline	TS-30mg/kg	6	999	1.303	0.091	0.370
Saline	TS-15mg/kg	6	999	0.802	0.780	1.000
Saline	anti-tip-chimer	6	999	1.348	0.106	0.370
Saline	anti-rsPilA	6	999	0.922	0.579	0.955
TS-30mg/kg	anti-tip-chimer	6	999	1.216	0.102	0.370
TS-30mg/kg	anti-rsPilA	6	999	1.064	0.437	0.925
TS-15mg/kg	TS-30mg/kg	6	999	1.182	0.312	0.749
TS-15mg/kg	anti-tip-chimer	6	999	1.013	0.607	0.955
TS-15mg/kg	anti-rsPilA	6	999	0.835	0.892	1.000
anti-tip-chimer	anti-rsPilA	6	999	0.975	0.516	0.955

Supplementary File 1
Beta Diversity Output

NPL Jaccard-2DPT

PERMANOVA Pairwise Comparisons (999 permutations)

Group 1	Group 2	Sample size	Permutations	pseudo-F	p-value	q-value
AC-PO7d	Ofloxacin	6	999	3.498	0.113	0.370
AC-PO7d	Saline	6	999	4.211	0.102	0.370
AC-PO7d	TS-30mg/kg	6	999	3.227	0.104	0.370
AC-PO7d	TS-15mg/kg	6	999	3.086	0.088	0.370
AC-PO7d	anti-tip-chimer	6	999	4.403	0.111	0.370
AC-PO7d	anti-rsPilA	6	999	3.941	0.088	0.370
AC-5mg/kg	AC-PO7d	6	999	3.547	0.105	0.370
AC-5mg/kg	AC-10mg/kg	5	999	1.024	0.517	0.809
AC-5mg/kg	Ofloxacin	6	999	0.800	0.775	0.893
AC-5mg/kg	Saline	6	999	0.956	0.689	0.886
AC-5mg/kg	TS-30mg/kg	6	999	1.320	0.199	0.516
AC-5mg/kg	TS-15mg/kg	6	999	0.881	0.678	0.886
AC-5mg/kg	anti-tip-chimer	6	999	0.764	1.000	1.000
AC-5mg/kg	anti-rsPilA	6	999	0.660	1.000	1.000
AC-10mg/kg	AC-PO7d	5	999	4.202	0.099	0.370
AC-10mg/kg	Ofloxacin	5	999	0.968	0.511	0.809
AC-10mg/kg	Saline	5	999	1.476	0.189	0.516
AC-10mg/kg	TS-30mg/kg	5	999	1.624	0.101	0.370
AC-10mg/kg	TS-15mg/kg	5	999	0.906	0.794	0.893
AC-10mg/kg	anti-tip-chimer	5	999	1.079	0.285	0.623
AC-10mg/kg	anti-rsPilA	5	999	1.063	0.483	0.809
Ofloxacin	Saline	6	999	0.980	0.592	0.852
Ofloxacin	TS-30mg/kg	6	999	1.050	0.368	0.718
Ofloxacin	TS-15mg/kg	6	999	0.658	1.000	1.000
Ofloxacin	anti-tip-chimer	6	999	0.824	0.784	0.893
Ofloxacin	anti-rsPilA	6	999	0.834	0.790	0.893
Saline	TS-30mg/kg	6	999	1.659	0.100	0.370
Saline	TS-15mg/kg	6	999	0.981	0.516	0.809
Saline	anti-tip-chimer	6	999	1.256	0.212	0.516
Saline	anti-rsPilA	6	999	1.092	0.215	0.516
TS-30mg/kg	anti-tip-chimer	6	999	1.529	0.082	0.370
TS-30mg/kg	anti-rsPilA	6	999	1.095	0.294	0.623
TS-15mg/kg	TS-30mg/kg	6	999	1.095	0.379	0.718
TS-15mg/kg	anti-tip-chimer	6	999	0.917	0.583	0.852
TS-15mg/kg	anti-rsPilA	6	999	0.853	0.895	0.976
anti-tip-chimer	anti-rsPilA	6	999	0.976	0.627	0.868

Supplementary File 1
Beta Diversity Output

NPL Jaccard-5DPT

PERMANOVA Pairwise Comparisons (999 permutations)

Group 1	Group 2	Sample size	Permutations	pseudo-F	p-value	q-value
AC-PO7d	Ofloxacin	6	999	1.603	0.095	0.436
AC-PO7d	Saline	6	999	1.791	0.121	0.436
AC-PO7d	TS-30mg/kg	6	999	2.224	0.093	0.436
AC-PO7d	TS-15mg/kg	6	999	2.048	0.101	0.436
AC-PO7d	anti-tip-chimer	6	999	2.195	0.096	0.436
AC-PO7d	anti-rsPilA	5	999	1.978	0.087	0.436
AC-5mg/kg	AC-PO7d	6	999	2.202	0.104	0.436
AC-5mg/kg	AC-10mg/kg	5	999	1.051	0.294	0.634
AC-5mg/kg	Ofloxacin	6	999	0.868	0.803	0.903
AC-5mg/kg	Saline	6	999	0.958	0.676	0.895
AC-5mg/kg	TS-30mg/kg	6	999	1.485	0.217	0.601
AC-5mg/kg	TS-15mg/kg	6	999	0.896	0.798	0.903
AC-5mg/kg	anti-tip-chimer	6	999	0.700	0.888	0.925
AC-5mg/kg	anti-rsPilA	5	999	0.801	0.680	0.895
AC-10mg/kg	AC-PO7d	5	999	1.931	0.113	0.436
AC-10mg/kg	Ofloxacin	5	999	0.994	0.623	0.895
AC-10mg/kg	Saline	5	999	1.177	0.317	0.634
AC-10mg/kg	TS-30mg/kg	5	999	1.640	0.102	0.436
AC-10mg/kg	TS-15mg/kg	5	999	1.081	0.307	0.634
AC-10mg/kg	anti-tip-chimer	5	999	0.898	0.714	0.895
AC-10mg/kg	anti-rsPilA	4	999	1.152	0.339	0.642
Ofloxacin	Saline	6	999	1.035	0.393	0.707
Ofloxacin	TS-30mg/kg	6	999	1.199	0.282	0.634
Ofloxacin	TS-15mg/kg	6	999	0.662	0.899	0.925
Ofloxacin	anti-tip-chimer	6	999	0.954	0.618	0.895
Ofloxacin	anti-rsPilA	5	999	0.837	0.887	0.925
Saline	TS-30mg/kg	6	999	1.423	0.206	0.601
Saline	TS-15mg/kg	6	999	0.958	0.677	0.895
Saline	anti-tip-chimer	6	999	0.962	0.596	0.895
Saline	anti-rsPilA	5	999	0.767	1.000	1.000
TS-30mg/kg	anti-tip-chimer	6	999	1.244	0.102	0.436
TS-30mg/kg	anti-rsPilA	5	999	1.496	0.207	0.601
TS-15mg/kg	TS-30mg/kg	6	999	1.220	0.317	0.634
TS-15mg/kg	anti-tip-chimer	6	999	0.868	0.676	0.895
TS-15mg/kg	anti-rsPilA	5	999	0.829	0.721	0.895
anti-tip-chimer	anti-rsPilA	5	999	0.863	0.802	0.903

Supplementary File 1
Beta Diversity Output

NPL Jaccard-7DPT

PERMANOVA Pairwise Comparisons (999 permutations)

Group 1	Group 2	Sample size	Permutations	pseudo-F	p-value	q-value
AC-PO7d	Ofloxacin	5	999	2.435	0.099	0.566
AC-PO7d	Saline	5	999	2.331	0.106	0.566
AC-PO7d	TS-30mg/kg	4	999	2.083	0.334	0.566
AC-PO7d	TS-15mg/kg	5	999	2.405	0.106	0.566
AC-PO7d	anti-tip-chimer	4	999	2.948	0.289	0.566
AC-PO7d	anti-rsPilA	4	999	2.577	0.346	0.566
AC-5mg/kg	AC-PO7d	5	999	2.680	0.106	0.566
AC-5mg/kg	AC-10mg/kg	4	999	1.406	0.269	0.566
AC-5mg/kg	Ofloxacin	6	999	0.734	1.000	1.000
AC-5mg/kg	Saline	6	999	1.135	0.437	0.629
AC-5mg/kg	TS-30mg/kg	5	999	1.484	0.101	0.566
AC-5mg/kg	TS-15mg/kg	6	999	1.089	0.295	0.566
AC-5mg/kg	anti-tip-chimer	5	999	0.733	1.000	1.000
AC-5mg/kg	anti-rsPilA	5	999	1.273	0.187	0.566
AC-10mg/kg	AC-PO7d	3	999	2.706	0.324	0.566
AC-10mg/kg	Ofloxacin	4	999	1.086	0.510	0.648
AC-10mg/kg	Saline	4	999	1.203	0.261	0.566
AC-10mg/kg	TS-30mg/kg	3	999	1.242	0.343	0.566
AC-10mg/kg	TS-15mg/kg	4	999	1.093	0.522	0.648
AC-10mg/kg	anti-tip-chimer	3	999	1.256	0.634	0.761
AC-10mg/kg	anti-rsPilA	3	999	1.324	0.316	0.566
Ofloxacin	Saline	6	999	0.940	0.498	0.648
Ofloxacin	TS-30mg/kg	5	999	1.236	0.402	0.606
Ofloxacin	TS-15mg/kg	6	999	0.683	1.000	1.000
Ofloxacin	anti-tip-chimer	5	999	0.746	0.892	0.973
Ofloxacin	anti-rsPilA	5	999	1.284	0.316	0.566
Saline	TS-30mg/kg	5	999	1.304	0.110	0.566
Saline	TS-15mg/kg	6	999	0.955	0.776	0.873
Saline	anti-tip-chimer	5	999	1.057	0.206	0.566
Saline	anti-rsPilA	5	999	1.117	0.404	0.606
TS-30mg/kg	anti-tip-chimer	4	999	1.387	0.326	0.566
TS-30mg/kg	anti-rsPilA	4	999	1.295	0.326	0.566
TS-15mg/kg	TS-30mg/kg	5	999	1.182	0.195	0.566
TS-15mg/kg	anti-tip-chimer	5	999	0.841	0.709	0.823
TS-15mg/kg	anti-rsPilA	5	999	1.034	0.516	0.648
anti-tip-chimer	anti-rsPilA	4	999	1.383	0.325	0.566

Supplementary File 1
Beta Diversity Output

NPL Jaccard-9DPT

PERMANOVA Pairwise Comparisons (999 permutations)

Group 1	Group 2	Sample size	Permutations	pseudo-F	p-value	q-value
AC-PO7d	Ofloxacin	4	999	2.287	0.328	0.604
AC-PO7d	Saline	4	999	4.589	0.324	0.604
AC-PO7d	TS-30mg/kg	5	999	2.529	0.101	0.597
AC-PO7d	TS-15mg/kg	5	999	2.711	0.099	0.597
AC-PO7d	anti-tip-chimer	5	999	3.168	0.102	0.597
AC-PO7d	anti-rsPilA	4	999	5.277	0.328	0.604
AC-5mg/kg	AC-PO7d	5	999	3.432	0.092	0.597
AC-5mg/kg	AC-10mg/kg	5	999	1.043	0.383	0.604
AC-5mg/kg	Ofloxacin	5	999	0.625	1.000	1.000
AC-5mg/kg	Saline	5	999	1.322	0.291	0.604
AC-5mg/kg	TS-30mg/kg	6	999	1.102	0.272	0.604
AC-5mg/kg	TS-15mg/kg	6	999	0.730	0.893	1.000
AC-5mg/kg	anti-tip-chimer	6	999	0.896	0.710	0.947
AC-5mg/kg	anti-rsPilA	5	999	1.293	0.386	0.604
AC-10mg/kg	AC-PO7d	4	999	3.236	0.325	0.604
AC-10mg/kg	Ofloxacin	4	999	0.710	1.000	1.000
AC-10mg/kg	Saline	4	999	1.832	0.337	0.604
AC-10mg/kg	TS-30mg/kg	5	999	1.342	0.116	0.597
AC-10mg/kg	TS-15mg/kg	5	999	1.056	0.300	0.604
AC-10mg/kg	anti-tip-chimer	5	999	0.963	0.513	0.739
AC-10mg/kg	anti-rsPilA	4	999	1.516	0.321	0.604
Ofloxacin	Saline	4	999	0.850	0.673	0.932
Ofloxacin	TS-30mg/kg	5	999	0.751	1.000	1.000
Ofloxacin	TS-15mg/kg	5	999	0.610	1.000	1.000
Ofloxacin	anti-tip-chimer	5	999	0.759	1.000	1.000
Ofloxacin	anti-rsPilA	4	999	0.945	1.000	1.000
Saline	TS-30mg/kg	5	999	1.304	0.197	0.604
Saline	TS-15mg/kg	5	999	1.061	0.479	0.719
Saline	anti-tip-chimer	5	999	1.428	0.113	0.597
Saline	anti-rsPilA	4	999	1.572	0.353	0.604
TS-30mg/kg	anti-tip-chimer	6	999	1.125	0.095	0.597
TS-30mg/kg	anti-rsPilA	5	999	1.602	0.192	0.604
TS-15mg/kg	TS-30mg/kg	6	999	0.811	0.799	1.000
TS-15mg/kg	anti-tip-chimer	6	999	0.783	1.000	1.000
TS-15mg/kg	anti-rsPilA	5	999	1.259	0.298	0.604
anti-tip-chimer	anti-rsPilA	5	999	1.301	0.198	0.604

Supplementary File 1
Beta Diversity Output

NPL Weighted UniFrac PERMDISP-Baseline

Pairwise Permdisp Results (999 permutations)

Group 1	Group 2	Sample size	Permutations	F-value	p-value	q-value
A.C (10mg.kg PO 7d)	Ofloxacin	6	999	0.580734274	0.343	0.958
A.C (10mg.kg PO 7d)	Saline	6	999	0.021530827	0.889	0.958
A.C (10mg.kg PO 7d)	T.S (15mg.kg @12h)	6	999	0.193760506	0.670	0.958
A.C (10mg.kg PO 7d)	T.S (7.5mg.kg @12h)	6	999	0.161072368	0.497	0.958
A.C (10mg.kg PO 7d)	anti-IHF	6	999	0.190373847	0.511	0.958
A.C (10mg.kg PO 7d)	anti-rsPilA	6	999	0.009875062	1.000	1.000
A.C (2.5mg.kg BID)	A.C (10mg.kg PO 7d)	6	999	40.52640138	0.096	0.806
A.C (2.5mg.kg BID)	A.C (5mg.kg BID)	5	999	0.032455503	0.780	0.958
A.C (2.5mg.kg BID)	Ofloxacin	6	999	0.379899031	0.757	0.958
A.C (2.5mg.kg BID)	Saline	6	999	0.972900893	0.105	0.806
A.C (2.5mg.kg BID)	T.S (15mg.kg @12h)	6	999	3.822600172	0.086	0.806
A.C (2.5mg.kg BID)	T.S (7.5mg.kg @12h)	6	999	0.708199172	0.606	0.958
A.C (2.5mg.kg BID)	anti-IHF	6	999	0.469574954	0.634	0.958
A.C (2.5mg.kg BID)	anti-rsPilA	6	999	14.69655524	0.198	0.958
A.C (5mg.kg BID)	A.C (10mg.kg PO 7d)	5	999	269.1115436	0.112	0.806
A.C (5mg.kg BID)	Ofloxacin	5	999	2.03E-01	1.000	1.000
A.C (5mg.kg BID)	Saline	5	999	0.558386698	0.797	0.958
A.C (5mg.kg BID)	T.S (15mg.kg @12h)	5	999	2.408900833	0.287	0.958
A.C (5mg.kg BID)	T.S (7.5mg.kg @12h)	5	999	0.396067344	0.894	0.958
A.C (5mg.kg BID)	anti-IHF	5	999	0.257830279	0.890	0.958
A.C (5mg.kg BID)	anti-rsPilA	5	999	12.54744119	0.095	0.806
Ofloxacin	Saline	6	999	0.272768582	0.593	0.958
Ofloxacin	T.S (15mg.kg @12h)	6	999	0.207752799	0.612	0.958
Ofloxacin	T.S (7.5mg.kg @12h)	6	999	0.045580693	0.696	0.958
Ofloxacin	anti-IHF	6	999	0.020279338	0.800	0.958
Ofloxacin	anti-rsPilA	6	999	0.498026958	0.453	0.958
Saline	T.S (15mg.kg @12h)	6	999	0.078254991	0.674	0.958
Saline	T.S (7.5mg.kg @12h)	6	999	0.119955334	0.646	0.958
Saline	anti-IHF	6	999	0.143936192	0.694	0.958
Saline	anti-rsPilA	6	999	0.026303604	0.850	0.958
T.S (15mg.kg @12h)	anti-IHF	6	999	0.04765262	0.722	0.958
T.S (15mg.kg @12h)	anti-rsPilA	6	999	0.106144786	0.520	0.958
T.S (7.5mg.kg @12h)	T.S (15mg.kg @12h)	6	999	0.02659436	0.798	0.958
T.S (7.5mg.kg @12h)	anti-IHF	6	999	0.003134036	0.905	0.958
T.S (7.5mg.kg @12h)	anti-rsPilA	6	999	0.132127512	0.603	0.958
anti-IHF	anti-rsPilA	6	999	0.161828763	0.817	0.958

Omnibus PERMDISP Test

p-value 0.679

Supplementary File 1
Beta Diversity Output

NPL Weighted UniFrac PERMDISP-2DPT

Pairwise Permdisp Results (999 permutations)

Group 1	Group 2	Sample size	Permutations	F-value	p-value	q-value
A.C (10mg.kg PO 7d)	Ofloxacin	6	999	0.269324556	0.042	0.564
A.C (10mg.kg PO 7d)	Saline	6	999	0.514893134	0.046	0.564
A.C (10mg.kg PO 7d)	T.S (15mg.kg @12h)	6	999	5.88392E-06	0.947	1.000
A.C (10mg.kg PO 7d)	T.S (7.5mg.kg @12h)	6	999	0.536457502	0.348	0.895
A.C (10mg.kg PO 7d)	anti-IHF	6	999	0.377540755	0.095	0.618
A.C (10mg.kg PO 7d)	anti-rsPilA	6	999	0.102216264	0.202	0.745
A.C (2.5mg.kg BID)	A.C (10mg.kg PO 7d)	6	999	0.96703961	0.103	0.618
A.C (2.5mg.kg BID)	A.C (5mg.kg BID)	5	999	7.473615123	0.091	0.618
A.C (2.5mg.kg BID)	Ofloxacin	6	999	1.205145957	0.316	0.875
A.C (2.5mg.kg BID)	Saline	6	999	2.329703118	0.047	0.564
A.C (2.5mg.kg BID)	T.S (15mg.kg @12h)	6	999	0.747477216	0.207	0.745
A.C (2.5mg.kg BID)	T.S (7.5mg.kg @12h)	6	999	1.836513512	0.254	0.831
A.C (2.5mg.kg BID)	anti-IHF	6	999	1.758079937	0.314	0.875
A.C (2.5mg.kg BID)	anti-rsPilA	6	999	4.96785815	0.148	0.720
A.C (5mg.kg BID)	A.C (10mg.kg PO 7d)	5	999	0.051478684	0.890	0.987
A.C (5mg.kg BID)	Ofloxacin	5	999	1.13E-01	0.581	0.980
A.C (5mg.kg BID)	Saline	5	999	0.267743434	0.787	0.980
A.C (5mg.kg BID)	T.S (15mg.kg @12h)	5	999	0.037260398	0.789	0.980
A.C (5mg.kg BID)	T.S (7.5mg.kg @12h)	5	999	0.272024787	0.617	0.980
A.C (5mg.kg BID)	anti-IHF	5	999	0.175643137	0.701	0.980
A.C (5mg.kg BID)	anti-rsPilA	5	999	0.010019251	0.905	0.987
Ofloxacin	Saline	6	999	0.002983804	0.897	0.987
Ofloxacin	T.S (15mg.kg @12h)	6	999	0.249647267	0.696	0.980
Ofloxacin	T.S (7.5mg.kg @12h)	6	999	0.025546322	0.545	0.980
Ofloxacin	anti-IHF	6	999	0.000205652	1.000	1.000
Ofloxacin	anti-rsPilA	6	999	0.150049122	0.767	0.980
Saline	T.S (15mg.kg @12h)	6	999	0.45808204	0.464	0.980
Saline	T.S (7.5mg.kg @12h)	6	999	0.015295762	0.802	0.980
Saline	anti-IHF	6	999	0.001691822	1.000	1.000
Saline	anti-rsPilA	6	999	0.348757533	0.557	0.980
T.S (15mg.kg @12h)	anti-IHF	6	999	0.341348253	0.585	0.980
T.S (15mg.kg @12h)	anti-rsPilA	6	999	0.07833238	0.420	0.945
T.S (7.5mg.kg @12h)	T.S (15mg.kg @12h)	6	999	0.494753976	0.403	0.945
T.S (7.5mg.kg @12h)	anti-IHF	6	999	0.014091542	0.817	0.980
T.S (7.5mg.kg @12h)	anti-rsPilA	6	999	0.382015725	0.160	0.720
anti-IHF	anti-rsPilA	6	999	0.229481128	0.654	0.980

Omnibus PERMDISP Test

p-value 0.803

Supplementary File 1
Beta Diversity Output

NPL Weighted UniFrac PERMDISP-5DPT

Pairwise Permdisp Results (999 permutations)

Group 1	Group 2	Sample size	Permutations	F-value	p-value	q-value
A.C (10mg.kg PO 7d)	Ofloxacin	6	999	0.109480438	0.949	1.000
A.C (10mg.kg PO 7d)	Saline	6	999	1.188621949	0.213	1.000
A.C (10mg.kg PO 7d)	T.S (15mg.kg @12h)	6	999	0.046688651	0.389	1.000
A.C (10mg.kg PO 7d)	T.S (7.5mg.kg @12h)	6	999	0.02721899	0.709	1.000
A.C (10mg.kg PO 7d)	anti-IHF	6	999	0.08473778	0.404	1.000
A.C (10mg.kg PO 7d)	anti-rsPilA	5	999	1.6066988	0.206	1.000
A.C (2.5mg.kg BID)	A.C (10mg.kg PO 7d)	6	999	0.399770001	0.103	1.000
A.C (2.5mg.kg BID)	A.C (5mg.kg BID)	5	999	6.725737075	0.292	1.000
A.C (2.5mg.kg BID)	Ofloxacin	6	999	0.359212803	0.910	1.000
A.C (2.5mg.kg BID)	Saline	6	999	4.485772828	0.283	1.000
A.C (2.5mg.kg BID)	T.S (15mg.kg @12h)	6	999	0.01823181	0.953	1.000
A.C (2.5mg.kg BID)	T.S (7.5mg.kg @12h)	6	999	0.105715098	0.897	1.000
A.C (2.5mg.kg BID)	anti-IHF	6	999	0.055226445	0.746	1.000
A.C (2.5mg.kg BID)	anti-rsPilA	5	999	9.608515348	0.312	1.000
A.C (5mg.kg BID)	A.C (10mg.kg PO 7d)	5	999	0.017989261	0.908	1.000
A.C (5mg.kg BID)	Ofloxacin	5	999	4.98E-02	0.803	1.000
A.C (5mg.kg BID)	Saline	5	999	1.115093031	0.408	1.000
A.C (5mg.kg BID)	T.S (15mg.kg @12h)	5	999	0.075487725	0.800	1.000
A.C (5mg.kg BID)	T.S (7.5mg.kg @12h)	5	999	0.078272204	0.700	1.000
A.C (5mg.kg BID)	anti-IHF	5	999	0.206785125	0.685	1.000
A.C (5mg.kg BID)	anti-rsPilA	4	999	inf	0.663	1.000
Ofloxacin	Saline	6	999	0.048402122	0.599	1.000
Ofloxacin	T.S (15mg.kg @12h)	6	999	0.188162148	0.807	1.000
Ofloxacin	T.S (7.5mg.kg @12h)	6	999	0.169284713	0.890	1.000
Ofloxacin	anti-IHF	6	999	0.230112689	0.785	1.000
Ofloxacin	anti-rsPilA	5	999	0.57283195	0.816	1.000
Saline	T.S (15mg.kg @12h)	6	999	1.013051684	0.270	1.000
Saline	T.S (7.5mg.kg @12h)	6	999	1.339801513	0.183	1.000
Saline	anti-IHF	6	999	1.913148313	0.182	1.000
Saline	anti-rsPilA	5	999	5.977668712	0.294	1.000
T.S (15mg.kg @12h)	anti-IHF	6	999	3.56901E-05	1.000	1.000
T.S (15mg.kg @12h)	anti-rsPilA	5	999	0.314108303	0.504	1.000
T.S (7.5mg.kg @12h)	T.S (15mg.kg @12h)	6	999	0.006497369	1.000	1.000
T.S (7.5mg.kg @12h)	anti-IHF	6	999	0.005778685	0.702	1.000
T.S (7.5mg.kg @12h)	anti-rsPilA	5	999	0.80819973	0.514	1.000
anti-IHF	anti-rsPilA	5	999	0.912410564	0.614	1.000

Omnibus PERMDISP Test

p-value 0.795

Supplementary File 1
Beta Diversity Output

NPL Weighted UniFrac PERMDISP-7DPT

Pairwise Permdisp Results (999 permutations)

Group 1	Group 2	Sample size	Permutations	F-value	p-value	q-value
A.C (10mg.kg PO 7d)	Ofloxacin	5	999	0.005255401	1.000	1.000
A.C (10mg.kg PO 7d)	Saline	5	999	2.710934845	0.304	0.995
A.C (10mg.kg PO 7d)	T.S (15mg.kg @12h)	4	999	inf	1.000	1.000
A.C (10mg.kg PO 7d)	T.S (7.5mg.kg @12h)	5	999	0.002285494	1.000	1.000
A.C (10mg.kg PO 7d)	anti-IHF	4	999	inf	1.000	1.000
A.C (10mg.kg PO 7d)	anti-rsPilA	4	999	inf	1.000	1.000
A.C (2.5mg.kg BID)	A.C (10mg.kg PO 7d)	5	999	4.261886363	0.091	0.995
A.C (2.5mg.kg BID)	A.C (5mg.kg BID)	4	999	6.462233996	0.271	0.995
A.C (2.5mg.kg BID)	Ofloxacin	6	999	0.524599366	0.714	1.000
A.C (2.5mg.kg BID)	Saline	6	999	9.021727412	0.039	0.995
A.C (2.5mg.kg BID)	T.S (15mg.kg @12h)	5	999	0.251925127	0.491	1.000
A.C (2.5mg.kg BID)	T.S (7.5mg.kg @12h)	6	999	1.397373094	0.273	0.995
A.C (2.5mg.kg BID)	anti-IHF	5	999	0.457440779	0.580	1.000
A.C (2.5mg.kg BID)	anti-rsPilA	5	999	1.374549338	0.297	0.995
A.C (5mg.kg BID)	A.C (10mg.kg PO 7d)	3	999	inf	1.000	1.000
A.C (5mg.kg BID)	Ofloxacin	4	999	9.90E-01	1.000	1.000
A.C (5mg.kg BID)	Saline	4	999	7.416979739	0.751	1.000
A.C (5mg.kg BID)	T.S (15mg.kg @12h)	3	999	inf	1.000	1.000
A.C (5mg.kg BID)	T.S (7.5mg.kg @12h)	4	999	3.375649986	0.497	1.000
A.C (5mg.kg BID)	anti-IHF	3	999	inf	1.000	1.000
A.C (5mg.kg BID)	anti-rsPilA	3	999	inf	1.000	1.000
Ofloxacin	Saline	6	999	0.945831098	0.218	0.995
Ofloxacin	T.S (15mg.kg @12h)	5	999	0.206825775	0.585	1.000
Ofloxacin	T.S (7.5mg.kg @12h)	6	999	0.002936766	0.937	1.000
Ofloxacin	anti-IHF	5	999	0.169831831	1.000	1.000
Ofloxacin	anti-rsPilA	5	999	0.084528425	0.820	1.000
Saline	T.S (15mg.kg @12h)	5	999	5.364561154	0.207	0.995
Saline	T.S (7.5mg.kg @12h)	6	999	2.319285824	0.114	0.995
Saline	anti-IHF	5	999	5.023867001	0.199	0.995
Saline	anti-rsPilA	5	999	4.116004646	0.206	0.995
T.S (15mg.kg @12h)	anti-IHF	4	999	inf	1.000	1.000
T.S (15mg.kg @12h)	anti-rsPilA	4	999	inf	1.000	1.000
T.S (7.5mg.kg @12h)	T.S (15mg.kg @12h)	5	999	0.602601585	0.381	1.000
T.S (7.5mg.kg @12h)	anti-IHF	5	999	0.482963595	0.805	1.000
T.S (7.5mg.kg @12h)	anti-rsPilA	5	999	0.215000277	0.600	1.000
anti-IHF	anti-rsPilA	4	999	inf	1.000	1.000

Omnibus PERMDISP Test

p-value 0.314

Supplementary File 1
Beta Diversity Output

NPL Weighted UniFrac PERMDISP-9DPT

Pairwise Permdisp Results (999 permutations)

Group 1	Group 2	Sample size	Permutations	F-value	p-value	q-value
A.C (10mg.kg PO 7d)	Ofloxacin	4	999	inf	1.000	1.000
A.C (10mg.kg PO 7d)	Saline	4	999	-1.17048E+16	1.000	1.000
A.C (10mg.kg PO 7d)	T.S (15mg.kg @12h)	5	999	32.38378742	0.091	0.648
A.C (10mg.kg PO 7d)	T.S (7.5mg.kg @12h)	5	999	0.897438375	0.276	1.000
A.C (10mg.kg PO 7d)	anti-IHF	5	999	3.127260566	0.101	0.648
A.C (10mg.kg PO 7d)	anti-rsPilA	4	999	inf	1.000	1.000
A.C (2.5mg.kg BID)	A.C (10mg.kg PO 7d)	5	999	2.108061968	0.101	0.648
A.C (2.5mg.kg BID)	A.C (5mg.kg BID)	5	999	0.331602225	0.684	1.000
A.C (2.5mg.kg BID)	Ofloxacin	5	999	3.614118863	0.314	1.000
A.C (2.5mg.kg BID)	Saline	5	999	1.870141344	0.394	1.000
A.C (2.5mg.kg BID)	T.S (15mg.kg @12h)	6	999	0.032846931	0.637	1.000
A.C (2.5mg.kg BID)	T.S (7.5mg.kg @12h)	6	999	0.083491145	0.897	1.000
A.C (2.5mg.kg BID)	anti-IHF	6	999	0.207648445	0.611	1.000
A.C (2.5mg.kg BID)	anti-rsPilA	5	999	0.922457406	0.425	1.000
A.C (5mg.kg BID)	A.C (10mg.kg PO 7d)	4	999	1.26985E+16	1.000	1.000
A.C (5mg.kg BID)	Ofloxacin	4	999	inf	1.000	1.000
A.C (5mg.kg BID)	Saline	4	999	inf	1.000	1.000
A.C (5mg.kg BID)	T.S (15mg.kg @12h)	5	999	1.880517228	0.403	1.000
A.C (5mg.kg BID)	T.S (7.5mg.kg @12h)	5	999	2.03334E-05	1.000	1.000
A.C (5mg.kg BID)	anti-IHF	5	999	0.000114324	0.893	1.000
A.C (5mg.kg BID)	anti-rsPilA	4	999	inf	1.000	1.000
Ofloxacin	Saline	4	999	inf	0.844	1.000
Ofloxacin	T.S (15mg.kg @12h)	5	999	31.47045786	0.108	0.648
Ofloxacin	T.S (7.5mg.kg @12h)	5	999	0.331539163	0.637	1.000
Ofloxacin	anti-IHF	5	999	1.163136747	0.293	1.000
Ofloxacin	anti-rsPilA	4	999	inf	0.846	1.000
Saline	T.S (15mg.kg @12h)	5	999	15.19232523	0.105	0.648
Saline	T.S (7.5mg.kg @12h)	5	999	0.110410345	0.603	1.000
Saline	anti-IHF	5	999	0.418817319	0.519	1.000
Saline	anti-rsPilA	4	999	inf	1.000	1.000
T.S (15mg.kg @12h)	anti-IHF	6	999	0.175646679	0.591	1.000
T.S (15mg.kg @12h)	anti-rsPilA	5	999	12.54758642	0.093	0.648
T.S (7.5mg.kg @12h)	T.S (15mg.kg @12h)	6	999	0.053226547	0.498	1.000
T.S (7.5mg.kg @12h)	anti-IHF	6	999	2.15442E-07	1.000	1.000
T.S (7.5mg.kg @12h)	anti-rsPilA	5	999	0.43264898	0.713	1.000
anti-IHF	anti-rsPilA	5	999	1.505932279	0.679	1.000

Omnibus PERMDISP Test

p-value 0.316

Supplementary File 1
Beta Diversity Output

NPL Unweighted UniFrac PERMDISP-Baseline

Pairwise Permdisp Results (999 permutations)

Group 1	Group 2	Sample size	Permutations	F-value	p-value	q-value
A.C (10mg.kg PO 7d)	Ofloxacin	6	999	1.64406722	0.052	0.671
A.C (10mg.kg PO 7d)	Saline	6	999	1.877724285	0.170	0.671
A.C (10mg.kg PO 7d)	T.S (15mg.kg @12h)	6	999	0.52064373	0.039	0.671
A.C (10mg.kg PO 7d)	T.S (7.5mg.kg @12h)	6	999	0.31111956	0.696	0.845
A.C (10mg.kg PO 7d)	anti-IHF	6	999	0.116404281	0.409	0.736
A.C (10mg.kg PO 7d)	anti-rsPilA	6	999	2.039882585	0.111	0.671
A.C (2.5mg.kg BID)	A.C (10mg.kg PO 7d)	6	999	0.029500893	0.753	0.847
A.C (2.5mg.kg BID)	A.C (5mg.kg BID)	5	999	0.774985235	0.473	0.736
A.C (2.5mg.kg BID)	Ofloxacin	6	999	0.821833121	0.313	0.736
A.C (2.5mg.kg BID)	Saline	6	999	1.204472811	0.308	0.736
A.C (2.5mg.kg BID)	T.S (15mg.kg @12h)	6	999	0.350600194	0.496	0.736
A.C (2.5mg.kg BID)	T.S (7.5mg.kg @12h)	6	999	0.130240413	0.729	0.847
A.C (2.5mg.kg BID)	anti-IHF	6	999	0.21756866	0.800	0.873
A.C (2.5mg.kg BID)	anti-rsPilA	6	999	1.347238701	0.144	0.671
A.C (5mg.kg BID)	A.C (10mg.kg PO 7d)	5	999	0.811354156	0.474	0.736
A.C (5mg.kg BID)	Ofloxacin	5	999	7.67E+00	0.205	0.671
A.C (5mg.kg BID)	Saline	5	999	3.875390464	0.299	0.736
A.C (5mg.kg BID)	T.S (15mg.kg @12h)	5	999	0.940768114	0.238	0.714
A.C (5mg.kg BID)	T.S (7.5mg.kg @12h)	5	999	1.385983878	0.610	0.788
A.C (5mg.kg BID)	anti-IHF	5	999	0.196027544	0.704	0.845
A.C (5mg.kg BID)	anti-rsPilA	5	999	3.893422257	0.110	0.671
Ofloxacin	Saline	6	999	0.197512583	0.411	0.736
Ofloxacin	T.S (15mg.kg @12h)	6	999	0.012496306	1.000	1.000
Ofloxacin	T.S (7.5mg.kg @12h)	6	999	0.175109298	0.494	0.736
Ofloxacin	anti-IHF	6	999	2.42519868	0.090	0.671
Ofloxacin	anti-rsPilA	6	999	0.286762383	0.511	0.736
Saline	T.S (15mg.kg @12h)	6	999	0.0216064	1.000	1.000
Saline	T.S (7.5mg.kg @12h)	6	999	0.490312089	0.448	0.736
Saline	anti-IHF	6	999	2.551316954	0.191	0.671
Saline	anti-rsPilA	6	999	0.007690239	0.908	0.961
T.S (15mg.kg @12h)	anti-IHF	6	999	0.806373181	0.568	0.786
T.S (15mg.kg @12h)	anti-rsPilA	6	999	0.042673827	0.434	0.736
T.S (7.5mg.kg @12h)	T.S (15mg.kg @12h)	6	999	0.114703244	0.613	0.788
T.S (7.5mg.kg @12h)	anti-IHF	6	999	0.671857834	0.464	0.736
T.S (7.5mg.kg @12h)	anti-rsPilA	6	999	0.594553746	0.191	0.671
anti-IHF	anti-rsPilA	6	999	2.716542525	0.174	0.671

Omnibus PERMDISP Test

p-value 0.251

NPL Unweighted UniFrac PERMDISP-2DPT

Pairwise Permdisp Results (999 permutations)

Group 1	Group 2	Sample size	Permutations	F-value	p-value	q-value
A.C (10mg.kg PO 7d)	Ofloxacin	6	999	0.073443731	0.804	0.998
A.C (10mg.kg PO 7d)	Saline	6	999	1.07975375	0.202	0.686
A.C (10mg.kg PO 7d)	T.S (15mg.kg @12h)	6	999	0.089134752	0.683	0.890
A.C (10mg.kg PO 7d)	T.S (7.5mg.kg @12h)	6	999	0.004027268	0.889	1.000
A.C (10mg.kg PO 7d)	anti-IHF	6	999	0.468581199	0.520	0.814
A.C (10mg.kg PO 7d)	anti-rsPilA	6	999	0.410870049	0.496	0.812
A.C (2.5mg.kg BID)	A.C (10mg.kg PO 7d)	6	999	0.665304886	0.366	0.729
A.C (2.5mg.kg BID)	A.C (5mg.kg BID)	5	999	0.280126562	0.602	0.842
A.C (2.5mg.kg BID)	Ofloxacin	6	999	0.958050643	0.405	0.729
A.C (2.5mg.kg BID)	Saline	6	999	0.002850952	0.958	1.000
A.C (2.5mg.kg BID)	T.S (15mg.kg @12h)	6	999	1.029999682	0.249	0.686
A.C (2.5mg.kg BID)	T.S (7.5mg.kg @12h)	6	999	0.70562381	0.201	0.686
A.C (2.5mg.kg BID)	anti-IHF	6	999	0.002638867	1.000	1.000
A.C (2.5mg.kg BID)	anti-rsPilA	6	999	0.152016828	0.389	0.729
A.C (5mg.kg BID)	A.C (10mg.kg PO 7d)	5	999	5.420331121	0.296	0.686
A.C (5mg.kg BID)	Ofloxacin	5	999	5.47E+00	0.094	0.686
A.C (5mg.kg BID)	Saline	5	999	0.361991353	0.887	1.000
A.C (5mg.kg BID)	T.S (15mg.kg @12h)	5	999	7.051869958	0.082	0.686
A.C (5mg.kg BID)	T.S (7.5mg.kg @12h)	5	999	13.37928734	0.114	0.686
A.C (5mg.kg BID)	anti-IHF	5	999	0.277798485	0.606	0.842
A.C (5mg.kg BID)	anti-rsPilA	5	999	2.685662707	0.192	0.686
Ofloxacin	Saline	6	999	1.479128069	0.194	0.686
Ofloxacin	T.S (15mg.kg @12h)	6	999	7.57988E-05	1.000	1.000
Ofloxacin	T.S (7.5mg.kg @12h)	6	999	0.140671847	0.692	0.890
Ofloxacin	anti-IHF	6	999	0.705082715	0.232	0.686
Ofloxacin	anti-rsPilA	6	999	0.760573495	0.094	0.686
Saline	T.S (15mg.kg @12h)	6	999	1.626973501	0.106	0.686
Saline	T.S (7.5mg.kg @12h)	6	999	1.23198426	0.305	0.686
Saline	anti-IHF	6	999	0.011230438	1.000	1.000
Saline	anti-rsPilA	6	999	0.285492999	0.243	0.686
T.S (15mg.kg @12h)	anti-IHF	6	999	0.751529405	0.495	0.812
T.S (15mg.kg @12h)	anti-rsPilA	6	999	0.884635988	0.196	0.686
T.S (7.5mg.kg @12h)	T.S (15mg.kg @12h)	6	999	0.178264266	0.906	1.000
T.S (7.5mg.kg @12h)	anti-IHF	6	999	0.480571433	0.278	0.686
T.S (7.5mg.kg @12h)	anti-rsPilA	6	999	0.500627986	0.393	0.729
anti-IHF	anti-rsPilA	6	999	0.086788322	0.608	0.842

Omnibus PERMDISP Test

p-value 0.475

Supplementary File 1
Beta Diversity Output

NPL Unweighted UniFrac PERMDISP-5DPT

Pairwise Permdisp Results (999 permutations)

Group 1	Group 2	Sample size	Permutations	F-value	p-value	q-value
A.C (10mg.kg PO 7d)	Ofloxacin	6	999	0.034744781	0.895	1.000
A.C (10mg.kg PO 7d)	Saline	6	999	0.325232263	0.525	0.876
A.C (10mg.kg PO 7d)	T.S (15mg.kg @12h)	6	999	0.07096293	0.896	1.000
A.C (10mg.kg PO 7d)	T.S (7.5mg.kg @12h)	6	999	0.006122334	0.858	1.000
A.C (10mg.kg PO 7d)	anti-IHF	6	999	0.193513686	0.781	1.000
A.C (10mg.kg PO 7d)	anti-rsPilA	5	999	0.209776638	0.605	0.876
A.C (2.5mg.kg BID)	A.C (10mg.kg PO 7d)	6	999	0.305090693	0.734	0.979
A.C (2.5mg.kg BID)	A.C (5mg.kg BID)	5	999	3.96723816	0.414	0.876
A.C (2.5mg.kg BID)	Ofloxacin	6	999	0.528496267	0.339	0.876
A.C (2.5mg.kg BID)	Saline	6	999	1.832546806	0.228	0.876
A.C (2.5mg.kg BID)	T.S (15mg.kg @12h)	6	999	1.936329363	0.105	0.876
A.C (2.5mg.kg BID)	T.S (7.5mg.kg @12h)	6	999	0.744215297	0.341	0.876
A.C (2.5mg.kg BID)	anti-IHF	6	999	0.005011862	1.000	1.000
A.C (2.5mg.kg BID)	anti-rsPilA	5	999	0.000114338	1.000	1.000
A.C (5mg.kg BID)	A.C (10mg.kg PO 7d)	5	999	1.635411715	0.484	0.876
A.C (5mg.kg BID)	Ofloxacin	5	999	9.55E+00	0.108	0.876
A.C (5mg.kg BID)	Saline	5	999	4.096148402	0.306	0.876
A.C (5mg.kg BID)	T.S (15mg.kg @12h)	5	999	9.298656489	0.206	0.876
A.C (5mg.kg BID)	T.S (7.5mg.kg @12h)	5	999	3.847395838	0.283	0.876
A.C (5mg.kg BID)	anti-IHF	5	999	1.479410581	0.414	0.876
A.C (5mg.kg BID)	anti-rsPilA	4	999	inf	1.000	1.000
Ofloxacin	Saline	6	999	0.957350434	0.561	0.876
Ofloxacin	T.S (15mg.kg @12h)	6	999	0.65066138	0.173	0.876
Ofloxacin	T.S (7.5mg.kg @12h)	6	999	0.141597839	0.516	0.876
Ofloxacin	anti-IHF	6	999	0.183030037	0.379	0.876
Ofloxacin	anti-rsPilA	5	999	0.698678527	0.608	0.876
Saline	T.S (15mg.kg @12h)	6	999	0.208969201	1.000	1.000
Saline	T.S (7.5mg.kg @12h)	6	999	0.348820503	0.718	0.979
Saline	anti-IHF	6	999	1.271104144	0.513	0.876
Saline	anti-rsPilA	5	999	1.30238144	0.594	0.876
T.S (15mg.kg @12h)	anti-IHF	6	999	0.93698311	0.319	0.876
T.S (15mg.kg @12h)	anti-rsPilA	5	999	1.934147159	0.412	0.876
T.S (7.5mg.kg @12h)	T.S (15mg.kg @12h)	6	999	0.056748806	0.578	0.876
T.S (7.5mg.kg @12h)	anti-IHF	6	999	0.411565626	0.530	0.876
T.S (7.5mg.kg @12h)	anti-rsPilA	5	999	0.594494082	0.597	0.876
anti-IHF	anti-rsPilA	5	999	0.003268648	0.893	1.000

Omnibus PERMDISP Test

p-value 0.365

Supplementary File 1
Beta Diversity Output

NPL Unweighted UniFrac PERMDISP-7DPT

Pairwise Permdisp Results (999 permutations)

Group 1	Group 2	Sample size	Permutations	F-value	p-value	q-value
A.C (10mg.kg PO 7d)	Ofloxacin	5	999	0.379312965	0.594	1.000
A.C (10mg.kg PO 7d)	Saline	5	999	0.744235448	0.501	1.000
A.C (10mg.kg PO 7d)	T.S (15mg.kg @12h)	4	999	inf	1.000	1.000
A.C (10mg.kg PO 7d)	T.S (7.5mg.kg @12h)	5	999	0.787931072	0.598	1.000
A.C (10mg.kg PO 7d)	anti-IHF	4	999	inf	0.852	1.000
A.C (10mg.kg PO 7d)	anti-rsPilA	4	999	inf	1.000	1.000
A.C (2.5mg.kg BID)	A.C (10mg.kg PO 7d)	5	999	0.676203085	0.489	1.000
A.C (2.5mg.kg BID)	A.C (5mg.kg BID)	4	999	9.492423938	0.492	1.000
A.C (2.5mg.kg BID)	Ofloxacin	6	999	0.031316712	0.796	1.000
A.C (2.5mg.kg BID)	Saline	6	999	0.467448931	0.698	1.000
A.C (2.5mg.kg BID)	T.S (15mg.kg @12h)	5	999	0.307387088	0.805	1.000
A.C (2.5mg.kg BID)	T.S (7.5mg.kg @12h)	6	999	0.09648925	0.784	1.000
A.C (2.5mg.kg BID)	anti-IHF	5	999	2.040733566	0.313	1.000
A.C (2.5mg.kg BID)	anti-rsPilA	5	999	0.522943727	0.595	1.000
A.C (5mg.kg BID)	A.C (10mg.kg PO 7d)	3	999	inf	1.000	1.000
A.C (5mg.kg BID)	Ofloxacin	4	999	3.32E+00	0.736	1.000
A.C (5mg.kg BID)	Saline	4	999	2.203951752	0.757	1.000
A.C (5mg.kg BID)	T.S (15mg.kg @12h)	3	999	inf	1.000	1.000
A.C (5mg.kg BID)	T.S (7.5mg.kg @12h)	4	999	5.7922915	0.523	1.000
A.C (5mg.kg BID)	anti-IHF	3	999	inf	1.000	1.000
A.C (5mg.kg BID)	anti-rsPilA	3	999	inf	1.000	1.000
Ofloxacin	Saline	6	999	0.248813264	0.690	1.000
Ofloxacin	T.S (15mg.kg @12h)	5	999	0.217513321	0.578	1.000
Ofloxacin	T.S (7.5mg.kg @12h)	6	999	0.005546824	0.859	1.000
Ofloxacin	anti-IHF	5	999	0.910494564	0.680	1.000
Ofloxacin	anti-rsPilA	5	999	0.314207394	0.518	1.000
Saline	T.S (15mg.kg @12h)	5	999	0.58558787	0.799	1.000
Saline	T.S (7.5mg.kg @12h)	6	999	0.227026257	0.774	1.000
Saline	anti-IHF	5	999	1.173376258	0.603	1.000
Saline	anti-rsPilA	5	999	0.683293383	0.795	1.000
T.S (15mg.kg @12h)	anti-IHF	4	999	inf	1.000	1.000
T.S (15mg.kg @12h)	anti-rsPilA	4	999	1.24453E+16	1.000	1.000
T.S (7.5mg.kg @12h)	T.S (15mg.kg @12h)	5	999	0.483058734	0.798	1.000
T.S (7.5mg.kg @12h)	anti-IHF	5	999	1.751839342	0.435	1.000
T.S (7.5mg.kg @12h)	anti-rsPilA	5	999	0.666422831	0.776	1.000
anti-IHF	anti-rsPilA	4	999	inf	1.000	1.000

Omnibus PERMDISP Test

p-value 0.961

Supplementary File 1
Beta Diversity Output

NPL Unweighted UniFrac PERMDISP-9DPT

Pairwise Permdisp Results (999 permutations)

Group 1	Group 2	Sample size	Permutations	F-value	p-value	q-value
A.C (10mg.kg PO 7d)	Ofloxacin	4	999	inf	1.000	1.000
A.C (10mg.kg PO 7d)	Saline	4	999	inf	1.000	1.000
A.C (10mg.kg PO 7d)	T.S (15mg.kg @12h)	5	999	6.425541683	0.094	0.545
A.C (10mg.kg PO 7d)	T.S (7.5mg.kg @12h)	5	999	24.5521789	0.099	0.545
A.C (10mg.kg PO 7d)	anti-IHF	5	999	5.635451178	0.086	0.545
A.C (10mg.kg PO 7d)	anti-rsPilA	4	999	-9.33581E+15	1.000	1.000
A.C (2.5mg.kg BID)	A.C (10mg.kg PO 7d)	5	999	1.643983406	0.087	0.545
A.C (2.5mg.kg BID)	A.C (5mg.kg BID)	5	999	0.031751326	1.000	1.000
A.C (2.5mg.kg BID)	Ofloxacin	5	999	0.04792046	0.804	1.000
A.C (2.5mg.kg BID)	Saline	5	999	0.264873139	0.808	1.000
A.C (2.5mg.kg BID)	T.S (15mg.kg @12h)	6	999	0.560991564	0.492	1.000
A.C (2.5mg.kg BID)	T.S (7.5mg.kg @12h)	6	999	0.900934656	0.291	0.847
A.C (2.5mg.kg BID)	anti-IHF	6	999	0.006143856	1.000	1.000
A.C (2.5mg.kg BID)	anti-rsPilA	5	999	0.07696751	0.501	1.000
A.C (5mg.kg BID)	A.C (10mg.kg PO 7d)	4	999	inf	1.000	1.000
A.C (5mg.kg BID)	Ofloxacin	4	999	inf	1.000	1.000
A.C (5mg.kg BID)	Saline	4	999	inf	1.000	1.000
A.C (5mg.kg BID)	T.S (15mg.kg @12h)	5	999	1.328722537	0.525	1.000
A.C (5mg.kg BID)	T.S (7.5mg.kg @12h)	5	999	5.403194732	0.188	0.691
A.C (5mg.kg BID)	anti-IHF	5	999	0.18969651	0.706	1.000
A.C (5mg.kg BID)	anti-rsPilA	4	999	inf	1.000	1.000
Ofloxacin	Saline	4	999	inf	0.835	1.000
Ofloxacin	T.S (15mg.kg @12h)	5	999	1.448846773	0.306	0.847
Ofloxacin	T.S (7.5mg.kg @12h)	5	999	5.863658816	0.100	0.545
Ofloxacin	anti-IHF	5	999	0.257083858	0.805	1.000
Ofloxacin	anti-rsPilA	4	999	inf	1.000	1.000
Saline	T.S (15mg.kg @12h)	5	999	2.477324307	0.206	0.691
Saline	T.S (7.5mg.kg @12h)	5	999	9.773133699	0.106	0.545
Saline	anti-IHF	5	999	1.053310503	0.496	1.000
Saline	anti-rsPilA	4	999	inf	0.673	1.000
T.S (15mg.kg @12h)	anti-IHF	6	999	0.78358212	0.402	1.000
T.S (15mg.kg @12h)	anti-rsPilA	5	999	1.630599469	0.211	0.691
T.S (7.5mg.kg @12h)	T.S (15mg.kg @12h)	6	999	0.006154847	0.862	1.000
T.S (7.5mg.kg @12h)	anti-IHF	6	999	1.762276794	0.098	0.545
T.S (7.5mg.kg @12h)	anti-rsPilA	5	999	6.55843785	0.196	0.691
anti-IHF	anti-rsPilA	5	999	0.371841099	0.914	1.000

Omnibus PERMDISP Test

p-value 0.241

Supplementary File 1
Beta Diversity Output

NPL Bray-Curtis PERMDISP-Baseline

Pairwise Permdisp Results (999 permutations)

Group 1	Group 2	Sample size	Permutations	F-value	p-value	q-value
A.C (10mg.kg PO 7d)	Ofloxacin	6	999	0.891056624	0.301	1.000
A.C (10mg.kg PO 7d)	Saline	6	999	0.18411755	0.512	1.000
A.C (10mg.kg PO 7d)	T.S (15mg.kg @12h)	6	999	0.550358593	0.389	1.000
A.C (10mg.kg PO 7d)	T.S (7.5mg.kg @12h)	6	999	0.213481468	0.652	1.000
A.C (10mg.kg PO 7d)	anti-IHF	6	999	1.263562881	0.139	1.000
A.C (10mg.kg PO 7d)	anti-rsPilA	6	999	0.729096857	0.293	1.000
A.C (2.5mg.kg BID)	A.C (10mg.kg PO 7d)	6	999	9.231667066	0.048	1.000
A.C (2.5mg.kg BID)	A.C (5mg.kg BID)	5	999	0.525130115	0.792	1.000
A.C (2.5mg.kg BID)	Ofloxacin	6	999	0.539150272	0.639	1.000
A.C (2.5mg.kg BID)	Saline	6	999	2.177895146	0.084	1.000
A.C (2.5mg.kg BID)	T.S (15mg.kg @12h)	6	999	4.066613287	0.129	1.000
A.C (2.5mg.kg BID)	T.S (7.5mg.kg @12h)	6	999	0.628791814	0.886	1.000
A.C (2.5mg.kg BID)	anti-IHF	6	999	0.996442508	0.403	1.000
A.C (2.5mg.kg BID)	anti-rsPilA	6	999	5.600419215	0.295	1.000
A.C (5mg.kg BID)	A.C (10mg.kg PO 7d)	5	999	5.112137059	0.310	1.000
A.C (5mg.kg BID)	Ofloxacin	5	999	2.45E-01	1.000	1.000
A.C (5mg.kg BID)	Saline	5	999	1.137669733	0.800	1.000
A.C (5mg.kg BID)	T.S (15mg.kg @12h)	5	999	2.1214252	0.291	1.000
A.C (5mg.kg BID)	T.S (7.5mg.kg @12h)	5	999	0.31506478	1.000	1.000
A.C (5mg.kg BID)	anti-IHF	5	999	0.459725169	1.000	1.000
A.C (5mg.kg BID)	anti-rsPilA	5	999	2.950492995	0.390	1.000
Ofloxacin	Saline	6	999	0.213821323	0.583	1.000
Ofloxacin	T.S (15mg.kg @12h)	6	999	0.167373127	0.691	1.000
Ofloxacin	T.S (7.5mg.kg @12h)	6	999	0.050141151	0.792	1.000
Ofloxacin	anti-IHF	6	999	0.000259215	0.949	1.000
Ofloxacin	anti-rsPilA	6	999	0.157750575	0.508	1.000
Saline	T.S (15mg.kg @12h)	6	999	0.019500447	0.828	1.000
Saline	T.S (7.5mg.kg @12h)	6	999	0.020175597	0.753	1.000
Saline	anti-IHF	6	999	0.261869399	0.480	1.000
Saline	anti-rsPilA	6	999	0.032104387	0.898	1.000
T.S (15mg.kg @12h)	anti-IHF	6	999	0.226111253	0.575	1.000
T.S (15mg.kg @12h)	anti-rsPilA	6	999	0.001932678	0.945	1.000
T.S (7.5mg.kg @12h)	T.S (15mg.kg @12h)	6	999	0.003221204	0.953	1.000
T.S (7.5mg.kg @12h)	anti-IHF	6	999	0.052671494	1.000	1.000
T.S (7.5mg.kg @12h)	anti-rsPilA	6	999	0.001272431	1.000	1.000
anti-IHF	anti-rsPilA	6	999	0.221293271	0.667	1.000

**Omnibus PERMDISP Test**	
**p-value**	0.724

Supplementary File 1
Beta Diversity Output

NPL Bray-Curtis PERMDISP-2DPT

Pairwise Permdisp Results (999 permutations)

Group 1	Group 2	Sample size	Permutations	F-value	p-value	q-value
A.C (10mg.kg PO 7d)	Ofloxacin	6	999	0.386521491	0.086	0.582
A.C (10mg.kg PO 7d)	Saline	6	999	0.831740883	0.087	0.582
A.C (10mg.kg PO 7d)	T.S (15mg.kg @12h)	6	999	0.390415856	0.097	0.582
A.C (10mg.kg PO 7d)	T.S (7.5mg.kg @12h)	6	999	0.855252935	0.090	0.582
A.C (10mg.kg PO 7d)	anti-IHF	6	999	0.286541213	0.093	0.582
A.C (10mg.kg PO 7d)	anti-rsPilA	6	999	0.046159497	0.177	0.706
A.C (2.5mg.kg BID)	A.C (10mg.kg PO 7d)	6	999	0.284267432	0.053	0.582
A.C (2.5mg.kg BID)	A.C (5mg.kg BID)	5	999	0.431799415	0.620	0.885
A.C (2.5mg.kg BID)	Ofloxacin	6	999	0.912405103	0.506	0.885
A.C (2.5mg.kg BID)	Saline	6	999	4.28454624	0.196	0.706
A.C (2.5mg.kg BID)	T.S (15mg.kg @12h)	6	999	1.122078559	0.191	0.706
A.C (2.5mg.kg BID)	T.S (7.5mg.kg @12h)	6	999	1.944840197	0.342	0.882
A.C (2.5mg.kg BID)	anti-IHF	6	999	1.237226733	0.185	0.706
A.C (2.5mg.kg BID)	anti-rsPilA	6	999	1.138211599	0.320	0.882
A.C (5mg.kg BID)	A.C (10mg.kg PO 7d)	5	999	0.381441868	0.427	0.882
A.C (5mg.kg BID)	Ofloxacin	5	999	7.05E-01	0.713	0.885
A.C (5mg.kg BID)	Saline	5	999	3.812214215	0.430	0.882
A.C (5mg.kg BID)	T.S (15mg.kg @12h)	5	999	0.900673304	0.290	0.882
A.C (5mg.kg BID)	T.S (7.5mg.kg @12h)	5	999	1.463621272	0.707	0.885
A.C (5mg.kg BID)	anti-IHF	5	999	1.077144122	0.546	0.885
A.C (5mg.kg BID)	anti-rsPilA	5	999	1.333964666	0.395	0.882
Ofloxacin	Saline	6	999	0.01404486	0.735	0.885
Ofloxacin	T.S (15mg.kg @12h)	6	999	0.007156559	1.000	1.000
Ofloxacin	T.S (7.5mg.kg @12h)	6	999	0.020655785	0.790	0.885
Ofloxacin	anti-IHF	6	999	0.056169939	0.632	0.885
Ofloxacin	anti-rsPilA	6	999	0.282667154	0.873	0.924
Saline	T.S (15mg.kg @12h)	6	999	0.000445906	1.000	1.000
Saline	T.S (7.5mg.kg @12h)	6	999	0.121008693	0.685	0.885
Saline	anti-IHF	6	999	0.044334308	0.641	0.885
Saline	anti-rsPilA	6	999	0.715886914	0.484	0.885
T.S (15mg.kg @12h)	anti-IHF	6	999	0.028496772	0.790	0.885
T.S (15mg.kg @12h)	anti-rsPilA	6	999	0.273895413	0.400	0.882
T.S (7.5mg.kg @12h)	T.S (15mg.kg @12h)	6	999	0.066486026	0.811	0.885
T.S (7.5mg.kg @12h)	anti-IHF	6	999	0.208553445	0.524	0.885
T.S (7.5mg.kg @12h)	anti-rsPilA	6	999	0.716187346	0.441	0.882
anti-IHF	anti-rsPilA	6	999	0.169324274	0.806	0.885

Omnibus PERMDISP Test

p-value 0.747

Supplementary File 1
Beta Diversity Output

NPL Bray-Curtis PERMDISP-5DPT

Pairwise Permdisp Results (999 permutations)

Group 1	Group 2	Sample size	Permutations	F-value	p-value	q-value
A.C (10mg.kg PO 7d)	Ofloxacin	6	999	0.061104179	0.810	1.000
A.C (10mg.kg PO 7d)	Saline	6	999	0.011913079	0.940	1.000
A.C (10mg.kg PO 7d)	T.S (15mg.kg @12h)	6	999	0.374759416	0.372	1.000
A.C (10mg.kg PO 7d)	T.S (7.5mg.kg @12h)	6	999	0.46629632	0.349	1.000
A.C (10mg.kg PO 7d)	anti-IHF	6	999	1.402342905	0.239	1.000
A.C (10mg.kg PO 7d)	anti-rsPilA	5	999	16.63766845	0.096	1.000
A.C (2.5mg.kg BID)	A.C (10mg.kg PO 7d)	6	999	3.032269004	0.095	1.000
A.C (2.5mg.kg BID)	A.C (5mg.kg BID)	5	999	0.001750279	1.000	1.000
A.C (2.5mg.kg BID)	Ofloxacin	6	999	0.282165337	0.896	1.000
A.C (2.5mg.kg BID)	Saline	6	999	1.371298636	0.396	1.000
A.C (2.5mg.kg BID)	T.S (15mg.kg @12h)	6	999	0.073028577	0.692	1.000
A.C (2.5mg.kg BID)	T.S (7.5mg.kg @12h)	6	999	0.054224261	1.000	1.000
A.C (2.5mg.kg BID)	anti-IHF	6	999	0.02890919	0.899	1.000
A.C (2.5mg.kg BID)	anti-rsPilA	5	999	0.776604267	0.579	1.000
A.C (5mg.kg BID)	A.C (10mg.kg PO 7d)	5	999	7.207285637	0.306	1.000
A.C (5mg.kg BID)	Ofloxacin	5	999	2.13E-01	0.892	1.000
A.C (5mg.kg BID)	Saline	5	999	1.195997992	0.589	1.000
A.C (5mg.kg BID)	T.S (15mg.kg @12h)	5	999	0.062246021	1.000	1.000
A.C (5mg.kg BID)	T.S (7.5mg.kg @12h)	5	999	0.048280316	1.000	1.000
A.C (5mg.kg BID)	anti-IHF	5	999	0.038056502	0.909	1.000
A.C (5mg.kg BID)	anti-rsPilA	4	999	inf	1.000	1.000
Ofloxacin	Saline	6	999	0.076573791	0.572	1.000
Ofloxacin	T.S (15mg.kg @12h)	6	999	0.053560787	0.920	1.000
Ofloxacin	T.S (7.5mg.kg @12h)	6	999	0.071972595	0.448	1.000
Ofloxacin	anti-IHF	6	999	0.160761364	0.545	1.000
Ofloxacin	anti-rsPilA	5	999	0.645352667	1.000	1.000
Saline	T.S (15mg.kg @12h)	6	999	0.336422106	0.680	1.000
Saline	T.S (7.5mg.kg @12h)	6	999	0.402597154	0.681	1.000
Saline	anti-IHF	6	999	0.849133233	0.510	1.000
Saline	anti-rsPilA	5	999	2.607323757	0.307	1.000
T.S (15mg.kg @12h)	anti-IHF	6	999	0.020051436	0.944	1.000
T.S (15mg.kg @12h)	anti-rsPilA	5	999	0.402667869	0.638	1.000
T.S (7.5mg.kg @12h)	T.S (15mg.kg @12h)	6	999	0.001243157	1.000	1.000
T.S (7.5mg.kg @12h)	anti-IHF	6	999	0.010548827	0.720	1.000
T.S (7.5mg.kg @12h)	anti-rsPilA	5	999	0.383942746	1.000	1.000
anti-IHF	anti-rsPilA	5	999	0.669463557	0.703	1.000

Omnibus PERMDISP Test

p-value 0.865

Supplementary File 1
Beta Diversity Output

NPL Bray-Curtis PERMDISP-7DPT

Pairwise Permdisp Results (999 permutations)

Group 1	Group 2	Sample size	Permutations	F-value	p-value	q-value
A.C (10mg.kg PO 7d)	Ofloxacin	5	999	0.002305909	0.889	1.000
A.C (10mg.kg PO 7d)	Saline	5	999	1.504437603	0.503	1.000
A.C (10mg.kg PO 7d)	T.S (15mg.kg @12h)	4	999	inf	1.000	1.000
A.C (10mg.kg PO 7d)	T.S (7.5mg.kg @12h)	5	999	0.01745271	0.715	1.000
A.C (10mg.kg PO 7d)	anti-IHF	4	999	inf	1.000	1.000
A.C (10mg.kg PO 7d)	anti-rsPilA	4	999	1.3456E+16	1.000	1.000
A.C (2.5mg.kg BID)	A.C (10mg.kg PO 7d)	5	999	11.93300699	0.089	1.000
A.C (2.5mg.kg BID)	A.C (5mg.kg BID)	4	999	36.87209853	0.255	1.000
A.C (2.5mg.kg BID)	Ofloxacin	6	999	0.261028523	0.902	1.000
A.C (2.5mg.kg BID)	Saline	6	999	9.232483558	0.136	1.000
A.C (2.5mg.kg BID)	T.S (15mg.kg @12h)	5	999	24.2509991	0.283	1.000
A.C (2.5mg.kg BID)	T.S (7.5mg.kg @12h)	6	999	0.616113745	0.731	1.000
A.C (2.5mg.kg BID)	anti-IHF	5	999	0.040398919	0.792	1.000
A.C (2.5mg.kg BID)	anti-rsPilA	5	999	0.015750901	1.000	1.000
A.C (5mg.kg BID)	A.C (10mg.kg PO 7d)	3	999	inf	1.000	1.000
A.C (5mg.kg BID)	Ofloxacin	4	999	1.09E+00	1.000	1.000
A.C (5mg.kg BID)	Saline	4	999	15.04516319	0.508	1.000
A.C (5mg.kg BID)	T.S (15mg.kg @12h)	3	999	inf	1.000	1.000
A.C (5mg.kg BID)	T.S (7.5mg.kg @12h)	4	999	2.985605291	1.000	1.000
A.C (5mg.kg BID)	anti-IHF	3	999	inf	1.000	1.000
A.C (5mg.kg BID)	anti-rsPilA	3	999	inf	1.000	1.000
Ofloxacin	Saline	6	999	0.335297482	0.469	1.000
Ofloxacin	T.S (15mg.kg @12h)	5	999	0.056777867	0.886	1.000
Ofloxacin	T.S (7.5mg.kg @12h)	6	999	0.001129882	0.889	1.000
Ofloxacin	anti-IHF	5	999	0.180677682	1.000	1.000
Ofloxacin	anti-rsPilA	5	999	0.172467283	0.414	1.000
Saline	T.S (15mg.kg @12h)	5	999	0.462267648	0.788	1.000
Saline	T.S (7.5mg.kg @12h)	6	999	0.900618674	0.574	1.000
Saline	anti-IHF	5	999	6.686357688	0.098	1.000
Saline	anti-rsPilA	5	999	6.541971965	0.206	1.000
T.S (15mg.kg @12h)	anti-IHF	4	999	-1.37917E+16	1.000	1.000
T.S (15mg.kg @12h)	anti-rsPilA	4	999	inf	1.000	1.000
T.S (7.5mg.kg @12h)	T.S (15mg.kg @12h)	5	999	0.205372638	0.719	1.000
T.S (7.5mg.kg @12h)	anti-IHF	5	999	0.44386531	0.773	1.000
T.S (7.5mg.kg @12h)	anti-rsPilA	5	999	0.422168175	0.496	1.000
anti-IHF	anti-rsPilA	4	999	inf	1.000	1.000

Omnibus PERMDISP Test

p-value 0.820

Supplementary File 1
Beta Diversity Output

NPL Bray-Curtis PERMDISP-9DPT

Pairwise Permdisp Results (999 permutations)

Group 1	Group 2	Sample size	Permutations	F-value	p-value	q-value
A.C (10mg.kg PO 7d)	Ofloxacin	4	999	inf	1.000	1.000
A.C (10mg.kg PO 7d)	Saline	4	999	inf	1.000	1.000
A.C (10mg.kg PO 7d)	T.S (15mg.kg @12h)	5	999	6.14254301	0.109	1.000
A.C (10mg.kg PO 7d)	T.S (7.5mg.kg @12h)	5	999	0.881175166	0.422	1.000
A.C (10mg.kg PO 7d)	anti-IHF	5	999	33.14283277	0.091	1.000
A.C (10mg.kg PO 7d)	anti-rsPilA	4	999	inf	1.000	1.000
A.C (2.5mg.kg BID)	A.C (10mg.kg PO 7d)	5	999	7.023514038	0.104	1.000
A.C (2.5mg.kg BID)	A.C (5mg.kg BID)	5	999	1.033458188	0.403	1.000
A.C (2.5mg.kg BID)	Ofloxacin	5	999	6.517636839	0.202	1.000
A.C (2.5mg.kg BID)	Saline	5	999	0.054798124	1.000	1.000
A.C (2.5mg.kg BID)	T.S (15mg.kg @12h)	6	999	2.136324564	0.216	1.000
A.C (2.5mg.kg BID)	T.S (7.5mg.kg @12h)	6	999	0.140070247	1.000	1.000
A.C (2.5mg.kg BID)	anti-IHF	6	999	2.204130391	0.315	1.000
A.C (2.5mg.kg BID)	anti-rsPilA	5	999	4.900081452	0.313	1.000
A.C (5mg.kg BID)	A.C (10mg.kg PO 7d)	4	999	inf	1.000	1.000
A.C (5mg.kg BID)	Ofloxacin	4	999	inf	1.000	1.000
A.C (5mg.kg BID)	Saline	4	999	inf	0.857	1.000
A.C (5mg.kg BID)	T.S (15mg.kg @12h)	5	999	0.602636033	0.540	1.000
A.C (5mg.kg BID)	T.S (7.5mg.kg @12h)	5	999	0.002763506	1.000	1.000
A.C (5mg.kg BID)	anti-IHF	5	999	0.306689052	0.695	1.000
A.C (5mg.kg BID)	anti-rsPilA	4	999	inf	1.000	1.000
Ofloxacin	Saline	4	999	inf	1.000	1.000
Ofloxacin	T.S (15mg.kg @12h)	5	999	0.003983098	0.899	1.000
Ofloxacin	T.S (7.5mg.kg @12h)	5	999	0.101583695	0.620	1.000
Ofloxacin	anti-IHF	5	999	2.644948221	0.298	1.000
Ofloxacin	anti-rsPilA	4	999	inf	1.000	1.000
Saline	T.S (15mg.kg @12h)	5	999	1.298535951	0.395	1.000
Saline	T.S (7.5mg.kg @12h)	5	999	0.058406232	0.899	1.000
Saline	anti-IHF	5	999	2.76945934	0.319	1.000
Saline	anti-rsPilA	4	999	inf	1.000	1.000
T.S (15mg.kg @12h)	anti-IHF	6	999	0.533241619	0.606	1.000
T.S (15mg.kg @12h)	anti-rsPilA	5	999	5.179064614	0.219	1.000
T.S (7.5mg.kg @12h)	T.S (15mg.kg @12h)	6	999	0.162082519	0.709	1.000
T.S (7.5mg.kg @12h)	anti-IHF	6	999	0.002822712	0.864	1.000
T.S (7.5mg.kg @12h)	anti-rsPilA	5	999	0.694226731	0.892	1.000
anti-IHF	anti-rsPilA	5	999	26.39363605	0.125	1.000

Omnibus PERMDISP Test

p-value 0.396

Supplementary File 1
Beta Diversity Output

NPL Jaccard PERMDISP-Baseline

Pairwise Permdisp Results (999 permutations)

Group 1	Group 2	Sample size	Permutations	F-value	p-value	q-value
A.C (10mg.kg PO 7d)	Ofloxacin	6	999	0.097208651	0.394	0.864
A.C (10mg.kg PO 7d)	Saline	6	999	0.845497734	0.042	0.530
A.C (10mg.kg PO 7d)	T.S (15mg.kg @12h)	6	999	1.238489577	0.100	0.530
A.C (10mg.kg PO 7d)	T.S (7.5mg.kg @12h)	6	999	0.302049674	0.191	0.860
A.C (10mg.kg PO 7d)	anti-IHF	6	999	0.000215817	1.000	1.000
A.C (10mg.kg PO 7d)	anti-rsPilA	6	999	0.444926401	0.059	0.530
A.C (2.5mg.kg BID)	A.C (10mg.kg PO 7d)	6	999	0.057605697	0.221	0.864
A.C (2.5mg.kg BID)	A.C (5mg.kg BID)	5	999	13.37896095	0.289	0.864
A.C (2.5mg.kg BID)	Ofloxacin	6	999	0.040266551	0.901	0.983
A.C (2.5mg.kg BID)	Saline	6	999	1.888819549	0.052	0.530
A.C (2.5mg.kg BID)	T.S (15mg.kg @12h)	6	999	1.900478873	0.095	0.530
A.C (2.5mg.kg BID)	T.S (7.5mg.kg @12h)	6	999	0.242603162	0.754	0.969
A.C (2.5mg.kg BID)	anti-IHF	6	999	0.363767697	0.504	0.864
A.C (2.5mg.kg BID)	anti-rsPilA	6	999	0.465283609	0.701	0.951
A.C (5mg.kg BID)	A.C (10mg.kg PO 7d)	5	999	0.368933905	0.613	0.951
A.C (5mg.kg BID)	Ofloxacin	5	999	6.13E-01	0.698	0.951
A.C (5mg.kg BID)	Saline	5	999	6.911681024	0.103	0.530
A.C (5mg.kg BID)	T.S (15mg.kg @12h)	5	999	4.124810251	0.304	0.864
A.C (5mg.kg BID)	T.S (7.5mg.kg @12h)	5	999	0.872556631	0.492	0.864
A.C (5mg.kg BID)	anti-IHF	5	999	2.527368485	0.502	0.864
A.C (5mg.kg BID)	anti-rsPilA	5	999	1.796946729	0.401	0.864
Ofloxacin	Saline	6	999	0.151713203	0.463	0.864
Ofloxacin	T.S (15mg.kg @12h)	6	999	0.391157294	0.664	0.951
Ofloxacin	T.S (7.5mg.kg @12h)	6	999	0.057761245	0.891	0.983
Ofloxacin	anti-IHF	6	999	0.153639647	0.338	0.864
Ofloxacin	anti-rsPilA	6	999	0.072819234	0.784	0.971
Saline	T.S (15mg.kg @12h)	6	999	0.173544118	0.498	0.864
Saline	T.S (7.5mg.kg @12h)	6	999	0.002291923	1.000	1.000
Saline	anti-IHF	6	999	2.61094889	0.045	0.530
Saline	anti-rsPilA	6	999	0.008145213	0.887	0.983
T.S (15mg.kg @12h)	anti-IHF	6	999	2.505240523	0.305	0.864
T.S (15mg.kg @12h)	anti-rsPilA	6	999	0.158856853	0.595	0.951
T.S (7.5mg.kg @12h)	T.S (15mg.kg @12h)	6	999	0.086390825	0.809	0.971
T.S (7.5mg.kg @12h)	anti-IHF	6	999	0.430735752	0.713	0.951
T.S (7.5mg.kg @12h)	anti-rsPilA	6	999	0.000252895	1.000	1.000
anti-IHF	anti-rsPilA	6	999	0.808152857	0.392	0.864

Omnibus PERMDISP Test

p-value 0.703

Supplementary File 1
Beta Diversity Output

NPL Jaccard PERMDISP-2DPT

Pairwise Permdisp Results (999 permutations)

Group 1	Group 2	Sample size	Permutations	F-value	p-value	q-value
A.C (10mg.kg PO 7d)	Ofloxacin	6	999	3.80938014	0.115	0.460
A.C (10mg.kg PO 7d)	Saline	6	999	15.66053808	0.112	0.460
A.C (10mg.kg PO 7d)	T.S (15mg.kg @12h)	6	999	2.750332242	0.097	0.460
A.C (10mg.kg PO 7d)	T.S (7.5mg.kg @12h)	6	999	5.229250033	0.093	0.460
A.C (10mg.kg PO 7d)	anti-IHF	6	999	1.496402119	0.104	0.460
A.C (10mg.kg PO 7d)	anti-rsPilA	6	999	5.962112929	0.098	0.460
A.C (2.5mg.kg BID)	A.C (10mg.kg PO 7d)	6	999	48.11195241	0.049	0.460
A.C (2.5mg.kg BID)	A.C (5mg.kg BID)	5	999	163.0085767	0.128	0.461
A.C (2.5mg.kg BID)	Ofloxacin	6	999	0.030074051	0.798	0.828
A.C (2.5mg.kg BID)	Saline	6	999	1.72097612	0.250	0.643
A.C (2.5mg.kg BID)	T.S (15mg.kg @12h)	6	999	0.111198032	0.805	0.828
A.C (2.5mg.kg BID)	T.S (7.5mg.kg @12h)	6	999	0.436110809	0.493	0.767
A.C (2.5mg.kg BID)	anti-IHF	6	999	1.26655076	0.225	0.643
A.C (2.5mg.kg BID)	anti-rsPilA	6	999	0.058901706	0.700	0.820
A.C (5mg.kg BID)	A.C (10mg.kg PO 7d)	5	999	4.325182034	0.092	0.460
A.C (5mg.kg BID)	Ofloxacin	5	999	4.01E+00	0.291	0.652
A.C (5mg.kg BID)	Saline	5	999	30.26587085	0.112	0.460
A.C (5mg.kg BID)	T.S (15mg.kg @12h)	5	999	2.676981329	0.434	0.710
A.C (5mg.kg BID)	T.S (7.5mg.kg @12h)	5	999	4.934013284	0.308	0.652
A.C (5mg.kg BID)	anti-IHF	5	999	2.526807338	0.382	0.710
A.C (5mg.kg BID)	anti-rsPilA	5	999	7.296932668	0.198	0.643
Ofloxacin	Saline	6	999	0.32312751	0.421	0.710
Ofloxacin	T.S (15mg.kg @12h)	6	999	0.027511469	1.000	1.000
Ofloxacin	T.S (7.5mg.kg @12h)	6	999	0.139554472	0.694	0.820
Ofloxacin	anti-IHF	6	999	0.688312324	0.287	0.652
Ofloxacin	anti-rsPilA	6	999	0.079605644	0.801	0.828
Saline	T.S (15mg.kg @12h)	6	999	0.394776009	0.340	0.680
Saline	T.S (7.5mg.kg @12h)	6	999	1.025896835	0.428	0.710
Saline	anti-IHF	6	999	0.31097546	0.511	0.767
Saline	anti-rsPilA	6	999	0.13121745	0.706	0.820
T.S (15mg.kg @12h)	anti-IHF	6	999	0.72234544	0.592	0.782
T.S (15mg.kg @12h)	anti-rsPilA	6	999	0.167908175	0.603	0.782
T.S (7.5mg.kg @12h)	T.S (15mg.kg @12h)	6	999	0.024798345	0.802	0.828
T.S (7.5mg.kg @12h)	anti-IHF	6	999	1.437064667	0.250	0.643
T.S (7.5mg.kg @12h)	anti-rsPilA	6	999	0.490488667	0.608	0.782
anti-IHF	anti-rsPilA	6	999	0.502717591	0.600	0.782

Omnibus PERMDISP Test

p-value 0.102

Supplementary File 1
Beta Diversity Output

NPL Jaccard PERMDISP-5DPT

Pairwise Permdisp Results (999 permutations)

Group 1	Group 2	Sample size	Permutations	F-value	p-value	q-value
A.C (10mg.kg PO 7d)	Ofloxacin	6	999	0.272723609	0.650	1.000
A.C (10mg.kg PO 7d)	Saline	6	999	0.058912331	0.689	1.000
A.C (10mg.kg PO 7d)	T.S (15mg.kg @12h)	6	999	0.025730621	0.597	1.000
A.C (10mg.kg PO 7d)	T.S (7.5mg.kg @12h)	6	999	0.019495165	0.900	1.000
A.C (10mg.kg PO 7d)	anti-IHF	6	999	0.088394755	0.782	1.000
A.C (10mg.kg PO 7d)	anti-rsPilA	5	999	0.540109289	0.681	1.000
A.C (2.5mg.kg BID)	A.C (10mg.kg PO 7d)	6	999	0.069620734	0.810	1.000
A.C (2.5mg.kg BID)	A.C (5mg.kg BID)	5	999	1.991348236	0.401	1.000
A.C (2.5mg.kg BID)	Ofloxacin	6	999	0.809240329	0.576	1.000
A.C (2.5mg.kg BID)	Saline	6	999	0.208354983	0.695	1.000
A.C (2.5mg.kg BID)	T.S (15mg.kg @12h)	6	999	1.96575E-05	1.000	1.000
A.C (2.5mg.kg BID)	T.S (7.5mg.kg @12h)	6	999	0.202593414	0.748	1.000
A.C (2.5mg.kg BID)	anti-IHF	6	999	0.003011564	0.898	1.000
A.C (2.5mg.kg BID)	anti-rsPilA	5	999	0.549669309	0.465	1.000
A.C (5mg.kg BID)	A.C (10mg.kg PO 7d)	5	999	1.386552685	0.420	1.000
A.C (5mg.kg BID)	Ofloxacin	5	999	3.41E+00	0.413	1.000
A.C (5mg.kg BID)	Saline	5	999	0.992686503	0.617	1.000
A.C (5mg.kg BID)	T.S (15mg.kg @12h)	5	999	0.277113483	0.581	1.000
A.C (5mg.kg BID)	T.S (7.5mg.kg @12h)	5	999	2.135063679	0.516	1.000
A.C (5mg.kg BID)	anti-IHF	5	999	1.423040272	0.698	1.000
A.C (5mg.kg BID)	anti-rsPilA	4	999	inf	1.000	1.000
Ofloxacin	Saline	6	999	0.023263304	0.894	1.000
Ofloxacin	T.S (15mg.kg @12h)	6	999	0.272454873	0.620	1.000
Ofloxacin	T.S (7.5mg.kg @12h)	6	999	0.163754659	0.481	1.000
Ofloxacin	anti-IHF	6	999	0.822264664	0.400	1.000
Ofloxacin	anti-rsPilA	5	999	1.889194557	0.482	1.000
Saline	T.S (15mg.kg @12h)	6	999	0.108590163	0.761	1.000
Saline	T.S (7.5mg.kg @12h)	6	999	0.020562113	0.791	1.000
Saline	anti-IHF	6	999	0.229495501	1.000	1.000
Saline	anti-rsPilA	5	999	0.498897605	0.502	1.000
T.S (15mg.kg @12h)	anti-IHF	6	999	0.000588766	1.000	1.000
T.S (15mg.kg @12h)	anti-rsPilA	5	999	0.075531193	0.794	1.000
T.S (7.5mg.kg @12h)	T.S (15mg.kg @12h)	6	999	0.067211564	0.902	1.000
T.S (7.5mg.kg @12h)	anti-IHF	6	999	0.22718189	0.674	1.000
T.S (7.5mg.kg @12h)	anti-rsPilA	5	999	0.945797102	0.514	1.000
anti-IHF	anti-rsPilA	5	999	0.359746335	0.716	1.000

Omnibus PERMDISP Test

p-value 0.905

Supplementary File 1
Beta Diversity Output

NPL Jaccard PERMDISP-7DPT

Pairwise Permdisp Results (999 permutations)

Group 1	Group 2	Sample size	Permutations	F-value	p-value	q-value
A.C (10mg.kg PO 7d)	Ofloxacin	5	999	0.852275953	0.629	1.000
A.C (10mg.kg PO 7d)	Saline	5	999	5.007880644	0.094	1.000
A.C (10mg.kg PO 7d)	T.S (15mg.kg @12h)	4	999	inf	1.000	1.000
A.C (10mg.kg PO 7d)	T.S (7.5mg.kg @12h)	5	999	3.787436386	0.096	1.000
A.C (10mg.kg PO 7d)	anti-IHF	4	999	inf	1.000	1.000
A.C (10mg.kg PO 7d)	anti-rsPilA	4	999	inf	0.672	1.000
A.C (2.5mg.kg BID)	A.C (10mg.kg PO 7d)	5	999	703.151154	0.100	1.000
A.C (2.5mg.kg BID)	A.C (5mg.kg BID)	4	999	5249.199299	0.248	1.000
A.C (2.5mg.kg BID)	Ofloxacin	6	999	0.059424614	0.892	1.000
A.C (2.5mg.kg BID)	Saline	6	999	1.029685593	0.288	1.000
A.C (2.5mg.kg BID)	T.S (15mg.kg @12h)	5	999	7.969996322	0.301	1.000
A.C (2.5mg.kg BID)	T.S (7.5mg.kg @12h)	6	999	0.468307029	0.805	1.000
A.C (2.5mg.kg BID)	anti-IHF	5	999	352.2878421	0.208	1.000
A.C (2.5mg.kg BID)	anti-rsPilA	5	999	95.45110298	0.112	1.000
A.C (5mg.kg BID)	A.C (10mg.kg PO 7d)	3	999	inf	1.000	1.000
A.C (5mg.kg BID)	Ofloxacin	4	999	4.53E+00	1.000	1.000
A.C (5mg.kg BID)	Saline	4	999	19.99578667	0.739	1.000
A.C (5mg.kg BID)	T.S (15mg.kg @12h)	3	999	inf	1.000	1.000
A.C (5mg.kg BID)	T.S (7.5mg.kg @12h)	4	999	17.72090232	0.746	1.000
A.C (5mg.kg BID)	anti-IHF	3	999	inf	1.000	1.000
A.C (5mg.kg BID)	anti-rsPilA	3	999	inf	1.000	1.000
Ofloxacin	Saline	6	999	0.058265148	0.667	1.000
Ofloxacin	T.S (15mg.kg @12h)	5	999	0.012259183	1.000	1.000
Ofloxacin	T.S (7.5mg.kg @12h)	6	999	0.009828325	0.697	1.000
Ofloxacin	anti-IHF	5	999	0.502177461	1.000	1.000
Ofloxacin	anti-rsPilA	5	999	0.211084013	0.516	1.000
Saline	T.S (15mg.kg @12h)	5	999	0.400363667	0.901	1.000
Saline	T.S (7.5mg.kg @12h)	6	999	0.048031303	0.805	1.000
Saline	anti-IHF	5	999	3.290538227	0.331	1.000
Saline	anti-rsPilA	5	999	1.746784327	0.511	1.000
T.S (15mg.kg @12h)	anti-IHF	4	999	inf	1.000	1.000
T.S (15mg.kg @12h)	anti-rsPilA	4	999	inf	1.000	1.000
T.S (7.5mg.kg @12h)	T.S (15mg.kg @12h)	5	999	0.144517658	0.894	1.000
T.S (7.5mg.kg @12h)	anti-IHF	5	999	2.34890942	0.364	1.000
T.S (7.5mg.kg @12h)	anti-rsPilA	5	999	1.107306252	0.412	1.000
anti-IHF	anti-rsPilA	4	999	inf	1.000	1.000

Omnibus PERMDISP Test

p-value 0.786

Supplementary File 1
Beta Diversity Output

NPL Jaccard PERMDISP-9DPT

Pairwise Permdisp Results (999 permutations)

Group 1	Group 2	Sample size	Permutations	F-value	p-value	q-value
A.C (10mg.kg PO 7d)	Ofloxacin	4	999	inf	1.000	1.000
A.C (10mg.kg PO 7d)	Saline	4	999	inf	1.000	1.000
A.C (10mg.kg PO 7d)	T.S (15mg.kg @12h)	5	999	9.07026569	0.334	1.000
A.C (10mg.kg PO 7d)	T.S (7.5mg.kg @12h)	5	999	7.763278273	0.295	1.000
A.C (10mg.kg PO 7d)	anti-IHF	5	999	58.11585083	0.098	1.000
A.C (10mg.kg PO 7d)	anti-rsPilA	4	999	inf	1.000	1.000
A.C (2.5mg.kg BID)	A.C (10mg.kg PO 7d)	5	999	9.452938696	0.094	1.000
A.C (2.5mg.kg BID)	A.C (5mg.kg BID)	5	999	0.436885547	0.504	1.000
A.C (2.5mg.kg BID)	Ofloxacin	5	999	0.21209791	0.789	1.000
A.C (2.5mg.kg BID)	Saline	5	999	2.085622587	0.390	1.000
A.C (2.5mg.kg BID)	T.S (15mg.kg @12h)	6	999	0.807459274	0.562	1.000
A.C (2.5mg.kg BID)	T.S (7.5mg.kg @12h)	6	999	0.399755594	0.797	1.000
A.C (2.5mg.kg BID)	anti-IHF	6	999	0.046211325	0.805	1.000
A.C (2.5mg.kg BID)	anti-rsPilA	5	999	4.289317351	0.276	1.000
A.C (5mg.kg BID)	A.C (10mg.kg PO 7d)	4	999	inf	0.835	1.000
A.C (5mg.kg BID)	Ofloxacin	4	999	inf	0.819	1.000
A.C (5mg.kg BID)	Saline	4	999	inf	1.000	1.000
A.C (5mg.kg BID)	T.S (15mg.kg @12h)	5	999	1.730070891	0.506	1.000
A.C (5mg.kg BID)	T.S (7.5mg.kg @12h)	5	999	1.146363272	0.494	1.000
A.C (5mg.kg BID)	anti-IHF	5	999	3.887644509	0.419	1.000
A.C (5mg.kg BID)	anti-rsPilA	4	999	inf	1.000	1.000
Ofloxacin	Saline	4	999	inf	1.000	1.000
Ofloxacin	T.S (15mg.kg @12h)	5	999	0.277810278	0.717	1.000
Ofloxacin	T.S (7.5mg.kg @12h)	5	999	0.074807776	0.688	1.000
Ofloxacin	anti-IHF	5	999	0.428274923	0.811	1.000
Ofloxacin	anti-rsPilA	4	999	inf	1.000	1.000
Saline	T.S (15mg.kg @12h)	5	999	3.481143397	0.299	1.000
Saline	T.S (7.5mg.kg @12h)	5	999	2.648364499	0.509	1.000
Saline	anti-IHF	5	999	14.48290937	0.313	1.000
Saline	anti-rsPilA	4	999	inf	1.000	1.000
T.S (15mg.kg @12h)	anti-IHF	6	999	0.800330668	0.484	1.000
T.S (15mg.kg @12h)	anti-rsPilA	5	999	5.319440129	0.073	1.000
T.S (7.5mg.kg @12h)	T.S (15mg.kg @12h)	6	999	0.055500864	0.664	1.000
T.S (7.5mg.kg @12h)	anti-IHF	6	999	0.340215314	0.607	1.000
T.S (7.5mg.kg @12h)	anti-rsPilA	5	999	4.297244469	0.301	1.000
anti-IHF	anti-rsPilA	5	999	27.81072197	0.112	1.000

Omnibus PERMDISP Test

p-value 0.164

Supplementary File 2
Longitudinal Pairwise-Distances and Pairwise-Differences

Fecal Weighted UniFrac Pairwise-Distances

Baseline - 2DPT

#SampleID	SubjectID	Distance	Group
3AF2	Chinch3	0.392	AC-PO7d
2AF2	Chinch2	0.464	AC-PO7d
22F1	Chinch22	0.457	AC-10mg/kg
20F1	Chinch20	0.344	AC-5mg/kg
23F1	Chinch23	0.213	AC-5mg/kg
17F1	Chinch17	0.071	anti-rsPilA
13F1	Chinch13	0.132	anti-rsPilA
11F1	Chinch11	0.257	anti-rsPilA
19F1	Chinch19	0.159	anti-tip-chimer
21F1	Chinch21	0.272	anti-tip-chimer
27F1	Chinch27	0.155	anti-tip-chimer
7F1	Chinch7	0.360	Ofloxacin
4F1	Chinch4	0.385	Ofloxacin
16F1	Chinch16	0.342	TS-30mg/kg
12F1	Chinch12	0.448	TS-30mg/kg
10F1	Chinch10	0.273	TS-30mg/kg
14F1	Chinch14	0.176	TS-15mg/kg
18F1	Chinch18	0.270	TS-15mg/kg
15F1	Chinch15	0.278	TS-15mg/kg
6F1	Chinch6	0.191	Saline
9F1	Chinch9	0.223	Saline
8F1	Chinch8	0.163	Saline

Supplementary File 2
Longitudinal Pairwise-Distances and Pairwise-Differences

Fecal Weighted UniFrac Pairwise-Distances
Baseline - 5DPT

#SampleID	SubjectID	Distance	Group
2AF5	Chinch2	0.337	AC-PO7d
3AF5	Chinch3	0.347	AC-PO7d
1AF5	Chinch1	0.236	AC-PO7d
25F2	Chinch25	0.529	AC-10mg/kg
22F2	Chinch22	0.586	AC-10mg/kg
23F2	Chinch23	0.568	AC-5mg/kg
20F2	Chinch20	0.477	AC-5mg/kg
11F2	Chinch11	0.170	anti-rsPilA
17F2	Chinch17	0.134	anti-rsPilA
27F2	Chinch27	0.149	anti-tip-chimer
19F2	Chinch19	0.269	anti-tip-chimer
21F2	Chinch21	0.288	anti-tip-chimer
4F2	Chinch4	0.498	Ofloxacin
5F2	Chinch5	0.209	Ofloxacin
7F2	Chinch7	0.188	Ofloxacin
16F2	Chinch16	0.334	TS-30mg/kg
10F2	Chinch10	0.227	TS-30mg/kg
12F2	Chinch12	0.559	TS-30mg/kg
18F2	Chinch18	0.197	TS-15mg/kg
14F2	Chinch14	0.254	TS-15mg/kg
15F2	Chinch15	0.214	TS-15mg/kg
8F2	Chinch8	0.165	Saline
9F2	Chinch9	0.250	Saline
6F2	Chinch6	0.169	Saline

Supplementary File 2
Longitudinal Pairwise-Distances and Pairwise-Differences

Fecal Weighted UniFrac Pairwise-Distances
Baseline - 7DPT

#SampleID	SubjectID	Distance	Group
2AF7	Chinch2	0.329	AC-PO7d
1AF7	Chinch1	0.411	AC-PO7d
3AF7	Chinch3	0.440	AC-PO7d
25F3	Chinch25	0.438	AC-10mg/kg
23F3	Chinch23	0.488	AC-5mg/kg
20F3	Chinch20	0.359	AC-5mg/kg
17F3	Chinch17	0.154	anti-rsPilA
11F3	Chinch11	0.139	anti-rsPilA
19F3	Chinch19	0.175	anti-tip-chimer
21F3	Chinch21	0.361	anti-tip-chimer
5F3	Chinch5	0.277	Ofloxacin
4F3	Chinch4	0.482	Ofloxacin
7F3	Chinch7	0.199	Ofloxacin
10F3	Chinch10	0.220	TS-30mg/kg
12F3	Chinch12	0.586	TS-30mg/kg
16F3	Chinch16	0.263	TS-30mg/kg
18F3	Chinch18	0.265	TS-15mg/kg
14F3	Chinch14	0.245	TS-15mg/kg
15F3	Chinch15	0.262	TS-15mg/kg
8F3	Chinch8	0.268	Saline
6F3	Chinch6	0.174	Saline

Supplementary File 2
Longitudinal Pairwise-Distances and Pairwise-Differences

Fecal Weighted UniFrac Pairwise-Distances

Baseline - 9DPT

#SampleID	SubjectID	Distance	Group
2AF9	Chinch2	0.296	AC-PO7d
3AF9	Chinch3	0.394	AC-PO7d
22F4	Chinch22	0.390	AC-10mg/kg
25F4	Chinch25	0.388	AC-10mg/kg
23F4	Chinch23	0.443	AC-5mg/kg
20F4	Chinch20	0.402	AC-5mg/kg
17F4	Chinch17	0.224	anti-rsPilA
11F4	Chinch11	0.138	anti-rsPilA
19F4	Chinch19	0.145	anti-tip-chimer
21F4	Chinch21	0.283	anti-tip-chimer
27F4	Chinch27	0.184	anti-tip-chimer
4F4	Chinch4	0.534	Ofloxacin
7F4	Chinch7	0.191	Ofloxacin
5F4	Chinch5	0.268	Ofloxacin
12F4	Chinch12	0.498	TS-30mg/kg
10F4	Chinch10	0.203	TS-30mg/kg
16F4	Chinch16	0.300	TS-30mg/kg
15F4	Chinch15	0.220	TS-15mg/kg
18F4	Chinch18	0.225	TS-15mg/kg
14F4	Chinch14	0.196	TS-15mg/kg
8F4	Chinch8	0.200	Saline

Supplementary File 2
Longitudinal Pairwise-Distances and Pairwise-Differences

Fecal Weighted UniFrac Pairwise-Distance Mann-Whitney Pairwise Comparisons
Baseline - 2DPT

Group A	Group B	Mann-Whitney U	P-value	FDR P-value
AC-PO7d	AC-5mg/kg	4	0.333	0.626
AC-PO7d	AC-10mg/kg	1	1.000	1.000
AC-PO7d	Ofloxacin	4	0.333	0.626
AC-PO7d	Saline	6	0.200	0.554
AC-PO7d	TS-30mg/kg	5	0.400	0.626
AC-PO7d	TS-15mg/kg	6	0.200	0.554
AC-PO7d	anti-tip-chimer	6	0.200	0.554
AC-PO7d	anti-rsPilA	6	0.200	0.554
AC-5mg/kg	AC-10mg/kg	0	0.667	0.788
AC-5mg/kg	Ofloxacin	0	0.333	0.626
AC-5mg/kg	Saline	5	0.400	0.626
AC-10mg/kg	Ofloxacin	2	0.667	0.788
AC-10mg/kg	Saline	3	0.500	0.643
Ofloxacin	Saline	6	0.200	0.554
TS-30mg/kg	AC-5mg/kg	4	0.800	0.823
TS-30mg/kg	AC-10mg/kg	0	0.500	0.643
TS-30mg/kg	Ofloxacin	2	0.800	0.823
TS-30mg/kg	Saline	9	0.100	0.554
TS-30mg/kg	TS-15mg/kg	8	0.200	0.554
TS-30mg/kg	anti-tip-chimer	9	0.100	0.554
TS-30mg/kg	anti-rsPilA	9	0.100	0.554
TS-15mg/kg	AC-5mg/kg	2	0.800	0.823
TS-15mg/kg	AC-10mg/kg	0	0.500	0.643
TS-15mg/kg	Ofloxacin	0	0.200	0.554
TS-15mg/kg	Saline	7	0.400	0.626
TS-15mg/kg	anti-tip-chimer	7	0.400	0.626
anti-tip-chimer	AC-5mg/kg	1	0.400	0.626
anti-tip-chimer	AC-10mg/kg	0	0.500	0.643
anti-tip-chimer	Ofloxacin	0	0.200	0.554
anti-tip-chimer	Saline	3	0.700	0.788
anti-rsPilA	AC-5mg/kg	1	0.400	0.626
anti-rsPilA	AC-10mg/kg	0	0.500	0.643
anti-rsPilA	Ofloxacin	0	0.200	0.554
anti-rsPilA	Saline	3	0.700	0.788
anti-rsPilA	TS-15mg/kg	1	0.200	0.554
anti-rsPilA	anti-tip-chimer	2	0.400	0.626

Kruskal-Wallis (Omnibus Test)

**H**	16.233
**p-value**	0.039

Supplementary File 2
Longitudinal Pairwise-Distances and Pairwise-Differences

Fecal Weighted UniFrac Pairwise-Distance Mann-Whitney Pairwise Comparisons
Baseline - 5DPT

Group A	Group B	Mann-Whitney U	P-value	FDR P-value
AC-PO7d	AC-5mg/kg	0	0.200	0.424
AC-PO7d	AC-10mg/kg	0	0.200	0.424
AC-PO7d	Ofloxacin	6	0.700	0.788
AC-PO7d	Saline	8	0.200	0.424
AC-PO7d	TS-30mg/kg	5	1.000	1.000
AC-PO7d	TS-15mg/kg	8	0.200	0.424
AC-PO7d	anti-tip-chimer	7	0.400	0.514
AC-PO7d	anti-rsPilA	6	0.200	0.424
AC-5mg/kg	AC-10mg/kg	1	0.667	0.788
AC-5mg/kg	Ofloxacin	5	0.400	0.514
AC-5mg/kg	Saline	6	0.200	0.424
AC-10mg/kg	Ofloxacin	6	0.200	0.424
AC-10mg/kg	Saline	6	0.200	0.424
Ofloxacin	Saline	7	0.400	0.514
TS-30mg/kg	AC-5mg/kg	1	0.400	0.514
TS-30mg/kg	AC-10mg/kg	1	0.400	0.514
TS-30mg/kg	Ofloxacin	7	0.400	0.514
TS-30mg/kg	Saline	8	0.200	0.424
TS-30mg/kg	TS-15mg/kg	8	0.200	0.424
TS-30mg/kg	anti-tip-chimer	7	0.400	0.514
TS-30mg/kg	anti-rsPilA	6	0.200	0.424
TS-15mg/kg	AC-5mg/kg	0	0.200	0.424
TS-15mg/kg	AC-10mg/kg	0	0.200	0.424
TS-15mg/kg	Ofloxacin	5	1.000	1.000
TS-15mg/kg	Saline	7	0.400	0.514
TS-15mg/kg	anti-tip-chimer	3	0.700	0.788
anti-tip-chimer	AC-5mg/kg	0	0.200	0.424
anti-tip-chimer	AC-10mg/kg	0	0.200	0.424
anti-tip-chimer	Ofloxacin	4	1.000	1.000
anti-tip-chimer	Saline	6	0.700	0.788
anti-rsPilA	AC-5mg/kg	0	0.333	0.514
anti-rsPilA	AC-10mg/kg	0	0.333	0.514
anti-rsPilA	Ofloxacin	0	0.200	0.424
anti-rsPilA	Saline	2	0.800	0.873
anti-rsPilA	TS-15mg/kg	0	0.200	0.424
anti-rsPilA	anti-tip-chimer	1	0.400	0.514

Kruskal-Wallis (Omnibus Test)

**H**	14.843
**p-value**	0.062

Fecal Weighted UniFrac Pairwise-Distance Mann-Whitney Pairwise Comparisons
Baseline - 7DPT

Group A	Group B	Mann-Whitney U	P-value	FDR P-value
AC-PO7d	AC-5mg/kg	2	0.800	1.000
AC-PO7d	AC-10mg/kg	1	1.000	1.000
AC-PO7d	Ofloxacin	6	0.700	1.000
AC-PO7d	Saline	6	0.200	1.000
AC-PO7d	TS-30mg/kg	6	0.700	1.000
AC-PO7d	TS-15mg/kg	9	0.100	1.000
AC-PO7d	anti-tip-chimer	5	0.400	1.000
AC-PO7d	anti-rsPilA	6	0.200	1.000
AC-5mg/kg	AC-10mg/kg	1	1.000	1.000
AC-5mg/kg	Ofloxacin	5	0.400	1.000
AC-5mg/kg	Saline	4	0.333	1.000
AC-10mg/kg	Ofloxacin	2	1.000	1.000
AC-10mg/kg	Saline	2	0.667	1.000
Ofloxacin	Saline	5	0.400	1.000
TS-30mg/kg	AC-5mg/kg	2	0.800	1.000
TS-30mg/kg	AC-10mg/kg	1	1.000	1.000
TS-30mg/kg	Ofloxacin	5	1.000	1.000
TS-30mg/kg	Saline	4	0.800	1.000
TS-30mg/kg	TS-15mg/kg	5	1.000	1.000
TS-30mg/kg	anti-tip-chimer	4	0.800	1.000
TS-30mg/kg	anti-rsPilA	6	0.200	1.000
TS-15mg/kg	AC-5mg/kg	0	0.200	1.000
TS-15mg/kg	AC-10mg/kg	0	0.500	1.000
TS-15mg/kg	Ofloxacin	3	0.700	1.000
TS-15mg/kg	Saline	3	1.000	1.000
TS-15mg/kg	anti-tip-chimer	3	1.000	1.000
anti-tip-chimer	AC-5mg/kg	1	0.667	1.000
anti-tip-chimer	AC-10mg/kg	0	0.667	1.000
anti-tip-chimer	Ofloxacin	2	0.800	1.000
anti-tip-chimer	Saline	3	0.667	1.000
anti-rsPilA	AC-5mg/kg	0	0.333	1.000
anti-rsPilA	AC-10mg/kg	0	0.667	1.000
anti-rsPilA	Ofloxacin	0	0.200	1.000
anti-rsPilA	Saline	0	0.333	1.000
anti-rsPilA	TS-15mg/kg	0	0.200	1.000
anti-rsPilA	anti-tip-chimer	0	0.333	1.000

Kruskal-Wallis (Omnibus Test)

**H**	10.848
**p-value**	0.210

Supplementary File 2
Longitudinal Pairwise-Distances and Pairwise-Differences

Fecal Weighted UniFrac Pairwise-Distance Mann-Whitney Pairwise Comparisons

Baseline - 9DPT

Group A	Group B	Mann-Whitney U	P-value	FDR P-value
AC-PO7d	AC-5mg/kg	0	0.333	0.900
AC-PO7d	AC-10mg/kg	2	1.000	1.000
AC-PO7d	Ofloxacin	4	0.800	0.993
AC-PO7d	Saline	2	0.667	0.993
AC-PO7d	TS-30mg/kg	3	1.000	1.000
AC-PO7d	TS-15mg/kg	6	0.200	0.900
AC-PO7d	anti-tip-chimer	6	0.200	0.900
AC-PO7d	anti-rsPilA	4	0.333	0.900
AC-5mg/kg	AC-10mg/kg	4	0.333	0.900
AC-5mg/kg	Ofloxacin	4	0.800	0.993
AC-5mg/kg	Saline	2	0.667	0.993
AC-10mg/kg	Ofloxacin	4	0.800	0.993
AC-10mg/kg	Saline	2	0.667	0.993
Ofloxacin	Saline	2	1.000	1.000
TS-30mg/kg	AC-5mg/kg	2	0.800	0.993
TS-30mg/kg	AC-10mg/kg	2	0.800	0.993
TS-30mg/kg	Ofloxacin	5	1.000	1.000
TS-30mg/kg	Saline	3	0.500	0.993
TS-30mg/kg	TS-15mg/kg	7	0.400	0.900
TS-30mg/kg	anti-tip-chimer	8	0.200	0.900
TS-30mg/kg	anti-rsPilA	5	0.400	0.900
TS-15mg/kg	AC-5mg/kg	0	0.200	0.900
TS-15mg/kg	AC-10mg/kg	0	0.200	0.900
TS-15mg/kg	Ofloxacin	3	0.700	0.993
TS-15mg/kg	Saline	2	1.000	1.000
TS-15mg/kg	anti-tip-chimer	6	0.700	0.993
anti-tip-chimer	AC-5mg/kg	0	0.200	0.900
anti-tip-chimer	AC-10mg/kg	0	0.200	0.900
anti-tip-chimer	Ofloxacin	2	0.400	0.900
anti-tip-chimer	Saline	1	1.000	1.000
anti-rsPilA	AC-5mg/kg	0	0.333	0.900
anti-rsPilA	AC-10mg/kg	0	0.333	0.900
anti-rsPilA	Ofloxacin	1	0.400	0.900
anti-rsPilA	Saline	1	1.000	1.000
anti-rsPilA	TS-15mg/kg	2	0.800	0.993
anti-rsPilA	anti-tip-chimer	2	0.800	0.993

Kruskal-Wallis (Omnibus Test)

**H**	11.039
**p-value**	0.200

Supplementary File 2
Longitudinal Pairwise-Distances and Pairwise-Differences

Fecal Bray-Curtis Pairwise-Distances

Baseline - 2DPT

#SampleID	SubjectID	Distance	Group
2AF2	Chinch2	0.977	AC-PO7d
3AF2	Chinch3	0.870	AC-PO7d
10F1	Chinch10	0.450	TS-30mg/kg
16F1	Chinch16	0.717	TS-30mg/kg
12F1	Chinch12	0.699	TS-30mg/kg
11F1	Chinch11	0.454	anti-rsPilA
13F1	Chinch13	0.531	anti-rsPilA
17F1	Chinch17	0.258	anti-rsPilA
18F1	Chinch18	0.530	TS-15mg/kg
15F1	Chinch15	0.699	TS-15mg/kg
14F1	Chinch14	0.545	TS-15mg/kg
19F1	Chinch19	0.368	anti-tip-chimer
27F1	Chinch27	0.611	anti-tip-chimer
21F1	Chinch21	0.549	anti-tip-chimer
20F1	Chinch20	0.556	AC-5mg/kg
23F1	Chinch23	0.324	AC-5mg/kg
22F1	Chinch22	0.704	AC-10mg/kg
7F1	Chinch7	0.769	Ofloxacin
4F1	Chinch4	0.629	Ofloxacin
8F1	Chinch8	0.557	Saline
6F1	Chinch6	0.511	Saline
9F1	Chinch9	0.482	Saline

Supplementary File 2
Longitudinal Pairwise-Distances and Pairwise-Differences

Fecal Bray-Curtis Pairwise-Distances

Baseline - 5DPT

#SampleID	SubjectID	Distance	Group
1AF5	Chinch1	0.662	AC-PO7d
3AF5	Chinch3	0.835	AC-PO7d
2AF5	Chinch2	0.895	AC-PO7d
10F2	Chinch10	0.381	TS-30mg/kg
16F2	Chinch16	0.722	TS-30mg/kg
12F2	Chinch12	0.724	TS-30mg/kg
17F2	Chinch17	0.441	anti-rsPilA
11F2	Chinch11	0.435	anti-rsPilA
15F2	Chinch15	0.690	TS-15mg/kg
14F2	Chinch14	0.603	TS-15mg/kg
18F2	Chinch18	0.485	TS-15mg/kg
19F2	Chinch19	0.497	anti-tip-chimer
27F2	Chinch27	0.734	anti-tip-chimer
21F2	Chinch21	0.625	anti-tip-chimer
20F2	Chinch20	0.929	AC-5mg/kg
23F2	Chinch23	0.959	AC-5mg/kg
22F2	Chinch22	0.992	AC-10mg/kg
25F2	Chinch25	0.937	AC-10mg/kg
5F2	Chinch5	0.510	Ofloxacin
7F2	Chinch7	0.535	Ofloxacin
4F2	Chinch4	0.753	Ofloxacin
9F2	Chinch9	0.541	Saline
6F2	Chinch6	0.466	Saline
8F2	Chinch8	0.582	Saline

Supplementary File 2
Longitudinal Pairwise-Distances and Pairwise-Differences

Fecal Bray-Curtis Pairwise-Distances

Baseline - 7DPT

#SampleID	SubjectID	Distance	Group
1AF7	Chinch1	0.951	AC-PO7d
2AF7	Chinch2	0.923	AC-PO7d
3AF7	Chinch3	0.845	AC-PO7d
12F3	Chinch12	0.722	TS-30mg/kg
10F3	Chinch10	0.522	TS-30mg/kg
16F3	Chinch16	0.592	TS-30mg/kg
11F3	Chinch11	0.410	anti-rsPilA
17F3	Chinch17	0.363	anti-rsPilA
18F3	Chinch18	0.550	TS-15mg/kg
15F3	Chinch15	0.697	TS-15mg/kg
14F3	Chinch14	0.633	TS-15mg/kg
19F3	Chinch19	0.402	anti-tip-chimer
21F3	Chinch21	0.616	anti-tip-chimer
20F3	Chinch20	0.891	AC-5mg/kg
23F3	Chinch23	0.928	AC-5mg/kg
25F3	Chinch25	0.890	AC-10mg/kg
4F3	Chinch4	0.718	Ofloxacin
5F3	Chinch5	0.595	Ofloxacin
7F3	Chinch7	0.557	Ofloxacin
6F3	Chinch6	0.545	Saline
8F3	Chinch8	0.829	Saline

Supplementary File 2
Longitudinal Pairwise-Distances and Pairwise-Differences

Fecal Bray-Curtis Pairwise-Distances

Baseline - 9DPT

#SampleID	SubjectID	Distance	Group
2AF9	Chinch2	0.814	AC-PO7d
3AF9	Chinch3	0.851	AC-PO7d
12F4	Chinch12	0.696	TS-30mg/kg
16F4	Chinch16	0.578	TS-30mg/kg
10F4	Chinch10	0.521	TS-30mg/kg
11F4	Chinch11	0.411	anti-rsPilA
17F4	Chinch17	0.536	anti-rsPilA
18F4	Chinch18	0.486	TS-15mg/kg
15F4	Chinch15	0.649	TS-15mg/kg
14F4	Chinch14	0.650	TS-15mg/kg
19F4	Chinch19	0.371	anti-tip-chimer
27F4	Chinch27	0.691	anti-tip-chimer
21F4	Chinch21	0.614	anti-tip-chimer
23F4	Chinch23	0.875	AC-5mg/kg
20F4	Chinch20	0.894	AC-5mg/kg
25F4	Chinch25	0.890	AC-10mg/kg
22F4	Chinch22	0.712	AC-10mg/kg
4F4	Chinch4	0.850	Ofloxacin
5F4	Chinch5	0.617	Ofloxacin
7F4	Chinch7	0.520	Ofloxacin
8F4	Chinch8	0.600	Saline

Supplementary File 2
Longitudinal Pairwise-Distances and Pairwise-Differences

Fecal Bray-Curtis Pairwise-Distance Mann-Whitney Pairwise Comparisons

Baseline - 2DPT

Group A	Group B	Mann-Whitney U	P-value	FDR P-value
AC-PO7d	AC-5mg/kg	4	0.333	0.758
AC-PO7d	AC-10mg/kg	2	0.667	0.929
AC-PO7d	Ofloxacin	4	0.333	0.758
AC-PO7d	Saline	6	0.200	0.758
AC-PO7d	TS-30mg/kg	6	0.200	0.758
AC-PO7d	TS-15mg/kg	6	0.200	0.758
AC-PO7d	anti-tip-chimer	6	0.200	0.758
AC-PO7d	anti-rsPilA	6	0.200	0.758
AC-5mg/kg	AC-10mg/kg	0	0.667	0.929
AC-5mg/kg	Ofloxacin	0	0.333	0.758
AC-5mg/kg	Saline	2	0.800	0.929
AC-10mg/kg	Ofloxacin	1	1.000	1.000
AC-10mg/kg	Saline	3	0.500	0.783
Ofloxacin	Saline	6	0.200	0.758
TS-30mg/kg	AC-5mg/kg	5	0.400	0.758
TS-30mg/kg	AC-10mg/kg	1	1.000	1.000
TS-30mg/kg	Ofloxacin	2	0.800	0.929
TS-30mg/kg	Saline	6	0.700	0.929
TS-30mg/kg	TS-15mg/kg	5	1.000	1.000
TS-30mg/kg	anti-tip-chimer	7	0.400	0.758
TS-30mg/kg	anti-rsPilA	7	0.400	0.758
TS-15mg/kg	AC-5mg/kg	4	0.800	0.929
TS-15mg/kg	AC-10mg/kg	0	0.500	0.783
TS-15mg/kg	Ofloxacin	1	0.400	0.758
TS-15mg/kg	Saline	7	0.400	0.758
TS-15mg/kg	anti-tip-chimer	5	1.000	1.000
anti-tip-chimer	AC-5mg/kg	4	0.800	0.929
anti-tip-chimer	AC-10mg/kg	0	0.500	0.783
anti-tip-chimer	Ofloxacin	0	0.200	0.758
anti-tip-chimer	Saline	5	1.000	1.000
anti-rsPilA	AC-5mg/kg	2	0.800	0.929
anti-rsPilA	AC-10mg/kg	0	0.500	0.783
anti-rsPilA	Ofloxacin	0	0.200	0.758
anti-rsPilA	Saline	2	0.400	0.758
anti-rsPilA	TS-15mg/kg	1	0.200	0.758
anti-rsPilA	anti-tip-chimer	2	0.400	0.758

Kruskal-Wallis (Omnibus Test)

**H**	12.486
**p-value**	0.131

Supplementary File 2
Longitudinal Pairwise-Distances and Pairwise-Differences

Fecal Bray-Curtis Pairwise-Distance Mann-Whitney Pairwise Comparisons

Baseline - 5DPT

Group A	Group B	Mann-Whitney U	P-value	FDR P-value
AC-PO7d	AC-5mg/kg	0	0.200	0.343
AC-PO7d	AC-10mg/kg	0	0.200	0.343
AC-PO7d	Ofloxacin	8	0.200	0.343
AC-PO7d	Saline	9	0.100	0.343
AC-PO7d	TS-30mg/kg	7	0.400	0.554
AC-PO7d	TS-15mg/kg	8	0.200	0.343
AC-PO7d	anti-tip-chimer	8	0.200	0.343
AC-PO7d	anti-rsPilA	6	0.200	0.343
AC-5mg/kg	AC-10mg/kg	1	0.667	0.840
AC-5mg/kg	Ofloxacin	6	0.200	0.343
AC-5mg/kg	Saline	6	0.200	0.343
AC-10mg/kg	Ofloxacin	6	0.200	0.343
AC-10mg/kg	Saline	6	0.200	0.343
Ofloxacin	Saline	5	1.000	1.000
TS-30mg/kg	AC-5mg/kg	0	0.200	0.343
TS-30mg/kg	AC-10mg/kg	0	0.200	0.343
TS-30mg/kg	Ofloxacin	4	1.000	1.000
TS-30mg/kg	Saline	6	0.700	0.840
TS-30mg/kg	TS-15mg/kg	6	0.700	0.840
TS-30mg/kg	anti-tip-chimer	4	1.000	1.000
TS-30mg/kg	anti-rsPilA	4	0.800	0.929
TS-15mg/kg	AC-5mg/kg	0	0.200	0.343
TS-15mg/kg	AC-10mg/kg	0	0.200	0.343
TS-15mg/kg	Ofloxacin	4	1.000	1.000
TS-15mg/kg	Saline	7	0.400	0.554
TS-15mg/kg	anti-tip-chimer	3	0.700	0.840
anti-tip-chimer	AC-5mg/kg	0	0.200	0.343
anti-tip-chimer	AC-10mg/kg	0	0.200	0.343
anti-tip-chimer	Ofloxacin	4	1.000	1.000
anti-tip-chimer	Saline	7	0.400	0.554
anti-rsPilA	AC-5mg/kg	0	0.333	0.522
anti-rsPilA	AC-10mg/kg	0	0.333	0.522
anti-rsPilA	Ofloxacin	0	0.200	0.343
anti-rsPilA	Saline	0	0.200	0.343
anti-rsPilA	TS-15mg/kg	0	0.200	0.343
anti-rsPilA	anti-tip-chimer	0	0.200	0.343

Kruskal-Wallis (Omnibus Test)

**H**	15.577
**p-value**	0.049

Supplementary File 2
Longitudinal Pairwise-Distances and Pairwise-Differences

Fecal Bray-Curtis Pairwise-Distance Mann-Whitney Pairwise Comparisons

Baseline - 7DPT

Group A	Group B	Mann-Whitney U	P-value	FDR P-value
AC-PO7d	AC-5mg/kg	3	1.000	1.000
AC-PO7d	AC-10mg/kg	2	1.000	1.000
AC-PO7d	Ofloxacin	9	0.100	0.600
AC-PO7d	Saline	6	0.200	0.600
AC-PO7d	TS-30mg/kg	9	0.100	0.600
AC-PO7d	TS-15mg/kg	9	0.100	0.600
AC-PO7d	anti-tip-chimer	6	0.200	0.600
AC-PO7d	anti-rsPilA	6	0.200	0.600
AC-5mg/kg	AC-10mg/kg	2	0.667	0.923
AC-5mg/kg	Ofloxacin	6	0.200	0.600
AC-5mg/kg	Saline	4	0.333	0.750
AC-10mg/kg	Ofloxacin	3	0.500	0.900
AC-10mg/kg	Saline	2	0.667	0.923
Ofloxacin	Saline	3	1.000	1.000
TS-30mg/kg	AC-5mg/kg	0	0.200	0.600
TS-30mg/kg	AC-10mg/kg	0	0.500	0.900
TS-30mg/kg	Ofloxacin	4	1.000	1.000
TS-30mg/kg	Saline	2	0.800	0.993
TS-30mg/kg	TS-15mg/kg	4	1.000	1.000
TS-30mg/kg	anti-tip-chimer	4	0.800	0.993
TS-30mg/kg	anti-rsPilA	6	0.200	0.600
TS-15mg/kg	AC-5mg/kg	0	0.200	0.600
TS-15mg/kg	AC-10mg/kg	0	0.500	0.900
TS-15mg/kg	Ofloxacin	4	1.000	1.000
TS-15mg/kg	Saline	3	1.000	1.000
TS-15mg/kg	anti-tip-chimer	5	0.400	0.847
anti-tip-chimer	AC-5mg/kg	0	0.333	0.750
anti-tip-chimer	AC-10mg/kg	0	0.667	0.923
anti-tip-chimer	Ofloxacin	2	0.800	0.993
anti-tip-chimer	Saline	1	0.667	0.923
anti-rsPilA	AC-5mg/kg	0	0.333	0.750
anti-rsPilA	AC-10mg/kg	0	0.667	0.923
anti-rsPilA	Ofloxacin	0	0.200	0.600
anti-rsPilA	Saline	0	0.333	0.750
anti-rsPilA	TS-15mg/kg	0	0.200	0.600
anti-rsPilA	anti-tip-chimer	1	0.667	0.923

Kruskal-Wallis (Omnibus Test)

**H**	15.100
**p-value**	0.057

Supplementary File 2
Longitudinal Pairwise-Distances and Pairwise-Differences

Fecal Bray-Curtis Pairwise-Distance Mann-Whitney Pairwise Comparisons

Baseline - 9DPT

Group A	Group B	Mann-Whitney U	P-value	FDR P-value
AC-PO7d	AC-5mg/kg	0	0.333	0.758
AC-PO7d	AC-10mg/kg	2	1.000	1.000
AC-PO7d	Ofloxacin	5	0.400	0.758
AC-PO7d	Saline	2	0.667	1.000
AC-PO7d	TS-30mg/kg	6	0.200	0.720
AC-PO7d	TS-15mg/kg	6	0.200	0.720
AC-PO7d	anti-tip-chimer	6	0.200	0.720
AC-PO7d	anti-rsPilA	4	0.333	0.758
AC-5mg/kg	AC-10mg/kg	3	0.667	1.000
AC-5mg/kg	Ofloxacin	6	0.200	0.720
AC-5mg/kg	Saline	2	0.667	1.000
AC-10mg/kg	Ofloxacin	5	0.400	0.758
AC-10mg/kg	Saline	2	0.667	1.000
Ofloxacin	Saline	2	1.000	1.000
TS-30mg/kg	AC-5mg/kg	0	0.200	0.720
TS-30mg/kg	AC-10mg/kg	0	0.200	0.720
TS-30mg/kg	Ofloxacin	4	1.000	1.000
TS-30mg/kg	Saline	1	1.000	1.000
TS-30mg/kg	TS-15mg/kg	5	1.000	1.000
TS-30mg/kg	anti-tip-chimer	5	1.000	1.000
TS-30mg/kg	anti-rsPilA	5	0.400	0.758
TS-15mg/kg	AC-5mg/kg	0	0.200	0.720
TS-15mg/kg	AC-10mg/kg	0	0.200	0.720
TS-15mg/kg	Ofloxacin	4	1.000	1.000
TS-15mg/kg	Saline	2	1.000	1.000
TS-15mg/kg	anti-tip-chimer	5	1.000	1.000
anti-tip-chimer	AC-5mg/kg	0	0.200	0.720
anti-tip-chimer	AC-10mg/kg	0	0.200	0.720
anti-tip-chimer	Ofloxacin	3	0.700	1.000
anti-tip-chimer	Saline	2	1.000	1.000
anti-rsPilA	AC-5mg/kg	0	0.333	0.758
anti-rsPilA	AC-10mg/kg	0	0.333	0.758
anti-rsPilA	Ofloxacin	1	0.400	0.758
anti-rsPilA	Saline	0	0.667	1.000
anti-rsPilA	TS-15mg/kg	1	0.400	0.758
anti-rsPilA	anti-tip-chimer	2	0.800	1.000

Kruskal-Wallis (Omnibus Test)

**H**	12.801
**p-value**	0.119

Supplementary File 2
Longitudinal Pairwise-Distances and Pairwise-Differences

Fecal Jaccard Pairwise-Distances

Baseline - 2DPT

#SampleID	SubjectID	Distance	Group
3AF2	Chinch3	0.927	AC-PO7d
2AF2	Chinch2	0.921	AC-PO7d
12F1	Chinch12	0.685	TS-30mg/kg
16F1	Chinch16	0.695	TS-30mg/kg
10F1	Chinch10	0.479	TS-30mg/kg
11F1	Chinch11	0.605	anti-rsPilA
13F1	Chinch13	0.619	anti-rsPilA
17F1	Chinch17	0.389	anti-rsPilA
15F1	Chinch15	0.798	TS-15mg/kg
14F1	Chinch14	0.650	TS-15mg/kg
18F1	Chinch18	0.665	TS-15mg/kg
27F1	Chinch27	0.668	anti-tip-chimer
19F1	Chinch19	0.578	anti-tip-chimer
21F1	Chinch21	0.674	anti-tip-chimer
23F1	Chinch23	0.541	AC-5mg/kg
20F1	Chinch20	0.649	AC-5mg/kg
22F1	Chinch22	0.782	AC-10mg/kg
7F1	Chinch7	0.767	Ofloxacin
4F1	Chinch4	0.761	Ofloxacin
9F1	Chinch9	0.630	Saline
8F1	Chinch8	0.674	Saline
6F1	Chinch6	0.690	Saline

Supplementary File 2
Longitudinal Pairwise-Distances and Pairwise-Differences

Fecal Jaccard Pairwise-Distances

Baseline - 5DPT

#SampleID	SubjectID	Distance	Group
3AF5	Chinch3	0.887	AC-PO7d
1AF5	Chinch1	0.707	AC-PO7d
2AF5	Chinch2	0.889	AC-PO7d
12F2	Chinch12	0.699	TS-30mg/kg
10F2	Chinch10	0.500	TS-30mg/kg
16F2	Chinch16	0.713	TS-30mg/kg
11F2	Chinch11	0.577	anti-rsPilA
17F2	Chinch17	0.622	anti-rsPilA
18F2	Chinch18	0.605	TS-15mg/kg
15F2	Chinch15	0.774	TS-15mg/kg
14F2	Chinch14	0.650	TS-15mg/kg
21F2	Chinch21	0.689	anti-tip-chimer
19F2	Chinch19	0.614	anti-tip-chimer
27F2	Chinch27	0.737	anti-tip-chimer
23F2	Chinch23	0.944	AC-5mg/kg
20F2	Chinch20	0.911	AC-5mg/kg
22F2	Chinch22	0.968	AC-10mg/kg
25F2	Chinch25	0.875	AC-10mg/kg
4F2	Chinch4	0.830	Ofloxacin
5F2	Chinch5	0.653	Ofloxacin
7F2	Chinch7	0.738	Ofloxacin
9F2	Chinch9	0.684	Saline
6F2	Chinch6	0.712	Saline
8F2	Chinch8	0.699	Saline

Supplementary File 2
Longitudinal Pairwise-Distances and Pairwise-Differences

Fecal Jaccard Pairwise-Distances

Baseline - 7DPT

#SampleID	SubjectID	Distance	Group
2AF7	Chinch2	0.854	AC-PO7d
1AF7	Chinch1	0.942	AC-PO7d
3AF7	Chinch3	0.906	AC-PO7d
16F3	Chinch16	0.690	TS-30mg/kg
10F3	Chinch10	0.613	TS-30mg/kg
12F3	Chinch12	0.742	TS-30mg/kg
11F3	Chinch11	0.578	anti-rsPilA
17F3	Chinch17	0.458	anti-rsPilA
15F3	Chinch15	0.792	TS-15mg/kg
14F3	Chinch14	0.742	TS-15mg/kg
18F3	Chinch18	0.664	TS-15mg/kg
21F3	Chinch21	0.682	anti-tip-chimer
19F3	Chinch19	0.575	anti-tip-chimer
23F3	Chinch23	0.876	AC-5mg/kg
20F3	Chinch20	0.830	AC-5mg/kg
25F3	Chinch25	0.849	AC-10mg/kg
4F3	Chinch4	0.848	Ofloxacin
5F3	Chinch5	0.676	Ofloxacin
7F3	Chinch7	0.665	Ofloxacin
8F3	Chinch8	0.847	Saline
6F3	Chinch6	0.712	Saline

Supplementary File 2
Longitudinal Pairwise-Distances and Pairwise-Differences

Fecal Jaccard Pairwise-Distances

Baseline - 9DPT

#SampleID	SubjectID	Distance	Group
2AF9	Chinch2	0.788	AC-PO7d
3AF9	Chinch3	0.836	AC-PO7d
12F4	Chinch12	0.743	TS-30mg/kg
10F4	Chinch10	0.620	TS-30mg/kg
16F4	Chinch16	0.633	TS-30mg/kg
17F4	Chinch17	0.650	anti-rsPilA
11F4	Chinch11	0.583	anti-rsPilA
18F4	Chinch18	0.575	TS-15mg/kg
15F4	Chinch15	0.773	TS-15mg/kg
14F4	Chinch14	0.709	TS-15mg/kg
19F4	Chinch19	0.566	anti-tip-chimer
27F4	Chinch27	0.760	anti-tip-chimer
21F4	Chinch21	0.702	anti-tip-chimer
20F4	Chinch20	0.837	AC-5mg/kg
23F4	Chinch23	0.813	AC-5mg/kg
25F4	Chinch25	0.854	AC-10mg/kg
22F4	Chinch22	0.740	AC-10mg/kg
7F4	Chinch7	0.667	Ofloxacin
4F4	Chinch4	0.902	Ofloxacin
5F4	Chinch5	0.721	Ofloxacin
8F4	Chinch8	0.668	Saline

Supplementary File 2
Longitudinal Pairwise-Distances and Pairwise-Differences

Fecal Jaccard Pairwise-Distance Mann-Whitney Pairwise Comparisons
Baseline - 2DPT

Group A	Group B	Mann-Whitney U	P-value	FDR P-value
AC-PO7d	AC-5mg/kg	4	0.333	0.758
AC-PO7d	AC-10mg/kg	2	0.667	0.900
AC-PO7d	Ofloxacin	4	0.333	0.758
AC-PO7d	Saline	6	0.200	0.600
AC-PO7d	TS-30mg/kg	6	0.200	0.600
AC-PO7d	TS-15mg/kg	6	0.200	0.600
AC-PO7d	anti-tip-chimer	6	0.200	0.600
AC-PO7d	anti-rsPilA	6	0.200	0.600
AC-5mg/kg	AC-10mg/kg	0	0.667	0.900
AC-5mg/kg	Ofloxacin	0	0.333	0.758
AC-5mg/kg	Saline	1	0.400	0.758
AC-10mg/kg	Ofloxacin	2	0.667	0.900
AC-10mg/kg	Saline	3	0.500	0.783
Ofloxacin	Saline	6	0.200	0.600
TS-30mg/kg	AC-5mg/kg	4	0.800	0.929
TS-30mg/kg	AC-10mg/kg	0	0.500	0.783
TS-30mg/kg	Ofloxacin	0	0.200	0.600
TS-30mg/kg	Saline	5	1.000	1.000
TS-30mg/kg	TS-15mg/kg	4	1.000	1.000
TS-30mg/kg	anti-tip-chimer	6	0.700	0.900
TS-30mg/kg	anti-rsPilA	7	0.400	0.758
TS-15mg/kg	AC-5mg/kg	6	0.200	0.600
TS-15mg/kg	AC-10mg/kg	1	1.000	1.000
TS-15mg/kg	Ofloxacin	2	0.800	0.929
TS-15mg/kg	Saline	5	1.000	1.000
TS-15mg/kg	anti-tip-chimer	5	1.000	1.000
anti-tip-chimer	AC-5mg/kg	5	0.400	0.758
anti-tip-chimer	AC-10mg/kg	0	0.500	0.783
anti-tip-chimer	Ofloxacin	0	0.200	0.600
anti-tip-chimer	Saline	3	0.700	0.900
anti-rsPilA	AC-5mg/kg	2	0.800	0.929
anti-rsPilA	AC-10mg/kg	0	0.500	0.783
anti-rsPilA	Ofloxacin	0	0.200	0.600
anti-rsPilA	Saline	0	0.100	0.600
anti-rsPilA	TS-15mg/kg	0	0.100	0.600
anti-rsPilA	anti-tip-chimer	2	0.400	0.758

Kruskal-Wallis (Omnibus Test)

**H**	14.040
**p-value**	0.081

Supplementary File 2
Longitudinal Pairwise-Distances and Pairwise-Differences

Fecal Jaccard Pairwise-Distance Mann-Whitney Pairwise Comparisons
Baseline - 5DPT

Group A	Group B	Mann-Whitney U	P-value	FDR P-value
AC-PO7d	AC-5mg/kg	0	0.200	0.400
AC-PO7d	AC-10mg/kg	2	0.800	0.960
AC-PO7d	Ofloxacin	7	0.400	0.554
AC-PO7d	Saline	8	0.200	0.400
AC-PO7d	TS-30mg/kg	8	0.200	0.400
AC-PO7d	TS-15mg/kg	8	0.200	0.400
AC-PO7d	anti-tip-chimer	8	0.200	0.400
AC-PO7d	anti-rsPilA	6	0.200	0.400
AC-5mg/kg	AC-10mg/kg	2	1.000	1.000
AC-5mg/kg	Ofloxacin	6	0.200	0.400
AC-5mg/kg	Saline	6	0.200	0.400
AC-10mg/kg	Ofloxacin	6	0.200	0.400
AC-10mg/kg	Saline	6	0.200	0.400
Ofloxacin	Saline	6	0.700	0.900
TS-30mg/kg	AC-5mg/kg	0	0.200	0.400
TS-30mg/kg	AC-10mg/kg	0	0.200	0.400
TS-30mg/kg	Ofloxacin	2	0.400	0.554
TS-30mg/kg	Saline	4	1.000	1.000
TS-30mg/kg	TS-15mg/kg	4	1.000	1.000
TS-30mg/kg	anti-tip-chimer	4	1.000	1.000
TS-30mg/kg	anti-rsPilA	4	0.800	0.960
TS-15mg/kg	AC-5mg/kg	0	0.200	0.400
TS-15mg/kg	AC-10mg/kg	0	0.200	0.400
TS-15mg/kg	Ofloxacin	2	0.400	0.554
TS-15mg/kg	Saline	3	0.700	0.900
TS-15mg/kg	anti-tip-chimer	4	1.000	1.000
anti-tip-chimer	AC-5mg/kg	0	0.200	0.400
anti-tip-chimer	AC-10mg/kg	0	0.200	0.400
anti-tip-chimer	Ofloxacin	2	0.400	0.554
anti-tip-chimer	Saline	4	1.000	1.000
anti-rsPilA	AC-5mg/kg	0	0.333	0.554
anti-rsPilA	AC-10mg/kg	0	0.333	0.554
anti-rsPilA	Ofloxacin	0	0.200	0.400
anti-rsPilA	Saline	0	0.200	0.400
anti-rsPilA	TS-15mg/kg	1	0.400	0.554
anti-rsPilA	anti-tip-chimer	1	0.400	0.554

Kruskal-Wallis (Omnibus Test)

**H**	14.890
**p-value**	0.061

Supplementary File 2
Longitudinal Pairwise-Distances and Pairwise-Differences

Fecal Jaccard Pairwise-Distance Mann-Whitney Pairwise Comparisons

Baseline - 7DPT

Group A	Group B	Mann-Whitney U	P-value	FDR P-value
AC-PO7d	AC-5mg/kg	5	0.400	0.720
AC-PO7d	AC-10mg/kg	3	0.500	0.750
AC-PO7d	Ofloxacin	9	0.100	0.655
AC-PO7d	Saline	6	0.200	0.655
AC-PO7d	TS-30mg/kg	9	0.100	0.655
AC-PO7d	TS-15mg/kg	9	0.100	0.655
AC-PO7d	anti-tip-chimer	6	0.200	0.655
AC-PO7d	anti-rsPilA	6	0.200	0.655
AC-5mg/kg	AC-10mg/kg	1	1.000	1.000
AC-5mg/kg	Ofloxacin	5	0.400	0.720
AC-5mg/kg	Saline	3	0.667	0.828
AC-10mg/kg	Ofloxacin	3	0.500	0.750
AC-10mg/kg	Saline	2	0.667	0.828
Ofloxacin	Saline	2	0.800	0.873
TS-30mg/kg	AC-5mg/kg	0	0.200	0.655
TS-30mg/kg	AC-10mg/kg	0	0.500	0.750
TS-30mg/kg	Ofloxacin	4	1.000	1.000
TS-30mg/kg	Saline	1	0.400	0.720
TS-30mg/kg	TS-15mg/kg	3	0.700	0.840
TS-30mg/kg	anti-tip-chimer	5	0.400	0.720
TS-30mg/kg	anti-rsPilA	6	0.200	0.655
TS-15mg/kg	AC-5mg/kg	0	0.200	0.655
TS-15mg/kg	AC-10mg/kg	0	0.500	0.750
TS-15mg/kg	Ofloxacin	4	1.000	1.000
TS-15mg/kg	Saline	2	0.800	0.873
TS-15mg/kg	anti-tip-chimer	5	0.400	0.720
anti-tip-chimer	AC-5mg/kg	0	0.333	0.720
anti-tip-chimer	AC-10mg/kg	0	0.667	0.828
anti-tip-chimer	Ofloxacin	2	0.800	0.873
anti-tip-chimer	Saline	0	0.333	0.720
anti-rsPilA	AC-5mg/kg	0	0.333	0.720
anti-rsPilA	AC-10mg/kg	0	0.667	0.828
anti-rsPilA	Ofloxacin	0	0.200	0.655
anti-rsPilA	Saline	0	0.333	0.720
anti-rsPilA	TS-15mg/kg	0	0.200	0.655
anti-rsPilA	anti-tip-chimer	1	0.667	0.828

Kruskal-Wallis (Omnibus Test)

**H**	15.385
**p-value**	0.052

Supplementary File 2
Longitudinal Pairwise-Distances and Pairwise-Differences

Fecal Jaccard Pairwise-Distance Mann-Whitney Pairwise Comparisons
Baseline - 9DPT

Group A	Group B	Mann-Whitney U	P-value	FDR P-value
AC-PO7d	AC-5mg/kg	1	0.667	1.000
AC-PO7d	AC-10mg/kg	2	1.000	1.000
AC-PO7d	Ofloxacin	4	0.800	1.000
AC-PO7d	Saline	2	0.667	1.000
AC-PO7d	TS-30mg/kg	6	0.200	1.000
AC-PO7d	TS-15mg/kg	6	0.200	1.000
AC-PO7d	anti-tip-chimer	6	0.200	1.000
AC-PO7d	anti-rsPilA	4	0.333	1.000
AC-5mg/kg	AC-10mg/kg	2	1.000	1.000
AC-5mg/kg	Ofloxacin	4	0.800	1.000
AC-5mg/kg	Saline	2	0.667	1.000
AC-10mg/kg	Ofloxacin	4	0.800	1.000
AC-10mg/kg	Saline	2	0.667	1.000
Ofloxacin	Saline	2	1.000	1.000
TS-30mg/kg	AC-5mg/kg	0	0.200	1.000
TS-30mg/kg	AC-10mg/kg	1	0.400	1.000
TS-30mg/kg	Ofloxacin	2	0.400	1.000
TS-30mg/kg	Saline	1	1.000	1.000
TS-30mg/kg	TS-15mg/kg	4	1.000	1.000
TS-30mg/kg	anti-tip-chimer	4	1.000	1.000
TS-30mg/kg	anti-rsPilA	4	0.800	1.000
TS-15mg/kg	AC-5mg/kg	0	0.200	1.000
TS-15mg/kg	AC-10mg/kg	1	0.400	1.000
TS-15mg/kg	Ofloxacin	3	0.700	1.000
TS-15mg/kg	Saline	2	1.000	1.000
TS-15mg/kg	anti-tip-chimer	6	0.700	1.000
anti-tip-chimer	AC-5mg/kg	0	0.200	1.000
anti-tip-chimer	AC-10mg/kg	1	0.400	1.000
anti-tip-chimer	Ofloxacin	3	0.700	1.000
anti-tip-chimer	Saline	2	1.000	1.000
anti-rsPilA	AC-5mg/kg	0	0.333	1.000
anti-rsPilA	AC-10mg/kg	0	0.333	1.000
anti-rsPilA	Ofloxacin	0	0.200	1.000
anti-rsPilA	Saline	0	0.667	1.000
anti-rsPilA	TS-15mg/kg	2	0.800	1.000
anti-rsPilA	anti-tip-chimer	2	0.800	1.000

Kruskal-Wallis (Omnibus Test)

**H**	10.515
**p-value**	0.231

Supplementary File 2
Longitudinal Pairwise-Distances and Pairwise-Differences

Fecal Unweighted UniFrac Pairwise-Distances
Baseline - 2DPT

#SampleID	SubjectID	Distance	Group
2AF2	Chinch2	0.712	AC-PO7d
3AF2	Chinch3	0.673	AC-PO7d
10F1	Chinch10	0.240	TS-30mg/kg
16F1	Chinch16	0.390	TS-30mg/kg
12F1	Chinch12	0.348	TS-30mg/kg
11F1	Chinch11	0.377	anti-rsPilA
13F1	Chinch13	0.361	anti-rsPilA
17F1	Chinch17	0.204	anti-rsPilA
14F1	Chinch14	0.355	TS-15mg/kg
15F1	Chinch15	0.595	TS-15mg/kg
18F1	Chinch18	0.431	TS-15mg/kg
27F1	Chinch27	0.421	anti-tip-chimer
19F1	Chinch19	0.339	anti-tip-chimer
21F1	Chinch21	0.356	anti-tip-chimer
20F1	Chinch20	0.412	AC-5mg/kg
23F1	Chinch23	0.347	AC-5mg/kg
22F1	Chinch22	0.539	AC-10mg/kg
4F1	Chinch4	0.441	Ofloxacin
7F1	Chinch7	0.439	Ofloxacin
9F1	Chinch9	0.382	Saline
6F1	Chinch6	0.341	Saline
8F1	Chinch8	0.420	Saline

Supplementary File 2
Longitudinal Pairwise-Distances and Pairwise-Differences

Fecal Unweighted UniFrac Pairwise-Distances
Baseline - 5DPT

#SampleID	SubjectID	Distance	Group
2AF5	Chinch2	0.649	AC-PO7d
1AF5	Chinch1	0.408	AC-PO7d
3AF5	Chinch3	0.612	AC-PO7d
10F2	Chinch10	0.312	TS-30mg/kg
16F2	Chinch16	0.420	TS-30mg/kg
12F2	Chinch12	0.367	TS-30mg/kg
17F2	Chinch17	0.308	anti-rsPilA
11F2	Chinch11	0.335	anti-rsPilA
18F2	Chinch18	0.321	TS-15mg/kg
14F2	Chinch14	0.310	TS-15mg/kg
15F2	Chinch15	0.524	TS-15mg/kg
21F2	Chinch21	0.416	anti-tip-chimer
19F2	Chinch19	0.357	anti-tip-chimer
27F2	Chinch27	0.407	anti-tip-chimer
20F2	Chinch20	0.633	AC-5mg/kg
23F2	Chinch23	0.744	AC-5mg/kg
22F2	Chinch22	0.749	AC-10mg/kg
25F2	Chinch25	0.612	AC-10mg/kg
5F2	Chinch5	0.348	Ofloxacin
7F2	Chinch7	0.462	Ofloxacin
4F2	Chinch4	0.488	Ofloxacin
9F2	Chinch9	0.410	Saline
6F2	Chinch6	0.379	Saline
8F2	Chinch8	0.377	Saline

Supplementary File 2
Longitudinal Pairwise-Distances and Pairwise-Differences

Fecal Unweighted UniFrac Pairwise-Distances
Baseline - 7DPT

#SampleID	SubjectID	Distance	Group
3AF7	Chinch3	0.655	AC-PO7d
2AF7	Chinch2	0.582	AC-PO7d
1AF7	Chinch1	0.724	AC-PO7d
10F3	Chinch10	0.267	TS-30mg/kg
12F3	Chinch12	0.408	TS-30mg/kg
16F3	Chinch16	0.388	TS-30mg/kg
11F3	Chinch11	0.326	anti-rsPilA
17F3	Chinch17	0.245	anti-rsPilA
15F3	Chinch15	0.486	TS-15mg/kg
14F3	Chinch14	0.391	TS-15mg/kg
18F3	Chinch18	0.454	TS-15mg/kg
21F3	Chinch21	0.415	anti-tip-chimer
19F3	Chinch19	0.321	anti-tip-chimer
20F3	Chinch20	0.568	AC-5mg/kg
23F3	Chinch23	0.608	AC-5mg/kg
25F3	Chinch25	0.580	AC-10mg/kg
4F3	Chinch4	0.521	Ofloxacin
7F3	Chinch7	0.373	Ofloxacin
5F3	Chinch5	0.321	Ofloxacin
8F3	Chinch8	0.517	Saline
6F3	Chinch6	0.391	Saline

Supplementary File 2
Longitudinal Pairwise-Distances and Pairwise-Differences

Fecal Unweighted UniFrac Pairwise-Distances
Baseline - 9DPT

#SampleID	SubjectID	Distance	Group
2AF9	Chinch2	0.528	AC-PO7d
3AF9	Chinch3	0.552	AC-PO7d
10F4	Chinch10	0.287	TS-30mg/kg
16F4	Chinch16	0.340	TS-30mg/kg
12F4	Chinch12	0.402	TS-30mg/kg
17F4	Chinch17	0.378	anti-rsPilA
11F4	Chinch11	0.310	anti-rsPilA
18F4	Chinch18	0.292	TS-15mg/kg
15F4	Chinch15	0.437	TS-15mg/kg
14F4	Chinch14	0.362	TS-15mg/kg
21F4	Chinch21	0.365	anti-tip-chimer
19F4	Chinch19	0.303	anti-tip-chimer
27F4	Chinch27	0.392	anti-tip-chimer
20F4	Chinch20	0.550	AC-5mg/kg
23F4	Chinch23	0.545	AC-5mg/kg
22F4	Chinch22	0.475	AC-10mg/kg
25F4	Chinch25	0.563	AC-10mg/kg
4F4	Chinch4	0.569	Ofloxacin
5F4	Chinch5	0.373	Ofloxacin
7F4	Chinch7	0.356	Ofloxacin
8F4	Chinch8	0.348	Saline

Supplementary File 2
Longitudinal Pairwise-Distances and Pairwise-Differences

Fecal Unweighted UniFrac Pairwise-Distance Mann-Whitney Pairwise Comparisons
Baseline - 2DPT

Group A	Group B	Mann-Whitney U	P-value	FDR P-value
AC-PO7d	AC-5mg/kg	4	0.333333333	0.8
AC-PO7d	AC-10mg/kg	2	0.666666667	0.933333333
AC-PO7d	Ofloxacin	4	0.333333333	0.8
AC-PO7d	Saline	6	0.2	0.72
AC-PO7d	TS-30mg/kg	6	0.2	0.72
AC-PO7d	TS-15mg/kg	6	0.2	0.72
AC-PO7d	anti-tip-chimer	6	0.2	0.72
AC-PO7d	anti-rsPilA	6	0.2	0.72
AC-5mg/kg	AC-10mg/kg	0	0.666666667	0.933333333
AC-5mg/kg	Ofloxacin	0	0.333333333	0.8
AC-5mg/kg	Saline	3	1	1
AC-10mg/kg	Ofloxacin	2	0.666666667	0.933333333
AC-10mg/kg	Saline	3	0.5	0.818181818
Ofloxacin	Saline	6	0.2	0.72
TS-30mg/kg	AC-5mg/kg	2	0.8	0.96
TS-30mg/kg	AC-10mg/kg	0	0.5	0.818181818
TS-30mg/kg	Ofloxacin	0	0.2	0.72
TS-30mg/kg	Saline	3	0.7	0.933333333
TS-30mg/kg	TS-15mg/kg	1	0.2	0.72
TS-30mg/kg	anti-tip-chimer	3	0.7	0.933333333
TS-30mg/kg	anti-rsPilA	5	1	1
TS-15mg/kg	AC-5mg/kg	5	0.4	0.8
TS-15mg/kg	AC-10mg/kg	1	1	1
TS-15mg/kg	Ofloxacin	2	0.8	0.96
TS-15mg/kg	Saline	7	0.4	0.8
TS-15mg/kg	anti-tip-chimer	7	0.4	0.8
anti-tip-chimer	AC-5mg/kg	3	1	1
anti-tip-chimer	AC-10mg/kg	0	0.5	0.818181818
anti-tip-chimer	Ofloxacin	0	0.2	0.72
anti-tip-chimer	Saline	4	1	1
anti-rsPilA	AC-5mg/kg	2	0.8	0.96
anti-rsPilA	AC-10mg/kg	0	0.5	0.818181818
anti-rsPilA	Ofloxacin	0	0.2	0.72
anti-rsPilA	Saline	2	0.4	0.8
anti-rsPilA	TS-15mg/kg	2	0.4	0.8
anti-rsPilA	anti-tip-chimer	4	1	1

Kruskal-Wallis (Omnibus Test)

**H**	12.787
**p-value**	0.119

Supplementary File 2
Longitudinal Pairwise-Distances and Pairwise-Differences

Fecal Unweighted UniFrac Pairwise-Distance Mann-Whitney Pairwise Comparisons
Baseline - 5DPT

Group A	Group B	Mann-Whitney U	P-value	FDR P-value
AC-PO7d	AC-5mg/kg	1	0.4	0.6
AC-PO7d	AC-10mg/kg	2	0.8	0.9
AC-PO7d	Ofloxacin	7	0.4	0.6
AC-PO7d	Saline	8	0.2	0.4
AC-PO7d	TS-30mg/kg	8	0.2	0.4
AC-PO7d	TS-15mg/kg	8	0.2	0.4
AC-PO7d	anti-tip-chimer	8	0.2	0.4
AC-PO7d	anti-rsPilA	6	0.2	0.4
AC-5mg/kg	AC-10mg/kg	2	1	1
AC-5mg/kg	Ofloxacin	6	0.2	0.4
AC-5mg/kg	Saline	6	0.2	0.4
AC-10mg/kg	Ofloxacin	6	0.2	0.4
AC-10mg/kg	Saline	6	0.2	0.4
Ofloxacin	Saline	6	0.7	0.84
TS-30mg/kg	AC-5mg/kg	0	0.2	0.4
TS-30mg/kg	AC-10mg/kg	0	0.2	0.4
TS-30mg/kg	Ofloxacin	2	0.4	0.6
TS-30mg/kg	Saline	3	0.7	0.84
TS-30mg/kg	TS-15mg/kg	5	1	1
TS-30mg/kg	anti-tip-chimer	4	1	1
TS-30mg/kg	anti-rsPilA	5	0.4	0.6
TS-15mg/kg	AC-5mg/kg	0	0.2	0.4
TS-15mg/kg	AC-10mg/kg	0	0.2	0.4
TS-15mg/kg	Ofloxacin	3	0.7	0.84
TS-15mg/kg	Saline	3	0.7	0.84
TS-15mg/kg	anti-tip-chimer	3	0.7	0.84
anti-tip-chimer	AC-5mg/kg	0	0.2	0.4
anti-tip-chimer	AC-10mg/kg	0	0.2	0.4
anti-tip-chimer	Ofloxacin	3	0.7	0.84
anti-tip-chimer	Saline	5	1	1
anti-rsPilA	AC-5mg/kg	0	0.333333333	0.6
anti-rsPilA	AC-10mg/kg	0	0.333333333	0.6
anti-rsPilA	Ofloxacin	0	0.2	0.4
anti-rsPilA	Saline	0	0.2	0.4
anti-rsPilA	TS-15mg/kg	2	0.8	0.9
anti-rsPilA	anti-tip-chimer	0	0.2	0.4

Kruskal-Wallis (Omnibus Test)

**H**	14.790
**p-value**	0.063

Supplementary File 2
Longitudinal Pairwise-Distances and Pairwise-Differences

Fecal Unweighted UniFrac Pairwise-Distance Mann-Whitney Pairwise Comparisons
Baseline - 7DPT

Group A	Group B	Mann-Whitney U	P-value	FDR P-value
AC-PO7d	AC-5mg/kg	5	0.4	0.72
AC-PO7d	AC-10mg/kg	3	0.5	0.75
AC-PO7d	Ofloxacin	9	0.1	0.654545455
AC-PO7d	Saline	6	0.2	0.654545455
AC-PO7d	TS-30mg/kg	9	0.1	0.654545455
AC-PO7d	TS-15mg/kg	9	0.1	0.654545455
AC-PO7d	anti-tip-chimer	6	0.2	0.654545455
AC-PO7d	anti-rsPilA	6	0.2	0.654545455
AC-5mg/kg	AC-10mg/kg	1	1	1
AC-5mg/kg	Ofloxacin	6	0.2	0.654545455
AC-5mg/kg	Saline	4	0.333333333	0.72
AC-10mg/kg	Ofloxacin	3	0.5	0.75
AC-10mg/kg	Saline	2	0.666666667	0.827586207
Ofloxacin	Saline	2	0.8	0.872727273
TS-30mg/kg	AC-5mg/kg	0	0.2	0.654545455
TS-30mg/kg	AC-10mg/kg	0	0.5	0.75
TS-30mg/kg	Ofloxacin	4	1	1
TS-30mg/kg	Saline	1	0.4	0.72
TS-30mg/kg	TS-15mg/kg	1	0.2	0.654545455
TS-30mg/kg	anti-tip-chimer	2	0.8	0.872727273
TS-30mg/kg	anti-rsPilA	5	0.4	0.72
TS-15mg/kg	AC-5mg/kg	0	0.2	0.654545455
TS-15mg/kg	AC-10mg/kg	0	0.5	0.75
TS-15mg/kg	Ofloxacin	6	0.7	0.84
TS-15mg/kg	Saline	2	0.8	0.872727273
TS-15mg/kg	anti-tip-chimer	5	0.4	0.72
anti-tip-chimer	AC-5mg/kg	0	0.333333333	0.72
anti-tip-chimer	AC-10mg/kg	0	0.666666667	0.827586207
anti-tip-chimer	Ofloxacin	3	1	1
anti-tip-chimer	Saline	1	0.666666667	0.827586207
anti-rsPilA	AC-5mg/kg	0	0.333333333	0.72
anti-rsPilA	AC-10mg/kg	0	0.666666667	0.827586207
anti-rsPilA	Ofloxacin	1	0.4	0.72
anti-rsPilA	Saline	0	0.333333333	0.72
anti-rsPilA	TS-15mg/kg	0	0.2	0.654545455
anti-rsPilA	anti-tip-chimer	1	0.666666667	0.827586207

Kruskal-Wallis (Omnibus Test)

**H**	15.355
**p-value**	0.053

Supplementary File 2
Longitudinal Pairwise-Distances and Pairwise-Differences

Fecal Unweighted UniFrac Pairwise-Distance Mann-Whitney Pairwise Comparisons
Baseline - 9DPT

Group A	Group B	Mann-Whitney U	P-value	FDR P-value
AC-PO7d	AC-5mg/kg	2	1	1
AC-PO7d	AC-10mg/kg	2	1	1
AC-PO7d	Ofloxacin	4	0.8	1
AC-PO7d	Saline	2	0.666666667	1
AC-PO7d	TS-30mg/kg	6	0.2	0.8
AC-PO7d	TS-15mg/kg	6	0.2	0.8
AC-PO7d	anti-tip-chimer	6	0.2	0.8
AC-PO7d	anti-rsPilA	4	0.333333333	1
AC-5mg/kg	AC-10mg/kg	2	1	1
AC-5mg/kg	Ofloxacin	4	0.8	1
AC-5mg/kg	Saline	2	0.666666667	1
AC-10mg/kg	Ofloxacin	4	0.8	1
AC-10mg/kg	Saline	2	0.666666667	1
Ofloxacin	Saline	3	0.5	1
TS-30mg/kg	AC-5mg/kg	0	0.2	0.8
TS-30mg/kg	AC-10mg/kg	0	0.2	0.8
TS-30mg/kg	Ofloxacin	2	0.4	1
TS-30mg/kg	Saline	1	1	1
TS-30mg/kg	TS-15mg/kg	3	0.7	1
TS-30mg/kg	anti-tip-chimer	4	1	1
TS-30mg/kg	anti-rsPilA	3	1	1
TS-15mg/kg	AC-5mg/kg	0	0.2	0.8
TS-15mg/kg	AC-10mg/kg	0	0.2	0.8
TS-15mg/kg	Ofloxacin	3	0.7	1
TS-15mg/kg	Saline	2	1	1
TS-15mg/kg	anti-tip-chimer	4	1	1
anti-tip-chimer	AC-5mg/kg	0	0.2	0.8
anti-tip-chimer	AC-10mg/kg	0	0.2	0.8
anti-tip-chimer	Ofloxacin	3	0.7	1
anti-tip-chimer	Saline	2	1	1
anti-rsPilA	AC-5mg/kg	0	0.333333333	1
anti-rsPilA	AC-10mg/kg	0	0.333333333	1
anti-rsPilA	Ofloxacin	2	0.8	1
anti-rsPilA	Saline	1	1	1
anti-rsPilA	TS-15mg/kg	3	1	1
anti-rsPilA	anti-tip-chimer	3	1	1

Kruskal-Wallis (Omnibus Test)

**H**	11.186
**p-value**	0.191

Supplementary File 2
Longitudinal Pairwise-Distances and Pairwise-Differences

Fecal Evenness Pairwise-Differences

Baseline - 2DPT

#SampleID	SubjectID	Difference	Group
3AF2	Chinch3	-0.235	AC-PO7d
2AF2	Chinch2	-0.255	AC-PO7d
7F1	Chinch7	-0.056	Ofloxacin
4F1	Chinch4	0.049	Ofloxacin
6F1	Chinch6	0.021	Saline
8F1	Chinch8	-0.034	Saline
9F1	Chinch9	-0.024	Saline
12F1	Chinch12	-0.123	TS-30mg/kg
10F1	Chinch10	-0.071	TS-30mg/kg
16F1	Chinch16	0.029	TS-30mg/kg
11F1	Chinch11	-0.082	anti-rsPilA
17F1	Chinch17	-0.019	anti-rsPilA
13F1	Chinch13	-0.033	anti-rsPilA
14F1	Chinch14	0.029	TS-15mg/kg
18F1	Chinch18	-0.037	TS-15mg/kg
15F1	Chinch15	-0.005	TS-15mg/kg
19F1	Chinch19	-0.070	anti-tip-chimer
27F1	Chinch27	-0.006	anti-tip-chimer
21F1	Chinch21	0.061	anti-tip-chimer
20F1	Chinch20	0.022	AC-5mg/kg
23F1	Chinch23	0.029	AC-5mg/kg
22F1	Chinch22	-0.080	AC-10mg/kg

Supplementary File 2
Longitudinal Pairwise-Distances and Pairwise-Differences

Fecal Evenness Pairwise-Differences

Baseline - 5DPT

#SampleID	SubjectID	Difference	Group
2AF5	Chinch2	-0.147	AC-PO7d
1AF5	Chinch1	0.081	AC-PO7d
3AF5	Chinch3	0.031	AC-PO7d
7F2	Chinch7	0.017	Ofloxacin
4F2	Chinch4	0.040	Ofloxacin
5F2	Chinch5	-0.035	Ofloxacin
8F2	Chinch8	-0.035	Saline
6F2	Chinch6	-0.008	Saline
9F2	Chinch9	0.025	Saline
10F2	Chinch10	-0.042	TS-30mg/kg
12F2	Chinch12	-0.190	TS-30mg/kg
16F2	Chinch16	0.022	TS-30mg/kg
17F2	Chinch17	-0.039	anti-rsPilA
11F2	Chinch11	-0.026	anti-rsPilA
14F2	Chinch14	-0.032	TS-15mg/kg
18F2	Chinch18	-0.056	TS-15mg/kg
15F2	Chinch15	0.033	TS-15mg/kg
19F2	Chinch19	-0.101	anti-tip-chimer
27F2	Chinch27	-0.031	anti-tip-chimer
21F2	Chinch21	0.075	anti-tip-chimer
20F2	Chinch20	-0.090	AC-5mg/kg
23F2	Chinch23	-0.036	AC-5mg/kg
25F2	Chinch25	-0.204	AC-10mg/kg
22F2	Chinch22	-0.093	AC-10mg/kg

Supplementary File 2
Longitudinal Pairwise-Distances and Pairwise-Differences

Fecal Evenness Pairwise-Differences
Baseline - 7DPT

#SampleID	SubjectID	Difference	Group
2AF7	Chinch2	-0.201	AC-PO7d
1AF7	Chinch1	-0.096	AC-PO7d
3AF7	Chinch3	-0.252	AC-PO7d
5F3	Chinch5	-0.037	Ofloxacin
4F3	Chinch4	0.036	Ofloxacin
7F3	Chinch7	-0.005	Ofloxacin
8F3	Chinch8	-0.030	Saline
6F3	Chinch6	-0.002	Saline
16F3	Chinch16	0.015	TS-30mg/kg
12F3	Chinch12	-0.136	TS-30mg/kg
10F3	Chinch10	-0.049	TS-30mg/kg
17F3	Chinch17	0.050	anti-rsPilA
11F3	Chinch11	-0.027	anti-rsPilA
14F3	Chinch14	-0.042	TS-15mg/kg
18F3	Chinch18	-0.084	TS-15mg/kg
15F3	Chinch15	-0.015	TS-15mg/kg
21F3	Chinch21	0.020	anti-tip-chimer
19F3	Chinch19	-0.036	anti-tip-chimer
20F3	Chinch20	-0.024	AC-5mg/kg
23F3	Chinch23	0.055	AC-5mg/kg
25F3	Chinch25	-0.087	AC-10mg/kg

Supplementary File 2
Longitudinal Pairwise-Distances and Pairwise-Differences

Fecal Evenness Pairwise-Differences
Baseline - 9DPT

#SampleID	SubjectID	Difference	Group
2AF9	Chinch2	-0.010	AC-PO7d
3AF9	Chinch3	-0.182	AC-PO7d
5F4	Chinch5	-0.026	Ofloxacin
4F4	Chinch4	0.071	Ofloxacin
7F4	Chinch7	-0.043	Ofloxacin
8F4	Chinch8	-0.049	Saline
16F4	Chinch16	-0.133	TS-30mg/kg
12F4	Chinch12	-0.072	TS-30mg/kg
10F4	Chinch10	-0.012	TS-30mg/kg
17F4	Chinch17	-0.032	anti-rsPilA
11F4	Chinch11	-0.062	anti-rsPilA
14F4	Chinch14	-0.020	TS-15mg/kg
15F4	Chinch15	0.031	TS-15mg/kg
18F4	Chinch18	-0.043	TS-15mg/kg
19F4	Chinch19	-0.003	anti-tip-chimer
21F4	Chinch21	0.087	anti-tip-chimer
27F4	Chinch27	0.032	anti-tip-chimer
20F4	Chinch20	-0.029	AC-5mg/kg
23F4	Chinch23	0.047	AC-5mg/kg
25F4	Chinch25	-0.096	AC-10mg/kg
22F4	Chinch22	-0.032	AC-10mg/kg

Fecal Evenness Pairwise-Difference Mann-Whitney Pairwise Comparisons
Baseline - 2DPT

Group A	Group B	Mann-Whitney U	P-value	FDR P-value
AC-PO7d	AC-5mg/kg	0	0.333	0.900
AC-PO7d	AC-10mg/kg	0	0.667	1.000
AC-PO7d	Ofloxacin	0	0.333	0.900
AC-PO7d	Saline	0	0.200	0.900
AC-PO7d	TS-30mg/kg	0	0.200	0.900
AC-PO7d	TS-15mg/kg	0	0.200	0.900
AC-PO7d	anti-tip-chimer	0	0.200	0.900
AC-PO7d	anti-rsPilA	0	0.200	0.900
AC-5mg/kg	AC-10mg/kg	2	0.667	1.000
Ofloxacin	AC-5mg/kg	2	1.000	1.000
Ofloxacin	AC-10mg/kg	2	0.667	1.000
Ofloxacin	Saline	3	1.000	1.000
Ofloxacin	TS-30mg/kg	5	0.400	0.900
Ofloxacin	TS-15mg/kg	3	1.000	1.000
Ofloxacin	anti-tip-chimer	3	1.000	1.000
Ofloxacin	anti-rsPilA	4	0.800	1.000
Saline	AC-5mg/kg	0	0.200	0.900
Saline	AC-10mg/kg	3	0.500	0.947
Saline	TS-30mg/kg	6	0.700	1.000
Saline	TS-15mg/kg	4	1.000	1.000
Saline	anti-tip-chimer	4	1.000	1.000
Saline	anti-rsPilA	6	0.700	1.000
TS-30mg/kg	AC-5mg/kg	1	0.400	0.900
TS-30mg/kg	AC-10mg/kg	2	1.000	1.000
TS-30mg/kg	TS-15mg/kg	2	0.400	0.900
TS-30mg/kg	anti-tip-chimer	2	0.400	0.900
TS-30mg/kg	anti-rsPilA	4	1.000	1.000
TS-15mg/kg	AC-5mg/kg	1	0.400	0.900
TS-15mg/kg	AC-10mg/kg	3	0.500	0.947
TS-15mg/kg	anti-tip-chimer	5	1.000	1.000
anti-tip-chimer	AC-5mg/kg	2	0.800	1.000
anti-tip-chimer	AC-10mg/kg	3	0.500	0.947
anti-rsPilA	AC-5mg/kg	0	0.200	0.900
anti-rsPilA	AC-10mg/kg	2	1.000	1.000
anti-rsPilA	TS-15mg/kg	2	0.400	0.900
anti-rsPilA	anti-tip-chimer	2	0.400	0.900

Kruskal-Wallis (Omnibus Test)

**H**	10.514
**p-value**	0.231

Supplementary File 2
Longitudinal Pairwise-Distances and Pairwise-Differences

Fecal Evenness Pairwise-Difference Mann-Whitney Pairwise Comparisons
Baseline - 5DPT

Group A	Group B	Mann-Whitney U	P-value	FDR P-value
AC-PO7d	AC-5mg/kg	4	0.800	1.000
AC-PO7d	AC-10mg/kg	5	0.400	0.900
AC-PO7d	Ofloxacin	5	1.000	1.000
AC-PO7d	Saline	6	0.700	1.000
AC-PO7d	TS-30mg/kg	7	0.400	0.900
AC-PO7d	TS-15mg/kg	5	1.000	1.000
AC-PO7d	anti-tip-chimer	5	1.000	1.000
AC-PO7d	anti-rsPilA	4	0.800	1.000
AC-5mg/kg	AC-10mg/kg	4	0.333	0.900
Ofloxacin	AC-5mg/kg	6	0.200	0.900
Ofloxacin	AC-10mg/kg	6	0.200	0.900
Ofloxacin	Saline	6	0.700	1.000
Ofloxacin	TS-30mg/kg	7	0.400	0.900
Ofloxacin	TS-15mg/kg	6	0.700	1.000
Ofloxacin	anti-tip-chimer	5	1.000	1.000
Ofloxacin	anti-rsPilA	5	0.400	0.900
Saline	AC-5mg/kg	6	0.200	0.900
Saline	AC-10mg/kg	6	0.200	0.900
Saline	TS-30mg/kg	7	0.400	0.900
Saline	TS-15mg/kg	5	1.000	1.000
Saline	anti-tip-chimer	5	1.000	1.000
Saline	anti-rsPilA	5	0.400	0.900
TS-30mg/kg	AC-5mg/kg	3	1.000	1.000
TS-30mg/kg	AC-10mg/kg	5	0.400	0.900
TS-30mg/kg	TS-15mg/kg	3	0.700	1.000
TS-30mg/kg	anti-tip-chimer	3	0.700	1.000
TS-30mg/kg	anti-rsPilA	2	0.800	1.000
TS-15mg/kg	AC-5mg/kg	5	0.400	0.900
TS-15mg/kg	AC-10mg/kg	6	0.200	0.900
TS-15mg/kg	anti-tip-chimer	4	1.000	1.000
anti-tip-chimer	AC-5mg/kg	4	0.800	1.000
anti-tip-chimer	AC-10mg/kg	5	0.400	0.900
anti-rsPilA	AC-5mg/kg	3	0.667	1.000
anti-rsPilA	AC-10mg/kg	4	0.333	0.900
anti-rsPilA	TS-15mg/kg	3	1.000	1.000
anti-rsPilA	anti-tip-chimer	3	1.000	1.000

Kruskal-Wallis (Omnibus Test)

**H**	7.493
**p-value**	0.484

Supplementary File 2
Longitudinal Pairwise-Distances and Pairwise-Differences

Fecal Evenness Pairwise-Difference Mann-Whitney Pairwise Comparisons
Baseline - 7DPT

Group A	Group B	Mann-Whitney U	P-value	FDR P-value
AC-PO7d	AC-5mg/kg	0	0.200	0.857
AC-PO7d	AC-10mg/kg	0	0.500	0.857
AC-PO7d	Ofloxacin	0	0.100	0.857
AC-PO7d	Saline	0	0.200	0.857
AC-PO7d	TS-30mg/kg	1	0.200	0.857
AC-PO7d	TS-15mg/kg	0	0.100	0.857
AC-PO7d	anti-tip-chimer	0	0.200	0.857
AC-PO7d	anti-rsPilA	0	0.200	0.857
AC-5mg/kg	AC-10mg/kg	2	0.667	0.857
Ofloxacin	AC-5mg/kg	2	0.800	0.929
Ofloxacin	AC-10mg/kg	3	0.500	0.857
Ofloxacin	Saline	3	1.000	1.000
Ofloxacin	TS-30mg/kg	7	0.400	0.857
Ofloxacin	TS-15mg/kg	8	0.200	0.857
Ofloxacin	anti-tip-chimer	3	1.000	1.000
Ofloxacin	anti-rsPilA	2	0.800	0.929
Saline	AC-5mg/kg	1	0.667	0.857
Saline	AC-10mg/kg	2	0.667	0.857
Saline	TS-30mg/kg	4	0.800	0.929
Saline	TS-15mg/kg	5	0.400	0.857
Saline	anti-tip-chimer	2	1.000	1.000
Saline	anti-rsPilA	1	0.667	0.857
TS-30mg/kg	AC-5mg/kg	1	0.400	0.857
TS-30mg/kg	AC-10mg/kg	2	1.000	1.000
TS-30mg/kg	TS-15mg/kg	4	1.000	1.000
TS-30mg/kg	anti-tip-chimer	1	0.400	0.857
TS-30mg/kg	anti-rsPilA	1	0.400	0.857
TS-15mg/kg	AC-5mg/kg	1	0.400	0.857
TS-15mg/kg	AC-10mg/kg	3	0.500	0.857
TS-15mg/kg	anti-tip-chimer	1	0.400	0.857
anti-tip-chimer	AC-5mg/kg	1	0.667	0.857
anti-tip-chimer	AC-10mg/kg	2	0.667	0.857
anti-rsPilA	AC-5mg/kg	1	0.667	0.857
anti-rsPilA	AC-10mg/kg	2	0.667	0.857
anti-rsPilA	TS-15mg/kg	5	0.400	0.857
anti-rsPilA	anti-tip-chimer	3	0.667	0.857

Kruskal-Wallis (Omnibus Test)

**H**	12.143
**p-value**	0.145

Fecal Evenness Pairwise-Difference Mann-Whitney Pairwise Comparisons
Baseline - 9DPT

Group A	Group B	Mann-Whitney U	P-value	FDR P-value
AC-PO7d	AC-5mg/kg	1	0.667	1.000
AC-PO7d	AC-10mg/kg	2	1.000	1.000
AC-PO7d	Ofloxacin	2	0.800	1.000
AC-PO7d	Saline	1	1.000	1.000
AC-PO7d	TS-30mg/kg	3	1.000	1.000
AC-PO7d	TS-15mg/kg	2	0.800	1.000
AC-PO7d	anti-tip-chimer	0	0.200	0.960
AC-PO7d	anti-rsPilA	2	1.000	1.000
AC-5mg/kg	AC-10mg/kg	4	0.333	0.960
Ofloxacin	AC-5mg/kg	3	1.000	1.000
Ofloxacin	AC-10mg/kg	5	0.400	0.960
Ofloxacin	Saline	3	0.500	1.000
Ofloxacin	TS-30mg/kg	7	0.400	0.960
Ofloxacin	TS-15mg/kg	4	1.000	1.000
Ofloxacin	anti-tip-chimer	2	0.400	0.960
Ofloxacin	anti-rsPilA	5	0.400	0.960
Saline	AC-5mg/kg	0	0.667	1.000
Saline	AC-10mg/kg	1	1.000	1.000
Saline	TS-30mg/kg	2	1.000	1.000
Saline	TS-15mg/kg	0	0.500	1.000
Saline	anti-tip-chimer	0	0.500	1.000
Saline	anti-rsPilA	1	1.000	1.000
TS-30mg/kg	AC-5mg/kg	1	0.400	0.960
TS-30mg/kg	AC-10mg/kg	3	1.000	1.000
TS-30mg/kg	TS-15mg/kg	2	0.400	0.960
TS-30mg/kg	anti-tip-chimer	0	0.100	0.960
TS-30mg/kg	anti-rsPilA	2	0.800	1.000
TS-15mg/kg	AC-5mg/kg	2	0.800	1.000
TS-15mg/kg	AC-10mg/kg	5	0.400	0.960
TS-15mg/kg	anti-tip-chimer	1	0.200	0.960
anti-tip-chimer	AC-5mg/kg	4	0.800	1.000
anti-tip-chimer	AC-10mg/kg	6	0.200	0.960
anti-rsPilA	AC-5mg/kg	0	0.333	0.960
anti-rsPilA	AC-10mg/kg	2	1.000	1.000
anti-rsPilA	TS-15mg/kg	1	0.400	0.960
anti-rsPilA	anti-tip-chimer	0	0.200	0.960

Kruskal-Wallis (Omnibus Test)

**H**	10.013
**p-value**	0.264

Supplementary File 2
Longitudinal Pairwise-Distances and Pairwise-Differences

Fecal FaithPD Pairwise-Differences

Baseline - 2DPT

#SampleID	SubjectID	Difference	Group
2AF2	Chinch2	-15.642	AC-PO7d
3AF2	Chinch3	-15.383	AC-PO7d
4F1	Chinch4	-0.508	Ofloxacin
7F1	Chinch7	-2.365	Ofloxacin
6F1	Chinch6	0.248	Saline
9F1	Chinch9	-3.457	Saline
8F1	Chinch8	-2.254	Saline
10F1	Chinch10	-1.703	TS-30mg/kg
12F1	Chinch12	0.596	TS-30mg/kg
16F1	Chinch16	-3.783	TS-30mg/kg
13F1	Chinch13	-2.544	anti-rsPilA
11F1	Chinch11	-3.761	anti-rsPilA
17F1	Chinch17	-0.765	anti-rsPilA
14F1	Chinch14	-2.279	TS-15mg/kg
18F1	Chinch18	-3.841	TS-15mg/kg
15F1	Chinch15	-10.049	TS-15mg/kg
27F1	Chinch27	-1.832	anti-tip-chimer
19F1	Chinch19	-3.669	anti-tip-chimer
21F1	Chinch21	1.298	anti-tip-chimer
23F1	Chinch23	-4.441	AC-5mg/kg
20F1	Chinch20	-5.305	AC-5mg/kg
22F1	Chinch22	-6.095	AC-10mg/kg

Supplementary File 2
Longitudinal Pairwise-Distances and Pairwise-Differences

Fecal FaithPD Pairwise-Differences

Baseline - 5DPT

#SampleID	SubjectID	Difference	Group
3AF5	Chinch3	-13.412	AC-PO7d
2AF5	Chinch2	-13.758	AC-PO7d
1AF5	Chinch1	-4.201	AC-PO7d
5F2	Chinch5	1.031	Ofloxacin
7F2	Chinch7	-1.496	Ofloxacin
4F2	Chinch4	-1.012	Ofloxacin
6F2	Chinch6	2.727	Saline
9F2	Chinch9	-1.212	Saline
8F2	Chinch8	-1.732	Saline
10F2	Chinch10	-2.008	TS-30mg/kg
12F2	Chinch12	1.377	TS-30mg/kg
16F2	Chinch16	-3.309	TS-30mg/kg
17F2	Chinch17	-2.540	anti-rsPilA
11F2	Chinch11	-1.301	anti-rsPilA
18F2	Chinch18	-1.383	TS-15mg/kg
15F2	Chinch15	-8.846	TS-15mg/kg
14F2	Chinch14	-2.040	TS-15mg/kg
21F2	Chinch21	0.596	anti-tip-chimer
27F2	Chinch27	-0.938	anti-tip-chimer
19F2	Chinch19	-5.007	anti-tip-chimer
20F2	Chinch20	-8.436	AC-5mg/kg
23F2	Chinch23	-8.913	AC-5mg/kg
22F2	Chinch22	-7.680	AC-10mg/kg
25F2	Chinch25	-4.313	AC-10mg/kg

Supplementary File 2
Longitudinal Pairwise-Distances and Pairwise-Differences

Fecal FaithPD Pairwise-Differences

Baseline - 7DPT

#SampleID	SubjectID	Difference	Group
3AF7	Chinch3	-14.757	AC-PO7d
1AF7	Chinch1	-14.665	AC-PO7d
2AF7	Chinch2	-12.086	AC-PO7d
4F3	Chinch4	-2.546	Ofloxacin
7F3	Chinch7	-1.491	Ofloxacin
5F3	Chinch5	1.451	Ofloxacin
8F3	Chinch8	-2.450	Saline
6F3	Chinch6	-0.280	Saline
12F3	Chinch12	-0.042	TS-30mg/kg
16F3	Chinch16	-1.897	TS-30mg/kg
10F3	Chinch10	0.158	TS-30mg/kg
11F3	Chinch11	-1.360	anti-rsPilA
17F3	Chinch17	-0.802	anti-rsPilA
15F3	Chinch15	-7.025	TS-15mg/kg
14F3	Chinch14	-3.335	TS-15mg/kg
18F3	Chinch18	-6.510	TS-15mg/kg
19F3	Chinch19	-4.288	anti-tip-chimer
21F3	Chinch21	2.871	anti-tip-chimer
23F3	Chinch23	-5.938	AC-5mg/kg
20F3	Chinch20	-4.789	AC-5mg/kg
25F3	Chinch25	-1.735	AC-10mg/kg

Supplementary File 2
Longitudinal Pairwise-Distances and Pairwise-Differences

Fecal FaithPD Pairwise-Differences

Baseline - 9DPT

#SampleID	SubjectID	Difference	Group
3AF9	Chinch3	-10.534	AC-PO7d
2AF9	Chinch2	-11.000	AC-PO7d
5F4	Chinch5	-0.865	Ofloxacin
4F4	Chinch4	-2.343	Ofloxacin
7F4	Chinch7	-1.095	Ofloxacin
8F4	Chinch8	-1.572	Saline
16F4	Chinch16	0.225	TS-30mg/kg
10F4	Chinch10	-1.772	TS-30mg/kg
12F4	Chinch12	0.580	TS-30mg/kg
11F4	Chinch11	-0.726	anti-rsPilA
17F4	Chinch17	-1.868	anti-rsPilA
15F4	Chinch15	-6.342	TS-15mg/kg
14F4	Chinch14	-1.430	TS-15mg/kg
18F4	Chinch18	-0.667	TS-15mg/kg
21F4	Chinch21	1.772	anti-tip-chimer
27F4	Chinch27	-0.394	anti-tip-chimer
19F4	Chinch19	-2.799	anti-tip-chimer
20F4	Chinch20	-5.596	AC-5mg/kg
23F4	Chinch23	-1.692	AC-5mg/kg
22F4	Chinch22	-2.677	AC-10mg/kg
25F4	Chinch25	-3.080	AC-10mg/kg

Supplementary File 2
Longitudinal Pairwise-Distances and Pairwise-Differences

Fecal FaithPD Pairwise-Difference Mann-Whitney Pairwise Comparisons
Baseline - 2DPT

Group A	Group B	Mann-Whitney U	P-value	FDR P-value
AC-PO7d	AC-5mg/kg	0	0.333	0.800
AC-PO7d	AC-10mg/kg	0	0.667	0.933
AC-PO7d	Ofloxacin	0	0.333	0.800
AC-PO7d	Saline	0	0.200	0.600
AC-PO7d	TS-30mg/kg	0	0.200	0.600
AC-PO7d	TS-15mg/kg	0	0.200	0.600
AC-PO7d	anti-tip-chimer	0	0.200	0.600
AC-PO7d	anti-rsPilA	0	0.200	0.600
AC-5mg/kg	AC-10mg/kg	2	0.667	0.933
Ofloxacin	AC-5mg/kg	4	0.333	0.800
Ofloxacin	AC-10mg/kg	2	0.667	0.933
Ofloxacin	Saline	3	1.000	1.000
Ofloxacin	TS-30mg/kg	3	1.000	1.000
Ofloxacin	TS-15mg/kg	5	0.400	0.800
Ofloxacin	anti-tip-chimer	3	1.000	1.000
Ofloxacin	anti-rsPilA	5	0.400	0.800
Saline	AC-5mg/kg	6	0.200	0.600
Saline	AC-10mg/kg	3	0.500	0.818
Saline	TS-30mg/kg	4	1.000	1.000
Saline	TS-15mg/kg	8	0.200	0.600
Saline	anti-tip-chimer	4	1.000	1.000
Saline	anti-rsPilA	6	0.700	0.933
TS-30mg/kg	AC-5mg/kg	6	0.200	0.600
TS-30mg/kg	AC-10mg/kg	3	0.500	0.818
TS-30mg/kg	TS-15mg/kg	8	0.200	0.600
TS-30mg/kg	anti-tip-chimer	4	1.000	1.000
TS-30mg/kg	anti-rsPilA	5	1.000	1.000
TS-15mg/kg	AC-5mg/kg	4	0.800	1.000
TS-15mg/kg	AC-10mg/kg	2	1.000	1.000
TS-15mg/kg	anti-tip-chimer	1	0.200	0.600
anti-tip-chimer	AC-5mg/kg	6	0.200	0.600
anti-tip-chimer	AC-10mg/kg	3	0.500	0.818
anti-rsPilA	AC-5mg/kg	6	0.200	0.600
anti-rsPilA	AC-10mg/kg	3	0.500	0.818
anti-rsPilA	TS-15mg/kg	7	0.400	0.800
anti-rsPilA	anti-tip-chimer	3	0.700	0.933

Kruskal-Wallis (Omnibus Test)

**H**	13.308
**p-value**	0.102

Fecal FaithPD Pairwise-Difference Mann-Whitney Pairwise Comparisons
Baseline - 5DPT

Group A	Group B	Mann-Whitney U	P-value	FDR P-value
AC-PO7d	AC-5mg/kg	2	0.800	0.929
AC-PO7d	AC-10mg/kg	2	0.800	0.929
AC-PO7d	Ofloxacin	0	0.100	0.480
AC-PO7d	Saline	0	0.100	0.480
AC-PO7d	TS-30mg/kg	0	0.100	0.480
AC-PO7d	TS-15mg/kg	1	0.200	0.480
AC-PO7d	anti-tip-chimer	1	0.200	0.480
AC-PO7d	anti-rsPilA	0	0.200	0.480
AC-5mg/kg	AC-10mg/kg	0	0.333	0.600
Ofloxacin	AC-5mg/kg	6	0.200	0.480
Ofloxacin	AC-10mg/kg	6	0.200	0.480
Ofloxacin	Saline	5	1.000	1.000
Ofloxacin	TS-30mg/kg	6	0.700	0.929
Ofloxacin	TS-15mg/kg	8	0.200	0.480
Ofloxacin	anti-tip-chimer	5	1.000	1.000
Ofloxacin	anti-rsPilA	5	0.400	0.600
Saline	AC-5mg/kg	6	0.200	0.480
Saline	AC-10mg/kg	6	0.200	0.480
Saline	TS-30mg/kg	7	0.400	0.600
Saline	TS-15mg/kg	8	0.200	0.480
Saline	anti-tip-chimer	5	1.000	1.000
Saline	anti-rsPilA	5	0.400	0.600
TS-30mg/kg	AC-5mg/kg	6	0.200	0.480
TS-30mg/kg	AC-10mg/kg	6	0.200	0.480
TS-30mg/kg	TS-15mg/kg	6	0.700	0.929
TS-30mg/kg	anti-tip-chimer	5	1.000	1.000
TS-30mg/kg	anti-rsPilA	3	1.000	1.000
TS-15mg/kg	AC-5mg/kg	5	0.400	0.600
TS-15mg/kg	AC-10mg/kg	4	0.800	0.929
TS-15mg/kg	anti-tip-chimer	2	0.400	0.600
anti-tip-chimer	AC-5mg/kg	6	0.200	0.480
anti-tip-chimer	AC-10mg/kg	5	0.400	0.600
anti-rsPilA	AC-5mg/kg	4	0.333	0.600
anti-rsPilA	AC-10mg/kg	4	0.333	0.600
anti-rsPilA	TS-15mg/kg	4	0.800	0.929
anti-rsPilA	anti-tip-chimer	2	0.800	0.929

Kruskal-Wallis (Omnibus Test)

**H**	14.507
**p-value**	0.069

Supplementary File 2
Longitudinal Pairwise-Distances and Pairwise-Differences

Fecal FaithPD Pairwise-Difference Mann-Whitney Pairwise Comparisons
Baseline - 7DPT

Group A	Group B	Mann-Whitney U	P-value	FDR P-value
AC-PO7d	AC-5mg/kg	0	0.200	0.554
AC-PO7d	AC-10mg/kg	0	0.500	0.900
AC-PO7d	Ofloxacin	0	0.100	0.554
AC-PO7d	Saline	0	0.200	0.554
AC-PO7d	TS-30mg/kg	0	0.100	0.554
AC-PO7d	TS-15mg/kg	0	0.100	0.554
AC-PO7d	anti-tip-chimer	0	0.200	0.554
AC-PO7d	anti-rsPilA	0	0.200	0.554
AC-5mg/kg	AC-10mg/kg	0	0.667	1.000
Ofloxacin	AC-5mg/kg	6	0.200	0.554
Ofloxacin	AC-10mg/kg	2	1.000	1.000
Ofloxacin	Saline	3	1.000	1.000
Ofloxacin	TS-30mg/kg	4	1.000	1.000
Ofloxacin	TS-15mg/kg	9	0.100	0.554
Ofloxacin	anti-tip-chimer	3	1.000	1.000
Ofloxacin	anti-rsPilA	2	0.800	1.000
Saline	AC-5mg/kg	4	0.333	0.750
Saline	AC-10mg/kg	1	1.000	1.000
Saline	TS-30mg/kg	1	0.400	0.800
Saline	TS-15mg/kg	6	0.200	0.554
Saline	anti-tip-chimer	2	1.000	1.000
Saline	anti-rsPilA	2	1.000	1.000
TS-30mg/kg	AC-5mg/kg	6	0.200	0.554
TS-30mg/kg	AC-10mg/kg	2	1.000	1.000
TS-30mg/kg	TS-15mg/kg	9	0.100	0.554
TS-30mg/kg	anti-tip-chimer	3	1.000	1.000
TS-30mg/kg	anti-rsPilA	4	0.800	1.000
TS-15mg/kg	AC-5mg/kg	2	0.800	1.000
TS-15mg/kg	AC-10mg/kg	0	0.500	0.900
TS-15mg/kg	anti-tip-chimer	1	0.400	0.800
anti-tip-chimer	AC-5mg/kg	4	0.333	0.750
anti-tip-chimer	AC-10mg/kg	1	1.000	1.000
anti-rsPilA	AC-5mg/kg	4	0.333	0.750
anti-rsPilA	AC-10mg/kg	2	0.667	1.000
anti-rsPilA	TS-15mg/kg	6	0.200	0.554
anti-rsPilA	anti-tip-chimer	2	1.000	1.000

Kruskal-Wallis (Omnibus Test)

**H**	14.835
**p-value**	0.062

Supplementary File 2
Longitudinal Pairwise-Distances and Pairwise-Differences

Fecal FaithPD Pairwise-Difference Mann-Whitney Pairwise Comparisons
Baseline - 9DPT

Group A	Group B	Mann-Whitney U	P-value	FDR P-value
AC-PO7d	AC-5mg/kg	0	0.333	0.800
AC-PO7d	AC-10mg/kg	0	0.333	0.800
AC-PO7d	Ofloxacin	0	0.200	0.800
AC-PO7d	Saline	0	0.667	1.000
AC-PO7d	TS-30mg/kg	0	0.200	0.800
AC-PO7d	TS-15mg/kg	0	0.200	0.800
AC-PO7d	anti-tip-chimer	0	0.200	0.800
AC-PO7d	anti-rsPilA	0	0.333	0.800
AC-5mg/kg	AC-10mg/kg	2	1.000	1.000
Ofloxacin	AC-5mg/kg	5	0.400	0.800
Ofloxacin	AC-10mg/kg	6	0.200	0.800
Ofloxacin	Saline	2	1.000	1.000
Ofloxacin	TS-30mg/kg	2	0.400	0.800
Ofloxacin	TS-15mg/kg	5	1.000	1.000
Ofloxacin	anti-tip-chimer	3	0.700	1.000
Ofloxacin	anti-rsPilA	2	0.800	1.000
Saline	AC-5mg/kg	2	0.667	1.000
Saline	AC-10mg/kg	2	0.667	1.000
Saline	TS-30mg/kg	1	1.000	1.000
Saline	TS-15mg/kg	1	1.000	1.000
Saline	anti-tip-chimer	1	1.000	1.000
Saline	anti-rsPilA	1	1.000	1.000
TS-30mg/kg	AC-5mg/kg	5	0.400	0.800
TS-30mg/kg	AC-10mg/kg	6	0.200	0.800
TS-30mg/kg	TS-15mg/kg	7	0.400	0.800
TS-30mg/kg	anti-tip-chimer	5	1.000	1.000
TS-30mg/kg	anti-rsPilA	5	0.400	0.800
TS-15mg/kg	AC-5mg/kg	4	0.800	1.000
TS-15mg/kg	AC-10mg/kg	4	0.800	1.000
TS-15mg/kg	anti-tip-chimer	2	0.400	0.800
anti-tip-chimer	AC-5mg/kg	5	0.400	0.800
anti-tip-chimer	AC-10mg/kg	5	0.400	0.800
anti-rsPilA	AC-5mg/kg	3	0.667	1.000
anti-rsPilA	AC-10mg/kg	4	0.333	0.800
anti-rsPilA	TS-15mg/kg	3	1.000	1.000
anti-rsPilA	anti-tip-chimer	2	0.800	1.000

Kruskal-Wallis (Omnibus Test)

**H**	10.368
**p-value**	0.240

Supplementary File 2
Longitudinal Pairwise-Distances and Pairwise-Differences

Fecal Richness Pairwise-Differences
Baseline - 2DPT

#SampleID	SubjectID	Difference	Group
2AF2	Chinch2	-379.000	AC-PO7d
3AF2	Chinch3	-411.000	AC-PO7d
7F1	Chinch7	-30.000	Ofloxacin
4F1	Chinch4	-31.000	Ofloxacin
9F1	Chinch9	-72.000	Saline
6F1	Chinch6	9.000	Saline
8F1	Chinch8	-29.000	Saline
16F1	Chinch16	-49.000	TS-30mg/kg
10F1	Chinch10	-21.000	TS-30mg/kg
12F1	Chinch12	22.000	TS-30mg/kg
17F1	Chinch17	-25.000	anti-rsPilA
13F1	Chinch13	-35.000	anti-rsPilA
11F1	Chinch11	-59.000	anti-rsPilA
18F1	Chinch18	-110.000	TS-15mg/kg
14F1	Chinch14	-39.000	TS-15mg/kg
15F1	Chinch15	-213.000	TS-15mg/kg
19F1	Chinch19	-89.000	anti-tip-chimer
21F1	Chinch21	9.000	anti-tip-chimer
27F1	Chinch27	-17.000	anti-tip-chimer
23F1	Chinch23	-83.000	AC-5mg/kg
20F1	Chinch20	-82.000	AC-5mg/kg
22F1	Chinch22	-135.000	AC-10mg/kg

Supplementary File 2
Longitudinal Pairwise-Distances and Pairwise-Differences

Fecal Richness Pairwise-Differences

Baseline - 5DPT

#SampleID	SubjectID	Difference	Group
1AF5	Chinch1	-121.000	AC-PO7d
2AF5	Chinch2	-333.000	AC-PO7d
3AF5	Chinch3	-368.000	AC-PO7d
7F2	Chinch7	-27.000	Ofloxacin
4F2	Chinch4	-60.000	Ofloxacin
5F2	Chinch5	59.000	Ofloxacin
8F2	Chinch8	-17.000	Saline
9F2	Chinch9	-22.000	Saline
6F2	Chinch6	95.000	Saline
12F2	Chinch12	44.000	TS-30mg/kg
10F2	Chinch10	-12.000	TS-30mg/kg
16F2	Chinch16	-40.000	TS-30mg/kg
11F2	Chinch11	-39.000	anti-rsPilA
17F2	Chinch17	-96.000	anti-rsPilA
14F2	Chinch14	-47.000	TS-15mg/kg
15F2	Chinch15	-203.000	TS-15mg/kg
18F2	Chinch18	-73.000	TS-15mg/kg
19F2	Chinch19	-118.000	anti-tip-chimer
21F2	Chinch21	15.000	anti-tip-chimer
27F2	Chinch27	-7.000	anti-tip-chimer
20F2	Chinch20	-153.000	AC-5mg/kg
23F2	Chinch23	-172.000	AC-5mg/kg
25F2	Chinch25	-78.000	AC-10mg/kg
22F2	Chinch22	-162	AC-10mg/kg

Supplementary File 2
Longitudinal Pairwise-Distances and Pairwise-Differences

Fecal Richness Pairwise-Differences

Baseline - 7DPT

#SampleID	SubjectID	Difference	Group
1AF7	Chinch1	-354.000	AC-PO7d
3AF7	Chinch3	-385.000	AC-PO7d
2AF7	Chinch2	-303.000	AC-PO7d
5F3	Chinch5	49.000	Ofloxacin
7F3	Chinch7	-5.000	Ofloxacin
4F3	Chinch4	-80.000	Ofloxacin
8F3	Chinch8	-49.000	Saline
6F3	Chinch6	-3.000	Saline
10F3	Chinch10	31.000	TS-30mg/kg
12F3	Chinch12	8.000	TS-30mg/kg
16F3	Chinch16	-32.000	TS-30mg/kg
17F3	Chinch17	-7.000	anti-rsPilA
11F3	Chinch11	-45.000	anti-rsPilA
18F3	Chinch18	-142.000	TS-15mg/kg
14F3	Chinch14	-73.000	TS-15mg/kg
15F3	Chinch15	-195.000	TS-15mg/kg
19F3	Chinch19	-105.000	anti-tip-chimer
21F3	Chinch21	41.000	anti-tip-chimer
20F3	Chinch20	-90.000	AC-5mg/kg
23F3	Chinch23	-129.000	AC-5mg/kg
25F3	Chinch25	-13	AC-10mg/kg

Supplementary File 2
Longitudinal Pairwise-Distances and Pairwise-Differences

Fecal Richness Pairwise-Differences
Baseline - 9DPT

#SampleID	SubjectID	Difference	Group
3AF9	Chinch3	-315.000	AC-PO7d
2AF9	Chinch2	-273.000	AC-PO7d
7F4	Chinch7	-4.000	Ofloxacin
5F4	Chinch5	8.000	Ofloxacin
4F4	Chinch4	-82.000	Ofloxacin
8F4	Chinch8	-25.000	Saline
12F4	Chinch12	43.000	TS-30mg/kg
16F4	Chinch16	9.000	TS-30mg/kg
10F4	Chinch10	-15.000	TS-30mg/kg
17F4	Chinch17	-36.000	anti-rsPilA
11F4	Chinch11	-21.000	anti-rsPilA
14F4	Chinch14	-26.000	TS-15mg/kg
15F4	Chinch15	-194.000	TS-15mg/kg
18F4	Chinch18	-20.000	TS-15mg/kg
19F4	Chinch19	-91.000	anti-tip-chimer
21F4	Chinch21	28.000	anti-tip-chimer
27F4	Chinch27	4.000	anti-tip-chimer
20F4	Chinch20	-104.000	AC-5mg/kg
23F4	Chinch23	-49.000	AC-5mg/kg
22F4	Chinch22	-55.000	AC-10mg/kg
25F4	Chinch25	-34	AC-10mg/kg

Fecal Richness Pairwise-Difference Mann-Whitney Pairwise Comparisons
Baseline - 2DPT

Group A	Group B	Mann-Whitney U	P-value	FDR P-value
AC-PO7d	AC-5mg/kg	0	0.333	0.750
AC-PO7d	AC-10mg/kg	0	0.667	0.900
AC-PO7d	Ofloxacin	0	0.333	0.750
AC-PO7d	Saline	0	0.200	0.554
AC-PO7d	TS-30mg/kg	0	0.200	0.554
AC-PO7d	TS-15mg/kg	0	0.200	0.554
AC-PO7d	anti-tip-chimer	0	0.200	0.554
AC-PO7d	anti-rsPilA	0	0.200	0.554
AC-5mg/kg	AC-10mg/kg	2	0.667	0.900
Ofloxacin	AC-5mg/kg	4	0.333	0.750
Ofloxacin	AC-10mg/kg	2	0.667	0.900
Ofloxacin	Saline	2	0.800	0.900
Ofloxacin	TS-30mg/kg	2	0.800	0.900
Ofloxacin	TS-15mg/kg	6	0.200	0.554
Ofloxacin	anti-tip-chimer	2	0.800	0.900
Ofloxacin	anti-rsPilA	4	0.800	0.900
Saline	AC-5mg/kg	6	0.200	0.554
Saline	AC-10mg/kg	3	0.500	0.857
Saline	TS-30mg/kg	3	0.700	0.900
Saline	TS-15mg/kg	8	0.200	0.554
Saline	anti-tip-chimer	4.5	1.000	1.000
Saline	anti-rsPilA	5	1.000	1.000
TS-30mg/kg	AC-5mg/kg	6	0.200	0.554
TS-30mg/kg	AC-10mg/kg	3	0.500	0.857
TS-30mg/kg	TS-15mg/kg	8	0.200	0.554
TS-30mg/kg	anti-tip-chimer	5	1.000	1.000
TS-30mg/kg	anti-rsPilA	7	0.400	0.847
TS-15mg/kg	AC-5mg/kg	2	0.800	0.900
TS-15mg/kg	AC-10mg/kg	2	1.000	1.000
TS-15mg/kg	anti-tip-chimer	1	0.200	0.554
anti-tip-chimer	AC-5mg/kg	4	0.800	0.900
anti-tip-chimer	AC-10mg/kg	3	0.500	0.857
anti-rsPilA	AC-5mg/kg	6	0.200	0.554
anti-rsPilA	AC-10mg/kg	3	0.500	0.857
anti-rsPilA	TS-15mg/kg	8	0.200	0.554
anti-rsPilA	anti-tip-chimer	3	0.700	0.900

Kruskal-Wallis (Omnibus Test)

**H**	13.237
**p-value**	0.104

Supplementary File 2
Longitudinal Pairwise-Distances and Pairwise-Differences

Fecal Richness Pairwise-Difference Mann-Whitney Pairwise Comparisons
Baseline - 5DPT

Group A	Group B	Mann-Whitney U	P-value	FDR P-value
AC-PO7d	AC-5mg/kg	2	0.800	0.929
AC-PO7d	AC-10mg/kg	1	0.400	0.600
AC-PO7d	Ofloxacin	0	0.100	0.424
AC-PO7d	Saline	0	0.100	0.424
AC-PO7d	TS-30mg/kg	0	0.100	0.424
AC-PO7d	TS-15mg/kg	1	0.200	0.424
AC-PO7d	anti-tip-chimer	0	0.100	0.424
AC-PO7d	anti-rsPilA	0	0.200	0.424
AC-5mg/kg	AC-10mg/kg	1	0.667	0.923
Ofloxacin	AC-5mg/kg	6	0.200	0.424
Ofloxacin	AC-10mg/kg	6	0.200	0.424
Ofloxacin	Saline	2	0.400	0.600
Ofloxacin	TS-30mg/kg	4	1.000	1.000
Ofloxacin	TS-15mg/kg	8	0.200	0.424
Ofloxacin	anti-tip-chimer	5	1.000	1.000
Ofloxacin	anti-rsPilA	5	0.400	0.600
Saline	AC-5mg/kg	6	0.200	0.424
Saline	AC-10mg/kg	6	0.200	0.424
Saline	TS-30mg/kg	5	1.000	1.000
Saline	TS-15mg/kg	9	0.100	0.424
Saline	anti-tip-chimer	5	1.000	1.000
Saline	anti-rsPilA	6	0.200	0.424
TS-30mg/kg	AC-5mg/kg	6	0.200	0.424
TS-30mg/kg	AC-10mg/kg	6	0.200	0.424
TS-30mg/kg	TS-15mg/kg	9	0.100	0.424
TS-30mg/kg	anti-tip-chimer	5	1.000	1.000
TS-30mg/kg	anti-rsPilA	5	0.400	0.600
TS-15mg/kg	AC-5mg/kg	4	0.800	0.929
TS-15mg/kg	AC-10mg/kg	4	0.800	0.929
TS-15mg/kg	anti-tip-chimer	2	0.400	0.600
anti-tip-chimer	AC-5mg/kg	6	0.200	0.424
anti-tip-chimer	AC-10mg/kg	5	0.400	0.600
anti-rsPilA	AC-5mg/kg	4	0.333	0.600
anti-rsPilA	AC-10mg/kg	3	0.667	0.923
anti-rsPilA	TS-15mg/kg	4	0.800	0.929
anti-rsPilA	anti-tip-chimer	2	0.800	0.929

Kruskal-Wallis (Omnibus Test)

**H**	16.257
**p-value**	0.039

Supplementary File 2
Longitudinal Pairwise-Distances and Pairwise-Differences

Fecal Richness Pairwise-Difference Mann-Whitney Pairwise Comparisons
Baseline - 7DPT

Group A	Group B	Mann-Whitney U	P-value	FDR P-value
AC-PO7d	AC-5mg/kg	0	0.200	0.554
AC-PO7d	AC-10mg/kg	0	0.500	0.900
AC-PO7d	Ofloxacin	0	0.100	0.554
AC-PO7d	Saline	0	0.200	0.554
AC-PO7d	TS-30mg/kg	0	0.100	0.554
AC-PO7d	TS-15mg/kg	0	0.100	0.554
AC-PO7d	anti-tip-chimer	0	0.200	0.554
AC-PO7d	anti-rsPilA	0	0.200	0.554
AC-5mg/kg	AC-10mg/kg	0	0.667	1.000
Ofloxacin	AC-5mg/kg	6	0.200	0.554
Ofloxacin	AC-10mg/kg	2	1.000	1.000
Ofloxacin	Saline	3	1.000	1.000
Ofloxacin	TS-30mg/kg	4	1.000	1.000
Ofloxacin	TS-15mg/kg	8	0.200	0.554
Ofloxacin	anti-tip-chimer	4	0.800	1.000
Ofloxacin	anti-rsPilA	4	0.800	1.000
Saline	AC-5mg/kg	4	0.333	0.800
Saline	AC-10mg/kg	1	1.000	1.000
Saline	TS-30mg/kg	1	0.400	0.800
Saline	TS-15mg/kg	6	0.200	0.554
Saline	anti-tip-chimer	2	1.000	1.000
Saline	anti-rsPilA	2	1.000	1.000
TS-30mg/kg	AC-5mg/kg	6	0.200	0.554
TS-30mg/kg	AC-10mg/kg	2	1.000	1.000
TS-30mg/kg	TS-15mg/kg	9	0.100	0.554
TS-30mg/kg	anti-tip-chimer	3	1.000	1.000
TS-30mg/kg	anti-rsPilA	5	0.400	0.800
TS-15mg/kg	AC-5mg/kg	2	0.800	1.000
TS-15mg/kg	AC-10mg/kg	0	0.500	0.900
TS-15mg/kg	anti-tip-chimer	1	0.400	0.800
anti-tip-chimer	AC-5mg/kg	3	0.667	1.000
anti-tip-chimer	AC-10mg/kg	1	1.000	1.000
anti-rsPilA	AC-5mg/kg	4	0.333	0.800
anti-rsPilA	AC-10mg/kg	1	1.000	1.000
anti-rsPilA	TS-15mg/kg	6	0.200	0.554
anti-rsPilA	anti-tip-chimer	2	1.000	1.000

Kruskal-Wallis (Omnibus Test)

**H**	14.156
**p-value**	0.078

Fecal Richness Pairwise-Difference Mann-Whitney Pairwise Comparisons
Baseline - 9DPT

Group A	Group B	Mann-Whitney U	P-value	FDR P-value
AC-PO7d	AC-5mg/kg	0	0.333	0.847
AC-PO7d	AC-10mg/kg	0	0.333	0.847
AC-PO7d	Ofloxacin	0	0.200	0.847
AC-PO7d	Saline	0	0.667	0.960
AC-PO7d	TS-30mg/kg	0	0.200	0.847
AC-PO7d	TS-15mg/kg	0	0.200	0.847
AC-PO7d	anti-tip-chimer	0	0.200	0.847
AC-PO7d	anti-rsPilA	0	0.333	0.847
AC-5mg/kg	AC-10mg/kg	1	0.667	0.960
Ofloxacin	AC-5mg/kg	5	0.400	0.847
Ofloxacin	AC-10mg/kg	4	0.800	0.960
Ofloxacin	Saline	2	1.000	1.000
Ofloxacin	TS-30mg/kg	2	0.400	0.847
Ofloxacin	TS-15mg/kg	7	0.400	0.847
Ofloxacin	anti-tip-chimer	4	1.000	1.000
Ofloxacin	anti-rsPilA	4	0.800	0.960
Saline	AC-5mg/kg	2	0.667	0.960
Saline	AC-10mg/kg	2	0.667	0.960
Saline	TS-30mg/kg	0	0.500	0.960
Saline	TS-15mg/kg	2	1.000	1.000
Saline	anti-tip-chimer	1	1.000	1.000
Saline	anti-rsPilA	1	1.000	1.000
TS-30mg/kg	AC-5mg/kg	6	0.200	0.847
TS-30mg/kg	AC-10mg/kg	6	0.200	0.847
TS-30mg/kg	TS-15mg/kg	9	0.100	0.847
TS-30mg/kg	anti-tip-chimer	6	0.700	0.960
TS-30mg/kg	anti-rsPilA	6	0.200	0.847
TS-15mg/kg	AC-5mg/kg	4	0.800	0.960
TS-15mg/kg	AC-10mg/kg	4	0.800	0.960
TS-15mg/kg	anti-tip-chimer	2	0.400	0.847
anti-tip-chimer	AC-5mg/kg	5	0.400	0.847
anti-tip-chimer	AC-10mg/kg	4	0.800	0.960
anti-rsPilA	AC-5mg/kg	4	0.333	0.847
anti-rsPilA	AC-10mg/kg	3	0.667	0.960
anti-rsPilA	TS-15mg/kg	3	1.000	1.000
anti-rsPilA	anti-tip-chimer	2	0.800	0.960

Kruskal-Wallis (Omnibus Test)

**H**	11.870
**p-value**	0.157

Supplementary File 2
Longitudinal Pairwise-Distances and Pairwise-Differences

Fecal Shannon Diversity Pairwise-Differences
Baseline - 2DPT

#SampleID	SubjectID	Difference	Group
3AF2	Chinch3	-3.682	AC-PO7d
2AF2	Chinch2	-3.848	AC-PO7d
7F1	Chinch7	-0.652	Ofloxacin
4F1	Chinch4	0.170	Ofloxacin
9F1	Chinch9	-0.713	Saline
6F1	Chinch6	0.222	Saline
8F1	Chinch8	-0.481	Saline
10F1	Chinch10	-0.676	TS-30mg/kg
12F1	Chinch12	-0.795	TS-30mg/kg
16F1	Chinch16	-0.126	TS-30mg/kg
17F1	Chinch17	-0.289	anti-rsPilA
11F1	Chinch11	-0.967	anti-rsPilA
13F1	Chinch13	-0.500	anti-rsPilA
15F1	Chinch15	-1.296	TS-15mg/kg
18F1	Chinch18	-0.926	TS-15mg/kg
14F1	Chinch14	-0.052	TS-15mg/kg
27F1	Chinch27	-0.164	anti-tip-chimer
21F1	Chinch21	0.518	anti-tip-chimer
19F1	Chinch19	-1.016	anti-tip-chimer
20F1	Chinch20	-0.335	AC-5mg/kg
23F1	Chinch23	-0.251	AC-5mg/kg
22F1	Chinch22	-1.559	AC-10mg/kg

Supplementary File 2
Longitudinal Pairwise-Distances and Pairwise-Differences

Fecal Shannon Diversity Pairwise-Differences
Baseline - 5DPT

#SampleID	SubjectID	Difference	Group
1AF5	Chinch1	0.273	AC-PO7d
2AF5	Chinch2	-2.666	AC-PO7d
3AF5	Chinch3	-1.474	AC-PO7d
5F2	Chinch5	0.167	Ofloxacin
4F2	Chinch4	-0.126	Ofloxacin
7F2	Chinch7	-0.109	Ofloxacin
9F2	Chinch9	0.043	Saline
6F2	Chinch6	0.521	Saline
8F2	Chinch8	-0.386	Saline
10F2	Chinch10	-0.399	TS-30mg/kg
12F2	Chinch12	-1.199	TS-30mg/kg
16F2	Chinch16	-0.105	TS-30mg/kg
17F2	Chinch17	-0.930	anti-rsPilA
11F2	Chinch11	-0.419	anti-rsPilA
15F2	Chinch15	-0.930	TS-15mg/kg
14F2	Chinch14	-0.559	TS-15mg/kg
18F2	Chinch18	-0.819	TS-15mg/kg
27F2	Chinch27	-0.276	anti-tip-chimer
21F2	Chinch21	0.659	anti-tip-chimer
19F2	Chinch19	-1.440	anti-tip-chimer
23F2	Chinch23	-1.612	AC-5mg/kg
20F2	Chinch20	-1.788	AC-5mg/kg
25F2	Chinch25	-2.089	AC-10mg/kg
22F2	Chinch22	-2.000	AC-10mg/kg

Supplementary File 2
Longitudinal Pairwise-Distances and Pairwise-Differences

Fecal Shannon Diversity Pairwise-Differences
Baseline - 7DPT

#SampleID	SubjectID	Difference	Group
3AF7	Chinch3	-3.540	AC-PO7d
2AF7	Chinch2	-2.813	AC-PO7d
1AF7	Chinch1	-2.733	AC-PO7d
4F3	Chinch4	-0.342	Ofloxacin
5F3	Chinch5	0.088	Ofloxacin
7F3	Chinch7	-0.079	Ofloxacin
6F3	Chinch6	-0.036	Saline
8F3	Chinch8	-0.632	Saline
16F3	Chinch16	-0.096	TS-30mg/kg
10F3	Chinch10	-0.167	TS-30mg/kg
12F3	Chinch12	-0.970	TS-30mg/kg
11F3	Chinch11	-0.464	anti-rsPilA
17F3	Chinch17	0.357	anti-rsPilA
18F3	Chinch18	-1.530	TS-15mg/kg
15F3	Chinch15	-1.189	TS-15mg/kg
14F3	Chinch14	-0.843	TS-15mg/kg
19F3	Chinch19	-0.876	anti-tip-chimer
21F3	Chinch21	0.370	anti-tip-chimer
23F3	Chinch23	-0.473	AC-5mg/kg
20F3	Chinch20	-0.733	AC-5mg/kg
25F3	Chinch25	-0.735	AC-10mg/kg

Supplementary File 2
Longitudinal Pairwise-Distances and Pairwise-Differences

Fecal Shannon Diversity Pairwise-Differences
Baseline - 9DPT

#SampleID	SubjectID	Difference	Group
2AF9	Chinch2	-1.220	AC-PO7d
3AF9	Chinch3	-2.589	AC-PO7d
5F4	Chinch5	-0.120	Ofloxacin
7F4	Chinch7	-0.341	Ofloxacin
4F4	Chinch4	-0.125	Ofloxacin
8F4	Chinch8	-0.551	Saline
12F4	Chinch12	-0.283	TS-30mg/kg
10F4	Chinch10	-0.205	TS-30mg/kg
16F4	Chinch16	-0.976	TS-30mg/kg
11F4	Chinch11	-0.596	anti-rsPilA
17F4	Chinch17	-0.455	anti-rsPilA
18F4	Chinch18	-0.435	TS-15mg/kg
14F4	Chinch14	-0.318	TS-15mg/kg
15F4	Chinch15	-0.858	TS-15mg/kg
21F4	Chinch21	0.827	anti-tip-chimer
27F4	Chinch27	0.268	anti-tip-chimer
19F4	Chinch19	-0.534	anti-tip-chimer
23F4	Chinch23	0.107	AC-5mg/kg
20F4	Chinch20	-0.882	AC-5mg/kg
22F4	Chinch22	-0.555	AC-10mg/kg
25F4	Chinch25	-0.958	AC-10mg/kg

Supplementary File 2
Longitudinal Pairwise-Distances and Pairwise-Differences

Fecal Shannon Diversity Pairwise-Difference Mann-Whitney Pairwise Comparisons
Baseline - 2DPT

Group A	Group B	Mann-Whitney U	P-value	FDR P-value
AC-PO7d	AC-5mg/kg	0	0.333	0.993
AC-PO7d	AC-10mg/kg	0	0.667	0.993
AC-PO7d	Ofloxacin	0	0.333	0.993
AC-PO7d	Saline	0	0.200	0.993
AC-PO7d	TS-30mg/kg	0	0.200	0.993
AC-PO7d	TS-15mg/kg	0	0.200	0.993
AC-PO7d	anti-tip-chimer	0	0.200	0.993
AC-PO7d	anti-rsPilA	0	0.200	0.993
AC-5mg/kg	AC-10mg/kg	2	0.667	0.993
Ofloxacin	AC-5mg/kg	2	1.000	1.000
Ofloxacin	AC-10mg/kg	2	0.667	0.993
Ofloxacin	Saline	3	1.000	1.000
Ofloxacin	TS-30mg/kg	5	0.400	0.993
Ofloxacin	TS-15mg/kg	5	0.400	0.993
Ofloxacin	anti-tip-chimer	3	1.000	1.000
Ofloxacin	anti-rsPilA	4	0.800	0.993
Saline	AC-5mg/kg	2	0.800	0.993
Saline	AC-10mg/kg	3	0.500	0.993
Saline	TS-30mg/kg	6	0.700	0.993
Saline	TS-15mg/kg	7	0.400	0.993
Saline	anti-tip-chimer	4	1.000	1.000
Saline	anti-rsPilA	6	0.700	0.993
TS-30mg/kg	AC-5mg/kg	2	0.800	0.993
TS-30mg/kg	AC-10mg/kg	3	0.500	0.993
TS-30mg/kg	TS-15mg/kg	6	0.700	0.993
TS-30mg/kg	anti-tip-chimer	4	1.000	1.000
TS-30mg/kg	anti-rsPilA	5	1.000	1.000
TS-15mg/kg	AC-5mg/kg	2	0.800	0.993
TS-15mg/kg	AC-10mg/kg	3	0.500	0.993
TS-15mg/kg	anti-tip-chimer	3	0.700	0.993
anti-tip-chimer	AC-5mg/kg	4	0.800	0.993
anti-tip-chimer	AC-10mg/kg	3	0.500	0.993
anti-rsPilA	AC-5mg/kg	1	0.400	0.993
anti-rsPilA	AC-10mg/kg	3	0.500	0.993
anti-rsPilA	TS-15mg/kg	5	1.000	1.000
anti-rsPilA	anti-tip-chimer	3	0.700	0.993

Kruskal-Wallis (Omnibus Test)

**H**	9.277
**p-value**	0.319

Fecal Shannon Diversity Pairwise-Difference Mann-Whitney Pairwise Comparisons
Baseline - 5DPT

Group A	Group B	Mann-Whitney U	P-value	FDR P-value
AC-PO7d	AC-5mg/kg	4	0.800	0.900
AC-PO7d	AC-10mg/kg	4	0.800	0.900
AC-PO7d	Ofloxacin	3	0.700	0.900
AC-PO7d	Saline	2	0.400	0.686
AC-PO7d	TS-30mg/kg	3	0.700	0.900
AC-PO7d	TS-15mg/kg	3	0.700	0.900
AC-PO7d	anti-tip-chimer	2	0.400	0.686
AC-PO7d	anti-rsPilA	2	0.800	0.900
AC-5mg/kg	AC-10mg/kg	4	0.333	0.667
Ofloxacin	AC-5mg/kg	6	0.200	0.480
Ofloxacin	AC-10mg/kg	6	0.200	0.480
Ofloxacin	Saline	4	1.000	1.000
Ofloxacin	TS-30mg/kg	7	0.400	0.686
Ofloxacin	TS-15mg/kg	9	0.100	0.480
Ofloxacin	anti-tip-chimer	6	0.700	0.900
Ofloxacin	anti-rsPilA	6	0.200	0.480
Saline	AC-5mg/kg	6	0.200	0.480
Saline	AC-10mg/kg	6	0.200	0.480
Saline	TS-30mg/kg	8	0.200	0.480
Saline	TS-15mg/kg	9	0.100	0.480
Saline	anti-tip-chimer	5	1.000	1.000
Saline	anti-rsPilA	6	0.200	0.480
TS-30mg/kg	AC-5mg/kg	6	0.200	0.480
TS-30mg/kg	AC-10mg/kg	6	0.200	0.480
TS-30mg/kg	TS-15mg/kg	6	0.700	0.900
TS-30mg/kg	anti-tip-chimer	4	1.000	1.000
TS-30mg/kg	anti-rsPilA	4	0.800	0.900
TS-15mg/kg	AC-5mg/kg	6	0.200	0.480
TS-15mg/kg	AC-10mg/kg	6	0.200	0.480
TS-15mg/kg	anti-tip-chimer	3	0.700	0.900
anti-tip-chimer	AC-5mg/kg	6	0.200	0.480
anti-tip-chimer	AC-10mg/kg	6	0.200	0.480
anti-rsPilA	AC-5mg/kg	4	0.333	0.667
anti-rsPilA	AC-10mg/kg	4	0.333	0.667
anti-rsPilA	TS-15mg/kg	3	1.000	1.000
anti-rsPilA	anti-tip-chimer	2	0.800	0.900

Kruskal-Wallis (Omnibus Test)

**H**	13.033
**p-value**	0.111

Fecal Shannon Diversity Pairwise-Difference Mann-Whitney Pairwise Comparisons
Baseline - 7DPT

Group A	Group B	Mann-Whitney U	P-value	FDR P-value
AC-PO7d	AC-5mg/kg	0	0.200	0.554
AC-PO7d	AC-10mg/kg	0	0.500	0.947
AC-PO7d	Ofloxacin	0	0.100	0.554
AC-PO7d	Saline	0	0.200	0.554
AC-PO7d	TS-30mg/kg	0	0.100	0.554
AC-PO7d	TS-15mg/kg	0	0.100	0.554
AC-PO7d	anti-tip-chimer	0	0.200	0.554
AC-PO7d	anti-rsPilA	0	0.200	0.554
AC-5mg/kg	AC-10mg/kg	2	0.667	0.993
Ofloxacin	AC-5mg/kg	6	0.200	0.554
Ofloxacin	AC-10mg/kg	3	0.500	0.947
Ofloxacin	Saline	4	0.800	0.993
Ofloxacin	TS-30mg/kg	7	0.400	0.900
Ofloxacin	TS-15mg/kg	9	0.100	0.554
Ofloxacin	anti-tip-chimer	3	1.000	1.000
Ofloxacin	anti-rsPilA	3	1.000	1.000
Saline	AC-5mg/kg	3	0.667	0.993
Saline	AC-10mg/kg	2	0.667	0.993
Saline	TS-30mg/kg	4	0.800	0.993
Saline	TS-15mg/kg	6	0.200	0.554
Saline	anti-tip-chimer	2	1.000	1.000
Saline	anti-rsPilA	1	0.667	0.993
TS-30mg/kg	AC-5mg/kg	4	0.800	0.993
TS-30mg/kg	AC-10mg/kg	2	1.000	1.000
TS-30mg/kg	TS-15mg/kg	8	0.200	0.554
TS-30mg/kg	anti-tip-chimer	2	0.800	0.993
TS-30mg/kg	anti-rsPilA	2	0.800	0.993
TS-15mg/kg	AC-5mg/kg	0	0.200	0.554
TS-15mg/kg	AC-10mg/kg	0	0.500	0.947
TS-15mg/kg	anti-tip-chimer	1	0.400	0.900
anti-tip-chimer	AC-5mg/kg	2	1.000	1.000
anti-tip-chimer	AC-10mg/kg	1	1.000	1.000
anti-rsPilA	AC-5mg/kg	4	0.333	0.857
anti-rsPilA	AC-10mg/kg	2	0.667	0.993
anti-rsPilA	TS-15mg/kg	6	0.200	0.554
anti-rsPilA	anti-tip-chimer	2	1.000	1.000

Kruskal-Wallis (Omnibus Test)

**H**	13.948
**p-value**	0.083

Supplementary File 2
Longitudinal Pairwise-Distances and Pairwise-Differences

Fecal Shannon Diversity Pairwise-Difference Mann-Whitney Pairwise Comparisons
Baseline - 9DPT

Group A	Group B	Mann-Whitney U	P-value	FDR P-value
AC-PO7d	AC-5mg/kg	0	0.333	0.847
AC-PO7d	AC-10mg/kg	0	0.333	0.847
AC-PO7d	Ofloxacin	0	0.200	0.847
AC-PO7d	Saline	0	0.667	0.993
AC-PO7d	TS-30mg/kg	0	0.200	0.847
AC-PO7d	TS-15mg/kg	0	0.200	0.847
AC-PO7d	anti-tip-chimer	0	0.200	0.847
AC-PO7d	anti-rsPilA	0	0.333	0.847
AC-5mg/kg	AC-10mg/kg	3	0.667	0.993
Ofloxacin	AC-5mg/kg	3	1.000	1.000
Ofloxacin	AC-10mg/kg	6	0.200	0.847
Ofloxacin	Saline	3	0.500	0.947
Ofloxacin	TS-30mg/kg	7	0.400	0.847
Ofloxacin	TS-15mg/kg	8	0.200	0.847
Ofloxacin	anti-tip-chimer	3	0.700	0.993
Ofloxacin	anti-rsPilA	6	0.200	0.847
Saline	AC-5mg/kg	1	1.000	1.000
Saline	AC-10mg/kg	2	0.667	0.993
Saline	TS-30mg/kg	1	1.000	1.000
Saline	TS-15mg/kg	1	1.000	1.000
Saline	anti-tip-chimer	0	0.500	0.947
Saline	anti-rsPilA	1	1.000	1.000
TS-30mg/kg	AC-5mg/kg	2	0.800	0.993
TS-30mg/kg	AC-10mg/kg	4	0.800	0.993
TS-30mg/kg	TS-15mg/kg	6	0.700	0.993
TS-30mg/kg	anti-tip-chimer	2	0.400	0.847
TS-30mg/kg	anti-rsPilA	4	0.800	0.993
TS-15mg/kg	AC-5mg/kg	3	1.000	1.000
TS-15mg/kg	AC-10mg/kg	5	0.400	0.847
TS-15mg/kg	anti-tip-chimer	2	0.400	0.847
anti-tip-chimer	AC-5mg/kg	5	0.400	0.847
anti-tip-chimer	AC-10mg/kg	6	0.200	0.847
anti-rsPilA	AC-5mg/kg	2	1.000	1.000
anti-rsPilA	AC-10mg/kg	3	0.667	0.993
anti-rsPilA	TS-15mg/kg	2	0.800	0.993
anti-rsPilA	anti-tip-chimer	1	0.400	0.847

Kruskal-Wallis (Omnibus Test)

**H**	11.121
**p-value**	0.195

Supplementary File 2
Longitudinal Pairwise-Distances and Pairwise-Differences

NPL Weighted UniFrac Pairwise-Distances

Baseline - 2DPT

#SampleID	SubjectID	Distance	Group
3AN2	Chinch3	0.427	AC-PO7d
1AN2	Chinch1	0.474	AC-PO7d
2AN2	Chinch2	0.142	AC-PO7d
22N1	Chinch22	0.350	AC-10mg/kg
25N1	Chinch25	0.122	AC-10mg/kg
20N1	Chinch20	0.105	AC-5mg/kg
23N1	Chinch23	0.105	AC-5mg/kg
26N1	Chinch26	0.111	AC-5mg/kg
11N1	Chinch11	0.132	anti-rsPilA
13N1	Chinch13	0.143	anti-rsPilA
17N1	Chinch17	0.098	anti-rsPilA
21N1	Chinch21	0.096	anti-tip-chimer
19N1	Chinch19	0.216	anti-tip-chimer
27N1	Chinch27	0.083	anti-tip-chimer
7N1	Chinch7	0.091	Ofloxacin
4N1	Chinch4	0.103	Ofloxacin
5N1	Chinch5	0.242	Ofloxacin
10N1	Chinch10	0.145	TS-30mg/kg
12N1	Chinch12	0.114	TS-30mg/kg
16N1	Chinch16	0.123	TS-30mg/kg
15N1	Chinch15	0.287	TS-15mg/kg
18N1	Chinch18	0.094	TS-15mg/kg
14N1	Chinch14	0.209	TS-15mg/kg
8N1	Chinch8	0.144	Saline
9N1	Chinch9	0.111	Saline
6N1	Chinch6	0.085	Saline

Supplementary File 2
Longitudinal Pairwise-Distances and Pairwise-Differences

NPL Weighted UniFrac Pairwise-Distances
Baseline - 5DPT

#SampleID	SubjectID	Distance	Group
3AN5	Chinch3	0.354	AC-PO7d
2AN5	Chinch2	0.350	AC-PO7d
1AN5	Chinch1	0.414	AC-PO7d
22N2	Chinch22	0.195	AC-10mg/kg
25N2	Chinch25	0.442	AC-10mg/kg
26N2	Chinch26	0.215	AC-5mg/kg
20N2	Chinch20	0.110	AC-5mg/kg
23N2	Chinch23	0.115	AC-5mg/kg
11N2	Chinch11	0.142	anti-rsPilA
17N2	Chinch17	0.111	anti-rsPilA
21N2	Chinch21	0.080	anti-tip-chimer
19N2	Chinch19	0.150	anti-tip-chimer
27N2	Chinch27	0.078	anti-tip-chimer
4N2	Chinch4	0.088	Ofloxacin
5N2	Chinch5	0.656	Ofloxacin
7N2	Chinch7	0.031	Ofloxacin
16N2	Chinch16	0.155	TS-30mg/kg
12N2	Chinch12	0.185	TS-30mg/kg
10N2	Chinch10	0.149	TS-30mg/kg
18N2	Chinch18	0.070	TS-15mg/kg
14N2	Chinch14	0.074	TS-15mg/kg
15N2	Chinch15	0.219	TS-15mg/kg
9N2	Chinch9	0.570	Saline
8N2	Chinch8	0.170	Saline
6N2	Chinch6	0.089	Saline

Supplementary File 2
Longitudinal Pairwise-Distances and Pairwise-Differences

NPL Weighted UniFrac Pairwise-Distances

Baseline - 7DPT

#SampleID	SubjectID	Distance	Group
1AN7	Chinch1	0.343	AC-PO7d
2AN7	Chinch2	0.440	AC-PO7d
25N3	Chinch25	0.298	AC-10mg/kg
20N3	Chinch20	0.072	AC-5mg/kg
26N3	Chinch26	0.127	AC-5mg/kg
23N3	Chinch23	0.114	AC-5mg/kg
17N3	Chinch17	0.238	anti-rsPilA
11N3	Chinch11	0.218	anti-rsPilA
27N3	Chinch27	0.105	anti-tip-chimer
21N3	Chinch21	0.161	anti-tip-chimer
5N3	Chinch5	0.330	Ofloxacin
7N3	Chinch7	0.056	Ofloxacin
4N3	Chinch4	0.104	Ofloxacin
16N3	Chinch16	0.266	TS-30mg/kg
10N3	Chinch10	0.106	TS-30mg/kg
18N3	Chinch18	0.078	TS-15mg/kg
15N3	Chinch15	0.130	TS-15mg/kg
14N3	Chinch14	0.105	TS-15mg/kg
6N3	Chinch6	0.127	Saline
9N3	Chinch9	0.546	Saline
8N3	Chinch8	0.164	Saline

Supplementary File 2
Longitudinal Pairwise-Distances and Pairwise-Differences

NPL Weighted UniFrac Pairwise-Distances
Baseline - 9DPT

#SampleID	SubjectID	Distance	Group
3AN9	Chinch3	0.205	AC-PO7d
2AN9	Chinch2	0.351	AC-PO7d
22N4	Chinch22	0.345	AC-10mg/kg
25N4	Chinch25	0.349	AC-10mg/kg
20N4	Chinch20	0.150	AC-5mg/kg
26N4	Chinch26	0.151	AC-5mg/kg
23N4	Chinch23	0.095	AC-5mg/kg
11N4	Chinch11	0.188	anti-rsPilA
17N4	Chinch17	0.108	anti-rsPilA
21N4	Chinch21	0.229	anti-tip-chimer
19N4	Chinch19	0.178	anti-tip-chimer
27N4	Chinch27	0.073	anti-tip-chimer
7N4	Chinch7	0.044	Ofloxacin
5N4	Chinch5	0.361	Ofloxacin
10N4	Chinch10	0.148	TS-30mg/kg
12N4	Chinch12	0.147	TS-30mg/kg
16N4	Chinch16	0.334	TS-30mg/kg
15N4	Chinch15	0.182	TS-15mg/kg
14N4	Chinch14	0.115	TS-15mg/kg
18N4	Chinch18	0.073	TS-15mg/kg
6N4	Chinch6	0.128	Saline
8N4	Chinch8	0.186	Saline

Supplementary File 2
Longitudinal Pairwise-Distances and Pairwise-Differences

NPL Weighted UniFrac Pairwise-Distance Mann-Whitney Pairwise Comparisons
Baseline - 2DPT

Group A	Group B	Mann-Whitney U	P-value	FDR P-value
AC-PO7d	AC-5mg/kg	9	0.100	0.900
AC-PO7d	AC-10mg/kg	5	0.400	0.900
AC-PO7d	Ofloxacin	8	0.200	0.900
AC-PO7d	Saline	8	0.200	0.900
AC-PO7d	TS-30mg/kg	8	0.200	0.900
AC-PO7d	TS-15mg/kg	7	0.400	0.900
AC-PO7d	anti-tip-chimer	8	0.200	0.900
AC-PO7d	anti-rsPilA	8	0.200	0.900
AC-5mg/kg	AC-10mg/kg	0	0.200	0.900
AC-5mg/kg	Ofloxacin	6	0.700	0.900
AC-5mg/kg	Saline	3	0.700	0.900
AC-10mg/kg	Ofloxacin	5	0.400	0.900
AC-10mg/kg	Saline	5	0.400	0.900
Ofloxacin	Saline	5	1.000	1.000
TS-30mg/kg	AC-5mg/kg	9	0.100	0.900
TS-30mg/kg	AC-10mg/kg	2	0.800	0.929
TS-30mg/kg	Ofloxacin	6	0.700	0.900
TS-30mg/kg	Saline	7	0.400	0.900
TS-30mg/kg	TS-15mg/kg	3	0.700	0.900
TS-30mg/kg	anti-tip-chimer	6	0.700	0.900
TS-30mg/kg	anti-rsPilA	5	1.000	1.000
TS-15mg/kg	AC-5mg/kg	6	0.700	0.900
TS-15mg/kg	AC-10mg/kg	2	0.800	0.929
TS-15mg/kg	Ofloxacin	6	0.700	0.900
TS-15mg/kg	Saline	7	0.400	0.900
TS-15mg/kg	anti-tip-chimer	6	0.700	0.900
anti-tip-chimer	AC-5mg/kg	3	0.700	0.900
anti-tip-chimer	AC-10mg/kg	1	0.400	0.900
anti-tip-chimer	Ofloxacin	3	0.700	0.900
anti-tip-chimer	Saline	4	1.000	1.000
anti-rsPilA	AC-5mg/kg	6	0.700	0.900
anti-rsPilA	AC-10mg/kg	2	0.800	0.929
anti-rsPilA	Ofloxacin	5	1.000	1.000
anti-rsPilA	Saline	5	1.000	1.000
anti-rsPilA	TS-15mg/kg	3	0.700	0.900
anti-rsPilA	anti-tip-chimer	6	0.700	0.900

Kruskal-Wallis (Omnibus Test)

**H**	8.251
**p-value**	0.409

Supplementary File 2
Longitudinal Pairwise-Distances and Pairwise-Differences

NPL Weighted UniFrac Pairwise-Distance Mann-Whitney Pairwise Comparisons
Baseline - 5DPT

Group A	Group B	Mann-Whitney U	P-value	FDR P-value
AC-PO7d	AC-5mg/kg	9	0.100	0.720
AC-PO7d	AC-10mg/kg	3	1.000	1.000
AC-PO7d	Ofloxacin	6	0.700	0.960
AC-PO7d	Saline	6	0.700	0.960
AC-PO7d	TS-30mg/kg	9	0.100	0.720
AC-PO7d	TS-15mg/kg	9	0.100	0.720
AC-PO7d	anti-tip-chimer	9	0.100	0.720
AC-PO7d	anti-rsPilA	6	0.200	0.720
AC-5mg/kg	AC-10mg/kg	1	0.400	0.960
AC-5mg/kg	Ofloxacin	6	0.700	0.960
AC-5mg/kg	Saline	4	1.000	1.000
AC-10mg/kg	Ofloxacin	4	0.800	0.960
AC-10mg/kg	Saline	4	0.800	0.960
Ofloxacin	Saline	3	0.700	0.960
TS-30mg/kg	AC-5mg/kg	6	0.700	0.960
TS-30mg/kg	AC-10mg/kg	0	0.200	0.720
TS-30mg/kg	Ofloxacin	6	0.700	0.960
TS-30mg/kg	Saline	4	1.000	1.000
TS-30mg/kg	TS-15mg/kg	6	0.700	0.960
TS-30mg/kg	anti-tip-chimer	8	0.200	0.720
TS-30mg/kg	anti-rsPilA	6	0.200	0.720
TS-15mg/kg	AC-5mg/kg	3	0.700	0.960
TS-15mg/kg	AC-10mg/kg	1	0.400	0.960
TS-15mg/kg	Ofloxacin	4	1.000	1.000
TS-15mg/kg	Saline	2	0.400	0.960
TS-15mg/kg	anti-tip-chimer	3	0.700	0.960
anti-tip-chimer	AC-5mg/kg	2	0.400	0.960
anti-tip-chimer	AC-10mg/kg	0	0.200	0.720
anti-tip-chimer	Ofloxacin	4	1.000	1.000
anti-tip-chimer	Saline	1	0.200	0.720
anti-rsPilA	AC-5mg/kg	3	1.000	1.000
anti-rsPilA	AC-10mg/kg	0	0.333	0.960
anti-rsPilA	Ofloxacin	4	0.800	0.960
anti-rsPilA	Saline	2	0.800	0.960
anti-rsPilA	TS-15mg/kg	4	0.800	0.960
anti-rsPilA	anti-tip-chimer	4	0.800	0.960

Kruskal-Wallis (Omnibus Test)

**H**	9.563
**p-value**	0.297

Supplementary File 2
Longitudinal Pairwise-Distances and Pairwise-Differences

NPL Weighted UniFrac Pairwise-Distance Mann-Whitney Pairwise Comparisons
Baseline - 7DPT

Group A	Group B	Mann-Whitney U	P-value	FDR P-value
AC-PO7d	AC-5mg/kg	6	0.200	0.960
AC-PO7d	AC-10mg/kg	2	0.667	0.960
AC-PO7d	Ofloxacin	6	0.200	0.960
AC-PO7d	Saline	4	0.800	0.960
AC-PO7d	TS-30mg/kg	4	0.333	0.960
AC-PO7d	TS-15mg/kg	6	0.200	0.960
AC-PO7d	anti-tip-chimer	4	0.333	0.960
AC-PO7d	anti-rsPilA	4	0.333	0.960
AC-5mg/kg	AC-10mg/kg	0	0.500	0.960
AC-5mg/kg	Ofloxacin	5	1.000	1.000
AC-5mg/kg	Saline	0	0.100	0.960
AC-10mg/kg	Ofloxacin	2	1.000	1.000
AC-10mg/kg	Saline	2	1.000	1.000
Ofloxacin	Saline	2	0.400	0.960
TS-30mg/kg	AC-5mg/kg	4	0.800	0.960
TS-30mg/kg	AC-10mg/kg	0	0.667	0.960
TS-30mg/kg	Ofloxacin	4	0.800	0.960
TS-30mg/kg	Saline	2	0.800	0.960
TS-30mg/kg	TS-15mg/kg	5	0.400	0.960
TS-30mg/kg	anti-tip-chimer	3	0.667	0.960
TS-30mg/kg	anti-rsPilA	2	1.000	1.000
TS-15mg/kg	AC-5mg/kg	5	1.000	1.000
TS-15mg/kg	AC-10mg/kg	0	0.500	0.960
TS-15mg/kg	Ofloxacin	5	1.000	1.000
TS-15mg/kg	Saline	1	0.200	0.960
TS-15mg/kg	anti-tip-chimer	1	0.400	0.960
anti-tip-chimer	AC-5mg/kg	4	0.800	0.960
anti-tip-chimer	AC-10mg/kg	0	0.667	0.960
anti-tip-chimer	Ofloxacin	4	0.800	0.960
anti-tip-chimer	Saline	1	0.400	0.960
anti-rsPilA	AC-5mg/kg	6	0.200	0.960
anti-rsPilA	AC-10mg/kg	0	0.667	0.960
anti-rsPilA	Ofloxacin	4	0.800	0.960
anti-rsPilA	Saline	4	0.800	0.960
anti-rsPilA	TS-15mg/kg	6	0.200	0.960
anti-rsPilA	anti-tip-chimer	4	0.333	0.960

Kruskal-Wallis (Omnibus Test)

**H**	10.853
**p-value**	0.210

Supplementary File 2
Longitudinal Pairwise-Distances and Pairwise-Differences

NPL Weighted UniFrac Pairwise-Distance Mann-Whitney Pairwise Comparisons
Baseline - 9DPT

Group A	Group B	Mann-Whitney U	P-value	FDR P-value
AC-PO7d	AC-5mg/kg	6	0.200	1.000
AC-PO7d	AC-10mg/kg	2	1.000	1.000
AC-PO7d	Ofloxacin	2	1.000	1.000
AC-PO7d	Saline	4	0.333	1.000
AC-PO7d	TS-30mg/kg	5	0.400	1.000
AC-PO7d	TS-15mg/kg	6	0.200	1.000
AC-PO7d	anti-tip-chimer	5	0.400	1.000
AC-PO7d	anti-rsPilA	4	0.333	1.000
AC-5mg/kg	AC-10mg/kg	0	0.200	1.000
AC-5mg/kg	Ofloxacin	3	1.000	1.000
AC-5mg/kg	Saline	2	0.800	1.000
AC-10mg/kg	Ofloxacin	2	1.000	1.000
AC-10mg/kg	Saline	4	0.333	1.000
Ofloxacin	Saline	2	1.000	1.000
TS-30mg/kg	AC-5mg/kg	5	1.000	1.000
TS-30mg/kg	AC-10mg/kg	0	0.200	1.000
TS-30mg/kg	Ofloxacin	3	1.000	1.000
TS-30mg/kg	Saline	4	0.800	1.000
TS-30mg/kg	TS-15mg/kg	7	0.400	1.000
TS-30mg/kg	anti-tip-chimer	5	1.000	1.000
TS-30mg/kg	anti-rsPilA	4	0.800	1.000
TS-15mg/kg	AC-5mg/kg	4	1.000	1.000
TS-15mg/kg	AC-10mg/kg	0	0.200	1.000
TS-15mg/kg	Ofloxacin	3	1.000	1.000
TS-15mg/kg	Saline	1	0.400	1.000
TS-15mg/kg	anti-tip-chimer	4	1.000	1.000
anti-tip-chimer	AC-5mg/kg	6	0.700	1.000
anti-tip-chimer	AC-10mg/kg	0	0.200	1.000
anti-tip-chimer	Ofloxacin	3	1.000	1.000
anti-tip-chimer	Saline	3	1.000	1.000
anti-rsPilA	AC-5mg/kg	4	0.800	1.000
anti-rsPilA	AC-10mg/kg	0	0.333	1.000
anti-rsPilA	Ofloxacin	2	1.000	1.000
anti-rsPilA	Saline	2	1.000	1.000
anti-rsPilA	TS-15mg/kg	4	0.800	1.000
anti-rsPilA	anti-tip-chimer	3	1.000	1.000

Kruskal-Wallis (Omnibus Test)

**H**	7.561
**p-value**	0.477

Supplementary File 2
Longitudinal Pairwise-Distances and Pairwise-Differences

NPL Bray Curtis Pairwise-Distances

Baseline - 2DPT

#SampleID	SubjectID	Distance	Group
1AN2	Chinch1	0.952	AC-PO7d
3AN2	Chinch3	0.962	AC-PO7d
2AN2	Chinch2	0.353	AC-PO7d
12N1	Chinch12	0.345	TS-30mg/kg
16N1	Chinch16	0.391	TS-30mg/kg
10N1	Chinch10	0.393	TS-30mg/kg
13N1	Chinch13	0.387	anti-rsPilA
17N1	Chinch17	0.288	anti-rsPilA
11N1	Chinch11	0.480	anti-rsPilA
15N1	Chinch15	0.684	TS-15mg/kg
18N1	Chinch18	0.303	TS-15mg/kg
14N1	Chinch14	0.418	TS-15mg/kg
27N1	Chinch27	0.269	anti-tip-chimer
19N1	Chinch19	0.520	anti-tip-chimer
21N1	Chinch21	0.334	anti-tip-chimer
26N1	Chinch26	0.345	AC-5mg/kg
23N1	Chinch23	0.350	AC-5mg/kg
20N1	Chinch20	0.343	AC-5mg/kg
25N1	Chinch25	0.372	AC-10mg/kg
22N1	Chinch22	0.603	AC-10mg/kg
4N1	Chinch4	0.372	Ofloxacin
5N1	Chinch5	0.568	Ofloxacin
7N1	Chinch7	0.278	Ofloxacin
9N1	Chinch9	0.478	Saline
6N1	Chinch6	0.225	Saline
8N1	Chinch8	0.564	Saline

Supplementary File 2
Longitudinal Pairwise-Distances and Pairwise-Differences

NPL Bray Curtis Pairwise-Distances

Baseline - 5DPT

#SampleID	SubjectID	Distance	Group
3AN5	Chinch3	0.853	AC-PO7d
2AN5	Chinch2	0.785	AC-PO7d
1AN5	Chinch1	0.893	AC-PO7d
10N2	Chinch10	0.473	TS-30mg/kg
12N2	Chinch12	0.405	TS-30mg/kg
16N2	Chinch16	0.443	TS-30mg/kg
17N2	Chinch17	0.325	anti-rsPilA
11N2	Chinch11	0.506	anti-rsPilA
15N2	Chinch15	0.710	TS-15mg/kg
18N2	Chinch18	0.341	TS-15mg/kg
14N2	Chinch14	0.338	TS-15mg/kg
21N2	Chinch21	0.398	anti-tip-chimer
19N2	Chinch19	0.367	anti-tip-chimer
27N2	Chinch27	0.276	anti-tip-chimer
20N2	Chinch20	0.288	AC-5mg/kg
26N2	Chinch26	0.542	AC-5mg/kg
23N2	Chinch23	0.351	AC-5mg/kg
25N2	Chinch25	0.667	AC-10mg/kg
22N2	Chinch22	0.501	AC-10mg/kg
5N2	Chinch5	0.957	Ofloxacin
7N2	Chinch7	0.281	Ofloxacin
4N2	Chinch4	0.373	Ofloxacin
9N2	Chinch9	0.978	Saline
8N2	Chinch8	0.560	Saline
6N2	Chinch6	0.284	Saline

Supplementary File 2
Longitudinal Pairwise-Distances and Pairwise-Differences

NPL Bray Curtis Pairwise-Distances

Baseline - 7DPT

#SampleID	SubjectID	Distance	Group
1AN7	Chinch1	0.747	AC-PO7d
2AN7	Chinch2	0.897	AC-PO7d
16N3	Chinch16	0.623	TS-30mg/kg
10N3	Chinch10	0.442	TS-30mg/kg
17N3	Chinch17	0.483	anti-rsPilA
11N3	Chinch11	0.649	anti-rsPilA
14N3	Chinch14	0.365	TS-15mg/kg
15N3	Chinch15	0.652	TS-15mg/kg
18N3	Chinch18	0.298	TS-15mg/kg
21N3	Chinch21	0.442	anti-tip-chimer
27N3	Chinch27	0.310	anti-tip-chimer
26N3	Chinch26	0.310	AC-5mg/kg
20N3	Chinch20	0.285	AC-5mg/kg
23N3	Chinch23	0.306	AC-5mg/kg
25N3	Chinch25	0.583	AC-10mg/kg
5N3	Chinch5	0.695	Ofloxacin
4N3	Chinch4	0.402	Ofloxacin
7N3	Chinch7	0.228	Ofloxacin
8N3	Chinch8	0.603	Saline
6N3	Chinch6	0.347	Saline
9N3	Chinch9	0.951	Saline

Supplementary File 2
Longitudinal Pairwise-Distances and Pairwise-Differences

NPL Bray Curtis Pairwise-Distances

Baseline - 9DPT

#SampleID	SubjectID	Distance	Group
3AN9	Chinch3	0.439	AC-PO7d
2AN9	Chinch2	0.833	AC-PO7d
12N4	Chinch12	0.431	TS-30mg/kg
16N4	Chinch16	0.651	TS-30mg/kg
10N4	Chinch10	0.444	TS-30mg/kg
17N4	Chinch17	0.296	anti-rsPilA
11N4	Chinch11	0.652	anti-rsPilA
15N4	Chinch15	0.712	TS-15mg/kg
14N4	Chinch14	0.354	TS-15mg/kg
18N4	Chinch18	0.285	TS-15mg/kg
19N4	Chinch19	0.423	anti-tip-chimer
27N4	Chinch27	0.312	anti-tip-chimer
21N4	Chinch21	0.576	anti-tip-chimer
20N4	Chinch20	0.351	AC-5mg/kg
23N4	Chinch23	0.369	AC-5mg/kg
26N4	Chinch26	0.341	AC-5mg/kg
22N4	Chinch22	0.684	AC-10mg/kg
25N4	Chinch25	0.591	AC-10mg/kg
7N4	Chinch7	0.197	Ofloxacin
5N4	Chinch5	0.689	Ofloxacin
6N4	Chinch6	0.312	Saline
8N4	Chinch8	0.638	Saline

Supplementary File 2
Longitudinal Pairwise-Distances and Pairwise-Differences

NPL Bray-Curtis Pairwise-Distance Mann-Whitney Pairwise Comparisons
Baseline - 2DPT

Group A	Group B	Mann-Whitney U	P-value	FDR P-value
AC-PO7d	AC-5mg/kg	9	0.100	1.000
AC-PO7d	AC-10mg/kg	4	0.800	1.000
AC-PO7d	Ofloxacin	7	0.400	1.000
AC-PO7d	Saline	7	0.400	1.000
AC-PO7d	TS-30mg/kg	7	0.400	1.000
AC-PO7d	TS-15mg/kg	7	0.400	1.000
AC-PO7d	anti-tip-chimer	8	0.200	1.000
AC-PO7d	anti-rsPilA	7	0.400	1.000
AC-5mg/kg	AC-10mg/kg	0	0.200	1.000
AC-5mg/kg	Ofloxacin	3	0.700	1.000
AC-5mg/kg	Saline	3	0.700	1.000
AC-10mg/kg	Ofloxacin	5	0.400	1.000
AC-10mg/kg	Saline	4	0.800	1.000
Ofloxacin	Saline	5	1.000	1.000
TS-30mg/kg	AC-5mg/kg	7.5	0.268	1.000
TS-30mg/kg	AC-10mg/kg	2	0.800	1.000
TS-30mg/kg	Ofloxacin	5	1.000	1.000
TS-30mg/kg	Saline	3	0.700	1.000
TS-30mg/kg	TS-15mg/kg	3	0.700	1.000
TS-30mg/kg	anti-tip-chimer	6	0.700	1.000
TS-30mg/kg	anti-rsPilA	5	1.000	1.000
TS-15mg/kg	AC-5mg/kg	6	0.700	1.000
TS-15mg/kg	AC-10mg/kg	3	1.000	1.000
TS-15mg/kg	Ofloxacin	6	0.700	1.000
TS-15mg/kg	Saline	5	1.000	1.000
TS-15mg/kg	anti-tip-chimer	6	0.700	1.000
anti-tip-chimer	AC-5mg/kg	3	0.700	1.000
anti-tip-chimer	AC-10mg/kg	1	0.400	1.000
anti-tip-chimer	Ofloxacin	3	0.700	1.000
anti-tip-chimer	Saline	4	1.000	1.000
anti-rsPilA	AC-5mg/kg	6	0.700	1.000
anti-rsPilA	AC-10mg/kg	2	0.800	1.000
anti-rsPilA	Ofloxacin	5	1.000	1.000
anti-rsPilA	Saline	4	1.000	1.000
anti-rsPilA	TS-15mg/kg	3	0.700	1.000
anti-rsPilA	anti-tip-chimer	5	1.000	1.000

Kruskal-Wallis (Omnibus Test)

**H**	5.820
**p-value**	0.667

Supplementary File 2
Longitudinal Pairwise-Distances and Pairwise-Differences

NPL Bray-Curtis Pairwise-Distance Mann-Whitney Pairwise Comparisons
Baseline - 5DPT

Group A	Group B	Mann-Whitney U	P-value	FDR P-value
AC-PO7d	AC-5mg/kg	9	0.100	0.720
AC-PO7d	AC-10mg/kg	6	0.200	0.800
AC-PO7d	Ofloxacin	6	0.700	1.000
AC-PO7d	Saline	6	0.700	1.000
AC-PO7d	TS-30mg/kg	9	0.100	0.720
AC-PO7d	TS-15mg/kg	9	0.100	0.720
AC-PO7d	anti-tip-chimer	9	0.100	0.720
AC-PO7d	anti-rsPilA	6	0.200	0.800
AC-5mg/kg	AC-10mg/kg	1	0.400	1.000
AC-5mg/kg	Ofloxacin	4	1.000	1.000
AC-5mg/kg	Saline	3	0.700	1.000
AC-10mg/kg	Ofloxacin	4	0.800	1.000
AC-10mg/kg	Saline	3	1.000	1.000
Ofloxacin	Saline	3	0.700	1.000
TS-30mg/kg	AC-5mg/kg	6	0.700	1.000
TS-30mg/kg	AC-10mg/kg	0	0.200	0.800
TS-30mg/kg	Ofloxacin	6	0.700	1.000
TS-30mg/kg	Saline	3	0.700	1.000
TS-30mg/kg	TS-15mg/kg	6	0.700	1.000
TS-30mg/kg	anti-tip-chimer	9	0.100	0.720
TS-30mg/kg	anti-rsPilA	3	1.000	1.000
TS-15mg/kg	AC-5mg/kg	5	1.000	1.000
TS-15mg/kg	AC-10mg/kg	2	0.800	1.000
TS-15mg/kg	Ofloxacin	4	1.000	1.000
TS-15mg/kg	Saline	4	1.000	1.000
TS-15mg/kg	anti-tip-chimer	5	1.000	1.000
anti-tip-chimer	AC-5mg/kg	4	1.000	1.000
anti-tip-chimer	AC-10mg/kg	0	0.200	0.800
anti-tip-chimer	Ofloxacin	3	0.700	1.000
anti-tip-chimer	Saline	2	0.400	1.000
anti-rsPilA	AC-5mg/kg	3	1.000	1.000
anti-rsPilA	AC-10mg/kg	1	0.667	1.000
anti-rsPilA	Ofloxacin	3	1.000	1.000
anti-rsPilA	Saline	2	0.800	1.000
anti-rsPilA	TS-15mg/kg	2	0.800	1.000
anti-rsPilA	anti-tip-chimer	4	0.800	1.000

Kruskal-Wallis (Omnibus Test)

**H**	8.495
**p-value**	0.387

Supplementary File 2
Longitudinal Pairwise-Distances and Pairwise-Differences

NPL Bray-Curtis Pairwise-Distance Mann-Whitney Pairwise Comparisons
Baseline - 7DPT

Group A	Group B	Mann-Whitney U	P-value	FDR P-value
AC-PO7d	AC-5mg/kg	6	0.200	1.000
AC-PO7d	AC-10mg/kg	2	0.667	1.000
AC-PO7d	Ofloxacin	6	0.200	1.000
AC-PO7d	Saline	4	0.800	1.000
AC-PO7d	TS-30mg/kg	4	0.333	1.000
AC-PO7d	TS-15mg/kg	6	0.200	1.000
AC-PO7d	anti-tip-chimer	4	0.333	1.000
AC-PO7d	anti-rsPilA	4	0.333	1.000
AC-5mg/kg	AC-10mg/kg	0	0.500	1.000
AC-5mg/kg	Ofloxacin	3	0.700	1.000
AC-5mg/kg	Saline	0	0.100	1.000
AC-10mg/kg	Ofloxacin	2	1.000	1.000
AC-10mg/kg	Saline	1	1.000	1.000
Ofloxacin	Saline	3	0.700	1.000
TS-30mg/kg	AC-5mg/kg	6	0.200	1.000
TS-30mg/kg	AC-10mg/kg	1	1.000	1.000
TS-30mg/kg	Ofloxacin	4	0.800	1.000
TS-30mg/kg	Saline	3	1.000	1.000
TS-30mg/kg	TS-15mg/kg	4	0.800	1.000
TS-30mg/kg	anti-tip-chimer	3.5	0.414	1.000
TS-30mg/kg	anti-rsPilA	1	0.667	1.000
TS-15mg/kg	AC-5mg/kg	7	0.400	1.000
TS-15mg/kg	AC-10mg/kg	1	1.000	1.000
TS-15mg/kg	Ofloxacin	4	1.000	1.000
TS-15mg/kg	Saline	3	0.700	1.000
TS-15mg/kg	anti-tip-chimer	3	1.000	1.000
anti-tip-chimer	AC-5mg/kg	5.5	0.236	1.000
anti-tip-chimer	AC-10mg/kg	0	0.667	1.000
anti-tip-chimer	Ofloxacin	3	1.000	1.000
anti-tip-chimer	Saline	1	0.400	1.000
anti-rsPilA	AC-5mg/kg	6	0.200	1.000
anti-rsPilA	AC-10mg/kg	1	1.000	1.000
anti-rsPilA	Ofloxacin	4	0.800	1.000
anti-rsPilA	Saline	3	1.000	1.000
anti-rsPilA	TS-15mg/kg	4	0.800	1.000
anti-rsPilA	anti-tip-chimer	4	0.333	1.000

Kruskal-Wallis (Omnibus Test)

**H**	10.101
**p-value**	0.258

Supplementary File 2
Longitudinal Pairwise-Distances and Pairwise-Differences

NPL Bray-Curtis Pairwise-Distance Mann-Whitney Pairwise Comparisons
Baseline - 9DPT

Group A	Group B	Mann-Whitney U	P-value	FDR P-value
AC-PO7d	AC-5mg/kg	6	0.200	1.000
AC-PO7d	AC-10mg/kg	2	1.000	1.000
AC-PO7d	Ofloxacin	3	0.667	1.000
AC-PO7d	Saline	3	0.667	1.000
AC-PO7d	TS-30mg/kg	4	0.800	1.000
AC-PO7d	TS-15mg/kg	5	0.400	1.000
AC-PO7d	anti-tip-chimer	5	0.400	1.000
AC-PO7d	anti-rsPilA	3	0.667	1.000
AC-5mg/kg	AC-10mg/kg	0	0.200	1.000
AC-5mg/kg	Ofloxacin	3	1.000	1.000
AC-5mg/kg	Saline	3	1.000	1.000
AC-10mg/kg	Ofloxacin	2	1.000	1.000
AC-10mg/kg	Saline	3	0.667	1.000
Ofloxacin	Saline	2	1.000	1.000
TS-30mg/kg	AC-5mg/kg	9	0.100	1.000
TS-30mg/kg	AC-10mg/kg	1	0.400	1.000
TS-30mg/kg	Ofloxacin	3	1.000	1.000
TS-30mg/kg	Saline	4	0.800	1.000
TS-30mg/kg	TS-15mg/kg	6	0.700	1.000
TS-30mg/kg	anti-tip-chimer	7	0.400	1.000
TS-30mg/kg	anti-rsPilA	3	1.000	1.000
TS-15mg/kg	AC-5mg/kg	5	1.000	1.000
TS-15mg/kg	AC-10mg/kg	2	0.800	1.000
TS-15mg/kg	Ofloxacin	4	0.800	1.000
TS-15mg/kg	Saline	3	1.000	1.000
TS-15mg/kg	anti-tip-chimer	4	1.000	1.000
anti-tip-chimer	AC-5mg/kg	6	0.700	1.000
anti-tip-chimer	AC-10mg/kg	0	0.200	1.000
anti-tip-chimer	Ofloxacin	3	1.000	1.000
anti-tip-chimer	Saline	3	1.000	1.000
anti-rsPilA	AC-5mg/kg	3	1.000	1.000
anti-rsPilA	AC-10mg/kg	1	0.667	1.000
anti-rsPilA	Ofloxacin	2	1.000	1.000
anti-rsPilA	Saline	2	1.000	1.000
anti-rsPilA	TS-15mg/kg	3	1.000	1.000
anti-rsPilA	anti-tip-chimer	3	1.000	1.000

Kruskal-Wallis (Omnibus Test)

**H**	4.976
**p-value**	0.760

Supplementary File 2
Longitudinal Pairwise-Distances and Pairwise-Differences

NPL Jaccard Pairwise-Distances

Baseline - 2DPT

#SampleID	SubjectID	Distance	Group
3AN2	Chinch3	0.937	AC-PO7d
2AN2	Chinch2	0.505	AC-PO7d
1AN2	Chinch1	0.909	AC-PO7d
16N1	Chinch16	0.646	TS-30mg/kg
10N1	Chinch10	0.622	TS-30mg/kg
12N1	Chinch12	0.506	TS-30mg/kg
13N1	Chinch13	0.438	anti-rsPilA
11N1	Chinch11	0.612	anti-rsPilA
17N1	Chinch17	0.447	anti-rsPilA
15N1	Chinch15	0.795	TS-15mg/kg
18N1	Chinch18	0.526	TS-15mg/kg
14N1	Chinch14	0.592	TS-15mg/kg
27N1	Chinch27	0.429	anti-tip-chimer
21N1	Chinch21	0.531	anti-tip-chimer
19N1	Chinch19	0.614	anti-tip-chimer
20N1	Chinch20	0.581	AC-5mg/kg
26N1	Chinch26	0.586	AC-5mg/kg
23N1	Chinch23	0.563	AC-5mg/kg
25N1	Chinch25	0.512	AC-10mg/kg
22N1	Chinch22	0.622	AC-10mg/kg
5N1	Chinch5	0.560	Ofloxacin
7N1	Chinch7	0.397	Ofloxacin
4N1	Chinch4	0.618	Ofloxacin
9N1	Chinch9	0.691	Saline
6N1	Chinch6	0.478	Saline
8N1	Chinch8	0.671	Saline

Supplementary File 2

Longitudinal Pairwise-Distances and Pairwise-Differences

NPL Jaccard Pairwise-Distances

Baseline - 5DPT

#SampleID	SubjectID	Distance	Group
2AN5	Chinch2	0.727	AC-PO7d
3AN5	Chinch3	0.841	AC-PO7d
1AN5	Chinch1	0.856	AC-PO7d
16N2	Chinch16	0.681	TS-30mg/kg
12N2	Chinch12	0.524	TS-30mg/kg
10N2	Chinch10	0.623	TS-30mg/kg
17N2	Chinch17	0.512	anti-rsPilA
11N2	Chinch11	0.650	anti-rsPilA
15N2	Chinch15	0.744	TS-15mg/kg
18N2	Chinch18	0.589	TS-15mg/kg
14N2	Chinch14	0.590	TS-15mg/kg
19N2	Chinch19	0.506	anti-tip-chimer
27N2	Chinch27	0.480	anti-tip-chimer
21N2	Chinch21	0.651	anti-tip-chimer
26N2	Chinch26	0.602	AC-5mg/kg
20N2	Chinch20	0.463	AC-5mg/kg
23N2	Chinch23	0.576	AC-5mg/kg
25N2	Chinch25	0.643	AC-10mg/kg
22N2	Chinch22	0.735	AC-10mg/kg
5N2	Chinch5	0.950	Ofloxacin
7N2	Chinch7	0.443	Ofloxacin
4N2	Chinch4	0.559	Ofloxacin
6N2	Chinch6	0.558	Saline
8N2	Chinch8	0.667	Saline
9N2	Chinch9	0.986	Saline

Supplementary File 2
Longitudinal Pairwise-Distances and Pairwise-Differences

NPL Jaccard Pairwise-Distances

Baseline - 7DPT

#SampleID	SubjectID	Distance	Group
1AN7	Chinch1	0.755	AC-PO7d
2AN7	Chinch2	0.805	AC-PO7d
10N3	Chinch10	0.658	TS-30mg/kg
16N3	Chinch16	0.697	TS-30mg/kg
11N3	Chinch11	0.661	anti-rsPilA
17N3	Chinch17	0.564	anti-rsPilA
15N3	Chinch15	0.719	TS-15mg/kg
14N3	Chinch14	0.590	TS-15mg/kg
18N3	Chinch18	0.568	TS-15mg/kg
27N3	Chinch27	0.531	anti-tip-chimer
21N3	Chinch21	0.633	anti-tip-chimer
26N3	Chinch26	0.514	AC-5mg/kg
23N3	Chinch23	0.580	AC-5mg/kg
20N3	Chinch20	0.558	AC-5mg/kg
25N3	Chinch25	0.695	AC-10mg/kg
7N3	Chinch7	0.406	Ofloxacin
4N3	Chinch4	0.574	Ofloxacin
5N3	Chinch5	0.679	Ofloxacin
8N3	Chinch8	0.739	Saline
6N3	Chinch6	0.620	Saline
9N3	Chinch9	0.889	Saline

Supplementary File 2
Longitudinal Pairwise-Distances and Pairwise-Differences

NPL Jaccard Pairwise-Distances

Baseline - 9DPT

#SampleID	SubjectID	Distance	Group
2AN9	Chinch2	0.620	AC-PO7d
3AN9	Chinch3	0.803	AC-PO7d
12N4	Chinch12	0.622	TS-30mg/kg
10N4	Chinch10	0.684	TS-30mg/kg
16N4	Chinch16	0.742	TS-30mg/kg
17N4	Chinch17	0.518	anti-rsPilA
11N4	Chinch11	0.748	anti-rsPilA
18N4	Chinch18	0.494	TS-15mg/kg
15N4	Chinch15	0.743	TS-15mg/kg
14N4	Chinch14	0.649	TS-15mg/kg
19N4	Chinch19	0.566	anti-tip-chimer
27N4	Chinch27	0.505	anti-tip-chimer
21N4	Chinch21	0.729	anti-tip-chimer
23N4	Chinch23	0.612	AC-5mg/kg
26N4	Chinch26	0.531	AC-5mg/kg
20N4	Chinch20	0.549	AC-5mg/kg
25N4	Chinch25	0.691	AC-10mg/kg
22N4	Chinch22	0.670	AC-10mg/kg
5N4	Chinch5	0.600	Ofloxacin
7N4	Chinch7	0.339	Ofloxacin
6N4	Chinch6	0.543	Saline
8N4	Chinch8	0.705	Saline

Supplementary File 2
Longitudinal Pairwise-Distances and Pairwise-Differences

NPL Jaccard Pairwise-Distance Mann-Whitney Pairwise Comparisons
Baseline - 2DPT

Group A	Group B	Mann-Whitney U	P-value	FDR P-value
AC-PO7d	AC-5mg/kg	6	0.700	0.993
AC-PO7d	AC-10mg/kg	4	0.800	0.993
AC-PO7d	Ofloxacin	7	0.400	0.993
AC-PO7d	Saline	7	0.400	0.993
AC-PO7d	TS-30mg/kg	6	0.700	0.993
AC-PO7d	TS-15mg/kg	6	0.700	0.993
AC-PO7d	anti-tip-chimer	7	0.400	0.993
AC-PO7d	anti-rsPilA	8	0.200	0.993
AC-5mg/kg	AC-10mg/kg	3	1.000	1.000
AC-5mg/kg	Ofloxacin	6	0.700	0.993
AC-5mg/kg	Saline	3	0.700	0.993
AC-10mg/kg	Ofloxacin	4	0.800	0.993
AC-10mg/kg	Saline	2	0.800	0.993
Ofloxacin	Saline	2	0.400	0.993
TS-30mg/kg	AC-5mg/kg	6	0.700	0.993
TS-30mg/kg	AC-10mg/kg	3	1.000	1.000
TS-30mg/kg	Ofloxacin	7	0.400	0.993
TS-30mg/kg	Saline	3	0.700	0.993
TS-30mg/kg	TS-15mg/kg	4	1.000	1.000
TS-30mg/kg	anti-tip-chimer	7	0.400	0.993
TS-30mg/kg	anti-rsPilA	8	0.200	0.993
TS-15mg/kg	AC-5mg/kg	6	0.700	0.993
TS-15mg/kg	AC-10mg/kg	4	0.800	0.993
TS-15mg/kg	Ofloxacin	6	0.700	0.993
TS-15mg/kg	Saline	5	1.000	1.000
TS-15mg/kg	anti-tip-chimer	6	0.700	0.993
anti-tip-chimer	AC-5mg/kg	3	0.700	0.993
anti-tip-chimer	AC-10mg/kg	2	0.800	0.993
anti-tip-chimer	Ofloxacin	4	1.000	1.000
anti-tip-chimer	Saline	2	0.400	0.993
anti-rsPilA	AC-5mg/kg	3	0.700	0.993
anti-rsPilA	AC-10mg/kg	1	0.400	0.993
anti-rsPilA	Ofloxacin	4	1.000	1.000
anti-rsPilA	Saline	1	0.200	0.993
anti-rsPilA	TS-15mg/kg	2	0.400	0.993
anti-rsPilA	anti-tip-chimer	4	1.000	1.000

Kruskal-Wallis (Omnibus Test)

**H**	5.775
**p-value**	0.672

Supplementary File 2
Longitudinal Pairwise-Distances and Pairwise-Differences

NPL Jaccard Pairwise-Distance Mann-Whitney Pairwise Comparisons
Baseline - 5DPT

Group A	Group B	Mann-Whitney U	P-value	FDR P-value
AC-PO7d	AC-5mg/kg	9	0.100	0.900
AC-PO7d	AC-10mg/kg	5	0.400	0.900
AC-PO7d	Ofloxacin	6	0.700	1.000
AC-PO7d	Saline	6	0.700	1.000
AC-PO7d	TS-30mg/kg	9	0.100	0.900
AC-PO7d	TS-15mg/kg	8	0.200	0.900
AC-PO7d	anti-tip-chimer	9	0.100	0.900
AC-PO7d	anti-rsPilA	6	0.200	0.900
AC-5mg/kg	AC-10mg/kg	0	0.200	0.900
AC-5mg/kg	Ofloxacin	5	1.000	1.000
AC-5mg/kg	Saline	2	0.400	0.900
AC-10mg/kg	Ofloxacin	4	0.800	1.000
AC-10mg/kg	Saline	3	1.000	1.000
Ofloxacin	Saline	3	0.700	1.000
TS-30mg/kg	AC-5mg/kg	7	0.400	0.900
TS-30mg/kg	AC-10mg/kg	1	0.400	0.900
TS-30mg/kg	Ofloxacin	5	1.000	1.000
TS-30mg/kg	Saline	3	0.700	1.000
TS-30mg/kg	TS-15mg/kg	4	1.000	1.000
TS-30mg/kg	anti-tip-chimer	7	0.400	0.900
TS-30mg/kg	anti-rsPilA	4	0.800	1.000
TS-15mg/kg	AC-5mg/kg	7	0.400	0.900
TS-15mg/kg	AC-10mg/kg	2	0.800	1.000
TS-15mg/kg	Ofloxacin	6	0.700	1.000
TS-15mg/kg	Saline	4	1.000	1.000
TS-15mg/kg	anti-tip-chimer	7	0.400	0.900
anti-tip-chimer	AC-5mg/kg	5	1.000	1.000
anti-tip-chimer	AC-10mg/kg	1	0.400	0.900
anti-tip-chimer	Ofloxacin	4	1.000	1.000
anti-tip-chimer	Saline	1	0.200	0.900
anti-rsPilA	AC-5mg/kg	4	0.800	1.000
anti-rsPilA	AC-10mg/kg	1	0.667	1.000
anti-rsPilA	Ofloxacin	3	1.000	1.000
anti-rsPilA	Saline	1	0.400	0.900
anti-rsPilA	TS-15mg/kg	2	0.800	1.000
anti-rsPilA	anti-tip-chimer	4	0.800	1.000

Kruskal-Wallis (Omnibus Test)

**H**	8.837
**p-value**	0.356

Supplementary File 2
Longitudinal Pairwise-Distances and Pairwise-Differences

NPL Jaccard Pairwise-Distance Mann-Whitney Pairwise Comparisons
Baseline - 7DPT

Group A	Group B	Mann-Whitney U	P-value	FDR P-value
AC-PO7d	AC-5mg/kg	6	0.200	0.900
AC-PO7d	AC-10mg/kg	2	0.667	0.960
AC-PO7d	Ofloxacin	6	0.200	0.900
AC-PO7d	Saline	4	0.800	0.960
AC-PO7d	TS-30mg/kg	4	0.333	0.900
AC-PO7d	TS-15mg/kg	6	0.200	0.900
AC-PO7d	anti-tip-chimer	4	0.333	0.900
AC-PO7d	anti-rsPilA	4	0.333	0.900
AC-5mg/kg	AC-10mg/kg	0	0.500	0.960
AC-5mg/kg	Ofloxacin	4	1.000	1.000
AC-5mg/kg	Saline	0	0.100	0.900
AC-10mg/kg	Ofloxacin	3	0.500	0.960
AC-10mg/kg	Saline	1	1.000	1.000
Ofloxacin	Saline	1	0.200	0.900
TS-30mg/kg	AC-5mg/kg	6	0.200	0.900
TS-30mg/kg	AC-10mg/kg	1	1.000	1.000
TS-30mg/kg	Ofloxacin	5	0.400	0.900
TS-30mg/kg	Saline	2	0.800	0.960
TS-30mg/kg	TS-15mg/kg	4	0.800	0.960
TS-30mg/kg	anti-tip-chimer	4	0.333	0.900
TS-30mg/kg	anti-rsPilA	3	0.667	0.960
TS-15mg/kg	AC-5mg/kg	8	0.200	0.900
TS-15mg/kg	AC-10mg/kg	1	1.000	1.000
TS-15mg/kg	Ofloxacin	6	0.700	0.960
TS-15mg/kg	Saline	1	0.200	0.900
TS-15mg/kg	anti-tip-chimer	4	0.800	0.960
anti-tip-chimer	AC-5mg/kg	4	0.800	0.960
anti-tip-chimer	AC-10mg/kg	0	0.667	0.960
anti-tip-chimer	Ofloxacin	3	1.000	1.000
anti-tip-chimer	Saline	1	0.400	0.900
anti-rsPilA	AC-5mg/kg	5	0.400	0.900
anti-rsPilA	AC-10mg/kg	0	0.667	0.960
anti-rsPilA	Ofloxacin	3	1.000	1.000
anti-rsPilA	Saline	1	0.400	0.900
anti-rsPilA	TS-15mg/kg	2	0.800	0.960
anti-rsPilA	anti-tip-chimer	3	0.667	0.960

Kruskal-Wallis (Omnibus Test)

**H**	12.074
**p-value**	0.148

Supplementary File 2
Longitudinal Pairwise-Distances and Pairwise-Differences

NPL Jaccard Pairwise-Distance Mann-Whitney Pairwise Comparisons
Baseline - 9DPT

Group A	Group B	Mann-Whitney U	P-value	FDR P-value
AC-PO7d	AC-5mg/kg	6	0.200	1.000
AC-PO7d	AC-10mg/kg	2	1.000	1.000
AC-PO7d	Ofloxacin	4	0.333	1.000
AC-PO7d	Saline	3	0.667	1.000
AC-PO7d	TS-30mg/kg	3	1.000	1.000
AC-PO7d	TS-15mg/kg	4	0.800	1.000
AC-PO7d	anti-tip-chimer	5	0.400	1.000
AC-PO7d	anti-rsPilA	3	0.667	1.000
AC-5mg/kg	AC-10mg/kg	0	0.200	1.000
AC-5mg/kg	Ofloxacin	4	0.800	1.000
AC-5mg/kg	Saline	2	0.800	1.000
AC-10mg/kg	Ofloxacin	4	0.333	1.000
AC-10mg/kg	Saline	2	1.000	1.000
Ofloxacin	Saline	1	0.667	1.000
TS-30mg/kg	AC-5mg/kg	9	0.100	1.000
TS-30mg/kg	AC-10mg/kg	3	1.000	1.000
TS-30mg/kg	Ofloxacin	6	0.200	1.000
TS-30mg/kg	Saline	4	0.800	1.000
TS-30mg/kg	TS-15mg/kg	5	1.000	1.000
TS-30mg/kg	anti-tip-chimer	7	0.400	1.000
TS-30mg/kg	anti-rsPilA	3	1.000	1.000
TS-15mg/kg	AC-5mg/kg	6	0.700	1.000
TS-15mg/kg	AC-10mg/kg	2	0.800	1.000
TS-15mg/kg	Ofloxacin	5	0.400	1.000
TS-15mg/kg	Saline	3	1.000	1.000
TS-15mg/kg	anti-tip-chimer	5	1.000	1.000
anti-tip-chimer	AC-5mg/kg	5	1.000	1.000
anti-tip-chimer	AC-10mg/kg	2	0.800	1.000
anti-tip-chimer	Ofloxacin	4	0.800	1.000
anti-tip-chimer	Saline	3	1.000	1.000
anti-rsPilA	AC-5mg/kg	3	1.000	1.000
anti-rsPilA	AC-10mg/kg	2	1.000	1.000
anti-rsPilA	Ofloxacin	3	0.667	1.000
anti-rsPilA	Saline	2	1.000	1.000
anti-rsPilA	TS-15mg/kg	4	0.800	1.000
anti-rsPilA	anti-tip-chimer	4	0.800	1.000

Kruskal-Wallis (Omnibus Test)

**H**	6.340
**p-value**	0.609

Supplementary File 2
Longitudinal Pairwise-Distances and Pairwise-Differences

NPL Unweighted UniFrac Pairwise-Distances
Baseline - 2DPT

#SampleID	SubjectID	Distance	Group
3AN2	Chinch3	0.574	AC-PO7d
1AN2	Chinch1	0.689	AC-PO7d
2AN2	Chinch2	0.328	AC-PO7d
16N1	Chinch16	0.476	TS-30mg/kg
10N1	Chinch10	0.492	TS-30mg/kg
12N1	Chinch12	0.350	TS-30mg/kg
13N1	Chinch13	0.278	anti-rsPilA
17N1	Chinch17	0.409	anti-rsPilA
11N1	Chinch11	0.335	anti-rsPilA
18N1	Chinch18	0.288	TS-15mg/kg
14N1	Chinch14	0.318	TS-15mg/kg
15N1	Chinch15	0.398	TS-15mg/kg
19N1	Chinch19	0.268	anti-tip-chimer
27N1	Chinch27	0.255	anti-tip-chimer
21N1	Chinch21	0.220	anti-tip-chimer
23N1	Chinch23	0.335	AC-5mg/kg
20N1	Chinch20	0.377	AC-5mg/kg
26N1	Chinch26	0.283	AC-5mg/kg
25N1	Chinch25	0.338	AC-10mg/kg
22N1	Chinch22	0.262	AC-10mg/kg
5N1	Chinch5	0.336	Ofloxacin
7N1	Chinch7	0.210	Ofloxacin
4N1	Chinch4	0.383	Ofloxacin
6N1	Chinch6	0.329	Saline
9N1	Chinch9	0.301	Saline
8N1	Chinch8	0.297	Saline

Supplementary File 2
Longitudinal Pairwise-Distances and Pairwise-Differences

NPL Unweighted UniFrac Pairwise-Distances
Baseline - 5DPT

#SampleID	SubjectID	Distance	Group
3AN5	Chinch3	0.422	AC-PO7d
1AN5	Chinch1	0.669	AC-PO7d
2AN5	Chinch2	0.428	AC-PO7d
16N2	Chinch16	0.430	TS-30mg/kg
10N2	Chinch10	0.371	TS-30mg/kg
12N2	Chinch12	0.340	TS-30mg/kg
11N2	Chinch11	0.294	anti-rsPilA
17N2	Chinch17	0.402	anti-rsPilA
15N2	Chinch15	0.351	TS-15mg/kg
14N2	Chinch14	0.487	TS-15mg/kg
18N2	Chinch18	0.299	TS-15mg/kg
27N2	Chinch27	0.380	anti-tip-chimer
19N2	Chinch19	0.275	anti-tip-chimer
21N2	Chinch21	0.298	anti-tip-chimer
20N2	Chinch20	0.412	AC-5mg/kg
23N2	Chinch23	0.288	AC-5mg/kg
26N2	Chinch26	0.313	AC-5mg/kg
25N2	Chinch25	0.347	AC-10mg/kg
22N2	Chinch22	0.550	AC-10mg/kg
7N2	Chinch7	0.379	Ofloxacin
5N2	Chinch5	0.576	Ofloxacin
4N2	Chinch4	0.337	Ofloxacin
9N2	Chinch9	0.770	Saline
8N2	Chinch8	0.302	Saline
6N2	Chinch6	0.410	Saline

Supplementary File 2
Longitudinal Pairwise-Distances and Pairwise-Differences

NPL Unweighted UniFrac Pairwise-Distances
Baseline - 7DPT

#SampleID	SubjectID	Distance	Group
1AN7	Chinch1	0.402	AC-PO7d
2AN7	Chinch2	0.595	AC-PO7d
10N3	Chinch10	0.316	TS-30mg/kg
16N3	Chinch16	0.357	TS-30mg/kg
11N3	Chinch11	0.411	anti-rsPilA
17N3	Chinch17	0.441	anti-rsPilA
18N3	Chinch18	0.238	TS-15mg/kg
14N3	Chinch14	0.484	TS-15mg/kg
15N3	Chinch15	0.313	TS-15mg/kg
27N3	Chinch27	0.276	anti-tip-chimer
21N3	Chinch21	0.200	anti-tip-chimer
23N3	Chinch23	0.342	AC-5mg/kg
20N3	Chinch20	0.364	AC-5mg/kg
26N3	Chinch26	0.426	AC-5mg/kg
25N3	Chinch25	0.342	AC-10mg/kg
5N3	Chinch5	0.349	Ofloxacin
4N3	Chinch4	0.272	Ofloxacin
7N3	Chinch7	0.203	Ofloxacin
9N3	Chinch9	0.710	Saline
8N3	Chinch8	0.237	Saline
6N3	Chinch6	0.470	Saline

Supplementary File 2
Longitudinal Pairwise-Distances and Pairwise-Differences

NPL Unweighted UniFrac Pairwise-Distances
Baseline - 9DPT

#SampleID	SubjectID	Distance	Group
2AN9	Chinch2	0.355	AC-PO7d
3AN9	Chinch3	0.491	AC-PO7d
16N4	Chinch16	0.358	TS-30mg/kg
10N4	Chinch10	0.470	TS-30mg/kg
12N4	Chinch12	0.345	TS-30mg/kg
17N4	Chinch17	0.430	anti-rsPilA
11N4	Chinch11	0.269	anti-rsPilA
18N4	Chinch18	0.352	TS-15mg/kg
15N4	Chinch15	0.396	TS-15mg/kg
14N4	Chinch14	0.352	TS-15mg/kg
27N4	Chinch27	0.246	anti-tip-chimer
21N4	Chinch21	0.367	anti-tip-chimer
19N4	Chinch19	0.289	anti-tip-chimer
26N4	Chinch26	0.270	AC-5mg/kg
23N4	Chinch23	0.274	AC-5mg/kg
20N4	Chinch20	0.403	AC-5mg/kg
22N4	Chinch22	0.424	AC-10mg/kg
25N4	Chinch25	0.396	AC-10mg/kg
5N4	Chinch5	0.171	Ofloxacin
7N4	Chinch7	0.096	Ofloxacin
8N4	Chinch8	0.282	Saline
6N4	Chinch6	0.292	Saline

Supplementary File 2
Longitudinal Pairwise-Distances and Pairwise-Differences

NPL Unweighted UniFrac Pairwise-Distance Mann-Whitney Pairwise Comparisons
Baseline - 2DPT

Group A	Group B	Mann-Whitney U	P-value	FDR P-value
AC-PO7d	AC-5mg/kg	7	0.400	0.758
AC-PO7d	AC-10mg/kg	5	0.400	0.758
AC-PO7d	Ofloxacin	7	0.400	0.758
AC-PO7d	Saline	8	0.200	0.514
AC-PO7d	TS-30mg/kg	6	0.700	1.000
AC-PO7d	TS-15mg/kg	8	0.200	0.514
AC-PO7d	anti-tip-chimer	9	0.100	0.514
AC-PO7d	anti-rsPilA	7	0.400	0.758
AC-5mg/kg	AC-10mg/kg	4	0.800	1.000
AC-5mg/kg	Ofloxacin	4	1.000	1.000
AC-5mg/kg	Saline	6	0.700	1.000
AC-10mg/kg	Ofloxacin	3	1.000	1.000
AC-10mg/kg	Saline	3	1.000	1.000
Ofloxacin	Saline	6	0.700	1.000
TS-30mg/kg	AC-5mg/kg	8	0.200	0.514
TS-30mg/kg	AC-10mg/kg	6	0.200	0.514
TS-30mg/kg	Ofloxacin	8	0.200	0.514
TS-30mg/kg	Saline	9	0.100	0.514
TS-30mg/kg	TS-15mg/kg	8	0.200	0.514
TS-30mg/kg	anti-tip-chimer	9	0.100	0.514
TS-30mg/kg	anti-rsPilA	8	0.200	0.514
TS-15mg/kg	AC-5mg/kg	5	1.000	1.000
TS-15mg/kg	AC-10mg/kg	4	0.800	1.000
TS-15mg/kg	Ofloxacin	5	1.000	1.000
TS-15mg/kg	Saline	5	1.000	1.000
TS-15mg/kg	anti-tip-chimer	9	0.100	0.514
anti-tip-chimer	AC-5mg/kg	0	0.100	0.514
anti-tip-chimer	AC-10mg/kg	1	0.400	0.758
anti-tip-chimer	Ofloxacin	3	0.700	1.000
anti-tip-chimer	Saline	0	0.100	0.514
anti-rsPilA	AC-5mg/kg	4	1.000	1.000
anti-rsPilA	AC-10mg/kg	4	0.800	1.000
anti-rsPilA	Ofloxacin	5	1.000	1.000
anti-rsPilA	Saline	6	0.700	1.000
anti-rsPilA	TS-15mg/kg	5	1.000	1.000
anti-rsPilA	anti-tip-chimer	9	0.100	0.514

Kruskal-Wallis (Omnibus Test)

**H**	12.410
**p-value**	0.134

Supplementary File 2
Longitudinal Pairwise-Distances and Pairwise-Differences

NPL Unweighted UniFrac Pairwise-Distance Mann-Whitney Pairwise Comparisons
Baseline - 5DPT

Group A	Group B	Mann-Whitney U	P-value	FDR P-value
AC-PO7d	AC-5mg/kg	9	0.100	0.960
AC-PO7d	AC-10mg/kg	4	0.800	0.993
AC-PO7d	Ofloxacin	7	0.400	0.960
AC-PO7d	Saline	6	0.700	0.993
AC-PO7d	TS-30mg/kg	7	0.400	0.960
AC-PO7d	TS-15mg/kg	7	0.400	0.960
AC-PO7d	anti-tip-chimer	9	0.100	0.960
AC-PO7d	anti-rsPilA	6	0.200	0.960
AC-5mg/kg	AC-10mg/kg	1	0.400	0.960
AC-5mg/kg	Ofloxacin	2	0.400	0.960
AC-5mg/kg	Saline	3	0.700	0.993
AC-10mg/kg	Ofloxacin	3	1.000	1.000
AC-10mg/kg	Saline	3	1.000	1.000
Ofloxacin	Saline	4	1.000	1.000
TS-30mg/kg	AC-5mg/kg	7	0.400	0.960
TS-30mg/kg	AC-10mg/kg	2	0.800	0.993
TS-30mg/kg	Ofloxacin	4	1.000	1.000
TS-30mg/kg	Saline	4	1.000	1.000
TS-30mg/kg	TS-15mg/kg	5	1.000	1.000
TS-30mg/kg	anti-tip-chimer	7	0.400	0.960
TS-30mg/kg	anti-rsPilA	4	0.800	0.993
TS-15mg/kg	AC-5mg/kg	6	0.700	0.993
TS-15mg/kg	AC-10mg/kg	2	0.800	0.993
TS-15mg/kg	Ofloxacin	3	0.700	0.993
TS-15mg/kg	Saline	3	0.700	0.993
TS-15mg/kg	anti-tip-chimer	7	0.400	0.960
anti-tip-chimer	AC-5mg/kg	3	0.700	0.993
anti-tip-chimer	AC-10mg/kg	1	0.400	0.960
anti-tip-chimer	Ofloxacin	2	0.400	0.960
anti-tip-chimer	Saline	1	0.200	0.960
anti-rsPilA	AC-5mg/kg	3	1.000	1.000
anti-rsPilA	AC-10mg/kg	1	0.667	0.993
anti-rsPilA	Ofloxacin	2	0.800	0.993
anti-rsPilA	Saline	1	0.400	0.960
anti-rsPilA	TS-15mg/kg	2	0.800	0.993
anti-rsPilA	anti-tip-chimer	4	0.800	0.993

Kruskal-Wallis (Omnibus Test)

**H**	0.800
**p-value**	0.433

Supplementary File 2
Longitudinal Pairwise-Distances and Pairwise-Differences

NPL Unweighted UniFrac Pairwise-Distance Mann-Whitney Pairwise Comparisons
Baseline - 7DPT

Group A	Group B	Mann-Whitney U	P-value	FDR P-value
AC-PO7d	AC-5mg/kg	5	0.400	0.847
AC-PO7d	AC-10mg/kg	2	0.667	0.993
AC-PO7d	Ofloxacin	6	0.200	0.847
AC-PO7d	Saline	3	1.000	1.000
AC-PO7d	TS-30mg/kg	4	0.333	0.847
AC-PO7d	TS-15mg/kg	5	0.400	0.847
AC-PO7d	anti-tip-chimer	4	0.333	0.847
AC-PO7d	anti-rsPilA	2	1.000	1.000
AC-5mg/kg	AC-10mg/kg	3	0.500	0.993
AC-5mg/kg	Ofloxacin	8	0.200	0.847
AC-5mg/kg	Saline	3	0.700	0.993
AC-10mg/kg	Ofloxacin	2	1.000	1.000
AC-10mg/kg	Saline	1	1.000	1.000
Ofloxacin	Saline	2	0.400	0.847
TS-30mg/kg	AC-5mg/kg	1	0.400	0.847
TS-30mg/kg	AC-10mg/kg	1	1.000	1.000
TS-30mg/kg	Ofloxacin	5	0.400	0.847
TS-30mg/kg	Saline	2	0.800	0.993
TS-30mg/kg	TS-15mg/kg	4	0.800	0.993
TS-30mg/kg	anti-tip-chimer	4	0.333	0.847
TS-30mg/kg	anti-rsPilA	0	0.333	0.847
TS-15mg/kg	AC-5mg/kg	3	0.700	0.993
TS-15mg/kg	AC-10mg/kg	1	1.000	1.000
TS-15mg/kg	Ofloxacin	6	0.700	0.993
TS-15mg/kg	Saline	4	1.000	1.000
TS-15mg/kg	anti-tip-chimer	5	0.400	0.847
anti-tip-chimer	AC-5mg/kg	0	0.200	0.847
anti-tip-chimer	AC-10mg/kg	0	0.667	0.993
anti-tip-chimer	Ofloxacin	2	0.800	0.993
anti-tip-chimer	Saline	1	0.400	0.847
anti-rsPilA	AC-5mg/kg	5	0.400	0.847
anti-rsPilA	AC-10mg/kg	2	0.667	0.993
anti-rsPilA	Ofloxacin	6	0.200	0.847
anti-rsPilA	Saline	2	0.800	0.993
anti-rsPilA	TS-15mg/kg	4	0.800	0.993
anti-rsPilA	anti-tip-chimer	4	0.333	0.847

Kruskal-Wallis (Omnibus Test)

**H**	9.286
**p-value**	0.319

Supplementary File 2
Longitudinal Pairwise-Distances and Pairwise-Differences

NPL Unweighted UniFrac Pairwise-Distance Mann-Whitney Pairwise Comparisons
Baseline - 9DPT

Group A	Group B	Mann-Whitney U	P-value	FDR P-value
AC-PO7d	AC-5mg/kg	5	0.400	0.686
AC-PO7d	AC-10mg/kg	2	1.000	1.000
AC-PO7d	Ofloxacin	4	0.333	0.686
AC-PO7d	Saline	4	0.333	0.686
AC-PO7d	TS-30mg/kg	4	0.800	1.000
AC-PO7d	TS-15mg/kg	5	0.400	0.686
AC-PO7d	anti-tip-chimer	5	0.400	0.686
AC-PO7d	anti-rsPilA	3	0.667	1.000
AC-5mg/kg	AC-10mg/kg	1	0.400	0.686
AC-5mg/kg	Ofloxacin	6	0.200	0.686
AC-5mg/kg	Saline	2	0.800	1.000
AC-10mg/kg	Ofloxacin	4	0.333	0.686
AC-10mg/kg	Saline	4	0.333	0.686
Ofloxacin	Saline	0	0.333	0.686
TS-30mg/kg	AC-5mg/kg	7	0.400	0.686
TS-30mg/kg	AC-10mg/kg	2	0.800	1.000
TS-30mg/kg	Ofloxacin	6	0.200	0.686
TS-30mg/kg	Saline	6	0.200	0.686
TS-30mg/kg	TS-15mg/kg	5	1.000	1.000
TS-30mg/kg	anti-tip-chimer	7	0.400	0.686
TS-30mg/kg	anti-rsPilA	4	0.800	1.000
TS-15mg/kg	AC-5mg/kg	6	0.700	1.000
TS-15mg/kg	AC-10mg/kg	0	0.200	0.686
TS-15mg/kg	Ofloxacin	6	0.200	0.686
TS-15mg/kg	Saline	6	0.200	0.686
TS-15mg/kg	anti-tip-chimer	7	0.400	0.686
anti-tip-chimer	AC-5mg/kg	4	1.000	1.000
anti-tip-chimer	AC-10mg/kg	0	0.200	0.686
anti-tip-chimer	Ofloxacin	6	0.200	0.686
anti-tip-chimer	Saline	3	1.000	1.000
anti-rsPilA	AC-5mg/kg	3	1.000	1.000
anti-rsPilA	AC-10mg/kg	2	1.000	1.000
anti-rsPilA	Ofloxacin	4	0.333	0.686
anti-rsPilA	Saline	2	1.000	1.000
anti-rsPilA	TS-15mg/kg	3	1.000	1.000
anti-rsPilA	anti-tip-chimer	4	0.800	1.000

Kruskal-Wallis (Omnibus Test)

**H**	10.889
**p-value**	0.208

Supplementary File 2
Longitudinal Pairwise-Distances and Pairwise-Differences

NPL Evenness Pairwise-Differences

Baseline - 2DPT

#SampleID	SubjectID	Difference	Group
2AN2	Chinch2	-0.058	AC-PO7d
3AN2	Chinch3	0.041	AC-PO7d
1AN2	Chinch1	0.085	AC-PO7d
5N1	Chinch5	-0.036	Ofloxacin
7N1	Chinch7	-0.018	Ofloxacin
4N1	Chinch4	-0.016	Ofloxacin
8N1	Chinch8	-0.016	Saline
6N1	Chinch6	0.032	Saline
9N1	Chinch9	-0.002	Saline
10N1	Chinch10	-0.014	TS-30mg/kg
12N1	Chinch12	-0.018	TS-30mg/kg
16N1	Chinch16	0.018	TS-30mg/kg
13N1	Chinch13	-0.027	anti-rsPilA
17N1	Chinch17	0.009	anti-rsPilA
11N1	Chinch11	-0.004	anti-rsPilA
15N1	Chinch15	0.012	TS-15mg/kg
18N1	Chinch18	-0.010	TS-15mg/kg
14N1	Chinch14	0.025	TS-15mg/kg
19N1	Chinch19	0.000	anti-tip-chimer
27N1	Chinch27	-0.002	anti-tip-chimer
21N1	Chinch21	0.004	anti-tip-chimer
26N1	Chinch26	-0.027	AC-5mg/kg
23N1	Chinch23	-0.019	AC-5mg/kg
20N1	Chinch20	-0.014	AC-5mg/kg
25N1	Chinch25	-0.014	AC-10mg/kg
22N1	Chinch22	0.017	AC-10mg/kg

Supplementary File 2
Longitudinal Pairwise-Distances and Pairwise-Differences

NPL Evenness Pairwise-Differences

Baseline - 5DPT

#SampleID	SubjectID	Difference	Group
2AN5	Chinch2	0.027	AC-PO7d
1AN5	Chinch1	0.098	AC-PO7d
3AN5	Chinch3	-0.074	AC-PO7d
7N2	Chinch7	-0.007	Ofloxacin
4N2	Chinch4	-0.002	Ofloxacin
5N2	Chinch5	-0.004	Ofloxacin
8N2	Chinch8	-0.006	Saline
9N2	Chinch9	-0.032	Saline
6N2	Chinch6	0.025	Saline
12N2	Chinch12	-0.020	TS-30mg/kg
16N2	Chinch16	0.029	TS-30mg/kg
10N2	Chinch10	0.002	TS-30mg/kg
11N2	Chinch11	-0.021	anti-rsPilA
17N2	Chinch17	0.025	anti-rsPilA
15N2	Chinch15	0.006	TS-15mg/kg
14N2	Chinch14	0.014	TS-15mg/kg
18N2	Chinch18	-0.015	TS-15mg/kg
27N2	Chinch27	-0.018	anti-tip-chimer
19N2	Chinch19	0.008	anti-tip-chimer
21N2	Chinch21	0.010	anti-tip-chimer
26N2	Chinch26	0.008	AC-5mg/kg
23N2	Chinch23	-0.002	AC-5mg/kg
20N2	Chinch20	-0.036	AC-5mg/kg
25N2	Chinch25	-0.038	AC-10mg/kg
22N2	Chinch22	0.016	AC-10mg/kg

Supplementary File 2
Longitudinal Pairwise-Distances and Pairwise-Differences

NPL Evenness Pairwise-Differences

Baseline - 7DPT

#SampleID	SubjectID	Difference	Group
1AN7	Chinch1	-0.023	AC-PO7d
2AN7	Chinch2	-0.224	AC-PO7d
5N3	Chinch5	-0.087	Ofloxacin
4N3	Chinch4	-0.005	Ofloxacin
7N3	Chinch7	-0.005	Ofloxacin
9N3	Chinch9	-0.122	Saline
6N3	Chinch6	0.012	Saline
8N3	Chinch8	0.003	Saline
16N3	Chinch16	0.049	TS-30mg/kg
10N3	Chinch10	0.001	TS-30mg/kg
17N3	Chinch17	0.028	anti-rsPilA
11N3	Chinch11	-0.047	anti-rsPilA
18N3	Chinch18	-0.028	TS-15mg/kg
15N3	Chinch15	0.010	TS-15mg/kg
14N3	Chinch14	0.008	TS-15mg/kg
21N3	Chinch21	-0.008	anti-tip-chimer
27N3	Chinch27	-0.015	anti-tip-chimer
26N3	Chinch26	-0.015	AC-5mg/kg
23N3	Chinch23	-0.009	AC-5mg/kg
20N3	Chinch20	-0.011	AC-5mg/kg
25N3	Chinch25	-0.025	AC-10mg/kg

Supplementary File 2
Longitudinal Pairwise-Distances and Pairwise-Differences

NPL Evenness Pairwise-Differences

Baseline - 9DPT

#SampleID	SubjectID	Difference	Group
3AN9	Chinch3	-0.040	AC-PO7d
2AN9	Chinch2	-0.154	AC-PO7d
5N4	Chinch5	-0.120	Ofloxacin
7N4	Chinch7	0.001	Ofloxacin
8N4	Chinch8	-0.017	Saline
6N4	Chinch6	0.026	Saline
12N4	Chinch12	-0.019	TS-30mg/kg
10N4	Chinch10	-0.020	TS-30mg/kg
16N4	Chinch16	0.046	TS-30mg/kg
11N4	Chinch11	-0.037	anti-rsPilA
17N4	Chinch17	0.012	anti-rsPilA
15N4	Chinch15	0.001	TS-15mg/kg
14N4	Chinch14	0.002	TS-15mg/kg
18N4	Chinch18	-0.013	TS-15mg/kg
19N4	Chinch19	0.003	anti-tip-chimer
27N4	Chinch27	-0.011	anti-tip-chimer
21N4	Chinch21	0.020	anti-tip-chimer
23N4	Chinch23	-0.018	AC-5mg/kg
20N4	Chinch20	-0.051	AC-5mg/kg
26N4	Chinch26	-0.038	AC-5mg/kg
22N4	Chinch22	-0.078	AC-10mg/kg
25N4	Chinch25	-0.025	AC-10mg/kg

Supplementary File 2
Longitudinal Pairwise-Distances and Pairwise-Differences

NPL Evenness Pairwise-Difference Mann-Whitney Pairwise Comparisons
Baseline - 2DPT

Group A	Group B	Mann-Whitney U	P-value	FDR P-value
AC-PO7d	AC-5mg/kg	6	0.700	0.969
AC-PO7d	AC-10mg/kg	4	0.800	0.993
AC-PO7d	Ofloxacin	6	0.700	0.969
AC-PO7d	Saline	6	0.700	0.969
AC-PO7d	TS-30mg/kg	6	0.700	0.969
AC-PO7d	TS-15mg/kg	6	0.700	0.969
AC-PO7d	anti-tip-chimer	6	0.700	0.969
AC-PO7d	anti-rsPilA	6	0.700	0.969
AC-5mg/kg	AC-10mg/kg	0	0.200	0.720
Ofloxacin	AC-5mg/kg	4	1.000	1.000
Ofloxacin	AC-10mg/kg	0	0.200	0.720
Ofloxacin	Saline	0	0.100	0.720
Ofloxacin	TS-30mg/kg	1	0.200	0.720
Ofloxacin	TS-15mg/kg	0	0.100	0.720
Ofloxacin	anti-tip-chimer	0	0.100	0.720
Ofloxacin	anti-rsPilA	2	0.400	0.969
Saline	AC-5mg/kg	8	0.200	0.720
Saline	AC-10mg/kg	3	1.000	1.000
Saline	TS-30mg/kg	6	0.700	0.969
Saline	TS-15mg/kg	4	1.000	1.000
Saline	anti-tip-chimer	4	1.000	1.000
Saline	anti-rsPilA	6	0.700	0.969
TS-30mg/kg	AC-5mg/kg	8	0.200	0.720
TS-30mg/kg	AC-10mg/kg	3	1.000	1.000
TS-30mg/kg	TS-15mg/kg	2	0.400	0.969
TS-30mg/kg	anti-tip-chimer	3	0.700	0.969
TS-30mg/kg	anti-rsPilA	5	1.000	1.000
TS-15mg/kg	AC-5mg/kg	9	0.100	0.720
TS-15mg/kg	AC-10mg/kg	4	0.800	0.993
TS-15mg/kg	anti-tip-chimer	6	0.700	0.969
anti-tip-chimer	AC-5mg/kg	9	0.100	0.720
anti-tip-chimer	AC-10mg/kg	3	1.000	1.000
anti-rsPilA	AC-5mg/kg	7	0.400	0.969
anti-rsPilA	AC-10mg/kg	2	0.800	0.993
anti-rsPilA	TS-15mg/kg	2	0.400	0.969
anti-rsPilA	anti-tip-chimer	3	0.700	0.969

Kruskal-Wallis (Omnibus Test)

**H**	9.638
**p-value**	0.291

Supplementary File 2
Longitudinal Pairwise-Distances and Pairwise-Differences

NPL Evenness Pairwise-Difference Mann-Whitney Pairwise Comparisons
Baseline - 5DPT

Group A	Group B	Mann-Whitney U	P-value	FDR P-value
AC-PO7d	AC-5mg/kg	6	0.700	1.000
AC-PO7d	AC-10mg/kg	4	0.800	1.000
AC-PO7d	Ofloxacin	6	0.700	1.000
AC-PO7d	Saline	6	0.700	1.000
AC-PO7d	TS-30mg/kg	5	1.000	1.000
AC-PO7d	TS-15mg/kg	6	0.700	1.000
AC-PO7d	anti-tip-chimer	6	0.700	1.000
AC-PO7d	anti-rsPilA	4	0.800	1.000
AC-5mg/kg	AC-10mg/kg	3	1.000	1.000
Ofloxacin	AC-5mg/kg	3	0.700	1.000
Ofloxacin	AC-10mg/kg	3	1.000	1.000
Ofloxacin	Saline	5	1.000	1.000
Ofloxacin	TS-30mg/kg	3	0.700	1.000
Ofloxacin	TS-15mg/kg	3	0.700	1.000
Ofloxacin	anti-tip-chimer	3	0.700	1.000
Ofloxacin	anti-rsPilA	3	1.000	1.000
Saline	AC-5mg/kg	5	1.000	1.000
Saline	AC-10mg/kg	4	0.800	1.000
Saline	TS-30mg/kg	3	0.700	1.000
Saline	TS-15mg/kg	4	1.000	1.000
Saline	anti-tip-chimer	4	1.000	1.000
Saline	anti-rsPilA	2	0.800	1.000
TS-30mg/kg	AC-5mg/kg	6	0.700	1.000
TS-30mg/kg	AC-10mg/kg	4	0.800	1.000
TS-30mg/kg	TS-15mg/kg	4	1.000	1.000
TS-30mg/kg	anti-tip-chimer	4	1.000	1.000
TS-30mg/kg	anti-rsPilA	4	0.800	1.000
TS-15mg/kg	AC-5mg/kg	6	0.700	1.000
TS-15mg/kg	AC-10mg/kg	3	1.000	1.000
TS-15mg/kg	anti-tip-chimer	5	1.000	1.000
anti-tip-chimer	AC-5mg/kg	6	0.700	1.000
anti-tip-chimer	AC-10mg/kg	3	1.000	1.000
anti-rsPilA	AC-5mg/kg	4	0.800	1.000
anti-rsPilA	AC-10mg/kg	3	0.667	1.000
anti-rsPilA	TS-15mg/kg	3	1.000	1.000
anti-rsPilA	anti-tip-chimer	3	1.000	1.000

Kruskal-Wallis (Omnibus Test)

**H**	1.628
**p-value**	0.990

Supplementary File 2
Longitudinal Pairwise-Distances and Pairwise-Differences

NPL Evenness Pairwise-Difference Mann-Whitney Pairwise Comparisons
Baseline - 7DPT

Group A	Group B	Mann-Whitney U	P-value	FDR P-value
AC-PO7d	AC-5mg/kg	0	0.200	1.000
AC-PO7d	AC-10mg/kg	1	1.000	1.000
AC-PO7d	Ofloxacin	1	0.400	1.000
AC-PO7d	Saline	1	0.400	1.000
AC-PO7d	TS-30mg/kg	0	0.333	1.000
AC-PO7d	TS-15mg/kg	1	0.400	1.000
AC-PO7d	anti-tip-chimer	0	0.333	1.000
AC-PO7d	anti-rsPilA	1	0.667	1.000
AC-5mg/kg	AC-10mg/kg	3	0.500	1.000
Ofloxacin	AC-5mg/kg	6	0.700	1.000
Ofloxacin	AC-10mg/kg	2	1.000	1.000
Ofloxacin	Saline	3	0.700	1.000
Ofloxacin	TS-30mg/kg	0	0.200	1.000
Ofloxacin	TS-15mg/kg	2	0.400	1.000
Ofloxacin	anti-tip-chimer	4	0.800	1.000
Ofloxacin	anti-rsPilA	2	0.800	1.000
Saline	AC-5mg/kg	6	0.700	1.000
Saline	AC-10mg/kg	2	1.000	1.000
Saline	TS-30mg/kg	2	0.800	1.000
Saline	TS-15mg/kg	4	1.000	1.000
Saline	anti-tip-chimer	4	0.800	1.000
Saline	anti-rsPilA	2	0.800	1.000
TS-30mg/kg	AC-5mg/kg	6	0.200	1.000
TS-30mg/kg	AC-10mg/kg	2	0.667	1.000
TS-30mg/kg	TS-15mg/kg	4	0.800	1.000
TS-30mg/kg	anti-tip-chimer	4	0.333	1.000
TS-30mg/kg	anti-rsPilA	3	0.667	1.000
TS-15mg/kg	AC-5mg/kg	6	0.700	1.000
TS-15mg/kg	AC-10mg/kg	2	1.000	1.000
TS-15mg/kg	anti-tip-chimer	4	0.800	1.000
anti-tip-chimer	AC-5mg/kg	4	0.800	1.000
anti-tip-chimer	AC-10mg/kg	2	0.667	1.000
anti-rsPilA	AC-5mg/kg	3	1.000	1.000
anti-rsPilA	AC-10mg/kg	1	1.000	1.000
anti-rsPilA	TS-15mg/kg	3	1.000	1.000
anti-rsPilA	anti-tip-chimer	2	1.000	1.000

Kruskal-Wallis (Omnibus Test)

**H**	6.584
**p-value**	0.582

Supplementary File 2
Longitudinal Pairwise-Distances and Pairwise-Differences

NPL Evenness Pairwise-Difference Mann-Whitney Pairwise Comparisons
Baseline - 9DPT

Group A	Group B	Mann-Whitney U	P-value	FDR P-value
AC-PO7d	AC-5mg/kg	1	0.400	0.800
AC-PO7d	AC-10mg/kg	1	0.667	0.929
AC-PO7d	Ofloxacin	1	0.667	0.929
AC-PO7d	Saline	0	0.333	0.800
AC-PO7d	TS-30mg/kg	0	0.200	0.800
AC-PO7d	TS-15mg/kg	0	0.200	0.800
AC-PO7d	anti-tip-chimer	0	0.200	0.800
AC-PO7d	anti-rsPilA	0	0.333	0.800
AC-5mg/kg	AC-10mg/kg	4	0.800	0.929
Ofloxacin	AC-5mg/kg	3	1.000	1.000
Ofloxacin	AC-10mg/kg	2	1.000	1.000
Ofloxacin	Saline	1	0.667	0.929
Ofloxacin	TS-30mg/kg	2	0.800	0.929
Ofloxacin	TS-15mg/kg	1	0.400	0.800
Ofloxacin	anti-tip-chimer	1	0.400	0.800
Ofloxacin	anti-rsPilA	1	0.667	0.929
Saline	AC-5mg/kg	6	0.200	0.800
Saline	AC-10mg/kg	4	0.333	0.800
Saline	TS-30mg/kg	4	0.800	0.929
Saline	TS-15mg/kg	3	1.000	1.000
Saline	anti-tip-chimer	3	1.000	1.000
Saline	anti-rsPilA	3	0.667	0.929
TS-30mg/kg	AC-5mg/kg	7	0.400	0.800
TS-30mg/kg	AC-10mg/kg	6	0.200	0.800
TS-30mg/kg	TS-15mg/kg	3	0.700	0.929
TS-30mg/kg	anti-tip-chimer	3	0.700	0.929
TS-30mg/kg	anti-rsPilA	4	0.800	0.929
TS-15mg/kg	AC-5mg/kg	9	0.100	0.800
TS-15mg/kg	AC-10mg/kg	6	0.200	0.800
TS-15mg/kg	anti-tip-chimer	2	0.400	0.800
anti-tip-chimer	AC-5mg/kg	9	0.100	0.800
anti-tip-chimer	AC-10mg/kg	6	0.200	0.800
anti-rsPilA	AC-5mg/kg	5	0.400	0.800
anti-rsPilA	AC-10mg/kg	3	0.667	0.929
anti-rsPilA	TS-15mg/kg	3	1.000	1.000
anti-rsPilA	anti-tip-chimer	2	0.800	0.929

Kruskal-Wallis (Omnibus Test)

**H**	12.095
**p-value**	0.147

Supplementary File 2
Longitudinal Pairwise-Distances and Pairwise-Differences

NPL FaithPD Pairwise-Differences

Baseline - 2DPT

#SampleID	SubjectID	Difference	Group
1AN2	Chinch1	-5.545	AC-PO7d
2AN2	Chinch2	-1.652	AC-PO7d
3AN2	Chinch3	-2.147	AC-PO7d
4N1	Chinch4	-0.162	Ofloxacin
7N1	Chinch7	-0.416	Ofloxacin
5N1	Chinch5	-0.259	Ofloxacin
6N1	Chinch6	-0.268	Saline
9N1	Chinch9	0.279	Saline
8N1	Chinch8	0.267	Saline
16N1	Chinch16	1.291	TS-30mg/kg
10N1	Chinch10	-2.292	TS-30mg/kg
12N1	Chinch12	-0.067	TS-30mg/kg
17N1	Chinch17	-1.526	anti-rsPilA
11N1	Chinch11	-0.005	anti-rsPilA
13N1	Chinch13	0.094	anti-rsPilA
14N1	Chinch14	-0.389	TS-15mg/kg
15N1	Chinch15	0.676	TS-15mg/kg
18N1	Chinch18	-1.425	TS-15mg/kg
19N1	Chinch19	0.783	anti-tip-chimer
21N1	Chinch21	-0.532	anti-tip-chimer
27N1	Chinch27	-0.660	anti-tip-chimer
20N1	Chinch20	0.858	AC-5mg/kg
23N1	Chinch23	-0.201	AC-5mg/kg
26N1	Chinch26	0.683	AC-5mg/kg
25N1	Chinch25	-0.431	AC-10mg/kg
22N1	Chinch22	0.659	AC-10mg/kg

Supplementary File 2
Longitudinal Pairwise-Distances and Pairwise-Differences

NPL FaithPD Pairwise-Differences

Baseline - 5DPT

#SampleID	SubjectID	Difference	Group
1AN5	Chinch1	-5.099	AC-PO7d
3AN5	Chinch3	0.294	AC-PO7d
2AN5	Chinch2	-1.157	AC-PO7d
4N2	Chinch4	0.640	Ofloxacin
7N2	Chinch7	1.350	Ofloxacin
5N2	Chinch5	-0.750	Ofloxacin
8N2	Chinch8	-0.698	Saline
9N2	Chinch9	-4.245	Saline
6N2	Chinch6	0.118	Saline
16N2	Chinch16	0.836	TS-30mg/kg
12N2	Chinch12	-1.439	TS-30mg/kg
10N2	Chinch10	-1.630	TS-30mg/kg
17N2	Chinch17	-1.926	anti-rsPilA
11N2	Chinch11	0.368	anti-rsPilA
18N2	Chinch18	-0.462	TS-15mg/kg
15N2	Chinch15	1.121	TS-15mg/kg
14N2	Chinch14	-2.179	TS-15mg/kg
19N2	Chinch19	-0.751	anti-tip-chimer
21N2	Chinch21	-0.169	anti-tip-chimer
27N2	Chinch27	-0.086	anti-tip-chimer
23N2	Chinch23	-0.901	AC-5mg/kg
26N2	Chinch26	0.113	AC-5mg/kg
20N2	Chinch20	-0.952	AC-5mg/kg
22N2	Chinch22	-0.353	AC-10mg/kg
25N2	Chinch25	-0.057	AC-10mg/kg

Supplementary File 2
Longitudinal Pairwise-Distances and Pairwise-Differences

NPL FaithPD Pairwise-Differences

Baseline - 7DPT

#SampleID	SubjectID	Difference	Group
1AN7	Chinch1	-2.273	AC-PO7d
2AN7	Chinch2	-2.143	AC-PO7d
5N3	Chinch5	0.177	Ofloxacin
7N3	Chinch7	0.175	Ofloxacin
4N3	Chinch4	-0.839	Ofloxacin
6N3	Chinch6	1.428	Saline
8N3	Chinch8	-0.469	Saline
9N3	Chinch9	-3.884	Saline
16N3	Chinch16	0.822	TS-30mg/kg
10N3	Chinch10	-1.071	TS-30mg/kg
17N3	Chinch17	-0.890	anti-rsPilA
11N3	Chinch11	0.521	anti-rsPilA
15N3	Chinch15	0.973	TS-15mg/kg
14N3	Chinch14	-1.897	TS-15mg/kg
18N3	Chinch18	-0.412	TS-15mg/kg
21N3	Chinch21	0.380	anti-tip-chimer
27N3	Chinch27	-0.117	anti-tip-chimer
26N3	Chinch26	-1.298	AC-5mg/kg
20N3	Chinch20	-0.993	AC-5mg/kg
23N3	Chinch23	-1.061	AC-5mg/kg
25N3	Chinch25	-0.832	AC-10mg/kg

Supplementary File 2
Longitudinal Pairwise-Distances and Pairwise-Differences

NPL FaithPD Pairwise-Differences

Baseline - 9DPT

#SampleID	SubjectID	Difference	Group
3AN9	Chinch3	-0.673	AC-PO7d
2AN9	Chinch2	-0.197	AC-PO7d
5N4	Chinch5	-0.539	Ofloxacin
7N4	Chinch7	0.065	Ofloxacin
6N4	Chinch6	0.493	Saline
8N4	Chinch8	-0.458	Saline
16N4	Chinch16	1.342	TS-30mg/kg
12N4	Chinch12	-0.583	TS-30mg/kg
10N4	Chinch10	-1.664	TS-30mg/kg
17N4	Chinch17	-2.012	anti-rsPilA
11N4	Chinch11	-0.343	anti-rsPilA
15N4	Chinch15	0.372	TS-15mg/kg
18N4	Chinch18	-1.341	TS-15mg/kg
14N4	Chinch14	-0.961	TS-15mg/kg
27N4	Chinch27	0.916	anti-tip-chimer
21N4	Chinch21	1.250	anti-tip-chimer
19N4	Chinch19	-0.233	anti-tip-chimer
20N4	Chinch20	-0.910	AC-5mg/kg
23N4	Chinch23	-0.274	AC-5mg/kg
26N4	Chinch26	-0.291	AC-5mg/kg
25N4	Chinch25	-0.554	AC-10mg/kg
22N4	Chinch22	-0.310	AC-10mg/kg

Supplementary File 2
Longitudinal Pairwise-Distances and Pairwise-Differences

NPL FaithPD Pairwise-Difference Mann-Whitney Pairwise Comparisons
Baseline - 2DPT

Group A	Group B	Mann-Whitney U	P-value	FDR P-value
AC-PO7d	AC-5mg/kg	0	0.100	0.600
AC-PO7d	AC-10mg/kg	0	0.200	0.800
AC-PO7d	Ofloxacin	0	0.100	0.600
AC-PO7d	Saline	0	0.100	0.600
AC-PO7d	TS-30mg/kg	2	0.400	0.900
AC-PO7d	TS-15mg/kg	0	0.100	0.600
AC-PO7d	anti-tip-chimer	0	0.100	0.600
AC-PO7d	anti-rsPilA	0	0.100	0.600
AC-5mg/kg	AC-10mg/kg	5	0.400	0.900
Ofloxacin	AC-5mg/kg	1	0.200	0.800
Ofloxacin	AC-10mg/kg	3	1.000	1.000
Ofloxacin	Saline	2	0.400	0.900
Ofloxacin	TS-30mg/kg	3	0.700	1.000
Ofloxacin	TS-15mg/kg	5	1.000	1.000
Ofloxacin	anti-tip-chimer	6	0.700	1.000
Ofloxacin	anti-rsPilA	3	0.700	1.000
Saline	AC-5mg/kg	2	0.400	0.900
Saline	AC-10mg/kg	3	1.000	1.000
Saline	TS-30mg/kg	5	1.000	1.000
Saline	TS-15mg/kg	6	0.700	1.000
Saline	anti-tip-chimer	6	0.700	1.000
Saline	anti-rsPilA	7	0.400	0.900
TS-30mg/kg	AC-5mg/kg	4	1.000	1.000
TS-30mg/kg	AC-10mg/kg	3	1.000	1.000
TS-30mg/kg	TS-15mg/kg	5	1.000	1.000
TS-30mg/kg	anti-tip-chimer	5	1.000	1.000
TS-30mg/kg	anti-rsPilA	4	1.000	1.000
TS-15mg/kg	AC-5mg/kg	1	0.200	0.800
TS-15mg/kg	AC-10mg/kg	3	1.000	1.000
TS-15mg/kg	anti-tip-chimer	4	1.000	1.000
anti-tip-chimer	AC-5mg/kg	2	0.400	0.900
anti-tip-chimer	AC-10mg/kg	2	0.800	1.000
anti-rsPilA	AC-5mg/kg	2	0.400	0.900
anti-rsPilA	AC-10mg/kg	2	0.800	1.000
anti-rsPilA	TS-15mg/kg	4	1.000	1.000
anti-rsPilA	anti-tip-chimer	4	1.000	1.000

Kruskal-Wallis (Omnibus Test)

**H**	9.490
**p-value**	0.303

Supplementary File 2
Longitudinal Pairwise-Distances and Pairwise-Differences

NPL FaithPD Pairwise-Difference Mann-Whitney Pairwise Comparisons
Baseline - 5DPT

Group A	Group B	Mann-Whitney U	P-value	FDR P-value
AC-PO7d	AC-5mg/kg	3	0.700	1.000
AC-PO7d	AC-10mg/kg	2	0.800	1.000
AC-PO7d	Ofloxacin	1	0.200	1.000
AC-PO7d	Saline	4	1.000	1.000
AC-PO7d	TS-30mg/kg	4	1.000	1.000
AC-PO7d	TS-15mg/kg	3	0.700	1.000
AC-PO7d	anti-tip-chimer	3	0.700	1.000
AC-PO7d	anti-rsPilA	2	0.800	1.000
AC-5mg/kg	AC-10mg/kg	2	0.800	1.000
Ofloxacin	AC-5mg/kg	8	0.200	1.000
Ofloxacin	AC-10mg/kg	4	0.800	1.000
Ofloxacin	Saline	7	0.400	1.000
Ofloxacin	TS-30mg/kg	7	0.400	1.000
Ofloxacin	TS-15mg/kg	6	0.700	1.000
Ofloxacin	anti-tip-chimer	7	0.400	1.000
Ofloxacin	anti-rsPilA	5	0.400	1.000
Saline	AC-5mg/kg	5	1.000	1.000
Saline	AC-10mg/kg	2	0.800	1.000
Saline	TS-30mg/kg	4	1.000	1.000
Saline	TS-15mg/kg	3	0.700	1.000
Saline	anti-tip-chimer	4	1.000	1.000
Saline	anti-rsPilA	2	0.800	1.000
TS-30mg/kg	AC-5mg/kg	3	0.700	1.000
TS-30mg/kg	AC-10mg/kg	2	0.800	1.000
TS-30mg/kg	TS-15mg/kg	4	1.000	1.000
TS-30mg/kg	anti-tip-chimer	3	0.700	1.000
TS-30mg/kg	anti-rsPilA	4	0.800	1.000
TS-15mg/kg	AC-5mg/kg	5	1.000	1.000
TS-15mg/kg	AC-10mg/kg	2	0.800	1.000
TS-15mg/kg	anti-tip-chimer	4	1.000	1.000
anti-tip-chimer	AC-5mg/kg	6	0.700	1.000
anti-tip-chimer	AC-10mg/kg	2	0.800	1.000
anti-rsPilA	AC-5mg/kg	3	1.000	1.000
anti-rsPilA	AC-10mg/kg	2	1.000	1.000
anti-rsPilA	TS-15mg/kg	3	1.000	1.000
anti-rsPilA	anti-tip-chimer	3	1.000	1.000

Kruskal-Wallis (Omnibus Test)

**H**	3.711
**p-value**	0.882

Supplementary File 2
Longitudinal Pairwise-Distances and Pairwise-Differences

NPL FaithPD Pairwise-Difference Mann-Whitney Pairwise Comparisons
Baseline - 7DPT

Group A	Group B	Mann-Whitney U	P-value	FDR P-value
AC-PO7d	AC-5mg/kg	0	0.200	1.000
AC-PO7d	AC-10mg/kg	0	0.667	1.000
AC-PO7d	Ofloxacin	0	0.200	1.000
AC-PO7d	Saline	2	0.800	1.000
AC-PO7d	TS-30mg/kg	0	0.333	1.000
AC-PO7d	TS-15mg/kg	0	0.200	1.000
AC-PO7d	anti-tip-chimer	0	0.333	1.000
AC-PO7d	anti-rsPilA	0	0.333	1.000
AC-5mg/kg	AC-10mg/kg	0	0.500	1.000
Ofloxacin	AC-5mg/kg	9	0.100	1.000
Ofloxacin	AC-10mg/kg	2	1.000	1.000
Ofloxacin	Saline	5	1.000	1.000
Ofloxacin	TS-30mg/kg	3	1.000	1.000
Ofloxacin	TS-15mg/kg	5	1.000	1.000
Ofloxacin	anti-tip-chimer	2	0.800	1.000
Ofloxacin	anti-rsPilA	3	1.000	1.000
Saline	AC-5mg/kg	6	0.700	1.000
Saline	AC-10mg/kg	2	1.000	1.000
Saline	TS-30mg/kg	3	1.000	1.000
Saline	TS-15mg/kg	4	1.000	1.000
Saline	anti-tip-chimer	2	0.800	1.000
Saline	anti-rsPilA	3	1.000	1.000
TS-30mg/kg	AC-5mg/kg	4	0.800	1.000
TS-30mg/kg	AC-10mg/kg	1	1.000	1.000
TS-30mg/kg	TS-15mg/kg	3	1.000	1.000
TS-30mg/kg	anti-tip-chimer	2	1.000	1.000
TS-30mg/kg	anti-rsPilA	2	1.000	1.000
TS-15mg/kg	AC-5mg/kg	6	0.700	1.000
TS-15mg/kg	AC-10mg/kg	2	1.000	1.000
TS-15mg/kg	anti-tip-chimer	2	0.800	1.000
anti-tip-chimer	AC-5mg/kg	6	0.200	1.000
anti-tip-chimer	AC-10mg/kg	2	0.667	1.000
anti-rsPilA	AC-5mg/kg	6	0.200	1.000
anti-rsPilA	AC-10mg/kg	1	1.000	1.000
anti-rsPilA	TS-15mg/kg	3	1.000	1.000
anti-rsPilA	anti-tip-chimer	2	1.000	1.000

Kruskal-Wallis (Omnibus Test)

**H**	7.411
**p-value**	0.493

NPL FaithPD Pairwise-Difference Mann-Whitney Pairwise Comparisons

Baseline - 9DPT

Group A	Group B	Mann-Whitney U	P-value	FDR P-value
AC-PO7d	AC-5mg/kg	4	0.800	0.900
AC-PO7d	AC-10mg/kg	2	1.000	1.000
AC-PO7d	Ofloxacin	1	0.667	0.900
AC-PO7d	Saline	1	0.667	0.900
AC-PO7d	TS-30mg/kg	3	1.000	1.000
AC-PO7d	TS-15mg/kg	4	0.800	0.900
AC-PO7d	anti-tip-chimer	1	0.400	0.900
AC-PO7d	anti-rsPilA	3	0.667	0.900
AC-5mg/kg	AC-10mg/kg	4	0.800	0.900
Ofloxacin	AC-5mg/kg	4	0.800	0.900
Ofloxacin	AC-10mg/kg	3	0.667	0.900
Ofloxacin	Saline	1	0.667	0.900
Ofloxacin	TS-30mg/kg	4	0.800	0.900
Ofloxacin	TS-15mg/kg	4	0.800	0.900
Ofloxacin	anti-tip-chimer	1	0.400	0.900
Ofloxacin	anti-rsPilA	3	0.667	0.900
Saline	AC-5mg/kg	4	0.800	0.900
Saline	AC-10mg/kg	3	0.667	0.900
Saline	TS-30mg/kg	4	0.800	0.900
Saline	TS-15mg/kg	5	0.400	0.900
Saline	anti-tip-chimer	1	0.400	0.900
Saline	anti-rsPilA	3	0.667	0.900
TS-30mg/kg	AC-5mg/kg	4	1.000	1.000
TS-30mg/kg	AC-10mg/kg	2	0.800	0.900
TS-30mg/kg	TS-15mg/kg	5	1.000	1.000
TS-30mg/kg	anti-tip-chimer	3	0.700	0.900
TS-30mg/kg	anti-rsPilA	4	0.800	0.900
TS-15mg/kg	AC-5mg/kg	3	0.700	0.900
TS-15mg/kg	AC-10mg/kg	2	0.800	0.900
TS-15mg/kg	anti-tip-chimer	1	0.200	0.900
anti-tip-chimer	AC-5mg/kg	9	0.100	0.900
anti-tip-chimer	AC-10mg/kg	6	0.200	0.900
anti-rsPilA	AC-5mg/kg	1	0.400	0.900
anti-rsPilA	AC-10mg/kg	1	0.667	0.900
anti-rsPilA	TS-15mg/kg	2	0.800	0.900
anti-rsPilA	anti-tip-chimer	0	0.200	0.900

Kruskal-Wallis (Omnibus Test)

**H**	6.601
**p-value**	0.580

Supplementary File 2
Longitudinal Pairwise-Distances and Pairwise-Differences

NPL Richness Pairwise-Differences

Baseline - 2DPT

#SampleID	SubjectID	Difference	Group
1AN2	Chinch1	-66.000	AC-PO7d
3AN2	Chinch3	-10.000	AC-PO7d
2AN2	Chinch2	-36.000	AC-PO7d
5N1	Chinch5	2.000	Ofloxacin
4N1	Chinch4	-4.000	Ofloxacin
7N1	Chinch7	-9.000	Ofloxacin
6N1	Chinch6	-4.000	Saline
8N1	Chinch8	9.000	Saline
9N1	Chinch9	5.000	Saline
10N1	Chinch10	-24.000	TS-30mg/kg
16N1	Chinch16	6.000	TS-30mg/kg
12N1	Chinch12	-1.000	TS-30mg/kg
13N1	Chinch13	5.000	anti-rsPilA
17N1	Chinch17	-14.000	anti-rsPilA
11N1	Chinch11	-15.000	anti-rsPilA
15N1	Chinch15	29.000	TS-15mg/kg
14N1	Chinch14	10.000	TS-15mg/kg
18N1	Chinch18	-12.000	TS-15mg/kg
19N1	Chinch19	10.000	anti-tip-chimer
21N1	Chinch21	5.000	anti-tip-chimer
27N1	Chinch27	-15.000	anti-tip-chimer
20N1	Chinch20	8.000	AC-5mg/kg
26N1	Chinch26	9.000	AC-5mg/kg
23N1	Chinch23	7.000	AC-5mg/kg
22N1	Chinch22	0.000	AC-10mg/kg
25N1	Chinch25	-16.000	AC-10mg/kg

Supplementary File 2
Longitudinal Pairwise-Distances and Pairwise-Differences

NPL Richness Pairwise-Differences

Baseline - 5DPT

#SampleID	SubjectID	Difference	Group
1AN5	Chinch1	-59.000	AC-PO7d
2AN5	Chinch2	-27.000	AC-PO7d
3AN5	Chinch3	18.000	AC-PO7d
7N2	Chinch7	7.000	Ofloxacin
5N2	Chinch5	0.000	Ofloxacin
4N2	Chinch4	0.000	Ofloxacin
9N2	Chinch9	-46.000	Saline
6N2	Chinch6	5.000	Saline
8N2	Chinch8	8.000	Saline
16N2	Chinch16	-4.000	TS-30mg/kg
12N2	Chinch12	-10.000	TS-30mg/kg
10N2	Chinch10	-20.000	TS-30mg/kg
17N2	Chinch17	-21.000	anti-rsPilA
11N2	Chinch11	0.000	anti-rsPilA
18N2	Chinch18	3.000	TS-15mg/kg
14N2	Chinch14	-11.000	TS-15mg/kg
15N2	Chinch15	46.000	TS-15mg/kg
27N2	Chinch27	-6.000	anti-tip-chimer
21N2	Chinch21	-2.000	anti-tip-chimer
19N2	Chinch19	-3.000	anti-tip-chimer
20N2	Chinch20	-11.000	AC-5mg/kg
23N2	Chinch23	3.000	AC-5mg/kg
26N2	Chinch26	2.000	AC-5mg/kg
25N2	Chinch25	-11.000	AC-10mg/kg
22N2	Chinch22	5.000	AC-10mg/kg

Supplementary File 2
Longitudinal Pairwise-Distances and Pairwise-Differences

NPL Richness Pairwise-Differences

Baseline - 7DPT

#SampleID	SubjectID	Difference	Group
1AN7	Chinch1	-22.000	AC-PO7d
2AN7	Chinch2	-43.000	AC-PO7d
4N3	Chinch4	-1.000	Ofloxacin
7N3	Chinch7	0.000	Ofloxacin
5N3	Chinch5	-3.000	Ofloxacin
6N3	Chinch6	21.000	Saline
8N3	Chinch8	16.000	Saline
9N3	Chinch9	-38.000	Saline
16N3	Chinch16	5.000	TS-30mg/kg
10N3	Chinch10	-20.000	TS-30mg/kg
17N3	Chinch17	-11.000	anti-rsPilA
11N3	Chinch11	4.000	anti-rsPilA
14N3	Chinch14	-11.000	TS-15mg/kg
18N3	Chinch18	2.000	TS-15mg/kg
15N3	Chinch15	17.000	TS-15mg/kg
27N3	Chinch27	-17.000	anti-tip-chimer
21N3	Chinch21	9.000	anti-tip-chimer
20N3	Chinch20	-3.000	AC-5mg/kg
26N3	Chinch26	-4.000	AC-5mg/kg
23N3	Chinch23	-3.000	AC-5mg/kg
25N3	Chinch25	-20.000	AC-10mg/kg

Supplementary File 2
Longitudinal Pairwise-Distances and Pairwise-Differences

NPL Richness Pairwise-Differences

Baseline - 9DPT

#SampleID	SubjectID	Difference	Group
2AN9	Chinch2	-12.000	AC-PO7d
3AN9	Chinch3	18.000	AC-PO7d
5N4	Chinch5	-8.000	Ofloxacin
7N4	Chinch7	-4.000	Ofloxacin
6N4	Chinch6	12.000	Saline
8N4	Chinch8	14.000	Saline
16N4	Chinch16	-2.000	TS-30mg/kg
10N4	Chinch10	-26.000	TS-30mg/kg
12N4	Chinch12	1.000	TS-30mg/kg
17N4	Chinch17	-20.000	anti-rsPilA
11N4	Chinch11	-24.000	anti-rsPilA
14N4	Chinch14	-1.000	TS-15mg/kg
15N4	Chinch15	21.000	TS-15mg/kg
18N4	Chinch18	-5.000	TS-15mg/kg
21N4	Chinch21	36.000	anti-tip-chimer
19N4	Chinch19	12.000	anti-tip-chimer
27N4	Chinch27	-1.000	anti-tip-chimer
23N4	Chinch23	18.000	AC-5mg/kg
20N4	Chinch20	-11.000	AC-5mg/kg
26N4	Chinch26	5.000	AC-5mg/kg
25N4	Chinch25	-17.000	AC-10mg/kg
22N4	Chinch22	1.000	AC-10mg/kg

Supplementary File 2
Longitudinal Pairwise-Distances and Pairwise-Differences

NPL Richness Pairwise-Difference Mann-Whitney Pairwise Comparisons
Baseline - 2DPT

Group A	Group B	Mann-Whitney U	P-value	FDR P-value
AC-PO7d	AC-5mg/kg	0	0.100	0.600
AC-PO7d	AC-10mg/kg	1	0.400	0.758
AC-PO7d	Ofloxacin	0	0.100	0.600
AC-PO7d	Saline	0	0.100	0.600
AC-PO7d	TS-30mg/kg	1	0.200	0.655
AC-PO7d	TS-15mg/kg	1	0.200	0.655
AC-PO7d	anti-tip-chimer	1	0.200	0.655
AC-PO7d	anti-rsPilA	2	0.400	0.758
AC-5mg/kg	AC-10mg/kg	6	0.200	0.655
Ofloxacin	AC-5mg/kg	0	0.100	0.600
Ofloxacin	AC-10mg/kg	4	0.800	0.900
Ofloxacin	Saline	1.5	0.268	0.743
Ofloxacin	TS-30mg/kg	4	1.000	1.000
Ofloxacin	TS-15mg/kg	3	0.700	0.840
Ofloxacin	anti-tip-chimer	3	0.700	0.840
Ofloxacin	anti-rsPilA	6	0.700	0.840
Saline	AC-5mg/kg	2.5	0.507	0.840
Saline	AC-10mg/kg	5	0.400	0.758
Saline	TS-30mg/kg	6	0.700	0.840
Saline	TS-15mg/kg	3	0.700	0.840
Saline	anti-tip-chimer	4.5	1.000	1.000
Saline	anti-rsPilA	7.5	0.268	0.743
TS-30mg/kg	AC-5mg/kg	0	0.100	0.600
TS-30mg/kg	AC-10mg/kg	3	1.000	1.000
TS-30mg/kg	TS-15mg/kg	2	0.400	0.758
TS-30mg/kg	anti-tip-chimer	3	0.700	0.840
TS-30mg/kg	anti-rsPilA	5	1.000	1.000
TS-15mg/kg	AC-5mg/kg	6	0.700	0.840
TS-15mg/kg	AC-10mg/kg	5	0.400	0.758
TS-15mg/kg	anti-tip-chimer	6.5	0.507	0.840
anti-tip-chimer	AC-5mg/kg	3	0.700	0.840
anti-tip-chimer	AC-10mg/kg	5	0.400	0.758
anti-rsPilA	AC-5mg/kg	0	0.100	0.600
anti-rsPilA	AC-10mg/kg	4	0.800	0.900
anti-rsPilA	TS-15mg/kg	1	0.200	0.655
anti-rsPilA	anti-tip-chimer	3	0.653	0.840

Kruskal-Wallis (Omnibus Test)

**H**	12.036
**p-value**	0.150

NPL Richness Pairwise-Difference Mann-Whitney Pairwise Comparisons
Baseline - 5DPT

Group A	Group B	Mann-Whitney U	P-value	FDR P-value
AC-PO7d	AC-5mg/kg	3	0.700	1.000
AC-PO7d	AC-10mg/kg	2	0.800	1.000
AC-PO7d	Ofloxacin	3	0.658	1.000
AC-PO7d	Saline	4	1.000	1.000
AC-PO7d	TS-30mg/kg	3	0.700	1.000
AC-PO7d	TS-15mg/kg	2	0.400	1.000
AC-PO7d	anti-tip-chimer	3	0.700	1.000
AC-PO7d	anti-rsPilA	2	0.800	1.000
AC-5mg/kg	AC-10mg/kg	2.5	1.000	1.000
Ofloxacin	AC-5mg/kg	5	1.000	1.000
Ofloxacin	AC-10mg/kg	4	0.767	1.000
Ofloxacin	Saline	4	1.000	1.000
Ofloxacin	TS-30mg/kg	9	0.077	1.000
Ofloxacin	TS-15mg/kg	4	1.000	1.000
Ofloxacin	anti-tip-chimer	9	0.077	1.000
Ofloxacin	anti-rsPilA	5	0.333	1.000
Saline	AC-5mg/kg	6	0.700	1.000
Saline	AC-10mg/kg	3.5	1.000	1.000
Saline	TS-30mg/kg	6	0.700	1.000
Saline	TS-15mg/kg	4	1.000	1.000
Saline	anti-tip-chimer	6	0.700	1.000
Saline	anti-rsPilA	4	0.800	1.000
TS-30mg/kg	AC-5mg/kg	2	0.400	1.000
TS-30mg/kg	AC-10mg/kg	2	0.800	1.000
TS-30mg/kg	TS-15mg/kg	2	0.400	1.000
TS-30mg/kg	anti-tip-chimer	1	0.200	1.000
TS-30mg/kg	anti-rsPilA	3	1.000	1.000
TS-15mg/kg	AC-5mg/kg	6	0.653	1.000
TS-15mg/kg	AC-10mg/kg	3.5	1.000	1.000
TS-15mg/kg	anti-tip-chimer	6	0.700	1.000
anti-tip-chimer	AC-5mg/kg	3	0.700	1.000
anti-tip-chimer	AC-10mg/kg	3	1.000	1.000
anti-rsPilA	AC-5mg/kg	1	0.400	1.000
anti-rsPilA	AC-10mg/kg	1	0.667	1.000
anti-rsPilA	TS-15mg/kg	1	0.400	1.000
anti-rsPilA	anti-tip-chimer	3	1.000	1.000

Kruskal-Wallis (Omnibus Test)

**H**	4.730
**p-value**	0.786

NPL Richness Pairwise-Difference Mann-Whitney Pairwise Comparisons

Baseline - 7DPT

Group A	Group B	Mann-Whitney U	P-value	FDR P-value
AC-PO7d	AC-5mg/kg	0	0.139	1.000
AC-PO7d	AC-10mg/kg	0	0.667	1.000
AC-PO7d	Ofloxacin	0	0.200	1.000
AC-PO7d	Saline	1	0.400	1.000
AC-PO7d	TS-30mg/kg	0	0.333	1.000
AC-PO7d	TS-15mg/kg	0	0.200	1.000
AC-PO7d	anti-tip-chimer	0	0.333	1.000
AC-PO7d	anti-rsPilA	0	0.333	1.000
AC-5mg/kg	AC-10mg/kg	3	0.346	1.000
Ofloxacin	AC-5mg/kg	8	0.164	1.000
Ofloxacin	AC-10mg/kg	3	0.500	1.000
Ofloxacin	Saline	3	0.700	1.000
Ofloxacin	TS-30mg/kg	3	1.000	1.000
Ofloxacin	TS-15mg/kg	3	0.700	1.000
Ofloxacin	anti-tip-chimer	3	1.000	1.000
Ofloxacin	anti-rsPilA	3	1.000	1.000
Saline	AC-5mg/kg	6	0.658	1.000
Saline	AC-10mg/kg	2	1.000	1.000
Saline	TS-30mg/kg	4	0.800	1.000
Saline	TS-15mg/kg	5	1.000	1.000
Saline	anti-tip-chimer	4	0.800	1.000
Saline	anti-rsPilA	4	0.800	1.000
TS-30mg/kg	AC-5mg/kg	3	1.000	1.000
TS-30mg/kg	AC-10mg/kg	1.5	1.000	1.000
TS-30mg/kg	TS-15mg/kg	2	0.800	1.000
TS-30mg/kg	anti-tip-chimer	1	0.667	1.000
TS-30mg/kg	anti-rsPilA	2	1.000	1.000
TS-15mg/kg	AC-5mg/kg	6	0.658	1.000
TS-15mg/kg	AC-10mg/kg	3	0.500	1.000
TS-15mg/kg	anti-tip-chimer	4	0.800	1.000
anti-tip-chimer	AC-5mg/kg	3	1.000	1.000
anti-tip-chimer	AC-10mg/kg	2	0.667	1.000
anti-rsPilA	AC-5mg/kg	3	1.000	1.000
anti-rsPilA	AC-10mg/kg	2	0.667	1.000
anti-rsPilA	TS-15mg/kg	2.5	1.000	1.000
anti-rsPilA	anti-tip-chimer	2	1.000	1.000

Kruskal-Wallis (Omnibus Test)

**H**	7.151
**p-value**	0.520

NPL Richness Pairwise-Difference Mann-Whitney Pairwise Comparisons
Baseline - 9DPT

Group A	Group B	Mann-Whitney U	P-value	FDR P-value
AC-PO7d	AC-5mg/kg	2.5	1.000	1.000
AC-PO7d	AC-10mg/kg	3	0.667	0.993
AC-PO7d	Ofloxacin	2	1.000	1.000
AC-PO7d	Saline	2	1.000	1.000
AC-PO7d	TS-30mg/kg	4	0.800	0.993
AC-PO7d	TS-15mg/kg	2	0.800	0.993
AC-PO7d	anti-tip-chimer	2	0.800	0.993
AC-PO7d	anti-rsPilA	4	0.333	0.900
AC-5mg/kg	AC-10mg/kg	5	0.400	0.900
Ofloxacin	AC-5mg/kg	2	0.800	0.993
Ofloxacin	AC-10mg/kg	2	1.000	1.000
Ofloxacin	Saline	0	0.333	0.900
Ofloxacin	TS-30mg/kg	2	0.800	0.993
Ofloxacin	TS-15mg/kg	1	0.400	0.900
Ofloxacin	anti-tip-chimer	0	0.200	0.900
Ofloxacin	anti-rsPilA	4	0.333	0.900
Saline	AC-5mg/kg	4	0.800	0.993
Saline	AC-10mg/kg	4	0.333	0.900
Saline	TS-30mg/kg	6	0.200	0.900
Saline	TS-15mg/kg	4	0.800	0.993
Saline	anti-tip-chimer	3.5	1.000	1.000
Saline	anti-rsPilA	4	0.333	0.900
TS-30mg/kg	AC-5mg/kg	2	0.400	0.900
TS-30mg/kg	AC-10mg/kg	2.5	1.000	1.000
TS-30mg/kg	TS-15mg/kg	3	0.700	0.993
TS-30mg/kg	anti-tip-chimer	1	0.200	0.900
TS-30mg/kg	anti-rsPilA	4	0.800	0.993
TS-15mg/kg	AC-5mg/kg	5	1.000	1.000
TS-15mg/kg	AC-10mg/kg	4	0.800	0.993
TS-15mg/kg	anti-tip-chimer	2.5	0.507	0.993
anti-tip-chimer	AC-5mg/kg	6	0.700	0.993
anti-tip-chimer	AC-10mg/kg	5	0.400	0.900
anti-rsPilA	AC-5mg/kg	0	0.200	0.900
anti-rsPilA	AC-10mg/kg	0	0.333	0.900
anti-rsPilA	TS-15mg/kg	0	0.200	0.900
anti-rsPilA	anti-tip-chimer	0	0.200	0.900

Kruskal-Wallis (Omnibus Test)

**H**	9.657
**p-value**	0.290

Supplementary File 2
Longitudinal Pairwise-Distances and Pairwise-Differences

NPL Shannon Diversity Pairwise-Differences
Baseline - 2DPT

#SampleID	SubjectID	Difference	Group
2AN2	Chinch2	-0.960	AC-PO7d
1AN2	Chinch1	-0.715	AC-PO7d
3AN2	Chinch3	0.036	AC-PO7d
7N1	Chinch7	-0.359	Ofloxacin
4N1	Chinch4	-0.206	Ofloxacin
5N1	Chinch5	-0.156	Ofloxacin
8N1	Chinch8	0.137	Saline
6N1	Chinch6	0.080	Saline
9N1	Chinch9	0.100	Saline
12N1	Chinch12	-0.130	TS-30mg/kg
10N1	Chinch10	-0.721	TS-30mg/kg
16N1	Chinch16	0.232	TS-30mg/kg
11N1	Chinch11	-0.313	anti-rsPilA
17N1	Chinch17	-0.234	anti-rsPilA
13N1	Chinch13	-0.065	anti-rsPilA
18N1	Chinch18	-0.342	TS-15mg/kg
14N1	Chinch14	0.352	TS-15mg/kg
15N1	Chinch15	0.678	TS-15mg/kg
27N1	Chinch27	-0.297	anti-tip-chimer
19N1	Chinch19	0.193	anti-tip-chimer
21N1	Chinch21	0.135	anti-tip-chimer
20N1	Chinch20	0.088	AC-5mg/kg
23N1	Chinch23	0.035	AC-5mg/kg
26N1	Chinch26	0.032	AC-5mg/kg
22N1	Chinch22	0.103	AC-10mg/kg
25N1	Chinch25	-0.421	AC-10mg/kg

Supplementary File 2
Longitudinal Pairwise-Distances and Pairwise-Differences

NPL Shannon Diversity Pairwise-Differences
Baseline - 5DPT

#SampleID	SubjectID	Difference	Group
2AN5	Chinch2	-0.266	AC-PO7d
1AN5	Chinch1	-0.431	AC-PO7d
3AN5	Chinch3	-0.177	AC-PO7d
5N2	Chinch5	-0.025	Ofloxacin
7N2	Chinch7	0.132	Ofloxacin
4N2	Chinch4	-0.011	Ofloxacin
8N2	Chinch8	0.168	Saline
6N2	Chinch6	0.260	Saline
9N2	Chinch9	-2.156	Saline
12N2	Chinch12	-0.339	TS-30mg/kg
10N2	Chinch10	-0.506	TS-30mg/kg
16N2	Chinch16	0.081	TS-30mg/kg
11N2	Chinch11	-0.131	anti-rsPilA
17N2	Chinch17	-0.311	anti-rsPilA
15N2	Chinch15	0.894	TS-15mg/kg
18N2	Chinch18	-0.025	TS-15mg/kg
14N2	Chinch14	-0.173	TS-15mg/kg
21N2	Chinch21	0.008	anti-tip-chimer
19N2	Chinch19	-0.020	anti-tip-chimer
27N2	Chinch27	-0.217	anti-tip-chimer
26N2	Chinch26	0.096	AC-5mg/kg
23N2	Chinch23	0.058	AC-5mg/kg
20N2	Chinch20	-0.477	AC-5mg/kg
22N2	Chinch22	0.202	AC-10mg/kg
25N2	Chinch25	-0.452	AC-10mg/kg

Supplementary File 2
Longitudinal Pairwise-Distances and Pairwise-Differences

NPL Shannon Diversity Pairwise-Differences

Baseline - 7DPT

#SampleID	SubjectID	Difference	Group
1AN7	Chinch1	-0.425	AC-PO7d
2AN7	Chinch2	-2.059	AC-PO7d
5N3	Chinch5	-0.571	Ofloxacin
7N3	Chinch7	-0.029	Ofloxacin
4N3	Chinch4	-0.059	Ofloxacin
8N3	Chinch8	0.405	Saline
6N3	Chinch6	0.503	Saline
9N3	Chinch9	-1.926	Saline
10N3	Chinch10	-0.514	TS-30mg/kg
16N3	Chinch16	0.400	TS-30mg/kg
11N3	Chinch11	-0.234	anti-rsPilA
17N3	Chinch17	-0.055	anti-rsPilA
15N3	Chinch15	0.440	TS-15mg/kg
18N3	Chinch18	-0.127	TS-15mg/kg
14N3	Chinch14	-0.205	TS-15mg/kg
21N3	Chinch21	0.151	anti-tip-chimer
27N3	Chinch27	-0.413	anti-tip-chimer
26N3	Chinch26	-0.183	AC-5mg/kg
20N3	Chinch20	-0.131	AC-5mg/kg
23N3	Chinch23	-0.121	AC-5mg/kg
25N3	Chinch25	-0.584	AC-10mg/kg

Supplementary File 2
Longitudinal Pairwise-Distances and Pairwise-Differences

NPL Shannon Diversity Pairwise-Differences
Baseline - 9DPT

#SampleID	SubjectID	Difference	Group
2AN9	Chinch2	-1.154	AC-PO7d
3AN9	Chinch3	0.041	AC-PO7d
5N4	Chinch5	-0.885	Ofloxacin
7N4	Chinch7	-0.105	Ofloxacin
6N4	Chinch6	0.418	Saline
8N4	Chinch8	0.240	Saline
10N4	Chinch10	-0.825	TS-30mg/kg
12N4	Chinch12	-0.094	TS-30mg/kg
16N4	Chinch16	0.228	TS-30mg/kg
17N4	Chinch17	-0.364	anti-rsPilA
11N4	Chinch11	-0.713	anti-rsPilA
15N4	Chinch15	0.465	TS-15mg/kg
18N4	Chinch18	-0.187	TS-15mg/kg
14N4	Chinch14	-0.007	TS-15mg/kg
19N4	Chinch19	0.254	anti-tip-chimer
21N4	Chinch21	0.794	anti-tip-chimer
27N4	Chinch27	-0.086	anti-tip-chimer
26N4	Chinch26	-0.114	AC-5mg/kg
23N4	Chinch23	0.244	AC-5mg/kg
20N4	Chinch20	-0.561	AC-5mg/kg
25N4	Chinch25	-0.513	AC-10mg/kg
22N4	Chinch22	-0.445	AC-10mg/kg

NPL Shannon Diversity Pairwise-Difference Mann-Whitney Pairwise Comparisons
Baseline - 2DPT

Group A	Group B	Mann-Whitney U	P-value	FDR P-value
AC-PO7d	AC-5mg/kg	2	0.400	0.900
AC-PO7d	AC-10mg/kg	1	0.400	0.900
AC-PO7d	Ofloxacin	3	0.700	0.933
AC-PO7d	Saline	0	0.100	0.720
AC-PO7d	TS-30mg/kg	3	0.700	0.933
AC-PO7d	TS-15mg/kg	1	0.200	0.900
AC-PO7d	anti-tip-chimer	1	0.200	0.900
AC-PO7d	anti-rsPilA	3	0.700	0.933
AC-5mg/kg	AC-10mg/kg	3	1.000	1.000
Ofloxacin	AC-5mg/kg	0	0.100	0.720
Ofloxacin	AC-10mg/kg	3	1.000	1.000
Ofloxacin	Saline	0	0.100	0.720
Ofloxacin	TS-30mg/kg	3	0.700	0.933
Ofloxacin	TS-15mg/kg	2	0.400	0.900
Ofloxacin	anti-tip-chimer	2	0.400	0.900
Ofloxacin	anti-rsPilA	4	1.000	1.000
Saline	AC-5mg/kg	8	0.200	0.900
Saline	AC-10mg/kg	4	0.800	1.000
Saline	TS-30mg/kg	6	0.700	0.933
Saline	TS-15mg/kg	3	0.700	0.933
Saline	anti-tip-chimer	4	1.000	1.000
Saline	anti-rsPilA	9	0.100	0.720
TS-30mg/kg	AC-5mg/kg	3	0.700	0.933
TS-30mg/kg	AC-10mg/kg	3	1.000	1.000
TS-30mg/kg	TS-15mg/kg	2	0.400	0.900
TS-30mg/kg	anti-tip-chimer	4	1.000	1.000
TS-30mg/kg	anti-rsPilA	5	1.000	1.000
TS-15mg/kg	AC-5mg/kg	6	0.700	0.933
TS-15mg/kg	AC-10mg/kg	5	0.400	0.900
TS-15mg/kg	anti-tip-chimer	6	0.700	0.933
anti-tip-chimer	AC-5mg/kg	6	0.700	0.933
anti-tip-chimer	AC-10mg/kg	5	0.400	0.900
anti-rsPilA	AC-5mg/kg	0	0.100	0.720
anti-rsPilA	AC-10mg/kg	3	1.000	1.000
anti-rsPilA	TS-15mg/kg	3	0.700	0.933
anti-rsPilA	anti-tip-chimer	2	0.400	0.900

Kruskal-Wallis (Omnibus Test)

**H**	8.749
**p-value**	0.364

Supplementary File 2
Longitudinal Pairwise-Distances and Pairwise-Differences

NPL Shannon Diversity Pairwise-Difference Mann-Whitney Pairwise Comparisons
Baseline - 5DPT

Group A	Group B	Mann-Whitney U	P-value	FDR P-value
AC-PO7d	AC-5mg/kg	3	0.700	1.000
AC-PO7d	AC-10mg/kg	3	1.000	1.000
AC-PO7d	Ofloxacin	0	0.100	1.000
AC-PO7d	Saline	3	0.700	1.000
AC-PO7d	TS-30mg/kg	5	1.000	1.000
AC-PO7d	TS-15mg/kg	0	0.100	1.000
AC-PO7d	anti-tip-chimer	1	0.200	1.000
AC-PO7d	anti-rsPilA	2	0.800	1.000
AC-5mg/kg	AC-10mg/kg	2	0.800	1.000
Ofloxacin	AC-5mg/kg	5	1.000	1.000
Ofloxacin	AC-10mg/kg	3	1.000	1.000
Ofloxacin	Saline	3	0.700	1.000
Ofloxacin	TS-30mg/kg	7	0.400	1.000
Ofloxacin	TS-15mg/kg	5	1.000	1.000
Ofloxacin	anti-tip-chimer	6	0.700	1.000
Ofloxacin	anti-rsPilA	6	0.200	1.000
Saline	AC-5mg/kg	6	0.700	1.000
Saline	AC-10mg/kg	3	1.000	1.000
Saline	TS-30mg/kg	6	0.700	1.000
Saline	TS-15mg/kg	4	1.000	1.000
Saline	anti-tip-chimer	6	0.700	1.000
Saline	anti-rsPilA	4	0.800	1.000
TS-30mg/kg	AC-5mg/kg	3	0.700	1.000
TS-30mg/kg	AC-10mg/kg	2	0.800	1.000
TS-30mg/kg	TS-15mg/kg	2	0.400	1.000
TS-30mg/kg	anti-tip-chimer	3	0.700	1.000
TS-30mg/kg	anti-rsPilA	2	0.800	1.000
TS-15mg/kg	AC-5mg/kg	5	1.000	1.000
TS-15mg/kg	AC-10mg/kg	4	0.800	1.000
TS-15mg/kg	anti-tip-chimer	5	1.000	1.000
anti-tip-chimer	AC-5mg/kg	3	0.700	1.000
anti-tip-chimer	AC-10mg/kg	3	1.000	1.000
anti-rsPilA	AC-5mg/kg	2	0.800	1.000
anti-rsPilA	AC-10mg/kg	2	1.000	1.000
anti-rsPilA	TS-15mg/kg	1	0.400	1.000
anti-rsPilA	anti-tip-chimer	1	0.400	1.000

Kruskal-Wallis (Omnibus Test)

**H**	4.855
**p-value**	0.773

Supplementary File 2
Longitudinal Pairwise-Distances and Pairwise-Differences

NPL Shannon Diversity Pairwise-Difference Mann-Whitney Pairwise Comparisons
Baseline - 7DPT

Group A	Group B	Mann-Whitney U	P-value	FDR P-value
AC-PO7d	AC-5mg/kg	0	0.200	1.000
AC-PO7d	AC-10mg/kg	1	1.000	1.000
AC-PO7d	Ofloxacin	1	0.400	1.000
AC-PO7d	Saline	1	0.400	1.000
AC-PO7d	TS-30mg/kg	1	0.667	1.000
AC-PO7d	TS-15mg/kg	0	0.200	1.000
AC-PO7d	anti-tip-chimer	0	0.333	1.000
AC-PO7d	anti-rsPilA	0	0.333	1.000
AC-5mg/kg	AC-10mg/kg	3	0.500	1.000
Ofloxacin	AC-5mg/kg	6	0.700	1.000
Ofloxacin	AC-10mg/kg	3	0.500	1.000
Ofloxacin	Saline	3	0.700	1.000
Ofloxacin	TS-30mg/kg	2	0.800	1.000
Ofloxacin	TS-15mg/kg	4	1.000	1.000
Ofloxacin	anti-tip-chimer	2	0.800	1.000
Ofloxacin	anti-rsPilA	3	1.000	1.000
Saline	AC-5mg/kg	6	0.700	1.000
Saline	AC-10mg/kg	2	1.000	1.000
Saline	TS-30mg/kg	4	0.800	1.000
Saline	TS-15mg/kg	5	1.000	1.000
Saline	anti-tip-chimer	4	0.800	1.000
Saline	anti-rsPilA	4	0.800	1.000
TS-30mg/kg	AC-5mg/kg	3	1.000	1.000
TS-30mg/kg	AC-10mg/kg	2	0.667	1.000
TS-30mg/kg	TS-15mg/kg	2	0.800	1.000
TS-30mg/kg	anti-tip-chimer	2	1.000	1.000
TS-30mg/kg	anti-rsPilA	2	1.000	1.000
TS-15mg/kg	AC-5mg/kg	5	1.000	1.000
TS-15mg/kg	AC-10mg/kg	3	0.500	1.000
TS-15mg/kg	anti-tip-chimer	4	0.800	1.000
anti-tip-chimer	AC-5mg/kg	3	1.000	1.000
anti-tip-chimer	AC-10mg/kg	2	0.667	1.000
anti-rsPilA	AC-5mg/kg	3	1.000	1.000
anti-rsPilA	AC-10mg/kg	2	0.667	1.000
anti-rsPilA	TS-15mg/kg	2	0.800	1.000
anti-rsPilA	anti-tip-chimer	2	1.000	1.000

Kruskal-Wallis (Omnibus Test)

**H**	5.935
**p-value**	0.655

Supplementary File 2
Longitudinal Pairwise-Distances and Pairwise-Differences

NPL Shannon Diversity Pairwise-Difference Mann-Whitney Pairwise Comparisons
Baseline - 9DPT

Group A	Group B	Mann-Whitney U	P-value	FDR P-value
AC-PO7d	AC-5mg/kg	2	0.800	1.000
AC-PO7d	AC-10mg/kg	2	1.000	1.000
AC-PO7d	Ofloxacin	2	1.000	1.000
AC-PO7d	Saline	0	0.333	0.847
AC-PO7d	TS-30mg/kg	2	0.800	1.000
AC-PO7d	TS-15mg/kg	2	0.800	1.000
AC-PO7d	anti-tip-chimer	1	0.400	0.847
AC-PO7d	anti-rsPilA	2	1.000	1.000
AC-5mg/kg	AC-10mg/kg	4	0.800	1.000
Ofloxacin	AC-5mg/kg	2	0.800	1.000
Ofloxacin	AC-10mg/kg	2	1.000	1.000
Ofloxacin	Saline	0	0.333	0.847
Ofloxacin	TS-30mg/kg	1	0.400	0.847
Ofloxacin	TS-15mg/kg	1	0.400	0.847
Ofloxacin	anti-tip-chimer	0	0.200	0.847
Ofloxacin	anti-rsPilA	2	1.000	1.000
Saline	AC-5mg/kg	5	0.400	0.847
Saline	AC-10mg/kg	4	0.333	0.847
Saline	TS-30mg/kg	6	0.200	0.847
Saline	TS-15mg/kg	4	0.800	1.000
Saline	anti-tip-chimer	3	1.000	1.000
Saline	anti-rsPilA	4	0.333	0.847
TS-30mg/kg	AC-5mg/kg	4	1.000	1.000
TS-30mg/kg	AC-10mg/kg	4	0.800	1.000
TS-30mg/kg	TS-15mg/kg	3	0.700	1.000
TS-30mg/kg	anti-tip-chimer	1	0.200	0.847
TS-30mg/kg	anti-rsPilA	4	0.800	1.000
TS-15mg/kg	AC-5mg/kg	6	0.700	1.000
TS-15mg/kg	AC-10mg/kg	6	0.200	0.847
TS-15mg/kg	anti-tip-chimer	3	0.700	1.000
anti-tip-chimer	AC-5mg/kg	8	0.200	0.847
anti-tip-chimer	AC-10mg/kg	6	0.200	0.847
anti-rsPilA	AC-5mg/kg	1	0.400	0.847
anti-rsPilA	AC-10mg/kg	2	1.000	1.000
anti-rsPilA	TS-15mg/kg	0	0.200	0.847
anti-rsPilA	anti-tip-chimer	0	0.200	0.847

Kruskal-Wallis (Omnibus Test)

**H**	10.545
**p-value**	0.229

Supplementary File 3
Alpha Diversity Output

id	Chinchilla_ID	Treatment	Sex	Collection	Fecal pielou_evenness
1BF	Chinch1	AC-PO7d	F	Baseline	0.77791084
2BF	Chinch2	AC-PO7d	F	Baseline	0.827754785
3BF	Chinch3	AC-PO7d	M	Baseline	0.816854269
20FBL	Chinch20	AC-5mg/kg	F	Baseline	0.828322138
23FBL	Chinch23	AC-5mg/kg	F	Baseline	0.745980937
22FBL	Chinch22	AC-10mg/kg	M	Baseline	0.780132726
25FBL	Chinch25	AC-10mg/kg	F	Baseline	0.866178634
11FBL	Chinch11	anti-rsPilA	F	Baseline	0.877743687
13FBL	Chinch13	anti-rsPilA	F	Baseline	0.864868998
17FBL	Chinch17	anti-rsPilA	F	Baseline	0.861692367
19FBL	Chinch19	anti-tip-chimer	F	Baseline	0.831320658
21FBL	Chinch21	anti-tip-chimer	F	Baseline	0.776413602
27FBL	Chinch27	anti-tip-chimer	M	Baseline	0.827772497
4FBL	Chinch4	Ofloxacin	F	Baseline	0.75064957
5FBL	Chinch5	Ofloxacin	F	Baseline	0.879030674
7FBL	Chinch7	Ofloxacin	M	Baseline	0.84574753
14FBL	Chinch14	TS-15mg/kg	F	Baseline	0.818756919
15FBL	Chinch15	TS-15mg/kg	F	Baseline	0.81440409
18FBL	Chinch18	TS-15mg/kg	M	Baseline	0.864388892
10FBL	Chinch10	TS-30mg/kg	F	Baseline	0.86657098
12FBL	Chinch12	TS-30mg/kg	M	Baseline	0.88699034
16FBL	Chinch16	TS-30mg/kg	F	Baseline	0.85027597
6FBL	Chinch6	Saline	F	Baseline	0.855281781
8FBL	Chinch8	Saline	M	Baseline	0.897263135
9FBL	Chinch9	Saline	F	Baseline	0.764069693
2AF2	Chinch2	AC-PO7d	F	1	0.573159694
3AF2	Chinch3	AC-PO7d	M	1	0.582110984
20F1	Chinch20	AC-5mg/kg	F	1	0.850530308
23F1	Chinch23	AC-5mg/kg	F	1	0.775245317
22F1	Chinch22	AC-10mg/kg	M	1	0.699831393
24F1	Chinch24	AC-10mg/kg	F	1	0.677034602
11F1	Chinch11	anti-rsPilA	F	1	0.795763758
13F1	Chinch13	anti-rsPilA	F	1	0.831412902
17F1	Chinch17	anti-rsPilA	F	1	0.842356857
19F1	Chinch19	anti-tip-chimer	F	1	0.761659417
21F1	Chinch21	anti-tip-chimer	F	1	0.837298745
27F1	Chinch27	anti-tip-chimer	M	1	0.821610398
4F1	Chinch4	Ofloxacin	F	1	0.799879236
7F1	Chinch7	Ofloxacin	M	1	0.789286237
14F1	Chinch14	TS-15mg/kg	F	1	0.847782921
15F1	Chinch15	TS-15mg/kg	F	1	0.80893302
18F1	Chinch18	TS-15mg/kg	M	1	0.827829939
10F1	Chinch10	TS-30mg/kg	F	1	0.795760407
12F1	Chinch12	TS-30mg/kg	M	1	0.763745539
16F1	Chinch16	TS-30mg/kg	F	1	0.879224011
6F1	Chinch6	Saline	F	1	0.876101988
8F1	Chinch8	Saline	M	1	0.863032726

Supplementary File 3
Alpha Diversity Output

id	Chinchilla_ID	Treatment	Sex	Collection	Fecal pielou_evenness
9F1	Chinch9	Saline	F	1	0.739670805
1AF5	Chinch1	AC-PO7d	F	2	0.85856679
2AF5	Chinch2	AC-PO7d	F	2	0.680898475
3AF5	Chinch3	AC-PO7d	M	2	0.847417205
20F2	Chinch20	AC-5mg/kg	F	2	0.737963835
23F2	Chinch23	AC-5mg/kg	F	2	0.709536548
26F2	Chinch26	AC-5mg/kg	M	2	0.834718251
22F2	Chinch22	AC-10mg/kg	M	2	0.687089324
25F2	Chinch25	AC-10mg/kg	F	2	0.661783441
11F2	Chinch11	anti-rsPilA	F	2	0.851837511
17F2	Chinch17	anti-rsPilA	F	2	0.823188284
19F2	Chinch19	anti-tip-chimer	F	2	0.730629506
21F2	Chinch21	anti-tip-chimer	F	2	0.851081987
27F2	Chinch27	anti-tip-chimer	M	2	0.796986893
4F2	Chinch4	Ofloxacin	F	2	0.790309027
5F2	Chinch5	Ofloxacin	F	2	0.844327807
7F2	Chinch7	Ofloxacin	M	2	0.862715607
14F2	Chinch14	TS-15mg/kg	F	2	0.786429108
15F2	Chinch15	TS-15mg/kg	F	2	0.847252977
18F2	Chinch18	TS-15mg/kg	M	2	0.808810778
10F2	Chinch10	TS-30mg/kg	F	2	0.82444362
12F2	Chinch12	TS-30mg/kg	M	2	0.6972746
16F2	Chinch16	TS-30mg/kg	F	2	0.872380663
6F2	Chinch6	Saline	F	2	0.847761244
8F2	Chinch8	Saline	M	2	0.862556882
9F2	Chinch9	Saline	F	2	0.789065304
1AF7	Chinch1	AC-PO7d	F	3	0.682252845
2AF7	Chinch2	AC-PO7d	F	3	0.626600529
3AF7	Chinch3	AC-PO7d	M	3	0.564976112
20F3	Chinch20	AC-5mg/kg	F	3	0.80428963
23F3	Chinch23	AC-5mg/kg	F	3	0.801174484
26F3	Chinch26	AC-5mg/kg	M	3	0.816563642
25F3	Chinch25	AC-10mg/kg	F	3	0.779214958
11F3	Chinch11	anti-rsPilA	F	3	0.850675695
17F3	Chinch17	anti-rsPilA	F	3	0.91208585
19F3	Chinch19	anti-tip-chimer	F	3	0.795382714
21F3	Chinch21	anti-tip-chimer	F	3	0.796182281
4F3	Chinch4	Ofloxacin	F	3	0.786165248
5F3	Chinch5	Ofloxacin	F	3	0.842091174
7F3	Chinch7	Ofloxacin	M	3	0.84032973
14F3	Chinch14	TS-15mg/kg	F	3	0.777064518
15F3	Chinch15	TS-15mg/kg	F	3	0.799122136
18F3	Chinch18	TS-15mg/kg	M	3	0.780020418
10F3	Chinch10	TS-30mg/kg	F	3	0.817567305
12F3	Chinch12	TS-30mg/kg	M	3	0.750992511
16F3	Chinch16	TS-30mg/kg	F	3	0.86553538
6F3	Chinch6	Saline	F	3	0.853516448
8F3	Chinch8	Saline	M	3	0.867662915

Supplementary File 3
Alpha Diversity Output

id	Chinchilla_ID	Treatment	Sex	Collection	Fecal pielou_evenness
2AF9	Chinch2	AC-PO7d	F	4	0.818095476
3AF9	Chinch3	AC-PO7d	M	4	0.634591
20F4	Chinch20	AC-5mg/kg	F	4	0.799178555
23F4	Chinch23	AC-5mg/kg	F	4	0.79312887
26F4	Chinch26	AC-5mg/kg	M	4	0.852568378
22F4	Chinch22	AC-10mg/kg	M	4	0.747974712
25F4	Chinch25	AC-10mg/kg	F	4	0.76996706
11F4	Chinch11	anti-rsPilA	F	4	0.815937136
17F4	Chinch17	anti-rsPilA	F	4	0.829316297
19F4	Chinch19	anti-tip-chimer	F	4	0.828286603
21F4	Chinch21	anti-tip-chimer	F	4	0.863140337
27F4	Chinch27	anti-tip-chimer	M	4	0.859970565
4F4	Chinch4	Ofloxacin	F	4	0.821209809
5F4	Chinch5	Ofloxacin	F	4	0.853129181
7F4	Chinch7	Ofloxacin	M	4	0.803119211
14F4	Chinch14	TS-15mg/kg	F	4	0.798562181
15F4	Chinch15	TS-15mg/kg	F	4	0.844970671
18F4	Chinch18	TS-15mg/kg	M	4	0.821822617
10F4	Chinch10	TS-30mg/kg	F	4	0.854186089
12F4	Chinch12	TS-30mg/kg	M	4	0.815346163
16F4	Chinch16	TS-30mg/kg	F	4	0.717195775
8F4	Chinch8	Saline	M	4	0.848701668

Supplementary File 3
Alpha Diversity Output

Fecal Evenness-Baseline

Kruskal-Wallis pairwise comparisons

Group 1	Group 2	H	p-value	q-value
AC-PO7d (n=3)	AC-5mg/kg (n=2)	0.000	1.000	1.000
AC-PO7d (n=3)	AC-10mg/kg (n=2)	0.333	0.564	0.752
AC-PO7d (n=3)	Ofloxacin (n=3)	0.429	0.513	0.752
AC-PO7d (n=3)	Saline (n=3)	0.429	0.513	0.752
AC-PO7d (n=3)	TS-30mg/kg (n=3)	3.857	0.050	0.446
AC-PO7d (n=3)	TS-15mg/kg (n=3)	0.429	0.513	0.752
AC-PO7d (n=3)	anti-tip-chimer (n=3)	0.429	0.513	0.752
AC-PO7d (n=3)	anti-rsPilA (n=3)	3.857	0.050	0.446
AC-5mg/kg (n=2)	AC-10mg/kg (n=2)	0.600	0.439	0.752
AC-5mg/kg (n=2)	Ofloxacin (n=3)	1.333	0.248	0.752
AC-5mg/kg (n=2)	Saline (n=3)	1.333	0.248	0.752
AC-5mg/kg (n=2)	TS-30mg/kg (n=3)	3.000	0.083	0.500
AC-5mg/kg (n=2)	TS-15mg/kg (n=3)	0.333	0.564	0.752
AC-5mg/kg (n=2)	anti-tip-chimer (n=3)	0.333	0.564	0.752
AC-5mg/kg (n=2)	anti-rsPilA (n=3)	3.000	0.083	0.500
AC-10mg/kg (n=2)	Ofloxacin (n=3)	0.000	1.000	1.000
AC-10mg/kg (n=2)	Saline (n=3)	0.000	1.000	1.000
AC-10mg/kg (n=2)	TS-30mg/kg (n=3)	1.333	0.248	0.752
AC-10mg/kg (n=2)	TS-15mg/kg (n=3)	0.000	1.000	1.000
AC-10mg/kg (n=2)	anti-tip-chimer (n=3)	0.333	0.564	0.752
AC-10mg/kg (n=2)	anti-rsPilA (n=3)	0.333	0.564	0.752
Ofloxacin (n=3)	Saline (n=3)	0.429	0.513	0.752
Ofloxacin (n=3)	TS-30mg/kg (n=3)	1.190	0.275	0.752
Ofloxacin (n=3)	TS-15mg/kg (n=3)	0.048	0.827	0.931
Ofloxacin (n=3)	anti-tip-chimer (n=3)	0.429	0.513	0.752
Ofloxacin (n=3)	anti-rsPilA (n=3)	0.429	0.513	0.752
Saline (n=3)	TS-30mg/kg (n=3)	0.048	0.827	0.931
Saline (n=3)	TS-15mg/kg (n=3)	0.048	0.827	0.931
Saline (n=3)	anti-tip-chimer (n=3)	0.429	0.513	0.752
Saline (n=3)	anti-rsPilA (n=3)	0.429	0.513	0.752
TS-30mg/kg (n=3)	TS-15mg/kg (n=3)	2.333	0.127	0.570
TS-30mg/kg (n=3)	anti-tip-chimer (n=3)	3.857	0.050	0.446
TS-30mg/kg (n=3)	anti-rsPilA (n=3)	0.048	0.827	0.931
TS-15mg/kg (n=3)	anti-tip-chimer (n=3)	0.048	0.827	0.931
TS-15mg/kg (n=3)	anti-rsPilA (n=3)	2.333	0.127	0.570
anti-tip-chimer (n=3)	anti-rsPilA (n=3)	3.857	0.050	0.446

Kruskal-Wallis (Omnibus Test)

**H**	9.123
**p-value**	0.332

Supplementary File 3
Alpha Diversity Output

Fecal Evenness-2DPT

Kruskal-Wallis pairwise comparisons

Group 1	Group 2	H	p-value	q-value
AC-PO7d (n=2)	AC-5mg/kg (n=2)	2.400	0.121	0.273
AC-PO7d (n=2)	AC-10mg/kg (n=2)	2.400	0.121	0.273
AC-PO7d (n=2)	Ofloxacin (n=2)	2.400	0.121	0.273
AC-PO7d (n=2)	Saline (n=3)	3.000	0.083	0.273
AC-PO7d (n=2)	TS-30mg/kg (n=3)	3.000	0.083	0.273
AC-PO7d (n=2)	TS-15mg/kg (n=3)	3.000	0.083	0.273
AC-PO7d (n=2)	anti-tip-chimer (n=3)	3.000	0.083	0.273
AC-PO7d (n=2)	anti-rsPilA (n=3)	3.000	0.083	0.273
AC-5mg/kg (n=2)	AC-10mg/kg (n=2)	2.400	0.121	0.273
AC-5mg/kg (n=2)	Ofloxacin (n=2)	0.000	1.000	1.000
AC-5mg/kg (n=2)	Saline (n=3)	0.333	0.564	0.725
AC-5mg/kg (n=2)	TS-30mg/kg (n=3)	0.000	1.000	1.000
AC-5mg/kg (n=2)	TS-15mg/kg (n=3)	0.000	1.000	1.000
AC-5mg/kg (n=2)	anti-tip-chimer (n=3)	0.333	0.564	0.725
AC-5mg/kg (n=2)	anti-rsPilA (n=3)	0.000	1.000	1.000
AC-10mg/kg (n=2)	Ofloxacin (n=2)	2.400	0.121	0.273
AC-10mg/kg (n=2)	Saline (n=3)	3.000	0.083	0.273
AC-10mg/kg (n=2)	TS-30mg/kg (n=3)	3.000	0.083	0.273
AC-10mg/kg (n=2)	TS-15mg/kg (n=3)	3.000	0.083	0.273
AC-10mg/kg (n=2)	anti-tip-chimer (n=3)	3.000	0.083	0.273
AC-10mg/kg (n=2)	anti-rsPilA (n=3)	3.000	0.083	0.273
Ofloxacin (n=2)	Saline (n=3)	0.333	0.564	0.725
Ofloxacin (n=2)	TS-30mg/kg (n=3)	0.000	1.000	1.000
Ofloxacin (n=2)	TS-15mg/kg (n=3)	3.000	0.083	0.273
Ofloxacin (n=2)	anti-tip-chimer (n=3)	0.333	0.564	0.725
Ofloxacin (n=2)	anti-rsPilA (n=3)	1.333	0.248	0.526
Saline (n=3)	TS-30mg/kg (n=3)	0.048	0.827	0.961
Saline (n=3)	TS-15mg/kg (n=3)	0.429	0.513	0.725
Saline (n=3)	anti-tip-chimer (n=3)	0.429	0.513	0.725
Saline (n=3)	anti-rsPilA (n=3)	0.429	0.513	0.725
TS-30mg/kg (n=3)	TS-15mg/kg (n=3)	0.429	0.513	0.725
TS-30mg/kg (n=3)	anti-tip-chimer (n=3)	0.048	0.827	0.961
TS-30mg/kg (n=3)	anti-rsPilA (n=3)	0.429	0.513	0.725
TS-15mg/kg (n=3)	anti-tip-chimer (n=3)	0.429	0.513	0.725
TS-15mg/kg (n=3)	anti-rsPilA (n=3)	0.048	0.827	0.961
anti-tip-chimer (n=3)	anti-rsPilA (n=3)	0.429	0.513	0.725

Kruskal-Wallis (Omnibus Test)

**H**	10.837
**p-value**	0.211

Supplementary File 3
Alpha Diversity Output

Fecal Evenness-5DPT

Kruskal-Wallis pairwise comparisons

Group 1	Group 2	H	p-value	q-value
AC-PO7d (n=3)	AC-5mg/kg (n=3)	0.429	0.513	0.839
AC-PO7d (n=3)	AC-10mg/kg (n=2)	1.333	0.248	0.661
AC-PO7d (n=3)	Ofloxacin (n=3)	0.048	0.827	0.931
AC-PO7d (n=3)	Saline (n=3)	0.429	0.513	0.839
AC-PO7d (n=3)	TS-30mg/kg (n=3)	0.048	0.827	0.931
AC-PO7d (n=3)	TS-15mg/kg (n=3)	0.429	0.513	0.839
AC-PO7d (n=3)	anti-tip-chimer (n=3)	0.048	0.827	0.931
AC-PO7d (n=3)	anti-rsPilA (n=2)	0.000	1.000	1.000
AC-5mg/kg (n=3)	AC-10mg/kg (n=2)	3.000	0.083	0.500
AC-5mg/kg (n=3)	Ofloxacin (n=3)	2.333	0.127	0.507
AC-5mg/kg (n=3)	Saline (n=3)	2.333	0.127	0.507
AC-5mg/kg (n=3)	TS-30mg/kg (n=3)	0.048	0.827	0.931
AC-5mg/kg (n=3)	TS-15mg/kg (n=3)	1.190	0.275	0.661
AC-5mg/kg (n=3)	anti-tip-chimer (n=3)	0.429	0.513	0.839
AC-5mg/kg (n=3)	anti-rsPilA (n=2)	1.333	0.248	0.661
AC-10mg/kg (n=2)	Ofloxacin (n=3)	3.000	0.083	0.500
AC-10mg/kg (n=2)	Saline (n=3)	3.000	0.083	0.500
AC-10mg/kg (n=2)	TS-30mg/kg (n=3)	3.000	0.083	0.500
AC-10mg/kg (n=2)	TS-15mg/kg (n=3)	3.000	0.083	0.500
AC-10mg/kg (n=2)	anti-tip-chimer (n=3)	3.000	0.083	0.500
AC-10mg/kg (n=2)	anti-rsPilA (n=2)	2.400	0.121	0.507
Ofloxacin (n=3)	Saline (n=3)	0.048	0.827	0.931
Ofloxacin (n=3)	TS-30mg/kg (n=3)	0.048	0.827	0.931
Ofloxacin (n=3)	TS-15mg/kg (n=3)	0.429	0.513	0.839
Ofloxacin (n=3)	anti-tip-chimer (n=3)	0.429	0.513	0.839
Ofloxacin (n=3)	anti-rsPilA (n=2)	0.000	1.000	1.000
Saline (n=3)	TS-30mg/kg (n=3)	0.048	0.827	0.931
Saline (n=3)	TS-15mg/kg (n=3)	1.190	0.275	0.661
Saline (n=3)	anti-tip-chimer (n=3)	0.429	0.513	0.839
Saline (n=3)	anti-rsPilA (n=2)	0.000	1.000	1.000
TS-30mg/kg (n=3)	TS-15mg/kg (n=3)	0.048	0.827	0.931
TS-30mg/kg (n=3)	anti-tip-chimer (n=3)	0.048	0.827	0.931
TS-30mg/kg (n=3)	anti-rsPilA (n=2)	0.000	1.000	1.000
TS-15mg/kg (n=3)	anti-tip-chimer (n=3)	0.048	0.827	0.931
TS-15mg/kg (n=3)	anti-rsPilA (n=2)	1.333	0.248	0.661
anti-tip-chimer (n=3)	anti-rsPilA (n=2)	1.333	0.248	0.661

Kruskal-Wallis (Omnibus Test)

**H**	7.778
**p-value**	0.455

Supplementary File 3
Alpha Diversity Output

Fecal Evenness-7DPT

Kruskal-Wallis pairwise comparisons

Group 1	Group 2	H	p-value	q-value
AC-PO7d (n=3)	AC-5mg/kg (n=3)	3.857	0.050	0.200
AC-PO7d (n=3)	AC-10mg/kg (n=1)	1.800	0.180	0.308
AC-PO7d (n=3)	Ofloxacin (n=3)	3.857	0.050	0.200
AC-PO7d (n=3)	Saline (n=2)	3.000	0.083	0.200
AC-PO7d (n=3)	TS-30mg/kg (n=3)	3.857	0.050	0.200
AC-PO7d (n=3)	TS-15mg/kg (n=3)	3.857	0.050	0.200
AC-PO7d (n=3)	anti-tip-chimer (n=2)	3.000	0.083	0.200
AC-PO7d (n=3)	anti-rsPilA (n=2)	3.000	0.083	0.200
AC-5mg/kg (n=3)	AC-10mg/kg (n=1)	1.800	0.180	0.308
AC-5mg/kg (n=3)	Ofloxacin (n=3)	0.429	0.513	0.634
AC-5mg/kg (n=3)	Saline (n=2)	3.000	0.083	0.200
AC-5mg/kg (n=3)	TS-30mg/kg (n=3)	0.429	0.513	0.634
AC-5mg/kg (n=3)	TS-15mg/kg (n=3)	3.857	0.050	0.200
AC-5mg/kg (n=3)	anti-tip-chimer (n=2)	3.000	0.083	0.200
AC-5mg/kg (n=3)	anti-rsPilA (n=2)	3.000	0.083	0.200
AC-10mg/kg (n=1)	Ofloxacin (n=3)	1.800	0.180	0.308
AC-10mg/kg (n=1)	Saline (n=2)	1.500	0.221	0.331
AC-10mg/kg (n=1)	TS-30mg/kg (n=3)	0.200	0.655	0.693
AC-10mg/kg (n=1)	TS-15mg/kg (n=3)	0.200	0.655	0.693
AC-10mg/kg (n=1)	anti-tip-chimer (n=2)	1.500	0.221	0.331
AC-10mg/kg (n=1)	anti-rsPilA (n=2)	1.500	0.221	0.331
Ofloxacin (n=3)	Saline (n=2)	3.000	0.083	0.200
Ofloxacin (n=3)	TS-30mg/kg (n=3)	0.048	0.827	0.851
Ofloxacin (n=3)	TS-15mg/kg (n=3)	2.333	0.127	0.253
Ofloxacin (n=3)	anti-tip-chimer (n=2)	0.333	0.564	0.634
Ofloxacin (n=3)	anti-rsPilA (n=2)	3.000	0.083	0.200
Saline (n=2)	TS-30mg/kg (n=3)	1.333	0.248	0.344
Saline (n=2)	TS-15mg/kg (n=3)	3.000	0.083	0.200
Saline (n=2)	anti-tip-chimer (n=2)	2.400	0.121	0.253
Saline (n=2)	anti-rsPilA (n=2)	0.000	1.000	1.000
TS-30mg/kg (n=3)	TS-15mg/kg (n=3)	0.429	0.513	0.634
TS-30mg/kg (n=3)	anti-tip-chimer (n=2)	0.333	0.564	0.634
TS-30mg/kg (n=3)	anti-rsPilA (n=2)	1.333	0.248	0.344
TS-15mg/kg (n=3)	anti-tip-chimer (n=2)	0.333	0.564	0.634
TS-15mg/kg (n=3)	anti-rsPilA (n=2)	3.000	0.083	0.200
anti-tip-chimer (n=2)	anti-rsPilA (n=2)	2.400	0.121	0.253

Kruskal-Wallis (Omnibus Test)

**H**	15.881
**p-value**	0.044

Supplementary File 3
Alpha Diversity Output

Fecal Evenness-9DPT

Kruskal-Wallis pairwise comparisons

Group 1	Group 2	H	p-value	q-value
AC-PO7d (n=2)	AC-5mg/kg (n=3)	0.333	0.564	0.842
AC-PO7d (n=2)	AC-10mg/kg (n=2)	0.000	1.000	1.000
AC-PO7d (n=2)	Ofloxacin (n=3)	1.333	0.248	0.526
AC-PO7d (n=2)	Saline (n=1)	1.500	0.221	0.526
AC-PO7d (n=2)	TS-30mg/kg (n=3)	0.333	0.564	0.842
AC-PO7d (n=2)	TS-15mg/kg (n=3)	1.333	0.248	0.526
AC-PO7d (n=2)	anti-tip-chimer (n=3)	3.000	0.083	0.456
AC-PO7d (n=2)	anti-rsPilA (n=2)	0.600	0.439	0.831
AC-5mg/kg (n=3)	AC-10mg/kg (n=2)	3.000	0.083	0.456
AC-5mg/kg (n=3)	Ofloxacin (n=3)	1.190	0.275	0.550
AC-5mg/kg (n=3)	Saline (n=1)	0.200	0.655	0.842
AC-5mg/kg (n=3)	TS-30mg/kg (n=3)	0.048	0.827	0.902
AC-5mg/kg (n=3)	TS-15mg/kg (n=3)	0.048	0.827	0.902
AC-5mg/kg (n=3)	anti-tip-chimer (n=3)	2.333	0.127	0.456
AC-5mg/kg (n=3)	anti-rsPilA (n=2)	0.333	0.564	0.842
AC-10mg/kg (n=2)	Ofloxacin (n=3)	3.000	0.083	0.456
AC-10mg/kg (n=2)	Saline (n=1)	1.500	0.221	0.526
AC-10mg/kg (n=2)	TS-30mg/kg (n=3)	0.333	0.564	0.842
AC-10mg/kg (n=2)	TS-15mg/kg (n=3)	3.000	0.083	0.456
AC-10mg/kg (n=2)	anti-tip-chimer (n=3)	3.000	0.083	0.456
AC-10mg/kg (n=2)	anti-rsPilA (n=2)	2.400	0.121	0.456
Ofloxacin (n=3)	Saline (n=1)	0.200	0.655	0.842
Ofloxacin (n=3)	TS-30mg/kg (n=3)	0.048	0.827	0.902
Ofloxacin (n=3)	TS-15mg/kg (n=3)	0.048	0.827	0.902
Ofloxacin (n=3)	anti-tip-chimer (n=3)	2.333	0.127	0.456
Ofloxacin (n=3)	anti-rsPilA (n=2)	0.000	1.000	1.000
Saline (n=1)	TS-30mg/kg (n=3)	0.200	0.655	0.842
Saline (n=1)	TS-15mg/kg (n=3)	1.800	0.180	0.526
Saline (n=1)	anti-tip-chimer (n=3)	0.200	0.655	0.842
Saline (n=1)	anti-rsPilA (n=2)	1.500	0.221	0.526
TS-30mg/kg (n=3)	TS-15mg/kg (n=3)	0.048	0.827	0.902
TS-30mg/kg (n=3)	anti-tip-chimer (n=3)	2.333	0.127	0.456
TS-30mg/kg (n=3)	anti-rsPilA (n=2)	0.333	0.564	0.842
TS-15mg/kg (n=3)	anti-tip-chimer (n=3)	2.333	0.127	0.456
TS-15mg/kg (n=3)	anti-rsPilA (n=2)	0.000	1.000	1.000
anti-tip-chimer (n=3)	anti-rsPilA (n=2)	1.333	0.248	0.526

Kruskal-Wallis (Omnibus Test)

**H**	9.656
p-value	0.290

Supplementary File 3
Alpha Diversity Output

Fecal Evenness--Within-treatment Kruskal-Wallis pairwise comparisons

Treatment	Group 1	Group 2	H	p-value	q-value
AC-PO7d	Baseline (n=3)	2DPT (n=2)	3.000	0.083	0.253
AC-PO7d	Baseline (n=3)	5DPT (n=3)	0.429	0.513	0.564
AC-PO7d	Baseline (n=3)	7DPT (n=3)	3.857	0.050	0.253
AC-PO7d	Baseline (n=3)	9DPT (n=2)	0.333	0.564	0.564
AC-PO7d	2DPT (n=2)	5DPT (n=3)	3.000	0.083	0.253
AC-PO7d	2DPT (n=2)	7DPT (n=3)	0.333	0.564	0.564
AC-PO7d	2DPT (n=2)	9DPT (n=2)	2.400	0.121	0.253
AC-PO7d	5DPT (n=3)	7DPT (n=3)	2.333	0.127	0.253
AC-PO7d	5DPT (n=3)	9DPT (n=2)	1.333	0.248	0.355
AC-PO7d	7DPT (n=3)	9DPT (n=2)	1.333	0.248	0.355

Treatment	Group 1	Group 2	H	p-value	q-value
AC-10mg/kg	Baseline (n=2)	2DPT (n=2)	2.400	0.121	0.243
AC-10mg/kg	Baseline (n=2)	5DPT (n=2)	2.400	0.121	0.243
AC-10mg/kg	Baseline (n=2)	7DPT (n=1)	1.500	0.221	0.245
AC-10mg/kg	Baseline (n=2)	9DPT (n=2)	2.400	0.121	0.243
AC-10mg/kg	2DPT (n=2)	5DPT (n=2)	0.600	0.439	0.439
AC-10mg/kg	2DPT (n=2)	7DPT (n=1)	1.500	0.221	0.245
AC-10mg/kg	2DPT (n=2)	9DPT (n=2)	2.400	0.121	0.243
AC-10mg/kg	5DPT (n=2)	7DPT (n=1)	1.500	0.221	0.245
AC-10mg/kg	5DPT (n=2)	9DPT (n=2)	2.400	0.121	0.243
AC-10mg/kg	7DPT (n=1)	9DPT (n=2)	1.500	0.221	0.245

Treatment	Group 1	Group 2	H	p-value	q-value
AC-5mg/kg	Baseline (n=2)	2DPT (n=2)	0.600	0.439	0.705
AC-5mg/kg	Baseline (n=2)	5DPT (n=3)	0.333	0.564	0.705
AC-5mg/kg	Baseline (n=2)	7DPT (n=3)	0.000	1.000	1.000
AC-5mg/kg	Baseline (n=2)	9DPT (n=3)	0.333	0.564	0.705
AC-5mg/kg	2DPT (n=2)	5DPT (n=3)	1.333	0.248	0.705
AC-5mg/kg	2DPT (n=2)	7DPT (n=3)	0.000	1.000	1.000
AC-5mg/kg	2DPT (n=2)	9DPT (n=3)	0.333	0.564	0.705
AC-5mg/kg	5DPT (n=3)	7DPT (n=3)	0.429	0.513	0.705
AC-5mg/kg	5DPT (n=3)	9DPT (n=3)	1.190	0.275	0.705
AC-5mg/kg	7DPT (n=3)	9DPT (n=3)	0.429	0.513	0.705

Supplementary File 3
Alpha Diversity Output

Fecal Evenness--Within-treatment Kruskal-Wallis pairwise comparisons

Treatment	Group 1	Group 2	H	p-value	q-value
anti-rsPilA	Baseline (n=3)	2DPT (n=3)	3.857	0.050	0.208
anti-rsPilA	Baseline (n=3)	5DPT (n=2)	3.000	0.083	0.208
anti-rsPilA	Baseline (n=3)	7DPT (n=2)	0.000	1.000	1.000
anti-rsPilA	Baseline (n=3)	9DPT (n=2)	3.000	0.083	0.208
anti-rsPilA	2DPT (n=3)	5DPT (n=2)	0.333	0.564	0.626
anti-rsPilA	2DPT (n=3)	7DPT (n=2)	3.000	0.083	0.208
anti-rsPilA	2DPT (n=3)	9DPT (n=2)	0.333	0.564	0.626
anti-rsPilA	5DPT (n=2)	7DPT (n=2)	0.600	0.439	0.626
anti-rsPilA	5DPT (n=2)	9DPT (n=2)	0.600	0.439	0.626
anti-rsPilA	7DPT (n=2)	9DPT (n=2)	2.400	0.121	0.243

Treatment	Group 1	Group 2	H	p-value	q-value
anti-tip-chimer	Baseline (n=3)	2DPT (n=3)	0.048	0.827	0.827
anti-tip-chimer	Baseline (n=3)	5DPT (n=3)	0.048	0.827	0.827
anti-tip-chimer	Baseline (n=3)	7DPT (n=2)	0.333	0.564	0.805
anti-tip-chimer	Baseline (n=3)	9DPT (n=3)	2.333	0.127	0.317
anti-tip-chimer	2DPT (n=3)	5DPT (n=3)	0.048	0.827	0.827
anti-tip-chimer	2DPT (n=3)	7DPT (n=2)	0.333	0.564	0.805
anti-tip-chimer	2DPT (n=3)	9DPT (n=3)	2.333	0.127	0.317
anti-tip-chimer	5DPT (n=3)	7DPT (n=2)	0.333	0.564	0.805
anti-tip-chimer	5DPT (n=3)	9DPT (n=3)	2.333	0.127	0.317
anti-tip-chimer	7DPT (n=2)	9DPT (n=3)	3.000	0.083	0.317

Treatment	Group 1	Group 2	H	p-value	q-value
Ofloxacin	Baseline (n=3)	2DPT (n=2)	0.333	0.564	0.827
Ofloxacin	Baseline (n=3)	5DPT (n=3)	0.048	0.827	0.827
Ofloxacin	Baseline (n=3)	7DPT (n=3)	0.429	0.513	0.827
Ofloxacin	Baseline (n=3)	9DPT (n=3)	0.048	0.827	0.827
Ofloxacin	2DPT (n=2)	5DPT (n=3)	1.333	0.248	0.827
Ofloxacin	2DPT (n=2)	7DPT (n=3)	0.333	0.564	0.827
Ofloxacin	2DPT (n=2)	9DPT (n=3)	3.000	0.083	0.827
Ofloxacin	5DPT (n=3)	7DPT (n=3)	1.190	0.275	0.827
Ofloxacin	5DPT (n=3)	9DPT (n=3)	0.048	0.827	0.827
Ofloxacin	7DPT (n=3)	9DPT (n=3)	0.048	0.827	0.827

Supplementary File 3
Alpha Diversity Output

Fecal Evenness--Within-treatment Kruskal-Wallis pairwise comparisons

Treatment	Group 1	Group 2	H	p-value	q-value
TS-30mg/kg	Baseline (n=3)	2DPT (n=3)	1.190	0.275	0.688
TS-30mg/kg	Baseline (n=3)	5DPT (n=3)	1.190	0.275	0.688
TS-30mg/kg	Baseline (n=3)	7DPT (n=3)	2.333	0.127	0.633
TS-30mg/kg	Baseline (n=3)	9DPT (n=3)	2.333	0.127	0.633
TS-30mg/kg	2DPT (n=3)	5DPT (n=3)	0.048	0.827	0.827
TS-30mg/kg	2DPT (n=3)	7DPT (n=3)	0.048	0.827	0.827
TS-30mg/kg	2DPT (n=3)	9DPT (n=3)	0.048	0.827	0.827
TS-30mg/kg	5DPT (n=3)	7DPT (n=3)	0.048	0.827	0.827
TS-30mg/kg	5DPT (n=3)	9DPT (n=3)	0.048	0.827	0.827
TS-30mg/kg	7DPT (n=3)	9DPT (n=3)	0.429	0.513	0.827

Treatment	Group 1	Group 2	H	p-value	q-value
TS-15mg/kg	Baseline (n=3)	2DPT (n=3)	0.048	0.827	0.827
TS-15mg/kg	Baseline (n=3)	5DPT (n=3)	1.190	0.275	0.459
TS-15mg/kg	Baseline (n=3)	7DPT (n=3)	3.857	0.050	0.248
TS-15mg/kg	Baseline (n=3)	9DPT (n=3)	0.048	0.827	0.827
TS-15mg/kg	2DPT (n=3)	5DPT (n=3)	1.190	0.275	0.459
TS-15mg/kg	2DPT (n=3)	7DPT (n=3)	3.857	0.050	0.248
TS-15mg/kg	2DPT (n=3)	9DPT (n=3)	0.429	0.513	0.732
TS-15mg/kg	5DPT (n=3)	7DPT (n=3)	2.333	0.127	0.317
TS-15mg/kg	5DPT (n=3)	9DPT (n=3)	0.048	0.827	0.827
TS-15mg/kg	7DPT (n=3)	9DPT (n=3)	2.333	0.127	0.317

Treatment	Group 1	Group 2	H	p-value	q-value
Saline	Baseline (n=3)	2DPT (n=3)	0.048	0.827	1.000
Saline	Baseline (n=3)	5DPT (n=3)	0.048	0.827	1.000
Saline	Baseline (n=3)	7DPT (n=2)	0.000	1.000	1.000
Saline	Baseline (n=3)	9DPT (n=1)	0.200	0.655	1.000
Saline	2DPT (n=3)	5DPT (n=3)	0.429	0.513	1.000
Saline	2DPT (n=3)	7DPT (n=2)	0.000	1.000	1.000
Saline	2DPT (n=3)	9DPT (n=1)	0.200	0.655	1.000
Saline	5DPT (n=3)	7DPT (n=2)	1.333	0.248	1.000
Saline	5DPT (n=3)	9DPT (n=1)	0.200	0.655	1.000
Saline	7DPT (n=2)	9DPT (n=1)	1.500	0.221	1.000

Supplementary File 3
Alpha Diversity Output

id	Chinchilla_ID	Treatment	Sex	Collection	Fecal faith_pd
1BF	Chinch1	AC-PO7d	F	Baseline	22.32535641
2BF	Chinch2	AC-PO7d	F	Baseline	23.92247276
3BF	Chinch3	AC-PO7d	M	Baseline	24.86078052
20FBL	Chinch20	AC-5mg/kg	F	Baseline	17.99947962
23FBL	Chinch23	AC-5mg/kg	F	Baseline	16.37684246
22FBL	Chinch22	AC-10mg/kg	M	Baseline	15.49171375
25FBL	Chinch25	AC-10mg/kg	F	Baseline	14.28718361
11FBL	Chinch11	anti-rsPilA	F	Baseline	17.46301741
13FBL	Chinch13	anti-rsPilA	F	Baseline	13.98591063
17FBL	Chinch17	anti-rsPilA	F	Baseline	16.92367952
19FBL	Chinch19	anti-tip-chimer	F	Baseline	18.50800804
21FBL	Chinch21	anti-tip-chimer	F	Baseline	15.44157581
27FBL	Chinch27	anti-tip-chimer	M	Baseline	15.69451771
4FBL	Chinch4	Ofloxacin	F	Baseline	14.57151162
5FBL	Chinch5	Ofloxacin	F	Baseline	14.73117945
7FBL	Chinch7	Ofloxacin	M	Baseline	14.43751812
14FBL	Chinch14	TS-15mg/kg	F	Baseline	15.7345052
15FBL	Chinch15	TS-15mg/kg	F	Baseline	19.04259359
18FBL	Chinch18	TS-15mg/kg	M	Baseline	18.00162152
10FBL	Chinch10	TS-30mg/kg	F	Baseline	15.98975083
12FBL	Chinch12	TS-30mg/kg	M	Baseline	16.36536881
16FBL	Chinch16	TS-30mg/kg	F	Baseline	16.68100351
6FBL	Chinch6	Saline	F	Baseline	14.52927472
8FBL	Chinch8	Saline	M	Baseline	15.08676239
9FBL	Chinch9	Saline	F	Baseline	15.09568967
2AF2	Chinch2	AC-PO7d	F	1	8.280152281
3AF2	Chinch3	AC-PO7d	M	1	9.477758647
20F1	Chinch20	AC-5mg/kg	F	1	12.69421915
23F1	Chinch23	AC-5mg/kg	F	1	11.93618031
22F1	Chinch22	AC-10mg/kg	M	1	9.396228868
24F1	Chinch24	AC-10mg/kg	F	1	11.73382119
11F1	Chinch11	anti-rsPilA	F	1	13.70215775
13F1	Chinch13	anti-rsPilA	F	1	11.44209881
17F1	Chinch17	anti-rsPilA	F	1	16.15868709
19F1	Chinch19	anti-tip-chimer	F	1	14.83912277
21F1	Chinch21	anti-tip-chimer	F	1	16.73944005
27F1	Chinch27	anti-tip-chimer	M	1	13.86275357
4F1	Chinch4	Ofloxacin	F	1	14.0635197
7F1	Chinch7	Ofloxacin	M	1	12.07286598
14F1	Chinch14	TS-15mg/kg	F	1	13.45504183
15F1	Chinch15	TS-15mg/kg	F	1	8.993471535
18F1	Chinch18	TS-15mg/kg	M	1	14.1601301
10F1	Chinch10	TS-30mg/kg	F	1	14.28696028
12F1	Chinch12	TS-30mg/kg	M	1	16.96116157
16F1	Chinch16	TS-30mg/kg	F	1	12.89794717
6F1	Chinch6	Saline	F	1	14.77680688
8F1	Chinch8	Saline	M	1	12.83287509

Supplementary File 3
Alpha Diversity Output

id	Chinchilla_ID	Treatment	Sex	Collection	Fecal faith_pd
9F1	Chinch9	Saline	F	1	11.63884612
1AF5	Chinch1	AC-PO7d	F	2	18.12478027
2AF5	Chinch2	AC-PO7d	F	2	10.16479968
3AF5	Chinch3	AC-PO7d	M	2	11.44840796
20F2	Chinch20	AC-5mg/kg	F	2	9.563934684
23F2	Chinch23	AC-5mg/kg	F	2	7.463796748
26F2	Chinch26	AC-5mg/kg	M	2	10.13928843
22F2	Chinch22	AC-10mg/kg	M	2	7.8114579
25F2	Chinch25	AC-10mg/kg	F	2	9.974426981
11F2	Chinch11	anti-rsPilA	F	2	16.16179181
17F2	Chinch17	anti-rsPilA	F	2	14.38409286
19F2	Chinch19	anti-tip-chimer	F	2	13.50128202
21F2	Chinch21	anti-tip-chimer	F	2	16.03759751
27F2	Chinch27	anti-tip-chimer	M	2	14.75653477
4F2	Chinch4	Ofloxacin	F	2	13.55945827
5F2	Chinch5	Ofloxacin	F	2	15.76175099
7F2	Chinch7	Ofloxacin	M	2	12.94191374
14F2	Chinch14	TS-15mg/kg	F	2	13.69467071
15F2	Chinch15	TS-15mg/kg	F	2	10.19640123
18F2	Chinch18	TS-15mg/kg	M	2	16.61829224
10F2	Chinch10	TS-30mg/kg	F	2	13.98167672
12F2	Chinch12	TS-30mg/kg	M	2	17.74262596
16F2	Chinch16	TS-30mg/kg	F	2	13.37195113
6F2	Chinch6	Saline	F	2	17.25635752
8F2	Chinch8	Saline	M	2	13.35437815
9F2	Chinch9	Saline	F	2	13.88355122
1AF7	Chinch1	AC-PO7d	F	3	7.660046683
2AF7	Chinch2	AC-PO7d	F	3	11.83607167
3AF7	Chinch3	AC-PO7d	M	3	10.10375329
20F3	Chinch20	AC-5mg/kg	F	3	13.21013779
23F3	Chinch23	AC-5mg/kg	F	3	10.4390606
26F3	Chinch26	AC-5mg/kg	M	3	12.13990851
25F3	Chinch25	AC-10mg/kg	F	3	12.55195604
11F3	Chinch11	anti-rsPilA	F	3	16.10294333
17F3	Chinch17	anti-rsPilA	F	3	16.12138159
19F3	Chinch19	anti-tip-chimer	F	3	14.22000674
21F3	Chinch21	anti-tip-chimer	F	3	18.31297857
4F3	Chinch4	Ofloxacin	F	3	12.02582071
5F3	Chinch5	Ofloxacin	F	3	16.18245136
7F3	Chinch7	Ofloxacin	M	3	12.94660104
14F3	Chinch14	TS-15mg/kg	F	3	12.39947261
15F3	Chinch15	TS-15mg/kg	F	3	12.0179004
18F3	Chinch18	TS-15mg/kg	M	3	11.49181145
10F3	Chinch10	TS-30mg/kg	F	3	16.14756707
12F3	Chinch12	TS-30mg/kg	M	3	16.32306211
16F3	Chinch16	TS-30mg/kg	F	3	14.78414646
6F3	Chinch6	Saline	F	3	14.24939287
8F3	Chinch8	Saline	M	3	12.63640903

Supplementary File 3
Alpha Diversity Output

id	Chinchilla_ID	Treatment	Sex	Collection	Fecal faith_pd
2AF9	Chinch2	AC-PO7d	F	4	12.92198787
3AF9	Chinch3	AC-PO7d	M	4	14.32680647
20F4	Chinch20	AC-5mg/kg	F	4	12.40321726
23F4	Chinch23	AC-5mg/kg	F	4	14.68532751
26F4	Chinch26	AC-5mg/kg	M	4	10.40434235
22F4	Chinch22	AC-10mg/kg	M	4	12.8148818
25F4	Chinch25	AC-10mg/kg	F	4	11.20756631
11F4	Chinch11	anti-rsPilA	F	4	16.73722725
17F4	Chinch17	anti-rsPilA	F	4	15.05536912
19F4	Chinch19	anti-tip-chimer	F	4	15.70932954
21F4	Chinch21	anti-tip-chimer	F	4	17.21326697
27F4	Chinch27	anti-tip-chimer	M	4	15.30042086
4F4	Chinch4	Ofloxacin	F	4	12.22838007
5F4	Chinch5	Ofloxacin	F	4	13.86646854
7F4	Chinch7	Ofloxacin	M	4	13.34241807
14F4	Chinch14	TS-15mg/kg	F	4	14.30414878
15F4	Chinch15	TS-15mg/kg	F	4	12.70014397
18F4	Chinch18	TS-15mg/kg	M	4	17.33462346
10F4	Chinch10	TS-30mg/kg	F	4	14.21735582
12F4	Chinch12	TS-30mg/kg	M	4	16.94498589
16F4	Chinch16	TS-30mg/kg	F	4	16.90582697
8F4	Chinch8	Saline	M	4	13.51454072

Supplementary File 3
Alpha Diversity Output

Fecal FaithPD-Baseline

Kruskal-Wallis pairwise comparisons

Group 1	Group 2	H	p-value	q-value
AC-PO7d (n=3)	AC-5mg/kg (n=2)	3.000	0.083	0.167
AC-PO7d (n=3)	AC-10mg/kg (n=2)	3.000	0.083	0.167
AC-PO7d (n=3)	Ofloxacin (n=3)	3.857	0.050	0.149
AC-PO7d (n=3)	Saline (n=3)	3.857	0.050	0.149
AC-PO7d (n=3)	TS-30mg/kg (n=3)	3.857	0.050	0.149
AC-PO7d (n=3)	TS-15mg/kg (n=3)	3.857	0.050	0.149
AC-PO7d (n=3)	anti-tip-chimer (n=3)	3.857	0.050	0.149
AC-PO7d (n=3)	anti-rsPilA (n=3)	3.857	0.050	0.149
AC-5mg/kg (n=2)	AC-10mg/kg (n=2)	2.400	0.121	0.230
AC-5mg/kg (n=2)	Ofloxacin (n=3)	3.000	0.083	0.167
AC-5mg/kg (n=2)	Saline (n=3)	3.000	0.083	0.167
AC-5mg/kg (n=2)	TS-30mg/kg (n=3)	1.333	0.248	0.413
AC-5mg/kg (n=2)	TS-15mg/kg (n=3)	0.333	0.564	0.615
AC-5mg/kg (n=2)	anti-tip-chimer (n=3)	0.333	0.564	0.615
AC-5mg/kg (n=2)	anti-rsPilA (n=3)	0.333	0.564	0.615
AC-10mg/kg (n=2)	Ofloxacin (n=3)	0.000	1.000	1.000
AC-10mg/kg (n=2)	Saline (n=3)	0.000	1.000	1.000
AC-10mg/kg (n=2)	TS-30mg/kg (n=3)	3.000	0.083	0.167
AC-10mg/kg (n=2)	TS-15mg/kg (n=3)	3.000	0.083	0.167
AC-10mg/kg (n=2)	anti-tip-chimer (n=3)	1.333	0.248	0.413
AC-10mg/kg (n=2)	anti-rsPilA (n=3)	0.333	0.564	0.615
Ofloxacin (n=3)	Saline (n=3)	1.190	0.275	0.413
Ofloxacin (n=3)	TS-30mg/kg (n=3)	3.857	0.050	0.149
Ofloxacin (n=3)	TS-15mg/kg (n=3)	3.857	0.050	0.149
Ofloxacin (n=3)	anti-tip-chimer (n=3)	3.857	0.050	0.149
Ofloxacin (n=3)	anti-rsPilA (n=3)	0.429	0.513	0.615
Saline (n=3)	TS-30mg/kg (n=3)	3.857	0.050	0.149
Saline (n=3)	TS-15mg/kg (n=3)	3.857	0.050	0.149
Saline (n=3)	anti-tip-chimer (n=3)	3.857	0.050	0.149
Saline (n=3)	anti-rsPilA (n=3)	0.429	0.513	0.615
TS-30mg/kg (n=3)	TS-15mg/kg (n=3)	0.429	0.513	0.615
TS-30mg/kg (n=3)	anti-tip-chimer (n=3)	0.429	0.513	0.615
TS-30mg/kg (n=3)	anti-rsPilA (n=3)	0.429	0.513	0.615
TS-15mg/kg (n=3)	anti-tip-chimer (n=3)	1.190	0.275	0.413
TS-15mg/kg (n=3)	anti-rsPilA (n=3)	1.190	0.275	0.413
anti-tip-chimer (n=3)	anti-rsPilA (n=3)	0.048	0.827	0.876

Kruskal-Wallis (Omnibus Test)

**H**	16.972
**p-value**	0.030

Supplementary File 3
Alpha Diversity Output

Fecal FaithPD-2DPT

Kruskal-Wallis pairwise comparisons

Group 1	Group 2	H	p-value	q-value
AC-PO7d (n=2)	AC-5mg/kg (n=2)	2.400	0.121	0.326
AC-PO7d (n=2)	AC-10mg/kg (n=2)	0.600	0.439	0.658
AC-PO7d (n=2)	Ofloxacin (n=2)	2.400	0.121	0.326
AC-PO7d (n=2)	Saline (n=3)	3.000	0.083	0.326
AC-PO7d (n=2)	TS-30mg/kg (n=3)	3.000	0.083	0.326
AC-PO7d (n=2)	TS-15mg/kg (n=3)	1.333	0.248	0.450
AC-PO7d (n=2)	anti-tip-chimer (n=3)	3.000	0.083	0.326
AC-PO7d (n=2)	anti-rsPilA (n=3)	3.000	0.083	0.326
AC-5mg/kg (n=2)	AC-10mg/kg (n=2)	2.400	0.121	0.326
AC-5mg/kg (n=2)	Ofloxacin (n=2)	0.600	0.439	0.658
AC-5mg/kg (n=2)	Saline (n=3)	0.333	0.564	0.676
AC-5mg/kg (n=2)	TS-30mg/kg (n=3)	3.000	0.083	0.326
AC-5mg/kg (n=2)	TS-15mg/kg (n=3)	0.333	0.564	0.676
AC-5mg/kg (n=2)	anti-tip-chimer (n=3)	3.000	0.083	0.326
AC-5mg/kg (n=2)	anti-rsPilA (n=3)	0.333	0.564	0.676
AC-10mg/kg (n=2)	Ofloxacin (n=2)	2.400	0.121	0.326
AC-10mg/kg (n=2)	Saline (n=3)	1.333	0.248	0.450
AC-10mg/kg (n=2)	TS-30mg/kg (n=3)	3.000	0.083	0.326
AC-10mg/kg (n=2)	TS-15mg/kg (n=3)	0.333	0.564	0.676
AC-10mg/kg (n=2)	anti-tip-chimer (n=3)	3.000	0.083	0.326
AC-10mg/kg (n=2)	anti-rsPilA (n=3)	1.333	0.248	0.450
Ofloxacin (n=2)	Saline (n=3)	0.000	1.000	1.000
Ofloxacin (n=2)	TS-30mg/kg (n=3)	1.333	0.248	0.450
Ofloxacin (n=2)	TS-15mg/kg (n=3)	0.000	1.000	1.000
Ofloxacin (n=2)	anti-tip-chimer (n=3)	1.333	0.248	0.450
Ofloxacin (n=2)	anti-rsPilA (n=3)	0.000	1.000	1.000
Saline (n=3)	TS-30mg/kg (n=3)	1.190	0.275	0.450
Saline (n=3)	TS-15mg/kg (n=3)	0.048	0.827	0.902
Saline (n=3)	anti-tip-chimer (n=3)	2.333	0.127	0.326
Saline (n=3)	anti-rsPilA (n=3)	0.048	0.827	0.902
TS-30mg/kg (n=3)	TS-15mg/kg (n=3)	1.190	0.275	0.450
TS-30mg/kg (n=3)	anti-tip-chimer (n=3)	0.048	0.827	0.902
TS-30mg/kg (n=3)	anti-rsPilA (n=3)	0.429	0.513	0.676
TS-15mg/kg (n=3)	anti-tip-chimer (n=3)	2.333	0.127	0.326
TS-15mg/kg (n=3)	anti-rsPilA (n=3)	0.429	0.513	0.676
anti-tip-chimer (n=3)	anti-rsPilA (n=3)	1.190	0.275	0.450

Kruskal-Wallis (Omnibus Test)

**H**	11.978
**p-value**	0.152

Supplementary File 3
Alpha Diversity Output

Fecal FaithPD-5DPT

Kruskal-Wallis pairwise comparisons

Group 1	Group 2	H	p-value	q-value
AC-PO7d (n=3)	AC-5mg/kg (n=3)	3.857	0.050	0.231
AC-PO7d (n=3)	AC-10mg/kg (n=2)	3.000	0.083	0.231
AC-PO7d (n=3)	Ofloxacin (n=3)	0.429	0.513	0.676
AC-PO7d (n=3)	Saline (n=3)	0.429	0.513	0.676
AC-PO7d (n=3)	TS-30mg/kg (n=3)	0.429	0.513	0.676
AC-PO7d (n=3)	TS-15mg/kg (n=3)	0.048	0.827	0.851
AC-PO7d (n=3)	anti-tip-chimer (n=3)	0.429	0.513	0.676
AC-PO7d (n=3)	anti-rsPilA (n=2)	0.333	0.564	0.676
AC-5mg/kg (n=3)	AC-10mg/kg (n=2)	0.000	1.000	1.000
AC-5mg/kg (n=3)	Ofloxacin (n=3)	3.857	0.050	0.231
AC-5mg/kg (n=3)	Saline (n=3)	3.857	0.050	0.231
AC-5mg/kg (n=3)	TS-30mg/kg (n=3)	3.857	0.050	0.231
AC-5mg/kg (n=3)	TS-15mg/kg (n=3)	3.857	0.050	0.231
AC-5mg/kg (n=3)	anti-tip-chimer (n=3)	3.857	0.050	0.231
AC-5mg/kg (n=3)	anti-rsPilA (n=2)	3.000	0.083	0.231
AC-10mg/kg (n=2)	Ofloxacin (n=3)	3.000	0.083	0.231
AC-10mg/kg (n=2)	Saline (n=3)	3.000	0.083	0.231
AC-10mg/kg (n=2)	TS-30mg/kg (n=3)	3.000	0.083	0.231
AC-10mg/kg (n=2)	TS-15mg/kg (n=3)	3.000	0.083	0.231
AC-10mg/kg (n=2)	anti-tip-chimer (n=3)	3.000	0.083	0.231
AC-10mg/kg (n=2)	anti-rsPilA (n=2)	2.400	0.121	0.312
Ofloxacin (n=3)	Saline (n=3)	0.429	0.513	0.676
Ofloxacin (n=3)	TS-30mg/kg (n=3)	0.429	0.513	0.676
Ofloxacin (n=3)	TS-15mg/kg (n=3)	0.048	0.827	0.851
Ofloxacin (n=3)	anti-tip-chimer (n=3)	0.429	0.513	0.676
Ofloxacin (n=3)	anti-rsPilA (n=2)	1.333	0.248	0.596
Saline (n=3)	TS-30mg/kg (n=3)	0.429	0.513	0.676
Saline (n=3)	TS-15mg/kg (n=3)	0.429	0.513	0.676
Saline (n=3)	anti-tip-chimer (n=3)	0.048	0.827	0.851
Saline (n=3)	anti-rsPilA (n=2)	0.333	0.564	0.676
TS-30mg/kg (n=3)	TS-15mg/kg (n=3)	0.429	0.513	0.676
TS-30mg/kg (n=3)	anti-tip-chimer (n=3)	0.048	0.827	0.851
TS-30mg/kg (n=3)	anti-rsPilA (n=2)	0.333	0.564	0.676
TS-15mg/kg (n=3)	anti-tip-chimer (n=3)	0.048	0.827	0.851
TS-15mg/kg (n=3)	anti-rsPilA (n=2)	0.333	0.564	0.676
anti-tip-chimer (n=3)	anti-rsPilA (n=2)	0.333	0.564	0.676

**Kruskal-Wallis (Omnibus Test)**	
**H**	12.812
**p-value**	0.118

Supplementary File 3
Alpha Diversity Output

Fecal FaithPD-7DPT

Kruskal-Wallis pairwise comparisons

Group 1	Group 2	H	p-value	q-value
AC-PO7d (n=3)	AC-5mg/kg (n=3)	2.333	0.127	0.268
AC-PO7d (n=3)	AC-10mg/kg (n=1)	1.800	0.180	0.323
AC-PO7d (n=3)	Ofloxacin (n=3)	3.857	0.050	0.231
AC-PO7d (n=3)	Saline (n=2)	3.000	0.083	0.231
AC-PO7d (n=3)	TS-30mg/kg (n=3)	3.857	0.050	0.231
AC-PO7d (n=3)	TS-15mg/kg (n=3)	2.333	0.127	0.268
AC-PO7d (n=3)	anti-tip-chimer (n=2)	3.000	0.083	0.231
AC-PO7d (n=3)	anti-rsPilA (n=2)	3.000	0.083	0.231
AC-5mg/kg (n=3)	AC-10mg/kg (n=1)	0.200	0.655	0.737
AC-5mg/kg (n=3)	Ofloxacin (n=3)	0.429	0.513	0.659
AC-5mg/kg (n=3)	Saline (n=2)	1.333	0.248	0.357
AC-5mg/kg (n=3)	TS-30mg/kg (n=3)	3.857	0.050	0.231
AC-5mg/kg (n=3)	TS-15mg/kg (n=3)	0.048	0.827	0.902
AC-5mg/kg (n=3)	anti-tip-chimer (n=2)	3.000	0.083	0.231
AC-5mg/kg (n=3)	anti-rsPilA (n=2)	3.000	0.083	0.231
AC-10mg/kg (n=1)	Ofloxacin (n=3)	0.200	0.655	0.737
AC-10mg/kg (n=1)	Saline (n=2)	1.500	0.221	0.345
AC-10mg/kg (n=1)	TS-30mg/kg (n=3)	1.800	0.180	0.323
AC-10mg/kg (n=1)	TS-15mg/kg (n=3)	1.800	0.180	0.323
AC-10mg/kg (n=1)	anti-tip-chimer (n=2)	1.500	0.221	0.345
AC-10mg/kg (n=1)	anti-rsPilA (n=2)	1.500	0.221	0.345
Ofloxacin (n=3)	Saline (n=2)	0.000	1.000	1.000
Ofloxacin (n=3)	TS-30mg/kg (n=3)	1.190	0.275	0.381
Ofloxacin (n=3)	TS-15mg/kg (n=3)	2.333	0.127	0.268
Ofloxacin (n=3)	anti-tip-chimer (n=2)	1.333	0.248	0.357
Ofloxacin (n=3)	anti-rsPilA (n=2)	0.333	0.564	0.676
Saline (n=2)	TS-30mg/kg (n=3)	3.000	0.083	0.231
Saline (n=2)	TS-15mg/kg (n=3)	3.000	0.083	0.231
Saline (n=2)	anti-tip-chimer (n=2)	0.600	0.439	0.585
Saline (n=2)	anti-rsPilA (n=2)	2.400	0.121	0.268
TS-30mg/kg (n=3)	TS-15mg/kg (n=3)	3.857	0.050	0.231
TS-30mg/kg (n=3)	anti-tip-chimer (n=2)	0.000	1.000	1.000
TS-30mg/kg (n=3)	anti-rsPilA (n=2)	0.333	0.564	0.676
TS-15mg/kg (n=3)	anti-tip-chimer (n=2)	3.000	0.083	0.231
TS-15mg/kg (n=3)	anti-rsPilA (n=2)	3.000	0.083	0.231
anti-tip-chimer (n=2)	anti-rsPilA (n=2)	0.000	1.000	1.000

Kruskal-Wallis (Omnibus Test)

**H**	16.008
**p-value**	0.042

Supplementary File 3
Alpha Diversity Output

Fecal FaithPD-9DPT

Kruskal-Wallis pairwise comparisons

Group 1	Group 2	H	p-value	q-value
AC-PO7d (n=2)	AC-5mg/kg (n=3)	0.333	0.564	0.752
AC-PO7d (n=2)	AC-10mg/kg (n=2)	2.400	0.121	0.380
AC-PO7d (n=2)	Ofloxacin (n=3)	0.333	0.564	0.752
AC-PO7d (n=2)	Saline (n=1)	0.000	1.000	1.000
AC-PO7d (n=2)	TS-30mg/kg (n=3)	1.333	0.248	0.470
AC-PO7d (n=2)	TS-15mg/kg (n=3)	0.000	1.000	1.000
AC-PO7d (n=2)	anti-tip-chimer (n=3)	3.000	0.083	0.375
AC-PO7d (n=2)	anti-rsPilA (n=2)	2.400	0.121	0.380
AC-5mg/kg (n=3)	AC-10mg/kg (n=2)	0.000	1.000	1.000
AC-5mg/kg (n=3)	Ofloxacin (n=3)	0.048	0.827	0.902
AC-5mg/kg (n=3)	Saline (n=1)	0.200	0.655	0.786
AC-5mg/kg (n=3)	TS-30mg/kg (n=3)	2.333	0.127	0.380
AC-5mg/kg (n=3)	TS-15mg/kg (n=3)	1.190	0.275	0.472
AC-5mg/kg (n=3)	anti-tip-chimer (n=3)	3.857	0.050	0.375
AC-5mg/kg (n=3)	anti-rsPilA (n=2)	3.000	0.083	0.375
AC-10mg/kg (n=2)	Ofloxacin (n=3)	1.333	0.248	0.470
AC-10mg/kg (n=2)	Saline (n=1)	1.500	0.221	0.470
AC-10mg/kg (n=2)	TS-30mg/kg (n=3)	3.000	0.083	0.375
AC-10mg/kg (n=2)	TS-15mg/kg (n=3)	1.333	0.248	0.470
AC-10mg/kg (n=2)	anti-tip-chimer (n=3)	3.000	0.083	0.375
AC-10mg/kg (n=2)	anti-rsPilA (n=2)	2.400	0.121	0.380
Ofloxacin (n=3)	Saline (n=1)	0.200	0.655	0.786
Ofloxacin (n=3)	TS-30mg/kg (n=3)	3.857	0.050	0.375
Ofloxacin (n=3)	TS-15mg/kg (n=3)	1.190	0.275	0.472
Ofloxacin (n=3)	anti-tip-chimer (n=3)	3.857	0.050	0.375
Ofloxacin (n=3)	anti-rsPilA (n=2)	3.000	0.083	0.375
Saline (n=1)	TS-30mg/kg (n=3)	1.800	0.180	0.462
Saline (n=1)	TS-15mg/kg (n=3)	0.200	0.655	0.786
Saline (n=1)	anti-tip-chimer (n=3)	1.800	0.180	0.462
Saline (n=1)	anti-rsPilA (n=2)	1.500	0.221	0.470
TS-30mg/kg (n=3)	TS-15mg/kg (n=3)	0.048	0.827	0.902
TS-30mg/kg (n=3)	anti-tip-chimer (n=3)	0.048	0.827	0.902
TS-30mg/kg (n=3)	anti-rsPilA (n=2)	0.333	0.564	0.752
TS-15mg/kg (n=3)	anti-tip-chimer (n=3)	0.429	0.513	0.752
TS-15mg/kg (n=3)	anti-rsPilA (n=2)	0.333	0.564	0.752
anti-tip-chimer (n=3)	anti-rsPilA (n=2)	0.333	0.564	0.752

Kruskal-Wallis (Omnibus Test)

**H**	12.514
p-value	0.130

Supplementary File 3
Alpha Diversity Output

Fecal FaithPD--Within-treatment Kruskal-Wallis pairwise comparisons

Treatment	Group 1	Group 2	H	p-value	q-value
AC-PO7d	Baseline (n=3)	2DPT (n=2)	3.000	0.083	0.139
AC-PO7d	Baseline (n=3)	5DPT (n=3)	3.857	0.050	0.139
AC-PO7d	Baseline (n=3)	7DPT (n=3)	3.857	0.050	0.139
AC-PO7d	Baseline (n=3)	9DPT (n=2)	3.000	0.083	0.139
AC-PO7d	2DPT (n=2)	5DPT (n=3)	3.000	0.083	0.139
AC-PO7d	2DPT (n=2)	7DPT (n=3)	0.333	0.564	0.564
AC-PO7d	2DPT (n=2)	9DPT (n=2)	2.400	0.121	0.173
AC-PO7d	5DPT (n=3)	7DPT (n=3)	1.190	0.275	0.344
AC-PO7d	5DPT (n=3)	9DPT (n=2)	0.333	0.564	0.564
AC-PO7d	7DPT (n=3)	9DPT (n=2)	3.000	0.083	0.139

Treatment	Group 1	Group 2	H	p-value	q-value
AC-10mg/kg	Baseline (n=2)	2DPT (n=2)	2.400	0.121	0.303
AC-10mg/kg	Baseline (n=2)	5DPT (n=2)	2.400	0.121	0.303
AC-10mg/kg	Baseline (n=2)	7DPT (n=1)	1.500	0.221	0.315
AC-10mg/kg	Baseline (n=2)	9DPT (n=2)	2.400	0.121	0.303
AC-10mg/kg	2DPT (n=2)	5DPT (n=2)	0.600	0.439	0.487
AC-10mg/kg	2DPT (n=2)	7DPT (n=1)	1.500	0.221	0.315
AC-10mg/kg	2DPT (n=2)	9DPT (n=2)	0.600	0.439	0.487
AC-10mg/kg	5DPT (n=2)	7DPT (n=1)	1.500	0.221	0.315
AC-10mg/kg	5DPT (n=2)	9DPT (n=2)	2.400	0.121	0.303
AC-10mg/kg	7DPT (n=1)	9DPT (n=2)	0.000	1.000	1.000

Treatment	Group 1	Group 2	H	p-value	q-value
AC-5mg/kg	Baseline (n=2)	2DPT (n=2)	2.400	0.121	0.173
AC-5mg/kg	Baseline (n=2)	5DPT (n=3)	3.000	0.083	0.139
AC-5mg/kg	Baseline (n=2)	7DPT (n=3)	3.000	0.083	0.139
AC-5mg/kg	Baseline (n=2)	9DPT (n=3)	3.000	0.083	0.139
AC-5mg/kg	2DPT (n=2)	5DPT (n=3)	3.000	0.083	0.139
AC-5mg/kg	2DPT (n=2)	7DPT (n=3)	0.000	1.000	1.000
AC-5mg/kg	2DPT (n=2)	9DPT (n=3)	0.000	1.000	1.000
AC-5mg/kg	5DPT (n=3)	7DPT (n=3)	3.857	0.050	0.139
AC-5mg/kg	5DPT (n=3)	9DPT (n=3)	3.857	0.050	0.139
AC-5mg/kg	7DPT (n=3)	9DPT (n=3)	0.048	0.827	1.000

Supplementary File 3
Alpha Diversity Output

Fecal FaithPD--Within-treatment Kruskal-Wallis pairwise comparisons

Treatment	Group 1	Group 2	H	p-value	q-value
anti-rsPilA	Baseline (n=3)	2DPT (n=3)	2.333	0.127	0.705
anti-rsPilA	Baseline (n=3)	5DPT (n=2)	0.333	0.564	0.705
anti-rsPilA	Baseline (n=3)	7DPT (n=2)	0.333	0.564	0.705
anti-rsPilA	Baseline (n=3)	9DPT (n=2)	0.333	0.564	0.705
anti-rsPilA	2DPT (n=3)	5DPT (n=2)	1.333	0.248	0.705
anti-rsPilA	2DPT (n=3)	7DPT (n=2)	0.333	0.564	0.705
anti-rsPilA	2DPT (n=3)	9DPT (n=2)	1.333	0.248	0.705
anti-rsPilA	5DPT (n=2)	7DPT (n=2)	0.000	1.000	1.000
anti-rsPilA	5DPT (n=2)	9DPT (n=2)	0.600	0.439	0.705
anti-rsPilA	7DPT (n=2)	9DPT (n=2)	0.000	1.000	1.000

Treatment	Group 1	Group 2	H	p-value	q-value
anti-tip-chimer	Baseline (n=3)	2DPT (n=3)	1.190	0.275	0.688
anti-tip-chimer	Baseline (n=3)	5DPT (n=3)	1.190	0.275	0.688
anti-tip-chimer	Baseline (n=3)	7DPT (n=2)	0.333	0.564	0.705
anti-tip-chimer	Baseline (n=3)	9DPT (n=3)	0.048	0.827	0.919
anti-tip-chimer	2DPT (n=3)	5DPT (n=3)	0.429	0.513	0.705
anti-tip-chimer	2DPT (n=3)	7DPT (n=2)	0.333	0.564	0.705
anti-tip-chimer	2DPT (n=3)	9DPT (n=3)	1.190	0.275	0.688
anti-tip-chimer	5DPT (n=3)	7DPT (n=2)	0.333	0.564	0.705
anti-tip-chimer	5DPT (n=3)	9DPT (n=3)	1.190	0.275	0.688
anti-tip-chimer	7DPT (n=2)	9DPT (n=3)	0.000	1.000	1.000

Treatment	Group 1	Group 2	H	p-value	q-value
Ofloxacin	Baseline (n=3)	2DPT (n=2)	3.000	0.083	0.416
Ofloxacin	Baseline (n=3)	5DPT (n=3)	0.429	0.513	0.940
Ofloxacin	Baseline (n=3)	7DPT (n=3)	0.429	0.513	0.940
Ofloxacin	Baseline (n=3)	9DPT (n=3)	3.857	0.050	0.416
Ofloxacin	2DPT (n=2)	5DPT (n=3)	0.333	0.564	0.940
Ofloxacin	2DPT (n=2)	7DPT (n=3)	0.000	1.000	1.000
Ofloxacin	2DPT (n=2)	9DPT (n=3)	0.000	1.000	1.000
Ofloxacin	5DPT (n=3)	7DPT (n=3)	0.048	0.827	1.000
Ofloxacin	5DPT (n=3)	9DPT (n=3)	0.429	0.513	0.940
Ofloxacin	7DPT (n=3)	9DPT (n=3)	0.048	0.827	1.000

Supplementary File 3
Alpha Diversity Output

Fecal FaithPD--Within-treatment Kruskal-Wallis pairwise comparisons

Treatment	Group 1	Group 2	H	p-value	q-value
TS-30mg/kg	Baseline (n=3)	2DPT (n=3)	0.429	0.513	0.641
TS-30mg/kg	Baseline (n=3)	5DPT (n=3)	0.429	0.513	0.641
TS-30mg/kg	Baseline (n=3)	7DPT (n=3)	1.190	0.275	0.641
TS-30mg/kg	Baseline (n=3)	9DPT (n=3)	0.429	0.513	0.641
TS-30mg/kg	2DPT (n=3)	5DPT (n=3)	0.048	0.827	0.827
TS-30mg/kg	2DPT (n=3)	7DPT (n=3)	0.429	0.513	0.641
TS-30mg/kg	2DPT (n=3)	9DPT (n=3)	0.048	0.827	0.827
TS-30mg/kg	5DPT (n=3)	7DPT (n=3)	0.429	0.513	0.641
TS-30mg/kg	5DPT (n=3)	9DPT (n=3)	0.429	0.513	0.641
TS-30mg/kg	7DPT (n=3)	9DPT (n=3)	0.429	0.513	0.641

Treatment	Group 1	Group 2	H	p-value	q-value
TS-15mg/kg	Baseline (n=3)	2DPT (n=3)	3.857	0.050	0.165
TS-15mg/kg	Baseline (n=3)	5DPT (n=3)	2.333	0.127	0.253
TS-15mg/kg	Baseline (n=3)	7DPT (n=3)	3.857	0.050	0.165
TS-15mg/kg	Baseline (n=3)	9DPT (n=3)	2.333	0.127	0.253
TS-15mg/kg	2DPT (n=3)	5DPT (n=3)	0.429	0.513	0.513
TS-15mg/kg	2DPT (n=3)	7DPT (n=3)	0.429	0.513	0.513
TS-15mg/kg	2DPT (n=3)	9DPT (n=3)	1.190	0.275	0.459
TS-15mg/kg	5DPT (n=3)	7DPT (n=3)	0.429	0.513	0.513
TS-15mg/kg	5DPT (n=3)	9DPT (n=3)	0.429	0.513	0.513
TS-15mg/kg	7DPT (n=3)	9DPT (n=3)	3.857	0.050	0.165

Treatment	Group 1	Group 2	H	p-value	q-value
Saline	Baseline (n=3)	2DPT (n=3)	2.333	0.127	0.599
Saline	Baseline (n=3)	5DPT (n=3)	0.429	0.513	0.818
Saline	Baseline (n=3)	7DPT (n=2)	3.000	0.083	0.599
Saline	Baseline (n=3)	9DPT (n=1)	1.800	0.180	0.599
Saline	2DPT (n=3)	5DPT (n=3)	1.190	0.275	0.688
Saline	2DPT (n=3)	7DPT (n=2)	0.000	1.000	1.000
Saline	2DPT (n=3)	9DPT (n=1)	0.200	0.655	0.818
Saline	5DPT (n=3)	7DPT (n=2)	0.333	0.564	0.818
Saline	5DPT (n=3)	9DPT (n=1)	0.200	0.655	0.818
Saline	7DPT (n=2)	9DPT (n=1)	0.000	1.000	1.000

Supplementary File 3
Alpha Diversity Output

id	Chinchilla_ID	Treatment	Sex	Collection	Fecal observed_features
1BF	Chinch1	AC-PO7d	F	Baseline	414
2BF	Chinch2	AC-PO7d	F	Baseline	442
3BF	Chinch3	AC-PO7d	M	Baseline	484
20FBL	Chinch20	AC-5mg/kg	F	Baseline	241
23FBL	Chinch23	AC-5mg/kg	F	Baseline	237
22FBL	Chinch22	AC-10mg/kg	M	Baseline	224
25FBL	Chinch25	AC-10mg/kg	F	Baseline	174
11FBL	Chinch11	anti-rsPilA	F	Baseline	245
13FBL	Chinch13	anti-rsPilA	F	Baseline	188
17FBL	Chinch17	anti-rsPilA	F	Baseline	234
19FBL	Chinch19	anti-tip-chimer	F	Baseline	262
21FBL	Chinch21	anti-tip-chimer	F	Baseline	195
27FBL	Chinch27	anti-tip-chimer	M	Baseline	179
4FBL	Chinch4	Ofloxacin	F	Baseline	192
5FBL	Chinch5	Ofloxacin	F	Baseline	145
7FBL	Chinch7	Ofloxacin	M	Baseline	158
14FBL	Chinch14	TS-15mg/kg	F	Baseline	195
15FBL	Chinch15	TS-15mg/kg	F	Baseline	324
18FBL	Chinch18	TS-15mg/kg	M	Baseline	268
10FBL	Chinch10	TS-30mg/kg	F	Baseline	171
12FBL	Chinch12	TS-30mg/kg	M	Baseline	179
16FBL	Chinch16	TS-30mg/kg	F	Baseline	204
6FBL	Chinch6	Saline	F	Baseline	158
8FBL	Chinch8	Saline	M	Baseline	175
9FBL	Chinch9	Saline	F	Baseline	184
2AF2	Chinch2	AC-PO7d	F	1	63
3AF2	Chinch3	AC-PO7d	M	1	73
20F1	Chinch20	AC-5mg/kg	F	1	159
23F1	Chinch23	AC-5mg/kg	F	1	154
22F1	Chinch22	AC-10mg/kg	M	1	89
24F1	Chinch24	AC-10mg/kg	F	1	99
11F1	Chinch11	anti-rsPilA	F	1	186
13F1	Chinch13	anti-rsPilA	F	1	153
17F1	Chinch17	anti-rsPilA	F	1	209
19F1	Chinch19	anti-tip-chimer	F	1	173
21F1	Chinch21	anti-tip-chimer	F	1	204
27F1	Chinch27	anti-tip-chimer	M	1	162
4F1	Chinch4	Ofloxacin	F	1	161
7F1	Chinch7	Ofloxacin	M	1	128
14F1	Chinch14	TS-15mg/kg	F	1	156
15F1	Chinch15	TS-15mg/kg	F	1	111
18F1	Chinch18	TS-15mg/kg	M	1	158
10F1	Chinch10	TS-30mg/kg	F	1	150
12F1	Chinch12	TS-30mg/kg	M	1	201
16F1	Chinch16	TS-30mg/kg	F	1	155
6F1	Chinch6	Saline	F	1	167
8F1	Chinch8	Saline	M	1	146

Supplementary File 3
Alpha Diversity Output

id	Chinchilla_ID	Treatment	Sex	Collection	Fecal observed_features
9F1	Chinch9	Saline	F	1	112
1AF5	Chinch1	AC-PO7d	F	2	293
2AF5	Chinch2	AC-PO7d	F	2	109
3AF5	Chinch3	AC-PO7d	M	2	116
20F2	Chinch20	AC-5mg/kg	F	2	88
23F2	Chinch23	AC-5mg/kg	F	2	65
26F2	Chinch26	AC-5mg/kg	M	2	105
22F2	Chinch22	AC-10mg/kg	M	2	62
25F2	Chinch25	AC-10mg/kg	F	2	96
11F2	Chinch11	anti-rsPilA	F	2	206
17F2	Chinch17	anti-rsPilA	F	2	138
19F2	Chinch19	anti-tip-chimer	F	2	144
21F2	Chinch21	anti-tip-chimer	F	2	210
27F2	Chinch27	anti-tip-chimer	M	2	172
4F2	Chinch4	Ofloxacin	F	2	132
5F2	Chinch5	Ofloxacin	F	2	204
7F2	Chinch7	Ofloxacin	M	2	131
14F2	Chinch14	TS-15mg/kg	F	2	148
15F2	Chinch15	TS-15mg/kg	F	2	121
18F2	Chinch18	TS-15mg/kg	M	2	195
10F2	Chinch10	TS-30mg/kg	F	2	159
12F2	Chinch12	TS-30mg/kg	M	2	223
16F2	Chinch16	TS-30mg/kg	F	2	164
6F2	Chinch6	Saline	F	2	253
8F2	Chinch8	Saline	M	2	158
9F2	Chinch9	Saline	F	2	162
1AF7	Chinch1	AC-PO7d	F	3	60
2AF7	Chinch2	AC-PO7d	F	3	139
3AF7	Chinch3	AC-PO7d	M	3	99
20F3	Chinch20	AC-5mg/kg	F	3	151
23F3	Chinch23	AC-5mg/kg	F	3	108
26F3	Chinch26	AC-5mg/kg	M	3	140
25F3	Chinch25	AC-10mg/kg	F	3	161
11F3	Chinch11	anti-rsPilA	F	3	200
17F3	Chinch17	anti-rsPilA	F	3	227
19F3	Chinch19	anti-tip-chimer	F	3	157
21F3	Chinch21	anti-tip-chimer	F	3	236
4F3	Chinch4	Ofloxacin	F	3	112
5F3	Chinch5	Ofloxacin	F	3	194
7F3	Chinch7	Ofloxacin	M	3	153
14F3	Chinch14	TS-15mg/kg	F	3	122
15F3	Chinch15	TS-15mg/kg	F	3	129
18F3	Chinch18	TS-15mg/kg	M	3	126
10F3	Chinch10	TS-30mg/kg	F	3	202
12F3	Chinch12	TS-30mg/kg	M	3	187
16F3	Chinch16	TS-30mg/kg	F	3	172
6F3	Chinch6	Saline	F	3	155
8F3	Chinch8	Saline	M	3	126

Supplementary File 3
Alpha Diversity Output

id	Chinchilla_ID	Treatment	Sex	Collection	Fecal observed_features
2AF9	Chinch2	AC-PO7d	F	4	169
3AF9	Chinch3	AC-PO7d	M	4	169
20F4	Chinch20	AC-5mg/kg	F	4	137
23F4	Chinch23	AC-5mg/kg	F	4	188
26F4	Chinch26	AC-5mg/kg	M	4	113
22F4	Chinch22	AC-10mg/kg	M	4	169
25F4	Chinch25	AC-10mg/kg	F	4	140
11F4	Chinch11	anti-rsPilA	F	4	224
17F4	Chinch17	anti-rsPilA	F	4	198
19F4	Chinch19	anti-tip-chimer	F	4	171
21F4	Chinch21	anti-tip-chimer	F	4	223
27F4	Chinch27	anti-tip-chimer	M	4	183
4F4	Chinch4	Ofloxacin	F	4	110
5F4	Chinch5	Ofloxacin	F	4	153
7F4	Chinch7	Ofloxacin	M	4	154
14F4	Chinch14	TS-15mg/kg	F	4	169
15F4	Chinch15	TS-15mg/kg	F	4	130
18F4	Chinch18	TS-15mg/kg	M	4	248
10F4	Chinch10	TS-30mg/kg	F	4	156
12F4	Chinch12	TS-30mg/kg	M	4	222
16F4	Chinch16	TS-30mg/kg	F	4	213
8F4	Chinch8	Saline	M	4	150

Supplementary File 3
Alpha Diversity Output

Fecal Richness-Baseline

Kruskal-Wallis pairwise comparisons

Group 1	Group 2	H	p-value	q-value
AC-PO7d (n=3)	AC-5mg/kg (n=2)	3.000	0.083	0.214
AC-PO7d (n=3)	AC-10mg/kg (n=2)	3.000	0.083	0.214
AC-PO7d (n=3)	Ofloxacin (n=3)	3.857	0.050	0.198
AC-PO7d (n=3)	Saline (n=3)	3.857	0.050	0.198
AC-PO7d (n=3)	TS-30mg/kg (n=3)	3.857	0.050	0.198
AC-PO7d (n=3)	TS-15mg/kg (n=3)	3.857	0.050	0.198
AC-PO7d (n=3)	anti-tip-chimer (n=3)	3.857	0.050	0.198
AC-PO7d (n=3)	anti-rsPilA (n=3)	3.857	0.050	0.198
AC-5mg/kg (n=2)	AC-10mg/kg (n=2)	2.400	0.121	0.228
AC-5mg/kg (n=2)	Ofloxacin (n=3)	3.000	0.083	0.214
AC-5mg/kg (n=2)	Saline (n=3)	3.000	0.083	0.214
AC-5mg/kg (n=2)	TS-30mg/kg (n=3)	3.000	0.083	0.214
AC-5mg/kg (n=2)	TS-15mg/kg (n=3)	0.333	0.564	0.597
AC-5mg/kg (n=2)	anti-tip-chimer (n=3)	0.333	0.564	0.597
AC-5mg/kg (n=2)	anti-rsPilA (n=3)	0.333	0.564	0.597
AC-10mg/kg (n=2)	Ofloxacin (n=3)	1.333	0.248	0.372
AC-10mg/kg (n=2)	Saline (n=3)	0.333	0.564	0.597
AC-10mg/kg (n=2)	TS-30mg/kg (n=3)	0.333	0.564	0.597
AC-10mg/kg (n=2)	TS-15mg/kg (n=3)	1.333	0.248	0.372
AC-10mg/kg (n=2)	anti-tip-chimer (n=3)	0.333	0.564	0.597
AC-10mg/kg (n=2)	anti-rsPilA (n=3)	1.333	0.248	0.372
Ofloxacin (n=3)	Saline (n=3)	0.196	0.658	0.677
Ofloxacin (n=3)	TS-30mg/kg (n=3)	1.190	0.275	0.381
Ofloxacin (n=3)	TS-15mg/kg (n=3)	3.857	0.050	0.198
Ofloxacin (n=3)	anti-tip-chimer (n=3)	2.333	0.127	0.228
Ofloxacin (n=3)	anti-rsPilA (n=3)	2.333	0.127	0.228
Saline (n=3)	TS-30mg/kg (n=3)	0.429	0.513	0.597
Saline (n=3)	TS-15mg/kg (n=3)	3.857	0.050	0.198
Saline (n=3)	anti-tip-chimer (n=3)	2.333	0.127	0.228
Saline (n=3)	anti-rsPilA (n=3)	3.857	0.050	0.198
TS-30mg/kg (n=3)	TS-15mg/kg (n=3)	2.333	0.127	0.228
TS-30mg/kg (n=3)	anti-tip-chimer (n=3)	0.784	0.376	0.501
TS-30mg/kg (n=3)	anti-rsPilA (n=3)	2.333	0.127	0.228
TS-15mg/kg (n=3)	anti-tip-chimer (n=3)	1.765	0.184	0.315
TS-15mg/kg (n=3)	anti-rsPilA (n=3)	1.190	0.275	0.381
anti-tip-chimer (n=3)	anti-rsPilA (n=3)	0.048	0.827	0.827

Kruskal-Wallis (Omnibus Test)

**H**	17.365
**p-value**	0.027

Supplementary File 3
Alpha Diversity Output

Fecal Richness-2DPT

Kruskal-Wallis pairwise comparisons

Group 1	Group 2	H	p-value	q-value
AC-PO7d (n=2)	AC-5mg/kg (n=2)	2.400	0.121	0.228
AC-PO7d (n=2)	AC-10mg/kg (n=2)	2.400	0.121	0.228
AC-PO7d (n=2)	Ofloxacin (n=2)	2.400	0.121	0.228
AC-PO7d (n=2)	Saline (n=3)	3.000	0.083	0.228
AC-PO7d (n=2)	TS-30mg/kg (n=3)	3.000	0.083	0.228
AC-PO7d (n=2)	TS-15mg/kg (n=3)	3.000	0.083	0.228
AC-PO7d (n=2)	anti-tip-chimer (n=3)	3.000	0.083	0.228
AC-PO7d (n=2)	anti-rsPilA (n=3)	3.000	0.083	0.228
AC-5mg/kg (n=2)	AC-10mg/kg (n=2)	2.400	0.121	0.228
AC-5mg/kg (n=2)	Ofloxacin (n=2)	0.000	1.000	1.000
AC-5mg/kg (n=2)	Saline (n=3)	0.333	0.564	0.676
AC-5mg/kg (n=2)	TS-30mg/kg (n=3)	0.000	1.000	1.000
AC-5mg/kg (n=2)	TS-15mg/kg (n=3)	0.333	0.564	0.676
AC-5mg/kg (n=2)	anti-tip-chimer (n=3)	3.000	0.083	0.228
AC-5mg/kg (n=2)	anti-rsPilA (n=3)	0.333	0.564	0.676
AC-10mg/kg (n=2)	Ofloxacin (n=2)	2.400	0.121	0.228
AC-10mg/kg (n=2)	Saline (n=3)	3.000	0.083	0.228
AC-10mg/kg (n=2)	TS-30mg/kg (n=3)	3.000	0.083	0.228
AC-10mg/kg (n=2)	TS-15mg/kg (n=3)	3.000	0.083	0.228
AC-10mg/kg (n=2)	anti-tip-chimer (n=3)	3.000	0.083	0.228
AC-10mg/kg (n=2)	anti-rsPilA (n=3)	3.000	0.083	0.228
Ofloxacin (n=2)	Saline (n=3)	0.000	1.000	1.000
Ofloxacin (n=2)	TS-30mg/kg (n=3)	0.333	0.564	0.676
Ofloxacin (n=2)	TS-15mg/kg (n=3)	0.333	0.564	0.676
Ofloxacin (n=2)	anti-tip-chimer (n=3)	3.000	0.083	0.228
Ofloxacin (n=2)	anti-rsPilA (n=3)	1.333	0.248	0.413
Saline (n=3)	TS-30mg/kg (n=3)	1.190	0.275	0.413
Saline (n=3)	TS-15mg/kg (n=3)	0.048	0.827	0.902
Saline (n=3)	anti-tip-chimer (n=3)	2.333	0.127	0.228
Saline (n=3)	anti-rsPilA (n=3)	2.333	0.127	0.228
TS-30mg/kg (n=3)	TS-15mg/kg (n=3)	0.048	0.827	0.902
TS-30mg/kg (n=3)	anti-tip-chimer (n=3)	1.190	0.275	0.413
TS-30mg/kg (n=3)	anti-rsPilA (n=3)	0.429	0.513	0.676
TS-15mg/kg (n=3)	anti-tip-chimer (n=3)	3.857	0.050	0.228
TS-15mg/kg (n=3)	anti-rsPilA (n=3)	1.190	0.275	0.413
anti-tip-chimer (n=3)	anti-rsPilA (n=3)	0.048	0.827	0.902

Kruskal-Wallis (Omnibus Test)

**H**	14.083
**p-value**	0.080

Supplementary File 3
Alpha Diversity Output

Fecal Richness-5DPT

Kruskal-Wallis pairwise comparisons

Group 1	Group 2	H	p-value	q-value
AC-PO7d (n=3)	AC-5mg/kg (n=3)	3.857	0.050	0.231
AC-PO7d (n=3)	AC-10mg/kg (n=2)	3.000	0.083	0.231
AC-PO7d (n=3)	Ofloxacin (n=3)	0.429	0.513	0.634
AC-PO7d (n=3)	Saline (n=3)	0.429	0.513	0.634
AC-PO7d (n=3)	TS-30mg/kg (n=3)	0.429	0.513	0.634
AC-PO7d (n=3)	TS-15mg/kg (n=3)	0.429	0.513	0.634
AC-PO7d (n=3)	anti-tip-chimer (n=3)	0.429	0.513	0.634
AC-PO7d (n=3)	anti-rsPilA (n=2)	0.333	0.564	0.634
AC-5mg/kg (n=3)	AC-10mg/kg (n=2)	0.333	0.564	0.634
AC-5mg/kg (n=3)	Ofloxacin (n=3)	3.857	0.050	0.231
AC-5mg/kg (n=3)	Saline (n=3)	3.857	0.050	0.231
AC-5mg/kg (n=3)	TS-30mg/kg (n=3)	3.857	0.050	0.231
AC-5mg/kg (n=3)	TS-15mg/kg (n=3)	3.857	0.050	0.231
AC-5mg/kg (n=3)	anti-tip-chimer (n=3)	3.857	0.050	0.231
AC-5mg/kg (n=3)	anti-rsPilA (n=2)	3.000	0.083	0.231
AC-10mg/kg (n=2)	Ofloxacin (n=3)	3.000	0.083	0.231
AC-10mg/kg (n=2)	Saline (n=3)	3.000	0.083	0.231
AC-10mg/kg (n=2)	TS-30mg/kg (n=3)	3.000	0.083	0.231
AC-10mg/kg (n=2)	TS-15mg/kg (n=3)	3.000	0.083	0.231
AC-10mg/kg (n=2)	anti-tip-chimer (n=3)	3.000	0.083	0.231
AC-10mg/kg (n=2)	anti-rsPilA (n=2)	2.400	0.121	0.312
Ofloxacin (n=3)	Saline (n=3)	1.190	0.275	0.495
Ofloxacin (n=3)	TS-30mg/kg (n=3)	1.190	0.275	0.495
Ofloxacin (n=3)	TS-15mg/kg (n=3)	0.048	0.827	0.827
Ofloxacin (n=3)	anti-tip-chimer (n=3)	1.190	0.275	0.495
Ofloxacin (n=3)	anti-rsPilA (n=2)	1.333	0.248	0.495
Saline (n=3)	TS-30mg/kg (n=3)	0.048	0.827	0.827
Saline (n=3)	TS-15mg/kg (n=3)	1.190	0.275	0.495
Saline (n=3)	anti-tip-chimer (n=3)	0.048	0.827	0.827
Saline (n=3)	anti-rsPilA (n=2)	0.333	0.564	0.634
TS-30mg/kg (n=3)	TS-15mg/kg (n=3)	1.190	0.275	0.495
TS-30mg/kg (n=3)	anti-tip-chimer (n=3)	0.048	0.827	0.827
TS-30mg/kg (n=3)	anti-rsPilA (n=2)	0.333	0.564	0.634
TS-15mg/kg (n=3)	anti-tip-chimer (n=3)	0.429	0.513	0.634
TS-15mg/kg (n=3)	anti-rsPilA (n=2)	0.333	0.564	0.634
anti-tip-chimer (n=3)	anti-rsPilA (n=2)	0.333	0.564	0.634

Kruskal-Wallis (Omnibus Test)

**H**	13.591
**p-value**	0.093

Supplementary File 3
Alpha Diversity Output

Fecal Richness-7DPT

Kruskal-Wallis pairwise comparisons

Group 1	Group 2	H	p-value	q-value
AC-PO7d (n=3)	AC-5mg/kg (n=3)	2.333	0.127	0.304
AC-PO7d (n=3)	AC-10mg/kg (n=1)	1.800	0.180	0.341
AC-PO7d (n=3)	Ofloxacin (n=3)	2.333	0.127	0.304
AC-PO7d (n=3)	Saline (n=2)	1.333	0.248	0.372
AC-PO7d (n=3)	TS-30mg/kg (n=3)	3.857	0.050	0.273
AC-PO7d (n=3)	TS-15mg/kg (n=3)	0.429	0.513	0.615
AC-PO7d (n=3)	anti-tip-chimer (n=2)	3.000	0.083	0.273
AC-PO7d (n=3)	anti-rsPilA (n=2)	3.000	0.083	0.273
AC-5mg/kg (n=3)	AC-10mg/kg (n=1)	1.800	0.180	0.341
AC-5mg/kg (n=3)	Ofloxacin (n=3)	1.190	0.275	0.381
AC-5mg/kg (n=3)	Saline (n=2)	0.333	0.564	0.655
AC-5mg/kg (n=3)	TS-30mg/kg (n=3)	3.857	0.050	0.273
AC-5mg/kg (n=3)	TS-15mg/kg (n=3)	0.429	0.513	0.615
AC-5mg/kg (n=3)	anti-tip-chimer (n=2)	3.000	0.083	0.273
AC-5mg/kg (n=3)	anti-rsPilA (n=2)	3.000	0.083	0.273
AC-10mg/kg (n=1)	Ofloxacin (n=3)	0.200	0.655	0.737
AC-10mg/kg (n=1)	Saline (n=2)	1.500	0.221	0.372
AC-10mg/kg (n=1)	TS-30mg/kg (n=3)	1.800	0.180	0.341
AC-10mg/kg (n=1)	TS-15mg/kg (n=3)	1.800	0.180	0.341
AC-10mg/kg (n=1)	anti-tip-chimer (n=2)	0.000	1.000	1.000
AC-10mg/kg (n=1)	anti-rsPilA (n=2)	1.500	0.221	0.372
Ofloxacin (n=3)	Saline (n=2)	0.000	1.000	1.000
Ofloxacin (n=3)	TS-30mg/kg (n=3)	1.190	0.275	0.381
Ofloxacin (n=3)	TS-15mg/kg (n=3)	0.429	0.513	0.615
Ofloxacin (n=3)	anti-tip-chimer (n=2)	1.333	0.248	0.372
Ofloxacin (n=3)	anti-rsPilA (n=2)	3.000	0.083	0.273
Saline (n=2)	TS-30mg/kg (n=3)	3.000	0.083	0.273
Saline (n=2)	TS-15mg/kg (n=3)	0.789	0.374	0.499
Saline (n=2)	anti-tip-chimer (n=2)	2.400	0.121	0.304
Saline (n=2)	anti-rsPilA (n=2)	2.400	0.121	0.304
TS-30mg/kg (n=3)	TS-15mg/kg (n=3)	3.857	0.050	0.273
TS-30mg/kg (n=3)	anti-tip-chimer (n=2)	0.000	1.000	1.000
TS-30mg/kg (n=3)	anti-rsPilA (n=2)	1.333	0.248	0.372
TS-15mg/kg (n=3)	anti-tip-chimer (n=2)	3.000	0.083	0.273
TS-15mg/kg (n=3)	anti-rsPilA (n=2)	3.000	0.083	0.273
anti-tip-chimer (n=2)	anti-rsPilA (n=2)	0.000	1.000	1.000

Kruskal-Wallis (Omnibus Test)

**H**	15.235
**p-value**	0.055

Supplementary File 3
Alpha Diversity Output

Fecal Richness-9DPT

Kruskal-Wallis pairwise comparisons

Group 1	Group 2	H	p-value	q-value
AC-PO7d (n=2)	AC-5mg/kg (n=3)	0.351	0.554	0.752
AC-PO7d (n=2)	AC-10mg/kg (n=2)	1.000	0.317	0.601
AC-PO7d (n=2)	Ofloxacin (n=3)	3.158	0.076	0.428
AC-PO7d (n=2)	Saline (n=1)	2.000	0.157	0.498
AC-PO7d (n=2)	TS-30mg/kg (n=3)	0.351	0.554	0.752
AC-PO7d (n=2)	TS-15mg/kg (n=3)	0.000	1.000	1.000
AC-PO7d (n=2)	anti-tip-chimer (n=3)	3.158	0.076	0.428
AC-PO7d (n=2)	anti-rsPilA (n=2)	2.667	0.102	0.456
AC-5mg/kg (n=3)	AC-10mg/kg (n=2)	0.333	0.564	0.752
AC-5mg/kg (n=3)	Ofloxacin (n=3)	0.048	0.827	0.876
AC-5mg/kg (n=3)	Saline (n=1)	0.200	0.655	0.786
AC-5mg/kg (n=3)	TS-30mg/kg (n=3)	2.333	0.127	0.456
AC-5mg/kg (n=3)	TS-15mg/kg (n=3)	0.429	0.513	0.752
AC-5mg/kg (n=3)	anti-tip-chimer (n=3)	1.190	0.275	0.550
AC-5mg/kg (n=3)	anti-rsPilA (n=2)	3.000	0.083	0.428
AC-10mg/kg (n=2)	Ofloxacin (n=3)	0.333	0.564	0.752
AC-10mg/kg (n=2)	Saline (n=1)	0.000	1.000	1.000
AC-10mg/kg (n=2)	TS-30mg/kg (n=3)	1.333	0.248	0.550
AC-10mg/kg (n=2)	TS-15mg/kg (n=3)	0.088	0.767	0.876
AC-10mg/kg (n=2)	anti-tip-chimer (n=3)	3.000	0.083	0.428
AC-10mg/kg (n=2)	anti-rsPilA (n=2)	2.400	0.121	0.456
Ofloxacin (n=3)	Saline (n=1)	0.200	0.655	0.786
Ofloxacin (n=3)	TS-30mg/kg (n=3)	3.857	0.050	0.428
Ofloxacin (n=3)	TS-15mg/kg (n=3)	1.190	0.275	0.550
Ofloxacin (n=3)	anti-tip-chimer (n=3)	3.857	0.050	0.428
Ofloxacin (n=3)	anti-rsPilA (n=2)	3.000	0.083	0.428
Saline (n=1)	TS-30mg/kg (n=3)	1.800	0.180	0.498
Saline (n=1)	TS-15mg/kg (n=3)	0.200	0.655	0.786
Saline (n=1)	anti-tip-chimer (n=3)	1.800	0.180	0.498
Saline (n=1)	anti-rsPilA (n=2)	1.500	0.221	0.550
TS-30mg/kg (n=3)	TS-15mg/kg (n=3)	0.048	0.827	0.876
TS-30mg/kg (n=3)	anti-tip-chimer (n=3)	0.048	0.827	0.876
TS-30mg/kg (n=3)	anti-rsPilA (n=2)	0.333	0.564	0.752
TS-15mg/kg (n=3)	anti-tip-chimer (n=3)	0.429	0.513	0.752
TS-15mg/kg (n=3)	anti-rsPilA (n=2)	0.333	0.564	0.752
anti-tip-chimer (n=3)	anti-rsPilA (n=2)	1.333	0.248	0.550

Kruskal-Wallis (Omnibus Test)

**H**	10.626
p-value	0.224

Supplementary File 3
Alpha Diversity Output

Fecal Richness--Within-treatment Kruskal-Wallis pairwise comparisons

Treatment	Group 1	Group 2	H	p-value	q-value
AC-PO7d	Baseline (n=3)	2DPT (n=2)	3.000	0.083	0.139
AC-PO7d	Baseline (n=3)	5DPT (n=3)	3.857	0.050	0.139
AC-PO7d	Baseline (n=3)	7DPT (n=3)	3.857	0.050	0.139
AC-PO7d	Baseline (n=3)	9DPT (n=2)	3.158	0.076	0.139
AC-PO7d	2DPT (n=2)	5DPT (n=3)	3.000	0.083	0.139
AC-PO7d	2DPT (n=2)	7DPT (n=3)	0.333	0.564	0.564
AC-PO7d	2DPT (n=2)	9DPT (n=2)	2.667	0.102	0.146
AC-PO7d	5DPT (n=3)	7DPT (n=3)	1.190	0.275	0.344
AC-PO7d	5DPT (n=3)	9DPT (n=2)	0.351	0.554	0.564
AC-PO7d	7DPT (n=3)	9DPT (n=2)	3.158	0.076	0.139

Treatment	Group 1	Group 2	H	p-value	q-value
AC-10mg/kg	Baseline (n=2)	2DPT (n=2)	2.400	0.121	0.243
AC-10mg/kg	Baseline (n=2)	5DPT (n=2)	2.400	0.121	0.243
AC-10mg/kg	Baseline (n=2)	7DPT (n=1)	1.500	0.221	0.276
AC-10mg/kg	Baseline (n=2)	9DPT (n=2)	2.400	0.121	0.243
AC-10mg/kg	2DPT (n=2)	5DPT (n=2)	0.600	0.439	0.487
AC-10mg/kg	2DPT (n=2)	7DPT (n=1)	1.500	0.221	0.276
AC-10mg/kg	2DPT (n=2)	9DPT (n=2)	2.400	0.121	0.243
AC-10mg/kg	5DPT (n=2)	7DPT (n=1)	1.500	0.221	0.276
AC-10mg/kg	5DPT (n=2)	9DPT (n=2)	2.400	0.121	0.243
AC-10mg/kg	7DPT (n=1)	9DPT (n=2)	0.000	1.000	1.000

Treatment	Group 1	Group 2	H	p-value	q-value
AC-5mg/kg	Baseline (n=2)	2DPT (n=2)	2.400	0.121	0.152
AC-5mg/kg	Baseline (n=2)	5DPT (n=3)	3.000	0.083	0.119
AC-5mg/kg	Baseline (n=2)	7DPT (n=3)	3.000	0.083	0.119
AC-5mg/kg	Baseline (n=2)	9DPT (n=3)	3.000	0.083	0.119
AC-5mg/kg	2DPT (n=2)	5DPT (n=3)	3.000	0.083	0.119
AC-5mg/kg	2DPT (n=2)	7DPT (n=3)	3.000	0.083	0.119
AC-5mg/kg	2DPT (n=2)	9DPT (n=3)	0.333	0.564	0.626
AC-5mg/kg	5DPT (n=3)	7DPT (n=3)	3.857	0.050	0.119
AC-5mg/kg	5DPT (n=3)	9DPT (n=3)	3.857	0.050	0.119
AC-5mg/kg	7DPT (n=3)	9DPT (n=3)	0.048	0.827	0.827

Supplementary File 3
Alpha Diversity Output

Fecal Richness--Within-treatment Kruskal-Wallis pairwise comparisons

Treatment	Group 1	Group 2	H	p-value	q-value
anti-rsPilA	Baseline (n=3)	2DPT (n=3)	2.333	0.127	0.564
anti-rsPilA	Baseline (n=3)	5DPT (n=2)	1.333	0.248	0.564
anti-rsPilA	Baseline (n=3)	7DPT (n=2)	0.333	0.564	0.564
anti-rsPilA	Baseline (n=3)	9DPT (n=2)	0.333	0.564	0.564
anti-rsPilA	2DPT (n=3)	5DPT (n=2)	0.333	0.564	0.564
anti-rsPilA	2DPT (n=3)	7DPT (n=2)	1.333	0.248	0.564
anti-rsPilA	2DPT (n=3)	9DPT (n=2)	1.333	0.248	0.564
anti-rsPilA	5DPT (n=2)	7DPT (n=2)	0.600	0.439	0.564
anti-rsPilA	5DPT (n=2)	9DPT (n=2)	0.600	0.439	0.564
anti-rsPilA	7DPT (n=2)	9DPT (n=2)	0.600	0.439	0.564

Treatment	Group 1	Group 2	H	p-value	q-value
anti-tip-chimer	Baseline (n=3)	2DPT (n=3)	1.190	0.275	0.805
anti-tip-chimer	Baseline (n=3)	5DPT (n=3)	1.190	0.275	0.805
anti-tip-chimer	Baseline (n=3)	7DPT (n=2)	0.333	0.564	0.805
anti-tip-chimer	Baseline (n=3)	9DPT (n=3)	0.429	0.513	0.805
anti-tip-chimer	2DPT (n=3)	5DPT (n=3)	0.048	0.827	1.000
anti-tip-chimer	2DPT (n=3)	7DPT (n=2)	0.000	1.000	1.000
anti-tip-chimer	2DPT (n=3)	9DPT (n=3)	0.429	0.513	0.805
anti-tip-chimer	5DPT (n=3)	7DPT (n=2)	0.333	0.564	0.805
anti-tip-chimer	5DPT (n=3)	9DPT (n=3)	0.429	0.513	0.805
anti-tip-chimer	7DPT (n=2)	9DPT (n=3)	0.000	1.000	1.000

Treatment	Group 1	Group 2	H	p-value	q-value
Ofloxacin	Baseline (n=3)	2DPT (n=2)	0.333	0.564	0.919
Ofloxacin	Baseline (n=3)	5DPT (n=3)	0.429	0.513	0.919
Ofloxacin	Baseline (n=3)	7DPT (n=3)	0.048	0.827	0.919
Ofloxacin	Baseline (n=3)	9DPT (n=3)	1.190	0.275	0.919
Ofloxacin	2DPT (n=2)	5DPT (n=3)	0.333	0.564	0.919
Ofloxacin	2DPT (n=2)	7DPT (n=3)	0.000	1.000	1.000
Ofloxacin	2DPT (n=2)	9DPT (n=3)	0.333	0.564	0.919
Ofloxacin	5DPT (n=3)	7DPT (n=3)	0.048	0.827	0.919
Ofloxacin	5DPT (n=3)	9DPT (n=3)	0.048	0.827	0.919
Ofloxacin	7DPT (n=3)	9DPT (n=3)	0.196	0.658	0.919

Supplementary File 3
Alpha Diversity Output

Fecal Richness--Within-treatment Kruskal-Wallis pairwise comparisons

Treatment	Group 1	Group 2	H	p-value	q-value
TS-30mg/kg	Baseline (n=3)	2DPT (n=3)	1.190	0.275	0.641
TS-30mg/kg	Baseline (n=3)	5DPT (n=3)	0.429	0.513	0.641
TS-30mg/kg	Baseline (n=3)	7DPT (n=3)	0.048	0.827	0.827
TS-30mg/kg	Baseline (n=3)	9DPT (n=3)	0.429	0.513	0.641
TS-30mg/kg	2DPT (n=3)	5DPT (n=3)	1.190	0.275	0.641
TS-30mg/kg	2DPT (n=3)	7DPT (n=3)	1.190	0.275	0.641
TS-30mg/kg	2DPT (n=3)	9DPT (n=3)	2.333	0.127	0.641
TS-30mg/kg	5DPT (n=3)	7DPT (n=3)	0.429	0.513	0.641
TS-30mg/kg	5DPT (n=3)	9DPT (n=3)	0.048	0.827	0.827
TS-30mg/kg	7DPT (n=3)	9DPT (n=3)	0.429	0.513	0.641

Treatment	Group 1	Group 2	H	p-value	q-value
TS-15mg/kg	Baseline (n=3)	2DPT (n=3)	3.857	0.050	0.165
TS-15mg/kg	Baseline (n=3)	5DPT (n=3)	3.137	0.077	0.191
TS-15mg/kg	Baseline (n=3)	7DPT (n=3)	3.857	0.050	0.165
TS-15mg/kg	Baseline (n=3)	9DPT (n=3)	2.333	0.127	0.253
TS-15mg/kg	2DPT (n=3)	5DPT (n=3)	0.048	0.827	0.827
TS-15mg/kg	2DPT (n=3)	7DPT (n=3)	0.429	0.513	0.570
TS-15mg/kg	2DPT (n=3)	9DPT (n=3)	1.190	0.275	0.459
TS-15mg/kg	5DPT (n=3)	7DPT (n=3)	0.429	0.513	0.570
TS-15mg/kg	5DPT (n=3)	9DPT (n=3)	0.429	0.513	0.570
TS-15mg/kg	7DPT (n=3)	9DPT (n=3)	3.857	0.050	0.165

Treatment	Group 1	Group 2	H	p-value	q-value
Saline	Baseline (n=3)	2DPT (n=3)	2.333	0.127	0.359
Saline	Baseline (n=3)	5DPT (n=3)	0.000	1.000	1.000
Saline	Baseline (n=3)	7DPT (n=2)	3.000	0.083	0.359
Saline	Baseline (n=3)	9DPT (n=1)	1.800	0.180	0.359
Saline	2DPT (n=3)	5DPT (n=3)	1.190	0.275	0.459
Saline	2DPT (n=3)	7DPT (n=2)	0.000	1.000	1.000
Saline	2DPT (n=3)	9DPT (n=1)	0.200	0.655	0.935
Saline	5DPT (n=3)	7DPT (n=2)	3.000	0.083	0.359
Saline	5DPT (n=3)	9DPT (n=1)	1.800	0.180	0.359
Saline	7DPT (n=2)	9DPT (n=1)	0.000	1.000	1.000

Supplementary File 3
Alpha Diversity Output

id	Chinchilla_ID	Treatment	Sex	Collection	Fecal shannon_entropy
1BF	Chinch1	AC-PO7d	F	Baseline	6.762757742
2BF	Chinch2	AC-PO7d	F	Baseline	7.274228392
3BF	Chinch3	AC-PO7d	M	Baseline	7.285411512
20FBL	Chinch20	AC-5mg/kg	F	Baseline	6.554421415
23FBL	Chinch23	AC-5mg/kg	F	Baseline	5.884852081
22FBL	Chinch22	AC-10mg/kg	M	Baseline	6.090773077
25FBL	Chinch25	AC-10mg/kg	F	Baseline	6.44691863
11FBL	Chinch11	anti-rsPilA	F	Baseline	6.96633385
13FBL	Chinch13	anti-rsPilA	F	Baseline	6.533729688
17FBL	Chinch17	anti-rsPilA	F	Baseline	6.781833207
19FBL	Chinch19	anti-tip-chimer	F	Baseline	6.678350498
21FBL	Chinch21	anti-tip-chimer	F	Baseline	5.906434731
27FBL	Chinch27	anti-tip-chimer	M	Baseline	6.194896873
4FBL	Chinch4	Ofloxacin	F	Baseline	5.693648838
5FBL	Chinch5	Ofloxacin	F	Baseline	6.311360327
7FBL	Chinch7	Ofloxacin	M	Baseline	6.177154529
14FBL	Chinch14	TS-15mg/kg	F	Baseline	6.228554327
15FBL	Chinch15	TS-15mg/kg	F	Baseline	6.792007952
18FBL	Chinch18	TS-15mg/kg	M	Baseline	6.972237896
10FBL	Chinch10	TS-30mg/kg	F	Baseline	6.428095721
12FBL	Chinch12	TS-30mg/kg	M	Baseline	6.638072302
16FBL	Chinch16	TS-30mg/kg	F	Baseline	6.5236789
6FBL	Chinch6	Saline	F	Baseline	6.246790606
8FBL	Chinch8	Saline	M	Baseline	6.685697045
9FBL	Chinch9	Saline	F	Baseline	5.748525671
2AF2	Chinch2	AC-PO7d	F	1	3.425935933
3AF2	Chinch3	AC-PO7d	M	1	3.603164867
20F1	Chinch20	AC-5mg/kg	F	1	6.21982859
23F1	Chinch23	AC-5mg/kg	F	1	5.633542233
22F1	Chinch22	AC-10mg/kg	M	1	4.531921545
24F1	Chinch24	AC-10mg/kg	F	1	4.488303821
11F1	Chinch11	anti-rsPilA	F	1	5.999389351
13F1	Chinch13	anti-rsPilA	F	1	6.033885884
17F1	Chinch17	anti-rsPilA	F	1	6.492346817
19F1	Chinch19	anti-tip-chimer	F	1	5.6626546
21F1	Chinch21	anti-tip-chimer	F	1	6.424112109
27F1	Chinch27	anti-tip-chimer	M	1	6.030497081
4F1	Chinch4	Ofloxacin	F	1	5.863848194
7F1	Chinch7	Ofloxacin	M	1	5.525003657
14F1	Chinch14	TS-15mg/kg	F	1	6.176439576
15F1	Chinch15	TS-15mg/kg	F	1	5.496227349
18F1	Chinch18	TS-15mg/kg	M	1	6.046288371
10F1	Chinch10	TS-30mg/kg	F	1	5.752407706
12F1	Chinch12	TS-30mg/kg	M	1	5.843456595
16F1	Chinch16	TS-30mg/kg	F	1	6.397343284
6F1	Chinch6	Saline	F	1	6.468878007
8F1	Chinch8	Saline	M	1	6.205053889

Supplementary File 3
Alpha Diversity Output

id	Chinchilla_ID	Treatment	Sex	Collection	Fecal shannon_entropy
9F1	Chinch9	Saline	F	1	5.035201696
1AF5	Chinch1	AC-PO7d	F	2	7.035746088
2AF5	Chinch2	AC-PO7d	F	2	4.608446385
3AF5	Chinch3	AC-PO7d	M	2	5.811571089
20F2	Chinch20	AC-5mg/kg	F	2	4.766826929
23F2	Chinch23	AC-5mg/kg	F	2	4.27309007
26F2	Chinch26	AC-5mg/kg	M	2	5.604503276
22F2	Chinch22	AC-10mg/kg	M	2	4.09106472
25F2	Chinch25	AC-10mg/kg	F	2	4.357819146
11F2	Chinch11	anti-rsPilA	F	2	6.54764948
17F2	Chinch17	anti-rsPilA	F	2	5.851654051
19F2	Chinch19	anti-tip-chimer	F	2	5.238558759
21F2	Chinch21	anti-tip-chimer	F	2	6.565455405
27F2	Chinch27	anti-tip-chimer	M	2	5.918635677
4F2	Chinch4	Ofloxacin	F	2	5.567248261
5F2	Chinch5	Ofloxacin	F	2	6.478042066
7F2	Chinch7	Ofloxacin	M	2	6.067843791
14F2	Chinch14	TS-15mg/kg	F	2	5.669723977
15F2	Chinch15	TS-15mg/kg	F	2	5.862027473
18F2	Chinch18	TS-15mg/kg	M	2	6.152890749
10F2	Chinch10	TS-30mg/kg	F	2	6.029059694
12F2	Chinch12	TS-30mg/kg	M	2	5.439369359
16F2	Chinch16	TS-30mg/kg	F	2	6.418586093
6F2	Chinch6	Saline	F	2	6.767672566
8F2	Chinch8	Saline	M	2	6.299926351
9F2	Chinch9	Saline	F	2	5.791620975
1AF7	Chinch1	AC-PO7d	F	3	4.029992911
2AF7	Chinch2	AC-PO7d	F	3	4.460732241
3AF7	Chinch3	AC-PO7d	M	3	3.74542813
20F3	Chinch20	AC-5mg/kg	F	3	5.821773869
23F3	Chinch23	AC-5mg/kg	F	3	5.411843512
26F3	Chinch26	AC-5mg/kg	M	3	5.821513304
25F3	Chinch25	AC-10mg/kg	F	3	5.71236009
11F3	Chinch11	anti-rsPilA	F	3	6.50244268
17F3	Chinch17	anti-rsPilA	F	3	7.138484129
19F3	Chinch19	anti-tip-chimer	F	3	5.802015248
21F3	Chinch21	anti-tip-chimer	F	3	6.27602072
4F3	Chinch4	Ofloxacin	F	3	5.351705871
5F3	Chinch5	Ofloxacin	F	3	6.399819529
7F3	Chinch7	Ofloxacin	M	3	6.098598767
14F3	Chinch14	TS-15mg/kg	F	3	5.385630067
15F3	Chinch15	TS-15mg/kg	F	3	5.6028269
18F3	Chinch18	TS-15mg/kg	M	3	5.442420803
10F3	Chinch10	TS-30mg/kg	F	3	6.261103325
12F3	Chinch12	TS-30mg/kg	M	3	5.667661223
16F3	Chinch16	TS-30mg/kg	F	3	6.427694889
6F3	Chinch6	Saline	F	3	6.210291856
8F3	Chinch8	Saline	M	3	6.053927038

Supplementary File 3
Alpha Diversity Output

id	Chinchilla_ID	Treatment	Sex	Collection	Fecal shannon_entropy
2AF9	Chinch2	AC-PO7d	F	4	6.054625985
3AF9	Chinch3	AC-PO7d	M	4	4.696531485
20F4	Chinch20	AC-5mg/kg	F	4	5.672595022
23F4	Chinch23	AC-5mg/kg	F	4	5.991762518
26F4	Chinch26	AC-5mg/kg	M	4	5.814668917
22F4	Chinch22	AC-10mg/kg	M	4	5.535670666
25F4	Chinch25	AC-10mg/kg	F	4	5.489313083
11F4	Chinch11	anti-rsPilA	F	4	6.370310816
17F4	Chinch17	anti-rsPilA	F	4	6.32714978
19F4	Chinch19	anti-tip-chimer	F	4	6.144107863
21F4	Chinch21	anti-tip-chimer	F	4	6.733271365
27F4	Chinch27	anti-tip-chimer	M	4	6.463280637
4F4	Chinch4	Ofloxacin	F	4	5.568919113
5F4	Chinch5	Ofloxacin	F	4	6.191489347
7F4	Chinch7	Ofloxacin	M	4	5.836095871
14F4	Chinch14	TS-15mg/kg	F	4	5.910062422
15F4	Chinch15	TS-15mg/kg	F	4	5.933694844
18F4	Chinch18	TS-15mg/kg	M	4	6.536938427
10F4	Chinch10	TS-30mg/kg	F	4	6.223089229
12F4	Chinch12	TS-30mg/kg	M	4	6.355147068
16F4	Chinch16	TS-30mg/kg	F	4	5.547301062
8F4	Chinch8	Saline	M	4	6.13511048

Supplementary File 3
Alpha Diversity Output

Fecal Shannon Diversity-Baseline

Kruskal-Wallis pairwise comparisons

Group 1	Group 2	H	p-value	q-value
AC-PO7d (n=3)	AC-5mg/kg (n=2)	3.000	0.083	0.326
AC-PO7d (n=3)	AC-10mg/kg (n=2)	3.000	0.083	0.326
AC-PO7d (n=3)	Ofloxacin (n=3)	3.857	0.050	0.297
AC-PO7d (n=3)	Saline (n=3)	3.857	0.050	0.297
AC-PO7d (n=3)	TS-30mg/kg (n=3)	3.857	0.050	0.297
AC-PO7d (n=3)	TS-15mg/kg (n=3)	1.190	0.275	0.472
AC-PO7d (n=3)	anti-tip-chimer (n=3)	3.857	0.050	0.297
AC-PO7d (n=3)	anti-rsPilA (n=3)	1.190	0.275	0.472
AC-5mg/kg (n=2)	AC-10mg/kg (n=2)	0.000	1.000	1.000
AC-5mg/kg (n=2)	Ofloxacin (n=3)	0.333	0.564	0.676
AC-5mg/kg (n=2)	Saline (n=3)	0.000	1.000	1.000
AC-5mg/kg (n=2)	TS-30mg/kg (n=3)	0.333	0.564	0.676
AC-5mg/kg (n=2)	TS-15mg/kg (n=3)	1.333	0.248	0.472
AC-5mg/kg (n=2)	anti-tip-chimer (n=3)	0.333	0.564	0.676
AC-5mg/kg (n=2)	anti-rsPilA (n=3)	1.333	0.248	0.472
AC-10mg/kg (n=2)	Ofloxacin (n=3)	0.333	0.564	0.676
AC-10mg/kg (n=2)	Saline (n=3)	0.000	1.000	1.000
AC-10mg/kg (n=2)	TS-30mg/kg (n=3)	1.333	0.248	0.472
AC-10mg/kg (n=2)	TS-15mg/kg (n=3)	1.333	0.248	0.472
AC-10mg/kg (n=2)	anti-tip-chimer (n=3)	0.000	1.000	1.000
AC-10mg/kg (n=2)	anti-rsPilA (n=3)	3.000	0.083	0.326
Ofloxacin (n=3)	Saline (n=3)	0.429	0.513	0.676
Ofloxacin (n=3)	TS-30mg/kg (n=3)	3.857	0.050	0.297
Ofloxacin (n=3)	TS-15mg/kg (n=3)	2.333	0.127	0.326
Ofloxacin (n=3)	anti-tip-chimer (n=3)	0.429	0.513	0.676
Ofloxacin (n=3)	anti-rsPilA (n=3)	3.857	0.050	0.297
Saline (n=3)	TS-30mg/kg (n=3)	0.429	0.513	0.676
Saline (n=3)	TS-15mg/kg (n=3)	1.190	0.275	0.472
Saline (n=3)	anti-tip-chimer (n=3)	0.048	0.827	0.931
Saline (n=3)	anti-rsPilA (n=3)	2.333	0.127	0.326
TS-30mg/kg (n=3)	TS-15mg/kg (n=3)	0.429	0.513	0.676
TS-30mg/kg (n=3)	anti-tip-chimer (n=3)	0.429	0.513	0.676
TS-30mg/kg (n=3)	anti-rsPilA (n=3)	2.333	0.127	0.326
TS-15mg/kg (n=3)	anti-tip-chimer (n=3)	2.333	0.127	0.326
TS-15mg/kg (n=3)	anti-rsPilA (n=3)	0.048	0.827	0.931
anti-tip-chimer (n=3)	anti-rsPilA (n=3)	2.333	0.127	0.326

Kruskal-Wallis (Omnibus Test)

**H**	13.677
**p-value**	0.091

Supplementary File 3
Alpha Diversity Output

Fecal Shannon Diversity-2DPT

Kruskal-Wallis pairwise comparisons

Group 1	Group 2	H	p-value	q-value
AC-PO7d (n=2)	AC-5mg/kg (n=2)	2.400	0.121	0.273
AC-PO7d (n=2)	AC-10mg/kg (n=2)	2.400	0.121	0.273
AC-PO7d (n=2)	Ofloxacin (n=2)	2.400	0.121	0.273
AC-PO7d (n=2)	Saline (n=3)	3.000	0.083	0.273
AC-PO7d (n=2)	TS-30mg/kg (n=3)	3.000	0.083	0.273
AC-PO7d (n=2)	TS-15mg/kg (n=3)	3.000	0.083	0.273
AC-PO7d (n=2)	anti-tip-chimer (n=3)	3.000	0.083	0.273
AC-PO7d (n=2)	anti-rsPilA (n=3)	3.000	0.083	0.273
AC-5mg/kg (n=2)	AC-10mg/kg (n=2)	2.400	0.121	0.273
AC-5mg/kg (n=2)	Ofloxacin (n=2)	0.600	0.439	0.725
AC-5mg/kg (n=2)	Saline (n=3)	0.000	1.000	1.000
AC-5mg/kg (n=2)	TS-30mg/kg (n=3)	0.333	0.564	0.725
AC-5mg/kg (n=2)	TS-15mg/kg (n=3)	0.333	0.564	0.725
AC-5mg/kg (n=2)	anti-tip-chimer (n=3)	0.333	0.564	0.725
AC-5mg/kg (n=2)	anti-rsPilA (n=3)	0.333	0.564	0.725
AC-10mg/kg (n=2)	Ofloxacin (n=2)	2.400	0.121	0.273
AC-10mg/kg (n=2)	Saline (n=3)	3.000	0.083	0.273
AC-10mg/kg (n=2)	TS-30mg/kg (n=3)	3.000	0.083	0.273
AC-10mg/kg (n=2)	TS-15mg/kg (n=3)	3.000	0.083	0.273
AC-10mg/kg (n=2)	anti-tip-chimer (n=3)	3.000	0.083	0.273
AC-10mg/kg (n=2)	anti-rsPilA (n=3)	3.000	0.083	0.273
Ofloxacin (n=2)	Saline (n=3)	0.333	0.564	0.725
Ofloxacin (n=2)	TS-30mg/kg (n=3)	0.333	0.564	0.725
Ofloxacin (n=2)	TS-15mg/kg (n=3)	0.333	0.564	0.725
Ofloxacin (n=2)	anti-tip-chimer (n=3)	1.333	0.248	0.526
Ofloxacin (n=2)	anti-rsPilA (n=3)	3.000	0.083	0.273
Saline (n=3)	TS-30mg/kg (n=3)	0.048	0.827	0.851
Saline (n=3)	TS-15mg/kg (n=3)	0.429	0.513	0.725
Saline (n=3)	anti-tip-chimer (n=3)	0.048	0.827	0.851
Saline (n=3)	anti-rsPilA (n=3)	0.048	0.827	0.851
TS-30mg/kg (n=3)	TS-15mg/kg (n=3)	0.048	0.827	0.851
TS-30mg/kg (n=3)	anti-tip-chimer (n=3)	0.048	0.827	0.851
TS-30mg/kg (n=3)	anti-rsPilA (n=3)	1.190	0.275	0.550
TS-15mg/kg (n=3)	anti-tip-chimer (n=3)	0.048	0.827	0.851
TS-15mg/kg (n=3)	anti-rsPilA (n=3)	0.048	0.827	0.851
anti-tip-chimer (n=3)	anti-rsPilA (n=3)	0.429	0.513	0.725

Kruskal-Wallis (Omnibus Test)

**H**	11.232
**p-value**	0.189

Supplementary File 3
Alpha Diversity Output

Fecal Shannon Diversity-5DPT

Kruskal-Wallis pairwise comparisons

Group 1	Group 2	H	p-value	q-value
AC-PO7d (n=3)	AC-5mg/kg (n=3)	1.190	0.275	0.619
AC-PO7d (n=3)	AC-10mg/kg (n=2)	3.000	0.083	0.333
AC-PO7d (n=3)	Ofloxacin (n=3)	0.048	0.827	0.876
AC-PO7d (n=3)	Saline (n=3)	0.048	0.827	0.876
AC-PO7d (n=3)	TS-30mg/kg (n=3)	0.048	0.827	0.876
AC-PO7d (n=3)	TS-15mg/kg (n=3)	0.048	0.827	0.876
AC-PO7d (n=3)	anti-tip-chimer (n=3)	0.048	0.827	0.876
AC-PO7d (n=3)	anti-rsPilA (n=2)	0.333	0.564	0.846
AC-5mg/kg (n=3)	AC-10mg/kg (n=2)	1.333	0.248	0.619
AC-5mg/kg (n=3)	Ofloxacin (n=3)	2.333	0.127	0.351
AC-5mg/kg (n=3)	Saline (n=3)	3.857	0.050	0.333
AC-5mg/kg (n=3)	TS-30mg/kg (n=3)	2.333	0.127	0.351
AC-5mg/kg (n=3)	TS-15mg/kg (n=3)	3.857	0.050	0.333
AC-5mg/kg (n=3)	anti-tip-chimer (n=3)	2.333	0.127	0.351
AC-5mg/kg (n=3)	anti-rsPilA (n=2)	3.000	0.083	0.333
AC-10mg/kg (n=2)	Ofloxacin (n=3)	3.000	0.083	0.333
AC-10mg/kg (n=2)	Saline (n=3)	3.000	0.083	0.333
AC-10mg/kg (n=2)	TS-30mg/kg (n=3)	3.000	0.083	0.333
AC-10mg/kg (n=2)	TS-15mg/kg (n=3)	3.000	0.083	0.333
AC-10mg/kg (n=2)	anti-tip-chimer (n=3)	3.000	0.083	0.333
AC-10mg/kg (n=2)	anti-rsPilA (n=2)	2.400	0.121	0.351
Ofloxacin (n=3)	Saline (n=3)	0.429	0.513	0.846
Ofloxacin (n=3)	TS-30mg/kg (n=3)	0.429	0.513	0.846
Ofloxacin (n=3)	TS-15mg/kg (n=3)	0.048	0.827	0.876
Ofloxacin (n=3)	anti-tip-chimer (n=3)	0.048	0.827	0.876
Ofloxacin (n=3)	anti-rsPilA (n=2)	0.333	0.564	0.846
Saline (n=3)	TS-30mg/kg (n=3)	0.429	0.513	0.846
Saline (n=3)	TS-15mg/kg (n=3)	1.190	0.275	0.619
Saline (n=3)	anti-tip-chimer (n=3)	0.429	0.513	0.846
Saline (n=3)	anti-rsPilA (n=2)	0.000	1.000	1.000
TS-30mg/kg (n=3)	TS-15mg/kg (n=3)	0.048	0.827	0.876
TS-30mg/kg (n=3)	anti-tip-chimer (n=3)	0.048	0.827	0.876
TS-30mg/kg (n=3)	anti-rsPilA (n=2)	0.333	0.564	0.846
TS-15mg/kg (n=3)	anti-tip-chimer (n=3)	0.048	0.827	0.876
TS-15mg/kg (n=3)	anti-rsPilA (n=2)	0.333	0.564	0.846
anti-tip-chimer (n=3)	anti-rsPilA (n=2)	0.000	1.000	1.000

Kruskal-Wallis (Omnibus Test)

**H**	10.489
**p-value**	0.232

Supplementary File 3
Alpha Diversity Output

Fecal Shannon Diversity-7DPT

Kruskal-Wallis pairwise comparisons

Group 1	Group 2	H	p-value	q-value
AC-PO7d (n=3)	AC-5mg/kg (n=3)	3.857	0.050	0.200
AC-PO7d (n=3)	AC-10mg/kg (n=1)	1.800	0.180	0.341
AC-PO7d (n=3)	Ofloxacin (n=3)	3.857	0.050	0.200
AC-PO7d (n=3)	Saline (n=2)	3.000	0.083	0.200
AC-PO7d (n=3)	TS-30mg/kg (n=3)	3.857	0.050	0.200
AC-PO7d (n=3)	TS-15mg/kg (n=3)	3.857	0.050	0.200
AC-PO7d (n=3)	anti-tip-chimer (n=2)	3.000	0.083	0.200
AC-PO7d (n=3)	anti-rsPilA (n=2)	3.000	0.083	0.200
AC-5mg/kg (n=3)	AC-10mg/kg (n=1)	0.200	0.655	0.737
AC-5mg/kg (n=3)	Ofloxacin (n=3)	0.429	0.513	0.684
AC-5mg/kg (n=3)	Saline (n=2)	3.000	0.083	0.200
AC-5mg/kg (n=3)	TS-30mg/kg (n=3)	1.190	0.275	0.413
AC-5mg/kg (n=3)	TS-15mg/kg (n=3)	1.190	0.275	0.413
AC-5mg/kg (n=3)	anti-tip-chimer (n=2)	0.333	0.564	0.700
AC-5mg/kg (n=3)	anti-rsPilA (n=2)	3.000	0.083	0.200
AC-10mg/kg (n=1)	Ofloxacin (n=3)	0.200	0.655	0.737
AC-10mg/kg (n=1)	Saline (n=2)	1.500	0.221	0.361
AC-10mg/kg (n=1)	TS-30mg/kg (n=3)	0.200	0.655	0.737
AC-10mg/kg (n=1)	TS-15mg/kg (n=3)	1.800	0.180	0.341
AC-10mg/kg (n=1)	anti-tip-chimer (n=2)	1.500	0.221	0.361
AC-10mg/kg (n=1)	anti-rsPilA (n=2)	1.500	0.221	0.361
Ofloxacin (n=3)	Saline (n=2)	0.000	1.000	1.000
Ofloxacin (n=3)	TS-30mg/kg (n=3)	0.429	0.513	0.684
Ofloxacin (n=3)	TS-15mg/kg (n=3)	0.429	0.513	0.684
Ofloxacin (n=3)	anti-tip-chimer (n=2)	0.000	1.000	1.000
Ofloxacin (n=3)	anti-rsPilA (n=2)	3.000	0.083	0.200
Saline (n=2)	TS-30mg/kg (n=3)	0.333	0.564	0.700
Saline (n=2)	TS-15mg/kg (n=3)	3.000	0.083	0.200
Saline (n=2)	anti-tip-chimer (n=2)	0.000	1.000	1.000
Saline (n=2)	anti-rsPilA (n=2)	2.400	0.121	0.257
TS-30mg/kg (n=3)	TS-15mg/kg (n=3)	3.857	0.050	0.200
TS-30mg/kg (n=3)	anti-tip-chimer (n=2)	0.000	1.000	1.000
TS-30mg/kg (n=3)	anti-rsPilA (n=2)	3.000	0.083	0.200
TS-15mg/kg (n=3)	anti-tip-chimer (n=2)	3.000	0.083	0.200
TS-15mg/kg (n=3)	anti-rsPilA (n=2)	3.000	0.083	0.200
anti-tip-chimer (n=2)	anti-rsPilA (n=2)	2.400	0.121	0.257

**Kruskal-Wallis (Omnibus Test)**	
**H**	15.126
**p-value**	0.057

Supplementary File 3
Alpha Diversity Output

Fecal Shannon Diversity-9DPT

Kruskal-Wallis pairwise comparisons

Group 1	Group 2	H	p-value	q-value
AC-PO7d (n=2)	AC-5mg/kg (n=3)	0.000	1.000	1.000
AC-PO7d (n=2)	AC-10mg/kg (n=2)	0.000	1.000	1.000
AC-PO7d (n=2)	Ofloxacin (n=3)	0.333	0.564	0.700
AC-PO7d (n=2)	Saline (n=1)	1.500	0.221	0.431
AC-PO7d (n=2)	TS-30mg/kg (n=3)	1.333	0.248	0.431
AC-PO7d (n=2)	TS-15mg/kg (n=3)	0.333	0.564	0.700
AC-PO7d (n=2)	anti-tip-chimer (n=3)	3.000	0.083	0.333
AC-PO7d (n=2)	anti-rsPilA (n=2)	2.400	0.121	0.380
AC-5mg/kg (n=3)	AC-10mg/kg (n=2)	3.000	0.083	0.333
AC-5mg/kg (n=3)	Ofloxacin (n=3)	0.048	0.827	0.876
AC-5mg/kg (n=3)	Saline (n=1)	1.800	0.180	0.431
AC-5mg/kg (n=3)	TS-30mg/kg (n=3)	0.429	0.513	0.700
AC-5mg/kg (n=3)	TS-15mg/kg (n=3)	1.190	0.275	0.431
AC-5mg/kg (n=3)	anti-tip-chimer (n=3)	3.857	0.050	0.333
AC-5mg/kg (n=3)	anti-rsPilA (n=2)	3.000	0.083	0.333
AC-10mg/kg (n=2)	Ofloxacin (n=3)	3.000	0.083	0.333
AC-10mg/kg (n=2)	Saline (n=1)	1.500	0.221	0.431
AC-10mg/kg (n=2)	TS-30mg/kg (n=3)	3.000	0.083	0.333
AC-10mg/kg (n=2)	TS-15mg/kg (n=3)	3.000	0.083	0.333
AC-10mg/kg (n=2)	anti-tip-chimer (n=3)	3.000	0.083	0.333
AC-10mg/kg (n=2)	anti-rsPilA (n=2)	2.400	0.121	0.380
Ofloxacin (n=3)	Saline (n=1)	0.200	0.655	0.737
Ofloxacin (n=3)	TS-30mg/kg (n=3)	0.429	0.513	0.700
Ofloxacin (n=3)	TS-15mg/kg (n=3)	1.190	0.275	0.431
Ofloxacin (n=3)	anti-tip-chimer (n=3)	2.333	0.127	0.380
Ofloxacin (n=3)	anti-rsPilA (n=2)	3.000	0.083	0.333
Saline (n=1)	TS-30mg/kg (n=3)	0.200	0.655	0.737
Saline (n=1)	TS-15mg/kg (n=3)	0.200	0.655	0.737
Saline (n=1)	anti-tip-chimer (n=3)	1.800	0.180	0.431
Saline (n=1)	anti-rsPilA (n=2)	1.500	0.221	0.431
TS-30mg/kg (n=3)	TS-15mg/kg (n=3)	0.048	0.827	0.876
TS-30mg/kg (n=3)	anti-tip-chimer (n=3)	1.190	0.275	0.431
TS-30mg/kg (n=3)	anti-rsPilA (n=2)	1.333	0.248	0.431
TS-15mg/kg (n=3)	anti-tip-chimer (n=3)	1.190	0.275	0.431
TS-15mg/kg (n=3)	anti-rsPilA (n=2)	0.333	0.564	0.700
anti-tip-chimer (n=3)	anti-rsPilA (n=2)	0.333	0.564	0.700

Kruskal-Wallis (Omnibus Test)

**H**	12.281
p-value	0.139

Supplementary File 3
Alpha Diversity Output

Fecal Shannon Diversity--Within-treatment Kruskal-Wallis pairwise comparisons

Treatment	Group 1	Group 2	H	p-value	q-value
AC-PO7d	Baseline (n=3)	2DPT (n=2)	3.000	0.083	0.119
AC-PO7d	Baseline (n=3)	5DPT (n=3)	2.333	0.127	0.141
AC-PO7d	Baseline (n=3)	7DPT (n=3)	3.857	0.050	0.119
AC-PO7d	Baseline (n=3)	9DPT (n=2)	3.000	0.083	0.119
AC-PO7d	2DPT (n=2)	5DPT (n=3)	3.000	0.083	0.119
AC-PO7d	2DPT (n=2)	7DPT (n=3)	3.000	0.083	0.119
AC-PO7d	2DPT (n=2)	9DPT (n=2)	2.400	0.121	0.141
AC-PO7d	5DPT (n=3)	7DPT (n=3)	3.857	0.050	0.119
AC-PO7d	5DPT (n=3)	9DPT (n=2)	0.000	1.000	1.000
AC-PO7d	7DPT (n=3)	9DPT (n=2)	3.000	0.083	0.119

Treatment	Group 1	Group 2	H	p-value	q-value
AC-10mg/kg	Baseline (n=2)	2DPT (n=2)	2.400	0.121	0.202
AC-10mg/kg	Baseline (n=2)	5DPT (n=2)	2.400	0.121	0.202
AC-10mg/kg	Baseline (n=2)	7DPT (n=1)	1.500	0.221	0.221
AC-10mg/kg	Baseline (n=2)	9DPT (n=2)	2.400	0.121	0.202
AC-10mg/kg	2DPT (n=2)	5DPT (n=2)	2.400	0.121	0.202
AC-10mg/kg	2DPT (n=2)	7DPT (n=1)	1.500	0.221	0.221
AC-10mg/kg	2DPT (n=2)	9DPT (n=2)	2.400	0.121	0.202
AC-10mg/kg	5DPT (n=2)	7DPT (n=1)	1.500	0.221	0.221
AC-10mg/kg	5DPT (n=2)	9DPT (n=2)	2.400	0.121	0.202
AC-10mg/kg	7DPT (n=1)	9DPT (n=2)	1.500	0.221	0.221

Treatment	Group 1	Group 2	H	p-value	q-value
AC-5mg/kg	Baseline (n=2)	2DPT (n=2)	0.600	0.439	0.627
AC-5mg/kg	Baseline (n=2)	5DPT (n=3)	3.000	0.083	0.208
AC-5mg/kg	Baseline (n=2)	7DPT (n=3)	3.000	0.083	0.208
AC-5mg/kg	Baseline (n=2)	9DPT (n=3)	1.333	0.248	0.414
AC-5mg/kg	2DPT (n=2)	5DPT (n=3)	3.000	0.083	0.208
AC-5mg/kg	2DPT (n=2)	7DPT (n=3)	0.333	0.564	0.705
AC-5mg/kg	2DPT (n=2)	9DPT (n=3)	0.000	1.000	1.000
AC-5mg/kg	5DPT (n=3)	7DPT (n=3)	2.333	0.127	0.253
AC-5mg/kg	5DPT (n=3)	9DPT (n=3)	3.857	0.050	0.208
AC-5mg/kg	7DPT (n=3)	9DPT (n=3)	0.048	0.827	0.919

Supplementary File 3
Alpha Diversity Output

Fecal Shannon Diversity--Within-treatment Kruskal-Wallis pairwise comparisons

Treatment	Group 1	Group 2	H	p-value	q-value
anti-rsPilA	Baseline (n=3)	2DPT (n=3)	3.857	0.050	0.278
anti-rsPilA	Baseline (n=3)	5DPT (n=2)	1.333	0.248	0.496
anti-rsPilA	Baseline (n=3)	7DPT (n=2)	0.000	1.000	1.000
anti-rsPilA	Baseline (n=3)	9DPT (n=2)	3.000	0.083	0.278
anti-rsPilA	2DPT (n=3)	5DPT (n=2)	0.000	1.000	1.000
anti-rsPilA	2DPT (n=3)	7DPT (n=2)	3.000	0.083	0.278
anti-rsPilA	2DPT (n=3)	9DPT (n=2)	0.333	0.564	0.805
anti-rsPilA	5DPT (n=2)	7DPT (n=2)	0.600	0.439	0.731
anti-rsPilA	5DPT (n=2)	9DPT (n=2)	0.000	1.000	1.000
anti-rsPilA	7DPT (n=2)	9DPT (n=2)	2.400	0.121	0.303

Treatment	Group 1	Group 2	H	p-value	q-value
anti-tip-chimer	Baseline (n=3)	2DPT (n=3)	0.429	0.513	0.805
anti-tip-chimer	Baseline (n=3)	5DPT (n=3)	0.429	0.513	0.805
anti-tip-chimer	Baseline (n=3)	7DPT (n=2)	0.333	0.564	0.805
anti-tip-chimer	Baseline (n=3)	9DPT (n=3)	0.429	0.513	0.805
anti-tip-chimer	2DPT (n=3)	5DPT (n=3)	0.048	0.827	1.000
anti-tip-chimer	2DPT (n=3)	7DPT (n=2)	0.000	1.000	1.000
anti-tip-chimer	2DPT (n=3)	9DPT (n=3)	2.333	0.127	0.805
anti-tip-chimer	5DPT (n=3)	7DPT (n=2)	0.000	1.000	1.000
anti-tip-chimer	5DPT (n=3)	9DPT (n=3)	1.190	0.275	0.805
anti-tip-chimer	7DPT (n=2)	9DPT (n=3)	1.333	0.248	0.805

Treatment	Group 1	Group 2	H	p-value	q-value
Ofloxacin	Baseline (n=3)	2DPT (n=2)	1.333	0.248	0.827
Ofloxacin	Baseline (n=3)	5DPT (n=3)	0.048	0.827	0.827
Ofloxacin	Baseline (n=3)	7DPT (n=3)	0.048	0.827	0.827
Ofloxacin	Baseline (n=3)	9DPT (n=3)	0.429	0.513	0.827
Ofloxacin	2DPT (n=2)	5DPT (n=3)	1.333	0.248	0.827
Ofloxacin	2DPT (n=2)	7DPT (n=3)	0.333	0.564	0.827
Ofloxacin	2DPT (n=2)	9DPT (n=3)	0.333	0.564	0.827
Ofloxacin	5DPT (n=3)	7DPT (n=3)	0.048	0.827	0.827
Ofloxacin	5DPT (n=3)	9DPT (n=3)	0.048	0.827	0.827
Ofloxacin	7DPT (n=3)	9DPT (n=3)	0.048	0.827	0.827

Supplementary File 3
Alpha Diversity Output

Fecal Shannon Diversity--Within-treatment Kruskal-Wallis pairwise comparisons

Treatment	Group 1	Group 2	H	p-value	q-value
TS-30mg/kg	Baseline (n=3)	2DPT (n=3)	3.857	0.050	0.124
TS-30mg/kg	Baseline (n=3)	5DPT (n=3)	3.857	0.050	0.124
TS-30mg/kg	Baseline (n=3)	7DPT (n=3)	3.857	0.050	0.124
TS-30mg/kg	Baseline (n=3)	9DPT (n=3)	3.857	0.050	0.124
TS-30mg/kg	2DPT (n=3)	5DPT (n=3)	0.048	0.827	0.827
TS-30mg/kg	2DPT (n=3)	7DPT (n=3)	0.048	0.827	0.827
TS-30mg/kg	2DPT (n=3)	9DPT (n=3)	0.048	0.827	0.827
TS-30mg/kg	5DPT (n=3)	7DPT (n=3)	0.429	0.513	0.827
TS-30mg/kg	5DPT (n=3)	9DPT (n=3)	0.048	0.827	0.827
TS-30mg/kg	7DPT (n=3)	9DPT (n=3)	0.429	0.513	0.827

Treatment	Group 1	Group 2	H	p-value	q-value
TS-15mg/kg	Baseline (n=3)	2DPT (n=3)	3.857	0.050	0.099
TS-15mg/kg	Baseline (n=3)	5DPT (n=3)	3.857	0.050	0.099
TS-15mg/kg	Baseline (n=3)	7DPT (n=3)	3.857	0.050	0.099
TS-15mg/kg	Baseline (n=3)	9DPT (n=3)	2.333	0.127	0.181
TS-15mg/kg	2DPT (n=3)	5DPT (n=3)	0.048	0.827	0.827
TS-15mg/kg	2DPT (n=3)	7DPT (n=3)	2.333	0.127	0.181
TS-15mg/kg	2DPT (n=3)	9DPT (n=3)	0.048	0.827	0.827
TS-15mg/kg	5DPT (n=3)	7DPT (n=3)	3.857	0.050	0.099
TS-15mg/kg	5DPT (n=3)	9DPT (n=3)	1.190	0.275	0.344
TS-15mg/kg	7DPT (n=3)	9DPT (n=3)	3.857	0.050	0.099

Treatment	Group 1	Group 2	H	p-value	q-value
Saline	Baseline (n=3)	2DPT (n=3)	0.429	0.513	0.818
Saline	Baseline (n=3)	5DPT (n=3)	0.429	0.513	0.818
Saline	Baseline (n=3)	7DPT (n=2)	0.333	0.564	0.818
Saline	Baseline (n=3)	9DPT (n=1)	0.200	0.655	0.818
Saline	2DPT (n=3)	5DPT (n=3)	0.429	0.513	0.818
Saline	2DPT (n=3)	7DPT (n=2)	0.000	1.000	1.000
Saline	2DPT (n=3)	9DPT (n=1)	0.200	0.655	0.818
Saline	5DPT (n=3)	7DPT (n=2)	0.333	0.564	0.818
Saline	5DPT (n=3)	9DPT (n=1)	0.200	0.655	0.818
Saline	7DPT (n=2)	9DPT (n=1)	0.000	1.000	1.000

Supplementary File 3
Alpha Diversity Output

id	Chinchilla_ID	Treatment	Sex	Collection	NPL pielou_evenness
1BN	Chinch1	AC-PO7d	F	Baseline	0.815697371
2BN	Chinch2	AC-PO7d	F	Baseline	0.897308001
3BN	Chinch3	AC-PO7d	M	Baseline	0.824644982
20NBL	Chinch20	AC-5mg/kg	F	Baseline	0.898680715
23NBL	Chinch23	AC-5mg/kg	F	Baseline	0.936459277
26NBL	Chinch26	AC-5mg/kg	M	Baseline	0.930558129
22NBL	Chinch22	AC-10mg/kg	M	Baseline	0.928196796
25NBL	Chinch25	AC-10mg/kg	F	Baseline	0.945128527
11NBL	Chinch11	anti-rsPilA	F	Baseline	0.95797858
13NBL	Chinch13	anti-rsPilA	F	Baseline	0.953263114
17NBL	Chinch17	anti-rsPilA	F	Baseline	0.931684368
19NBL	Chinch19	anti-tip-chimer	F	Baseline	0.950252508
21NBL	Chinch21	anti-tip-chimer	F	Baseline	0.938486149
27NBL	Chinch27	anti-tip-chimer	M	Baseline	0.931846713
4NBL	Chinch4	Ofloxacin	F	Baseline	0.958167831
5NBL	Chinch5	Ofloxacin	F	Baseline	0.965281651
7NBL	Chinch7	Ofloxacin	M	Baseline	0.933341319
14NBL	Chinch14	TS-15mg/kg	F	Baseline	0.931963053
15NBL	Chinch15	TS-15mg/kg	F	Baseline	0.950089443
18NBL	Chinch18	TS-15mg/kg	M	Baseline	0.920836802
10NBL	Chinch10	TS-30mg/kg	F	Baseline	0.937854864
12NBL	Chinch12	TS-30mg/kg	M	Baseline	0.944501134
16NBL	Chinch16	TS-30mg/kg	F	Baseline	0.916996684
6NBL	Chinch6	Saline	F	Baseline	0.883796327
8NBL	Chinch8	Saline	M	Baseline	0.959950934
9NBL	Chinch9	Saline	F	Baseline	0.933038898
1AN2	Chinch1	AC-PO7d	F	1	0.90095409
2AN2	Chinch2	AC-PO7d	F	1	0.83885826
3AN2	Chinch3	AC-PO7d	M	1	0.865965961
20N1	Chinch20	AC-5mg/kg	F	1	0.885069375
23N1	Chinch23	AC-5mg/kg	F	1	0.917264178
26N1	Chinch26	AC-5mg/kg	M	1	0.903285752
22N1	Chinch22	AC-10mg/kg	M	1	0.945557251
25N1	Chinch25	AC-10mg/kg	F	1	0.93157203
11N1	Chinch11	anti-rsPilA	F	1	0.95435933
13N1	Chinch13	anti-rsPilA	F	1	0.926727653
17N1	Chinch17	anti-rsPilA	F	1	0.940554966
19N1	Chinch19	anti-tip-chimer	F	1	0.94978915
21N1	Chinch21	anti-tip-chimer	F	1	0.942007305
27N1	Chinch27	anti-tip-chimer	M	1	0.929456473
4N1	Chinch4	Ofloxacin	F	1	0.942121356
5N1	Chinch5	Ofloxacin	F	1	0.929295556
7N1	Chinch7	Ofloxacin	M	1	0.915192009
14N1	Chinch14	TS-15mg/kg	F	1	0.957173528
15N1	Chinch15	TS-15mg/kg	F	1	0.962571483
18N1	Chinch18	TS-15mg/kg	M	1	0.910928079
10N1	Chinch10	TS-30mg/kg	F	1	0.924299911

Supplementary File 3
Alpha Diversity Output

id	Chinchilla_ID	Treatment	Sex	Collection	NPL pielou_evenness
12N1	Chinch12	TS-30mg/kg	M	1	0.926427813
16N1	Chinch16	TS-30mg/kg	F	1	0.934982881
6N1	Chinch6	Saline	F	1	0.915854742
8N1	Chinch8	Saline	M	1	0.944299235
9N1	Chinch9	Saline	F	1	0.931398006
1AN5	Chinch1	AC-PO7d	F	2	0.913509967
2AN5	Chinch2	AC-PO7d	F	2	0.924678146
3AN5	Chinch3	AC-PO7d	M	2	0.750413651
20N2	Chinch20	AC-5mg/kg	F	2	0.862723391
23N2	Chinch23	AC-5mg/kg	F	2	0.93492213
26N2	Chinch26	AC-5mg/kg	M	2	0.938955197
22N2	Chinch22	AC-10mg/kg	M	2	0.944334509
25N2	Chinch25	AC-10mg/kg	F	2	0.906978797
11N2	Chinch11	anti-rsPilA	F	2	0.937215154
17N2	Chinch17	anti-rsPilA	F	2	0.957035822
19N2	Chinch19	anti-tip-chimer	F	2	0.957837217
21N2	Chinch21	anti-tip-chimer	F	2	0.948184908
27N2	Chinch27	anti-tip-chimer	M	2	0.913913937
4N2	Chinch4	Ofloxacin	F	2	0.956244022
5N2	Chinch5	Ofloxacin	F	2	0.960949138
7N2	Chinch7	Ofloxacin	M	2	0.926324855
14N2	Chinch14	TS-15mg/kg	F	2	0.945989159
15N2	Chinch15	TS-15mg/kg	F	2	0.955806621
18N2	Chinch18	TS-15mg/kg	M	2	0.906307862
10N2	Chinch10	TS-30mg/kg	F	2	0.939909357
12N2	Chinch12	TS-30mg/kg	M	2	0.924205003
16N2	Chinch16	TS-30mg/kg	F	2	0.945910963
6N2	Chinch6	Saline	F	2	0.908496961
8N2	Chinch8	Saline	M	2	0.953470076
9N2	Chinch9	Saline	F	2	0.900742675
1AN7	Chinch1	AC-PO7d	F	3	0.792461764
2AN7	Chinch2	AC-PO7d	F	3	0.672876442
20N3	Chinch20	AC-5mg/kg	F	3	0.888019847
23N3	Chinch23	AC-5mg/kg	F	3	0.927849412
26N3	Chinch26	AC-5mg/kg	M	3	0.915590689
25N3	Chinch25	AC-10mg/kg	F	3	0.920449792
11N3	Chinch11	anti-rsPilA	F	3	0.910604071
17N3	Chinch17	anti-rsPilA	F	3	0.959275493
21N3	Chinch21	anti-tip-chimer	F	3	0.930651276
27N3	Chinch27	anti-tip-chimer	M	3	0.917187875
4N3	Chinch4	Ofloxacin	F	3	0.952734689
5N3	Chinch5	Ofloxacin	F	3	0.878559208
7N3	Chinch7	Ofloxacin	M	3	0.928146413
14N3	Chinch14	TS-15mg/kg	F	3	0.940367103
15N3	Chinch15	TS-15mg/kg	F	3	0.95966481
18N3	Chinch18	TS-15mg/kg	M	3	0.892579179
10N3	Chinch10	TS-30mg/kg	F	3	0.938363691
16N3	Chinch16	TS-30mg/kg	F	3	0.966009098

Supplementary File 3
Alpha Diversity Output

id	Chinchilla_ID	Treatment	Sex	Collection	NPL pielou_evenness
6N3	Chinch6	Saline	F	3	0.896292449
8N3	Chinch8	Saline	M	3	0.963307769
9N3	Chinch9	Saline	F	3	0.811091773
2AN9	Chinch2	AC-PO7d	F	4	0.743701445
3AN9	Chinch3	AC-PO7d	M	4	0.78468136
20N4	Chinch20	AC-5mg/kg	F	4	0.847442281
23N4	Chinch23	AC-5mg/kg	F	4	0.917987496
26N4	Chinch26	AC-5mg/kg	M	4	0.892424766
22N4	Chinch22	AC-10mg/kg	M	4	0.850131359
25N4	Chinch25	AC-10mg/kg	F	4	0.92000355
11N4	Chinch11	anti-rsPilA	F	4	0.921193837
17N4	Chinch17	anti-rsPilA	F	4	0.943252064
19N4	Chinch19	anti-tip-chimer	F	4	0.953692949
21N4	Chinch21	anti-tip-chimer	F	4	0.958600354
27N4	Chinch27	anti-tip-chimer	M	4	0.920964743
5N4	Chinch5	Ofloxacin	F	4	0.845561902
7N4	Chinch7	Ofloxacin	M	4	0.934221147
14N4	Chinch14	TS-15mg/kg	F	4	0.934386414
15N4	Chinch15	TS-15mg/kg	F	4	0.951265136
18N4	Chinch18	TS-15mg/kg	M	4	0.907876597
10N4	Chinch10	TS-30mg/kg	F	4	0.917792967
12N4	Chinch12	TS-30mg/kg	M	4	0.925726394
16N4	Chinch16	TS-30mg/kg	F	4	0.962933726
6N4	Chinch6	Saline	F	4	0.910012769
8N4	Chinch8	Saline	M	4	0.942980374

Supplementary File 3
Alpha Diversity Output

NPL Evenness-Baseline

Kruskal-Wallis pairwise comparisons

Group 1	Group 2	H	p-value	q-value
AC-PO7d (n=3)	AC-5mg/kg (n=3)	3.857	0.050	0.297
AC-PO7d (n=3)	AC-10mg/kg (n=2)	3.000	0.083	0.380
AC-PO7d (n=3)	Ofloxacin (n=3)	3.857	0.050	0.297
AC-PO7d (n=3)	Saline (n=3)	2.333	0.127	0.380
AC-PO7d (n=3)	TS-30mg/kg (n=3)	3.857	0.050	0.297
AC-PO7d (n=3)	TS-15mg/kg (n=3)	3.857	0.050	0.297
AC-PO7d (n=3)	anti-tip-chimer (n=3)	3.857	0.050	0.297
AC-PO7d (n=3)	anti-rsPilA (n=3)	3.857	0.050	0.297
AC-5mg/kg (n=3)	AC-10mg/kg (n=2)	0.333	0.564	0.725
AC-5mg/kg (n=3)	Ofloxacin (n=3)	2.333	0.127	0.380
AC-5mg/kg (n=3)	Saline (n=3)	0.048	0.827	0.876
AC-5mg/kg (n=3)	TS-30mg/kg (n=3)	1.190	0.275	0.472
AC-5mg/kg (n=3)	TS-15mg/kg (n=3)	0.429	0.513	0.725
AC-5mg/kg (n=3)	anti-tip-chimer (n=3)	2.333	0.127	0.380
AC-5mg/kg (n=3)	anti-rsPilA (n=3)	2.333	0.127	0.380
AC-10mg/kg (n=2)	Ofloxacin (n=3)	1.333	0.248	0.472
AC-10mg/kg (n=2)	Saline (n=3)	0.000	1.000	1.000
AC-10mg/kg (n=2)	TS-30mg/kg (n=3)	0.333	0.564	0.725
AC-10mg/kg (n=2)	TS-15mg/kg (n=3)	0.000	1.000	1.000
AC-10mg/kg (n=2)	anti-tip-chimer (n=3)	0.333	0.564	0.725
AC-10mg/kg (n=2)	anti-rsPilA (n=3)	1.333	0.248	0.472
Ofloxacin (n=3)	Saline (n=3)	1.190	0.275	0.472
Ofloxacin (n=3)	TS-30mg/kg (n=3)	1.190	0.275	0.472
Ofloxacin (n=3)	TS-15mg/kg (n=3)	2.333	0.127	0.380
Ofloxacin (n=3)	anti-tip-chimer (n=3)	1.190	0.275	0.472
Ofloxacin (n=3)	anti-rsPilA (n=3)	1.190	0.275	0.472
Saline (n=3)	TS-30mg/kg (n=3)	0.048	0.827	0.876
Saline (n=3)	TS-15mg/kg (n=3)	0.048	0.827	0.876
Saline (n=3)	anti-tip-chimer (n=3)	0.048	0.827	0.876
Saline (n=3)	anti-rsPilA (n=3)	0.048	0.827	0.876
TS-30mg/kg (n=3)	TS-15mg/kg (n=3)	0.048	0.827	0.876
TS-30mg/kg (n=3)	anti-tip-chimer (n=3)	0.429	0.513	0.725
TS-30mg/kg (n=3)	anti-rsPilA (n=3)	1.190	0.275	0.472
TS-15mg/kg (n=3)	anti-tip-chimer (n=3)	0.429	0.513	0.725
TS-15mg/kg (n=3)	anti-rsPilA (n=3)	1.190	0.275	0.472
anti-tip-chimer (n=3)	anti-rsPilA (n=3)	0.429	0.513	0.725

Kruskal-Wallis (Omnibus Test)

**H**	11.920
**p-value**	0.155

Supplementary File 3
Alpha Diversity Output

NPL Evenness-2DPT

Kruskal-Wallis pairwise comparisons

Group 1	Group 2	H	p-value	q-value
AC-PO7d (n=3)	AC-5mg/kg (n=3)	2.333	0.127	0.268
AC-PO7d (n=3)	AC-10mg/kg (n=2)	3.000	0.083	0.268
AC-PO7d (n=3)	Ofloxacin (n=3)	3.857	0.050	0.198
AC-PO7d (n=3)	Saline (n=3)	3.857	0.050	0.198
AC-PO7d (n=3)	TS-30mg/kg (n=3)	3.857	0.050	0.198
AC-PO7d (n=3)	TS-15mg/kg (n=3)	3.857	0.050	0.198
AC-PO7d (n=3)	anti-tip-chimer (n=3)	3.857	0.050	0.198
AC-PO7d (n=3)	anti-rsPilA (n=3)	3.857	0.050	0.198
AC-5mg/kg (n=3)	AC-10mg/kg (n=2)	3.000	0.083	0.268
AC-5mg/kg (n=3)	Ofloxacin (n=3)	2.333	0.127	0.268
AC-5mg/kg (n=3)	Saline (n=3)	2.333	0.127	0.268
AC-5mg/kg (n=3)	TS-30mg/kg (n=3)	3.857	0.050	0.198
AC-5mg/kg (n=3)	TS-15mg/kg (n=3)	2.333	0.127	0.268
AC-5mg/kg (n=3)	anti-tip-chimer (n=3)	3.857	0.050	0.198
AC-5mg/kg (n=3)	anti-rsPilA (n=3)	3.857	0.050	0.198
AC-10mg/kg (n=2)	Ofloxacin (n=3)	1.333	0.248	0.447
AC-10mg/kg (n=2)	Saline (n=3)	1.333	0.248	0.447
AC-10mg/kg (n=2)	TS-30mg/kg (n=3)	1.333	0.248	0.447
AC-10mg/kg (n=2)	TS-15mg/kg (n=3)	0.333	0.564	0.655
AC-10mg/kg (n=2)	anti-tip-chimer (n=3)	0.000	1.000	1.000
AC-10mg/kg (n=2)	anti-rsPilA (n=3)	0.000	1.000	1.000
Ofloxacin (n=3)	Saline (n=3)	0.429	0.513	0.615
Ofloxacin (n=3)	TS-30mg/kg (n=3)	0.048	0.827	0.876
Ofloxacin (n=3)	TS-15mg/kg (n=3)	0.429	0.513	0.615
Ofloxacin (n=3)	anti-tip-chimer (n=3)	1.190	0.275	0.472
Ofloxacin (n=3)	anti-rsPilA (n=3)	0.429	0.513	0.615
Saline (n=3)	TS-30mg/kg (n=3)	0.048	0.827	0.876
Saline (n=3)	TS-15mg/kg (n=3)	0.429	0.513	0.615
Saline (n=3)	anti-tip-chimer (n=3)	0.429	0.513	0.615
Saline (n=3)	anti-rsPilA (n=3)	0.429	0.513	0.615
TS-30mg/kg (n=3)	TS-15mg/kg (n=3)	0.429	0.513	0.615
TS-30mg/kg (n=3)	anti-tip-chimer (n=3)	2.333	0.127	0.268
TS-30mg/kg (n=3)	anti-rsPilA (n=3)	2.333	0.127	0.268
TS-15mg/kg (n=3)	anti-tip-chimer (n=3)	0.429	0.513	0.615
TS-15mg/kg (n=3)	anti-rsPilA (n=3)	0.429	0.513	0.615
anti-tip-chimer (n=3)	anti-rsPilA (n=3)	0.048	0.827	0.876

Kruskal-Wallis (Omnibus Test)

**H**	14.641
**p-value**	0.067

Supplementary File 3
Alpha Diversity Output

NPL Evenness-5DPT

Kruskal-Wallis pairwise comparisons

**Group 1**	Group 2	H	p-value	q-value
AC-PO7d (n=3)	AC-5mg/kg (n=3)	1.190	0.275	0.550
AC-PO7d (n=3)	AC-10mg/kg (n=2)	0.333	0.564	0.655
AC-PO7d (n=3)	Ofloxacin (n=3)	3.857	0.050	0.550
AC-PO7d (n=3)	Saline (n=3)	0.048	0.827	0.902
AC-PO7d (n=3)	TS-30mg/kg (n=3)	2.333	0.127	0.550
AC-PO7d (n=3)	TS-15mg/kg (n=3)	1.190	0.275	0.550
AC-PO7d (n=3)	anti-tip-chimer (n=3)	2.333	0.127	0.550
AC-PO7d (n=3)	anti-rsPilA (n=2)	3.000	0.083	0.550
AC-5mg/kg (n=3)	AC-10mg/kg (n=2)	0.333	0.564	0.655
AC-5mg/kg (n=3)	Ofloxacin (n=3)	1.190	0.275	0.550
AC-5mg/kg (n=3)	Saline (n=3)	0.048	0.827	0.902
AC-5mg/kg (n=3)	TS-30mg/kg (n=3)	1.190	0.275	0.550
AC-5mg/kg (n=3)	TS-15mg/kg (n=3)	1.190	0.275	0.550
AC-5mg/kg (n=3)	anti-tip-chimer (n=3)	1.190	0.275	0.550
AC-5mg/kg (n=3)	anti-rsPilA (n=2)	1.333	0.248	0.550
AC-10mg/kg (n=2)	Ofloxacin (n=3)	1.333	0.248	0.550
AC-10mg/kg (n=2)	Saline (n=3)	0.000	1.000	1.000
AC-10mg/kg (n=2)	TS-30mg/kg (n=3)	0.333	0.564	0.655
AC-10mg/kg (n=2)	TS-15mg/kg (n=3)	0.333	0.564	0.655
AC-10mg/kg (n=2)	anti-tip-chimer (n=3)	1.333	0.248	0.550
AC-10mg/kg (n=2)	anti-rsPilA (n=2)	0.600	0.439	0.655
Ofloxacin (n=3)	Saline (n=3)	2.333	0.127	0.550
Ofloxacin (n=3)	TS-30mg/kg (n=3)	1.190	0.275	0.550
Ofloxacin (n=3)	TS-15mg/kg (n=3)	1.190	0.275	0.550
Ofloxacin (n=3)	anti-tip-chimer (n=3)	0.429	0.513	0.655
Ofloxacin (n=3)	anti-rsPilA (n=2)	0.000	1.000	1.000
Saline (n=3)	TS-30mg/kg (n=3)	0.429	0.513	0.655
Saline (n=3)	TS-15mg/kg (n=3)	0.429	0.513	0.655
Saline (n=3)	anti-tip-chimer (n=3)	1.190	0.275	0.550
Saline (n=3)	anti-rsPilA (n=2)	1.333	0.248	0.550
TS-30mg/kg (n=3)	TS-15mg/kg (n=3)	0.429	0.513	0.655
TS-30mg/kg (n=3)	anti-tip-chimer (n=3)	0.429	0.513	0.655
TS-30mg/kg (n=3)	anti-rsPilA (n=2)	0.333	0.564	0.655
TS-15mg/kg (n=3)	anti-tip-chimer (n=3)	0.429	0.513	0.655
TS-15mg/kg (n=3)	anti-rsPilA (n=2)	0.333	0.564	0.655
anti-tip-chimer (n=3)	anti-rsPilA (n=2)	0.000	1.000	1.000

Kruskal-Wallis (Omnibus Test)

**H**	8.458
**p-value**	0.390

Supplementary File 3
Alpha Diversity Output

NPL Evenness-7DPT

Kruskal-Wallis pairwise comparisons

Group 1	Group 2	H	p-value	q-value
AC-PO7d (n=2)	AC-5mg/kg (n=3)	3.000	0.083	0.485
AC-PO7d (n=2)	AC-10mg/kg (n=1)	1.500	0.221	0.638
AC-PO7d (n=2)	Ofloxacin (n=3)	3.000	0.083	0.485
AC-PO7d (n=2)	Saline (n=3)	3.000	0.083	0.485
AC-PO7d (n=2)	TS-30mg/kg (n=2)	2.400	0.121	0.485
AC-PO7d (n=2)	TS-15mg/kg (n=3)	3.000	0.083	0.485
AC-PO7d (n=2)	anti-tip-chimer (n=2)	2.400	0.121	0.485
AC-PO7d (n=2)	anti-rsPilA (n=2)	2.400	0.121	0.485
AC-5mg/kg (n=3)	AC-10mg/kg (n=1)	0.200	0.655	0.842
AC-5mg/kg (n=3)	Ofloxacin (n=3)	0.429	0.513	0.842
AC-5mg/kg (n=3)	Saline (n=3)	0.048	0.827	0.961
AC-5mg/kg (n=3)	TS-30mg/kg (n=2)	3.000	0.083	0.485
AC-5mg/kg (n=3)	TS-15mg/kg (n=3)	1.190	0.275	0.661
AC-5mg/kg (n=3)	anti-tip-chimer (n=2)	1.333	0.248	0.638
AC-5mg/kg (n=3)	anti-rsPilA (n=2)	0.333	0.564	0.842
AC-10mg/kg (n=1)	Ofloxacin (n=3)	0.200	0.655	0.842
AC-10mg/kg (n=1)	Saline (n=3)	0.200	0.655	0.842
AC-10mg/kg (n=1)	TS-30mg/kg (n=2)	1.500	0.221	0.638
AC-10mg/kg (n=1)	TS-15mg/kg (n=3)	0.200	0.655	0.842
AC-10mg/kg (n=1)	anti-tip-chimer (n=2)	0.000	1.000	1.000
AC-10mg/kg (n=1)	anti-rsPilA (n=2)	0.000	1.000	1.000
Ofloxacin (n=3)	Saline (n=3)	0.048	0.827	0.961
Ofloxacin (n=3)	TS-30mg/kg (n=2)	1.333	0.248	0.638
Ofloxacin (n=3)	TS-15mg/kg (n=3)	0.429	0.513	0.842
Ofloxacin (n=3)	anti-tip-chimer (n=2)	0.000	1.000	1.000
Ofloxacin (n=3)	anti-rsPilA (n=2)	0.333	0.564	0.842
Saline (n=3)	TS-30mg/kg (n=2)	1.333	0.248	0.638
Saline (n=3)	TS-15mg/kg (n=3)	0.048	0.827	0.961
Saline (n=3)	anti-tip-chimer (n=2)	0.333	0.564	0.842
Saline (n=3)	anti-rsPilA (n=2)	0.333	0.564	0.842
TS-30mg/kg (n=2)	TS-15mg/kg (n=3)	0.333	0.564	0.842
TS-30mg/kg (n=2)	anti-tip-chimer (n=2)	2.400	0.121	0.485
TS-30mg/kg (n=2)	anti-rsPilA (n=2)	0.600	0.439	0.842
TS-15mg/kg (n=3)	anti-tip-chimer (n=2)	0.333	0.564	0.842
TS-15mg/kg (n=3)	anti-rsPilA (n=2)	0.000	1.000	1.000
anti-tip-chimer (n=2)	anti-rsPilA (n=2)	0.000	1.000	1.000

Kruskal-Wallis (Omnibus Test)

**H**	8.558
**p-value**	0.381

Supplementary File 3
Alpha Diversity Output

NPL Evenness-9DPT

Kruskal-Wallis pairwise comparisons

Group 1	Group 2	H	p-value	q-value
AC-PO7d (n=2)	AC-5mg/kg (n=3)	3.000	0.083	0.326
AC-PO7d (n=2)	AC-10mg/kg (n=2)	2.400	0.121	0.326
AC-PO7d (n=2)	Ofloxacin (n=2)	2.400	0.121	0.326
AC-PO7d (n=2)	Saline (n=2)	2.400	0.121	0.326
AC-PO7d (n=2)	TS-30mg/kg (n=3)	3.000	0.083	0.326
AC-PO7d (n=2)	TS-15mg/kg (n=3)	3.000	0.083	0.326
AC-PO7d (n=2)	anti-tip-chimer (n=3)	3.000	0.083	0.326
AC-PO7d (n=2)	anti-rsPilA (n=2)	2.400	0.121	0.326
AC-5mg/kg (n=3)	AC-10mg/kg (n=2)	0.333	0.564	0.700
AC-5mg/kg (n=3)	Ofloxacin (n=2)	0.000	1.000	1.000
AC-5mg/kg (n=3)	Saline (n=2)	1.333	0.248	0.447
AC-5mg/kg (n=3)	TS-30mg/kg (n=3)	2.333	0.127	0.326
AC-5mg/kg (n=3)	TS-15mg/kg (n=3)	2.333	0.127	0.326
AC-5mg/kg (n=3)	anti-tip-chimer (n=3)	3.857	0.050	0.326
AC-5mg/kg (n=3)	anti-rsPilA (n=2)	3.000	0.083	0.326
AC-10mg/kg (n=2)	Ofloxacin (n=2)	0.000	1.000	1.000
AC-10mg/kg (n=2)	Saline (n=2)	0.600	0.439	0.632
AC-10mg/kg (n=2)	TS-30mg/kg (n=3)	1.333	0.248	0.447
AC-10mg/kg (n=2)	TS-15mg/kg (n=3)	1.333	0.248	0.447
AC-10mg/kg (n=2)	anti-tip-chimer (n=3)	3.000	0.083	0.326
AC-10mg/kg (n=2)	anti-rsPilA (n=2)	2.400	0.121	0.326
Ofloxacin (n=2)	Saline (n=2)	0.600	0.439	0.632
Ofloxacin (n=2)	TS-30mg/kg (n=3)	0.333	0.564	0.700
Ofloxacin (n=2)	TS-15mg/kg (n=3)	1.333	0.248	0.447
Ofloxacin (n=2)	anti-tip-chimer (n=3)	1.333	0.248	0.447
Ofloxacin (n=2)	anti-rsPilA (n=2)	0.600	0.439	0.632
Saline (n=2)	TS-30mg/kg (n=3)	0.333	0.564	0.700
Saline (n=2)	TS-15mg/kg (n=3)	0.000	1.000	1.000
Saline (n=2)	anti-tip-chimer (n=3)	1.333	0.248	0.447
Saline (n=2)	anti-rsPilA (n=2)	0.600	0.439	0.632
TS-30mg/kg (n=3)	TS-15mg/kg (n=3)	0.048	0.827	0.961
TS-30mg/kg (n=3)	anti-tip-chimer (n=3)	0.048	0.827	0.961
TS-30mg/kg (n=3)	anti-rsPilA (n=2)	0.000	1.000	1.000
TS-15mg/kg (n=3)	anti-tip-chimer (n=3)	1.190	0.275	0.472
TS-15mg/kg (n=3)	anti-rsPilA (n=2)	0.000	1.000	1.000
anti-tip-chimer (n=3)	anti-rsPilA (n=2)	0.333	0.564	0.700

Kruskal-Wallis (Omnibus Test)

**H**	12.111
p-value	0.146

Supplementary File 3
Alpha Diversity Output

NPL Evenness--Within-treatment Kruskal-Wallis pairwise comparisons

Treatment	Group 1	Group 2	H	p-value	q-value
AC-PO7d	0 (n=3)	2 (n=3)	1.190	0.275	0.393
AC-PO7d	0 (n=3)	5 (n=3)	0.429	0.513	0.570
AC-PO7d	0 (n=3)	7 (n=2)	3.000	0.083	0.208
AC-PO7d	0 (n=3)	9 (n=2)	3.000	0.083	0.208
AC-PO7d	2 (n=3)	5 (n=3)	0.429	0.513	0.570
AC-PO7d	2 (n=3)	7 (n=2)	3.000	0.083	0.208
AC-PO7d	2 (n=3)	9 (n=2)	3.000	0.083	0.208
AC-PO7d	5 (n=3)	7 (n=2)	1.333	0.248	0.393
AC-PO7d	5 (n=3)	9 (n=2)	1.333	0.248	0.393
AC-PO7d	7 (n=2)	9 (n=2)	0.000	1.000	1.000

Treatment	Group 1	Group 2	H	p-value	q-value
AC-10mg/kg	0 (n=2)	2 (n=2)	0.600	0.439	0.487
AC-10mg/kg	0 (n=2)	5 (n=2)	0.600	0.439	0.487
AC-10mg/kg	0 (n=2)	7 (n=1)	1.500	0.221	0.441
AC-10mg/kg	0 (n=2)	9 (n=2)	2.400	0.121	0.441
AC-10mg/kg	2 (n=2)	5 (n=2)	0.600	0.439	0.487
AC-10mg/kg	2 (n=2)	7 (n=1)	1.500	0.221	0.441
AC-10mg/kg	2 (n=2)	9 (n=2)	2.400	0.121	0.441
AC-10mg/kg	5 (n=2)	7 (n=1)	0.000	1.000	1.000
AC-10mg/kg	5 (n=2)	9 (n=2)	0.600	0.439	0.487
AC-10mg/kg	7 (n=1)	9 (n=2)	1.500	0.221	0.441

Treatment	Group 1	Group 2	H	p-value	q-value
AC-5mg/kg	0 (n=3)	2 (n=3)	1.190	0.275	0.641
AC-5mg/kg	0 (n=3)	5 (n=3)	0.048	0.827	0.827
AC-5mg/kg	0 (n=3)	7 (n=3)	1.190	0.275	0.641
AC-5mg/kg	0 (n=3)	9 (n=3)	2.333	0.127	0.641
AC-5mg/kg	2 (n=3)	5 (n=3)	0.429	0.513	0.641
AC-5mg/kg	2 (n=3)	7 (n=3)	0.429	0.513	0.641
AC-5mg/kg	2 (n=3)	9 (n=3)	0.048	0.827	0.827
AC-5mg/kg	5 (n=3)	7 (n=3)	0.429	0.513	0.641
AC-5mg/kg	5 (n=3)	9 (n=3)	1.190	0.275	0.641
AC-5mg/kg	7 (n=3)	9 (n=3)	0.429	0.513	0.641

Supplementary File 3
Alpha Diversity Output

NPL Evenness--Within-treatment Kruskal-Wallis pairwise comparisons

Treatment	Group 1	Group 2	H	p-value	q-value
anti-rsPilA	0 (n=3)	2 (n=3)	0.429	0.513	1.000
anti-rsPilA	0 (n=3)	5 (n=2)	0.000	1.000	1.000
anti-rsPilA	0 (n=3)	7 (n=2)	0.000	1.000	1.000
anti-rsPilA	0 (n=3)	9 (n=2)	1.333	0.248	1.000
anti-rsPilA	2 (n=3)	5 (n=2)	0.333	0.564	1.000
anti-rsPilA	2 (n=3)	7 (n=2)	0.000	1.000	1.000
anti-rsPilA	2 (n=3)	9 (n=2)	0.333	0.564	1.000
anti-rsPilA	5 (n=2)	7 (n=2)	0.000	1.000	1.000
anti-rsPilA	5 (n=2)	9 (n=2)	0.600	0.439	1.000
anti-rsPilA	7 (n=2)	9 (n=2)	0.000	1.000	1.000

Treatment	Group 1	Group 2	H	p-value	q-value
anti-tip-chimer	0 (n=3)	2 (n=3)	0.048	0.827	0.827
anti-tip-chimer	0 (n=3)	5 (n=3)	0.048	0.827	0.827
anti-tip-chimer	0 (n=3)	7 (n=2)	3.000	0.083	0.805
anti-tip-chimer	0 (n=3)	9 (n=3)	0.429	0.513	0.805
anti-tip-chimer	2 (n=3)	5 (n=3)	0.048	0.827	0.827
anti-tip-chimer	2 (n=3)	7 (n=2)	1.333	0.248	0.805
anti-tip-chimer	2 (n=3)	9 (n=3)	0.429	0.513	0.805
anti-tip-chimer	5 (n=3)	7 (n=2)	0.333	0.564	0.805
anti-tip-chimer	5 (n=3)	9 (n=3)	0.429	0.513	0.805
anti-tip-chimer	7 (n=2)	9 (n=3)	1.333	0.248	0.805

Treatment	Group 1	Group 2	H	p-value	q-value
Ofloxacin	0 (n=3)	2 (n=3)	2.333	0.127	0.459
Ofloxacin	0 (n=3)	5 (n=3)	0.429	0.513	0.626
Ofloxacin	0 (n=3)	7 (n=3)	2.333	0.127	0.459
Ofloxacin	0 (n=3)	9 (n=2)	1.333	0.248	0.459
Ofloxacin	2 (n=3)	5 (n=3)	1.190	0.275	0.459
Ofloxacin	2 (n=3)	7 (n=3)	0.048	0.827	0.827
Ofloxacin	2 (n=3)	9 (n=2)	0.333	0.564	0.626
Ofloxacin	5 (n=3)	7 (n=3)	1.190	0.275	0.459
Ofloxacin	5 (n=3)	9 (n=2)	1.333	0.248	0.459
Ofloxacin	7 (n=3)	9 (n=2)	0.333	0.564	0.626

Supplementary File 3
Alpha Diversity Output

NPL Evenness--Within-treatment Kruskal-Wallis pairwise comparisons

Treatment	Group 1	Group 2	H	p-value	q-value
TS-30mg/kg	0 (n=3)	2 (n=3)	0.429	0.513	0.805
TS-30mg/kg	0 (n=3)	5 (n=3)	0.429	0.513	0.805
TS-30mg/kg	0 (n=3)	7 (n=2)	1.333	0.248	0.805
TS-30mg/kg	0 (n=3)	9 (n=3)	0.048	0.827	0.827
TS-30mg/kg	2 (n=3)	5 (n=3)	0.429	0.513	0.805
TS-30mg/kg	2 (n=3)	7 (n=2)	3.000	0.083	0.805
TS-30mg/kg	2 (n=3)	9 (n=3)	0.048	0.827	0.827
TS-30mg/kg	5 (n=3)	7 (n=2)	0.333	0.564	0.805
TS-30mg/kg	5 (n=3)	9 (n=3)	0.048	0.827	0.827
TS-30mg/kg	7 (n=2)	9 (n=3)	1.333	0.248	0.805

Treatment	Group 1	Group 2	H	p-value	q-value
TS-15mg/kg	0 (n=3)	2 (n=3)	0.429	0.513	0.827
TS-15mg/kg	0 (n=3)	5 (n=3)	0.048	0.827	0.827
TS-15mg/kg	0 (n=3)	7 (n=3)	0.048	0.827	0.827
TS-15mg/kg	0 (n=3)	9 (n=3)	0.048	0.827	0.827
TS-15mg/kg	2 (n=3)	5 (n=3)	1.190	0.275	0.827
TS-15mg/kg	2 (n=3)	7 (n=3)	0.429	0.513	0.827
TS-15mg/kg	2 (n=3)	9 (n=3)	1.190	0.275	0.827
TS-15mg/kg	5 (n=3)	7 (n=3)	0.048	0.827	0.827
TS-15mg/kg	5 (n=3)	9 (n=3)	0.048	0.827	0.827
TS-15mg/kg	7 (n=3)	9 (n=3)	0.048	0.827	0.827

Treatment	Group 1	Group 2	H	p-value	q-value
Saline	0 (n=3)	2 (n=3)	0.048	0.827	0.919
Saline	0 (n=3)	5 (n=3)	0.048	0.827	0.919
Saline	0 (n=3)	7 (n=3)	0.048	0.827	0.919
Saline	0 (n=3)	9 (n=2)	0.000	1.000	1.000
Saline	2 (n=3)	5 (n=3)	0.429	0.513	0.919
Saline	2 (n=3)	7 (n=3)	0.429	0.513	0.919
Saline	2 (n=3)	9 (n=2)	0.333	0.564	0.919
Saline	5 (n=3)	7 (n=3)	0.429	0.513	0.919
Saline	5 (n=3)	9 (n=2)	0.333	0.564	0.919
Saline	7 (n=3)	9 (n=2)	0.333	0.564	0.919

Supplementary File 3
Alpha Diversity Output

id	Chinchilla_ID	Treatment	Sex	Collection	NPL faith_pd
1BN	Chinch1	AC-PO7d	F	Baseline	8.693244623
2BN	Chinch2	AC-PO7d	F	Baseline	7.270751587
3BN	Chinch3	AC-PO7d	M	Baseline	6.921263699
20NBL	Chinch20	AC-5mg/kg	F	Baseline	4.97383207
23NBL	Chinch23	AC-5mg/kg	F	Baseline	5.410771465
26NBL	Chinch26	AC-5mg/kg	M	Baseline	4.562573196
22NBL	Chinch22	AC-10mg/kg	M	Baseline	5.384661838
25NBL	Chinch25	AC-10mg/kg	F	Baseline	5.450835134
11NBL	Chinch11	anti-rsPilA	F	Baseline	5.191512398
13NBL	Chinch13	anti-rsPilA	F	Baseline	5.559760703
17NBL	Chinch17	anti-rsPilA	F	Baseline	5.322835583
19NBL	Chinch19	anti-tip-chimer	F	Baseline	5.700827972
21NBL	Chinch21	anti-tip-chimer	F	Baseline	5.637074808
27NBL	Chinch27	anti-tip-chimer	M	Baseline	4.966716028
4NBL	Chinch4	Ofloxacin	F	Baseline	4.601597569
5NBL	Chinch5	Ofloxacin	F	Baseline	4.556416864
7NBL	Chinch7	Ofloxacin	M	Baseline	3.333996254
14NBL	Chinch14	TS-15mg/kg	F	Baseline	5.532597957
15NBL	Chinch15	TS-15mg/kg	F	Baseline	5.406846449
18NBL	Chinch18	TS-15mg/kg	M	Baseline	6.14792885
10NBL	Chinch10	TS-30mg/kg	F	Baseline	5.413239766
12NBL	Chinch12	TS-30mg/kg	M	Baseline	5.614279022
16NBL	Chinch16	TS-30mg/kg	F	Baseline	4.901590913
6NBL	Chinch6	Saline	F	Baseline	4.20835026
8NBL	Chinch8	Saline	M	Baseline	5.561387722
9NBL	Chinch9	Saline	F	Baseline	5.612024063
1AN2	Chinch1	AC-PO7d	F	1	3.148473871
2AN2	Chinch2	AC-PO7d	F	1	5.618540336
3AN2	Chinch3	AC-PO7d	M	1	4.774086538
20N1	Chinch20	AC-5mg/kg	F	1	5.831952121
23N1	Chinch23	AC-5mg/kg	F	1	5.209564384
26N1	Chinch26	AC-5mg/kg	M	1	5.245924749
22N1	Chinch22	AC-10mg/kg	M	1	6.043374939
25N1	Chinch25	AC-10mg/kg	F	1	5.019486876
11N1	Chinch11	anti-rsPilA	F	1	5.186017913
13N1	Chinch13	anti-rsPilA	F	1	5.653302407
17N1	Chinch17	anti-rsPilA	F	1	3.797067418
19N1	Chinch19	anti-tip-chimer	F	1	6.484031111
21N1	Chinch21	anti-tip-chimer	F	1	5.105508712
27N1	Chinch27	anti-tip-chimer	M	1	4.306239084
4N1	Chinch4	Ofloxacin	F	1	4.439330425
5N1	Chinch5	Ofloxacin	F	1	4.297267201
7N1	Chinch7	Ofloxacin	M	1	2.917835156
14N1	Chinch14	TS-15mg/kg	F	1	5.143527596
15N1	Chinch15	TS-15mg/kg	F	1	6.082629547
18N1	Chinch18	TS-15mg/kg	M	1	4.722756447
10N1	Chinch10	TS-30mg/kg	F	1	3.121513376

Supplementary File 3
Alpha Diversity Output

id	Chinchilla_ID	Treatment	Sex	Collection	NPL faith_pd
12N1	Chinch12	TS-30mg/kg	M	1	5.54776224
16N1	Chinch16	TS-30mg/kg	F	1	6.192196236
6N1	Chinch6	Saline	F	1	3.940213215
8N1	Chinch8	Saline	M	1	5.828816949
9N1	Chinch9	Saline	F	1	5.890847186
1AN5	Chinch1	AC-PO7d	F	2	3.594289316
2AN5	Chinch2	AC-PO7d	F	2	6.113552719
3AN5	Chinch3	AC-PO7d	M	2	7.215433846
20N2	Chinch20	AC-5mg/kg	F	2	4.021599811
23N2	Chinch23	AC-5mg/kg	F	2	4.509427775
26N2	Chinch26	AC-5mg/kg	M	2	4.67580118
22N2	Chinch22	AC-10mg/kg	M	2	5.031556088
25N2	Chinch25	AC-10mg/kg	F	2	5.393469475
11N2	Chinch11	anti-rsPilA	F	2	5.559738098
17N2	Chinch17	anti-rsPilA	F	2	3.396379416
19N2	Chinch19	anti-tip-chimer	F	2	4.950204185
21N2	Chinch21	anti-tip-chimer	F	2	5.467777372
27N2	Chinch27	anti-tip-chimer	M	2	4.88100255
4N2	Chinch4	Ofloxacin	F	2	5.241148269
5N2	Chinch5	Ofloxacin	F	2	3.80676984
7N2	Chinch7	Ofloxacin	M	2	4.683930133
14N2	Chinch14	TS-15mg/kg	F	2	3.353400248
15N2	Chinch15	TS-15mg/kg	F	2	6.527418247
18N2	Chinch18	TS-15mg/kg	M	2	5.685697378
10N2	Chinch10	TS-30mg/kg	F	2	3.783347115
12N2	Chinch12	TS-30mg/kg	M	2	4.175709064
16N2	Chinch16	TS-30mg/kg	F	2	5.737741907
6N2	Chinch6	Saline	F	2	4.326695407
8N2	Chinch8	Saline	M	2	4.863859729
9N2	Chinch9	Saline	F	2	1.367224278
1AN7	Chinch1	AC-PO7d	F	3	6.419810977
2AN7	Chinch2	AC-PO7d	F	3	5.128097134
20N3	Chinch20	AC-5mg/kg	F	3	3.981125542
23N3	Chinch23	AC-5mg/kg	F	3	4.350022888
26N3	Chinch26	AC-5mg/kg	M	3	3.264328965
25N3	Chinch25	AC-10mg/kg	F	3	4.618527294
11N3	Chinch11	anti-rsPilA	F	3	5.712738389
17N3	Chinch17	anti-rsPilA	F	3	4.43269973
21N3	Chinch21	anti-tip-chimer	F	3	6.016975942
27N3	Chinch27	anti-tip-chimer	M	3	4.849461878
4N3	Chinch4	Ofloxacin	F	3	3.763038972
5N3	Chinch5	Ofloxacin	F	3	4.733509277
7N3	Chinch7	Ofloxacin	M	3	3.509103798
14N3	Chinch14	TS-15mg/kg	F	3	3.635318066
15N3	Chinch15	TS-15mg/kg	F	3	6.37938173
18N3	Chinch18	TS-15mg/kg	M	3	5.735742157
10N3	Chinch10	TS-30mg/kg	F	3	4.342340286
16N3	Chinch16	TS-30mg/kg	F	3	5.723535522

Supplementary File 3
Alpha Diversity Output

id	Chinchilla_ID	Treatment	Sex	Collection	NPL faith_pd
6N3	Chinch6	Saline	F	3	5.636085173
8N3	Chinch8	Saline	M	3	5.092816873
9N3	Chinch9	Saline	F	3	1.727969866
2AN9	Chinch2	AC-PO7d	F	4	7.074008231
3AN9	Chinch3	AC-PO7d	M	4	6.247778628
20N4	Chinch20	AC-5mg/kg	F	4	4.063410074
23N4	Chinch23	AC-5mg/kg	F	4	5.136701997
26N4	Chinch26	AC-5mg/kg	M	4	4.272056506
22N4	Chinch22	AC-10mg/kg	M	4	5.075053126
25N4	Chinch25	AC-10mg/kg	F	4	4.896734922
11N4	Chinch11	anti-rsPilA	F	4	4.848583316
17N4	Chinch17	anti-rsPilA	F	4	3.311325296
19N4	Chinch19	anti-tip-chimer	F	4	5.467475063
21N4	Chinch21	anti-tip-chimer	F	4	6.887398446
27N4	Chinch27	anti-tip-chimer	M	4	5.883049533
5N4	Chinch5	Ofloxacin	F	4	4.01783948
7N4	Chinch7	Ofloxacin	M	4	3.398783566
14N4	Chinch14	TS-15mg/kg	F	4	4.571144252
15N4	Chinch15	TS-15mg/kg	F	4	5.778622032
18N4	Chinch18	TS-15mg/kg	M	4	4.807343562
10N4	Chinch10	TS-30mg/kg	F	4	3.749074905
12N4	Chinch12	TS-30mg/kg	M	4	5.031640209
16N4	Chinch16	TS-30mg/kg	F	4	6.243123575
6N4	Chinch6	Saline	F	4	4.701618146
8N4	Chinch8	Saline	M	4	5.103083909

Supplementary File 3
Alpha Diversity Output

NPL FaithPD-Baseline

Kruskal-Wallis pairwise comparisons

Group 1	Group 2	H	p-value	q-value
AC-PO7d (n=3)	AC-5mg/kg (n=3)	3.857	0.050	0.162
AC-PO7d (n=3)	AC-10mg/kg (n=2)	3.000	0.083	0.231
AC-PO7d (n=3)	Ofloxacin (n=3)	3.857	0.050	0.162
AC-PO7d (n=3)	Saline (n=3)	3.857	0.050	0.162
AC-PO7d (n=3)	TS-30mg/kg (n=3)	3.857	0.050	0.162
AC-PO7d (n=3)	TS-15mg/kg (n=3)	3.857	0.050	0.162
AC-PO7d (n=3)	anti-tip-chimer (n=3)	3.857	0.050	0.162
AC-PO7d (n=3)	anti-rsPilA (n=3)	3.857	0.050	0.162
AC-5mg/kg (n=3)	AC-10mg/kg (n=2)	1.333	0.248	0.413
AC-5mg/kg (n=3)	Ofloxacin (n=3)	2.333	0.127	0.304
AC-5mg/kg (n=3)	Saline (n=3)	0.429	0.513	0.655
AC-5mg/kg (n=3)	TS-30mg/kg (n=3)	1.190	0.275	0.413
AC-5mg/kg (n=3)	TS-15mg/kg (n=3)	2.333	0.127	0.304
AC-5mg/kg (n=3)	anti-tip-chimer (n=3)	1.190	0.275	0.413
AC-5mg/kg (n=3)	anti-rsPilA (n=3)	1.190	0.275	0.413
AC-10mg/kg (n=2)	Ofloxacin (n=3)	3.000	0.083	0.231
AC-10mg/kg (n=2)	Saline (n=3)	0.333	0.564	0.655
AC-10mg/kg (n=2)	TS-30mg/kg (n=3)	0.000	1.000	1.000
AC-10mg/kg (n=2)	TS-15mg/kg (n=3)	1.333	0.248	0.413
AC-10mg/kg (n=2)	anti-tip-chimer (n=3)	0.333	0.564	0.655
AC-10mg/kg (n=2)	anti-rsPilA (n=3)	0.333	0.564	0.655
Ofloxacin (n=3)	Saline (n=3)	1.190	0.275	0.413
Ofloxacin (n=3)	TS-30mg/kg (n=3)	3.857	0.050	0.162
Ofloxacin (n=3)	TS-15mg/kg (n=3)	3.857	0.050	0.162
Ofloxacin (n=3)	anti-tip-chimer (n=3)	3.857	0.050	0.162
Ofloxacin (n=3)	anti-rsPilA (n=3)	3.857	0.050	0.162
Saline (n=3)	TS-30mg/kg (n=3)	0.048	0.827	0.851
Saline (n=3)	TS-15mg/kg (n=3)	0.048	0.827	0.851
Saline (n=3)	anti-tip-chimer (n=3)	1.190	0.275	0.413
Saline (n=3)	anti-rsPilA (n=3)	0.429	0.513	0.655
TS-30mg/kg (n=3)	TS-15mg/kg (n=3)	0.429	0.513	0.655
TS-30mg/kg (n=3)	anti-tip-chimer (n=3)	1.190	0.275	0.413
TS-30mg/kg (n=3)	anti-rsPilA (n=3)	0.048	0.827	0.851
TS-15mg/kg (n=3)	anti-tip-chimer (n=3)	0.048	0.827	0.851
TS-15mg/kg (n=3)	anti-rsPilA (n=3)	1.190	0.275	0.413
anti-tip-chimer (n=3)	anti-rsPilA (n=3)	0.429	0.513	0.655

Kruskal-Wallis (Omnibus Test)

**H**	15.085
**p-value**	0.058

Supplementary File 3
Alpha Diversity Output

NPL FaithPD-2DPT

Kruskal-Wallis pairwise comparisons

Group 1	Group 2	H	p-value	q-value
AC-PO7d (n=3)	AC-5mg/kg (n=3)	1.190	0.275	0.762
AC-PO7d (n=3)	AC-10mg/kg (n=2)	1.333	0.248	0.762
AC-PO7d (n=3)	Ofloxacin (n=3)	1.190	0.275	0.762
AC-PO7d (n=3)	Saline (n=3)	1.190	0.275	0.762
AC-PO7d (n=3)	TS-30mg/kg (n=3)	0.048	0.827	0.931
AC-PO7d (n=3)	TS-15mg/kg (n=3)	0.429	0.513	0.931
AC-PO7d (n=3)	anti-tip-chimer (n=3)	0.429	0.513	0.931
AC-PO7d (n=3)	anti-rsPilA (n=3)	0.429	0.513	0.931
AC-5mg/kg (n=3)	AC-10mg/kg (n=2)	0.000	1.000	1.000
AC-5mg/kg (n=3)	Ofloxacin (n=3)	3.857	0.050	0.762
AC-5mg/kg (n=3)	Saline (n=3)	0.048	0.827	0.931
AC-5mg/kg (n=3)	TS-30mg/kg (n=3)	0.048	0.827	0.931
AC-5mg/kg (n=3)	TS-15mg/kg (n=3)	0.429	0.513	0.931
AC-5mg/kg (n=3)	anti-tip-chimer (n=3)	0.429	0.513	0.931
AC-5mg/kg (n=3)	anti-rsPilA (n=3)	1.190	0.275	0.762
AC-10mg/kg (n=2)	Ofloxacin (n=3)	3.000	0.083	0.762
AC-10mg/kg (n=2)	Saline (n=3)	0.333	0.564	0.931
AC-10mg/kg (n=2)	TS-30mg/kg (n=3)	0.000	1.000	1.000
AC-10mg/kg (n=2)	TS-15mg/kg (n=3)	0.000	1.000	1.000
AC-10mg/kg (n=2)	anti-tip-chimer (n=3)	0.000	1.000	1.000
AC-10mg/kg (n=2)	anti-rsPilA (n=3)	0.333	0.564	0.931
Ofloxacin (n=3)	Saline (n=3)	1.190	0.275	0.762
Ofloxacin (n=3)	TS-30mg/kg (n=3)	1.190	0.275	0.762
Ofloxacin (n=3)	TS-15mg/kg (n=3)	3.857	0.050	0.762
Ofloxacin (n=3)	anti-tip-chimer (n=3)	2.333	0.127	0.762
Ofloxacin (n=3)	anti-rsPilA (n=3)	1.190	0.275	0.762
Saline (n=3)	TS-30mg/kg (n=3)	0.048	0.827	0.931
Saline (n=3)	TS-15mg/kg (n=3)	0.048	0.827	0.931
Saline (n=3)	anti-tip-chimer (n=3)	0.048	0.827	0.931
Saline (n=3)	anti-rsPilA (n=3)	1.190	0.275	0.762
TS-30mg/kg (n=3)	TS-15mg/kg (n=3)	0.048	0.827	0.931
TS-30mg/kg (n=3)	anti-tip-chimer (n=3)	0.048	0.827	0.931
TS-30mg/kg (n=3)	anti-rsPilA (n=3)	0.048	0.827	0.931
TS-15mg/kg (n=3)	anti-tip-chimer (n=3)	0.048	0.827	0.931
TS-15mg/kg (n=3)	anti-rsPilA (n=3)	0.048	0.827	0.931
anti-tip-chimer (n=3)	anti-rsPilA (n=3)	0.048	0.827	0.931

Kruskal-Wallis (Omnibus Test)

**H**	6.060
**p-value**	0.641

Supplementary File 3
Alpha Diversity Output

NPL FaithPD-5DPT

Kruskal-Wallis pairwise comparisons

Group 1	Group 2	H	p-value	q-value
AC-PO7d (n=3)	AC-5mg/kg (n=3)	0.429	0.513	0.752
AC-PO7d (n=3)	AC-10mg/kg (n=2)	0.333	0.564	0.752
AC-PO7d (n=3)	Ofloxacin (n=3)	0.429	0.513	0.752
AC-PO7d (n=3)	Saline (n=3)	1.190	0.275	0.752
AC-PO7d (n=3)	TS-30mg/kg (n=3)	0.429	0.513	0.752
AC-PO7d (n=3)	TS-15mg/kg (n=3)	0.429	0.513	0.752
AC-PO7d (n=3)	anti-tip-chimer (n=3)	0.429	0.513	0.752
AC-PO7d (n=3)	anti-rsPilA (n=2)	1.333	0.248	0.752
AC-5mg/kg (n=3)	AC-10mg/kg (n=2)	3.000	0.083	0.749
AC-5mg/kg (n=3)	Ofloxacin (n=3)	0.429	0.513	0.752
AC-5mg/kg (n=3)	Saline (n=3)	0.048	0.827	0.931
AC-5mg/kg (n=3)	TS-30mg/kg (n=3)	0.048	0.827	0.931
AC-5mg/kg (n=3)	TS-15mg/kg (n=3)	0.429	0.513	0.752
AC-5mg/kg (n=3)	anti-tip-chimer (n=3)	3.857	0.050	0.749
AC-5mg/kg (n=3)	anti-rsPilA (n=2)	0.000	1.000	1.000
AC-10mg/kg (n=2)	Ofloxacin (n=3)	1.333	0.248	0.752
AC-10mg/kg (n=2)	Saline (n=3)	3.000	0.083	0.749
AC-10mg/kg (n=2)	TS-30mg/kg (n=3)	0.333	0.564	0.752
AC-10mg/kg (n=2)	TS-15mg/kg (n=3)	0.333	0.564	0.752
AC-10mg/kg (n=2)	anti-tip-chimer (n=3)	0.333	0.564	0.752
AC-10mg/kg (n=2)	anti-rsPilA (n=2)	0.000	1.000	1.000
Ofloxacin (n=3)	Saline (n=3)	0.429	0.513	0.752
Ofloxacin (n=3)	TS-30mg/kg (n=3)	0.048	0.827	0.931
Ofloxacin (n=3)	TS-15mg/kg (n=3)	0.429	0.513	0.752
Ofloxacin (n=3)	anti-tip-chimer (n=3)	1.190	0.275	0.752
Ofloxacin (n=3)	anti-rsPilA (n=2)	0.000	1.000	1.000
Saline (n=3)	TS-30mg/kg (n=3)	0.048	0.827	0.931
Saline (n=3)	TS-15mg/kg (n=3)	1.190	0.275	0.752
Saline (n=3)	anti-tip-chimer (n=3)	3.857	0.050	0.749
Saline (n=3)	anti-rsPilA (n=2)	0.333	0.564	0.752
TS-30mg/kg (n=3)	TS-15mg/kg (n=3)	0.048	0.827	0.931
TS-30mg/kg (n=3)	anti-tip-chimer (n=3)	0.429	0.513	0.752
TS-30mg/kg (n=3)	anti-rsPilA (n=2)	0.333	0.564	0.752
TS-15mg/kg (n=3)	anti-tip-chimer (n=3)	0.429	0.513	0.752
TS-15mg/kg (n=3)	anti-rsPilA (n=2)	0.333	0.564	0.752
anti-tip-chimer (n=3)	anti-rsPilA (n=2)	0.000	1.000	1.000

Kruskal-Wallis (Omnibus Test)

**H**	5.123
**p-value**	0.744

Supplementary File 3
Alpha Diversity Output

NPL FaithPD-7DPT

Kruskal-Wallis pairwise comparisons

Group 1	Group 2	H	p-value	q-value
AC-PO7d (n=2)	AC-5mg/kg (n=3)	3.000	0.083	0.600
AC-PO7d (n=2)	AC-10mg/kg (n=1)	1.500	0.221	0.661
AC-PO7d (n=2)	Ofloxacin (n=3)	3.000	0.083	0.600
AC-PO7d (n=2)	Saline (n=3)	1.333	0.248	0.661
AC-PO7d (n=2)	TS-30mg/kg (n=2)	0.600	0.439	0.725
AC-PO7d (n=2)	TS-15mg/kg (n=3)	0.333	0.564	0.725
AC-PO7d (n=2)	anti-tip-chimer (n=2)	0.600	0.439	0.725
AC-PO7d (n=2)	anti-rsPilA (n=2)	0.600	0.439	0.725
AC-5mg/kg (n=3)	AC-10mg/kg (n=1)	1.800	0.180	0.661
AC-5mg/kg (n=3)	Ofloxacin (n=3)	0.048	0.827	0.931
AC-5mg/kg (n=3)	Saline (n=3)	0.429	0.513	0.725
AC-5mg/kg (n=3)	TS-30mg/kg (n=2)	1.333	0.248	0.661
AC-5mg/kg (n=3)	TS-15mg/kg (n=3)	1.190	0.275	0.661
AC-5mg/kg (n=3)	anti-tip-chimer (n=2)	3.000	0.083	0.600
AC-5mg/kg (n=3)	anti-rsPilA (n=2)	3.000	0.083	0.600
AC-10mg/kg (n=1)	Ofloxacin (n=3)	0.200	0.655	0.760
AC-10mg/kg (n=1)	Saline (n=3)	0.200	0.655	0.760
AC-10mg/kg (n=1)	TS-30mg/kg (n=2)	0.000	1.000	1.000
AC-10mg/kg (n=1)	TS-15mg/kg (n=3)	0.200	0.655	0.760
AC-10mg/kg (n=1)	anti-tip-chimer (n=2)	1.500	0.221	0.661
AC-10mg/kg (n=1)	anti-rsPilA (n=2)	0.000	1.000	1.000
Ofloxacin (n=3)	Saline (n=3)	0.429	0.513	0.725
Ofloxacin (n=3)	TS-30mg/kg (n=2)	1.333	0.248	0.661
Ofloxacin (n=3)	TS-15mg/kg (n=3)	1.190	0.275	0.661
Ofloxacin (n=3)	anti-tip-chimer (n=2)	3.000	0.083	0.600
Ofloxacin (n=3)	anti-rsPilA (n=2)	1.333	0.248	0.661
Saline (n=3)	TS-30mg/kg (n=2)	0.333	0.564	0.725
Saline (n=3)	TS-15mg/kg (n=3)	1.190	0.275	0.661
Saline (n=3)	anti-tip-chimer (n=2)	0.333	0.564	0.725
Saline (n=3)	anti-rsPilA (n=2)	0.333	0.564	0.725
TS-30mg/kg (n=2)	TS-15mg/kg (n=3)	0.333	0.564	0.725
TS-30mg/kg (n=2)	anti-tip-chimer (n=2)	0.600	0.439	0.725
TS-30mg/kg (n=2)	anti-rsPilA (n=2)	0.000	1.000	1.000
TS-15mg/kg (n=3)	anti-tip-chimer (n=2)	0.000	1.000	1.000
TS-15mg/kg (n=3)	anti-rsPilA (n=2)	0.333	0.564	0.725
anti-tip-chimer (n=2)	anti-rsPilA (n=2)	0.600	0.439	0.725

Kruskal-Wallis (Omnibus Test)

**H**	8.481
**p-value**	0.388

Supplementary File 3
Alpha Diversity Output

NPL FaithPD-9DPT

Kruskal-Wallis pairwise comparisons

Group 1	Group 2	H	p-value	q-value
AC-PO7d (n=2)	AC-5mg/kg (n=3)	3.000	0.083	0.253
AC-PO7d (n=2)	AC-10mg/kg (n=2)	2.400	0.121	0.253
AC-PO7d (n=2)	Ofloxacin (n=2)	2.400	0.121	0.253
AC-PO7d (n=2)	Saline (n=2)	2.400	0.121	0.253
AC-PO7d (n=2)	TS-30mg/kg (n=3)	3.000	0.083	0.253
AC-PO7d (n=2)	TS-15mg/kg (n=3)	3.000	0.083	0.253
AC-PO7d (n=2)	anti-tip-chimer (n=3)	1.333	0.248	0.426
AC-PO7d (n=2)	anti-rsPilA (n=2)	2.400	0.121	0.253
AC-5mg/kg (n=3)	AC-10mg/kg (n=2)	0.333	0.564	0.700
AC-5mg/kg (n=3)	Ofloxacin (n=2)	3.000	0.083	0.253
AC-5mg/kg (n=3)	Saline (n=2)	0.333	0.564	0.700
AC-5mg/kg (n=3)	TS-30mg/kg (n=3)	0.048	0.827	0.961
AC-5mg/kg (n=3)	TS-15mg/kg (n=3)	1.190	0.275	0.431
AC-5mg/kg (n=3)	anti-tip-chimer (n=3)	3.857	0.050	0.253
AC-5mg/kg (n=3)	anti-rsPilA (n=2)	0.333	0.564	0.700
AC-10mg/kg (n=2)	Ofloxacin (n=2)	2.400	0.121	0.253
AC-10mg/kg (n=2)	Saline (n=2)	0.000	1.000	1.000
AC-10mg/kg (n=2)	TS-30mg/kg (n=3)	0.000	1.000	1.000
AC-10mg/kg (n=2)	TS-15mg/kg (n=3)	0.333	0.564	0.700
AC-10mg/kg (n=2)	anti-tip-chimer (n=3)	3.000	0.083	0.253
AC-10mg/kg (n=2)	anti-rsPilA (n=2)	2.400	0.121	0.253
Ofloxacin (n=2)	Saline (n=2)	2.400	0.121	0.253
Ofloxacin (n=2)	TS-30mg/kg (n=3)	1.333	0.248	0.426
Ofloxacin (n=2)	TS-15mg/kg (n=3)	3.000	0.083	0.253
Ofloxacin (n=2)	anti-tip-chimer (n=3)	3.000	0.083	0.253
Ofloxacin (n=2)	anti-rsPilA (n=2)	0.000	1.000	1.000
Saline (n=2)	TS-30mg/kg (n=3)	0.000	1.000	1.000
Saline (n=2)	TS-15mg/kg (n=3)	0.000	1.000	1.000
Saline (n=2)	anti-tip-chimer (n=3)	3.000	0.083	0.253
Saline (n=2)	anti-rsPilA (n=2)	0.600	0.439	0.658
TS-30mg/kg (n=3)	TS-15mg/kg (n=3)	0.048	0.827	0.961
TS-30mg/kg (n=3)	anti-tip-chimer (n=3)	1.190	0.275	0.431
TS-30mg/kg (n=3)	anti-rsPilA (n=2)	1.333	0.248	0.426
TS-15mg/kg (n=3)	anti-tip-chimer (n=3)	2.333	0.127	0.253
TS-15mg/kg (n=3)	anti-rsPilA (n=2)	0.333	0.564	0.700
anti-tip-chimer (n=3)	anti-rsPilA (n=2)	3.000	0.083	0.253

Kruskal-Wallis (Omnibus Test)

**H**	13.352
p-value	0.100

Supplementary File 3
Alpha Diversity Output

NPL FaithPD--Within-treatment Kruskal-Wallis pairwise comparisons

Treatment	Group 1	Group 2	H	p-value	q-value
AC-PO7d	0 (n=3)	2 (n=3)	3.857	0.050	0.278
AC-PO7d	0 (n=3)	5 (n=3)	2.333	0.127	0.317
AC-PO7d	0 (n=3)	7 (n=2)	3.000	0.083	0.278
AC-PO7d	0 (n=3)	9 (n=2)	1.333	0.248	0.393
AC-PO7d	2 (n=3)	5 (n=3)	1.190	0.275	0.393
AC-PO7d	2 (n=3)	7 (n=2)	1.333	0.248	0.393
AC-PO7d	2 (n=3)	9 (n=2)	3.000	0.083	0.278
AC-PO7d	5 (n=3)	7 (n=2)	0.000	1.000	1.000
AC-PO7d	5 (n=3)	9 (n=2)	0.333	0.564	0.626
AC-PO7d	7 (n=2)	9 (n=2)	0.600	0.439	0.548

Treatment	Group 1	Group 2	H	p-value	q-value
AC-10mg/kg	0 (n=2)	2 (n=2)	0.000	1.000	1.000
AC-10mg/kg	0 (n=2)	5 (n=2)	0.600	0.439	0.548
AC-10mg/kg	0 (n=2)	7 (n=1)	1.500	0.221	0.441
AC-10mg/kg	0 (n=2)	9 (n=2)	2.400	0.121	0.441
AC-10mg/kg	2 (n=2)	5 (n=2)	0.000	1.000	1.000
AC-10mg/kg	2 (n=2)	7 (n=1)	1.500	0.221	0.441
AC-10mg/kg	2 (n=2)	9 (n=2)	0.600	0.439	0.548
AC-10mg/kg	5 (n=2)	7 (n=1)	1.500	0.221	0.441
AC-10mg/kg	5 (n=2)	9 (n=2)	0.600	0.439	0.548
AC-10mg/kg	7 (n=1)	9 (n=2)	1.500	0.221	0.441

Treatment	Group 1	Group 2	H	p-value	q-value
AC-5mg/kg	0 (n=3)	2 (n=3)	1.190	0.275	0.306
AC-5mg/kg	0 (n=3)	5 (n=3)	2.333	0.127	0.211
AC-5mg/kg	0 (n=3)	7 (n=3)	3.857	0.050	0.124
AC-5mg/kg	0 (n=3)	9 (n=3)	1.190	0.275	0.306
AC-5mg/kg	2 (n=3)	5 (n=3)	3.857	0.050	0.124
AC-5mg/kg	2 (n=3)	7 (n=3)	3.857	0.050	0.124
AC-5mg/kg	2 (n=3)	9 (n=3)	3.857	0.050	0.124
AC-5mg/kg	5 (n=3)	7 (n=3)	2.333	0.127	0.211
AC-5mg/kg	5 (n=3)	9 (n=3)	0.048	0.827	0.827
AC-5mg/kg	7 (n=3)	9 (n=3)	1.190	0.275	0.306

Supplementary File 3
Alpha Diversity Output

NPL FaithPD--Within-treatment Kruskal-Wallis pairwise comparisons

Treatment	Group 1	Group 2	H	p-value	q-value
anti-rsPilA	0 (n=3)	2 (n=3)	0.429	0.513	0.626
anti-rsPilA	0 (n=3)	5 (n=2)	0.333	0.564	0.626
anti-rsPilA	0 (n=3)	7 (n=2)	0.000	1.000	1.000
anti-rsPilA	0 (n=3)	9 (n=2)	3.000	0.083	0.626
anti-rsPilA	2 (n=3)	5 (n=2)	0.333	0.564	0.626
anti-rsPilA	2 (n=3)	7 (n=2)	0.333	0.564	0.626
anti-rsPilA	2 (n=3)	9 (n=2)	1.333	0.248	0.626
anti-rsPilA	5 (n=2)	7 (n=2)	0.600	0.439	0.626
anti-rsPilA	5 (n=2)	9 (n=2)	0.600	0.439	0.626
anti-rsPilA	7 (n=2)	9 (n=2)	0.600	0.439	0.626

Treatment	Group 1	Group 2	H	p-value	q-value
anti-tip-chimer	0 (n=3)	2 (n=3)	0.048	0.827	1.000
anti-tip-chimer	0 (n=3)	5 (n=3)	2.333	0.127	0.633
anti-tip-chimer	0 (n=3)	7 (n=2)	0.000	1.000	1.000
anti-tip-chimer	0 (n=3)	9 (n=3)	1.190	0.275	0.688
anti-tip-chimer	2 (n=3)	5 (n=3)	0.048	0.827	1.000
anti-tip-chimer	2 (n=3)	7 (n=2)	0.000	1.000	1.000
anti-tip-chimer	2 (n=3)	9 (n=3)	1.190	0.275	0.688
anti-tip-chimer	5 (n=3)	7 (n=2)	0.000	1.000	1.000
anti-tip-chimer	5 (n=3)	9 (n=3)	2.333	0.127	0.633
anti-tip-chimer	7 (n=2)	9 (n=3)	0.333	0.564	1.000

Treatment	Group 1	Group 2	H	p-value	q-value
Ofloxacin	0 (n=3)	2 (n=3)	1.190	0.275	0.550
Ofloxacin	0 (n=3)	5 (n=3)	1.190	0.275	0.550
Ofloxacin	0 (n=3)	7 (n=3)	0.048	0.827	0.827
Ofloxacin	0 (n=3)	9 (n=2)	0.333	0.564	0.705
Ofloxacin	2 (n=3)	5 (n=3)	1.190	0.275	0.550
Ofloxacin	2 (n=3)	7 (n=3)	0.048	0.827	0.827
Ofloxacin	2 (n=3)	9 (n=2)	0.333	0.564	0.705
Ofloxacin	5 (n=3)	7 (n=3)	1.190	0.275	0.550
Ofloxacin	5 (n=3)	9 (n=2)	1.333	0.248	0.550
Ofloxacin	7 (n=3)	9 (n=2)	0.333	0.564	0.705

Supplementary File 3
Alpha Diversity Output

NPL FaithPD--Within-treatment Kruskal-Wallis pairwise comparisons

Treatment	Group 1	Group 2	H	p-value	q-value
TS-30mg/kg	0 (n=3)	2 (n=3)	0.048	0.827	1.000
TS-30mg/kg	0 (n=3)	5 (n=3)	0.429	0.513	1.000
TS-30mg/kg	0 (n=3)	7 (n=2)	0.000	1.000	1.000
TS-30mg/kg	0 (n=3)	9 (n=3)	0.048	0.827	1.000
TS-30mg/kg	2 (n=3)	5 (n=3)	0.048	0.827	1.000
TS-30mg/kg	2 (n=3)	7 (n=2)	0.000	1.000	1.000
TS-30mg/kg	2 (n=3)	9 (n=3)	0.048	0.827	1.000
TS-30mg/kg	5 (n=3)	7 (n=2)	0.333	0.564	1.000
TS-30mg/kg	5 (n=3)	9 (n=3)	0.048	0.827	1.000
TS-30mg/kg	7 (n=2)	9 (n=3)	0.000	1.000	1.000

Treatment	Group 1	Group 2	H	p-value	q-value
TS-15mg/kg	0 (n=3)	2 (n=3)	1.190	0.275	0.827
TS-15mg/kg	0 (n=3)	5 (n=3)	0.048	0.827	0.827
TS-15mg/kg	0 (n=3)	7 (n=3)	0.048	0.827	0.827
TS-15mg/kg	0 (n=3)	9 (n=3)	1.190	0.275	0.827
TS-15mg/kg	2 (n=3)	5 (n=3)	0.048	0.827	0.827
TS-15mg/kg	2 (n=3)	7 (n=3)	0.048	0.827	0.827
TS-15mg/kg	2 (n=3)	9 (n=3)	0.429	0.513	0.827
TS-15mg/kg	5 (n=3)	7 (n=3)	0.048	0.827	0.827
TS-15mg/kg	5 (n=3)	9 (n=3)	0.048	0.827	0.827
TS-15mg/kg	7 (n=3)	9 (n=3)	0.048	0.827	0.827

Treatment	Group 1	Group 2	H	p-value	q-value
Saline	0 (n=3)	2 (n=3)	0.429	0.513	0.705
Saline	0 (n=3)	5 (n=3)	1.190	0.275	0.550
Saline	0 (n=3)	7 (n=3)	0.048	0.827	0.919
Saline	0 (n=3)	9 (n=2)	0.333	0.564	0.705
Saline	2 (n=3)	5 (n=3)	1.190	0.275	0.550
Saline	2 (n=3)	7 (n=3)	1.190	0.275	0.550
Saline	2 (n=3)	9 (n=2)	0.333	0.564	0.705
Saline	5 (n=3)	7 (n=3)	1.190	0.275	0.550
Saline	5 (n=3)	9 (n=2)	1.333	0.248	0.550
Saline	7 (n=3)	9 (n=2)	0.000	1.000	1.000

Supplementary File 3
Alpha Diversity Output

id	Chinchilla_ID	Treatment	Sex	Collection	NPL observed_features
1BN	Chinch1	AC-PO7d	F	Baseline	105
2BN	Chinch2	AC-PO7d	F	Baseline	95
3BN	Chinch3	AC-PO7d	M	Baseline	64
20NBL	Chinch20	AC-5mg/kg	F	Baseline	57
23NBL	Chinch23	AC-5mg/kg	F	Baseline	59
26NBL	Chinch26	AC-5mg/kg	M	Baseline	57
22NBL	Chinch22	AC-10mg/kg	M	Baseline	62
25NBL	Chinch25	AC-10mg/kg	F	Baseline	72
11NBL	Chinch11	anti-rsPilA	F	Baseline	79
13NBL	Chinch13	anti-rsPilA	F	Baseline	67
17NBL	Chinch17	anti-rsPilA	F	Baseline	73
19NBL	Chinch19	anti-tip-chimer	F	Baseline	65
21NBL	Chinch21	anti-tip-chimer	F	Baseline	57
27NBL	Chinch27	anti-tip-chimer	M	Baseline	79
4NBL	Chinch4	Ofloxacin	F	Baseline	49
5NBL	Chinch5	Ofloxacin	F	Baseline	53
7NBL	Chinch7	Ofloxacin	M	Baseline	51
14NBL	Chinch14	TS-15mg/kg	F	Baseline	64
15NBL	Chinch15	TS-15mg/kg	F	Baseline	53
18NBL	Chinch18	TS-15mg/kg	M	Baseline	62
10NBL	Chinch10	TS-30mg/kg	F	Baseline	63
12NBL	Chinch12	TS-30mg/kg	M	Baseline	67
16NBL	Chinch16	TS-30mg/kg	F	Baseline	62
6NBL	Chinch6	Saline	F	Baseline	53
8NBL	Chinch8	Saline	M	Baseline	50
9NBL	Chinch9	Saline	F	Baseline	59
1AN2	Chinch1	AC-PO7d	F	1	39
2AN2	Chinch2	AC-PO7d	F	1	59
3AN2	Chinch3	AC-PO7d	M	1	54
20N1	Chinch20	AC-5mg/kg	F	1	65
23N1	Chinch23	AC-5mg/kg	F	1	66
26N1	Chinch26	AC-5mg/kg	M	1	66
22N1	Chinch22	AC-10mg/kg	M	1	62
25N1	Chinch25	AC-10mg/kg	F	1	56
11N1	Chinch11	anti-rsPilA	F	1	64
13N1	Chinch13	anti-rsPilA	F	1	72
17N1	Chinch17	anti-rsPilA	F	1	59
19N1	Chinch19	anti-tip-chimer	F	1	75
21N1	Chinch21	anti-tip-chimer	F	1	62
27N1	Chinch27	anti-tip-chimer	M	1	64
4N1	Chinch4	Ofloxacin	F	1	45
5N1	Chinch5	Ofloxacin	F	1	55
7N1	Chinch7	Ofloxacin	M	1	42
14N1	Chinch14	TS-15mg/kg	F	1	74
15N1	Chinch15	TS-15mg/kg	F	1	82
18N1	Chinch18	TS-15mg/kg	M	1	50
10N1	Chinch10	TS-30mg/kg	F	1	39

Supplementary File 3
Alpha Diversity Output

id	Chinchilla_ID	Treatment	Sex	Collection	NPL observed_features
12N1	Chinch12	TS-30mg/kg	M	1	66
16N1	Chinch16	TS-30mg/kg	F	1	68
6N1	Chinch6	Saline	F	1	49
8N1	Chinch8	Saline	M	1	59
9N1	Chinch9	Saline	F	1	64
1AN5	Chinch1	AC-PO7d	F	2	46
2AN5	Chinch2	AC-PO7d	F	2	68
3AN5	Chinch3	AC-PO7d	M	2	82
20N2	Chinch20	AC-5mg/kg	F	2	46
23N2	Chinch23	AC-5mg/kg	F	2	62
26N2	Chinch26	AC-5mg/kg	M	2	59
22N2	Chinch22	AC-10mg/kg	M	2	67
25N2	Chinch25	AC-10mg/kg	F	2	61
11N2	Chinch11	anti-rsPilA	F	2	79
17N2	Chinch17	anti-rsPilA	F	2	52
19N2	Chinch19	anti-tip-chimer	F	2	62
21N2	Chinch21	anti-tip-chimer	F	2	55
27N2	Chinch27	anti-tip-chimer	M	2	73
4N2	Chinch4	Ofloxacin	F	2	49
5N2	Chinch5	Ofloxacin	F	2	53
7N2	Chinch7	Ofloxacin	M	2	58
14N2	Chinch14	TS-15mg/kg	F	2	53
15N2	Chinch15	TS-15mg/kg	F	2	99
18N2	Chinch18	TS-15mg/kg	M	2	65
10N2	Chinch10	TS-30mg/kg	F	2	43
12N2	Chinch12	TS-30mg/kg	M	2	57
16N2	Chinch16	TS-30mg/kg	F	2	58
6N2	Chinch6	Saline	F	2	58
8N2	Chinch8	Saline	M	2	58
9N2	Chinch9	Saline	F	2	13
1AN7	Chinch1	AC-PO7d	F	3	83
2AN7	Chinch2	AC-PO7d	F	3	52
20N3	Chinch20	AC-5mg/kg	F	3	54
23N3	Chinch23	AC-5mg/kg	F	3	56
26N3	Chinch26	AC-5mg/kg	M	3	53
25N3	Chinch25	AC-10mg/kg	F	3	52
11N3	Chinch11	anti-rsPilA	F	3	83
17N3	Chinch17	anti-rsPilA	F	3	62
21N3	Chinch21	anti-tip-chimer	F	3	66
27N3	Chinch27	anti-tip-chimer	M	3	62
4N3	Chinch4	Ofloxacin	F	3	48
5N3	Chinch5	Ofloxacin	F	3	50
7N3	Chinch7	Ofloxacin	M	3	51
14N3	Chinch14	TS-15mg/kg	F	3	53
15N3	Chinch15	TS-15mg/kg	F	3	70
18N3	Chinch18	TS-15mg/kg	M	3	64
10N3	Chinch10	TS-30mg/kg	F	3	43
16N3	Chinch16	TS-30mg/kg	F	3	67

Supplementary File 3
Alpha Diversity Output

id	Chinchilla_ID	Treatment	Sex	Collection	NPL observed_features
6N3	Chinch6	Saline	F	3	74
8N3	Chinch8	Saline	M	3	66
9N3	Chinch9	Saline	F	3	21
2AN9	Chinch2	AC-PO7d	F	4	83
3AN9	Chinch3	AC-PO7d	M	4	82
20N4	Chinch20	AC-5mg/kg	F	4	46
23N4	Chinch23	AC-5mg/kg	F	4	77
26N4	Chinch26	AC-5mg/kg	M	4	62
22N4	Chinch22	AC-10mg/kg	M	4	63
25N4	Chinch25	AC-10mg/kg	F	4	55
11N4	Chinch11	anti-rsPilA	F	4	55
17N4	Chinch17	anti-rsPilA	F	4	53
19N4	Chinch19	anti-tip-chimer	F	4	77
21N4	Chinch21	anti-tip-chimer	F	4	93
27N4	Chinch27	anti-tip-chimer	M	4	78
5N4	Chinch5	Ofloxacin	F	4	45
7N4	Chinch7	Ofloxacin	M	4	47
14N4	Chinch14	TS-15mg/kg	F	4	63
15N4	Chinch15	TS-15mg/kg	F	4	74
18N4	Chinch18	TS-15mg/kg	M	4	57
10N4	Chinch10	TS-30mg/kg	F	4	37
12N4	Chinch12	TS-30mg/kg	M	4	68
16N4	Chinch16	TS-30mg/kg	F	4	60
6N4	Chinch6	Saline	F	4	65
8N4	Chinch8	Saline	M	4	64

Supplementary File 3
Alpha Diversity Output

NPL Richness-Baseline

Kruskal-Wallis pairwise comparisons

Group 1	Group 2	H	p-value	q-value
AC-PO7d (n=3)	AC-5mg/kg (n=3)	3.971	0.046	0.149
AC-PO7d (n=3)	AC-10mg/kg (n=2)	1.333	0.248	0.372
AC-PO7d (n=3)	Ofloxacin (n=3)	3.857	0.050	0.149
AC-PO7d (n=3)	Saline (n=3)	3.857	0.050	0.149
AC-PO7d (n=3)	TS-30mg/kg (n=3)	2.333	0.127	0.228
AC-PO7d (n=3)	TS-15mg/kg (n=3)	3.137	0.077	0.167
AC-PO7d (n=3)	anti-tip-chimer (n=3)	1.190	0.275	0.381
AC-PO7d (n=3)	anti-rsPilA (n=3)	0.429	0.513	0.559
AC-5mg/kg (n=3)	AC-10mg/kg (n=2)	3.158	0.076	0.167
AC-5mg/kg (n=3)	Ofloxacin (n=3)	3.971	0.046	0.149
AC-5mg/kg (n=3)	Saline (n=3)	0.808	0.369	0.436
AC-5mg/kg (n=3)	TS-30mg/kg (n=3)	3.971	0.046	0.149
AC-5mg/kg (n=3)	TS-15mg/kg (n=3)	0.441	0.507	0.559
AC-5mg/kg (n=3)	anti-tip-chimer (n=3)	1.344	0.246	0.372
AC-5mg/kg (n=3)	anti-rsPilA (n=3)	3.971	0.046	0.149
AC-10mg/kg (n=2)	Ofloxacin (n=3)	3.000	0.083	0.167
AC-10mg/kg (n=2)	Saline (n=3)	3.000	0.083	0.167
AC-10mg/kg (n=2)	TS-30mg/kg (n=3)	0.088	0.767	0.812
AC-10mg/kg (n=2)	TS-15mg/kg (n=3)	0.789	0.374	0.436
AC-10mg/kg (n=2)	anti-tip-chimer (n=3)	0.000	1.000	1.000
AC-10mg/kg (n=2)	anti-rsPilA (n=3)	1.333	0.248	0.372
Ofloxacin (n=3)	Saline (n=3)	0.784	0.376	0.436
Ofloxacin (n=3)	TS-30mg/kg (n=3)	3.857	0.050	0.149
Ofloxacin (n=3)	TS-15mg/kg (n=3)	3.137	0.077	0.167
Ofloxacin (n=3)	anti-tip-chimer (n=3)	3.857	0.050	0.149
Ofloxacin (n=3)	anti-rsPilA (n=3)	3.857	0.050	0.149
Saline (n=3)	TS-30mg/kg (n=3)	3.857	0.050	0.149
Saline (n=3)	TS-15mg/kg (n=3)	1.765	0.184	0.315
Saline (n=3)	anti-tip-chimer (n=3)	2.333	0.127	0.228
Saline (n=3)	anti-rsPilA (n=3)	3.857	0.050	0.149
TS-30mg/kg (n=3)	TS-15mg/kg (n=3)	0.784	0.376	0.436
TS-30mg/kg (n=3)	anti-tip-chimer (n=3)	0.048	0.827	0.851
TS-30mg/kg (n=3)	anti-rsPilA (n=3)	3.137	0.077	0.167
TS-15mg/kg (n=3)	anti-tip-chimer (n=3)	1.190	0.275	0.381
TS-15mg/kg (n=3)	anti-rsPilA (n=3)	3.857	0.050	0.149
anti-tip-chimer (n=3)	anti-rsPilA (n=3)	0.784	0.376	0.436

Kruskal-Wallis (Omnibus Test)

**H**	18.827
**p-value**	0.016

Supplementary File 3
Alpha Diversity Output

NPL Richness-2DPT

Kruskal-Wallis pairwise comparisons

Group 1	Group 2	H	p-value	q-value
AC-PO7d (n=3)	AC-5mg/kg (n=3)	3.971	0.046	0.297
AC-PO7d (n=3)	AC-10mg/kg (n=2)	1.333	0.248	0.521
AC-PO7d (n=3)	Ofloxacin (n=3)	0.048	0.827	0.851
AC-PO7d (n=3)	Saline (n=3)	0.784	0.376	0.644
AC-PO7d (n=3)	TS-30mg/kg (n=3)	0.784	0.376	0.644
AC-PO7d (n=3)	TS-15mg/kg (n=3)	1.190	0.275	0.521
AC-PO7d (n=3)	anti-tip-chimer (n=3)	3.857	0.050	0.297
AC-PO7d (n=3)	anti-rsPilA (n=3)	3.137	0.077	0.333
AC-5mg/kg (n=3)	AC-10mg/kg (n=2)	3.158	0.076	0.333
AC-5mg/kg (n=3)	Ofloxacin (n=3)	3.971	0.046	0.297
AC-5mg/kg (n=3)	Saline (n=3)	3.971	0.046	0.297
AC-5mg/kg (n=3)	TS-30mg/kg (n=3)	0.054	0.817	0.851
AC-5mg/kg (n=3)	TS-15mg/kg (n=3)	0.441	0.507	0.684
AC-5mg/kg (n=3)	anti-tip-chimer (n=3)	0.441	0.507	0.684
AC-5mg/kg (n=3)	anti-rsPilA (n=3)	0.441	0.507	0.684
AC-10mg/kg (n=2)	Ofloxacin (n=3)	3.000	0.083	0.333
AC-10mg/kg (n=2)	Saline (n=3)	0.000	1.000	1.000
AC-10mg/kg (n=2)	TS-30mg/kg (n=3)	0.333	0.564	0.700
AC-10mg/kg (n=2)	TS-15mg/kg (n=3)	0.333	0.564	0.700
AC-10mg/kg (n=2)	anti-tip-chimer (n=3)	2.193	0.139	0.416
AC-10mg/kg (n=2)	anti-rsPilA (n=3)	1.333	0.248	0.521
Ofloxacin (n=3)	Saline (n=3)	2.333	0.127	0.414
Ofloxacin (n=3)	TS-30mg/kg (n=3)	0.429	0.513	0.684
Ofloxacin (n=3)	TS-15mg/kg (n=3)	2.333	0.127	0.414
Ofloxacin (n=3)	anti-tip-chimer (n=3)	3.857	0.050	0.297
Ofloxacin (n=3)	anti-rsPilA (n=3)	3.857	0.050	0.297
Saline (n=3)	TS-30mg/kg (n=3)	0.429	0.513	0.684
Saline (n=3)	TS-15mg/kg (n=3)	1.190	0.275	0.521
Saline (n=3)	anti-tip-chimer (n=3)	1.765	0.184	0.510
Saline (n=3)	anti-rsPilA (n=3)	1.263	0.261	0.521
TS-30mg/kg (n=3)	TS-15mg/kg (n=3)	1.190	0.275	0.521
TS-30mg/kg (n=3)	anti-tip-chimer (n=3)	0.048	0.827	0.851
TS-30mg/kg (n=3)	anti-rsPilA (n=3)	0.048	0.827	0.851
TS-15mg/kg (n=3)	anti-tip-chimer (n=3)	0.048	0.827	0.851
TS-15mg/kg (n=3)	anti-rsPilA (n=3)	0.429	0.513	0.684
anti-tip-chimer (n=3)	anti-rsPilA (n=3)	0.196	0.658	0.789

Kruskal-Wallis (Omnibus Test)

**H**	11.655
**p-value**	0.167

Supplementary File 3
Alpha Diversity Output

NPL Richness-5DPT

Kruskal-Wallis pairwise comparisons

Group 1	Group 2	H	p-value	q-value
AC-PO7d (n=3)	AC-5mg/kg (n=3)	0.784	0.376	0.796
AC-PO7d (n=3)	AC-10mg/kg (n=2)	0.333	0.564	0.812
AC-PO7d (n=3)	Ofloxacin (n=3)	0.429	0.513	0.812
AC-PO7d (n=3)	Saline (n=3)	1.225	0.268	0.661
AC-PO7d (n=3)	TS-30mg/kg (n=3)	1.190	0.275	0.661
AC-PO7d (n=3)	TS-15mg/kg (n=3)	0.048	0.827	0.993
AC-PO7d (n=3)	anti-tip-chimer (n=3)	0.048	0.827	0.993
AC-PO7d (n=3)	anti-rsPilA (n=2)	0.000	1.000	1.000
AC-5mg/kg (n=3)	AC-10mg/kg (n=2)	1.333	0.248	0.661
AC-5mg/kg (n=3)	Ofloxacin (n=3)	0.429	0.513	0.812
AC-5mg/kg (n=3)	Saline (n=3)	1.225	0.268	0.661
AC-5mg/kg (n=3)	TS-30mg/kg (n=3)	1.190	0.275	0.661
AC-5mg/kg (n=3)	TS-15mg/kg (n=3)	1.190	0.275	0.661
AC-5mg/kg (n=3)	anti-tip-chimer (n=3)	0.784	0.376	0.796
AC-5mg/kg (n=3)	anti-rsPilA (n=2)	0.333	0.564	0.812
AC-10mg/kg (n=2)	Ofloxacin (n=3)	3.000	0.083	0.661
AC-10mg/kg (n=2)	Saline (n=3)	3.158	0.076	0.661
AC-10mg/kg (n=2)	TS-30mg/kg (n=3)	3.000	0.083	0.661
AC-10mg/kg (n=2)	TS-15mg/kg (n=3)	0.000	1.000	1.000
AC-10mg/kg (n=2)	anti-tip-chimer (n=3)	0.000	1.000	1.000
AC-10mg/kg (n=2)	anti-rsPilA (n=2)	0.000	1.000	1.000
Ofloxacin (n=3)	Saline (n=3)	0.054	0.817	0.993
Ofloxacin (n=3)	TS-30mg/kg (n=3)	0.000	1.000	1.000
Ofloxacin (n=3)	TS-15mg/kg (n=3)	1.765	0.184	0.661
Ofloxacin (n=3)	anti-tip-chimer (n=3)	2.333	0.127	0.661
Ofloxacin (n=3)	anti-rsPilA (n=2)	0.333	0.564	0.812
Saline (n=3)	TS-30mg/kg (n=3)	0.054	0.817	0.993
Saline (n=3)	TS-15mg/kg (n=3)	1.225	0.268	0.661
Saline (n=3)	anti-tip-chimer (n=3)	1.225	0.268	0.661
Saline (n=3)	anti-rsPilA (n=2)	0.351	0.554	0.812
TS-30mg/kg (n=3)	TS-15mg/kg (n=3)	1.190	0.275	0.661
TS-30mg/kg (n=3)	anti-tip-chimer (n=3)	1.190	0.275	0.661
TS-30mg/kg (n=3)	anti-rsPilA (n=2)	0.333	0.564	0.812
TS-15mg/kg (n=3)	anti-tip-chimer (n=3)	0.048	0.827	0.993
TS-15mg/kg (n=3)	anti-rsPilA (n=2)	0.333	0.564	0.812
anti-tip-chimer (n=3)	anti-rsPilA (n=2)	0.000	1.000	1.000

Kruskal-Wallis (Omnibus Test)

**H**	6.707
**p-value**	0.569

Supplementary File 3
Alpha Diversity Output

NPL Richness-7DPT

Kruskal-Wallis pairwise comparisons

Group 1	Group 2	H	p-value	q-value
AC-PO7d (n=2)	AC-5mg/kg (n=3)	0.000	1.000	1.000
AC-PO7d (n=2)	AC-10mg/kg (n=1)	0.500	0.480	0.922
AC-PO7d (n=2)	Ofloxacin (n=3)	3.000	0.083	0.428
AC-PO7d (n=2)	Saline (n=3)	0.333	0.564	0.922
AC-PO7d (n=2)	TS-30mg/kg (n=2)	0.600	0.439	0.922
AC-PO7d (n=2)	TS-15mg/kg (n=3)	0.000	1.000	1.000
AC-PO7d (n=2)	anti-tip-chimer (n=2)	0.000	1.000	1.000
AC-PO7d (n=2)	anti-rsPilA (n=2)	0.167	0.683	0.984
AC-5mg/kg (n=3)	AC-10mg/kg (n=1)	1.800	0.180	0.647
AC-5mg/kg (n=3)	Ofloxacin (n=3)	3.857	0.050	0.428
AC-5mg/kg (n=3)	Saline (n=3)	0.429	0.513	0.922
AC-5mg/kg (n=3)	TS-30mg/kg (n=2)	0.000	1.000	1.000
AC-5mg/kg (n=3)	TS-15mg/kg (n=3)	0.784	0.376	0.922
AC-5mg/kg (n=3)	anti-tip-chimer (n=2)	3.000	0.083	0.428
AC-5mg/kg (n=3)	anti-rsPilA (n=2)	3.000	0.083	0.428
AC-10mg/kg (n=1)	Ofloxacin (n=3)	1.800	0.180	0.647
AC-10mg/kg (n=1)	Saline (n=3)	0.200	0.655	0.984
AC-10mg/kg (n=1)	TS-30mg/kg (n=2)	0.000	1.000	1.000
AC-10mg/kg (n=1)	TS-15mg/kg (n=3)	1.800	0.180	0.647
AC-10mg/kg (n=1)	anti-tip-chimer (n=2)	1.500	0.221	0.662
AC-10mg/kg (n=1)	anti-rsPilA (n=2)	1.500	0.221	0.662
Ofloxacin (n=3)	Saline (n=3)	0.429	0.513	0.922
Ofloxacin (n=3)	TS-30mg/kg (n=2)	0.000	1.000	1.000
Ofloxacin (n=3)	TS-15mg/kg (n=3)	3.857	0.050	0.428
Ofloxacin (n=3)	anti-tip-chimer (n=2)	3.000	0.083	0.428
Ofloxacin (n=3)	anti-rsPilA (n=2)	3.000	0.083	0.428
Saline (n=3)	TS-30mg/kg (n=2)	0.000	1.000	1.000
Saline (n=3)	TS-15mg/kg (n=3)	0.048	0.827	1.000
Saline (n=3)	anti-tip-chimer (n=2)	0.088	0.767	1.000
Saline (n=3)	anti-rsPilA (n=2)	0.333	0.564	0.922
TS-30mg/kg (n=2)	TS-15mg/kg (n=3)	0.333	0.564	0.922
TS-30mg/kg (n=2)	anti-tip-chimer (n=2)	0.000	1.000	1.000
TS-30mg/kg (n=2)	anti-rsPilA (n=2)	0.600	0.439	0.922
TS-15mg/kg (n=3)	anti-tip-chimer (n=2)	0.000	1.000	1.000
TS-15mg/kg (n=3)	anti-rsPilA (n=2)	0.333	0.564	0.922
anti-tip-chimer (n=2)	anti-rsPilA (n=2)	0.167	0.683	0.984

Kruskal-Wallis (Omnibus Test)

**H**	7.496
**p-value**	0.484

Supplementary File 3
Alpha Diversity Output

NPL Richness-9DPT

Kruskal-Wallis pairwise comparisons

Group 1	Group 2	H	p-value	q-value
AC-PO7d (n=2)	AC-5mg/kg (n=3)	3.000	0.083	0.208
AC-PO7d (n=2)	AC-10mg/kg (n=2)	2.400	0.121	0.208
AC-PO7d (n=2)	Ofloxacin (n=2)	2.400	0.121	0.208
AC-PO7d (n=2)	Saline (n=2)	2.400	0.121	0.208
AC-PO7d (n=2)	TS-30mg/kg (n=3)	3.000	0.083	0.208
AC-PO7d (n=2)	TS-15mg/kg (n=3)	3.000	0.083	0.208
AC-PO7d (n=2)	anti-tip-chimer (n=3)	0.333	0.564	0.615
AC-PO7d (n=2)	anti-rsPilA (n=2)	2.400	0.121	0.208
AC-5mg/kg (n=3)	AC-10mg/kg (n=2)	0.000	1.000	1.000
AC-5mg/kg (n=3)	Ofloxacin (n=2)	1.333	0.248	0.389
AC-5mg/kg (n=3)	Saline (n=2)	0.333	0.564	0.615
AC-5mg/kg (n=3)	TS-30mg/kg (n=3)	0.429	0.513	0.615
AC-5mg/kg (n=3)	TS-15mg/kg (n=3)	0.048	0.827	0.876
AC-5mg/kg (n=3)	anti-tip-chimer (n=3)	3.137	0.077	0.208
AC-5mg/kg (n=3)	anti-rsPilA (n=2)	0.333	0.564	0.615
AC-10mg/kg (n=2)	Ofloxacin (n=2)	2.400	0.121	0.208
AC-10mg/kg (n=2)	Saline (n=2)	2.400	0.121	0.208
AC-10mg/kg (n=2)	TS-30mg/kg (n=3)	0.000	1.000	1.000
AC-10mg/kg (n=2)	TS-15mg/kg (n=3)	0.789	0.374	0.561
AC-10mg/kg (n=2)	anti-tip-chimer (n=3)	3.000	0.083	0.208
AC-10mg/kg (n=2)	anti-rsPilA (n=2)	1.500	0.221	0.361
Ofloxacin (n=2)	Saline (n=2)	2.400	0.121	0.208
Ofloxacin (n=2)	TS-30mg/kg (n=3)	0.333	0.564	0.615
Ofloxacin (n=2)	TS-15mg/kg (n=3)	3.000	0.083	0.208
Ofloxacin (n=2)	anti-tip-chimer (n=3)	3.000	0.083	0.208
Ofloxacin (n=2)	anti-rsPilA (n=2)	2.400	0.121	0.208
Saline (n=2)	TS-30mg/kg (n=3)	0.333	0.564	0.615
Saline (n=2)	TS-15mg/kg (n=3)	0.333	0.564	0.615
Saline (n=2)	anti-tip-chimer (n=3)	3.000	0.083	0.208
Saline (n=2)	anti-rsPilA (n=2)	2.400	0.121	0.208
TS-30mg/kg (n=3)	TS-15mg/kg (n=3)	0.429	0.513	0.615
TS-30mg/kg (n=3)	anti-tip-chimer (n=3)	3.857	0.050	0.208
TS-30mg/kg (n=3)	anti-rsPilA (n=2)	0.333	0.564	0.615
TS-15mg/kg (n=3)	anti-tip-chimer (n=3)	3.857	0.050	0.208
TS-15mg/kg (n=3)	anti-rsPilA (n=2)	3.000	0.083	0.208
anti-tip-chimer (n=3)	anti-rsPilA (n=2)	3.000	0.083	0.208

Kruskal-Wallis (Omnibus Test)

**H**	14.749
p-value	0.064

Supplementary File 3
Alpha Diversity Output

NPL Richness--Within-treatment Kruskal-Wallis pairwise comparisons

Treatment	Group 1	Group 2	H	p-value	q-value
AC-PO7d	Baseline (n=3)	2DPT (n=3)	3.857	0.050	0.416
AC-PO7d	Baseline (n=3)	5DPT (n=3)	1.190	0.275	0.459
AC-PO7d	Baseline (n=3)	7DPT (n=2)	1.333	0.248	0.459
AC-PO7d	Baseline (n=3)	9DPT (n=2)	0.333	0.564	0.626
AC-PO7d	2DPT (n=3)	5DPT (n=3)	1.190	0.275	0.459
AC-PO7d	2DPT (n=3)	7DPT (n=2)	0.333	0.564	0.626
AC-PO7d	2DPT (n=3)	9DPT (n=2)	3.000	0.083	0.416
AC-PO7d	5DPT (n=3)	7DPT (n=2)	0.333	0.564	0.626
AC-PO7d	5DPT (n=3)	9DPT (n=2)	2.193	0.139	0.459
AC-PO7d	7DPT (n=2)	9DPT (n=2)	0.167	0.683	0.683

Treatment	Group 1	Group 2	H	p-value	q-value
AC-10mg/kg	Baseline (n=2)	2DPT (n=2)	1.500	0.221	0.441
AC-10mg/kg	Baseline (n=2)	5DPT (n=2)	0.600	0.439	0.487
AC-10mg/kg	Baseline (n=2)	7DPT (n=1)	1.500	0.221	0.441
AC-10mg/kg	Baseline (n=2)	9DPT (n=2)	0.600	0.439	0.487
AC-10mg/kg	2DPT (n=2)	5DPT (n=2)	0.600	0.439	0.487
AC-10mg/kg	2DPT (n=2)	7DPT (n=1)	1.500	0.221	0.441
AC-10mg/kg	2DPT (n=2)	9DPT (n=2)	0.000	1.000	1.000
AC-10mg/kg	5DPT (n=2)	7DPT (n=1)	1.500	0.221	0.441
AC-10mg/kg	5DPT (n=2)	9DPT (n=2)	0.600	0.439	0.487
AC-10mg/kg	7DPT (n=1)	9DPT (n=2)	1.500	0.221	0.441

Treatment	Group 1	Group 2	H	p-value	q-value
AC-5mg/kg	Baseline (n=3)	2DPT (n=3)	4.091	0.043	0.116
AC-5mg/kg	Baseline (n=3)	5DPT (n=3)	0.202	0.653	0.653
AC-5mg/kg	Baseline (n=3)	7DPT (n=3)	3.971	0.046	0.116
AC-5mg/kg	Baseline (n=3)	9DPT (n=3)	0.441	0.507	0.570
AC-5mg/kg	2DPT (n=3)	5DPT (n=3)	3.971	0.046	0.116
AC-5mg/kg	2DPT (n=3)	7DPT (n=3)	3.971	0.046	0.116
AC-5mg/kg	2DPT (n=3)	9DPT (n=3)	0.441	0.507	0.570
AC-5mg/kg	5DPT (n=3)	7DPT (n=3)	0.429	0.513	0.570
AC-5mg/kg	5DPT (n=3)	9DPT (n=3)	0.455	0.500	0.570
AC-5mg/kg	7DPT (n=3)	9DPT (n=3)	0.429	0.513	0.570

Supplementary File 3
Alpha Diversity Output

NPL Richness--Within-treatment Kruskal-Wallis pairwise comparisons

Treatment	Group 1	Group 2	H	p-value	q-value
anti-rsPilA	Baseline (n=3)	2DPT (n=3)	2.333	0.127	0.317
anti-rsPilA	Baseline (n=3)	5DPT (n=2)	0.088	0.767	1.000
anti-rsPilA	Baseline (n=3)	7DPT (n=2)	0.000	1.000	1.000
anti-rsPilA	Baseline (n=3)	9DPT (n=2)	3.000	0.083	0.317
anti-rsPilA	2DPT (n=3)	5DPT (n=2)	0.000	1.000	1.000
anti-rsPilA	2DPT (n=3)	7DPT (n=2)	0.333	0.564	0.940
anti-rsPilA	2DPT (n=3)	9DPT (n=2)	3.000	0.083	0.317
anti-rsPilA	5DPT (n=2)	7DPT (n=2)	0.600	0.439	0.877
anti-rsPilA	5DPT (n=2)	9DPT (n=2)	0.000	1.000	1.000
anti-rsPilA	7DPT (n=2)	9DPT (n=2)	2.400	0.121	0.317

Treatment	Group 1	Group 2	H	p-value	q-value
anti-tip-chimer	Baseline (n=3)	2DPT (n=3)	0.048	0.827	0.919
anti-tip-chimer	Baseline (n=3)	5DPT (n=3)	0.429	0.513	0.854
anti-tip-chimer	Baseline (n=3)	7DPT (n=2)	0.000	1.000	1.000
anti-tip-chimer	Baseline (n=3)	9DPT (n=3)	1.190	0.275	0.688
anti-tip-chimer	2DPT (n=3)	5DPT (n=3)	0.784	0.376	0.752
anti-tip-chimer	2DPT (n=3)	7DPT (n=2)	0.088	0.767	0.919
anti-tip-chimer	2DPT (n=3)	9DPT (n=3)	3.857	0.050	0.248
anti-tip-chimer	5DPT (n=3)	7DPT (n=2)	0.088	0.767	0.919
anti-tip-chimer	5DPT (n=3)	9DPT (n=3)	3.857	0.050	0.248
anti-tip-chimer	7DPT (n=2)	9DPT (n=3)	3.000	0.083	0.278

Treatment	Group 1	Group 2	H	p-value	q-value
Ofloxacin	Baseline (n=3)	2DPT (n=3)	0.429	0.513	0.570
Ofloxacin	Baseline (n=3)	5DPT (n=3)	0.455	0.500	0.570
Ofloxacin	Baseline (n=3)	7DPT (n=3)	0.784	0.376	0.570
Ofloxacin	Baseline (n=3)	9DPT (n=2)	3.000	0.083	0.278
Ofloxacin	2DPT (n=3)	5DPT (n=3)	1.190	0.275	0.550
Ofloxacin	2DPT (n=3)	7DPT (n=3)	0.429	0.513	0.570
Ofloxacin	2DPT (n=3)	9DPT (n=2)	0.088	0.767	0.767
Ofloxacin	5DPT (n=3)	7DPT (n=3)	1.190	0.275	0.550
Ofloxacin	5DPT (n=3)	9DPT (n=2)	3.000	0.083	0.278
Ofloxacin	7DPT (n=3)	9DPT (n=2)	3.000	0.083	0.278

Supplementary File 3
Alpha Diversity Output

NPL Richness--Within-treatment Kruskal-Wallis pairwise comparisons

Treatment	Group 1	Group 2	H	p-value	q-value
TS-30mg/kg	Baseline (n=3)	2DPT (n=3)	0.048	0.827	1.000
TS-30mg/kg	Baseline (n=3)	5DPT (n=3)	3.857	0.050	0.495
TS-30mg/kg	Baseline (n=3)	7DPT (n=2)	0.088	0.767	1.000
TS-30mg/kg	Baseline (n=3)	9DPT (n=3)	0.429	0.513	1.000
TS-30mg/kg	2DPT (n=3)	5DPT (n=3)	0.429	0.513	1.000
TS-30mg/kg	2DPT (n=3)	7DPT (n=2)	0.000	1.000	1.000
TS-30mg/kg	2DPT (n=3)	9DPT (n=3)	0.196	0.658	1.000
TS-30mg/kg	5DPT (n=3)	7DPT (n=2)	0.088	0.767	1.000
TS-30mg/kg	5DPT (n=3)	9DPT (n=3)	0.429	0.513	1.000
TS-30mg/kg	7DPT (n=2)	9DPT (n=3)	0.000	1.000	1.000

Treatment	Group 1	Group 2	H	p-value	q-value
TS-15mg/kg	Baseline (n=3)	2DPT (n=3)	0.429	0.513	0.827
TS-15mg/kg	Baseline (n=3)	5DPT (n=3)	0.784	0.376	0.827
TS-15mg/kg	Baseline (n=3)	7DPT (n=3)	0.455	0.500	0.827
TS-15mg/kg	Baseline (n=3)	9DPT (n=3)	0.429	0.513	0.827
TS-15mg/kg	2DPT (n=3)	5DPT (n=3)	0.048	0.827	0.827
TS-15mg/kg	2DPT (n=3)	7DPT (n=3)	0.429	0.513	0.827
TS-15mg/kg	2DPT (n=3)	9DPT (n=3)	0.196	0.658	0.827
TS-15mg/kg	5DPT (n=3)	7DPT (n=3)	0.196	0.658	0.827
TS-15mg/kg	5DPT (n=3)	9DPT (n=3)	0.048	0.827	0.827
TS-15mg/kg	7DPT (n=3)	9DPT (n=3)	0.048	0.827	0.827

Treatment	Group 1	Group 2	H	p-value	q-value
Saline	Baseline (n=3)	2DPT (n=3)	0.196	0.658	0.731
Saline	Baseline (n=3)	5DPT (n=3)	0.049	0.825	0.825
Saline	Baseline (n=3)	7DPT (n=3)	0.429	0.513	0.705
Saline	Baseline (n=3)	9DPT (n=2)	3.000	0.083	0.416
Saline	2DPT (n=3)	5DPT (n=3)	1.225	0.268	0.537
Saline	2DPT (n=3)	7DPT (n=3)	0.429	0.513	0.705
Saline	2DPT (n=3)	9DPT (n=2)	2.193	0.139	0.462
Saline	5DPT (n=3)	7DPT (n=3)	1.225	0.268	0.537
Saline	5DPT (n=3)	9DPT (n=2)	3.158	0.076	0.416
Saline	7DPT (n=3)	9DPT (n=2)	0.333	0.564	0.705

Supplementary File 3
Alpha Diversity Output

id	Chinchilla_ID	Treatment	Sex	Collection	NPL shannon_entropy
1BN	Chinch1	AC-PO7d	F	Baseline	5.476792415
2BN	Chinch2	AC-PO7d	F	Baseline	5.895184006
3BN	Chinch3	AC-PO7d	M	Baseline	4.94786989
20NBL	Chinch20	AC-5mg/kg	F	Baseline	5.241905766
23NBL	Chinch23	AC-5mg/kg	F	Baseline	5.508855655
26NBL	Chinch26	AC-5mg/kg	M	Baseline	5.42784322
22NBL	Chinch22	AC-10mg/kg	M	Baseline	5.526665936
25NBL	Chinch25	AC-10mg/kg	F	Baseline	5.831372131
11NBL	Chinch11	anti-rsPilA	F	Baseline	6.03888693
13NBL	Chinch13	anti-rsPilA	F	Baseline	5.782579073
17NBL	Chinch17	anti-rsPilA	F	Baseline	5.766962783
19NBL	Chinch19	anti-tip-chimer	F	Baseline	5.722770116
21NBL	Chinch21	anti-tip-chimer	F	Baseline	5.474086486
27NBL	Chinch27	anti-tip-chimer	M	Baseline	5.874157367
4NBL	Chinch4	Ofloxacin	F	Baseline	5.379834352
5NBL	Chinch5	Ofloxacin	F	Baseline	5.529056512
7NBL	Chinch7	Ofloxacin	M	Baseline	5.294308948
14NBL	Chinch14	TS-15mg/kg	F	Baseline	5.59177832
15NBL	Chinch15	TS-15mg/kg	F	Baseline	5.442036751
18NBL	Chinch18	TS-15mg/kg	M	Baseline	5.482843089
10NBL	Chinch10	TS-30mg/kg	F	Baseline	5.605821052
12NBL	Chinch12	TS-30mg/kg	M	Baseline	5.729428117
16NBL	Chinch16	TS-30mg/kg	F	Baseline	5.459978275
6NBL	Chinch6	Saline	F	Baseline	5.06231506
8NBL	Chinch8	Saline	M	Baseline	5.417825021
9NBL	Chinch9	Saline	F	Baseline	5.488734788
1AN2	Chinch1	AC-PO7d	F	1	4.761904746
2AN2	Chinch2	AC-PO7d	F	1	4.934703715
3AN2	Chinch3	AC-PO7d	M	1	4.983536685
20N1	Chinch20	AC-5mg/kg	F	1	5.330213318
23N1	Chinch23	AC-5mg/kg	F	1	5.544306205
26N1	Chinch26	AC-5mg/kg	M	1	5.45981509
22N1	Chinch22	AC-10mg/kg	M	1	5.630033494
25N1	Chinch25	AC-10mg/kg	F	1	5.409969413
11N1	Chinch11	anti-rsPilA	F	1	5.726155978
13N1	Chinch13	anti-rsPilA	F	1	5.717840118
17N1	Chinch17	anti-rsPilA	F	1	5.532949133
19N1	Chinch19	anti-tip-chimer	F	1	5.916064407
21N1	Chinch21	anti-tip-chimer	F	1	5.608896418
27N1	Chinch27	anti-tip-chimer	M	1	5.576738835
4N1	Chinch4	Ofloxacin	F	1	5.173992085
5N1	Chinch5	Ofloxacin	F	1	5.372591889
7N1	Chinch7	Ofloxacin	M	1	4.935005815
14N1	Chinch14	TS-15mg/kg	F	1	5.943524387
15N1	Chinch15	TS-15mg/kg	F	1	6.11959826
18N1	Chinch18	TS-15mg/kg	M	1	5.141147075
10N1	Chinch10	TS-30mg/kg	F	1	4.885296803

Supplementary File 3
Alpha Diversity Output

id	Chinchilla_ID	Treatment	Sex	Collection	NPL shannon_entropy
12N1	Chinch12	TS-30mg/kg	M	1	5.599694825
16N1	Chinch16	TS-30mg/kg	F	1	5.691673547
6N1	Chinch6	Saline	F	1	5.142258637
8N1	Chinch8	Saline	M	1	5.554975328
9N1	Chinch9	Saline	F	1	5.588388037
1AN5	Chinch1	AC-PO7d	F	2	5.0458289
2AN5	Chinch2	AC-PO7d	F	2	5.628943855
3AN5	Chinch3	AC-PO7d	M	2	4.770793812
20N2	Chinch20	AC-5mg/kg	F	2	4.765306104
23N2	Chinch23	AC-5mg/kg	F	2	5.566709899
26N2	Chinch26	AC-5mg/kg	M	2	5.523538263
22N2	Chinch22	AC-10mg/kg	M	2	5.728417359
25N2	Chinch25	AC-10mg/kg	F	2	5.379053018
11N2	Chinch11	anti-rsPilA	F	2	5.907998848
17N2	Chinch17	anti-rsPilA	F	2	5.455525011
19N2	Chinch19	anti-tip-chimer	F	2	5.703150825
21N2	Chinch21	anti-tip-chimer	F	2	5.481798027
27N2	Chinch27	anti-tip-chimer	M	2	5.656966931
4N2	Chinch4	Ofloxacin	F	2	5.369032723
5N2	Chinch5	Ofloxacin	F	2	5.504240226
7N2	Chinch7	Ofloxacin	M	2	5.426393396
14N2	Chinch14	TS-15mg/kg	F	2	5.418550656
15N2	Chinch15	TS-15mg/kg	F	2	6.336382954
18N2	Chinch18	TS-15mg/kg	M	2	5.458119298
10N2	Chinch10	TS-30mg/kg	F	2	5.100197018
12N2	Chinch12	TS-30mg/kg	M	2	5.390786135
16N2	Chinch16	TS-30mg/kg	F	2	5.541128444
6N2	Chinch6	Saline	F	2	5.321957931
8N2	Chinch8	Saline	M	2	5.585409587
9N2	Chinch9	Saline	F	2	3.333143971
1AN7	Chinch1	AC-PO7d	F	3	5.051974992
2AN7	Chinch2	AC-PO7d	F	3	3.835691593
20N3	Chinch20	AC-5mg/kg	F	3	5.11045432
23N3	Chinch23	AC-5mg/kg	F	3	5.388350847
26N3	Chinch26	AC-5mg/kg	M	3	5.244430634
25N3	Chinch25	AC-10mg/kg	F	3	5.246968554
11N3	Chinch11	anti-rsPilA	F	3	5.805136856
17N3	Chinch17	anti-rsPilA	F	3	5.711714601
21N3	Chinch21	anti-tip-chimer	F	3	5.625223101
27N3	Chinch27	anti-tip-chimer	M	3	5.461116658
4N3	Chinch4	Ofloxacin	F	3	5.320987512
5N3	Chinch5	Ofloxacin	F	3	4.958461824
7N3	Chinch7	Ofloxacin	M	3	5.264841236
14N3	Chinch14	TS-15mg/kg	F	3	5.386347964
15N3	Chinch15	TS-15mg/kg	F	3	5.882057222
18N3	Chinch18	TS-15mg/kg	M	3	5.355475075
10N3	Chinch10	TS-30mg/kg	F	3	5.091809823
16N3	Chinch16	TS-30mg/kg	F	3	5.859897345

Supplementary File 3
Alpha Diversity Output

id	Chinchilla_ID	Treatment	Sex	Collection	NPL shannon_entropy
6N3	Chinch6	Saline	F	3	5.565486165
8N3	Chinch8	Saline	M	3	5.822611813
9N3	Chinch9	Saline	F	3	3.562572527
2AN9	Chinch2	AC-PO7d	F	4	4.741126038
3AN9	Chinch3	AC-PO7d	M	4	4.988652556
20N4	Chinch20	AC-5mg/kg	F	4	4.680899941
23N4	Chinch23	AC-5mg/kg	F	4	5.752831686
26N4	Chinch26	AC-5mg/kg	M	4	5.313672246
22N4	Chinch22	AC-10mg/kg	M	4	5.081473103
25N4	Chinch25	AC-10mg/kg	F	4	5.318871459
11N4	Chinch11	anti-rsPilA	F	4	5.325752939
17N4	Chinch17	anti-rsPilA	F	4	5.402872792
19N4	Chinch19	anti-tip-chimer	F	4	5.976590136
21N4	Chinch21	anti-tip-chimer	F	4	6.268439952
27N4	Chinch27	anti-tip-chimer	M	4	5.788633839
5N4	Chinch5	Ofloxacin	F	4	4.643701748
7N4	Chinch7	Ofloxacin	M	4	5.189214369
14N4	Chinch14	TS-15mg/kg	F	4	5.585089154
15N4	Chinch15	TS-15mg/kg	F	4	5.9068365
18N4	Chinch18	TS-15mg/kg	M	4	5.295544339
10N4	Chinch10	TS-30mg/kg	F	4	4.781199663
12N4	Chinch12	TS-30mg/kg	M	4	5.635325026
16N4	Chinch16	TS-30mg/kg	F	4	5.687944169
6N4	Chinch6	Saline	F	4	5.480431612
8N4	Chinch8	Saline	M	4	5.657882243

Supplementary File 3
Alpha Diversity Output

NPL Shannon Diversity-Baseline

Kruskal-Wallis pairwise comparisons

Group 1	Group 2	H	p-value	q-value
AC-PO7d (n=3)	AC-5mg/kg (n=3)	0.048	0.827	0.851
AC-PO7d (n=3)	AC-10mg/kg (n=2)	0.333	0.564	0.752
AC-PO7d (n=3)	Ofloxacin (n=3)	0.048	0.827	0.851
AC-PO7d (n=3)	Saline (n=3)	0.048	0.827	0.851
AC-PO7d (n=3)	TS-30mg/kg (n=3)	0.048	0.827	0.851
AC-PO7d (n=3)	TS-15mg/kg (n=3)	0.048	0.827	0.851
AC-PO7d (n=3)	anti-tip-chimer (n=3)	0.048	0.827	0.851
AC-PO7d (n=3)	anti-rsPilA (n=3)	1.190	0.275	0.450
AC-5mg/kg (n=3)	AC-10mg/kg (n=2)	3.000	0.083	0.351
AC-5mg/kg (n=3)	Ofloxacin (n=3)	0.048	0.827	0.851
AC-5mg/kg (n=3)	Saline (n=3)	0.429	0.513	0.752
AC-5mg/kg (n=3)	TS-30mg/kg (n=3)	2.333	0.127	0.351
AC-5mg/kg (n=3)	TS-15mg/kg (n=3)	1.190	0.275	0.450
AC-5mg/kg (n=3)	anti-tip-chimer (n=3)	2.333	0.127	0.351
AC-5mg/kg (n=3)	anti-rsPilA (n=3)	3.857	0.050	0.351
AC-10mg/kg (n=2)	Ofloxacin (n=3)	1.333	0.248	0.450
AC-10mg/kg (n=2)	Saline (n=3)	3.000	0.083	0.351
AC-10mg/kg (n=2)	TS-30mg/kg (n=3)	0.333	0.564	0.752
AC-10mg/kg (n=2)	TS-15mg/kg (n=3)	1.333	0.248	0.450
AC-10mg/kg (n=2)	anti-tip-chimer (n=3)	0.000	1.000	1.000
AC-10mg/kg (n=2)	anti-rsPilA (n=3)	0.333	0.564	0.752
Ofloxacin (n=3)	Saline (n=3)	0.048	0.827	0.851
Ofloxacin (n=3)	TS-30mg/kg (n=3)	2.333	0.127	0.351
Ofloxacin (n=3)	TS-15mg/kg (n=3)	1.190	0.275	0.450
Ofloxacin (n=3)	anti-tip-chimer (n=3)	2.333	0.127	0.351
Ofloxacin (n=3)	anti-rsPilA (n=3)	3.857	0.050	0.351
Saline (n=3)	TS-30mg/kg (n=3)	2.333	0.127	0.351
Saline (n=3)	TS-15mg/kg (n=3)	1.190	0.275	0.450
Saline (n=3)	anti-tip-chimer (n=3)	2.333	0.127	0.351
Saline (n=3)	anti-rsPilA (n=3)	3.857	0.050	0.351
TS-30mg/kg (n=3)	TS-15mg/kg (n=3)	1.190	0.275	0.450
TS-30mg/kg (n=3)	anti-tip-chimer (n=3)	0.429	0.513	0.752
TS-30mg/kg (n=3)	anti-rsPilA (n=3)	3.857	0.050	0.351
TS-15mg/kg (n=3)	anti-tip-chimer (n=3)	1.190	0.275	0.450
TS-15mg/kg (n=3)	anti-rsPilA (n=3)	3.857	0.050	0.351
anti-tip-chimer (n=3)	anti-rsPilA (n=3)	1.190	0.275	0.450

Kruskal-Wallis (Omnibus Test)

**H**	12.020
**p-value**	0.150

Supplementary File 3
Alpha Diversity Output

NPL Shannon Diversity-2DPT

Kruskal-Wallis pairwise comparisons

Group 1	Group 2	H	p-value	q-value
AC-PO7d (n=3)	AC-5mg/kg (n=3)	3.857	0.050	0.223
AC-PO7d (n=3)	AC-10mg/kg (n=2)	3.000	0.083	0.300
AC-PO7d (n=3)	Ofloxacin (n=3)	2.333	0.127	0.326
AC-PO7d (n=3)	Saline (n=3)	3.857	0.050	0.223
AC-PO7d (n=3)	TS-30mg/kg (n=3)	1.190	0.275	0.472
AC-PO7d (n=3)	TS-15mg/kg (n=3)	3.857	0.050	0.223
AC-PO7d (n=3)	anti-tip-chimer (n=3)	3.857	0.050	0.223
AC-PO7d (n=3)	anti-rsPilA (n=3)	3.857	0.050	0.223
AC-5mg/kg (n=3)	AC-10mg/kg (n=2)	0.333	0.564	0.597
AC-5mg/kg (n=3)	Ofloxacin (n=3)	2.333	0.127	0.326
AC-5mg/kg (n=3)	Saline (n=3)	0.429	0.513	0.597
AC-5mg/kg (n=3)	TS-30mg/kg (n=3)	0.429	0.513	0.597
AC-5mg/kg (n=3)	TS-15mg/kg (n=3)	0.429	0.513	0.597
AC-5mg/kg (n=3)	anti-tip-chimer (n=3)	3.857	0.050	0.223
AC-5mg/kg (n=3)	anti-rsPilA (n=3)	2.333	0.127	0.326
AC-10mg/kg (n=2)	Ofloxacin (n=3)	3.000	0.083	0.300
AC-10mg/kg (n=2)	Saline (n=3)	0.333	0.564	0.597
AC-10mg/kg (n=2)	TS-30mg/kg (n=3)	0.000	1.000	1.000
AC-10mg/kg (n=2)	TS-15mg/kg (n=3)	0.333	0.564	0.597
AC-10mg/kg (n=2)	anti-tip-chimer (n=3)	0.333	0.564	0.597
AC-10mg/kg (n=2)	anti-rsPilA (n=3)	1.333	0.248	0.472
Ofloxacin (n=3)	Saline (n=3)	1.190	0.275	0.472
Ofloxacin (n=3)	TS-30mg/kg (n=3)	0.429	0.513	0.597
Ofloxacin (n=3)	TS-15mg/kg (n=3)	1.190	0.275	0.472
Ofloxacin (n=3)	anti-tip-chimer (n=3)	3.857	0.050	0.223
Ofloxacin (n=3)	anti-rsPilA (n=3)	3.857	0.050	0.223
Saline (n=3)	TS-30mg/kg (n=3)	0.429	0.513	0.597
Saline (n=3)	TS-15mg/kg (n=3)	0.429	0.513	0.597
Saline (n=3)	anti-tip-chimer (n=3)	2.333	0.127	0.326
Saline (n=3)	anti-rsPilA (n=3)	1.190	0.275	0.472
TS-30mg/kg (n=3)	TS-15mg/kg (n=3)	1.190	0.275	0.472
TS-30mg/kg (n=3)	anti-tip-chimer (n=3)	0.429	0.513	0.597
TS-30mg/kg (n=3)	anti-rsPilA (n=3)	1.190	0.275	0.472
TS-15mg/kg (n=3)	anti-tip-chimer (n=3)	0.429	0.513	0.597
TS-15mg/kg (n=3)	anti-rsPilA (n=3)	0.429	0.513	0.597
anti-tip-chimer (n=3)	anti-rsPilA (n=3)	0.048	0.827	0.851

Kruskal-Wallis (Omnibus Test)

**H**	13.174
**p-value**	0.106

Supplementary File 3
Alpha Diversity Output

NPL Shannon Diversity-5DPT

Kruskal-Wallis pairwise comparisons

Group 1	Group 2	H	p-value	q-value
AC-PO7d (n=3)	AC-5mg/kg (n=3)	0.048	0.827	0.902
AC-PO7d (n=3)	AC-10mg/kg (n=2)	1.333	0.248	0.708
AC-PO7d (n=3)	Ofloxacin (n=3)	0.429	0.513	0.781
AC-PO7d (n=3)	Saline (n=3)	0.048	0.827	0.902
AC-PO7d (n=3)	TS-30mg/kg (n=3)	0.429	0.513	0.781
AC-PO7d (n=3)	TS-15mg/kg (n=3)	1.190	0.275	0.708
AC-PO7d (n=3)	anti-tip-chimer (n=3)	2.333	0.127	0.708
AC-PO7d (n=3)	anti-rsPilA (n=2)	1.333	0.248	0.708
AC-5mg/kg (n=3)	AC-10mg/kg (n=2)	0.333	0.564	0.781
AC-5mg/kg (n=3)	Ofloxacin (n=3)	0.429	0.513	0.781
AC-5mg/kg (n=3)	Saline (n=3)	0.048	0.827	0.902
AC-5mg/kg (n=3)	TS-30mg/kg (n=3)	0.048	0.827	0.902
AC-5mg/kg (n=3)	TS-15mg/kg (n=3)	0.048	0.827	0.902
AC-5mg/kg (n=3)	anti-tip-chimer (n=3)	1.190	0.275	0.708
AC-5mg/kg (n=3)	anti-rsPilA (n=2)	0.333	0.564	0.781
AC-10mg/kg (n=2)	Ofloxacin (n=3)	0.333	0.564	0.781
AC-10mg/kg (n=2)	Saline (n=3)	1.333	0.248	0.708
AC-10mg/kg (n=2)	TS-30mg/kg (n=3)	0.333	0.564	0.781
AC-10mg/kg (n=2)	TS-15mg/kg (n=3)	0.333	0.564	0.781
AC-10mg/kg (n=2)	anti-tip-chimer (n=3)	0.000	1.000	1.000
AC-10mg/kg (n=2)	anti-rsPilA (n=2)	0.600	0.439	0.781
Ofloxacin (n=3)	Saline (n=3)	0.429	0.513	0.781
Ofloxacin (n=3)	TS-30mg/kg (n=3)	0.048	0.827	0.902
Ofloxacin (n=3)	TS-15mg/kg (n=3)	0.429	0.513	0.781
Ofloxacin (n=3)	anti-tip-chimer (n=3)	2.333	0.127	0.708
Ofloxacin (n=3)	anti-rsPilA (n=2)	1.333	0.248	0.708
Saline (n=3)	TS-30mg/kg (n=3)	0.048	0.827	0.902
Saline (n=3)	TS-15mg/kg (n=3)	1.190	0.275	0.708
Saline (n=3)	anti-tip-chimer (n=3)	2.333	0.127	0.708
Saline (n=3)	anti-rsPilA (n=2)	1.333	0.248	0.708
TS-30mg/kg (n=3)	TS-15mg/kg (n=3)	1.190	0.275	0.708
TS-30mg/kg (n=3)	anti-tip-chimer (n=3)	2.333	0.127	0.708
TS-30mg/kg (n=3)	anti-rsPilA (n=2)	1.333	0.248	0.708
TS-15mg/kg (n=3)	anti-tip-chimer (n=3)	0.429	0.513	0.781
TS-15mg/kg (n=3)	anti-rsPilA (n=2)	0.000	1.000	1.000
anti-tip-chimer (n=3)	anti-rsPilA (n=2)	0.000	1.000	1.000

Kruskal-Wallis (Omnibus Test)

**H**	6.243
**p-value**	0.620

Supplementary File 3
Alpha Diversity Output

NPL Shannon Diversity-7DPT

Kruskal-Wallis pairwise comparisons

Group 1	Group 2	H	p-value	q-value
AC-PO7d (n=2)	AC-5mg/kg (n=3)	3.000	0.083	0.397
AC-PO7d (n=2)	AC-10mg/kg (n=1)	1.500	0.221	0.530
AC-PO7d (n=2)	Ofloxacin (n=3)	1.333	0.248	0.558
AC-PO7d (n=2)	Saline (n=3)	0.333	0.564	0.781
AC-PO7d (n=2)	TS-30mg/kg (n=2)	2.400	0.121	0.397
AC-PO7d (n=2)	TS-15mg/kg (n=3)	3.000	0.083	0.397
AC-PO7d (n=2)	anti-tip-chimer (n=2)	2.400	0.121	0.397
AC-PO7d (n=2)	anti-rsPilA (n=2)	2.400	0.121	0.397
AC-5mg/kg (n=3)	AC-10mg/kg (n=1)	0.200	0.655	0.813
AC-5mg/kg (n=3)	Ofloxacin (n=3)	0.048	0.827	0.961
AC-5mg/kg (n=3)	Saline (n=3)	0.429	0.513	0.781
AC-5mg/kg (n=3)	TS-30mg/kg (n=2)	0.000	1.000	1.000
AC-5mg/kg (n=3)	TS-15mg/kg (n=3)	1.190	0.275	0.583
AC-5mg/kg (n=3)	anti-tip-chimer (n=2)	3.000	0.083	0.397
AC-5mg/kg (n=3)	anti-rsPilA (n=2)	3.000	0.083	0.397
AC-10mg/kg (n=1)	Ofloxacin (n=3)	0.200	0.655	0.813
AC-10mg/kg (n=1)	Saline (n=3)	0.200	0.655	0.813
AC-10mg/kg (n=1)	TS-30mg/kg (n=2)	0.000	1.000	1.000
AC-10mg/kg (n=1)	TS-15mg/kg (n=3)	1.800	0.180	0.530
AC-10mg/kg (n=1)	anti-tip-chimer (n=2)	1.500	0.221	0.530
AC-10mg/kg (n=1)	anti-rsPilA (n=2)	1.500	0.221	0.530
Ofloxacin (n=3)	Saline (n=3)	0.429	0.513	0.781
Ofloxacin (n=3)	TS-30mg/kg (n=2)	0.333	0.564	0.781
Ofloxacin (n=3)	TS-15mg/kg (n=3)	3.857	0.050	0.397
Ofloxacin (n=3)	anti-tip-chimer (n=2)	3.000	0.083	0.397
Ofloxacin (n=3)	anti-rsPilA (n=2)	3.000	0.083	0.397
Saline (n=3)	TS-30mg/kg (n=2)	0.333	0.564	0.781
Saline (n=3)	TS-15mg/kg (n=3)	0.048	0.827	0.961
Saline (n=3)	anti-tip-chimer (n=2)	0.000	1.000	1.000
Saline (n=3)	anti-rsPilA (n=2)	0.333	0.564	0.781
TS-30mg/kg (n=2)	TS-15mg/kg (n=3)	0.333	0.564	0.781
TS-30mg/kg (n=2)	anti-tip-chimer (n=2)	0.000	1.000	1.000
TS-30mg/kg (n=2)	anti-rsPilA (n=2)	0.000	1.000	1.000
TS-15mg/kg (n=3)	anti-tip-chimer (n=2)	0.333	0.564	0.781
TS-15mg/kg (n=3)	anti-rsPilA (n=2)	0.333	0.564	0.781
anti-tip-chimer (n=2)	anti-rsPilA (n=2)	2.400	0.121	0.397

Kruskal-Wallis (Omnibus Test)

**H**	9.255
**p-value**	0.321

Supplementary File 3
Alpha Diversity Output

NPL Shannon Diversity-9DPT

Kruskal-Wallis pairwise comparisons

Group 1	Group 2	H	p-value	q-value
AC-PO7d (n=2)	AC-5mg/kg (n=3)	0.333	0.564	0.676
AC-PO7d (n=2)	AC-10mg/kg (n=2)	2.400	0.121	0.253
AC-PO7d (n=2)	Ofloxacin (n=2)	0.000	1.000	1.000
AC-PO7d (n=2)	Saline (n=2)	2.400	0.121	0.253
AC-PO7d (n=2)	TS-30mg/kg (n=3)	1.333	0.248	0.406
AC-PO7d (n=2)	TS-15mg/kg (n=3)	3.000	0.083	0.253
AC-PO7d (n=2)	anti-tip-chimer (n=3)	3.000	0.083	0.253
AC-PO7d (n=2)	anti-rsPilA (n=2)	2.400	0.121	0.253
AC-5mg/kg (n=3)	AC-10mg/kg (n=2)	0.000	1.000	1.000
AC-5mg/kg (n=3)	Ofloxacin (n=2)	1.333	0.248	0.406
AC-5mg/kg (n=3)	Saline (n=2)	0.333	0.564	0.676
AC-5mg/kg (n=3)	TS-30mg/kg (n=3)	0.048	0.827	0.931
AC-5mg/kg (n=3)	TS-15mg/kg (n=3)	0.429	0.513	0.676
AC-5mg/kg (n=3)	anti-tip-chimer (n=3)	3.857	0.050	0.253
AC-5mg/kg (n=3)	anti-rsPilA (n=2)	0.333	0.564	0.676
AC-10mg/kg (n=2)	Ofloxacin (n=2)	0.600	0.439	0.676
AC-10mg/kg (n=2)	Saline (n=2)	2.400	0.121	0.253
AC-10mg/kg (n=2)	TS-30mg/kg (n=3)	0.333	0.564	0.676
AC-10mg/kg (n=2)	TS-15mg/kg (n=3)	1.333	0.248	0.406
AC-10mg/kg (n=2)	anti-tip-chimer (n=3)	3.000	0.083	0.253
AC-10mg/kg (n=2)	anti-rsPilA (n=2)	2.400	0.121	0.253
Ofloxacin (n=2)	Saline (n=2)	2.400	0.121	0.253
Ofloxacin (n=2)	TS-30mg/kg (n=3)	1.333	0.248	0.406
Ofloxacin (n=2)	TS-15mg/kg (n=3)	3.000	0.083	0.253
Ofloxacin (n=2)	anti-tip-chimer (n=3)	3.000	0.083	0.253
Ofloxacin (n=2)	anti-rsPilA (n=2)	2.400	0.121	0.253
Saline (n=2)	TS-30mg/kg (n=3)	0.000	1.000	1.000
Saline (n=2)	TS-15mg/kg (n=3)	0.000	1.000	1.000
Saline (n=2)	anti-tip-chimer (n=3)	3.000	0.083	0.253
Saline (n=2)	anti-rsPilA (n=2)	2.400	0.121	0.253
TS-30mg/kg (n=3)	TS-15mg/kg (n=3)	0.048	0.827	0.931
TS-30mg/kg (n=3)	anti-tip-chimer (n=3)	3.857	0.050	0.253
TS-30mg/kg (n=3)	anti-rsPilA (n=2)	0.333	0.564	0.676
TS-15mg/kg (n=3)	anti-tip-chimer (n=3)	2.333	0.127	0.253
TS-15mg/kg (n=3)	anti-rsPilA (n=2)	0.333	0.564	0.676
anti-tip-chimer (n=3)	anti-rsPilA (n=2)	3.000	0.083	0.253

Kruskal-Wallis (Omnibus Test)

**H**	13.024
p-value	0.111

Supplementary File 3
Alpha Diversity Output

NPL Shannon Diversity--Within-treatment Kruskal-Wallis pairwise comparisons

Treatment	Group 1	Group 2	H	p-value	q-value
AC-PO7d	0 (n=3)	2 (n=3)	2.333	0.127	0.550
AC-PO7d	0 (n=3)	5 (n=3)	0.429	0.513	0.805
AC-PO7d	0 (n=3)	7 (n=2)	1.333	0.248	0.550
AC-PO7d	0 (n=3)	9 (n=2)	1.333	0.248	0.550
AC-PO7d	2 (n=3)	5 (n=3)	1.190	0.275	0.550
AC-PO7d	2 (n=3)	7 (n=2)	0.000	1.000	1.000
AC-PO7d	2 (n=3)	9 (n=2)	0.000	1.000	1.000
AC-PO7d	5 (n=3)	7 (n=2)	0.333	0.564	0.805
AC-PO7d	5 (n=3)	9 (n=2)	1.333	0.248	0.550
AC-PO7d	7 (n=2)	9 (n=2)	0.000	1.000	1.000

Treatment	Group 1	Group 2	H	p-value	q-value
AC-10mg/kg	0 (n=2)	2 (n=2)	0.600	0.439	0.548
AC-10mg/kg	0 (n=2)	5 (n=2)	0.600	0.439	0.548
AC-10mg/kg	0 (n=2)	7 (n=1)	1.500	0.221	0.368
AC-10mg/kg	0 (n=2)	9 (n=2)	2.400	0.121	0.368
AC-10mg/kg	2 (n=2)	5 (n=2)	0.000	1.000	1.000
AC-10mg/kg	2 (n=2)	7 (n=1)	1.500	0.221	0.368
AC-10mg/kg	2 (n=2)	9 (n=2)	2.400	0.121	0.368
AC-10mg/kg	5 (n=2)	7 (n=1)	1.500	0.221	0.368
AC-10mg/kg	5 (n=2)	9 (n=2)	2.400	0.121	0.368
AC-10mg/kg	7 (n=1)	9 (n=2)	0.000	1.000	1.000

Treatment	Group 1	Group 2	H	p-value	q-value
AC-5mg/kg	0 (n=3)	2 (n=3)	0.429	0.513	0.827
AC-5mg/kg	0 (n=3)	5 (n=3)	0.429	0.513	0.827
AC-5mg/kg	0 (n=3)	7 (n=3)	1.190	0.275	0.827
AC-5mg/kg	0 (n=3)	9 (n=3)	0.048	0.827	0.827
AC-5mg/kg	2 (n=3)	5 (n=3)	0.048	0.827	0.827
AC-5mg/kg	2 (n=3)	7 (n=3)	2.333	0.127	0.827
AC-5mg/kg	2 (n=3)	9 (n=3)	0.429	0.513	0.827
AC-5mg/kg	5 (n=3)	7 (n=3)	0.429	0.513	0.827
AC-5mg/kg	5 (n=3)	9 (n=3)	0.048	0.827	0.827
AC-5mg/kg	7 (n=3)	9 (n=3)	0.048	0.827	0.827

Supplementary File 3
Alpha Diversity Output

NPL Shannon Diversity--Within-treatment Kruskal-Wallis pairwise comparisons

Treatment	Group 1	Group 2	H	p-value	q-value
anti-rsPilA	0 (n=3)	2 (n=3)	3.857	0.050	0.243
anti-rsPilA	0 (n=3)	5 (n=2)	0.333	0.564	0.705
anti-rsPilA	0 (n=3)	7 (n=2)	0.333	0.564	0.705
anti-rsPilA	0 (n=3)	9 (n=2)	3.000	0.083	0.243
anti-rsPilA	2 (n=3)	5 (n=2)	0.000	1.000	1.000
anti-rsPilA	2 (n=3)	7 (n=2)	0.333	0.564	0.705
anti-rsPilA	2 (n=3)	9 (n=2)	3.000	0.083	0.243
anti-rsPilA	5 (n=2)	7 (n=2)	0.000	1.000	1.000
anti-rsPilA	5 (n=2)	9 (n=2)	2.400	0.121	0.243
anti-rsPilA	7 (n=2)	9 (n=2)	2.400	0.121	0.243

Treatment	Group 1	Group 2	H	p-value	q-value
anti-tip-chimer	0 (n=3)	2 (n=3)	0.048	0.827	0.827
anti-tip-chimer	0 (n=3)	5 (n=3)	0.429	0.513	0.705
anti-tip-chimer	0 (n=3)	7 (n=2)	1.333	0.248	0.414
anti-tip-chimer	0 (n=3)	9 (n=3)	2.333	0.127	0.317
anti-tip-chimer	2 (n=3)	5 (n=3)	0.048	0.827	0.827
anti-tip-chimer	2 (n=3)	7 (n=2)	0.333	0.564	0.705
anti-tip-chimer	2 (n=3)	9 (n=3)	2.333	0.127	0.317
anti-tip-chimer	5 (n=3)	7 (n=2)	1.333	0.248	0.414
anti-tip-chimer	5 (n=3)	9 (n=3)	3.857	0.050	0.317
anti-tip-chimer	7 (n=2)	9 (n=3)	3.000	0.083	0.317

Treatment	Group 1	Group 2	H	p-value	q-value
Ofloxacin	0 (n=3)	2 (n=3)	2.333	0.127	0.211
Ofloxacin	0 (n=3)	5 (n=3)	0.048	0.827	0.827
Ofloxacin	0 (n=3)	7 (n=3)	2.333	0.127	0.211
Ofloxacin	0 (n=3)	9 (n=2)	3.000	0.083	0.211
Ofloxacin	2 (n=3)	5 (n=3)	2.333	0.127	0.211
Ofloxacin	2 (n=3)	7 (n=3)	0.048	0.827	0.827
Ofloxacin	2 (n=3)	9 (n=2)	0.333	0.564	0.705
Ofloxacin	5 (n=3)	7 (n=3)	3.857	0.050	0.211
Ofloxacin	5 (n=3)	9 (n=2)	3.000	0.083	0.211
Ofloxacin	7 (n=3)	9 (n=2)	1.333	0.248	0.355

Supplementary File 3
Alpha Diversity Output

NPL Shannon Diversity--Within-treatment Kruskal-Wallis pairwise comparisons

Treatment	Group 1	Group 2	H	p-value	q-value
TS-30mg/kg	0 (n=3)	2 (n=3)	0.429	0.513	0.940
TS-30mg/kg	0 (n=3)	5 (n=3)	2.333	0.127	0.940
TS-30mg/kg	0 (n=3)	7 (n=2)	0.000	1.000	1.000
TS-30mg/kg	0 (n=3)	9 (n=3)	0.048	0.827	1.000
TS-30mg/kg	2 (n=3)	5 (n=3)	0.429	0.513	0.940
TS-30mg/kg	2 (n=3)	7 (n=2)	0.333	0.564	0.940
TS-30mg/kg	2 (n=3)	9 (n=3)	0.048	0.827	1.000
TS-30mg/kg	5 (n=3)	7 (n=2)	0.000	1.000	1.000
TS-30mg/kg	5 (n=3)	9 (n=3)	0.429	0.513	0.940
TS-30mg/kg	7 (n=2)	9 (n=3)	0.333	0.564	0.940

Treatment	Group 1	Group 2	H	p-value	q-value
TS-15mg/kg	0 (n=3)	2 (n=3)	0.429	0.513	0.827
TS-15mg/kg	0 (n=3)	5 (n=3)	0.048	0.827	0.827
TS-15mg/kg	0 (n=3)	7 (n=3)	0.429	0.513	0.827
TS-15mg/kg	0 (n=3)	9 (n=3)	0.048	0.827	0.827
TS-15mg/kg	2 (n=3)	5 (n=3)	0.048	0.827	0.827
TS-15mg/kg	2 (n=3)	7 (n=3)	0.429	0.513	0.827
TS-15mg/kg	2 (n=3)	9 (n=3)	0.429	0.513	0.827
TS-15mg/kg	5 (n=3)	7 (n=3)	1.190	0.275	0.827
TS-15mg/kg	5 (n=3)	9 (n=3)	0.048	0.827	0.827
TS-15mg/kg	7 (n=3)	9 (n=3)	0.048	0.827	0.827

Treatment	Group 1	Group 2	H	p-value	q-value
Saline	0 (n=3)	2 (n=3)	1.190	0.275	0.805
Saline	0 (n=3)	5 (n=3)	0.048	0.827	0.919
Saline	0 (n=3)	7 (n=3)	0.429	0.513	0.805
Saline	0 (n=3)	9 (n=2)	1.333	0.248	0.805
Saline	2 (n=3)	5 (n=3)	0.429	0.513	0.805
Saline	2 (n=3)	7 (n=3)	0.048	0.827	0.919
Saline	2 (n=3)	9 (n=2)	0.333	0.564	0.805
Saline	5 (n=3)	7 (n=3)	0.429	0.513	0.805
Saline	5 (n=3)	9 (n=2)	1.333	0.248	0.805
Saline	7 (n=3)	9 (n=2)	0.000	1.000	1.000

Supplementary File 4
ANCOM Output

Fecal 2DPT Genus Level ANCOM	W	Reject null hypothesis
d__Bacteria;p__Fusobacteriota;c__Fusobacteriia;o__Fusobacteriales;f__Fusobacteriaceae;g__Fusobacterium	66	TRUE
d__Bacteria;p__Firmicutes;c__Clostridia;o__Oscillospirales;f__Ruminococcaceae;g__Ruminococcaceae	4	FALSE
d__Bacteria;p__Firmicutes;c__Clostridia;o__Lachnospirales;f__Lachnospiraceae;__	4	FALSE
d__Bacteria;p__Firmicutes;c__Bacilli;o__Erysipelotrichales;f__Erysipelatoclostridiaceae;__	3	FALSE
d__Bacteria;p__Firmicutes;c__Clostridia;o__Christensenellales;f__Christensenellaceae;g__Christensenellaceae_R-7_group	3	FALSE
d__Bacteria;p__Firmicutes;c__Clostridia;o__Oscillospirales;f__Ruminococcaceae;g__[Eubacterium]_siraeum_group	3	FALSE
d__Bacteria;p__Proteobacteria;c__Gammaproteobacteria;o__Enterobacterales;f__Enterobacteriaceae;g__Escherichia-Shigella	3	FALSE
d__Bacteria;p__Bacteroidota;c__Bacteroidia;o__Bacteroidales;f__Prevotellaceae;g__Prevotellaceae_Ga6A1_group	2	FALSE
d__Bacteria;p__Firmicutes;c__Clostridia;o__Eubacteriales;f__Eubacteriaceae;g__uncultured	2	FALSE
d__Bacteria;p__Actinobacteriota;c__Coriobacteriia;o__Coriobacteriales;f__Eggerthellaceae;g__Paraeggerthella	2	FALSE
d__Bacteria;p__Firmicutes;c__Clostridia;o__Oscillospirales;f__Butyricicoccaceae;g__UCG-008	2	FALSE
d__Bacteria;p__Actinobacteriota;c__Actinobacteria;o__Actinomycetales;f__Actinomycetaceae;g__Actinomycetaceae	2	FALSE
d__Bacteria;p__Desulfobacterota;c__Desulfovibrionia;o__Desulfovibrionales;f__Desulfovibrionaceae;g__Desulfovibrio	1	FALSE
d__Bacteria;p__Firmicutes;c__Bacilli;o__Erysipelotrichales;f__Erysipelotrichaceae;g__Faecalibaculum	1	FALSE
d__Bacteria;p__Actinobacteriota;c__Coriobacteriia;o__Coriobacteriales;f__Eggerthellaceae;g__uncultured	1	FALSE
d__Bacteria;p__Firmicutes;c__Bacilli;o__Erysipelotrichales;f__Erysipelotrichaceae;__	1	FALSE
d__Bacteria;p__Actinobacteriota;c__Coriobacteriia;o__Coriobacteriales;f__Eggerthellaceae;__	1	FALSE
d__Bacteria;p__Firmicutes;c__Clostridia;o__Clostridia;f__Hungateiclostridiaceae;g__Ruminiclostridium	1	FALSE
d__Bacteria;p__Firmicutes;c__Clostridia;o__Oscillospirales;f__Ruminococcaceae;__	1	FALSE
d__Bacteria;p__Firmicutes;c__Clostridia;o__Lachnospirales;f__Lachnospiraceae;g__Lachnoclostridium	1	FALSE
d__Bacteria;p__Bacteroidota;c__Bacteroidia;o__Bacteroidales;f__Prevotellaceae;g__Prevotellaceae_UCG-003	1	FALSE
d__Bacteria;p__Firmicutes;c__Bacilli;o__Lactobacillales;f__Streptococcaceae;g__Streptococcus	1	FALSE
d__Bacteria;p__Firmicutes;c__Clostridia;o__Lachnospirales;f__Lachnospiraceae;g__Lachnospiraceae_UCG-001	1	FALSE
d__Bacteria;p__Firmicutes;c__Clostridia;o__Oscillospirales;f__Oscillospiraceae;g__uncultured	1	FALSE
d__Bacteria;p__Desulfobacterota;c__Desulfovibrionia;o__Desulfovibrionales;f__Desulfovibrionaceae;g__Bilophila	1	FALSE
d__Bacteria;p__Firmicutes;c__Clostridia;o__Lachnospirales;f__Lachnospiraceae;g__Lachnospira	1	FALSE
d__Bacteria;p__Bacteroidota;c__Bacteroidia;o__Bacteroidales;f__Muribaculaceae;g__Muribaculum	1	FALSE
d__Bacteria;p__Bacteroidota;c__Bacteroidia;o__Bacteroidales;f__Marinifilaceae;g__Odoribacter	1	FALSE
d__Bacteria;p__Firmicutes;c__Clostridia;o__Oscillospirales;__;__	1	FALSE
d__Bacteria;p__Actinobacteriota;c__Coriobacteriia;o__Coriobacteriales;f__Eggerthellaceae;g__Adlercreutzia	1	FALSE
d__Bacteria;p__Bacteroidota;c__Bacteroidia;o__Bacteroidales;f__Rikenellaceae;g__dgA-11_gut_group	1	FALSE
d__Bacteria;p__Actinobacteriota;c__Coriobacteriia;o__Coriobacteriales;f__Coriobacteriaceae;g__Collinsella	1	FALSE
d__Bacteria;p__Firmicutes;c__Clostridia;o__Oscillospirales;f__Oscillospiraceae;g__Oscillibacter	1	FALSE
d__Bacteria;p__Firmicutes;c__Clostridia;o__Peptostreptococcales-Tissierellales;f__Anaerovoracaceae;g__Mogibacterium	1	FALSE
d__Bacteria;p__Firmicutes;c__Clostridia;o__Lachnospirales;f__Lachnospiraceae;g__Lachnospiraceae_UCG-010	1	FALSE
d__Bacteria;p__Firmicutes;c__Bacilli;o__Erysipelotrichales;f__Erysipelatoclostridiaceae;g__Erysipelatoclostridiaceae	1	FALSE
d__Bacteria;p__Firmicutes;c__Clostridia;o__Lachnospirales;f__Lachnospiraceae;g__Dorea	1	FALSE
d__Bacteria;p__Actinobacteriota;c__Coriobacteriia;o__Coriobacteriales;f__Atopobiaceae;g__uncultured	1	FALSE
d__Bacteria;p__Actinobacteriota;c__Coriobacteriia;o__Coriobacteriales;f__Coriobacteriales_Incertae_Sedis;g__uncultured	1	FALSE
d__Bacteria;p__Firmicutes;c__Clostridia;o__Lachnospirales;f__Lachnospiraceae;g__Lachnospiraceae_NK4A136_group	1	FALSE
d__Bacteria;p__Firmicutes;c__Clostridia;o__Oscillospirales;f__Oscillospiraceae;g__NK4A214_group	1	FALSE
d__Bacteria;p__Firmicutes;c__Clostridia;o__Oscillospirales;f__Ruminococcaceae;g__Incertae_Sedis	1	FALSE
d__Bacteria;p__Bacteroidota;c__Bacteroidia;o__Bacteroidales;f__Prevotellaceae;g__Prevotella	1	FALSE
d__Bacteria;p__Firmicutes;c__Clostridia;o__Oscillospirales;f__[Eubacterium]_coprostanoligenes_group;g__[Eubacterium]_coprostanoligenes_group	1	FALSE
d__Bacteria;p__Bacteroidota;c__Bacteroidia;o__Bacteroidales;f__Prevotellaceae;g__Prevotellaceae_UCG-001	1	FALSE
d__Bacteria;p__Bacteroidota;c__Bacteroidia;o__Bacteroidales;f__Rikenellaceae;g__Rikenellaceae_RC9_gut_group	1	FALSE
d__Bacteria;p__Firmicutes;c__Bacilli;o__RF39;f__RF39;g__RF39	1	FALSE
d__Bacteria;p__Firmicutes;c__Clostridia;o__Peptostreptococcales-Tissierellales;f__Anaerovoracaceae;g__[Eubacterium]_nodatum_group	1	FALSE
d__Bacteria;p__Actinobacteriota;c__Coriobacteriia;o__Coriobacteriales;f__uncultured;g__uncultured	1	FALSE
d__Bacteria;p__Firmicutes;c__Clostridia;o__Oscillospirales;f__Ruminococcaceae;g__Ruminococcus	1	FALSE
d__Bacteria;p__Firmicutes;c__Clostridia;o__Clostridia_UCG-014;f__Clostridia_UCG-014;g__Clostridia_UCG-014	1	FALSE
d__Bacteria;p__Actinobacteriota;c__Coriobacteriia;o__Coriobacteriales;f__Eggerthellaceae;g__Enterorhabdus	1	FALSE
d__Bacteria;p__Bacteroidota;c__Bacteroidia;o__Bacteroidales;f__Muribaculaceae;g__Muribaculaceae	1	FALSE
d__Bacteria;p__Patescibacteria;c__Saccharimonadia;o__Saccharimonadales;f__Saccharimonadaceae;g__Candidatus_Saccharimonas	1	FALSE
d__Bacteria;p__Actinobacteriota;c__Actinobacteria;o__Actinomycetales;f__Actinomycetaceae;g__Actinomyces	1	FALSE
d__Bacteria;p__Bacteroidota;c__Bacteroidia;o__Bacteroidales;__;__	1	FALSE
d__Bacteria;p__Planctomycetota;c__Planctomycetes;o__Pirellulales;f__Pirellulaceae;g__p-1088-a5_gut_group	1	FALSE
d__Bacteria;p__Firmicutes;c__Clostridia;o__Lachnospirales;f__Lachnospiraceae;g__[Ruminococcus]_torques_group	1	FALSE
d__Bacteria;p__Firmicutes;c__Clostridia;o__Lachnospirales;f__Lachnospiraceae;g__uncultured	1	FALSE
d__Bacteria;p__Firmicutes;c__Negativicutes;o__Acidaminococcales;f__Acidaminococcaceae;g__Phascolarctobacterium	1	FALSE
d__Bacteria;p__Firmicutes;c__Clostridia;o__Monoglobales;f__Monoglobaceae;g__Monoglobus	1	FALSE
d__Bacteria;p__Bacteroidota;c__Bacteroidia;o__Bacteroidales;f__Muribaculaceae;__	1	FALSE
d__Bacteria;p__Firmicutes;c__Clostridia;o__Lachnospirales;f__Defluviitaleaceae;g__Defluviitaleaceae_UCG-011	0	FALSE
d__Bacteria;p__Firmicutes;c__Bacilli;o__Lactobacillales;f__Lactobacillaceae;g__Lactobacillus	0	FALSE
d__Bacteria;p__Firmicutes;c__Bacilli;o__Erysipelotrichales;f__Erysipelotrichaceae;g__uncultured	0	FALSE
d__Bacteria;p__Firmicutes;c__Clostridia;o__Christensenellales;f__Christensenellaceae;__	0	FALSE
d__Archaea;p__Euryarchaeota;c__Methanobacteria;o__Methanobacteriales;f__Methanobacteriaceae;g__Methanobrevibacter	0	FALSE
d__Bacteria;p__Firmicutes;c__Clostridia;o__Oscillospirales;f__Oscillospiraceae;g__UCG-005	0	FALSE
d__Bacteria;p__Firmicutes;c__Bacilli;o__Erysipelotrichales;f__Erysipelotrichaceae;g__Allobaculum	0	FALSE
d__Bacteria;p__Firmicutes;c__Clostridia;o__Lachnospirales;f__Lachnospiraceae;g__Coprococcus	0	FALSE
d__Bacteria;p__Firmicutes;c__Clostridia;o__Peptostreptococcales-Tissierellales;f__Anaerovoracaceae;g__Family_XIII_AD3011_group	0	FALSE
d__Bacteria;p__Bacteroidota;c__Bacteroidia;o__Bacteroidales;f__Prevotellaceae;g__Prevotellaceae_NK3B31_group	0	FALSE
d__Bacteria;p__Bacteroidota;c__Bacteroidia;o__Bacteroidales;f__Marinifilaceae;g__Butyricimonas	0	FALSE
d__Bacteria;p__Firmicutes;c__Clostridia;o__Oscillospirales;f__Oscillospiraceae;__	0	FALSE
d__Archaea;p__Euryarchaeota;c__Methanobacteria;o__Methanobacteriales;f__Methanobacteriaceae;g__Methanosphaera	0	FALSE
d__Bacteria;p__Spirochaetota;c__Spirochaetia;o__Spirochaetales;f__Spirochaetaceae;g__Treponema	0	FALSE
d__Bacteria;p__Proteobacteria;c__Gammaproteobacteria;o__Pasteurellales;f__Pasteurellaceae;g__Haemophilus	0	FALSE
d__Bacteria;p__Proteobacteria;c__Gammaproteobacteria;o__Burkholderiales;f__Sutterellaceae;g__Parasutterella	0	FALSE
d__Bacteria;p__Firmicutes;c__Clostridia;o__Oscillospirales;f__Oscillospiraceae;g__Papillibacter	0	FALSE
d__Bacteria;p__Firmicutes;c__Bacilli;o__Erysipelotrichales;f__Erysipelotrichaceae;g__Dubosiella	0	FALSE
d__Bacteria;p__Actinobacteriota;c__Coriobacteriia;o__Coriobacteriales;f__Atopobiaceae;__	0	FALSE
d__Bacteria;p__Elusimicrobiota;c__Elusimicrobia;o__Elusimicrobiales;f__Elusimicrobiaceae;g__Elusimicrobium	0	FALSE
d__Bacteria;p__Actinobacteriota;c__Actinobacteria;o__Bifidobacteriales;f__Bifidobacteriaceae;g__Bifidobacterium	0	FALSE
d__Bacteria;p__Bacteroidota;c__Bacteroidia;o__Bacteroidales;f__Tannerellaceae;g__Parabacteroides	0	FALSE
d__Bacteria;p__Bacteroidota;c__Bacteroidia;o__Bacteroidales;f__Bacteroidaceae;g__Bacteroides	0	FALSE
d__Bacteria;p__Firmicutes;c__Bacilli;o__Erysipelotrichales;f__Erysipelotrichaceae;g__Ileibacterium	0	FALSE
d__Bacteria;p__Verrucomicrobiota;c__Verrucomicrobiae;o__Verrucomicrobiales;f__Akkermansiaceae;g__Akkermansia	0	FALSE
d__Bacteria;p__Bacteroidota;c__Bacteroidia;o__Bacteroidales;f__Rikenellaceae;g__Alistipes	0	FALSE
d__Bacteria;p__Firmicutes;c__Clostridia;o__Lachnospirales;f__Lachnospiraceae;g__Lachnospiraceae_UCG-004	0	FALSE
d__Bacteria;p__Firmicutes;c__Clostridia;o__Lachnospirales;f__Lachnospiraceae;g__Blautia	0	FALSE
d__Bacteria;p__Firmicutes;c__Clostridia;o__Lachnospirales;f__Lachnospiraceae;g__[Eubacterium]_ruminantium_group	0	FALSE
d__Bacteria;p__Firmicutes;c__Clostridia;o__Peptostreptococcales-Tissierellales;f__Anaerovoracaceae;g__[Eubacterium]_brachy_group	0	FALSE
d__Bacteria;p__Firmicutes;c__Clostridia;o__Lachnospirales;f__Lachnospiraceae;g__Marvinbryantia	0	FALSE
d__Bacteria;p__Firmicutes;c__Clostridia;o__Oscillospirales;f__Ruminococcaceae;g__UBA1819	0	FALSE
d__Bacteria;p__Actinobacteriota;c__Coriobacteriia;o__Coriobacteriales;__;__	0	FALSE
d__Bacteria;p__Firmicutes;c__Clostridia;o__Lachnospirales;f__Lachnospiraceae;g__Frisingicoccus	0	FALSE
d__Bacteria;p__Firmicutes;c__Clostridia;o__Oscillospirales;f__Oscillospiraceae;g__Colidextribacter	0	FALSE

Supplementary File 4
ANCOM Output

Fecal 5DPT Genus Level ANCOM	W	Reject null hypothesis
d__Bacteria;p__Fusobacteriota;c__Fusobacteriia;o__Fusobacteriales;f__Fusobacteriaceae;g__Fusobacterium	28	TRUE
d__Bacteria;p__Firmicutes;c__Clostridia;o__Eubacteriales;f__Eubacteriaceae;g__uncultured	28	TRUE
d__Bacteria;p__Firmicutes;c__Bacilli;o__Erysipelotrichales;f__Erysipelotrichaceae;__	19	TRUE
d__Bacteria;p__Actinobacteriota;c__Coriobacteriia;o__Coriobacteriales;f__Coriobacteriaceae;g__Collinsella	18	TRUE
d__Bacteria;p__Bacteroidota;c__Bacteroidia;o__Bacteroidales;f__Prevotellaceae;g__Prevotellaceae_UCG-001	16	TRUE
d__Bacteria;p__Firmicutes;c__Clostridia;o__Oscillospirales;f__Ruminococcaceae;g__Incertae_Sedis	16	TRUE
d__Bacteria;p__Actinobacteriota;c__Coriobacteriia;o__Coriobacteriales;f__Eggerthellaceae;g__uncultured	16	TRUE
d__Bacteria;p__Proteobacteria;c__Gammaproteobacteria;o__Pasteurellales;f__Pasteurellaceae;g__Haemophilus	13	TRUE
d__Bacteria;p__Actinobacteriota;c__Coriobacteriia;o__Coriobacteriales;f__Eggerthellaceae;g__Paraeggerthella	12	TRUE
d__Bacteria;p__Bacteroidota;c__Bacteroidia;o__Bacteroidales;f__Prevotellaceae;g__Prevotella	12	TRUE
d__Bacteria;p__Bacteroidota;c__Bacteroidia;o__Bacteroidales;f__Bacteroidaceae;g__Bacteroides	11	TRUE
d__Bacteria;p__Firmicutes;c__Bacilli;o__Erysipelotrichales;f__Erysipelatoclostridiaceae;g__Erysipelatoclostridiaceae	11	TRUE
d__Bacteria;p__Firmicutes;c__Clostridia;o__Christensenellales;f__Christensenellaceae;g__Christensenellaceae_R-7_group	10	TRUE
d__Bacteria;p__Actinobacteriota;c__Actinobacteria;o__Actinomycetales;f__Actinomycetaceae;g__Actinomycetaceae	10	TRUE
d__Bacteria;p__Firmicutes;c__Clostridia;o__Oscillospirales;f__Ruminococcaceae;g__[Eubacterium]_siraeum_group	9	TRUE
d__Bacteria;p__Firmicutes;c__Clostridia;o__Lachnospirales;f__Lachnospiraceae;g__Marvinbryantia	9	TRUE
d__Bacteria;p__Actinobacteriota;c__Coriobacteriia;o__Coriobacteriales;f__Eggerthellaceae;g__Adlercreutzia	7	TRUE
d__Bacteria;p__Bacteroidota;c__Bacteroidia;o__Bacteroidales;f__Marinifilaceae;g__Odoribacter	7	TRUE
d__Bacteria;p__Firmicutes;c__Clostridia;o__Oscillospirales;f__Ruminococcaceae;g__UBA1819	7	TRUE
d__Bacteria;p__Bacteroidota;c__Bacteroidia;o__Bacteroidales;__;__	7	TRUE
d__Bacteria;p__Actinobacteriota;c__Coriobacteriia;o__Coriobacteriales;f__Eggerthellaceae;g__Enterorhabdus	7	TRUE
d__Bacteria;p__Firmicutes;c__Clostridia;o__Lachnospirales;f__Lachnospiraceae;g__[Eubacterium]_ruminantium_group	6	TRUE
d__Bacteria;p__Actinobacteriota;c__Coriobacteriia;o__Coriobacteriales;f__Eggerthellaceae;__	6	TRUE
d__Bacteria;p__Bacteroidota;c__Bacteroidia;o__Bacteroidales;f__Tannerellaceae;g__Parabacteroides	6	TRUE
d__Bacteria;p__Actinobacteriota;c__Actinobacteria;o__Actinomycetales;f__Actinomycetaceae;g__Actinomyces	6	TRUE
d__Bacteria;p__Actinobacteriota;c__Coriobacteriia;o__Coriobacteriales;f__Atopobiaceae;__	6	TRUE
d__Bacteria;p__Firmicutes;c__Clostridia;o__Peptostreptococcales-Tissierellales;f__Anaerovoracaceae;g__Mogibacterium	5	TRUE
d__Bacteria;p__Firmicutes;c__Bacilli;o__Erysipelotrichales;f__Erysipelatoclostridiaceae;__	5	TRUE
d__Bacteria;p__Firmicutes;c__Clostridia;o__Clostridia;f__Hungateiclostridiaceae;g__Ruminiclostridium	4	FALSE
d__Bacteria;p__Firmicutes;c__Clostridia;o__Oscillospirales;f__[Eubacterium]_coprostanoligenes_group;g__[Eubacterium]_coprostanoligenes_group	4	FALSE
d__Bacteria;p__Proteobacteria;c__Gammaproteobacteria;o__Enterobacterales;f__Enterobacteriaceae;g__Escherichia-Shigella	4	FALSE
d__Bacteria;p__Firmicutes;c__Clostridia;o__Lachnospirales;f__Lachnospiraceae;g__Lachnospiraceae_UCG-010	4	FALSE
d__Archaea;p__Euryarchaeota;c__Methanobacteria;o__Methanobacteriales;f__Methanobacteriaceae;g__Methanobrevibacter	3	FALSE
d__Bacteria;p__Firmicutes;c__Clostridia;o__Oscillospirales;f__Oscillospiraceae;g__Papillibacter	3	FALSE
d__Bacteria;p__Firmicutes;c__Bacilli;o__Erysipelotrichales;f__Erysipelotrichaceae;g__Ileibacterium	3	FALSE
d__Bacteria;p__Desulfobacterota;c__Desulfovibrionia;o__Desulfovibrionales;f__Desulfovibrionaceae;g__Bilophila	3	FALSE
d__Bacteria;p__Bacteroidota;c__Bacteroidia;o__Bacteroidales;f__Prevotellaceae;g__Prevotellaceae_UCG-003	2	FALSE
d__Bacteria;p__Desulfobacterota;c__Desulfovibrionia;o__Desulfovibrionales;f__Desulfovibrionaceae;g__Desulfovibrio	2	FALSE
d__Bacteria;p__Firmicutes;c__Clostridia;o__Lachnospirales;f__Lachnospiraceae;g__[Ruminococcus]_torques_group	2	FALSE
d__Bacteria;p__Bacteroidota;c__Bacteroidia;o__Bacteroidales;f__Muribaculaceae;__	2	FALSE
d__Bacteria;p__Firmicutes;c__Clostridia;o__Christensenellales;f__Christensenellaceae;__	2	FALSE
d__Bacteria;p__Firmicutes;c__Clostridia;o__Oscillospirales;f__Oscillospiraceae;g__Oscillibacter	2	FALSE
d__Bacteria;p__Bacteroidota;c__Bacteroidia;o__Bacteroidales;f__Marinifilaceae;g__Butyricimonas	2	FALSE
d__Bacteria;p__Firmicutes;c__Bacilli;o__Erysipelotrichales;f__Erysipelotrichaceae;g__uncultured	2	FALSE
d__Bacteria;p__Firmicutes;c__Clostridia;o__Lachnospirales;f__Lachnospiraceae;__	2	FALSE
d__Bacteria;p__Patescibacteria;c__Saccharimonadia;o__Saccharimonadales;f__Saccharimonadaceae;g__Candidatus_Saccharimonas	2	FALSE
d__Bacteria;p__Firmicutes;c__Clostridia;o__Lachnospirales;f__Lachnospiraceae;g__Lachnospiraceae_UCG-004	2	FALSE
d__Bacteria;p__Firmicutes;c__Clostridia;o__Peptostreptococcales-Tissierellales;f__Anaerovoracaceae;g__[Eubacterium]_brachy_group	1	FALSE
d__Bacteria;p__Firmicutes;c__Clostridia;o__Lachnospirales;f__Lachnospiraceae;g__Frisingicoccus	1	FALSE
d__Bacteria;p__Bacteroidota;c__Bacteroidia;o__Bacteroidales;f__Rikenellaceae;g__dgA-11_gut_group	1	FALSE
d__Bacteria;p__Actinobacteriota;c__Coriobacteriia;o__Coriobacteriales;f__Atopobiaceae;g__uncultured	1	FALSE
d__Bacteria;p__Firmicutes;c__Clostridia;o__Oscillospirales;f__Oscillospiraceae;g__uncultured	1	FALSE
d__Bacteria;p__Firmicutes;c__Clostridia;o__Lachnospirales;f__Lachnospiraceae;g__uncultured	1	FALSE
d__Bacteria;p__Bacteroidota;c__Bacteroidia;o__Bacteroidales;f__Rikenellaceae;g__Rikenellaceae_RC9_gut_group	1	FALSE
d__Bacteria;p__Firmicutes;c__Clostridia;o__Oscillospirales;f__Butyricicoccaceae;g__UCG-008	1	FALSE
d__Bacteria;p__Firmicutes;c__Clostridia;o__Lachnospirales;f__Lachnospiraceae;g__Coprococcus	1	FALSE
d__Bacteria;p__Firmicutes;c__Clostridia;o__Monoglobales;f__Monoglobaceae;g__Monoglobus	1	FALSE
d__Bacteria;p__Firmicutes;c__Clostridia;o__Oscillospirales;f__Oscillospiraceae;g__NK4A214_group	1	FALSE
d__Bacteria;p__Actinobacteriota;c__Coriobacteriia;o__Coriobacteriales;__;__	0	FALSE
d__Bacteria;p__Firmicutes;c__Clostridia;o__Lachnospirales;f__Lachnospiraceae;g__Dorea	0	FALSE
d__Bacteria;p__Firmicutes;c__Clostridia;o__Peptostreptococcales-Tissierellales;f__Anaerovoracaceae;g__[Eubacterium]_nodatum_group	0	FALSE
d__Bacteria;p__Firmicutes;c__Clostridia;o__Peptostreptococcales-Tissierellales;f__Anaerovoracaceae;g__Family_XIII_AD3011_group	0	FALSE
d__Bacteria;p__Bacteroidota;c__Bacteroidia;o__Bacteroidales;f__Prevotellaceae;g__Prevotellaceae_NK3B31_group	0	FALSE
d__Bacteria;p__Firmicutes;c__Negativicutes;o__Acidaminococcales;f__Acidaminococcaceae;g__Phascolarctobacterium	0	FALSE
d__Bacteria;p__Firmicutes;c__Bacilli;o__Erysipelotrichales;f__Erysipelotrichaceae;g__Allobaculum	0	FALSE
d__Bacteria;p__Planctomycetota;c__Planctomycetes;o__Pirellulales;f__Pirellulaceae;g__p-1088-a5_gut_group	0	FALSE
d__Bacteria;p__Firmicutes;c__Clostridia;o__Oscillospirales;f__Ruminococcaceae;g__Ruminococcus	0	FALSE
d__Bacteria;p__Bacteroidota;c__Bacteroidia;o__Bacteroidales;f__Prevotellaceae;g__Prevotellaceae_Ga6A1_group	0	FALSE
d__Bacteria;p__Actinobacteriota;c__Coriobacteriia;o__Coriobacteriales;f__uncultured;g__uncultured	0	FALSE
d__Bacteria;p__Bacteroidota;c__Bacteroidia;o__Bacteroidales;f__Rikenellaceae;g__Alistipes	0	FALSE
d__Bacteria;p__Proteobacteria;c__Gammaproteobacteria;o__Burkholderiales;f__Sutterellaceae;g__Parasutterella	0	FALSE
d__Bacteria;p__Spirochaetota;c__Spirochaetia;o__Spirochaetales;f__Spirochaetaceae;g__Treponema	0	FALSE
d__Bacteria;p__Bacteroidota;c__Bacteroidia;o__Bacteroidales;f__Muribaculaceae;g__Muribaculaceae	0	FALSE
d__Bacteria;p__Firmicutes;c__Bacilli;o__Lactobacillales;f__Lactobacillaceae;g__Lactobacillus	0	FALSE
d__Bacteria;p__Firmicutes;c__Clostridia;o__Lachnospirales;f__Lachnospiraceae;g__Blautia	0	FALSE
d__Bacteria;p__Firmicutes;c__Clostridia;o__Clostridia_UCG-014;f__Clostridia_UCG-014;g__Clostridia_UCG-014	0	FALSE
d__Bacteria;p__Firmicutes;c__Clostridia;o__Lachnospirales;f__Lachnospiraceae;g__Lachnospiraceae_UCG-001	0	FALSE
d__Bacteria;p__Firmicutes;c__Clostridia;o__Oscillospirales;f__Ruminococcaceae;__	0	FALSE
d__Bacteria;p__Firmicutes;c__Clostridia;o__Lachnospirales;f__Lachnospiraceae;g__Lachnoclostridium	0	FALSE
d__Bacteria;p__Firmicutes;c__Clostridia;o__Lachnospirales;f__Lachnospiraceae;g__Lachnospiraceae_NK4A136_group	0	FALSE
d__Bacteria;p__Elusimicrobiota;c__Elusimicrobia;o__Elusimicrobiales;f__Elusimicrobiaceae;g__Elusimicrobium	0	FALSE
d__Bacteria;p__Firmicutes;c__Clostridia;o__Lachnospirales;f__Defluviitaleaceae;g__Defluviitaleaceae_UCG-011	0	FALSE
d__Bacteria;p__Firmicutes;c__Bacilli;o__Erysipelotrichales;f__Erysipelotrichaceae;g__Dubosiella	0	FALSE
d__Bacteria;p__Firmicutes;c__Clostridia;o__Oscillospirales;f__Ruminococcaceae;g__Ruminococcaceae	0	FALSE
d__Bacteria;p__Actinobacteriota;c__Actinobacteria;o__Bifidobacteriales;f__Bifidobacteriaceae;g__Bifidobacterium	0	FALSE
d__Bacteria;p__Firmicutes;c__Bacilli;o__Erysipelotrichales;f__Erysipelotrichaceae;g__Faecalibaculum	0	FALSE
d__Archaea;p__Euryarchaeota;c__Methanobacteria;o__Methanobacteriales;f__Methanobacteriaceae;g__Methanosphaera	0	FALSE
d__Bacteria;p__Firmicutes;c__Clostridia;o__Oscillospirales;f__Oscillospiraceae;__	0	FALSE
d__Bacteria;p__Verrucomicrobiota;c__Verrucomicrobiae;o__Verrucomicrobiales;f__Akkermansiaceae;g__Akkermansia	0	FALSE
d__Bacteria;p__Bacteroidota;c__Bacteroidia;o__Bacteroidales;f__Muribaculaceae;g__Muribaculum	0	FALSE
d__Bacteria;p__Actinobacteriota;c__Coriobacteriia;o__Coriobacteriales;f__Coriobacteriales_Incertae_Sedis;g__uncultured	0	FALSE
d__Bacteria;p__Firmicutes;c__Bacilli;o__Lactobacillales;f__Streptococcaceae;g__Streptococcus	0	FALSE
d__Bacteria;p__Firmicutes;c__Clostridia;o__Oscillospirales;f__Oscillospiraceae;g__Colidextribacter	0	FALSE
d__Bacteria;p__Firmicutes;c__Bacilli;o__RF39;f__RF39;g__RF39	0	FALSE
d__Bacteria;p__Firmicutes;c__Clostridia;o__Oscillospirales;__;__	0	FALSE
d__Bacteria;p__Firmicutes;c__Clostridia;o__Oscillospirales;f__Oscillospiraceae;g__UCG-005	0	FALSE

Supplementary File 4
ANCOM Output

Fecal 7DPT Genus Level ANCOM	W	Reject null hypothesis
d__Bacteria;p__Firmicutes;c__Clostridia;o__Eubacteriales;f__Eubacteriaceae;g__uncultured	38	TRUE
d__Bacteria;p__Firmicutes;c__Bacilli;o__Erysipelotrichales;f__Erysipelotrichaceae;__	29	TRUE
d__Bacteria;p__Patescibacteria;c__Saccharimonadia;o__Saccharimonadales;f__Saccharimonadaceae;g__Candidatus_Saccharimonas	13	FALSE
d__Bacteria;p__Fusobacteriota;c__Fusobacteriia;o__Fusobacteriales;f__Fusobacteriaceae;g__Fusobacterium	11	FALSE
d__Bacteria;p__Bacteroidota;c__Bacteroidia;o__Bacteroidales;f__Bacteroidaceae;g__Bacteroides	5	FALSE
d__Bacteria;p__Actinobacteriota;c__Coriobacteriia;o__Coriobacteriales;f__Coriobacteriales_Incertae_Sedis;g__uncultured	5	FALSE
d__Archaea;p__Euryarchaeota;c__Methanobacteria;o__Methanobacteriales;f__Methanobacteriaceae;g__Methanobrevibacter	5	FALSE
d__Bacteria;p__Bacteroidota;c__Bacteroidia;o__Bacteroidales;f__Tannerellaceae;g__Parabacteroides	5	FALSE
d__Bacteria;p__Firmicutes;c__Clostridia;o__Oscillospirales;f__Ruminococcaceae;g__UBA1819	4	FALSE
d__Bacteria;p__Actinobacteriota;c__Coriobacteriia;o__Coriobacteriales;f__Eggerthellaceae;g__Paraeggerthella	3	FALSE
d__Bacteria;p__Actinobacteriota;c__Coriobacteriia;o__Coriobacteriales;f__Coriobacteriaceae;g__Collinsella	3	FALSE
d__Bacteria;p__Firmicutes;c__Bacilli;o__Erysipelotrichales;f__Erysipelotrichaceae;g__Ileibacterium	3	FALSE
d__Bacteria;p__Firmicutes;c__Clostridia;o__Lachnospirales;f__Lachnospiraceae;g__Coprococcus	3	FALSE
d__Bacteria;p__Actinobacteriota;c__Coriobacteriia;o__Coriobacteriales;f__uncultured;g__uncultured	3	FALSE
d__Bacteria;p__Proteobacteria;c__Gammaproteobacteria;o__Enterobacterales;f__Enterobacteriaceae;g__Escherichia-Shigella	3	FALSE
d__Bacteria;p__Actinobacteriota;c__Actinobacteria;o__Actinomycetales;f__Actinomycetaceae;g__Actinomycetaceae	3	FALSE
d__Bacteria;p__Desulfobacterota;c__Desulfovibrionia;o__Desulfovibrionales;f__Desulfovibrionaceae;g__Desulfovibrio	3	FALSE
d__Bacteria;p__Firmicutes;c__Clostridia;o__Lachnospirales;f__Lachnospiraceae;g__Lachnospiraceae_UCG-001	2	FALSE
d__Bacteria;p__Firmicutes;c__Clostridia;o__Oscillospirales;f__Butyricicoccaceae;g__UCG-008	2	FALSE
d__Bacteria;p__Firmicutes;c__Clostridia;o__Lachnospirales;f__Lachnospiraceae;g__Lachnoclostridium	2	FALSE
d__Bacteria;p__Firmicutes;c__Clostridia;o__Lachnospirales;f__Lachnospiraceae;g__Lachnospiraceae_UCG-004	2	FALSE
d__Bacteria;p__Firmicutes;c__Clostridia;o__Oscillospirales;__;__	2	FALSE
d__Bacteria;p__Firmicutes;c__Bacilli;o__Erysipelotrichales;f__Erysipelotrichaceae;g__Faecalibaculum	2	FALSE
d__Bacteria;p__Firmicutes;c__Clostridia;o__Oscillospirales;f__Oscillospiraceae;g__Papillibacter	2	FALSE
d__Bacteria;p__Firmicutes;c__Clostridia;o__Lachnospirales;f__Lachnospiraceae;g__Dorea	2	FALSE
d__Bacteria;p__Firmicutes;c__Clostridia;o__Lachnospirales;f__Lachnospiraceae;g__Marvinbryantia	2	FALSE
d__Bacteria;p__Actinobacteriota;c__Actinobacteria;o__Actinomycetales;f__Actinomycetaceae;g__Actinomyces	2	FALSE
d__Bacteria;p__Actinobacteriota;c__Coriobacteriia;o__Coriobacteriales;f__Atopobiaceae;g__uncultured	2	FALSE
d__Bacteria;p__Firmicutes;c__Bacilli;o__Erysipelotrichales;f__Erysipelatoclostridiaceae;g__Erysipelatoclostridiaceae	2	FALSE
d__Bacteria;p__Firmicutes;c__Clostridia;o__Lachnospirales;f__Lachnospiraceae;__	2	FALSE
d__Bacteria;p__Bacteroidota;c__Bacteroidia;o__Bacteroidales;f__Rikenellaceae;g__dgA-11_gut_group	2	FALSE
d__Bacteria;p__Firmicutes;c__Clostridia;o__Lachnospirales;f__Lachnospiraceae;g__Blautia	1	FALSE
d__Bacteria;p__Firmicutes;c__Clostridia;o__Oscillospirales;f__Ruminococcaceae;g__[Eubacterium]_siraeum_group	1	FALSE
d__Bacteria;p__Spirochaetota;c__Spirochaetia;o__Spirochaetales;f__Spirochaetaceae;g__Treponema	1	FALSE
d__Bacteria;p__Actinobacteriota;c__Coriobacteriia;o__Coriobacteriales;f__Eggerthellaceae;__	1	FALSE
d__Bacteria;p__Firmicutes;c__Clostridia;o__Clostridia;f__Hungateiclostridiaceae;g__Ruminiclostridium	1	FALSE
d__Bacteria;p__Firmicutes;c__Bacilli;o__Erysipelotrichales;f__Erysipelotrichaceae;g__Dubosiella	1	FALSE
d__Bacteria;p__Firmicutes;c__Clostridia;o__Lachnospirales;f__Defluviitaleaceae;g__Defluviitaleaceae_UCG-011	1	FALSE
d__Bacteria;p__Firmicutes;c__Clostridia;o__Oscillospirales;f__Ruminococcaceae;g__Ruminococcaceae	1	FALSE
d__Bacteria;p__Firmicutes;c__Clostridia;o__Lachnospirales;f__Lachnospiraceae;g__Lachnospiraceae_UCG-010	1	FALSE
d__Bacteria;p__Firmicutes;c__Clostridia;o__Oscillospirales;f__Oscillospiraceae;g__Oscillibacter	1	FALSE
d__Bacteria;p__Bacteroidota;c__Bacteroidia;o__Bacteroidales;f__Muribaculaceae;g__Muribaculum	1	FALSE
d__Bacteria;p__Firmicutes;c__Clostridia;o__Christensenellales;f__Christensenellaceae;__	1	FALSE
d__Bacteria;p__Proteobacteria;c__Gammaproteobacteria;o__Burkholderiales;f__Sutterellaceae;g__Parasutterella	1	FALSE
d__Bacteria;p__Bacteroidota;c__Bacteroidia;o__Bacteroidales;f__Marinifilaceae;g__Odoribacter	1	FALSE
d__Bacteria;p__Firmicutes;c__Clostridia;o__Peptostreptococcales-Tissierellales;f__Anaerovoracaceae;g__[Eubacterium]_brachy_group	1	FALSE
d__Bacteria;p__Firmicutes;c__Clostridia;o__Oscillospirales;f__Oscillospiraceae;g__uncultured	1	FALSE
d__Bacteria;p__Firmicutes;c__Clostridia;o__Lachnospirales;f__Lachnospiraceae;g__Lachnospiraceae_NK4A136_group	1	FALSE
d__Bacteria;p__Planctomycetota;c__Planctomycetes;o__Pirellulales;f__Pirellulaceae;g__p-1088-a5_gut_group	1	FALSE
d__Bacteria;p__Firmicutes;c__Clostridia;o__Lachnospirales;f__Lachnospiraceae;g__uncultured	1	FALSE
d__Bacteria;p__Firmicutes;c__Clostridia;o__Oscillospirales;f__[Eubacterium]_coprostanoligenes_group;g__[Eubacterium]_coprostanoligenes_group	1	FALSE
d__Bacteria;p__Firmicutes;c__Clostridia;o__Oscillospirales;f__Ruminococcaceae;g__Ruminococcus	1	FALSE
d__Bacteria;p__Bacteroidota;c__Bacteroidia;o__Bacteroidales;f__Prevotellaceae;g__Prevotellaceae_Ga6A1_group	1	FALSE
d__Bacteria;p__Bacteroidota;c__Bacteroidia;o__Bacteroidales;f__Rikenellaceae;g__Alistipes	1	FALSE
d__Bacteria;p__Firmicutes;c__Clostridia;o__Peptostreptococcales-Tissierellales;f__Anaerovoracaceae;g__[Eubacterium]_nodatum_group	1	FALSE
d__Bacteria;p__Actinobacteriota;c__Coriobacteriia;o__Coriobacteriales;__;__	1	FALSE
d__Bacteria;p__Firmicutes;c__Bacilli;o__Erysipelotrichales;f__Erysipelotrichaceae;g__uncultured	1	FALSE
d__Bacteria;p__Firmicutes;c__Clostridia;o__Oscillospirales;f__Ruminococcaceae;g__Incertae_Sedis	1	FALSE
d__Bacteria;p__Bacteroidota;c__Bacteroidia;o__Bacteroidales;__;__	1	FALSE
d__Bacteria;p__Firmicutes;c__Clostridia;o__Clostridia_UCG-014;f__Clostridia_UCG-014;g__Clostridia_UCG-014	1	FALSE
d__Bacteria;p__Proteobacteria;c__Gammaproteobacteria;o__Pasteurellales;f__Pasteurellaceae;g__Haemophilus	1	FALSE
d__Bacteria;p__Firmicutes;c__Bacilli;o__Lactobacillales;f__Streptococcaceae;g__Streptococcus	1	FALSE
d__Bacteria;p__Firmicutes;c__Bacilli;o__Erysipelotrichales;f__Erysipelotrichaceae;g__Allobaculum	0	FALSE
d__Bacteria;p__Firmicutes;c__Clostridia;o__Christensenellales;f__Christensenellaceae;g__Christensenellaceae_R-7_group	0	FALSE
d__Bacteria;p__Bacteroidota;c__Bacteroidia;o__Bacteroidales;f__Prevotellaceae;g__Prevotellaceae_UCG-001	0	FALSE
d__Bacteria;p__Bacteroidota;c__Bacteroidia;o__Bacteroidales;f__Rikenellaceae;g__Rikenellaceae_RC9_gut_group	0	FALSE
d__Bacteria;p__Firmicutes;c__Clostridia;o__Oscillospirales;f__Oscillospiraceae;g__NK4A214_group	0	FALSE
d__Bacteria;p__Bacteroidota;c__Bacteroidia;o__Bacteroidales;f__Muribaculaceae;g__Muribaculaceae	0	FALSE
d__Bacteria;p__Firmicutes;c__Clostridia;o__Peptostreptococcales-Tissierellales;f__Anaerovoracaceae;g__Family_XIII_AD3011_group	0	FALSE
d__Bacteria;p__Actinobacteriota;c__Coriobacteriia;o__Coriobacteriales;f__Eggerthellaceae;g__Enterorhabdus	0	FALSE
d__Bacteria;p__Bacteroidota;c__Bacteroidia;o__Bacteroidales;f__Marinifilaceae;g__Butyricimonas	0	FALSE
d__Bacteria;p__Firmicutes;c__Clostridia;o__Peptostreptococcales-Tissierellales;f__Anaerovoracaceae;g__Mogibacterium	0	FALSE
d__Bacteria;p__Firmicutes;c__Clostridia;o__Lachnospirales;f__Lachnospiraceae;g__Frisingicoccus	0	FALSE
d__Bacteria;p__Bacteroidota;c__Bacteroidia;o__Bacteroidales;f__Prevotellaceae;g__Prevotellaceae_NK3B31_group	0	FALSE
d__Bacteria;p__Actinobacteriota;c__Coriobacteriia;o__Coriobacteriales;f__Atopobiaceae;__	0	FALSE
d__Bacteria;p__Actinobacteriota;c__Coriobacteriia;o__Coriobacteriales;f__Eggerthellaceae;g__uncultured	0	FALSE
d__Bacteria;p__Actinobacteriota;c__Coriobacteriia;o__Coriobacteriales;f__Eggerthellaceae;g__Adlercreutzia	0	FALSE
d__Bacteria;p__Firmicutes;c__Bacilli;o__Erysipelotrichales;f__Erysipelatoclostridiaceae;__	0	FALSE
d__Bacteria;p__Firmicutes;c__Clostridia;o__Oscillospirales;f__Oscillospiraceae;__	0	FALSE
d__Bacteria;p__Bacteroidota;c__Bacteroidia;o__Bacteroidales;f__Prevotellaceae;g__Prevotellaceae_UCG-003	0	FALSE
d__Bacteria;p__Firmicutes;c__Clostridia;o__Oscillospirales;f__Oscillospiraceae;g__Colidextribacter	0	FALSE
d__Bacteria;p__Firmicutes;c__Clostridia;o__Oscillospirales;f__Ruminococcaceae;__	0	FALSE
d__Bacteria;p__Firmicutes;c__Bacilli;o__RF39;f__RF39;g__RF39	0	FALSE
d__Archaea;p__Euryarchaeota;c__Methanobacteria;o__Methanobacteriales;f__Methanobacteriaceae;g__Methanosphaera	0	FALSE
d__Bacteria;p__Elusimicrobiota;c__Elusimicrobia;o__Elusimicrobiales;f__Elusimicrobiaceae;g__Elusimicrobium	0	FALSE
d__Bacteria;p__Firmicutes;c__Negativicutes;o__Acidaminococcales;f__Acidaminococcaceae;g__Phascolarctobacterium	0	FALSE
d__Bacteria;p__Actinobacteriota;c__Actinobacteria;o__Bifidobacteriales;f__Bifidobacteriaceae;g__Bifidobacterium	0	FALSE
d__Bacteria;p__Bacteroidota;c__Bacteroidia;o__Bacteroidales;f__Prevotellaceae;g__Prevotella	0	FALSE
d__Bacteria;p__Bacteroidota;c__Bacteroidia;o__Bacteroidales;f__Muribaculaceae;__	0	FALSE
d__Bacteria;p__Firmicutes;c__Bacilli;o__Lactobacillales;f__Lactobacillaceae;g__Lactobacillus	0	FALSE
d__Bacteria;p__Desulfobacterota;c__Desulfovibrionia;o__Desulfovibrionales;f__Desulfovibrionaceae;g__Bilophila	0	FALSE
d__Bacteria;p__Verrucomicrobiota;c__Verrucomicrobiae;o__Verrucomicrobiales;f__Akkermansiaceae;g__Akkermansia	0	FALSE
d__Bacteria;p__Firmicutes;c__Clostridia;o__Lachnospirales;f__Lachnospiraceae;g__Lachnospira	0	FALSE
d__Bacteria;p__Firmicutes;c__Clostridia;o__Monoglobales;f__Monoglobaceae;g__Monoglobus	0	FALSE
d__Bacteria;p__Firmicutes;c__Clostridia;o__Lachnospirales;f__Lachnospiraceae;g__[Eubacterium]_ruminantium_group	0	FALSE
d__Bacteria;p__Firmicutes;c__Clostridia;o__Oscillospirales;f__Oscillospiraceae;g__UCG-005	0	FALSE
d__Bacteria;p__Firmicutes;c__Clostridia;o__Lachnospirales;f__Lachnospiraceae;g__[Ruminococcus]_torques_group	0	FALSE

Supplementary File 4
ANCOM Output

Fecal Baseline Genus Level ANCOM
No significant differentially abundant taxa detected

Fecal 9DPT Genus Level ANCOM
No significant differentially abundant taxa detected

Supplementary File 4
ANCOM Output

NPL 2DPT Genus Level ANCOM	W	Reject null hypothesis
d__Bacteria;p__Bacteroidota;c__Bacteroidia;o__Bacteroidales;f__Bacteroidaceae;g__Bacteroides	12	TRUE
d__Bacteria;p__Actinobacteriota;c__Actinobacteria;o__Actinomycetales;f__Actinomycetaceae;g__Mobiluncus	6	TRUE
d__Bacteria;p__Actinobacteriota;c__Actinobacteria;o__Corynebacteriales;f__Corynebacteriaceae;g__Corynebacterium	4	TRUE
d__Bacteria;p__Firmicutes;c__Bacilli;o__Lactobacillales;f__Streptococcaceae;g__Streptococcus	4	TRUE
d__Archaea;p__Euryarchaeota;c__Methanobacteria;o__Methanobacteriales;f__Methanobacteriaceae;g__Methanobrevibacter	4	TRUE
d__Bacteria;p__Proteobacteria;c__Gammaproteobacteria;o__Enterobacterales;f__Enterobacteriaceae;g__Escherichia-Shigella	3	FALSE
d__Bacteria;p__Campilobacterota;c__Campylobacteria;o__Campylobacterales;f__Campylobacteraceae;g__Campylobacter	3	FALSE
d__Bacteria;p__Proteobacteria;c__Gammaproteobacteria;o__Xanthomonadales;f__Xanthomonadaceae;g__Xanthomonas	3	FALSE
d__Bacteria;p__Bacteroidota;c__Bacteroidia;o__Chitinophagales;f__Chitinophagaceae;__	3	FALSE
d__Bacteria;p__Bacteroidota;c__Bacteroidia;o__Bacteroidales;f__Prevotellaceae;__	1	FALSE
d__Bacteria;p__Firmicutes;c__Bacilli;o__Erysipelotrichales;f__Erysipelotrichaceae;__	1	FALSE
d__Bacteria;p__Bacteroidota;c__Bacteroidia;o__Bacteroidales;f__Prevotellaceae;g__Prevotellaceae_UCG-001	1	FALSE
d__Bacteria;p__Bacteroidota;c__Bacteroidia;o__Bacteroidales;__;__	1	FALSE
d__Bacteria;p__Bacteroidota;c__Bacteroidia;o__Bacteroidales;f__Muribaculaceae;g__Muribaculaceae	1	FALSE
d__Bacteria;p__Actinobacteriota;c__Coriobacteriia;o__Coriobacteriales;f__Atopobiaceae;g__Olsenella	1	FALSE
d__Bacteria;p__Fusobacteriota;c__Fusobacteriia;o__Fusobacteriales;f__Fusobacteriaceae;g__Fusobacterium	0	FALSE
d__Bacteria;p__Bacteroidota;c__Bacteroidia;o__Sphingobacteriales;f__Sphingobacteriaceae;__	0	FALSE
d__Bacteria;p__Actinobacteriota;c__Actinobacteria;o__Micrococcales;__;__	0	FALSE
d__Bacteria;p__Firmicutes;c__Clostridia;o__Lachnospirales;f__Lachnospiraceae;__	0	FALSE
d__Bacteria;p__Firmicutes;c__Clostridia;o__Eubacteriales;f__Eubacteriaceae;g__uncultured	0	FALSE
d__Bacteria;p__Firmicutes;c__Bacilli;o__Mycoplasmatales;f__Mycoplasmataceae;g__Mycoplasma	0	FALSE
d__Bacteria;p__Proteobacteria;c__Gammaproteobacteria;o__Pasteurellales;f__Pasteurellaceae;g__Actinobacillus	0	FALSE
d__Bacteria;p__Fusobacteriota;c__Fusobacteriia;o__Fusobacteriales;f__Leptotrichiaceae;__	0	FALSE
d__Bacteria;p__Firmicutes;c__Clostridia;o__Peptostreptococcales-Tissierellales;f__Peptostreptococcales-Tissierellales;g__Peptoniphilus	0	FALSE
d__Bacteria;p__Patescibacteria;c__Saccharimonadia;o__Saccharimonadales;f__Saccharimonadales;g__Saccharimonadales	0	FALSE
d__Bacteria;p__Actinobacteriota;c__Actinobacteria;o__Actinomycetales;f__Actinomycetaceae;__	0	FALSE
d__Bacteria;p__Firmicutes;c__Bacilli;o__Lactobacillales;f__Aerococcaceae;g__Aerococcus	0	FALSE
d__Bacteria;p__Proteobacteria;c__Gammaproteobacteria;o__Burkholderiales;__;__	0	FALSE
d__Bacteria;p__Actinobacteriota;c__Actinobacteria;o__Bifidobacteriales;f__Bifidobacteriaceae;g__Bifidobacterium	0	FALSE
d__Bacteria;p__Proteobacteria;c__Gammaproteobacteria;o__Enterobacterales;f__Enterobacteriaceae;__	0	FALSE
d__Bacteria;p__Firmicutes;c__Bacilli;o__Staphylococcales;f__Staphylococcaceae;g__Staphylococcus	0	FALSE
d__Bacteria;p__Firmicutes;c__Bacilli;o__Erysipelotrichales;f__Erysipelotrichaceae;g__Ileibacterium	0	FALSE

NPL 5DPT Genus Level ANCOM	W	Reject null hypothesis
d__Bacteria;p__Bacteroidota;c__Bacteroidia;o__Bacteroidales;f__Bacteroidaceae;g__Bacteroides	4	TRUE
d__Bacteria;p__Actinobacteriota;c__Actinobacteria;o__Actinomycetales;f__Actinomycetaceae;g__Mobiluncus	1	TRUE
d__Bacteria;p__Firmicutes;c__Clostridia;o__Lachnospirales;f__Lachnospiraceae;__	1	TRUE
d__Bacteria;p__Actinobacteriota;c__Coriobacteriia;o__Coriobacteriales;f__Atopobiaceae;g__Olsenella	1	TRUE
d__Bacteria;p__Proteobacteria;c__Gammaproteobacteria;o__Pasteurellales;f__Pasteurellaceae;g__Actinobacillus	1	TRUE
d__Bacteria;p__Proteobacteria;c__Gammaproteobacteria;o__Burkholderiales;__;__	0	TRUE
d__Bacteria;p__Actinobacteriota;c__Actinobacteria;o__Micrococcales;__;__	0	TRUE
d__Bacteria;p__Proteobacteria;c__Gammaproteobacteria;o__Xanthomonadales;f__Xanthomonadaceae;g__Xanthomonas	0	TRUE
d__Bacteria;p__Firmicutes;c__Clostridia;o__Eubacteriales;f__Eubacteriaceae;g__uncultured	0	TRUE
d__Bacteria;p__Actinobacteriota;c__Actinobacteria;o__Corynebacteriales;f__Corynebacteriaceae;g__Corynebacterium	0	TRUE
d__Bacteria;p__Firmicutes;c__Bacilli;o__Mycoplasmatales;f__Mycoplasmataceae;g__Mycoplasma	0	TRUE
d__Bacteria;p__Fusobacteriota;c__Fusobacteriia;o__Fusobacteriales;f__Leptotrichiaceae;__	0	TRUE
d__Bacteria;p__Bacteroidota;c__Bacteroidia;o__Bacteroidales;f__Prevotellaceae;__	0	TRUE
d__Bacteria;p__Patescibacteria;c__Saccharimonadia;o__Saccharimonadales;f__Saccharimonadales;g__Saccharimonadales	0	TRUE
d__Bacteria;p__Firmicutes;c__Bacilli;o__Erysipelotrichales;f__Erysipelotrichaceae;__	0	TRUE
d__Bacteria;p__Bacteroidota;c__Bacteroidia;o__Chitinophagales;f__Chitinophagaceae;__	0	TRUE
d__Bacteria;p__Firmicutes;c__Bacilli;o__Erysipelotrichales;f__Erysipelotrichaceae;g__Ileibacterium	0	TRUE
d__Bacteria;p__Actinobacteriota;c__Actinobacteria;o__Bifidobacteriales;f__Bifidobacteriaceae;g__Bifidobacterium	0	TRUE
d__Bacteria;p__Proteobacteria;c__Gammaproteobacteria;o__Enterobacterales;f__Enterobacteriaceae;__	0	TRUE
d__Bacteria;p__Firmicutes;c__Bacilli;o__Lactobacillales;f__Streptococcaceae;g__Streptococcus	0	TRUE
d__Bacteria;p__Bacteroidota;c__Bacteroidia;o__Sphingobacteriales;f__Sphingobacteriaceae;__	0	TRUE
d__Archaea;p__Euryarchaeota;c__Methanobacteria;o__Methanobacteriales;f__Methanobacteriaceae;g__Methanobrevibacter	0	TRUE
d__Bacteria;p__Proteobacteria;c__Gammaproteobacteria;o__Enterobacterales;f__Enterobacteriaceae;g__Escherichia-Shigella	0	TRUE
d__Bacteria;p__Fusobacteriota;c__Fusobacteriia;o__Fusobacteriales;f__Fusobacteriaceae;g__Fusobacterium	0	TRUE
d__Bacteria;p__Firmicutes;c__Bacilli;o__Staphylococcales;f__Staphylococcaceae;g__Staphylococcus	0	TRUE
d__Bacteria;p__Firmicutes;c__Bacilli;o__Lactobacillales;f__Aerococcaceae;g__Aerococcus	0	TRUE
d__Bacteria;p__Actinobacteriota;c__Actinobacteria;o__Actinomycetales;f__Actinomycetaceae;__	0	TRUE
d__Bacteria;p__Firmicutes;c__Clostridia;o__Peptostreptococcales-Tissierellales;f__Peptostreptococcales-Tissierellales;g__Peptoniphilus	0	TRUE
d__Bacteria;p__Bacteroidota;c__Bacteroidia;o__Bacteroidales;f__Muribaculaceae;g__Muribaculaceae	0	TRUE
d__Bacteria;p__Bacteroidota;c__Bacteroidia;o__Bacteroidales;__;__	0	TRUE
d__Bacteria;p__Campilobacterota;c__Campylobacteria;o__Campylobacterales;f__Campylobacteraceae;g__Campylobacter	0	TRUE
d__Bacteria;p__Bacteroidota;c__Bacteroidia;o__Bacteroidales;f__Prevotellaceae;g__Prevotellaceae_UCG-001	0	TRUE

NPL Baseline Genus Level ANCOM

No significant differentially abundant taxa detected

NPL 7DPT Genus Level ANCOM

No significant differentially abundant taxa detected

NPL 9DPT Genus Level ANCOM

No significant differentially abundant taxa detected

Supplementary File 5
Relative Abundance Data

FECAL SAMPLE TAXA
0.01% in 10% Conditional Filtered Taxa

Feature ID	Taxon ID
08022a5e87ba92d0d71e798e91f087d2	d__Bacteria; p__Firmicutes; c__Clostridia; o__Oscillospirales; f__Oscillospiraceae; g__uncultured; s__uncultured_Clostridiales
890158b78073004135337c2a166b3ab6	d__Bacteria; p__Firmicutes; c__Bacilli; o__Erysipelotrichales; f__Erysipelotrichaceae
7a64c90b1106920a3ce708cb0597cf0b	d__Bacteria; p__Firmicutes; c__Clostridia; o__Christensenellales; f__Christensenellaceae; g__Christensenellaceae_R-7_group
86360e802ea776af481f2936f04cb149	d__Bacteria; p__Proteobacteria; c__Gammaproteobacteria; o__Pasteurellales; f__Pasteurellaceae; g__Haemophilus; s__Pasteurella_sp.
b85297971270c8f1627f88de1d1d5868	d__Bacteria; p__Bacteroidota; c__Bacteroidia; o__Bacteroidales
4bbc54c3e96e98d2ba46072f9ce02b47	d__Bacteria; p__Actinobacteriota; c__Actinobacteria; o__Actinomycetales; f__Actinomycetaceae; g__Actinomyces; s__Actinomyces_denticolens
f5dd2b0e74207739fe9ec161e09e9df2	d__Bacteria; p__Patescibacteria; c__Saccharimonadia; o__Saccharimonadales; f__Saccharimonadaceae; g__Candidatus_Saccharimonas; s__uncultured_bacterium
c4cbf1d5c4ed34754e238a21c4c2b04e	d__Bacteria; p__Bacteroidota; c__Bacteroidia; o__Bacteroidales; f__Muribaculaceae; g__Muribaculaceae
b2071611249895c0c8b89fef2fca61df	d__Bacteria; p__Bacteroidota; c__Bacteroidia; o__Bacteroidales; f__Muribaculaceae; g__Muribaculaceae; s__uncultured_bacterium
eebd8b2415d8416d2cf7f04987f46d95	d__Bacteria; p__Firmicutes; c__Clostridia; o__Lachnospirales; f__Lachnospiraceae
2222c9aaa6f4afa1ba645f166702f1ee	d__Bacteria; p__Actinobacteriota; c__Coriobacteriia; o__Coriobacteriales; f__Eggerthellaceae; g__Enterorhabdus; s__uncultured_bacterium
ea47282093f8126c02bc874bcbf4253b	d__Bacteria; p__Bacteroidota; c__Bacteroidia; o__Bacteroidales; f__Muribaculaceae; g__Muribaculaceae; s__uncultured_Bacteroidales
a6eca05784ff59a0dd025f2a1a818852	d__Bacteria; p__Firmicutes; c__Clostridia; o__Clostridia_UCG-014; f__Clostridia_UCG-014; g__Clostridia_UCG-014
18715a9e3d4b849fc5872d8b5385b291	d__Bacteria; p__Actinobacteriota; c__Coriobacteriia; o__Coriobacteriales; f__uncultured; g__uncultured; s__uncultured_bacterium
3be04d1ced41c96732de51c984a76164	d__Bacteria; p__Firmicutes; c__Bacilli; o__Erysipelotrichales; f__Erysipelotrichaceae; g__Allobaculum; s__uncultured_bacterium
e0eec2e30f1c59319502589c7fec7ff9	d__Bacteria; p__Patescibacteria; c__Saccharimonadia; o__Saccharimonadales; f__Saccharimonadaceae; g__Candidatus_Saccharimonas; s__uncultured_bacterium
ac5088f13bccd9135b505c1756acdf4a	d__Bacteria; p__Firmicutes; c__Clostridia; o__Oscillospirales; f__Ruminococcaceae; g__Incertae_Sedis; s__uncultured_bacterium
641c026f48187c73adb6a88588484423	d__Bacteria; p__Firmicutes; c__Clostridia; o__Oscillospirales; f__Ruminococcaceae; g__Incertae_Sedis
43009714c70cc15e6b5d50ff39bebef3	d__Bacteria; p__Firmicutes; c__Bacilli; o__Erysipelotrichales; f__Erysipelotrichaceae; g__uncultured; s__uncultured_bacterium
77f5e7677187129e40d80e99ff25270a	d__Bacteria; p__Bacteroidota; c__Bacteroidia; o__Bacteroidales; f__Marinifilaceae; g__Butyricimonas
7f865f0e617e7bdfc38668701998459b	d__Bacteria; p__Actinobacteriota; c__Coriobacteriia; o__Coriobacteriales
7f2daab36b8a637038fbc90c2f7a61d9	d__Bacteria; p__Firmicutes; c__Bacilli; o__Erysipelotrichales; f__Erysipelotrichaceae
ee5d9feced0b968e55a7605377c5b631	d__Bacteria; p__Firmicutes; c__Clostridia; o__Oscillospirales; f__Ruminococcaceae; g__Incertae_Sedis; s__uncultured_bacterium
70cdd93c4e2a368b3b0f71588870c912	d__Bacteria; p__Firmicutes; c__Clostridia; o__Lachnospirales; f__Lachnospiraceae; g__Marvinbryantia
6cadccbb4fb02b309ba035f0467133b2	d__Bacteria; p__Firmicutes; c__Clostridia; o__Peptostreptococcales-Tissierellales; f__Anaerovoracaceae; g__[Eubacterium]_nodatum_group
29f4fdb3b7921719f48294f768211822	d__Bacteria; p__Firmicutes; c__Clostridia; o__Oscillospirales; f__Oscillospiraceae; g__NK4A214_group; s__uncultured_Clostridiaceae
ee80d6aadbf689cf5e41845da9101897	d__Bacteria; p__Bacteroidota; c__Bacteroidia; o__Bacteroidales; f__Rikenellaceae; g__Rikenellaceae_RC9_gut_group; s__uncultured_bacterium
3e6824a0b203cc130113cbe6a1ce8ba9	d__Bacteria; p__Firmicutes; c__Clostridia; o__Christensenellales; f__Christensenellaceae; g__Christensenellaceae_R-7_group
e2fc6bd05b57f8fb9b45da04e47dc638	d__Bacteria; p__Firmicutes; c__Clostridia; o__Lachnospirales; f__Lachnospiraceae
ae188c8a52c294a0404dfd9b7240e217	d__Bacteria; p__Bacteroidota; c__Bacteroidia; o__Bacteroidales; f__Prevotellaceae; g__Prevotellaceae_UCG-001; s__uncultured_bacterium
ca80ba88693081015c4e21cfbdbcda61	d__Bacteria; p__Firmicutes; c__Clostridia; o__Oscillospirales; f__[Eubacterium]_coprostanoligenes_group; g__[Eubacterium]_coprostanoligenes_group; s__uncultured_rumen
86ee96e2c497a070c7f5a7e9fcda69d5	d__Bacteria; p__Bacteroidota; c__Bacteroidia; o__Bacteroidales; f__Prevotellaceae; g__Prevotella; s__uncultured_bacterium
3a518ebe9b2c89142064a07d5ea80715	d__Bacteria; p__Firmicutes; c__Clostridia; o__Oscillospirales; f__Oscillospiraceae; g__uncultured
c281682eccc9c19ec742599638ee105b	d__Bacteria; p__Planctomycetota; c__Planctomycetes; o__Pirellulales; f__Pirellulaceae; g__p-1088-a5_gut_group; s__uncultured_bacterium
db181b48d3fee4a5eabb7ef3597f6615	d__Bacteria; p__Firmicutes; c__Clostridia; o__Oscillospirales; f__Oscillospiraceae; g__NK4A214_group; s__uncultured_rumen
19b34e038b6e353e9c454b14fcc4c4e0	d__Bacteria; p__Firmicutes; c__Clostridia; o__Lachnospirales; f__Lachnospiraceae
180e3ac0ac4db4cafdbc262d2c17b54f	d__Bacteria; p__Bacteroidota; c__Bacteroidia; o__Bacteroidales; f__Muribaculaceae; g__Muribaculaceae; s__uncultured_bacterium
994a67bfc4f6e1841b6167e9abf944fc	d__Bacteria; p__Firmicutes; c__Clostridia; o__Oscillospirales; f__Ruminococcaceae; g__Ruminococcus
7a18ad931d9b663859f8f89ea118c507	d__Bacteria; p__Firmicutes; c__Clostridia; o__Christensenellales; f__Christensenellaceae; g__Christensenellaceae_R-7_group; s__uncultured_bacterium
b69a4f1338973931a5e6615874940c43	d__Bacteria; p__Bacteroidota; c__Bacteroidia; o__Bacteroidales; f__Prevotellaceae; g__Prevotellaceae_Ga6A1_group; s__uncultured_bacterium
7d74488415542b78d10b5973fb2635e4	d__Bacteria; p__Bacteroidota; c__Bacteroidia; o__Bacteroidales; f__Muribaculaceae; g__Muribaculaceae; s__uncultured_bacterium
52762f7f6646d64fd58ad4fec3eb67b2	d__Bacteria; p__Bacteroidota; c__Bacteroidia; o__Bacteroidales; f__Bacteroidaceae; g__Bacteroides; s__Bacteroides_sartorii
7725b5ac83e30e5ccb9fef7444bd60f0	d__Bacteria; p__Bacteroidota; c__Bacteroidia; o__Bacteroidales; f__Muribaculaceae; g__Muribaculaceae; s__uncultured_bacterium
3eea9647857ca0d7ce4733bcef222047	d__Bacteria; p__Firmicutes; c__Bacilli; o__Erysipelotrichales; f__Erysipelotrichaceae; g__Ileibacterium; s__uncultured_bacterium
35e302d1855af23b0f138db415125520	d__Bacteria; p__Firmicutes; c__Clostridia; o__Monoglobales; f__Monoglobaceae; g__Monoglobus; s__uncultured_bacterium
3999c7a7b47f931f424d6982b0dc4e86	d__Bacteria; p__Bacteroidota; c__Bacteroidia; o__Bacteroidales; f__Muribaculaceae; g__Muribaculaceae; s__uncultured_bacterium
ed6bfe367d7a88ef3554ee9d2e322e91	d__Bacteria; p__Bacteroidota; c__Bacteroidia; o__Bacteroidales; f__Rikenellaceae; g__Alistipes; s__Alistipes_finegoldii
ff726a2cf334158a087cc67d1e529bd5	d__Bacteria; p__Firmicutes; c__Clostridia; o__Lachnospirales; f__Lachnospiraceae; g__Coprococcus; s__Clostridium_sp.
e9dda0d3491df30006189be0e3552d83	d__Bacteria; p__Firmicutes; c__Bacilli; o__Erysipelotrichales; f__Erysipelotrichaceae; g__Ileibacterium; s__uncultured_bacterium
8d4980b75e1189e61fcd16b833130e9f	d__Bacteria; p__Bacteroidota; c__Bacteroidia; o__Bacteroidales; f__Bacteroidaceae; g__Bacteroides
d9bc3adeb66bac15d0c11ea64c107222	d__Bacteria; p__Firmicutes; c__Bacilli; o__Erysipelotrichales; f__Erysipelotrichaceae; g__uncultured; s__uncultured_bacterium
b5d53a721ef7707521e9236a5c12438b	d__Bacteria; p__Firmicutes; c__Bacilli; o__Lactobacillales; f__Lactobacillaceae; g__Lactobacillus
6239c62a5d99cefccf8b666387d8bc51	d__Bacteria; p__Bacteroidota; c__Bacteroidia; o__Bacteroidales; f__Muribaculaceae
71200ced87015be2c510d1dd3581c33c	d__Bacteria; p__Bacteroidota; c__Bacteroidia; o__Bacteroidales; f__Bacteroidaceae; g__Bacteroides; s__Bacteroides_sartorii
9b4b7c2baa84d2a290e16a1900b03676	d__Bacteria; p__Firmicutes; c__Bacilli; o__Erysipelotrichales; f__Erysipelatoclostridiaceae
117749c5f275afea00b8e1dc82bdd801	d__Bacteria; p__Bacteroidota; c__Bacteroidia; o__Bacteroidales; f__Muribaculaceae; g__Muribaculaceae; s__uncultured_bacterium
564fc29910bee47525ceda29ce85d28c	d__Bacteria; p__Firmicutes; c__Negativicutes; o__Acidaminococcales; f__Acidaminococcaceae; g__Phascolarctobacterium; s__uncultured_Veillonellaceae
130d60e7f57b5037088a24c73508d46e	d__Bacteria; p__Firmicutes; c__Clostridia; o__Lachnospirales; f__Lachnospiraceae; g__uncultured
41bebc66d8e6fc64e776a127560781f5	d__Bacteria; p__Bacteroidota; c__Bacteroidia; o__Bacteroidales; f__Bacteroidaceae; g__Bacteroides; s__uncultured_bacterium
b7fcc5a94167c8780e65f6cc5993ba2e	d__Bacteria; p__Actinobacteriota; c__Coriobacteriia; o__Coriobacteriales; f__Eggerthellaceae; g__Enterorhabdus; s__uncultured_bacterium
c2d61960d6e80eaf1af2f97aae78addc	d__Bacteria; p__Bacteroidota; c__Bacteroidia; o__Bacteroidales; f__Bacteroidaceae; g__Bacteroides
babb15bc952ec29329dabd55a5a1c68e	d__Bacteria; p__Firmicutes; c__Clostridia; o__Oscillospirales; f__Oscillospiraceae; g__uncultured; s__uncultured_rumen
ae526389f9203043343d9fa8845f2ea7	d__Bacteria; p__Firmicutes; c__Clostridia; o__Oscillospirales; f__Ruminococcaceae; g__Ruminococcus; s__uncultured_rumen
d27ea57592f5bd90753f809e1e55957c	d__Archaea; p__Euryarchaeota; c__Methanobacteria; o__Methanobacteriales; f__Methanobacteriaceae; g__Methanosphaera; s__uncultured_archaeon
debb1dd424a51cfbe7520fe4f20af8d9	d__Bacteria; p__Firmicutes; c__Clostridia; o__Oscillospirales; f__Butyricicoccaceae; g__UCG-008; s__uncultured_bacterium
2173b0a494a7df59c48d8f0a10d62230	d__Bacteria; p__Firmicutes; c__Bacilli; o__Erysipelotrichales; f__Erysipelotrichaceae
565d121f2c1155bc410d4207556c1bdc	d__Bacteria; p__Firmicutes; c__Clostridia; o__Christensenellales; f__Christensenellaceae; g__Christensenellaceae_R-7_group
ff0d1c7da1589b1137c35aa27209f2ef	d__Bacteria; p__Firmicutes; c__Clostridia; o__Lachnospirales; f__Lachnospiraceae
decd770874db64e405109f6125a2fc00	d__Bacteria; p__Firmicutes; c__Clostridia; o__Peptostreptococcales-Tissierellales; f__Anaerovoracaceae; g__[Eubacterium]_nodatum_group
142ebd68374f42e79cf0b89994f97c0d	d__Bacteria; p__Actinobacteriota; c__Coriobacteriia; o__Coriobacteriales; f__Eggerthellaceae; g__Enterorhabdus; s__uncultured_bacterium
09b8f2c99691ea423b746d04c86fa157	d__Bacteria; p__Bacteroidota; c__Bacteroidia; o__Bacteroidales; f__Muribaculaceae; g__Muribaculaceae; s__uncultured_bacterium
5d1b097da0e1d391129680ce26e0f26f	d__Bacteria; p__Firmicutes; c__Bacilli; o__RF39; f__RF39; g__RF39; s__uncultured_bacterium
76d3a36be6ab78a76d14a8de9681b305	d__Bacteria; p__Firmicutes; c__Clostridia; o__Oscillospirales; f__Oscillospiraceae; g__Colidextribacter
910153a84ffc3ccaeb4696190c56faa9	d__Bacteria; p__Firmicutes; c__Clostridia; o__Oscillospirales; f__Ruminococcaceae; g__Ruminococcus; s__unidentified
1c956b363d855e66cdfc1ed3ef323775	d__Bacteria; p__Patescibacteria; c__Saccharimonadia; o__Saccharimonadales; f__Saccharimonadaceae; g__Candidatus_Saccharimonas; s__uncultured_bacterium
3d3ee5669e1dc20b4be7731c973a64c3	d__Bacteria; p__Firmicutes; c__Bacilli; o__Erysipelotrichales; f__Erysipelotrichaceae; g__uncultured; s__uncultured_bacterium
baaa402ce2e12665e7ace5216171d264	d__Bacteria; p__Bacteroidota; c__Bacteroidia; o__Bacteroidales; f__Muribaculaceae; g__Muribaculaceae; s__uncultured_bacterium
dd9c4c02a326a3626aa0557a1b98095d	d__Bacteria; p__Firmicutes; c__Clostridia; o__Eubacteriales; f__Eubacteriaceae; g__uncultured; s__uncultured_bacterium

Supplementary File 5
Relative Abundance Data

FECAL SAMPLE TAXA CONTINUED

de37aecb84ee66e4d7cd0b429a3e8c22	d__Bacteria; p__Firmicutes; c__Bacilli; o__Erysipelotrichales; f__Erysipelotrichaceae
dfb5cac126cb99a80fa0f3e8db187354	d__Bacteria; p__Actinobacteriota; c__Coriobacteriia; o__Coriobacteriales; f__Eggerthellaceae; g__Enterorhabdus; s__uncultured_bacterium
702be321eb5b3e7d9a47d98bb7acbc39	d__Bacteria; p__Bacteroidota; c__Bacteroidia; o__Bacteroidales; f__Muribaculaceae; g__Muribaculaceae; s__uncultured_bacterium
aa9dac8c36ae0d2c812e58023a3483ee	d__Bacteria; p__Bacteroidota; c__Bacteroidia; o__Bacteroidales; f__Muribaculaceae; g__Muribaculaceae; s__uncultured_bacterium
e6fc30e1ee2902ae5f983faa05f33a5d	d__Bacteria; p__Firmicutes; c__Bacilli; o__Lactobacillales; f__Streptococcaceae; g__Streptococcus
1e62cc22168d960ef53dd25ec28aa72a	d__Bacteria; p__Bacteroidota; c__Bacteroidia; o__Bacteroidales; f__Prevotellaceae; g__Prevotellaceae_UCG-003; s__uncultured_bacterium
fc6601f255fcd986001beff6e115a87a	d__Bacteria; p__Bacteroidota; c__Bacteroidia; o__Bacteroidales
2a899b0c2d71448fcac35f78af3b8fea	d__Bacteria; p__Desulfobacterota; c__Desulfovibrionia; o__Desulfovibrionales; f__Desulfovibrionaceae; g__Desulfovibrio
64b9ee9159c84eb32ade4569b9875825	d__Bacteria; p__Bacteroidota; c__Bacteroidia; o__Bacteroidales; f__Muribaculaceae; g__Muribaculaceae; s__uncultured_bacterium
430fd617b217af23f3f39a56b794582e	d__Bacteria; p__Firmicutes; c__Clostridia; o__Oscillospirales; f__Oscillospiraceae
d3ce8b24e18c8865cfc63d1fee6c667a	d__Bacteria; p__Patescibacteria; c__Saccharimonadia; o__Saccharimonadales; f__Saccharimonadaceae; g__Candidatus_Saccharimonas; s__uncultured_bacterium
2146e16ef49b8178b265815d1c9a8a79	d__Bacteria; p__Bacteroidota; c__Bacteroidia; o__Bacteroidales; f__Muribaculaceae; g__Muribaculaceae
e3a77d968b151ea5017b38fc70d37d6c	d__Bacteria; p__Actinobacteriota; c__Coriobacteriia; o__Coriobacteriales; f__Eggerthellaceae; g__Enterorhabdus; s__uncultured_bacterium
46f1a4c4a9bd453d0d7628517e5e7478	d__Bacteria; p__Firmicutes; c__Bacilli; o__Erysipelotrichales; f__Erysipelotrichaceae; g__Faecalibaculum
8f7e26a6dcc3de49c6638b051ccb4b58	d__Bacteria; p__Bacteroidota; c__Bacteroidia; o__Bacteroidales; f__Muribaculaceae; g__Muribaculaceae; s__uncultured_bacterium
151a152695c04bb2a47ad27432299e5c	d__Bacteria; p__Firmicutes; c__Clostridia; o__Oscillospirales; f__Ruminococcaceae; g__Ruminococcus; s__Ruminococcus_sp.
9f784da1124c6b94133c147dd31f97e9	d__Bacteria; p__Bacteroidota; c__Bacteroidia; o__Bacteroidales; f__Muribaculaceae; g__Muribaculaceae
730a261fcd333a25667cd9b555b4321a	d__Bacteria; p__Bacteroidota; c__Bacteroidia; o__Bacteroidales; f__Muribaculaceae; g__Muribaculaceae; s__uncultured_organism
86f3bb6adde5570c794551e32cfe3662	d__Bacteria; p__Bacteroidota; c__Bacteroidia; o__Bacteroidales; f__Muribaculaceae; g__Muribaculaceae; s__uncultured_bacterium
b90f9d6ed20948465b8c6c8eb1e40303	d__Bacteria; p__Patescibacteria; c__Saccharimonadia; o__Saccharimonadales; f__Saccharimonadaceae; g__Candidatus_Saccharimonas; s__uncultured_bacterium
d919bcb48438f57e6bfe82f2ed023cf1	d__Bacteria; p__Actinobacteriota; c__Coriobacteriia; o__Coriobacteriales; f__Eggerthellaceae; g__uncultured; s__uncultured_bacterium
47c07cdf52f56dbec841ef9cc710770a	d__Bacteria; p__Firmicutes; c__Clostridia; o__Lachnospirales; f__Lachnospiraceae; g__Lachnospiraceae_NK4A136_group; s__Clostridiales_bacterium
e1deaf634caf8eb04579ed9df760fbaa	d__Bacteria; p__Firmicutes; c__Clostridia; o__Oscillospirales; f__Oscillospiraceae; g__UCG-005; s__uncultured_bacterium
290934538f39e18a0f8dc93345332d9d	d__Bacteria; p__Actinobacteriota; c__Coriobacteriia; o__Coriobacteriales; f__uncultured; g__uncultured; s__uncultured_bacterium
6059d10ec896a72d7516917bd42f86e8	d__Bacteria; p__Firmicutes; c__Clostridia; o__Lachnospirales; f__Lachnospiraceae; g__Blautia
be95d9f9dd8b536c259b34b7095bedf9	d__Bacteria; p__Bacteroidota; c__Bacteroidia; o__Bacteroidales; f__Prevotellaceae; g__Prevotellaceae_UCG-001; s__uncultured_bacterium
c3c2d15a3da193d36fe380d2c02d09f1	d__Bacteria; p__Firmicutes; c__Clostridia; o__Lachnospirales; f__Lachnospiraceae; g__Lachnospiraceae_UCG-004; s__uncultured_organism
78bed1c9f0b9c51b1465891ba4b955ad	d__Bacteria; p__Firmicutes; c__Clostridia; o__Lachnospirales; f__Lachnospiraceae
a89cf9c5df99eebc3a386ec4706c1419	d__Bacteria; p__Bacteroidota; c__Bacteroidia; o__Bacteroidales; f__Bacteroidaceae; g__Bacteroides; s__Bacteroides_acidifaciens
860e6361852f2cb78289b92fbb533a19	d__Bacteria; p__Actinobacteriota; c__Coriobacteriia; o__Coriobacteriales; f__Eggerthellaceae
4412fb10ed7f18dd250b79adfd851215	d__Bacteria; p__Firmicutes; c__Clostridia; o__Lachnospirales; f__Lachnospiraceae; g__Blautia
ddc3609ee2b75311b538d7c64a97ba28	d__Bacteria; p__Firmicutes; c__Clostridia; o__Lachnospirales; f__Lachnospiraceae
1f7fd4eeac087d35e56ec95c6c34995d	d__Bacteria; p__Firmicutes; c__Clostridia; o__Oscillospirales; f__Ruminococcaceae; g__Incertae_Sedis; s__uncultured_bacterium
308daeac771c02b435ee0ea970a83c94	d__Bacteria; p__Bacteroidota; c__Bacteroidia; o__Bacteroidales; f__Muribaculaceae; g__Muribaculaceae; s__uncultured_bacterium
248a1d7e276fd131ee47d57637928854	d__Bacteria; p__Firmicutes; c__Clostridia; o__Lachnospirales; f__Lachnospiraceae; g__Lachnospiraceae_NK4A136_group; s__uncultured_bacterium
c11ec0b66ddfdc92cd515f722ccecd9b	d__Bacteria; p__Bacteroidota; c__Bacteroidia; o__Bacteroidales; f__Bacteroidaceae; g__Bacteroides; s__Bacteroides_sartorii
d8ad197e6ff5c0be04bf9c86880643bd	d__Bacteria; p__Firmicutes; c__Clostridia; o__Clostridia; f__Hungateiclostridiaceae; g__Ruminiclostridium; s__uncultured_organism
e3bcb75d4083f6e7ffe4e53985976d3e	d__Bacteria; p__Firmicutes; c__Clostridia; o__Monoglobales; f__Monoglobaceae; g__Monoglobus; s__uncultured_organism
c22781c15563e5a6d384fe88898b476b	d__Bacteria; p__Bacteroidota; c__Bacteroidia; o__Bacteroidales; f__Muribaculaceae; g__Muribaculaceae; s__uncultured_bacterium
ee77870e2bd26fd2be3ab1ab874db8c1	d__Bacteria; p__Firmicutes; c__Bacilli; o__Erysipelotrichales; f__Erysipelotrichaceae; g__uncultured; s__uncultured_bacterium
b239e36437f197c1fb5e2331a6da3060	d__Bacteria; p__Firmicutes; c__Clostridia; o__Oscillospirales; f__Ruminococcaceae; g__Incertae_Sedis
4debdb83f8ec77f1717e599877cf303a	d__Bacteria; p__Firmicutes; c__Clostridia; o__Oscillospirales; f__Ruminococcaceae; g__Ruminococcus; s__uncultured_Ruminococcus
d96e3fd43075137a0a9360e99336ce71	d__Bacteria; p__Firmicutes; c__Bacilli; o__Erysipelotrichales; f__Erysipelotrichaceae; g__uncultured; s__uncultured_bacterium
33e76705cc13994f1340ec827e7ff666	d__Bacteria; p__Firmicutes; c__Clostridia; o__Christensenellales; f__Christensenellaceae; g__Christensenellaceae_R-7_group; s__bacterium_YE57
55f579c351ddf7c4cc0e60d47fbd770a	d__Bacteria; p__Firmicutes; c__Clostridia; o__Oscillospirales; f__[Eubacterium]_coprostanoligenes_group; g__[Eubacterium]_coprostanoligenes_group; s__gut_metagenome
f1dd967b46622ea0fcb24a2dc9497cf5	d__Bacteria; p__Bacteroidota; c__Bacteroidia; o__Bacteroidales; f__Muribaculaceae; g__Muribaculaceae; s__uncultured_bacterium
f15ce9131a3c179c1c2a8f72cc90dd9b	d__Bacteria; p__Bacteroidota; c__Bacteroidia; o__Bacteroidales; f__Muribaculaceae; g__Muribaculaceae; s__uncultured_bacterium
2ae6ec466302fa58be2da0b61c4626ab	d__Bacteria; p__Bacteroidota; c__Bacteroidia; o__Bacteroidales; f__Prevotellaceae; g__Prevotella
384f46a83f2253b13803dee52a02bdf4	d__Bacteria; p__Bacteroidota; c__Bacteroidia; o__Bacteroidales; f__Bacteroidaceae; g__Bacteroides; s__Bacteroides_fragilis
0da0d77918b41fc86b8bd6814dfa6960	d__Bacteria; p__Firmicutes; c__Clostridia; o__Clostridia_UCG-014; f__Clostridia_UCG-014; g__Clostridia_UCG-014
4f39197042640bbbc0f0af338e2399c0	d__Bacteria; p__Firmicutes; c__Clostridia; o__Oscillospirales; f__Ruminococcaceae
6cbb76587c3a48fd9696a3196957bf27	d__Bacteria; p__Firmicutes; c__Clostridia; o__Oscillospirales; f__Oscillospiraceae; g__NK4A214_group; s__uncultured_bacterium
742702f8cf8b62709fe1af97421fd77f	d__Bacteria; p__Firmicutes; c__Clostridia; o__Oscillospirales; f__Oscillospiraceae; g__uncultured; s__uncultured_rumen
47a99eaf210693db50113156d3932368	d__Bacteria; p__Firmicutes; c__Clostridia; o__Lachnospirales; f__Lachnospiraceae; g__Lachnoclostridium
e45d21435a010db9cd799f5be89a5237	d__Bacteria; p__Firmicutes; c__Clostridia; o__Oscillospirales; f__Ruminococcaceae; g__Ruminococcus
73f080ad7e4f02f90281e7ce08a30d04	d__Bacteria; p__Bacteroidota; c__Bacteroidia; o__Bacteroidales; f__Muribaculaceae; g__Muribaculaceae
fdca784c68a12837d7961253958ad238	d__Bacteria; p__Firmicutes; c__Clostridia; o__Oscillospirales; f__Ruminococcaceae
6724821b8b000c415022b95ec87aae54	d__Bacteria; p__Bacteroidota; c__Bacteroidia; o__Bacteroidales; f__Muribaculaceae; g__Muribaculaceae; s__uncultured_bacterium
7990b83878bbb5dec1efcb5908a0e7b0	d__Bacteria; p__Firmicutes; c__Clostridia; o__Lachnospirales; f__Lachnospiraceae
cd72fe7ac97bd7a0c1570993ba503bf4	d__Bacteria; p__Firmicutes; c__Clostridia; o__Clostridia_UCG-014; f__Clostridia_UCG-014; g__Clostridia_UCG-014
38a47808d27347f800595bada06cf019	d__Bacteria; p__Firmicutes; c__Clostridia; o__Christensenellales; f__Christensenellaceae; g__Christensenellaceae_R-7_group
3c89e63231e1394bae71fa5c65adca80	d__Bacteria; p__Firmicutes; c__Clostridia; o__Christensenellales; f__Christensenellaceae; g__Christensenellaceae_R-7_group
2fe3ac8300d9a94a79a191e4d4fcd9af	d__Bacteria; p__Bacteroidota; c__Bacteroidia; o__Bacteroidales; f__Muribaculaceae; g__Muribaculaceae; s__uncultured_bacterium
96c946790503fe4c30f1daa38612e571	d__Bacteria; p__Firmicutes; c__Clostridia; o__Oscillospirales; f__Ruminococcaceae; g__Ruminococcus
9d59125c46fa50603a7628b92d5dfdeb	d__Bacteria; p__Bacteroidota; c__Bacteroidia; o__Bacteroidales; f__Tannerellaceae; g__Parabacteroides; s__Parabacteroides_distasonis
7998f9a74ecaa36f051943fc78384446	d__Bacteria; p__Elusimicrobiota; c__Elusimicrobia; o__Elusimicrobiales; f__Elusimicrobiaceae; g__Elusimicrobium; s__uncultured_bacterium
6c21d0ee3e60691d0a6deed26536c6fe	d__Bacteria; p__Firmicutes; c__Clostridia; o__Lachnospirales; f__Defluviitaleaceae; g__Defluviitaleaceae_UCG-011; s__uncultured_bacterium
5077a094f6654bf6cdbe8f4aade747e3	d__Bacteria; p__Firmicutes; c__Clostridia; o__Oscillospirales; f__Oscillospiraceae; g__UCG-005
70f41f9b826c494bb86184a252a1966a	d__Bacteria; p__Bacteroidota; c__Bacteroidia; o__Bacteroidales; f__Marinifilaceae; g__Butyricimonas; s__uncultured_bacterium
5d02b2f791ccc6b28079a8ab906885b3	d__Bacteria; p__Firmicutes; c__Bacilli; o__Erysipelotrichales; f__Erysipelotrichaceae; g__Dubosiella; s__uncultured_Erysipelotrichales
ae5a2eccffb0e02e2e2a21446fe19f9f	d__Bacteria; p__Firmicutes; c__Clostridia; o__Oscillospirales; f__Ruminococcaceae; g__Ruminococcus; s__Ruminococcus_flavefaciens
b722c02f83589262ed10a3932e60f2fe	d__Bacteria; p__Actinobacteriota; c__Coriobacteriia; o__Coriobacteriales; f__Eggerthellaceae
c998c9754d4fb96fcd45cdbde8015333	d__Bacteria; p__Bacteroidota; c__Bacteroidia; o__Bacteroidales; f__Bacteroidaceae; g__Bacteroides; s__Bacteroides_rodentium
f532af63844a3463565141b21b997bf4	d__Bacteria; p__Firmicutes; c__Clostridia; o__Oscillospirales; f__Ruminococcaceae; g__Ruminococcaceae; s__uncultured_bacterium
54ab79bdc8003ea694d5ff977b76abb9	d__Bacteria; p__Firmicutes; c__Clostridia; o__Christensenellales; f__Christensenellaceae; g__Christensenellaceae_R-7_group
c119608de19b53801bea363d987d720a	d__Bacteria; p__Actinobacteriota; c__Coriobacteriia; o__Coriobacteriales; f__Eggerthellaceae; g__Enterorhabdus; s__uncultured_bacterium
fbe017c8fc536520d084115db3e32e36	d__Bacteria; p__Actinobacteriota; c__Actinobacteria; o__Bifidobacteriales; f__Bifidobacteriaceae; g__Bifidobacterium; s__Bifidobacterium_magnum
7c19a77f8e22e5d6995b9f4fd4dd1f98	d__Bacteria; p__Firmicutes; c__Bacilli; o__Lactobacillales; f__Streptococcaceae; g__Streptococcus; s__Streptococcus_criceti
27c9fe1a17289aeed428e15736fcae8b	d__Bacteria; p__Firmicutes; c__Clostridia; o__Oscillospirales; f__Oscillospiraceae; g__uncultured
a2bbd7ee283b7536aea61abf222e2ddc	d__Bacteria; p__Firmicutes; c__Clostridia; o__Oscillospirales; f__Ruminococcaceae; g__Incertae_Sedis; s__uncultured_bacterium
532b672a0cf044e0756edc671395beea	d__Bacteria; p__Actinobacteriota; c__Coriobacteriia; o__Coriobacteriales; f__Eggerthellaceae; g__Enterorhabdus; s__uncultured_bacterium

Supplementary File 5
Relative Abundance Data

FECAL SAMPLE TAXA CONTINUED

12578cbf9e5fa07f032d6f9959219fe0	d__Bacteria; p__Firmicutes; c__Bacilli; o__Lactobacillales; f__Streptococcaceae; g__Streptococcus
56f120eadef210dd81a8f751ab90bf56	d__Bacteria; p__Firmicutes; c__Bacilli; o__Erysipelotrichales; f__Erysipelotrichaceae; g__Allobaculum
af4b0130f942ce77d90a6750865b376e	d__Bacteria; p__Firmicutes; c__Clostridia; o__Monoglobales; f__Monoglobaceae; g__Monoglobus
36965daacad10217bdc814015c51eb9c	d__Bacteria; p__Firmicutes; c__Clostridia; o__Lachnospirales; f__Lachnospiraceae; g__Lachnospiraceae_UCG-001
01d5a8194bf127062916681bdac70f94	d__Bacteria; p__Patescibacteria; c__Saccharimonadia; o__Saccharimonadales; f__Saccharimonadaceae; g__Candidatus_Saccharimonas; s__uncultured_bacterium
cc366d60ab2dceb4768c6e86921f8c6e	d__Bacteria; p__Firmicutes; c__Bacilli; o__Erysipelotrichales; f__Erysipelotrichaceae; g__Allobaculum; s__uncultured_bacterium
37700f44b83ebec11371835191932ac4	d__Bacteria; p__Bacteroidota; c__Bacteroidia; o__Bacteroidales; f__Muribaculaceae; g__Muribaculaceae; s__uncultured_bacterium
6f9d35256a8f2d72716f5195f61ce177	d__Bacteria; p__Actinobacteriota; c__Coriobacteriia; o__Coriobacteriales; f__Eggerthellaceae; g__Paraeggerthella
da83a89a8cde3d1ca67ec80b8297ec33	d__Bacteria; p__Bacteroidota; c__Bacteroidia; o__Bacteroidales; f__Prevotellaceae; g__Prevotellaceae_Ga6A1_group; s__uncultured_bacterium
824f9b3378f96aae3fe31ca0e625c5e5	d__Bacteria; p__Desulfobacterota; c__Desulfovibrionia; o__Desulfovibrionales; f__Desulfovibrionaceae; g__Bilophila
7123122a5076388757cc917661ec2679	d__Bacteria; p__Verrucomicrobiota; c__Verrucomicrobiae; o__Verrucomicrobiales; f__Akkermansiaceae; g__Akkermansia; s__Akkermansia_muciniphila
48bc75a3cf6ede8e4a6a3fc03b5e1005	d__Bacteria; p__Firmicutes; c__Clostridia; o__Lachnospirales; f__Lachnospiraceae
7b10406529ff4462c7e08ffd441cbcfc	d__Bacteria; p__Firmicutes; c__Clostridia; o__Lachnospirales; f__Lachnospiraceae; g__Lachnospira
d126b4cb324a637413e20c014b8acfa0	d__Bacteria; p__Firmicutes; c__Clostridia; o__Lachnospirales; f__Lachnospiraceae
991e16e89cbaeec552db76f922107603	d__Bacteria; p__Firmicutes; c__Clostridia; o__Monoglobales; f__Monoglobaceae; g__Monoglobus
728216da3bc5277cdf518d9343fd5171	d__Bacteria; p__Bacteroidota; c__Bacteroidia; o__Bacteroidales; f__Muribaculaceae; g__Muribaculaceae; s__uncultured_bacterium
e2b6ac8c7987e60f623525842cf0c324	d__Bacteria; p__Bacteroidota; c__Bacteroidia; o__Bacteroidales; f__Muribaculaceae; g__Muribaculaceae; s__uncultured_Muribaculaceae
c012f11c1ed6775b6019b1982ee601a8	d__Bacteria; p__Firmicutes; c__Bacilli; o__Erysipelotrichales; f__Erysipelotrichaceae; g__Allobaculum; s__uncultured_bacterium
aac72e4763b237ca8d1eaf491490e396	d__Bacteria; p__Bacteroidota; c__Bacteroidia; o__Bacteroidales; f__Muribaculaceae; g__Muribaculum
a24ee5420ca8829b431bb5c8f07054ff	d__Bacteria; p__Actinobacteriota; c__Coriobacteriia; o__Coriobacteriales; f__Coriobacteriales_Incertae_Sedis; g__uncultured; s__gut_metagenome
fe17f26f7cf5c90a8a90c397c8076015	d__Bacteria; p__Patescibacteria; c__Saccharimonadia; o__Saccharimonadales; f__Saccharimonadaceae; g__Candidatus_Saccharimonas; s__uncultured_bacterium
696e4f476bb8a4c46f5a50eb90943c39	d__Bacteria; p__Firmicutes; c__Clostridia; o__Oscillospirales; f__[Eubacterium]_coprostanoligenes_group; g__[Eubacterium]_coprostanoligenes_group
f4eba1a7d03c7ab11ab9698eacdff995	d__Bacteria; p__Firmicutes; c__Clostridia; o__Lachnospirales; f__Lachnospiraceae
62780fc3f951f5a6f418fe8b40e6fae1	d__Bacteria; p__Bacteroidota; c__Bacteroidia; o__Bacteroidales; f__Muribaculaceae; g__Muribaculaceae; s__uncultured_bacterium
7268043971f23c0bfcc54c0580261147	d__Bacteria; p__Firmicutes; c__Clostridia; o__Oscillospirales; f__Oscillospiraceae; g__UCG-005; s__uncultured_bacterium
29b78af4bc1dce5f48f1ec1767456bfb	d__Bacteria; p__Patescibacteria; c__Saccharimonadia; o__Saccharimonadales; f__Saccharimonadaceae; g__Candidatus_Saccharimonas; s__uncultured_bacterium
823fd8b097496e923a42da1df9341321	d__Bacteria; p__Firmicutes; c__Clostridia; o__Lachnospirales; f__Lachnospiraceae
9d08b0d29823dcebd27171b1014c2263	d__Bacteria; p__Bacteroidota; c__Bacteroidia; o__Bacteroidales; f__Prevotellaceae; g__Prevotellaceae_UCG-001; s__uncultured_bacterium
6dec7570076f516ac5bdd4a9d3302b2d	d__Bacteria; p__Bacteroidota; c__Bacteroidia; o__Bacteroidales; f__Muribaculaceae; g__Muribaculaceae; s__uncultured_bacterium
b2e75fcb71a420e2ea914cb5c4e3e696	d__Bacteria; p__Firmicutes; c__Clostridia; o__Lachnospirales; f__Lachnospiraceae; g__Lachnoclostridium
4b053376591771cc1a5799349cb000c6	d__Bacteria; p__Firmicutes; c__Clostridia; o__Lachnospirales; f__Lachnospiraceae; g__Lachnoclostridium
d545b17e628425f615c46ee31f3881d5	d__Bacteria; p__Firmicutes; c__Clostridia; o__Oscillospirales; f__Oscillospiraceae; g__Colidextribacter
4e3fd7a92f31906c46c792121fc5b8ab	d__Bacteria; p__Bacteroidota; c__Bacteroidia; o__Bacteroidales; f__Muribaculaceae; g__Muribaculaceae; s__uncultured_bacterium
d94384c0820da27ef5e166759b664ed4	d__Bacteria; p__Firmicutes; c__Bacilli; o__Erysipelotrichales; f__Erysipelotrichaceae; g__Allobaculum; s__uncultured_bacterium
8f592db3a3bd071a02db0c81fc2d70b2	d__Bacteria; p__Firmicutes; c__Clostridia; o__Oscillospirales; f__Oscillospiraceae; g__Colidextribacter
c48e77e038883b8c696c3c3e01f5dd30	d__Bacteria; p__Firmicutes; c__Clostridia; o__Oscillospirales; f__Ruminococcaceae; g__Incertae_Sedis; s__uncultured_bacterium
a2224a8859fc22d395f2ba5ccf4ece5e	d__Bacteria; p__Bacteroidota; c__Bacteroidia; o__Bacteroidales; f__Marinifilaceae; g__Odoribacter; s__uncultured_bacterium
85f204e0a9b546155801dd51d526b101	d__Bacteria; p__Bacteroidota; c__Bacteroidia; o__Bacteroidales; f__Prevotellaceae; g__Prevotella; s__uncultured_bacterium
f031bbe0a53ba435540b145ac13437ba	d__Bacteria; p__Firmicutes; c__Clostridia; o__Oscillospirales; f__Ruminococcaceae; g__Ruminococcus
647d9df392b2554ee19144e04bb55c0e	d__Bacteria; p__Firmicutes; c__Bacilli; o__Lactobacillales; f__Lactobacillaceae; g__Lactobacillus
940546c99e57ec873caf28b878406af3	d__Bacteria; p__Firmicutes; c__Clostridia; o__Lachnospirales; f__Lachnospiraceae; g__Lachnospiraceae_NK4A136_group
06d69fcd0f5624abf23fdde3252c80a3	d__Bacteria; p__Firmicutes; c__Clostridia; o__Eubacteriales; f__Eubacteriaceae; g__uncultured; s__uncultured_bacterium
c557066b0454cc80ca8eddc55b72e162	d__Bacteria; p__Firmicutes; c__Clostridia; o__Lachnospirales; f__Lachnospiraceae
c9a97ed832e4eecc3f74d8c5fe7dfec9	d__Bacteria; p__Bacteroidota; c__Bacteroidia; o__Bacteroidales; f__Muribaculaceae; g__Muribaculaceae; s__uncultured_bacterium
f8619324696671c238395d7772435942	d__Bacteria; p__Firmicutes; c__Clostridia; o__Lachnospirales; f__Lachnospiraceae
3b05aacfe8ac3af35512083031eb74eb	d__Bacteria; p__Firmicutes; c__Clostridia; o__Lachnospirales; f__Lachnospiraceae
f6770ab94332d7547dae2ff625b4421e	d__Bacteria; p__Firmicutes; c__Clostridia; o__Lachnospirales; f__Lachnospiraceae; g__[Eubacterium]_ruminantium_group; s__uncultured_bacterium
10f66e74ffc4e588ccd33335abaccc33	d__Bacteria; p__Firmicutes; c__Bacilli; o__Erysipelotrichales; f__Erysipelotrichaceae
3c2c09073cfac434c119f27f263d62ae	d__Bacteria; p__Firmicutes; c__Clostridia; o__Oscillospirales; f__Oscillospiraceae; g__UCG-005
57fb6e07e216fad06dc84d96449aea2d	d__Bacteria; p__Bacteroidota; c__Bacteroidia; o__Bacteroidales; f__Muribaculaceae; g__Muribaculaceae; s__uncultured_bacterium
c6dfc5871b0aeffe1493fe3297acd454	d__Bacteria; p__Bacteroidota; c__Bacteroidia; o__Bacteroidales; f__Muribaculaceae; g__Muribaculaceae; s__uncultured_bacterium
d5383b802e012ab0da2ee9b505ca0b72	d__Bacteria; p__Firmicutes; c__Clostridia; o__Oscillospirales; f__Ruminococcaceae; g__Incertae_Sedis; s__uncultured_bacterium
63f16b815c40d6d8c355a3591fd99431	d__Bacteria; p__Patescibacteria; c__Saccharimonadia; o__Saccharimonadales; f__Saccharimonadaceae; g__Candidatus_Saccharimonas; s__uncultured_bacterium
a53de8d7e900bff698c8a9665389b2ef	d__Bacteria; p__Bacteroidota; c__Bacteroidia; o__Bacteroidales; f__Muribaculaceae; g__Muribaculaceae; s__uncultured_bacterium
80e834a3b2800e75e8da88d435fc5bc7	d__Bacteria; p__Firmicutes; c__Clostridia; o__Peptostreptococcales-Tissierellales; f__Anaerovoracaceae; g__[Eubacterium]_brachy_group; s__uncultured_bacterium
c6def382820a5c70263a1d8c7b0c94c1	d__Bacteria; p__Firmicutes; c__Clostridia; o__Oscillospirales; f__Oscillospiraceae; g__UCG-005; s__uncultured_bacterium
dcaa4b66cd727671e72eb93aec0476bb	d__Bacteria; p__Bacteroidota; c__Bacteroidia; o__Bacteroidales; f__Bacteroidaceae; g__Bacteroides; s__Bacteroides_eggerthii
32f2c8a05e87c134a440896db3fe12ed	d__Bacteria; p__Bacteroidota; c__Bacteroidia; o__Bacteroidales; f__Muribaculaceae; g__Muribaculaceae; s__uncultured_bacterium
abb67785119de30c73ec370c4d29a813	d__Bacteria; p__Firmicutes; c__Clostridia; o__Oscillospirales; f__[Eubacterium]_coprostanoligenes_group; g__[Eubacterium]_coprostanoligenes_group
fd5adfc2066dea98038aec240c05bb23	d__Bacteria; p__Firmicutes; c__Clostridia; o__Oscillospirales
40dbef84bdc7c9888244792b376ff5ec	d__Bacteria; p__Firmicutes; c__Clostridia; o__Lachnospirales; f__Defluviitaleaceae; g__Defluviitaleaceae_UCG-011; s__uncultured_bacterium
99133dffc3da111c68a41d6a8aa68669	d__Bacteria; p__Firmicutes; c__Clostridia; o__Oscillospirales; f__Ruminococcaceae; g__Incertae_Sedis; s__uncultured_bacterium
6ba55d67c156eeddc4f0c187d7ee1539	d__Bacteria; p__Firmicutes; c__Clostridia; o__Oscillospirales; f__Oscillospiraceae; g__uncultured
01da5f260b187096ca94fbeadf3803ad	d__Bacteria; p__Firmicutes; c__Clostridia; o__Oscillospirales; f__Ruminococcaceae; g__Ruminococcus
c625dddb17671f95d6261a70e013df12	d__Bacteria; p__Firmicutes; c__Clostridia; o__Clostridia_UCG-014; f__Clostridia_UCG-014; g__Clostridia_UCG-014; s__uncultured_rumen
34e5ea04a7f87b701c1cd0386fdeec5d	d__Bacteria; p__Firmicutes; c__Clostridia; o__Oscillospirales; f__Ruminococcaceae; g__UBA1819; s__uncultured_organism
a27c46c6a90285fb40b5965a05f51e25	d__Bacteria; p__Bacteroidota; c__Bacteroidia; o__Bacteroidales; f__Muribaculaceae; g__Muribaculaceae; s__uncultured_bacterium
6dca68bfddf4e16270bf09903be386f8	d__Bacteria; p__Firmicutes; c__Clostridia; o__Oscillospirales; f__Ruminococcaceae; g__Ruminococcus
d1f015ac9efc2f1f3f16b4c55f66e371	d__Bacteria; p__Firmicutes; c__Clostridia; o__Clostridia_UCG-014; f__Clostridia_UCG-014; g__Clostridia_UCG-014; s__unidentified
4b49bd18ce0d7c50711be2e484e06a81	d__Bacteria; p__Firmicutes; c__Bacilli; o__Erysipelotrichales; f__Erysipelotrichaceae
7c26a1bce8d9d7587ce723605a1afc79	d__Bacteria; p__Actinobacteriota; c__Coriobacteriia; o__Coriobacteriales; f__Eggerthellaceae; g__Adlercreutzia
c8149fb09af63dbeaddf836691e004ce	d__Bacteria; p__Firmicutes; c__Clostridia; o__Lachnospirales; f__Lachnospiraceae; g__Blautia
52351dd47889ae3cc525a47bed349ed2	d__Bacteria; p__Firmicutes; c__Clostridia; o__Lachnospirales; f__Lachnospiraceae; g__Marvinbryantia
12d6d5367882b1fdd95a650b3a69f35f	d__Bacteria; p__Bacteroidota; c__Bacteroidia; o__Bacteroidales; f__Rikenellaceae; g__Alistipes; s__uncultured_bacterium
c95f14cbd1b3ccf8bdb97d835710a1b7	d__Bacteria; p__Firmicutes; c__Clostridia; o__Clostridia_UCG-014; f__Clostridia_UCG-014; g__Clostridia_UCG-014
102369d995f55b3ffbed12009ce71be9	d__Bacteria; p__Bacteroidota; c__Bacteroidia; o__Bacteroidales; f__Muribaculaceae; g__Muribaculaceae
6ae424e0753cdaee8e70b5db55b26b0d	d__Bacteria; p__Firmicutes; c__Clostridia; o__Christensenellales; f__Christensenellaceae; g__Christensenellaceae_R-7_group; s__uncultured_prokaryote
89b6022834f3c5ba1e5ba5ec4490a348	d__Bacteria; p__Firmicutes; c__Clostridia; o__Lachnospirales; f__Lachnospiraceae; g__Lachnoclostridium
42873fd9391ac4202da5348751608b07	d__Bacteria; p__Firmicutes; c__Clostridia; o__Clostridia_UCG-014; f__Clostridia_UCG-014; g__Clostridia_UCG-014; s__unidentified
403efa451c839d45298f1b9d1f70be35	d__Bacteria; p__Firmicutes; c__Clostridia; o__Lachnospirales; f__Lachnospiraceae; g__Frisingicoccus
9bd05c1bc0f2936d377059f4dc50bc53	d__Bacteria; p__Actinobacteriota; c__Coriobacteriia; o__Coriobacteriales; f__Atopobiaceae

FECAL SAMPLE TAXA CONTINUED

ae128da86bf22745de825d03eee72a89	d__Bacteria; p__Firmicutes; c__Clostridia; o__Lachnospirales; f__Lachnospiraceae; g__Lachnoclostridium; s__uncultured_Firmicutes
262d81a66b44c642d0a66088fa6d7442	d__Bacteria; p__Firmicutes; c__Bacilli; o__Erysipelotrichales; f__Erysipelotrichaceae
c207154a8e56d0691213c0e0260c87d6	d__Bacteria; p__Firmicutes; c__Clostridia; o__Oscillospirales; f__Oscillospiraceae; g__Papillibacter; s__uncultured_bacterium
cd6ec3a05a59aaed00eac282337fcff4	d__Bacteria; p__Bacteroidota; c__Bacteroidia; o__Bacteroidales; f__Rikenellaceae; g__dgA-11_gut_group; s__uncultured_bacterium
9305f6e96e437f2c400f433856598a51	d__Bacteria; p__Firmicutes; c__Clostridia; o__Lachnospirales; f__Lachnospiraceae; g__Lachnospiraceae_NK4A136_group
ff4b06d9c7a8f4235ce632b24640e472	d__Bacteria; p__Proteobacteria; c__Gammaproteobacteria; o__Burkholderiales; f__Sutterellaceae; g__Parasutterella
3269df4dde8fba2ddf3cda62f76303e0	d__Bacteria; p__Bacteroidota; c__Bacteroidia; o__Bacteroidales; f__Muribaculaceae; g__Muribaculaceae; s__uncultured_bacterium
7b453c045b63166292ff74b00351634d	d__Bacteria; p__Actinobacteriota; c__Coriobacteriia; o__Coriobacteriales; f__Eggerthellaceae; g__Enterorhabdus; s__uncultured_bacterium
27d15b3ad99d9ca6a6657b7ff6300ef0	d__Bacteria; p__Firmicutes; c__Clostridia; o__Lachnospirales; f__Lachnospiraceae
3815df3452041739dfeec838f1529f8d	d__Bacteria; p__Actinobacteriota; c__Coriobacteriia; o__Coriobacteriales; f__Eggerthellaceae; g__Enterorhabdus; s__uncultured_bacterium
ffb31935f877fb0c6add085dd4daf2bf	d__Bacteria; p__Actinobacteriota; c__Coriobacteriia; o__Coriobacteriales; f__Eggerthellaceae
78cbad34947e468ef91dba44921336db	d__Bacteria; p__Bacteroidota; c__Bacteroidia; o__Bacteroidales; f__Muribaculaceae; g__Muribaculaceae; s__uncultured_bacterium
469ce5b20002b4a9843aee01e8672578	d__Bacteria; p__Bacteroidota; c__Bacteroidia; o__Bacteroidales; f__Tannerellaceae; g__Parabacteroides; s__Parabacteroides_distasonis
9ec4a0790531c7b75f361cc0620a89fd	d__Bacteria; p__Firmicutes; c__Bacilli; o__Erysipelotrichales; f__Erysipelotrichaceae; g__uncultured; s__uncultured_bacterium
dbabbb21b99de0113fa544a3bfcb1abd	d__Bacteria; p__Firmicutes; c__Clostridia; o__Monoglobales; f__Monoglobaceae; g__Monoglobus; s__uncultured_bacterium
508e44276bcd5edf3c49a809b65646b2	d__Bacteria; p__Firmicutes; c__Clostridia; o__Oscillospirales; f__Ruminococcaceae; g__Ruminococcus; s__unidentified
e81e8cd6f6f35a0c8ef5bad2728e8fc2	d__Bacteria; p__Firmicutes; c__Clostridia; o__Oscillospirales; f__Oscillospiraceae; g__UCG-005; s__uncultured_bacterium
dc1faaaddaeb97166418766b5c1dda14	d__Bacteria; p__Firmicutes; c__Clostridia; o__Christensenellales; f__Christensenellaceae; g__Christensenellaceae_R-7_group
5e13054d296ad3f96a136e271aee0fe8	d__Bacteria; p__Actinobacteriota; c__Coriobacteriia; o__Coriobacteriales; f__Coriobacteriaceae; g__Collinsella; s__Collinsella_sp.
b321a0cf14235da980ebff3431c69cda	d__Bacteria; p__Actinobacteriota; c__Coriobacteriia; o__Coriobacteriales; f__Eggerthellaceae
cabb43d30ad8d8164d51aa79f357ebd5	d__Bacteria; p__Bacteroidota; c__Bacteroidia; o__Bacteroidales; f__Muribaculaceae; g__Muribaculaceae
9056430b057dbaf9adaf7e8f016a226e	d__Bacteria; p__Firmicutes; c__Bacilli; o__Erysipelotrichales; f__Erysipelotrichaceae; g__Ileibacterium; s__uncultured_bacterium
30272824c66faa8804b14c1462e97f9b	d__Bacteria; p__Firmicutes; c__Clostridia; o__Christensenellales; f__Christensenellaceae; g__Christensenellaceae_R-7_group
916d07cf4b54c77e92cd5b8b22d40be5	d__Bacteria; p__Firmicutes; c__Bacilli; o__Lactobacillales; f__Lactobacillaceae; g__Lactobacillus; s__Lactobacillus_murinus
135cc0ec925d92babed963dcbbb5852c	d__Bacteria; p__Firmicutes; c__Clostridia; o__Christensenellales; f__Christensenellaceae
5fa02ea22112e839954473443951d695	d__Bacteria; p__Bacteroidota; c__Bacteroidia; o__Bacteroidales; f__Muribaculaceae; g__Muribaculaceae; s__uncultured_bacterium
525eace45984d928c5da79169ed2c78b	d__Archaea; p__Euryarchaeota; c__Methanobacteria; o__Methanobacteriales; f__Methanobacteriaceae; g__Methanobrevibacter
8134463c8af08c7e21ce88a90d113025	d__Bacteria; p__Firmicutes; c__Clostridia; o__Oscillospirales; f__[Eubacterium]_coprostanoligenes_group; g__[Eubacterium]_coprostanoligenes_group; s__uncultured_bacterium
9047d4adb73ddd7c6564382555d99a3b	d__Bacteria; p__Bacteroidota; c__Bacteroidia; o__Bacteroidales; f__Prevotellaceae; g__Prevotella; s__uncultured_bacterium
b875d28b5f7da4396da25f484e3ed3fd	d__Bacteria; p__Firmicutes; c__Clostridia; o__Christensenellales; f__Christensenellaceae; g__Christensenellaceae_R-7_group
112cd74b05b36c9b556a04a127c642e5	d__Bacteria; p__Firmicutes; c__Clostridia; o__Oscillospirales; f__Ruminococcaceae; g__Ruminococcus
efd8bfa27c098e2e12925e5937f51501	d__Bacteria; p__Firmicutes; c__Clostridia; o__Oscillospirales; f__Ruminococcaceae; g__Ruminococcus; s__Ruminococcus_sp.
f54ee87d634d32508d5dc79644110131	d__Bacteria; p__Firmicutes; c__Clostridia; o__Oscillospirales; f__Oscillospiraceae; g__Colidextribacter
01458476d70cb77a9d3db6f4a576ece4	d__Bacteria; p__Bacteroidota; c__Bacteroidia; o__Bacteroidales; f__Muribaculaceae; g__Muribaculaceae; s__uncultured_bacterium
8b56f3a30f0c261ed5fc3e7a9bfa8912	d__Bacteria; p__Firmicutes; c__Bacilli; o__Lactobacillales; f__Streptococcaceae; g__Streptococcus; s__Streptococcus_hyointestinalis
770e757de65a7f296955758c5f527e7a	d__Bacteria; p__Firmicutes; c__Bacilli; o__RF39; f__RF39; g__RF39
b01d5cfb319a7f92034e73d3f4c4a123	d__Bacteria; p__Firmicutes; c__Clostridia; o__Monoglobales; f__Monoglobaceae; g__Monoglobus; s__uncultured_rumen
e75b087e5e93c68bcec26499a5e57872	d__Bacteria; p__Firmicutes; c__Clostridia; o__Eubacteriales; f__Eubacteriaceae; g__uncultured; s__uncultured_bacterium
fe32fd6c90021d2639fe07ce0ceeb43e	d__Bacteria; p__Bacteroidota; c__Bacteroidia; o__Bacteroidales; f__Muribaculaceae; g__Muribaculaceae; s__uncultured_bacterium
01aa81d6a6edc625ebfb60fa674f3c9c	d__Bacteria; p__Bacteroidota; c__Bacteroidia; o__Bacteroidales; f__Bacteroidaceae; g__Bacteroides; s__Bacteroides_thetaiotaomicron
66156d80d43206e221242dd74cd8301a	d__Bacteria; p__Firmicutes; c__Bacilli; o__Erysipelotrichales; f__Erysipelotrichaceae; g__Allobaculum
eeb6b2492eb7c8325fc8bdbd88156fea	d__Bacteria; p__Firmicutes; c__Bacilli; o__RF39; f__RF39; g__RF39; s__uncultured_Lachnospiraceae
23852fc8929eace1fde4b3a69472608f	d__Bacteria; p__Bacteroidota; c__Bacteroidia; o__Bacteroidales; f__Muribaculaceae; g__Muribaculaceae; s__uncultured_bacterium
353882b669eab143a6681ec79e1b5036	d__Bacteria; p__Firmicutes; c__Clostridia; o__Oscillospirales; f__Ruminococcaceae; g__Ruminococcus
60b65da26ec7fff4fd9c013793f38994	d__Bacteria; p__Firmicutes; c__Clostridia; o__Oscillospirales; f__Ruminococcaceae; g__Ruminococcus
876dc021928ab83a546ec299d1823ba4	d__Bacteria; p__Firmicutes; c__Clostridia; o__Lachnospirales; f__Lachnospiraceae; g__Lachnospiraceae_NK4A136_group
76003ab07100e4504d15ddf980c621c7	d__Bacteria; p__Firmicutes; c__Clostridia; o__Christensenellales; f__Christensenellaceae; g__Christensenellaceae_R-7_group; s__uncultured_prokaryote
ec3992faf76a3e0d55ed68c6b7fa8165	d__Bacteria; p__Firmicutes; c__Bacilli; o__Erysipelotrichales; f__Erysipelotrichaceae
158327867995219c03ecfd5cd9763d17	d__Bacteria; p__Bacteroidota; c__Bacteroidia; o__Bacteroidales; f__Tannerellaceae; g__Parabacteroides; s__Parabacteroides_distasonis
1586c258313cf3499e0d7a98c5a7ffa0	d__Bacteria; p__Actinobacteriota; c__Coriobacteriia; o__Coriobacteriales; f__Eggerthellaceae
56611ada065dabde0cb5c5ff3e8c4532	d__Bacteria; p__Firmicutes; c__Clostridia; o__Oscillospirales; f__Oscillospiraceae; g__Oscillibacter
85987ad3bd8f243f96c52e9b92cd122a	d__Bacteria; p__Firmicutes; c__Clostridia; o__Oscillospirales; f__Oscillospiraceae; g__UCG-005
b0ab93d4128b07531086a5e0d01de399	d__Bacteria; p__Firmicutes; c__Clostridia; o__Christensenellales; f__Christensenellaceae; g__Christensenellaceae_R-7_group
64b874ca2db7fd58fce41ce8c00cc943	d__Bacteria; p__Firmicutes; c__Clostridia; o__Oscillospirales; f__Ruminococcaceae; g__Incertae_Sedis; s__uncultured_bacterium
940e67bbf860b02d1993a3da36958cd8	d__Bacteria; p__Firmicutes; c__Clostridia; o__Peptostreptococcales-Tissierellales; f__Anaerovoracaceae; g__Mogibacterium; s__uncultured_bacterium
27b990b25dc6b591b5bb4d5c684ebbc0	d__Bacteria; p__Firmicutes; c__Clostridia; o__Lachnospirales; f__Lachnospiraceae; g__Coprococcus
fa148ce9984012a46e731e6743a1a9f8	d__Bacteria; p__Firmicutes; c__Clostridia; o__Clostridia_UCG-014; f__Clostridia_UCG-014; g__Clostridia_UCG-014
812d3b1f13c63d595e5944db697c6a65	d__Bacteria; p__Fusobacteriota; c__Fusobacteriia; o__Fusobacteriales; f__Fusobacteriaceae; g__Fusobacterium; s__Fusobacterium_varium
97f712e6adfb03ad82be19a4721d6fe7	d__Bacteria; p__Firmicutes; c__Clostridia; o__Lachnospirales; f__Lachnospiraceae; g__Lachnospiraceae_NK4A136_group
5880580612bc1f1fcbdfd01386d78492	d__Bacteria; p__Bacteroidota; c__Bacteroidia; o__Bacteroidales; f__Rikenellaceae; g__Alistipes; s__Alistipes_inops
d91e63eb57429d8a1e15195a7c444389	d__Bacteria; p__Bacteroidota; c__Bacteroidia; o__Bacteroidales; f__Muribaculaceae; g__Muribaculaceae; s__uncultured_bacterium
099466f8f413f0a9de2f891571e7f045	d__Bacteria; p__Bacteroidota; c__Bacteroidia; o__Bacteroidales; f__Rikenellaceae; g__Alistipes; s__Alistipes_shahii
9c9f5ba2230399c5ed6db4f00954de22	d__Bacteria; p__Firmicutes; c__Clostridia; o__Lachnospirales; f__Lachnospiraceae; g__[Eubacterium]_ruminantium_group; s__uncultured_bacterium
397d2a7d28c63e90b5c7b3ac35b95252	d__Bacteria; p__Bacteroidota; c__Bacteroidia; o__Bacteroidales; f__Muribaculaceae; g__Muribaculaceae; s__uncultured_bacterium
66e848c1c4ff4c5aa6bfaf013604d430	d__Bacteria; p__Bacteroidota; c__Bacteroidia; o__Bacteroidales; f__Muribaculaceae; g__Muribaculaceae; s__uncultured_bacterium
80b45f1d34c62b3328818e643650bbfd	d__Bacteria; p__Actinobacteriota; c__Coriobacteriia; o__Coriobacteriales; f__Eggerthellaceae
7212a22e450a7ed06864716e66eab86e	d__Bacteria; p__Bacteroidota; c__Bacteroidia; o__Bacteroidales; f__Muribaculaceae; g__Muribaculaceae
bbb98bd770d1fcf9d17d49b85d080e61	d__Bacteria; p__Firmicutes; c__Clostridia; o__Lachnospirales; f__Lachnospiraceae; g__Lachnospiraceae_UCG-010; s__uncultured_bacterium
930f48b87d9ded1db03ccc9675328672	d__Bacteria; p__Firmicutes; c__Clostridia; o__Oscillospirales; f__Ruminococcaceae; g__Ruminococcus
99c130232acc8f9bbd4807b7be421ffa	d__Bacteria; p__Firmicutes; c__Clostridia; o__Oscillospirales; f__Ruminococcaceae; g__Incertae_Sedis; s__uncultured_bacterium
4c04280a25f2445b846ba7533074da26	d__Bacteria; p__Firmicutes; c__Clostridia; o__Lachnospirales; f__Lachnospiraceae
4aff84ae489bcbf725ca0721ef6f8049	d__Bacteria; p__Actinobacteriota; c__Coriobacteriia; o__Coriobacteriales; f__Eggerthellaceae; g__Enterorhabdus; s__uncultured_bacterium
fb7f6f699a6794e29a5454981b693e75	d__Bacteria; p__Bacteroidota; c__Bacteroidia; o__Bacteroidales; f__Prevotellaceae; g__Prevotellaceae_NK3B31_group; s__uncultured_bacterium
2bc79bf3a76fd3cdff24f55dc974b109	d__Bacteria; p__Firmicutes; c__Clostridia; o__Peptostreptococcales-Tissierellales; f__Anaerovoracaceae; g__Family_XIII_AD3011_group; s__uncultured_bacterium
cdd657033b671c6204946a9d95af9ad8	d__Bacteria; p__Firmicutes; c__Bacilli; o__Erysipelotrichales; f__Erysipelotrichaceae
59a4463900368aa7f44cc429c0b72717	d__Bacteria; p__Bacteroidota; c__Bacteroidia; o__Bacteroidales; f__Muribaculaceae; g__Muribaculaceae; s__uncultured_bacterium
d96ffd532b073b69f0405ae5c8f3bc5d	d__Bacteria; p__Bacteroidota; c__Bacteroidia; o__Bacteroidales; f__Muribaculaceae; g__Muribaculaceae; s__uncultured_bacterium
b4256283220c5a220d1b9152a4f674e8	d__Bacteria; p__Firmicutes; c__Clostridia; o__Lachnospirales; f__Lachnospiraceae
1eb86b1f8db465bfd231bb18ba729be1	d__Bacteria; p__Firmicutes; c__Clostridia; o__Peptostreptococcales-Tissierellales; f__Anaerovoracaceae; g__Family_XIII_AD3011_group
7fefe035f3b27412b0948e3d3528198a	d__Bacteria; p__Firmicutes; c__Clostridia; o__Oscillospirales; f__Oscillospiraceae
5fdef3befdb143c70aa9f173213e9dae	d__Bacteria; p__Firmicutes; c__Clostridia; o__Peptostreptococcales-Tissierellales; f__Anaerovoracaceae; g__Family_XIII_AD3011_group; s__uncultured_bacterium

Supplementary File 5
Relative Abundance Data

FECAL SAMPLE TAXA CONTINUED

5ef39dbc53fa461a6cae0952a2738cf0	d__Bacteria; p__Firmicutes; c__Clostridia; o__Oscillospirales; f__Oscillospiraceae; g__NK4A214_group
c1c048eaac9caa64f0990cb7015bd528	d__Bacteria; p__Bacteroidota; c__Bacteroidia; o__Bacteroidales; f__Prevotellaceae; g__Prevotellaceae_UCG-001; s__uncultured_bacterium
e5efd987a62040e998a5f46ba5bc77d7	d__Bacteria; p__Bacteroidota; c__Bacteroidia; o__Bacteroidales; f__Muribaculaceae; g__Muribaculaceae; s__uncultured_Bacteroidales
910657ea807f247e5c193a874f6616d2	d__Bacteria; p__Firmicutes; c__Bacilli; o__Erysipelotrichales; f__Erysipelatoclostridiaceae; g__Erysipelatoclostridiaceae; s__uncultured_bacterium
c5644403c8167a6e9537b16154a857e5	d__Bacteria; p__Patescibacteria; c__Saccharimonadia; o__Saccharimonadales; f__Saccharimonadaceae; g__Candidatus_Saccharimonas
b7c94fea15b6345c35d99fbe8ccf8f44	d__Bacteria; p__Firmicutes; c__Clostridia; o__Lachnospirales; f__Lachnospiraceae; g__Dorea
772d8340f287b749f911043260658429	d__Bacteria; p__Bacteroidota; c__Bacteroidia; o__Bacteroidales; f__Muribaculaceae; g__Muribaculaceae; s__uncultured_bacterium
1c0e5f5fd68e0ace342f39aa9d3046c6	d__Bacteria; p__Bacteroidota; c__Bacteroidia; o__Bacteroidales; f__Muribaculaceae; g__Muribaculaceae; s__uncultured_bacterium
41017b318591a04ce579c77cc761a509	d__Bacteria; p__Bacteroidota; c__Bacteroidia; o__Bacteroidales; f__Muribaculaceae; g__Muribaculaceae; s__uncultured_bacterium
591256b01a466d6ebfd827e3cb8f184f	d__Bacteria; p__Firmicutes; c__Clostridia; o__Lachnospirales; f__Lachnospiraceae
295b3e1e585f4da5b074f19eddc3bfb3	d__Bacteria; p__Patescibacteria; c__Saccharimonadia; o__Saccharimonadales; f__Saccharimonadaceae; g__Candidatus_Saccharimonas; s__uncultured_bacterium
7008725b2d1322ebceffc1323c66f7aa	d__Bacteria; p__Bacteroidota; c__Bacteroidia; o__Bacteroidales; f__Muribaculaceae; g__Muribaculaceae
66413ea4d4ffcaf49747051deaa8467e	d__Bacteria; p__Bacteroidota; c__Bacteroidia; o__Bacteroidales; f__Muribaculaceae; g__Muribaculaceae
5ab9226ed8c91613810a98bcc0dfb6af	d__Bacteria; p__Proteobacteria; c__Gammaproteobacteria; o__Enterobacterales; f__Enterobacteriaceae; g__Escherichia-Shigella
cbb24db86a179c1bef082d497598bde6	d__Bacteria; p__Firmicutes; c__Clostridia; o__Oscillospirales; f__Ruminococcaceae; g__Incertae_Sedis
69e7116dc594c166ede483165c112139	d__Bacteria; p__Firmicutes; c__Clostridia; o__Lachnospirales; f__Lachnospiraceae; g__Marvinbryantia; s__uncultured_bacterium
0b29ae5866b512cbbacfcfe70aac585e	d__Bacteria; p__Actinobacteriota; c__Coriobacteriia; o__Coriobacteriales; f__Atopobiaceae; g__uncultured; s__uncultured_bacterium
175292821a2247cd0e1691dc6174e093	d__Bacteria; p__Actinobacteriota; c__Coriobacteriia; o__Coriobacteriales; f__Coriobacteriales_Incertae_Sedis; g__uncultured; s__gut_metagenome
bba94e120964d557a954d2b43ba5759f	d__Bacteria; p__Bacteroidota; c__Bacteroidia; o__Bacteroidales; f__Bacteroidaceae; g__Bacteroides; s__Bacteroides_caccae
1cbda2af087151e80322549bb52b7d11	d__Bacteria; p__Actinobacteriota; c__Coriobacteriia; o__Coriobacteriales; f__Eggerthellaceae; g__uncultured; s__uncultured_bacterium
f701f7f8f8aacaadc0c21412f993d04f	d__Bacteria; p__Firmicutes; c__Clostridia; o__Lachnospirales; f__Lachnospiraceae
9674961dd11e5ebbc5167304bbcf126a	d__Bacteria; p__Bacteroidota; c__Bacteroidia; o__Bacteroidales; f__Muribaculaceae; g__Muribaculaceae; s__uncultured_bacterium
fa0d3238e4dcef137aee6f7dec33da40	d__Bacteria; p__Firmicutes; c__Clostridia; o__Lachnospirales; f__Lachnospiraceae; g__Blautia
ff8b43b36749f0af30b19d560e75dae7	d__Archaea; p__Euryarchaeota; c__Methanobacteria; o__Methanobacteriales; f__Methanobacteriaceae; g__Methanobrevibacter
8929237d22993e390d646bfa1827c38d	d__Bacteria; p__Bacteroidota; c__Bacteroidia; o__Bacteroidales; f__Muribaculaceae; g__Muribaculaceae; s__mouse_gut
0454a5b339657901d2d0f7fc4ed17b34	d__Bacteria; p__Firmicutes; c__Clostridia; o__Lachnospirales; f__Lachnospiraceae; g__uncultured; s__uncultured_bacterium
3c62693ef461bb977cdeeec36ea277fd	d__Bacteria; p__Bacteroidota; c__Bacteroidia; o__Bacteroidales; f__Prevotellaceae; g__Prevotellaceae_UCG-001; s__uncultured_bacterium
493557152d2faa8200be031eb1b3322e	d__Bacteria; p__Firmicutes; c__Clostridia; o__Monoglobales; f__Monoglobaceae; g__Monoglobus; s__uncultured_bacterium
5545f2bf2f6854d516a6f31cca93c9c2	d__Bacteria; p__Firmicutes; c__Clostridia; o__Oscillospirales; f__Oscillospiraceae; g__UCG-005; s__uncultured_bacterium
befd10a4c430bd6f35f6f29328ab6928	d__Bacteria; p__Firmicutes; c__Clostridia; o__Lachnospirales; f__Lachnospiraceae; g__Lachnospiraceae_NK4A136_group; s__Trichinella_pseudospiralis
069a972d01f877569b2196b0595b6d5f	d__Bacteria; p__Bacteroidota; c__Bacteroidia; o__Bacteroidales; f__Bacteroidaceae; g__Bacteroides
061b6c4e167260ccf6cc31e456476679	d__Bacteria; p__Firmicutes; c__Clostridia; o__Christensenellales; f__Christensenellaceae; g__Christensenellaceae_R-7_group
06d943dcea12b9f7c68345989c19ea60	d__Bacteria; p__Firmicutes; c__Clostridia; o__Lachnospirales; f__Lachnospiraceae
5394aca0925a650736070d95ab11b97b	d__Bacteria; p__Firmicutes; c__Clostridia; o__Christensenellales; f__Christensenellaceae; g__Christensenellaceae_R-7_group; s__uncultured_prokaryote
79966f722bb4e8c54b8364ad922cf496	d__Bacteria; p__Firmicutes; c__Clostridia; o__Christensenellales; f__Christensenellaceae; g__Christensenellaceae_R-7_group
99ae3d7beceb134360c06f0c745cb686	d__Bacteria; p__Firmicutes; c__Clostridia; o__Oscillospirales; f__Oscillospiraceae
d112d6a256d3b4f865da870415917e06	d__Bacteria; p__Actinobacteriota; c__Actinobacteria; o__Actinomycetales; f__Actinomycetaceae; g__Actinomycetaceae; s__uncultured_Actinomycetaceae
5424a16c4a28650ecf15020444946cbe	d__Bacteria; p__Bacteroidota; c__Bacteroidia; o__Bacteroidales; f__Muribaculaceae; g__Muribaculaceae; s__uncultured_bacterium
fd382be370d1d3630fb012d4aba721a3	d__Bacteria; p__Firmicutes; c__Clostridia; o__Lachnospirales; f__Lachnospiraceae; g__Lachnospiraceae_NK4A136_group
d525781ec0026412c370fd0ec05d32d1	d__Bacteria; p__Firmicutes; c__Bacilli; o__Erysipelotrichales; f__Erysipelotrichaceae; g__Allobaculum; s__uncultured_bacterium
5a596fd5c935509e0d3042a9c0e28610	d__Bacteria; p__Firmicutes; c__Clostridia; o__Oscillospirales; f__Oscillospiraceae; g__UCG-005
94ce33fda7b12f1c935a6d3467d8050f	d__Bacteria; p__Firmicutes; c__Clostridia; o__Lachnospirales; f__Lachnospiraceae
ca878fa8a5e541bb075ec26edefc1ed2	d__Bacteria; p__Firmicutes; c__Clostridia; o__Clostridia_UCG-014; f__Clostridia_UCG-014; g__Clostridia_UCG-014; s__unidentified
16c90dd639effe41873eabde8435f811	d__Bacteria; p__Spirochaetota; c__Spirochaetia; o__Spirochaetales; f__Spirochaetaceae; g__Treponema
0039474bf08f239c459e09a0591614ec	d__Bacteria; p__Bacteroidota; c__Bacteroidia; o__Bacteroidales; f__Muribaculaceae; g__Muribaculaceae; s__uncultured_bacterium
ff98deb342b0f40403c06a6bc5128e31	d__Bacteria; p__Firmicutes; c__Clostridia; o__Oscillospirales; f__Ruminococcaceae; g__[Eubacterium]_siraeum_group; s__uncultured_bacterium
7a0fa2ffdc8bcb8be85c2fcd2b8574ec	d__Bacteria; p__Actinobacteriota; c__Coriobacteriia; o__Coriobacteriales; f__Atopobiaceae; g__uncultured; s__uncultured_bacterium
e8c0bf8330e85da4add38e261fb8410d	d__Bacteria; p__Firmicutes; c__Clostridia; o__Lachnospirales; f__Lachnospiraceae; g__[Ruminococcus]_torques_group
f4e4ad0023e7517b931ff52351a30546	d__Bacteria; p__Spirochaetota; c__Spirochaetia; o__Spirochaetales; f__Spirochaetaceae; g__Treponema; s__uncultured_bacterium
9f0130655e15c7bac78f893ed6f9b633	d__Bacteria; p__Verrucomicrobiota; c__Verrucomicrobiae; o__Verrucomicrobiales; f__Akkermansiaceae; g__Akkermansia; s__uncultured_bacterium
0f987b75c258494a2b936f439a49fa4c	d__Bacteria; p__Firmicutes; c__Bacilli; o__RF39; f__RF39; g__RF39
c98784fa2a0d220f304443d95da8cb40	d__Bacteria; p__Bacteroidota; c__Bacteroidia; o__Bacteroidales; f__Muribaculaceae; g__Muribaculaceae; s__uncultured_bacterium
bc70e42845504d7ca28bbc47481ac481	d__Bacteria; p__Patescibacteria; c__Saccharimonadia; o__Saccharimonadales; f__Saccharimonadaceae; g__Candidatus_Saccharimonas; s__uncultured_bacterium
d59fcd47846d61b8e2c0d7b512906680	d__Bacteria; p__Firmicutes; c__Clostridia; o__Monoglobales; f__Monoglobaceae; g__Monoglobus; s__uncultured_bacterium
78a532074285b9b271bd9aca96a99479	d__Bacteria; p__Bacteroidota; c__Bacteroidia; o__Bacteroidales; f__Prevotellaceae; g__Prevotellaceae_UCG-001
1d77965d782cf4ce2d6baf5d4168e510	d__Bacteria; p__Firmicutes; c__Clostridia; o__Oscillospirales; f__Oscillospiraceae
50e74de68869a95997cae13214a74b7d	d__Bacteria; p__Bacteroidota; c__Bacteroidia; o__Bacteroidales; f__Muribaculaceae; g__Muribaculaceae
87e51a59b0f0f44f3fad24134b8071bd	d__Bacteria; p__Bacteroidota; c__Bacteroidia; o__Bacteroidales; f__Muribaculaceae; g__Muribaculaceae
ff952b53a6daaf1ff18a8fa9459bc755	d__Bacteria; p__Firmicutes; c__Clostridia; o__Lachnospirales; f__Lachnospiraceae
a864ccdaea134671742f3aa8c8bfb27e	d__Bacteria; p__Firmicutes; c__Clostridia; o__Oscillospirales; f__Oscillospiraceae; g__UCG-005; s__uncultured_bacterium
ac49161335c15e94b0abb384933df068	d__Bacteria; p__Firmicutes; c__Clostridia; o__Oscillospirales; f__[Eubacterium]_coprostanoligenes_group; g__[Eubacterium]_coprostanoligenes_group

Supplementary File 5
Relative Abundance Data

NPL SAMPLE TAXA
0.01% in 10% Conditional Filtered Taxa

**Feature ID**	**Taxon ID**
39997fb6f4dda788741ac7dbd90d462e	d__Bacteria; p__Actinobacteriota; c__Actinobacteria; o__Actinomycetales; f__Actinomycetaceae; g__Mobiluncus
78c1191bdd2b70d5410b502ea2af7b30	d__Bacteria; p__Actinobacteriota; c__Actinobacteria; o__Bifidobacteriales; f__Bifidobacteriaceae; g__Bifidobacterium
e8338e54732377ded53aefc4b6724df1	d__Bacteria; p__Campilobacterota; c__Campylobacteria; o__Campylobacterales; f__Campylobacteraceae; g__Campylobacter; s__uncultured_Campylobacter
f508ec2f3cb9a30a030890d2cc426f1f	d__Bacteria; p__Bacteroidota; c__Bacteroidia; o__Bacteroidales
9f0340fbbe35e0494418baa818878ce4	d__Bacteria; p__Bacteroidota; c__Bacteroidia; o__Bacteroidales; f__Muribaculaceae; g__Muribaculaceae
515033aab092baef28bed03fc034be2d	d__Bacteria; p__Actinobacteriota; c__Coriobacteriia; o__Coriobacteriales; f__Atopobiaceae; g__Olsenella
7b396dbe8d94284752e128c119406d09	d__Bacteria; p__Actinobacteriota; c__Actinobacteria; o__Actinomycetales; f__Actinomycetaceae; g__Mobiluncus
3293c6d6a7a598bdb85961865c250de6	d__Bacteria; p__Firmicutes; c__Clostridia; o__Peptostreptococcales-Tissierellales; f__Peptostreptococcales-Tissierellales; g__Peptoniphilus; s__Peptoniphilus_olsenii
c9769b61332c32a844e4e89949e70c59	d__Bacteria; p__Actinobacteriota; c__Actinobacteria; o__Actinomycetales; f__Actinomycetaceae
94e71f9a3d353a924c1a4038e8b205d9	d__Bacteria; p__Firmicutes; c__Bacilli; o__Lactobacillales; f__Aerococcaceae; g__Aerococcus
c4a48a26de0a805b16ae14c034b0afbb	d__Bacteria; p__Firmicutes; c__Bacilli; o__Staphylococcales; f__Staphylococcaceae; g__Staphylococcus
a888b7f563a79225174c799cf7959cd3	d__Bacteria; p__Campilobacterota; c__Campylobacteria; o__Campylobacterales; f__Campylobacteraceae; g__Campylobacter; s__uncultured_Campylobacter
cfa1e2e6c8acbf9474bd45964ca3dc75	d__Bacteria; p__Fusobacteriota; c__Fusobacteriia; o__Fusobacteriales; f__Fusobacteriaceae; g__Fusobacterium; s__Fusobacterium_sp.
f24722692657f1cf8111d2923d93aab3	d__Bacteria; p__Campilobacterota; c__Campylobacteria; o__Campylobacterales; f__Campylobacteraceae; g__Campylobacter; s__uncultured_Campylobacter
1c641c73af5c68f3e84e75c0b90ec42c	d__Bacteria; p__Actinobacteriota; c__Actinobacteria; o__Actinomycetales; f__Actinomycetaceae
8b41ca9b1d8b3633497bff30ec4610e9	d__Bacteria; p__Campilobacterota; c__Campylobacteria; o__Campylobacterales; f__Campylobacteraceae; g__Campylobacter; s__uncultured_Campylobacter
0f1256254623f91f9e5244294684bbdb	d__Bacteria; p__Proteobacteria; c__Gammaproteobacteria; o__Enterobacterales; f__Enterobacteriaceae; g__Escherichia-Shigella
210352bccf3537c7299a29970fafd3de	d__Bacteria; p__Fusobacteriota; c__Fusobacteriia; o__Fusobacteriales; f__Fusobacteriaceae; g__Fusobacterium; s__Fusobacterium_sp.
25b1f8009809d66950a13dc1e0f41d6c	d__Bacteria; p__Campilobacterota; c__Campylobacteria; o__Campylobacterales; f__Campylobacteraceae; g__Campylobacter; s__uncultured_Campylobacter
c977ca5640cddafcd80d5ccc615e7930	d__Bacteria; p__Bacteroidota; c__Bacteroidia; o__Bacteroidales; f__Muribaculaceae; g__Muribaculaceae
609b10521218d57e04d2a1b24c8285b7	d__Bacteria; p__Actinobacteriota; c__Actinobacteria; o__Actinomycetales; f__Actinomycetaceae
df0583952fcdc5e9777855f10c3b9d99	d__Bacteria; p__Campilobacterota; c__Campylobacteria; o__Campylobacterales; f__Campylobacteraceae; g__Campylobacter; s__uncultured_Campylobacter
b22e03c4a1fd9b48be9f345df3e261c8	d__Archaea; p__Euryarchaeota; c__Methanobacteria; o__Methanobacteriales; f__Methanobacteriaceae; g__Methanobrevibacter; s__uncultured_Methanobacteriales
a759802a9107c948c16ed5b93ef356ba	d__Bacteria; p__Bacteroidota; c__Bacteroidia; o__Sphingobacteriales; f__Sphingobacteriaceae
f70cc88fce6a8d7e10ea28f6b27c6ac6	d__Bacteria; p__Campilobacterota; c__Campylobacteria; o__Campylobacterales; f__Campylobacteraceae; g__Campylobacter; s__uncultured_Campylobacter
0d5a257632f50eb30086f3552af0c8e5	d__Bacteria; p__Firmicutes; c__Bacilli; o__Lactobacillales; f__Streptococcaceae; g__Streptococcus; s__Streptococcus_merionis
d03ec53dcad2acb72ccf186b3119aaf3	d__Bacteria; p__Campilobacterota; c__Campylobacteria; o__Campylobacterales; f__Campylobacteraceae; g__Campylobacter; s__uncultured_Campylobacter
7e2e7823c6161e51ab0e9de94f935666	d__Bacteria; p__Bacteroidota; c__Bacteroidia; o__Sphingobacteriales; f__Sphingobacteriaceae
0c5173311401ca3b76a77c0cad57b316	d__Bacteria; p__Proteobacteria; c__Gammaproteobacteria; o__Enterobacterales; f__Enterobacteriaceae
f2fb19f1caa2f1af0c2ac00cbcac9306	d__Bacteria; p__Firmicutes; c__Bacilli; o__Erysipelotrichales; f__Erysipelotrichaceae; g__Ileibacterium; s__uncultured_bacterium
4144e2392cc674e4f9b2bc78a96eb23d	d__Bacteria; p__Proteobacteria; c__Gammaproteobacteria; o__Burkholderiales
f63c0f89d0b4577ffcd152233628d3de	d__Bacteria; p__Firmicutes; c__Bacilli; o__Staphylococcales; f__Staphylococcaceae; g__Staphylococcus
ea5a4816fff06092bead41aaa465c06d	d__Bacteria; p__Actinobacteriota; c__Actinobacteria; o__Actinomycetales; f__Actinomycetaceae
55b63e633318170d9a6625f7dd4407b8	d__Bacteria; p__Bacteroidota; c__Bacteroidia; o__Chitinophagales; f__Chitinophagaceae
a81f52dc3b76391da1d52c1d6c0bf197	d__Bacteria; p__Campilobacterota; c__Campylobacteria; o__Campylobacterales; f__Campylobacteraceae; g__Campylobacter; s__uncultured_Campylobacter
9930a020ef33306fd263af76dbcc9d33	d__Bacteria; p__Actinobacteriota; c__Actinobacteria; o__Actinomycetales; f__Actinomycetaceae; g__Mobiluncus
7f284a474919b3776de06281c3d11e22	d__Bacteria; p__Firmicutes; c__Bacilli; o__Erysipelotrichales; f__Erysipelotrichaceae; g__Ileibacterium; s__uncultured_bacterium
ff50f85d1595d560939cf1e6a85369c8	d__Bacteria; p__Firmicutes; c__Bacilli; o__Erysipelotrichales; f__Erysipelotrichaceae
6f4aa3e5577cf1d3de65db204cd8c8e2	d__Bacteria; p__Proteobacteria; c__Gammaproteobacteria; o__Enterobacterales; f__Enterobacteriaceae; g__Escherichia-Shigella
3af38dfe51cb188490b6d1e3fb231ec6	d__Bacteria; p__Proteobacteria; c__Gammaproteobacteria; o__Enterobacterales; f__Enterobacteriaceae
59bb12385e0e58d607e3c959140e7e2b	d__Bacteria; p__Patescibacteria; c__Saccharimonadia; o__Saccharimonadales; f__Saccharimonadales; g__Saccharimonadales; s__Candidatus_Saccharibacteria
37ce295df48a44fcb2ab1819dace8cec	d__Bacteria; p__Firmicutes; c__Bacilli; o__Staphylococcales; f__Staphylococcaceae; g__Staphylococcus
fd30d0939f2c7b1d02f646501a80b65a	d__Bacteria; p__Firmicutes; c__Bacilli; o__Lactobacillales; f__Streptococcaceae; g__Streptococcus; s__Streptococcus_equi
c9e31d250de3ac82d1a86aedd8dd6bbb	d__Bacteria; p__Bacteroidota; c__Bacteroidia; o__Bacteroidales; f__Prevotellaceae
63350afe4425f4b9342f482c8016eda5	d__Bacteria; p__Bacteroidota; c__Bacteroidia; o__Bacteroidales
29c89ba67ba4b71729733f3c982188ad	d__Bacteria; p__Firmicutes; c__Bacilli; o__Staphylococcales; f__Staphylococcaceae; g__Staphylococcus
5f4753d173117297247db0734cba35f6	d__Bacteria; p__Bacteroidota; c__Bacteroidia; o__Bacteroidales
c40d47b7daf465791fd1ccf83178c621	d__Bacteria; p__Campilobacterota; c__Campylobacteria; o__Campylobacterales; f__Campylobacteraceae; g__Campylobacter; s__uncultured_Campylobacter
da6fa4909dad8cf0b8d457490ef2b607	d__Bacteria; p__Actinobacteriota; c__Actinobacteria; o__Actinomycetales; f__Actinomycetaceae; g__Mobiluncus
f826b4d72b767904eefb4376058264a9	d__Bacteria; p__Proteobacteria; c__Gammaproteobacteria; o__Burkholderiales
80a73d172c8bfdf6cc2028a4eab3b8b5	d__Bacteria; p__Fusobacteriota; c__Fusobacteriia; o__Fusobacteriales; f__Leptotrichiaceae
28754e5303cd0555a8764732e49a9a38	d__Bacteria; p__Fusobacteriota; c__Fusobacteriia; o__Fusobacteriales; f__Fusobacteriaceae; g__Fusobacterium
83bc760378eae0d52b4775ee5a72b724	d__Bacteria; p__Bacteroidota; c__Bacteroidia; o__Bacteroidales; f__Muribaculaceae; g__Muribaculaceae
9a60c39a4b6d590d16aaa03fc4d4fb08	d__Bacteria; p__Patescibacteria; c__Saccharimonadia; o__Saccharimonadales; f__Saccharimonadales; g__Saccharimonadales; s__Candidatus_Saccharibacteria
bbe0a789b455c562ddfdff00a400bcc9	d__Bacteria; p__Proteobacteria; c__Gammaproteobacteria; o__Pasteurellales; f__Pasteurellaceae; g__Actinobacillus; s__Actinobacillus_sp.
4b593894d7b49d50e0b98a5d375adf1f	d__Bacteria; p__Firmicutes; c__Bacilli; o__Mycoplasmatales; f__Mycoplasmataceae; g__Mycoplasma
5c328a7faba76b2b485a22846ff97c87	d__Bacteria; p__Bacteroidota; c__Bacteroidia; o__Chitinophagales; f__Chitinophagaceae
2e3d3897b4a98089fb5529072a69b01b	d__Bacteria; p__Bacteroidota; c__Bacteroidia; o__Sphingobacteriales; f__Sphingobacteriaceae
6ba68a0aa1751418fb6e7d189ed7758a	d__Bacteria; p__Campilobacterota; c__Campylobacteria; o__Campylobacterales; f__Campylobacteraceae; g__Campylobacter; s__uncultured_Campylobacter
400e518f671c966c2815ea5cd53782ad	d__Bacteria; p__Proteobacteria; c__Gammaproteobacteria; o__Enterobacterales; f__Enterobacteriaceae; g__Escherichia-Shigella
132dbd5080b591d6f6c79a5580fba18a	d__Bacteria; p__Firmicutes; c__Bacilli; o__Staphylococcales; f__Staphylococcaceae; g__Staphylococcus
665cc09c177ad9aac3d94f30286745af	d__Bacteria; p__Firmicutes; c__Bacilli; o__Erysipelotrichales; f__Erysipelotrichaceae
cefb014991db5c8c1d4f09cfb68832b6	d__Bacteria; p__Actinobacteriota; c__Actinobacteria; o__Corynebacteriales; f__Corynebacteriaceae; g__Corynebacterium
4f365f8b99df497f81b500d026a5a3fa	d__Bacteria; p__Bacteroidota; c__Bacteroidia; o__Bacteroidales
f7a63e3a65fbe056b919b971e2f63c9b	d__Bacteria; p__Firmicutes; c__Clostridia; o__Eubacteriales; f__Eubacteriaceae; g__uncultured; s__uncultured_bacterium
d6f0424867babcfb53a908d0fff8f38d	d__Bacteria; p__Firmicutes; c__Bacilli; o__Erysipelotrichales; f__Erysipelotrichaceae; g__Ileibacterium; s__uncultured_bacterium
52c5045c4b6e45a2a69010e298f6ea14	d__Bacteria; p__Proteobacteria; c__Gammaproteobacteria; o__Xanthomonadales; f__Xanthomonadaceae; g__Xanthomonas
a65b263d5cc61f6cf325966bc961516a	d__Bacteria; p__Fusobacteriota; c__Fusobacteriia; o__Fusobacteriales; f__Leptotrichiaceae
cba2983f63db59f36ecaa49227ce7f43	d__Bacteria; p__Actinobacteriota; c__Actinobacteria; o__Actinomycetales; f__Actinomycetaceae; g__Mobiluncus
fa2ac46dc3919bb3f6eb67cbd91eb13f	d__Bacteria; p__Bacteroidota; c__Bacteroidia; o__Bacteroidales; f__Muribaculaceae; g__Muribaculaceae
ea3eaec89a3316fb8ee28c925e24f984	d__Bacteria; p__Firmicutes; c__Bacilli; o__Staphylococcales; f__Staphylococcaceae; g__Staphylococcus; s__Staphylococcus_pettenkoferi
56d9597fdaa8c2d310ec0acd99f06e51	d__Bacteria; p__Proteobacteria; c__Gammaproteobacteria; o__Pasteurellales; f__Pasteurellaceae; g__Actinobacillus; s__Actinobacillus_sp.
42325bf18e5c6c0c666aaefc03be47e8	d__Bacteria; p__Actinobacteriota; c__Actinobacteria; o__Bifidobacteriales; f__Bifidobacteriaceae; g__Bifidobacterium
4aee77955dcfc2af663b2044e8ec19ec	d__Bacteria; p__Firmicutes; c__Bacilli; o__Mycoplasmatales; f__Mycoplasmataceae; g__Mycoplasma
43eefa87936875e978fc6437ba00535e	d__Bacteria; p__Bacteroidota; c__Bacteroidia; o__Sphingobacteriales; f__Sphingobacteriaceae
3c03222a7cc276c04341fe9df70df758	d__Bacteria; p__Proteobacteria; c__Gammaproteobacteria; o__Enterobacterales; f__Enterobacteriaceae; g__Escherichia-Shigella
d6bf67259d0bbf6905fdf2fd9b3ecc24	d__Bacteria; p__Bacteroidota; c__Bacteroidia; o__Sphingobacteriales; f__Sphingobacteriaceae
ba0d55d76f7d5e635e4c6d0fc7e08261	d__Bacteria; p__Firmicutes; c__Bacilli; o__Lactobacillales; f__Streptococcaceae; g__Streptococcus; s__Streptococcus_merionis

Supplementary File 5
Relative Abundance Data

**NPL SAMPLE TAXA CONTINUED**	
7c0a96a5e7f15be36a7f307d5876fc15	d__Bacteria; p__Bacteroidota; c__Bacteroidia; o__Bacteroidales; f__Muribaculaceae; g__Muribaculaceae
0247ef726702c50899ac2126d0c1540d	d__Bacteria; p__Firmicutes; c__Bacilli; o__Staphylococcales; f__Staphylococcaceae; g__Staphylococcus
bf52982085cc28a43ef1acedcb86e525	d__Bacteria; p__Bacteroidota; c__Bacteroidia; o__Bacteroidales
4006e73c231487f839249debed173cd7	d__Bacteria; p__Firmicutes; c__Bacilli; o__Erysipelotrichales; f__Erysipelotrichaceae
7ef281b7a40df9a2d11d9dcbca950888	d__Bacteria; p__Proteobacteria; c__Gammaproteobacteria; o__Xanthomonadales; f__Xanthomonadaceae; g__Xanthomonas
fcb7e2b6fd1dd55ab1e2b817216676f5	d__Bacteria; p__Bacteroidota; c__Bacteroidia; o__Bacteroidales; f__Bacteroidaceae; g__Bacteroides
6991e8e3afa991e29c93eaf86d9939e3	d__Bacteria; p__Firmicutes; c__Clostridia; o__Lachnospirales; f__Lachnospiraceae
979d9786d57ad8481559c92b8e86d5ee	d__Bacteria; p__Actinobacteriota; c__Actinobacteria; o__Actinomycetales; f__Actinomycetaceae; g__Mobiluncus
542d74d4d7cff8bbb9e611423286de77	d__Bacteria; p__Actinobacteriota; c__Actinobacteria; o__Bifidobacteriales; f__Bifidobacteriaceae; g__Bifidobacterium
5c1d1e4c99513e9f3bd694b954df0d96	d__Bacteria; p__Firmicutes; c__Bacilli; o__Staphylococcales; f__Staphylococcaceae; g__Staphylococcus
21dbfa8d2597bbbba9c53a400c9c29fb	d__Bacteria; p__Actinobacteriota; c__Actinobacteria; o__Actinomycetales; f__Actinomycetaceae; g__Mobiluncus
6c0d077003854af3e97b82733ef2df7c	d__Bacteria; p__Campilobacterota; c__Campylobacteria; o__Campylobacterales; f__Campylobacteraceae; g__Campylobacter; s__uncultured_Campylobacter
7a3c8748467d44d76c3c705272898486	d__Bacteria; p__Firmicutes; c__Bacilli; o__Mycoplasmatales; f__Mycoplasmataceae; g__Mycoplasma
991065bd12489b20c2c34e8a210976a1	d__Bacteria; p__Proteobacteria; c__Gammaproteobacteria; o__Enterobacterales; f__Enterobacteriaceae
46c152524b8ab16ced2fcab41d3b3f17	d__Bacteria; p__Bacteroidota; c__Bacteroidia; o__Chitinophagales; f__Chitinophagaceae
3fb3fdfee3e8a0647073d5d0074dab71	d__Bacteria; p__Proteobacteria; c__Gammaproteobacteria; o__Enterobacterales; f__Enterobacteriaceae
9ad10641a2f39ebf829978ffce85ba51	d__Bacteria; p__Firmicutes; c__Bacilli; o__Staphylococcales; f__Staphylococcaceae; g__Staphylococcus
d1b957ee8ce94b795ffccca44ce4c535	d__Bacteria; p__Proteobacteria; c__Gammaproteobacteria; o__Burkholderiales
02da5c516418c19c717df3a0a92b7e58	d__Bacteria; p__Bacteroidota; c__Bacteroidia; o__Bacteroidales
3014b1d25e104c7d7af19403685901d3	d__Bacteria; p__Proteobacteria; c__Gammaproteobacteria; o__Burkholderiales
9f9e2b6560024805f591ddf181cd4dbf	d__Bacteria; p__Proteobacteria; c__Gammaproteobacteria; o__Burkholderiales
23749cf0513ebabcbd9ddd033baacd2d	d__Bacteria; p__Campilobacterota; c__Campylobacteria; o__Campylobacterales; f__Campylobacteraceae; g__Campylobacter; s__uncultured_Campylobacter
340c9ccbd7128ad9ffae33c07f42d9e5	d__Bacteria; p__Bacteroidota; c__Bacteroidia; o__Chitinophagales; f__Chitinophagaceae
bf47b73d2faddf87f10e1be20acb3ab6	d__Bacteria; p__Proteobacteria; c__Gammaproteobacteria; o__Burkholderiales
91c069a7323db9f35d8d5c59e5ebf422	d__Bacteria; p__Fusobacteriota; c__Fusobacteriia; o__Fusobacteriales; f__Leptotrichiaceae
f365d7906b0398706135ed235fbd58a4	d__Bacteria; p__Bacteroidota; c__Bacteroidia; o__Bacteroidales; f__Bacteroidaceae; g__Bacteroides
a43dba8945d55a90f972b3cdabaa73aa	d__Bacteria; p__Firmicutes; c__Bacilli; o__Staphylococcales; f__Staphylococcaceae; g__Staphylococcus
e729849e1e47248d0bfff63b36999604	d__Bacteria; p__Campilobacterota; c__Campylobacteria; o__Campylobacterales; f__Campylobacteraceae; g__Campylobacter; s__uncultured_Campylobacter
c70dc57dc9e509951ef2553141389756	d__Bacteria; p__Firmicutes; c__Clostridia; o__Eubacteriales; f__Eubacteriaceae; g__uncultured; s__uncultured_bacterium
57084c09971f8229effbf5140d92598e	d__Bacteria; p__Firmicutes; c__Bacilli; o__Mycoplasmatales; f__Mycoplasmataceae; g__Mycoplasma
3fb951ea7621d8501d390a2f5693749a	d__Bacteria; p__Firmicutes; c__Bacilli; o__Erysipelotrichales; f__Erysipelotrichaceae; g__Ileibacterium; s__uncultured_bacterium
dea5889d35e6bb531597e4a8c45a115e	d__Bacteria; p__Actinobacteriota; c__Actinobacteria; o__Actinomycetales; f__Actinomycetaceae; g__Mobiluncus
dd035bd4b15734f9b0ba6294d065aa0b	d__Bacteria; p__Actinobacteriota; c__Actinobacteria; o__Actinomycetales; f__Actinomycetaceae; g__Mobiluncus
b8b5f04ff98c164782023d4516b4ff42	d__Bacteria; p__Actinobacteriota; c__Actinobacteria; o__Micrococcales
7fa2db535f4bacb876ff074c21311d15	d__Bacteria; p__Bacteroidota; c__Bacteroidia; o__Sphingobacteriales; f__Sphingobacteriaceae
2f85e6da767979c6042ac856c84df15c	d__Bacteria; p__Patescibacteria; c__Saccharimonadia; o__Saccharimonadales; f__Saccharimonadales; g__Saccharimonadales
cbb52f76962bc5ddbe1d694f8ea20555	d__Bacteria; p__Firmicutes; c__Bacilli; o__Erysipelotrichales; f__Erysipelotrichaceae; g__Ileibacterium; s__uncultured_bacterium
05be0580829cd220ce3cf6f9541a401a	d__Bacteria; p__Campilobacterota; c__Campylobacteria; o__Campylobacterales; f__Campylobacteraceae; g__Campylobacter; s__uncultured_Campylobacter
fab0d977927068d644ef2c99e79e2573	d__Bacteria; p__Firmicutes; c__Bacilli; o__Mycoplasmatales; f__Mycoplasmataceae; g__Mycoplasma
bb4dad8eb99c4ab6265fd5226a798ea4	d__Bacteria; p__Firmicutes; c__Bacilli; o__Erysipelotrichales; f__Erysipelotrichaceae
54ea308cec2e159307fecbbdffa8ccbb	d__Bacteria; p__Patescibacteria; c__Saccharimonadia; o__Saccharimonadales; f__Saccharimonadales; g__Saccharimonadales; s__Candidatus_Saccharibacteria
d88c98c1ae38ee0b93ff0076b81e77b4	d__Bacteria; p__Firmicutes; c__Bacilli; o__Staphylococcales; f__Staphylococcaceae; g__Staphylococcus
e7f64dc8b236d5440b63c9000dbe8461	d__Bacteria; p__Bacteroidota; c__Bacteroidia; o__Bacteroidales; f__Muribaculaceae; g__Muribaculaceae
5962d31b1eb335bb3c650e8a7e83cf17	d__Bacteria; p__Firmicutes; c__Clostridia; o__Peptostreptococcales-Tissierellales; f__Peptostreptococcales-Tissierellales; g__Peptoniphilus; s__Peptoniphilus_olsenii
258dd227416ab970c386f45ab25c1ced	d__Bacteria; p__Firmicutes; c__Bacilli; o__Staphylococcales; f__Staphylococcaceae; g__Staphylococcus
4e20305c6bd6dddfa12624db77a8c97d	d__Bacteria; p__Bacteroidota; c__Bacteroidia; o__Bacteroidales
e4c8b6ab9a0f9eece3f5163f12d1c546	d__Bacteria; p__Bacteroidota; c__Bacteroidia; o__Bacteroidales
3339685a39627b6105dc3334dab6685b	d__Bacteria; p__Firmicutes; c__Bacilli; o__Staphylococcales; f__Staphylococcaceae; g__Staphylococcus
f7248330776b9c5450be7f84000c1615	d__Bacteria; p__Bacteroidota; c__Bacteroidia; o__Sphingobacteriales; f__Sphingobacteriaceae
b47544eefbbe4a09c36a86a7b50b1ea2	d__Bacteria; p__Firmicutes; c__Bacilli; o__Lactobacillales; f__Aerococcaceae; g__Aerococcus
86c0bcedf228bcccf44d1d49b8bc4552	d__Bacteria; p__Proteobacteria; c__Gammaproteobacteria; o__Burkholderiales
42110a784389d16efa5edfc5aad2963a	d__Archaea; p__Euryarchaeota; c__Methanobacteria; o__Methanobacteriales; f__Methanobacteriaceae; g__Methanobrevibacter; s__uncultured_Methanobacteriales
6fc2d96345e0347bfb2bee713f979f03	d__Bacteria; p__Bacteroidota; c__Bacteroidia; o__Sphingobacteriales; f__Sphingobacteriaceae
9ff8c30bc5ba267dab3f06e5ec7a5af4	d__Bacteria; p__Campilobacterota; c__Campylobacteria; o__Campylobacterales; f__Campylobacteraceae; g__Campylobacter; s__uncultured_Campylobacter
69934052ae461d07c43b25be920edd3f	d__Bacteria; p__Proteobacteria; c__Gammaproteobacteria; o__Xanthomonadales; f__Xanthomonadaceae; g__Xanthomonas
14bd024e814f02164b6f2bc11bb5e495	d__Bacteria; p__Proteobacteria; c__Gammaproteobacteria; o__Burkholderiales
63fec17b3b527a5b4ad4e3a9780356f7	d__Bacteria; p__Fusobacteriota; c__Fusobacteriia; o__Fusobacteriales; f__Fusobacteriaceae; g__Fusobacterium
6e5f3f5afbcfea0ca07fcf06ccc776a5	d__Bacteria; p__Firmicutes; c__Bacilli; o__Mycoplasmatales; f__Mycoplasmataceae; g__Mycoplasma
40dd8ae90bd7002df8df279e72674f4a	d__Bacteria; p__Bacteroidota; c__Bacteroidia; o__Bacteroidales; f__Prevotellaceae; g__Prevotellaceae_UCG-001; s__uncultured_bacterium
19f7c576f8900635b26c74da69319856	d__Bacteria; p__Firmicutes; c__Bacilli; o__Erysipelotrichales; f__Erysipelotrichaceae; g__Ileibacterium; s__uncultured_bacterium
6debba36773017a4f145d7083d46ce5c	d__Bacteria; p__Patescibacteria; c__Saccharimonadia; o__Saccharimonadales; f__Saccharimonadales; g__Saccharimonadales; s__Candidatus_Saccharibacteria
856743bc5eb9956c95647a26c07901b9	d__Bacteria; p__Campilobacterota; c__Campylobacteria; o__Campylobacterales; f__Campylobacteraceae; g__Campylobacter; s__uncultured_Campylobacter

Supplementary File 5
Relative Abundance Data

Baseline Fecal Genus Level Taxon Frequency	AC-PO7d	AC-10mg/kg	AC-5mg/kg	anti-rsPilA	anti-tip-chimer	Ofloxacin	TS-30mg/kg	TS-15mg/kg	Saline
d__Bacteria;p__Firmicutes;c__Clostridia;o__Oscillospirales;f__Oscillospiraceae;g__uncultured	1343	329	302	1547	304	263	744	903	364
d__Bacteria;p__Firmicutes;c__Bacilli;o__Erysipelotrichales;f__Erysipelotrichaceae;__	12886	7225	3312	4031	3527	2998	3254	8600	3117
d__Bacteria;p__Firmicutes;c__Clostridia;o__Christensenellales;f__Christensenellaceae;g__Christensenellaceae_R-7_group	4842	1615	391	3001	1456	752	351	3375	686
d__Bacteria;p__Proteobacteria;c__Gammaproteobacteria;o__Pasteurellales;f__Pasteurellaceae;g__Haemophilus	0	0	0	24	96	0	0	17	0
d__Bacteria;p__Bacteroidota;c__Bacteroidia;o__Bacteroidales;__;__	1422	11	94	229	101	179	192	218	87
d__Bacteria;p__Actinobacteriota;c__Actinobacteria;o__Actinomycetales;f__Actinomycetaceae;g__Actinomyces	20	167	36	40	32	8	52	135	0
d__Bacteria;p__Patescibacteria;c__Saccharimonadia;o__Saccharimonadales;f__Saccharimonadaceae;g__Candidatus_Saccharimonas	4495	1589	1872	1692	960	6557	2581	2599	1911
d__Bacteria;p__Bacteroidota;c__Bacteroidia;o__Bacteroidales;f__Muribaculaceae;g__Muribaculaceae	45291	3026	5369	7859	8405	3840	7950	10159	5886
d__Bacteria;p__Firmicutes;c__Clostridia;o__Lachnospirales;f__Lachnospiraceae;__	16461	913	940	1493	3886	478	863	1837	1196
d__Bacteria;p__Actinobacteriota;c__Coriobacteriia;o__Coriobacteriales;f__Eggerthellaceae;g__Enterorhabdus	1340	692	280	375	487	343	229	568	176
d__Bacteria;p__Firmicutes;c__Clostridia;o__Clostridia_UCG-014;f__Clostridia_UCG-014;g__Clostridia_UCG-014	2582	510	266	451	451	379	627	527	245
d__Bacteria;p__Actinobacteriota;c__Coriobacteriia;o__Coriobacteriales;f__uncultured;g__uncultured	176	30	9	13	24	66	45	195	79
d__Bacteria;p__Firmicutes;c__Bacilli;o__Erysipelotrichales;f__Erysipelotrichaceae;g__Allobaculum	613	129	2771	475	1093	99	26	410	743
d__Bacteria;p__Firmicutes;c__Clostridia;o__Oscillospirales;f__Ruminococcaceae;g__Incertae_Sedis	2383	462	580	890	323	720	412	1164	562
d__Bacteria;p__Firmicutes;c__Bacilli;o__Erysipelotrichales;f__Erysipelotrichaceae;g__uncultured	3885	862	4305	1398	1420	649	329	803	1314
d__Bacteria;p__Bacteroidota;c__Bacteroidia;o__Bacteroidales;f__Marinifilaceae;g__Butyricimonas	104	0	15	8	17	0	5	40	19
d__Bacteria;p__Actinobacteriota;c__Coriobacteriia;o__Coriobacteriales;__;__	383	11	125	85	128	287	35	30	14
d__Bacteria;p__Firmicutes;c__Clostridia;o__Lachnospirales;f__Lachnospiraceae;g__Marvinbryantia	324	89	0	119	103	113	0	0	0
d__Bacteria;p__Firmicutes;c__Clostridia;o__Peptostreptococcales-Tissierellales;f__Anaerovoracaceae;g__[Eubacterium]_nodatum_group	429	16	94	113	210	97	16	92	0
d__Bacteria;p__Firmicutes;c__Clostridia;o__Oscillospirales;f__Oscillospiraceae;g__NK4A214_group	625	40	167	182	296	93	23	286	175
d__Bacteria;p__Bacteroidota;c__Bacteroidia;o__Bacteroidales;f__Rikenellaceae;g__Rikenellaceae_RC9_gut_group	378	3	16	0	40	18	62	50	68
d__Bacteria;p__Bacteroidota;c__Bacteroidia;o__Bacteroidales;f__Prevotellaceae;g__Prevotellaceae_UCG-001	4241	740	222	144	68	706	1025	1575	972
d__Bacteria;p__Firmicutes;c__Clostridia;o__Oscillospirales;f__[Eubacterium]_coprostanoligenes_group;g__[Eubacterium]_coprostanoligenes_group	584	31	54	712	343	124	145	615	83
d__Bacteria;p__Bacteroidota;c__Bacteroidia;o__Bacteroidales;f__Prevotellaceae;g__Prevotella	6806	441	398	278	290	743	787	303	803
d__Bacteria;p__Planctomycetota;c__Planctomycetes;o__Pirellulales;f__Pirellulaceae;g__p-1088-a5_gut_group	209	117	51	34	91	81	35	245	42
d__Bacteria;p__Firmicutes;c__Clostridia;o__Oscillospirales;f__Ruminococcaceae;g__Ruminococcus	3981	886	732	768	821	907	1665	1645	1083
d__Bacteria;p__Bacteroidota;c__Bacteroidia;o__Bacteroidales;f__Prevotellaceae;g__Prevotellaceae_Ga6A1_group	307	0	23	38	83	58	148	31	87
d__Bacteria;p__Bacteroidota;c__Bacteroidia;o__Bacteroidales;f__Bacteroidaceae;g__Bacteroides	3087	157	315	843	257	180	353	579	269
d__Bacteria;p__Firmicutes;c__Bacilli;o__Erysipelotrichales;f__Erysipelotrichaceae;g__Ileibacterium	2040	250	4141	1367	2031	475	86	1994	2067
d__Bacteria;p__Firmicutes;c__Clostridia;o__Monoglobales;f__Monoglobaceae;g__Monoglobus	1555	761	226	413	378	541	371	227	160
d__Bacteria;p__Firmicutes;c__Clostridia;o__Lachnospirales;f__Lachnospiraceae;g__Coprococcus	116	0	58	408	33	61	29	168	18
d__Bacteria;p__Firmicutes;c__Bacilli;o__Lactobacillales;f__Lactobacillaceae;g__Lactobacillus	506	0	477	624	75	15	0	205	867
d__Bacteria;p__Bacteroidota;c__Bacteroidia;o__Bacteroidales;f__Muribaculaceae;__	248	395	0	148	0	0	54	0	63
d__Bacteria;p__Firmicutes;c__Bacilli;o__Erysipelotrichales;f__Erysipelatoclostridiaceae;__	116	0	0	96	30	0	54	0	0
d__Bacteria;p__Firmicutes;c__Negativicutes;o__Acidaminococcales;f__Acidaminococcaceae;g__Phascolarctobacterium	1498	423	229	183	144	362	142	376	303
d__Bacteria;p__Firmicutes;c__Clostridia;o__Lachnospirales;f__Lachnospiraceae;g__uncultured	714	10	192	123	9	18	278	164	68
d__Archaea;p__Euryarchaeota;c__Methanobacteria;o__Methanobacteriales;f__Methanobacteriaceae;g__Methanosphaera	1833	208	570	0	3	135	59	221	275
d__Bacteria;p__Firmicutes;c__Clostridia;o__Oscillospirales;f__Butyricicoccaceae;g__UCG-008	315	37	0	153	203	0	36	18	28
d__Bacteria;p__Firmicutes;c__Bacilli;o__RF39;f__RF39;g__RF39	265	42	16	253	178	84	171	400	157
d__Bacteria;p__Firmicutes;c__Clostridia;o__Oscillospirales;f__Oscillospiraceae;g__Colidextribacter	670	91	216	197	214	39	326	213	183
d__Bacteria;p__Firmicutes;c__Clostridia;o__Eubacteriales;f__Eubacteriaceae;g__uncultured	5316	2042	1574	1580	1879	488	514	3566	2296
d__Bacteria;p__Firmicutes;c__Bacilli;o__Lactobacillales;f__Streptococcaceae;g__Streptococcus	715	35	75	378	210	169	60	289	547
d__Bacteria;p__Bacteroidota;c__Bacteroidia;o__Bacteroidales;f__Prevotellaceae;g__Prevotellaceae_UCG-003	154	0	22	129	60	199	0	0	0
d__Bacteria;p__Desulfobacterota;c__Desulfovibrionia;o__Desulfovibrionales;f__Desulfovibrionaceae;g__Desulfovibrio	174	13	78	0	0	0	0	0	74
d__Bacteria;p__Firmicutes;c__Clostridia;o__Oscillospirales;f__Oscillospiraceae;__	1486	229	124	188	24	281	184	438	108
d__Bacteria;p__Firmicutes;c__Bacilli;o__Erysipelotrichales;f__Erysipelotrichaceae;g__Faecalibaculum	164	25	78	58	8	8	26	0	19
d__Bacteria;p__Actinobacteriota;c__Coriobacteriia;o__Coriobacteriales;f__Eggerthellaceae;g__uncultured	746	459	110	1233	568	297	209	631	230

Supplementary File 5
Relative Abundance Data

Baseline Fecal Genus Level Taxon Frequency Continued	AC-PO7d	AC-10mg/kg	AC-5mg/kg	anti-rsPilA	anti-tip-chimer	Ofloxacin	TS-30mg/kg	TS-15mg/kg	Saline
d__Bacteria;p__Firmicutes;c__Clostridia;o__Lachnospirales;f__Lachnospiraceae;g__Lachnospiraceae_NK4A136_group	2050	140	22	164	433	0	264	285	366
d__Bacteria;p__Firmicutes;c__Clostridia;o__Oscillospirales;f__Oscillospiraceae;g__UCG-005	4514	251	211	702	791	387	732	503	489
d__Bacteria;p__Firmicutes;c__Clostridia;o__Lachnospirales;f__Lachnospiraceae;g__Blautia	976	74	120	372	276	27	88	269	130
d__Bacteria;p__Firmicutes;c__Clostridia;o__Lachnospirales;f__Lachnospiraceae;g__Lachnospiraceae_UCG-004	223	0	21	0	39	0	11	0	0
d__Bacteria;p__Actinobacteriota;c__Coriobacteriia;o__Coriobacteriales;f__Eggerthellaceae;__	429	399	54	278	165	238	53	432	95
d__Bacteria;p__Firmicutes;c__Clostridia;o__Clostridia;f__Hungateiclostridiaceae;g__Ruminiclostridium	174	21	14	15	54	0	0	69	0
d__Bacteria;p__Firmicutes;c__Clostridia;o__Oscillospirales;f__Ruminococcaceae;__	1642	13	57	60	0	81	38	76	0
d__Bacteria;p__Firmicutes;c__Clostridia;o__Lachnospirales;f__Lachnospiraceae;g__Lachnoclostridium	418	82	97	801	229	0	210	757	448
d__Bacteria;p__Bacteroidota;c__Bacteroidia;o__Bacteroidales;f__Tannerellaceae;g__Parabacteroides	727	32	69	109	60	135	40	176	141
d__Bacteria;p__Elusimicrobiota;c__Elusimicrobia;o__Elusimicrobiales;f__Elusimicrobiaceae;g__Elusimicrobium	1198	0	119	230	84	80	71	101	131
d__Bacteria;p__Firmicutes;c__Clostridia;o__Lachnospirales;f__Defluviitaleaceae;g__Defluviitaleaceae_UCG-011	253	49	108	10	80	85	62	191	69
d__Bacteria;p__Firmicutes;c__Bacilli;o__Erysipelotrichales;f__Erysipelotrichaceae;g__Dubosiella	350	0	0	0	0	20	0	1475	274
d__Bacteria;p__Firmicutes;c__Clostridia;o__Oscillospirales;f__Ruminococcaceae;g__Ruminococcaceae	105	55	34	0	14	92	41	0	258
d__Bacteria;p__Actinobacteriota;c__Actinobacteria;o__Bifidobacteriales;f__Bifidobacteriaceae;g__Bifidobacterium	4776	0	848	862	10	66	202	541	1612
d__Bacteria;p__Firmicutes;c__Clostridia;o__Lachnospirales;f__Lachnospiraceae;g__Lachnospiraceae_UCG-001	132	0	70	97	347	14	71	0	112
d__Bacteria;p__Desulfobacterota;c__Desulfovibrionia;o__Desulfovibrionales;f__Desulfovibrionaceae;g__Bilophila	0	0	0	0	0	0	0	115	0
d__Bacteria;p__Verrucomicrobiota;c__Verrucomicrobiae;o__Verrucomicrobiales;f__Akkermansiaceae;g__Akkermansia	0	59	2922	0	2001	132	0	39	2547
d__Bacteria;p__Firmicutes;c__Clostridia;o__Lachnospirales;f__Lachnospiraceae;g__Lachnospira	12	0	0	209	58	0	17	0	0
d__Bacteria;p__Bacteroidota;c__Bacteroidia;o__Bacteroidales;f__Muribaculaceae;g__Muribaculum	374	0	14	27	0	12	15	127	0
d__Bacteria;p__Actinobacteriota;c__Coriobacteriia;o__Coriobacteriales;f__Coriobacteriales_Incertae_Sedis;g__uncultured	187	28	15	51	0	78	45	180	106
d__Bacteria;p__Bacteroidota;c__Bacteroidia;o__Bacteroidales;f__Marinifilaceae;g__Odoribacter	92	0	0	0	0	0	0	106	0
d__Bacteria;p__Firmicutes;c__Clostridia;o__Lachnospirales;f__Lachnospiraceae;g__[Eubacterium]_ruminantium_group	18	0	62	169	17	328	625	694	272
d__Bacteria;p__Firmicutes;c__Clostridia;o__Peptostreptococcales-Tissierellales;f__Anaerovoracaceae;g__[Eubacterium]_brachy_group	130	58	0	69	16	15	0	152	33
d__Bacteria;p__Firmicutes;c__Clostridia;o__Oscillospirales;__;__	158	137	0	40	8	35	58	87	55
d__Bacteria;p__Firmicutes;c__Clostridia;o__Oscillospirales;f__Ruminococcaceae;g__UBA1819	25	0	0	11	0	0	0	0	0
d__Bacteria;p__Actinobacteriota;c__Coriobacteriia;o__Coriobacteriales;f__Eggerthellaceae;g__Adlercreutzia	65	0	0	49	66	0	25	0	0
d__Bacteria;p__Bacteroidota;c__Bacteroidia;o__Bacteroidales;f__Rikenellaceae;g__Alistipes	317	12	64	159	107	93	142	142	64
d__Bacteria;p__Firmicutes;c__Clostridia;o__Lachnospirales;f__Lachnospiraceae;g__Frisingicoccus	0	0	0	100	70	0	0	143	17
d__Bacteria;p__Actinobacteriota;c__Coriobacteriia;o__Coriobacteriales;f__Atopobiaceae;__	1713	414	818	401	522	190	484	1057	320
d__Bacteria;p__Firmicutes;c__Clostridia;o__Oscillospirales;f__Oscillospiraceae;g__Papillibacter	184	0	0	33	73	0	43	28	0
d__Bacteria;p__Bacteroidota;c__Bacteroidia;o__Bacteroidales;f__Rikenellaceae;g__dgA-11_gut_group	350	14	38	5	49	13	52	29	21
d__Bacteria;p__Proteobacteria;c__Gammaproteobacteria;o__Burkholderiales;f__Sutterellaceae;g__Parasutterella	312	68	85	142	131	24	37	104	112
d__Bacteria;p__Actinobacteriota;c__Coriobacteriia;o__Coriobacteriales;f__Coriobacteriaceae;g__Collinsella	0	0	0	0	0	0	0	0	13
d__Bacteria;p__Firmicutes;c__Clostridia;o__Christensenellales;f__Christensenellaceae;__	15	0	0	0	0	0	0	0	0
d__Archaea;p__Euryarchaeota;c__Methanobacteria;o__Methanobacteriales;f__Methanobacteriaceae;g__Methanobrevibacter	10846	3199	415	1722	3162	5971	671	1877	692
d__Bacteria;p__Firmicutes;c__Clostridia;o__Oscillospirales;f__Oscillospiraceae;g__Oscillibacter	114	0	0	30	40	16	36	23	50
d__Bacteria;p__Firmicutes;c__Clostridia;o__Peptostreptococcales-Tissierellales;f__Anaerovoracaceae;g__Mogibacterium	333	109	41	267	222	42	38	664	66
d__Bacteria;p__Fusobacteriota;c__Fusobacteriia;o__Fusobacteriales;f__Fusobacteriaceae;g__Fusobacterium	7	0	0	0	0	0	0	490	2
d__Bacteria;p__Firmicutes;c__Clostridia;o__Lachnospirales;f__Lachnospiraceae;g__Lachnospiraceae_UCG-010	249	0	0	153	29	0	0	30	0
d__Bacteria;p__Bacteroidota;c__Bacteroidia;o__Bacteroidales;f__Prevotellaceae;g__Prevotellaceae_NK3B31_group	697	61	9	47	30	211	82	8	58
d__Bacteria;p__Firmicutes;c__Clostridia;o__Peptostreptococcales-Tissierellales;f__Anaerovoracaceae;g__Family_XIII_AD3011_group	295	87	94	644	198	48	19	292	47
d__Bacteria;p__Firmicutes;c__Bacilli;o__Erysipelotrichales;f__Erysipelatoclostridiaceae;g__Erysipelatoclostridiaceae	49	9	0	70	34	0	0	20	0
d__Bacteria;p__Firmicutes;c__Clostridia;o__Lachnospirales;f__Lachnospiraceae;g__Dorea	356	14	15	176	39	0	0	26	0
d__Bacteria;p__Proteobacteria;c__Gammaproteobacteria;o__Enterobacterales;f__Enterobacteriaceae;g__Escherichia-Shigella	0	0	16	24	137	0	936	261	69
d__Bacteria;p__Actinobacteriota;c__Coriobacteriia;o__Coriobacteriales;f__Atopobiaceae;g__uncultured	152	72	40	76	40	19	22	26	22
d__Bacteria;p__Actinobacteriota;c__Actinobacteria;o__Actinomycetales;f__Actinomycetaceae;g__Actinomycetaceae	0	0	0	0	0	0	0	0	13
d__Bacteria;p__Spirochaetota;c__Spirochaetia;o__Spirochaetales;f__Spirochaetaceae;g__Treponema	6701	166	1164	456	332	1052	1275	279	76
d__Bacteria;p__Firmicutes;c__Clostridia;o__Oscillospirales;f__Ruminococcaceae;g__[Eubacterium]_siraeum_group	586	0	43	0	0	0	0	0	6
d__Bacteria;p__Firmicutes;c__Clostridia;o__Lachnospirales;f__Lachnospiraceae;g__[Ruminococcus]_torques_group	14	0	0	0	67	19	31	68	0
**Total Count/Treatment**	**180146**	**30734**	**38624**	**43203**	**41389**	**33433**	**31089**	**57853**	**36230**

Supplementary File 5
Relative Abundance Data

2DPT Fecal Genus Level Taxon Frequency	AC-PO7d	AC-10mg/kg	AC-5mg/kg	anti-rsPilA	anti-tip-chimer	Ofloxacin	TS-30mg/kg	TS-15mg/kg	Saline
d__Bacteria;p__Firmicutes;c__Clostridia;o__Oscillospirales;f__Oscillospiraceae;g__uncultured	93	27	216	976	187	302	748	1424	150
d__Bacteria;p__Firmicutes;c__Bacilli;o__Erysipelotrichales;f__Erysipelotrichaceae;__	405	4565	2824	4406	3061	2115	8361	6587	2879
d__Bacteria;p__Firmicutes;c__Clostridia;o__Christensenellales;f__Christensenellaceae;g__Christensenellaceae_R-7_group	0	325	491	2919	1579	1466	1293	4082	1941
d__Bacteria;p__Proteobacteria;c__Gammaproteobacteria;o__Pasteurellales;f__Pasteurellaceae;g__Haemophilus	664	327	1358	27	216	0	0	0	0
d__Bacteria;p__Bacteroidota;c__Bacteroidia;o__Bacteroidales;__;__	29	7	0	49	79	107	91	93	143
d__Bacteria;p__Actinobacteriota;c__Actinobacteria;o__Actinomycetales;f__Actinomycetaceae;g__Actinomyces	0	621	37	78	7	0	42	4	0
d__Bacteria;p__Patescibacteria;c__Saccharimonadia;o__Saccharimonadales;f__Saccharimonadaceae;g__Candidatus_Saccharimonas	135	413	715	872	1361	794	1653	175	494
d__Bacteria;p__Bacteroidota;c__Bacteroidia;o__Bacteroidales;f__Muribaculaceae;g__Muribaculaceae	143	683	1881	7005	6986	4498	4349	5036	5750
d__Bacteria;p__Firmicutes;c__Clostridia;o__Lachnospirales;f__Lachnospiraceae;__	22	374	1380	1714	3970	695	1419	1603	1306
d__Bacteria;p__Actinobacteriota;c__Coriobacteriia;o__Coriobacteriales;f__Eggerthellaceae;g__Enterorhabdus	56	559	475	378	319	165	439	166	334
d__Bacteria;p__Firmicutes;c__Clostridia;o__Clostridia_UCG-014;f__Clostridia_UCG-014;g__Clostridia_UCG-014	0	13	212	383	373	193	273	663	93
d__Bacteria;p__Actinobacteriota;c__Coriobacteriia;o__Coriobacteriales;f__uncultured;g__uncultured	0	77	47	6	42	0	58	60	53
d__Bacteria;p__Firmicutes;c__Bacilli;o__Erysipelotrichales;f__Erysipelotrichaceae;g__Allobaculum	0	117	2474	641	1412	678	467	2459	1203
d__Bacteria;p__Firmicutes;c__Clostridia;o__Oscillospirales;f__Ruminococcaceae;g__Incertae_Sedis	0	216	366	563	261	121	467	975	140
d__Bacteria;p__Firmicutes;c__Bacilli;o__Erysipelotrichales;f__Erysipelotrichaceae;g__uncultured	13	1375	3989	1690	1602	1120	768	284	1212
d__Bacteria;p__Bacteroidota;c__Bacteroidia;o__Bacteroidales;f__Marinifilaceae;g__Butyricimonas	102	0	0	76	0	22	0	58	28
d__Bacteria;p__Actinobacteriota;c__Coriobacteriia;o__Coriobacteriales;__;__	47	40	72	71	17	49	35	32	0
d__Bacteria;p__Firmicutes;c__Clostridia;o__Lachnospirales;f__Lachnospiraceae;g__Marvinbryantia	8	45	236	39	25	0	102	60	0
d__Bacteria;p__Firmicutes;c__Clostridia;o__Peptostreptococcales-Tissierellales;f__Anaerovoracaceae;g__[Eubacterium]_nodatum_group	0	0	109	98	26	30	0	19	0
d__Bacteria;p__Firmicutes;c__Clostridia;o__Oscillospirales;f__Oscillospiraceae;g__NK4A214_group	0	0	36	210	166	191	200	92	150
d__Bacteria;p__Bacteroidota;c__Bacteroidia;o__Bacteroidales;f__Rikenellaceae;g__Rikenellaceae_RC9_gut_group	0	0	0	0	141	0	137	0	164
d__Bacteria;p__Bacteroidota;c__Bacteroidia;o__Bacteroidales;f__Prevotellaceae;g__Prevotellaceae_UCG-001	0	37	68	83	1555	1519	1851	402	1583
d__Bacteria;p__Firmicutes;c__Clostridia;o__Oscillospirales;f__[Eubacterium]_coprostanoligenes_group;g__[Eubacterium]_coprostanoligenes_group	0	17	87	623	169	172	225	343	20
d__Bacteria;p__Bacteroidota;c__Bacteroidia;o__Bacteroidales;f__Prevotellaceae;g__Prevotella	0	34	197	104	570	449	1844	650	827
d__Bacteria;p__Planctomycetota;c__Planctomycetes;o__Pirellulales;f__Pirellulaceae;g__p-1088-a5_gut_group	12	133	37	32	21	79	276	10	12
d__Bacteria;p__Firmicutes;c__Clostridia;o__Oscillospirales;f__Ruminococcaceae;g__Ruminococcus	0	291	394	693	1255	502	1438	747	831
d__Bacteria;p__Bacteroidota;c__Bacteroidia;o__Bacteroidales;f__Prevotellaceae;g__Prevotellaceae_Ga6A1_group	0	0	0	0	115	46	234	54	296
d__Bacteria;p__Bacteroidota;c__Bacteroidia;o__Bacteroidales;f__Bacteroidaceae;g__Bacteroides	36133	1062	759	1627	419	473	531	420	401
d__Bacteria;p__Firmicutes;c__Bacilli;o__Erysipelotrichales;f__Erysipelotrichaceae;g__Ileibacterium	196	495	5130	1962	2156	2133	281	1650	1482
d__Bacteria;p__Firmicutes;c__Clostridia;o__Monoglobales;f__Monoglobaceae;g__Monoglobus	27	48	158	358	176	135	399	78	161
d__Bacteria;p__Bacteroidota;c__Bacteroidia;o__Bacteroidales;f__Rikenellaceae;g__Alistipes	187	38	7	63	79	0	147	168	107
d__Bacteria;p__Firmicutes;c__Clostridia;o__Lachnospirales;f__Lachnospiraceae;g__Coprococcus	0	16	322	457	25	38	91	33	8
d__Bacteria;p__Firmicutes;c__Bacilli;o__Lactobacillales;f__Lactobacillaceae;g__Lactobacillus	38	3051	210	538	0	452	20	164	267
d__Bacteria;p__Bacteroidota;c__Bacteroidia;o__Bacteroidales;f__Muribaculaceae;__	0	0	9	107	0	0	77	0	0
d__Bacteria;p__Firmicutes;c__Bacilli;o__Erysipelotrichales;f__Erysipelatoclostridiaceae;__	0	0	0	150	0	0	52	0	46
d__Bacteria;p__Firmicutes;c__Negativicutes;o__Acidaminococcales;f__Acidaminococcaceae;g__Phascolarctobacterium	0	105	95	161	335	99	169	95	195
d__Bacteria;p__Firmicutes;c__Clostridia;o__Lachnospirales;f__Lachnospiraceae;g__uncultured	0	0	0	0	0	0	5	130	36
d__Archaea;p__Euryarchaeota;c__Methanobacteria;o__Methanobacteriales;f__Methanobacteriaceae;g__Methanosphaera	9	492	376	0	0	304	110	77	13
d__Bacteria;p__Firmicutes;c__Clostridia;o__Oscillospirales;f__Butyricicoccaceae;g__UCG-008	20	242	0	179	84	78	0	0	29
d__Bacteria;p__Firmicutes;c__Bacilli;o__RF39;f__RF39;g__RF39	0	0	77	91	134	140	7	10	15
d__Bacteria;p__Firmicutes;c__Clostridia;o__Oscillospirales;f__Oscillospiraceae;g__Colidextribacter	18	0	0	99	98	41	291	113	211
d__Bacteria;p__Firmicutes;c__Clostridia;o__Eubacteriales;f__Eubacteriaceae;g__uncultured	21	477	1471	2648	2608	3426	1634	2169	4556
d__Bacteria;p__Firmicutes;c__Bacilli;o__Lactobacillales;f__Streptococcaceae;g__Streptococcus	43	90	157	551	78	35	499	128	458
d__Bacteria;p__Bacteroidota;c__Bacteroidia;o__Bacteroidales;f__Prevotellaceae;g__Prevotellaceae_UCG-003	0	0	0	6	36	56	0	0	0
d__Bacteria;p__Desulfobacterota;c__Desulfovibrionia;o__Desulfovibrionales;f__Desulfovibrionaceae;g__Desulfovibrio	0	0	0	0	14	0	0	0	44
d__Bacteria;p__Firmicutes;c__Clostridia;o__Oscillospirales;f__Oscillospiraceae;__	84	18	0	688	85	0	164	19	238
d__Bacteria;p__Firmicutes;c__Bacilli;o__Erysipelotrichales;f__Erysipelotrichaceae;g__Faecalibaculum	17	369	103	37	14	0	19	50	23

Supplementary File 5
Relative Abundance Data

2DPT Fecal Genus Level Taxon Frequency Continued	AC-PO7d	AC-10mg/kg	AC-5mg/kg	anti-rsPilA	anti-tip-chimer	Ofloxacin	TS-30mg/kg	TS-15mg/kg	Saline
d__Bacteria;p__Actinobacteriota;c__Coriobacteriia;o__Coriobacteriales;f__Eggerthellaceae;g__uncultured	8	55	200	1861	269	403	790	332	345
d__Bacteria;p__Firmicutes;c__Clostridia;o__Lachnospirales;f__Lachnospiraceae;g__Lachnospiraceae_NK4A136_group	0	47	0	134	185	0	377	534	274
d__Bacteria;p__Firmicutes;c__Clostridia;o__Oscillospirales;f__Oscillospiraceae;g__UCG-005	307	134	218	492	827	518	724	235	788
d__Bacteria;p__Firmicutes;c__Clostridia;o__Lachnospirales;f__Lachnospiraceae;g__Blautia	239	69	282	421	80	125	227	391	269
d__Bacteria;p__Firmicutes;c__Clostridia;o__Lachnospirales;f__Lachnospiraceae;g__Lachnospiraceae_UCG-004	0	0	0	11	20	28	0	28	27
d__Bacteria;p__Actinobacteriota;c__Coriobacteriia;o__Coriobacteriales;f__Eggerthellaceae;__	0	136	137	149	95	79	403	218	120
d__Bacteria;p__Firmicutes;c__Clostridia;o__Clostridia;f__Hungateiclostridiaceae;g__Ruminiclostridium	0	0	0	28	0	0	24	17	0
d__Bacteria;p__Firmicutes;c__Clostridia;o__Oscillospirales;f__Ruminococcaceae;__	0	0	27	20	23	24	138	28	0
d__Bacteria;p__Firmicutes;c__Clostridia;o__Lachnospirales;f__Lachnospiraceae;g__Lachnoclostridium	214	0	666	1474	32	449	746	720	363
d__Bacteria;p__Bacteroidota;c__Bacteroidia;o__Bacteroidales;f__Tannerellaceae;g__Parabacteroides	2176	336	645	439	60	66	178	90	102
d__Bacteria;p__Elusimicrobiota;c__Elusimicrobia;o__Elusimicrobiales;f__Elusimicrobiaceae;g__Elusimicrobium	0	22	11	23	34	26	166	3	30
d__Bacteria;p__Firmicutes;c__Clostridia;o__Lachnospirales;f__Defluviitaleaceae;g__Defluviitaleaceae_UCG-011	0	0	50	105	11	33	223	61	37
d__Bacteria;p__Firmicutes;c__Bacilli;o__Erysipelotrichales;f__Erysipelotrichaceae;g__Dubosiella	0	0	0	0	0	778	0	254	860
d__Bacteria;p__Firmicutes;c__Clostridia;o__Oscillospirales;f__Ruminococcaceae;g__Ruminococcaceae	0	0	0	0	0	0	71	0	0
d__Bacteria;p__Actinobacteriota;c__Actinobacteria;o__Bifidobacteriales;f__Bifidobacteriaceae;g__Bifidobacterium	9	131	804	671	36	553	201	1332	1336
d__Bacteria;p__Firmicutes;c__Clostridia;o__Lachnospirales;f__Lachnospiraceae;g__Lachnospiraceae_UCG-001	0	0	40	0	0	0	0	0	0
d__Bacteria;p__Actinobacteriota;c__Coriobacteriia;o__Coriobacteriales;f__Eggerthellaceae;g__Paraeggerthella	0	0	16	0	0	0	0	0	0
d__Bacteria;p__Desulfobacterota;c__Desulfovibrionia;o__Desulfovibrionales;f__Desulfovibrionaceae;g__Bilophila	2746	13	0	0	0	3	21	16	31
d__Bacteria;p__Verrucomicrobiota;c__Verrucomicrobiae;o__Verrucomicrobiales;f__Akkermansiaceae;g__Akkermansia	4	0	478	0	383	14	1306	0	94
d__Bacteria;p__Firmicutes;c__Clostridia;o__Lachnospirales;f__Lachnospiraceae;g__Lachnospira	0	0	0	61	0	17	0	9	0
d__Bacteria;p__Bacteroidota;c__Bacteroidia;o__Bacteroidales;f__Muribaculaceae;g__Muribaculum	0	0	0	27	0	0	0	0	0
d__Bacteria;p__Actinobacteriota;c__Coriobacteriia;o__Coriobacteriales;f__Coriobacteriales_Incertae_Sedis;g__uncultured	7	204	115	63	46	73	26	13	61
d__Bacteria;p__Bacteroidota;c__Bacteroidia;o__Bacteroidales;f__Marinifilaceae;g__Odoribacter	4252	0	0	0	3	0	0	0	0
d__Bacteria;p__Firmicutes;c__Clostridia;o__Lachnospirales;f__Lachnospiraceae;g__[Eubacterium]_ruminantium_group	0	526	36	170	0	183	317	250	260
d__Bacteria;p__Firmicutes;c__Clostridia;o__Peptostreptococcales-Tissierellales;f__Anaerovoracaceae;g__[Eubacterium]_brachy_group	11	19	20	65	0	16	24	30	17
d__Bacteria;p__Firmicutes;c__Clostridia;o__Oscillospirales;__;__	0	0	0	69	103	51	76	52	83
d__Bacteria;p__Firmicutes;c__Clostridia;o__Oscillospirales;f__Ruminococcaceae;g__UBA1819	116	24	7	232	0	0	0	50	16
d__Bacteria;p__Actinobacteriota;c__Coriobacteriia;o__Coriobacteriales;f__Eggerthellaceae;g__Adlercreutzia	0	0	0	48	46	31	49	0	0
d__Bacteria;p__Firmicutes;c__Clostridia;o__Lachnospirales;f__Lachnospiraceae;g__Frisingicoccus	0	0	0	516	0	21	33	231	21
d__Bacteria;p__Actinobacteriota;c__Coriobacteriia;o__Coriobacteriales;f__Atopobiaceae;__	47	356	1106	542	321	279	705	401	306
d__Bacteria;p__Firmicutes;c__Clostridia;o__Oscillospirales;f__Oscillospiraceae;g__Papillibacter	0	0	0	28	109	0	0	27	37
d__Bacteria;p__Bacteroidota;c__Bacteroidia;o__Bacteroidales;f__Rikenellaceae;g__dgA-11_gut_group	0	5	0	0	14	0	103	68	33
d__Bacteria;p__Proteobacteria;c__Gammaproteobacteria;o__Burkholderiales;f__Sutterellaceae;g__Parasutterella	92	194	88	276	81	88	45	91	141
d__Bacteria;p__Actinobacteriota;c__Coriobacteriia;o__Coriobacteriales;f__Coriobacteriaceae;g__Collinsella	11	176	0	0	0	0	0	0	26
d__Bacteria;p__Firmicutes;c__Clostridia;o__Christensenellales;f__Christensenellaceae;__	182	369	0	0	0	0	0	49	0
d__Archaea;p__Euryarchaeota;c__Methanobacteria;o__Methanobacteriales;f__Methanobacteriaceae;g__Methanobrevibacter	6693	8355	4049	3923	3571	3470	1697	2442	97
d__Bacteria;p__Firmicutes;c__Clostridia;o__Oscillospirales;f__Oscillospiraceae;g__Oscillibacter	0	0	0	38	54	0	49	8	15
d__Bacteria;p__Firmicutes;c__Clostridia;o__Peptostreptococcales-Tissierellales;f__Anaerovoracaceae;g__Mogibacterium	0	240	235	184	110	111	117	267	34
d__Bacteria;p__Fusobacteriota;c__Fusobacteriia;o__Fusobacteriales;f__Fusobacteriaceae;g__Fusobacterium	34701	1002	0	0	0	9	0	0	10
d__Bacteria;p__Firmicutes;c__Clostridia;o__Lachnospirales;f__Lachnospiraceae;g__Lachnospiraceae_UCG-010	0	0	0	182	20	30	0	131	0
d__Bacteria;p__Bacteroidota;c__Bacteroidia;o__Bacteroidales;f__Prevotellaceae;g__Prevotellaceae_NK3B31_group	0	32	0	11	74	112	20	37	87
d__Bacteria;p__Firmicutes;c__Clostridia;o__Peptostreptococcales-Tissierellales;f__Anaerovoracaceae;g__Family_XIII_AD3011_group	61	104	314	458	181	188	40	27	194
d__Bacteria;p__Firmicutes;c__Bacilli;o__Erysipelotrichales;f__Erysipelatoclostridiaceae;g__Erysipelatoclostridiaceae	0	0	0	98	44	12	49	34	0
d__Bacteria;p__Firmicutes;c__Clostridia;o__Lachnospirales;f__Lachnospiraceae;g__Dorea	0	0	39	36	0	0	272	58	0
d__Bacteria;p__Proteobacteria;c__Gammaproteobacteria;o__Enterobacterales;f__Enterobacteriaceae;g__Escherichia-Shigella	8679	643	6	0	69	211	0	0	27
d__Bacteria;p__Actinobacteriota;c__Coriobacteriia;o__Coriobacteriales;f__Atopobiaceae;g__uncultured	0	33	86	59	28	30	30	20	85
d__Bacteria;p__Actinobacteriota;c__Actinobacteria;o__Actinomycetales;f__Actinomycetaceae;g__Actinomycetaceae	113	67	0	0	0	0	0	0	14
d__Bacteria;p__Spirochaetota;c__Spirochaetia;o__Spirochaetales;f__Spirochaetaceae;g__Treponema	0	12	34	40	213	586	21	419	76
d__Bacteria;p__Firmicutes;c__Clostridia;o__Oscillospirales;f__Ruminococcaceae;g__[Eubacterium]_siraeum_group	0	0	0	0	124	0	13	0	0
d__Bacteria;p__Firmicutes;c__Clostridia;o__Lachnospirales;f__Lachnospiraceae;g__[Ruminococcus]_torques_group	0	0	24	0	51	30	45	80	0
**Total Count/Treatment**	**99190**	**30103**	**36308**	**46412**	**39173**	**31370**	**40592**	**40435**	**34215**

Supplementary File 5
Relative Abundance Data

5DPT Fecal Genus Level Taxon Frequency	AC-PO7d	AC-10mg/kg	AC-5mg/kg	anti-rsPilA	anti-tip-chimer	Ofloxacin	TS-30mg/kg	TS-15mg/kg	Saline
d__Bacteria;p__Firmicutes;c__Clostridia;o__Oscillospirales;f__Oscillospiraceae;g__uncultured	2027	26	100	316	475	327	568	542	266
d__Bacteria;p__Firmicutes;c__Bacilli;o__Erysipelotrichales;f__Erysipelotrichaceae;__	1713	0	64	2175	2296	3715	8731	7103	5527
d__Bacteria;p__Firmicutes;c__Clostridia;o__Christensenellales;f__Christensenellaceae;g__Christensenellaceae_R-7_group	701	15	139	990	986	1915	1054	2529	1846
d__Bacteria;p__Proteobacteria;c__Gammaproteobacteria;o__Pasteurellales;f__Pasteurellaceae;g__Haemophilus	0	1024	1211	4	57	0	0	0	0
d__Bacteria;p__Bacteroidota;c__Bacteroidia;o__Bacteroidales;__;__	98	0	7	98	46	73	55	159	207
d__Bacteria;p__Actinobacteriota;c__Actinobacteria;o__Actinomycetales;f__Actinomycetaceae;g__Actinomyces	0	0	0	27	0	0	19	23	79
d__Bacteria;p__Patescibacteria;c__Saccharimonadia;o__Saccharimonadales;f__Saccharimonadaceae;g__Candidatus_Saccharimonas	500	0	73	1683	1133	1721	3560	1113	1143
d__Bacteria;p__Bacteroidota;c__Bacteroidia;o__Bacteroidales;f__Muribaculaceae;g__Muribaculaceae	10400	439	2201	4784	6527	5491	4542	5932	10136
d__Bacteria;p__Firmicutes;c__Clostridia;o__Lachnospirales;f__Lachnospiraceae;__	6843	1088	1888	487	3417	668	1111	1854	2907
d__Bacteria;p__Actinobacteriota;c__Coriobacteriia;o__Coriobacteriales;f__Eggerthellaceae;g__Enterorhabdus	156	0	12	167	177	53	414	252	549
d__Bacteria;p__Actinobacteriota;c__Coriobacteriia;o__Coriobacteriales;f__uncultured;g__uncultured	29	10	19	0	12	8	135	149	22
d__Bacteria;p__Firmicutes;c__Bacilli;o__Erysipelotrichales;f__Erysipelotrichaceae;g__Allobaculum	72	0	136	194	1768	413	373	2112	1138
d__Bacteria;p__Firmicutes;c__Clostridia;o__Oscillospirales;f__Ruminococcaceae;g__Incertae_Sedis	346	0	0	612	583	180	652	1198	189
d__Bacteria;p__Firmicutes;c__Bacilli;o__Erysipelotrichales;f__Erysipelotrichaceae;g__uncultured	368	0	13	223	1555	364	792	280	1377
d__Bacteria;p__Bacteroidota;c__Bacteroidia;o__Bacteroidales;f__Marinifilaceae;g__Butyricimonas	74	130	226	6	17	99	0	19	56
d__Bacteria;p__Actinobacteriota;c__Coriobacteriia;o__Coriobacteriales;__;__	30	0	15	105	27	54	29	26	23
d__Bacteria;p__Firmicutes;c__Clostridia;o__Lachnospirales;f__Lachnospiraceae;g__Marvinbryantia	287	0	31	0	0	0	26	0	55
d__Bacteria;p__Firmicutes;c__Clostridia;o__Peptostreptococcales-Tissierellales;f__Anaerovoracaceae;g__[Eubacterium]_nodatum_group	69	0	85	33	66	91	0	41	44
d__Bacteria;p__Firmicutes;c__Clostridia;o__Oscillospirales;f__Oscillospiraceae;g__NK4A214_group	20	0	0	96	189	44	71	71	223
d__Bacteria;p__Bacteroidota;c__Bacteroidia;o__Bacteroidales;f__Rikenellaceae;g__Rikenellaceae_RC9_gut_group	112	0	0	0	35	0	80	0	261
d__Bacteria;p__Bacteroidota;c__Bacteroidia;o__Bacteroidales;f__Prevotellaceae;g__Prevotellaceae_UCG-001	207	0	0	111	1207	1075	1929	148	1533
d__Bacteria;p__Firmicutes;c__Clostridia;o__Oscillospirales;f__[Eubacterium]_coprostanoligenes_group;g__[Eubacterium]_coprostanoligenes_group	41	13	12	152	42	340	210	178	152
d__Bacteria;p__Bacteroidota;c__Bacteroidia;o__Bacteroidales;f__Prevotellaceae;g__Prevotella	1236	0	0	255	1154	904	2321	114	351
d__Bacteria;p__Planctomycetota;c__Planctomycetes;o__Pirellulales;f__Pirellulaceae;g__p-1088-a5_gut_group	64	9	49	7	32	35	89	43	17
d__Bacteria;p__Firmicutes;c__Clostridia;o__Oscillospirales;f__Ruminococcaceae;g__Ruminococcus	1593	313	643	387	1151	1862	1177	278	851
d__Bacteria;p__Bacteroidota;c__Bacteroidia;o__Bacteroidales;f__Prevotellaceae;g__Prevotellaceae_Ga6A1_group	87	0	0	5	58	74	70	33	203
d__Bacteria;p__Bacteroidota;c__Bacteroidia;o__Bacteroidales;f__Bacteroidaceae;g__Bacteroides	7703	3152	5341	149	423	670	422	539	451
d__Bacteria;p__Firmicutes;c__Bacilli;o__Erysipelotrichales;f__Erysipelotrichaceae;g__Ileibacterium	158	4	422	699	2359	1798	355	1009	1419
d__Bacteria;p__Firmicutes;c__Clostridia;o__Monoglobales;f__Monoglobaceae;g__Monoglobus	215	140	0	234	164	512	655	139	135
d__Bacteria;p__Bacteroidota;c__Bacteroidia;o__Bacteroidales;f__Rikenellaceae;g__Alistipes	627	113	58	34	85	0	94	231	135
d__Bacteria;p__Firmicutes;c__Clostridia;o__Lachnospirales;f__Lachnospiraceae;g__Coprococcus	185	11	35	154	0	30	46	20	24
d__Bacteria;p__Firmicutes;c__Bacilli;o__Lactobacillales;f__Lactobacillaceae;g__Lactobacillus	548	82	91	472	31	0	24	121	687
d__Bacteria;p__Bacteroidota;c__Bacteroidia;o__Bacteroidales;f__Muribaculaceae;__	0	0	0	29	0	0	66	0	0
d__Bacteria;p__Firmicutes;c__Bacilli;o__Erysipelotrichales;f__Erysipelatoclostridiaceae;__	60	0	155	29	0	0	57	0	44
d__Bacteria;p__Firmicutes;c__Negativicutes;o__Acidaminococcales;f__Acidaminococcaceae;g__Phascolarctobacterium	80	0	26	229	301	276	191	289	234
d__Bacteria;p__Firmicutes;c__Clostridia;o__Lachnospirales;f__Lachnospiraceae;g__uncultured	25	0	0	14	96	38	44	52	33
d__Archaea;p__Euryarchaeota;c__Methanobacteria;o__Methanobacteriales;f__Methanobacteriaceae;g__Methanosphaera	48	4	115	0	0	9	135	119	79
d__Bacteria;p__Firmicutes;c__Clostridia;o__Oscillospirales;f__Butyricicoccaceae;g__UCG-008	176	0	6	74	39	51	20	0	93
d__Bacteria;p__Firmicutes;c__Bacilli;o__RF39;f__RF39;g__RF39	71	0	0	313	111	192	33	112	148
d__Bacteria;p__Firmicutes;c__Clostridia;o__Oscillospirales;f__Oscillospiraceae;g__Colidextribacter	240	0	142	0	167	120	233	65	123
d__Bacteria;p__Firmicutes;c__Clostridia;o__Eubacteriales;f__Eubacteriaceae;g__uncultured	436	0	4	663	3660	1749	1676	2241	4740
d__Bacteria;p__Firmicutes;c__Bacilli;o__Lactobacillales;f__Streptococcaceae;g__Streptococcus	58	46	14	900	87	14	455	127	359
d__Bacteria;p__Bacteroidota;c__Bacteroidia;o__Bacteroidales;f__Prevotellaceae;g__Prevotellaceae_UCG-003	0	0	0	151	13	51	0	0	0
d__Bacteria;p__Desulfobacterota;c__Desulfovibrionia;o__Desulfovibrionales;f__Desulfovibrionaceae;g__Desulfovibrio	0	0	0	12	40	0	0	0	142
d__Bacteria;p__Firmicutes;c__Clostridia;o__Oscillospirales;f__Oscillospiraceae;__	138	0	80	102	285	43	15	91	288
d__Bacteria;p__Firmicutes;c__Bacilli;o__Erysipelotrichales;f__Erysipelotrichaceae;g__Faecalibaculum	0	33	305	0	0	0	41	28	20
d__Bacteria;p__Actinobacteriota;c__Coriobacteriia;o__Coriobacteriales;f__Eggerthellaceae;g__uncultured	0	0	154	84	130	724	372	723	708

Supplementary File 5
Relative Abundance Data

5DPT Fecal Genus Level Taxon Frequency Continued	AC-PO7d	AC-10mg/kg	AC-5mg/kg	anti-rsPilA	anti-tip-chimer	Ofloxacin	TS-30mg/kg	TS-15mg/kg	Saline
d__Bacteria;p__Firmicutes;c__Clostridia;o__Lachnospirales;f__Lachnospiraceae;g__Lachnospiraceae_NK4A136_group	1157	0	310	127	351	85	438	272	249
d__Bacteria;p__Firmicutes;c__Clostridia;o__Oscillospirales;f__Oscillospiraceae;g__UCG-005	1505	154	271	382	518	490	706	282	1147
d__Bacteria;p__Firmicutes;c__Clostridia;o__Lachnospirales;f__Lachnospiraceae;g__Blautia	116	90	566	117	150	375	151	554	414
d__Bacteria;p__Firmicutes;c__Clostridia;o__Lachnospirales;f__Lachnospiraceae;g__Lachnospiraceae_UCG-004	28	22	100	26	0	0	17	0	14
d__Bacteria;p__Actinobacteriota;c__Coriobacteriia;o__Coriobacteriales;f__Eggerthellaceae;__	0	0	33	72	92	95	285	362	129
d__Bacteria;p__Firmicutes;c__Clostridia;o__Clostridia;f__Hungateiclostridiaceae;g__Ruminiclostridium	72	18	16	51	27	25	0	16	0
d__Bacteria;p__Firmicutes;c__Clostridia;o__Clostridia_UCG-014;f__Clostridia_UCG-014;g__Clostridia_UCG-014	195	0	72	111	345	582	125	214	607
d__Bacteria;p__Firmicutes;c__Clostridia;o__Oscillospirales;f__Ruminococcaceae;__	447	0	0	31	15	20	147	34	0
d__Bacteria;p__Firmicutes;c__Clostridia;o__Lachnospirales;f__Lachnospiraceae;g__Lachnoclostridium	389	344	1073	294	191	693	414	652	886
d__Bacteria;p__Bacteroidota;c__Bacteroidia;o__Bacteroidales;f__Tannerellaceae;g__Parabacteroides	911	303	1030	82	42	79	182	68	176
d__Bacteria;p__Elusimicrobiota;c__Elusimicrobia;o__Elusimicrobiales;f__Elusimicrobiaceae;g__Elusimicrobium	30	0	68	221	19	0	21	33	115
d__Bacteria;p__Firmicutes;c__Clostridia;o__Lachnospirales;f__Defluviitaleaceae;g__Defluviitaleaceae_UCG-011	85	0	16	0	30	0	187	46	53
d__Bacteria;p__Firmicutes;c__Bacilli;o__Erysipelotrichales;f__Erysipelotrichaceae;g__Dubosiella	123	0	0	0	0	166	0	154	712
d__Bacteria;p__Firmicutes;c__Clostridia;o__Oscillospirales;f__Ruminococcaceae;g__Ruminococcaceae	18	0	0	0	58	33	67	0	0
d__Bacteria;p__Actinobacteriota;c__Actinobacteria;o__Bifidobacteriales;f__Bifidobacteriaceae;g__Bifidobacterium	645	0	24	199	118	5	691	1044	1600
d__Bacteria;p__Firmicutes;c__Clostridia;o__Lachnospirales;f__Lachnospiraceae;g__Lachnospiraceae_UCG-001	0	0	0	0	50	24	22	0	0
d__Bacteria;p__Actinobacteriota;c__Coriobacteriia;o__Coriobacteriales;f__Eggerthellaceae;g__Paraeggerthella	0	668	1991	0	0	0	0	100	0
d__Bacteria;p__Desulfobacterota;c__Desulfovibrionia;o__Desulfovibrionales;f__Desulfovibrionaceae;g__Bilophila	381	0	1391	0	0	0	16	8	110
d__Bacteria;p__Verrucomicrobiota;c__Verrucomicrobiae;o__Verrucomicrobiales;f__Akkermansiaceae;g__Akkermansia	0	4821	0	0	225	0	3145	0	812
d__Bacteria;p__Bacteroidota;c__Bacteroidia;o__Bacteroidales;f__Muribaculaceae;g__Muribaculum	9	66	0	19	0	36	0	44	102
d__Bacteria;p__Actinobacteriota;c__Coriobacteriia;o__Coriobacteriales;f__Coriobacteriales_Incertae_Sedis;g__uncultured	88	0	16	30	30	28	52	41	72
d__Bacteria;p__Bacteroidota;c__Bacteroidia;o__Bacteroidales;f__Marinifilaceae;g__Odoribacter	217	72	570	0	22	4	0	17	0
d__Bacteria;p__Firmicutes;c__Clostridia;o__Lachnospirales;f__Lachnospiraceae;g__[Eubacterium]_ruminantium_group	0	0	0	0	0	310	324	371	112
d__Bacteria;p__Firmicutes;c__Clostridia;o__Peptostreptococcales-Tissierellales;f__Anaerovoracaceae;g__[Eubacterium]_brachy_group	88	0	0	32	0	0	29	48	15
d__Bacteria;p__Firmicutes;c__Clostridia;o__Oscillospirales;__;__	0	0	0	34	50	0	29	29	76
d__Bacteria;p__Firmicutes;c__Clostridia;o__Oscillospirales;f__Ruminococcaceae;g__UBA1819	1085	930	305	0	0	0	0	91	14
d__Bacteria;p__Actinobacteriota;c__Coriobacteriia;o__Coriobacteriales;f__Eggerthellaceae;g__Adlercreutzia	338	0	25	0	0	54	0	0	23
d__Bacteria;p__Firmicutes;c__Clostridia;o__Lachnospirales;f__Lachnospiraceae;g__Frisingicoccus	0	23	0	35	0	500	0	479	33
d__Bacteria;p__Actinobacteriota;c__Coriobacteriia;o__Coriobacteriales;f__Atopobiaceae;__	107	0	36	483	321	498	813	206	812
d__Bacteria;p__Firmicutes;c__Clostridia;o__Oscillospirales;f__Oscillospiraceae;g__Papillibacter	79	0	0	0	0	48	0	0	36
d__Bacteria;p__Bacteroidota;c__Bacteroidia;o__Bacteroidales;f__Rikenellaceae;g__dgA-11_gut_group	62	0	0	47	25	30	47	27	41
d__Bacteria;p__Proteobacteria;c__Gammaproteobacteria;o__Burkholderiales;f__Sutterellaceae;g__Parasutterella	129	10	197	31	75	56	62	91	216
d__Bacteria;p__Actinobacteriota;c__Coriobacteriia;o__Coriobacteriales;f__Coriobacteriaceae;g__Collinsella	297	442	87	0	0	0	0	0	35
d__Bacteria;p__Firmicutes;c__Clostridia;o__Christensenellales;f__Christensenellaceae;__	32	27	23	0	0	0	0	88	0
d__Archaea;p__Euryarchaeota;c__Methanobacteria;o__Methanobacteriales;f__Methanobacteriaceae;g__Methanobrevibacter	1484	17	3906	2910	1709	4416	2000	3771	117
d__Bacteria;p__Firmicutes;c__Clostridia;o__Oscillospirales;f__Oscillospiraceae;g__Oscillibacter	35	0	0	0	10	0	84	0	0
d__Bacteria;p__Firmicutes;c__Clostridia;o__Peptostreptococcales-Tissierellales;f__Anaerovoracaceae;g__Mogibacterium	0	0	54	120	77	11	107	220	116
d__Bacteria;p__Fusobacteriota;c__Fusobacteriia;o__Fusobacteriales;f__Fusobacteriaceae;g__Fusobacterium	9866	1892	7602	0	0	0	0	39	7
d__Bacteria;p__Firmicutes;c__Clostridia;o__Lachnospirales;f__Lachnospiraceae;g__Lachnospiraceae_UCG-010	33	28	0	0	0	17	0	61	0
d__Bacteria;p__Bacteroidota;c__Bacteroidia;o__Bacteroidales;f__Prevotellaceae;g__Prevotellaceae_NK3B31_group	23	0	326	221	145	132	49	39	106
d__Bacteria;p__Firmicutes;c__Clostridia;o__Peptostreptococcales-Tissierellales;f__Anaerovoracaceae;g__Family_XIII_AD3011_group	85	7	177	138	102	122	37	21	203
d__Bacteria;p__Firmicutes;c__Bacilli;o__Erysipelotrichales;f__Erysipelatoclostridiaceae;g__Erysipelatoclostridiaceae	42	422	448	0	17	74	6	38	9
d__Bacteria;p__Firmicutes;c__Clostridia;o__Lachnospirales;f__Lachnospiraceae;g__Dorea	0	41	0	0	0	36	187	58	48
d__Bacteria;p__Proteobacteria;c__Gammaproteobacteria;o__Enterobacterales;f__Enterobacteriaceae;g__Escherichia-Shigella	2350	104	1751	0	203	0	10	184	49
d__Bacteria;p__Actinobacteriota;c__Coriobacteriia;o__Coriobacteriales;f__Atopobiaceae;g__uncultured	35	8	10	16	0	16	36	45	114
d__Bacteria;p__Actinobacteriota;c__Actinobacteria;o__Actinomycetales;f__Actinomycetaceae;g__Actinomycetaceae	0	803	226	0	0	0	0	0	11
d__Bacteria;p__Spirochaetota;c__Spirochaetia;o__Spirochaetales;f__Spirochaetaceae;g__Treponema	663	0	0	164	37	591	592	1419	7
d__Bacteria;p__Firmicutes;c__Clostridia;o__Oscillospirales;f__Ruminococcaceae;g__[Eubacterium]_siraeum_group	80	0	0	0	89	0	0	0	0
d__Bacteria;p__Firmicutes;c__Clostridia;o__Lachnospirales;f__Lachnospiraceae;g__[Ruminococcus]_torques_group	0	0	0	20	0	0	44	45	0
**Total Count/Treatment**	**61170**	**17964**	**36592**	**23272**	**36144**	**35434**	**44064**	**41414**	**48415**

Supplementary File 5
Relative Abundance Data

7DPT Fecal Genus Level Taxon Frequency	AC-PO7d	AC-10mg/kg	AC-5mg/kg	anti-rsPilA	anti-tip-chimer	Ofloxacin	TS-30mg/kg	TS-15mg/kg	Saline
d__Bacteria;p__Firmicutes;c__Clostridia;o__Oscillospirales;f__Oscillospiraceae;g__uncultured	1941	152	443	300	212	244	321	103	155
d__Bacteria;p__Firmicutes;c__Bacilli;o__Erysipelotrichales;f__Erysipelotrichaceae;__	49	0	4	2238	2967	2782	8145	6753	1916
d__Bacteria;p__Firmicutes;c__Clostridia;o__Christensenellales;f__Christensenellaceae;g__Christensenellaceae_R-7_group	735	419	625	965	670	1795	513	1934	416
d__Bacteria;p__Proteobacteria;c__Gammaproteobacteria;o__Pasteurellales;f__Pasteurellaceae;g__Haemophilus	0	0	0	0	31	91	0	0	1159
d__Bacteria;p__Bacteroidota;c__Bacteroidia;o__Bacteroidales;__;__	62	0	9	73	49	38	94	126	368
d__Bacteria;p__Actinobacteriota;c__Actinobacteria;o__Actinomycetales;f__Actinomycetaceae;g__Actinomyces	0	191	0	41	29	0	37	0	9
d__Bacteria;p__Patescibacteria;c__Saccharimonadia;o__Saccharimonadales;f__Saccharimonadaceae;g__Candidatus_Saccharimonas	19	0	9	1483	3934	590	7402	604	181
d__Bacteria;p__Bacteroidota;c__Bacteroidia;o__Bacteroidales;f__Muribaculaceae;g__Muribaculaceae	10133	712	5047	5833	7280	6174	9361	6827	4182
d__Bacteria;p__Firmicutes;c__Clostridia;o__Lachnospirales;f__Lachnospiraceae;__	5156	1023	4431	1129	1018	1893	1568	2575	839
d__Bacteria;p__Actinobacteriota;c__Coriobacteriia;o__Coriobacteriales;f__Eggerthellaceae;g__Enterorhabdus	24	31	224	306	328	56	631	94	166
d__Bacteria;p__Firmicutes;c__Clostridia;o__Clostridia_UCG-014;f__Clostridia_UCG-014;g__Clostridia_UCG-014	29	0	71	730	102	632	537	134	167
d__Bacteria;p__Actinobacteriota;c__Coriobacteriia;o__Coriobacteriales;f__uncultured;g__uncultured	64	227	185	0	50	10	151	93	69
d__Bacteria;p__Firmicutes;c__Bacilli;o__Erysipelotrichales;f__Erysipelotrichaceae;g__Allobaculum	0	0	48	613	1720	547	607	524	171
d__Bacteria;p__Firmicutes;c__Clostridia;o__Oscillospirales;f__Ruminococcaceae;g__Incertae_Sedis	0	94	32	795	419	161	681	488	101
d__Bacteria;p__Firmicutes;c__Bacilli;o__Erysipelotrichales;f__Erysipelotrichaceae;g__uncultured	72	0	60	744	1301	306	452	362	499
d__Bacteria;p__Bacteroidota;c__Bacteroidia;o__Bacteroidales;f__Marinifilaceae;g__Butyricimonas	0	0	77	0	11	244	0	38	66
d__Bacteria;p__Actinobacteriota;c__Coriobacteriia;o__Coriobacteriales;__;__	0	0	0	224	94	27	41	10	0
d__Bacteria;p__Firmicutes;c__Clostridia;o__Lachnospirales;f__Lachnospiraceae;g__Marvinbryantia	500	0	86	63	0	0	47	0	72
d__Bacteria;p__Firmicutes;c__Clostridia;o__Peptostreptococcales-Tissierellales;f__Anaerovoracaceae;g__[Eubacterium]_nodatum_group	36	0	35	131	75	92	0	49	0
d__Bacteria;p__Firmicutes;c__Clostridia;o__Oscillospirales;f__Oscillospiraceae;g__NK4A214_group	0	0	61	178	83	42	50	85	125
d__Bacteria;p__Bacteroidota;c__Bacteroidia;o__Bacteroidales;f__Rikenellaceae;g__Rikenellaceae_RC9_gut_group	0	0	0	0	4	31	169	0	30
d__Bacteria;p__Bacteroidota;c__Bacteroidia;o__Bacteroidales;f__Prevotellaceae;g__Prevotellaceae_UCG-001	78	0	0	130	278	1064	950	702	128
d__Bacteria;p__Firmicutes;c__Clostridia;o__Oscillospirales;f__[Eubacterium]_coprostanoligenes_group;g__[Eubacterium]_coprostanoligenes_group	137	87	301	176	10	539	249	119	51
d__Bacteria;p__Bacteroidota;c__Bacteroidia;o__Bacteroidales;f__Prevotellaceae;g__Prevotella	419	0	301	285	557	625	729	64	133
d__Bacteria;p__Planctomycetota;c__Planctomycetes;o__Pirellulales;f__Pirellulaceae;g__p-1088-a5_gut_group	146	170	45	44	129	92	69	49	13
d__Bacteria;p__Firmicutes;c__Clostridia;o__Oscillospirales;f__Ruminococcaceae;g__Ruminococcus	371	335	1150	461	314	1589	1212	917	338
d__Bacteria;p__Bacteroidota;c__Bacteroidia;o__Bacteroidales;f__Prevotellaceae;g__Prevotellaceae_Ga6A1_group	0	0	0	32	77	102	129	0	48
d__Bacteria;p__Bacteroidota;c__Bacteroidia;o__Bacteroidales;f__Bacteroidaceae;g__Bacteroides	10263	649	4546	177	152	1321	421	936	567
d__Bacteria;p__Firmicutes;c__Bacilli;o__Erysipelotrichales;f__Erysipelotrichaceae;g__Ileibacterium	10	73	125	1294	1321	2138	1243	1053	504
d__Bacteria;p__Firmicutes;c__Clostridia;o__Monoglobales;f__Monoglobaceae;g__Monoglobus	38	481	214	275	222	291	472	148	216
d__Bacteria;p__Bacteroidota;c__Bacteroidia;o__Bacteroidales;f__Rikenellaceae;g__Alistipes	2576	129	211	29	31	0	93	32	180
d__Bacteria;p__Firmicutes;c__Clostridia;o__Lachnospirales;f__Lachnospiraceae;g__Coprococcus	313	171	149	249	0	130	0	11	20
d__Bacteria;p__Firmicutes;c__Bacilli;o__Lactobacillales;f__Lactobacillaceae;g__Lactobacillus	0	742	0	560	36	7	41	82	9
d__Bacteria;p__Bacteroidota;c__Bacteroidia;o__Bacteroidales;f__Muribaculaceae;__	0	0	0	140	0	0	65	0	0
d__Bacteria;p__Firmicutes;c__Bacilli;o__Erysipelotrichales;f__Erysipelatoclostridiaceae;__	0	0	176	55	0	21	0	0	14
d__Bacteria;p__Firmicutes;c__Negativicutes;o__Acidaminococcales;f__Acidaminococcaceae;g__Phascolarctobacterium	0	264	125	181	888	126	181	197	96
d__Bacteria;p__Firmicutes;c__Clostridia;o__Lachnospirales;f__Lachnospiraceae;g__uncultured	0	0	0	40	0	35	83	0	0
d__Archaea;p__Euryarchaeota;c__Methanobacteria;o__Methanobacteriales;f__Methanobacteriaceae;g__Methanosphaera	16	3	67	0	0	0	22	53	3
d__Bacteria;p__Firmicutes;c__Clostridia;o__Oscillospirales;f__Butyricicoccaceae;g__UCG-008	127	41	0	174	69	0	54	0	19
d__Bacteria;p__Firmicutes;c__Bacilli;o__RF39;f__RF39;g__RF39	0	0	0	56	58	139	136	13	78
d__Bacteria;p__Firmicutes;c__Clostridia;o__Oscillospirales;f__Oscillospiraceae;g__Colidextribacter	111	116	368	56	202	0	77	33	0
d__Bacteria;p__Firmicutes;c__Clostridia;o__Eubacteriales;f__Eubacteriaceae;g__uncultured	0	0	39	893	2957	1553	1491	2077	1256
d__Bacteria;p__Firmicutes;c__Bacilli;o__Lactobacillales;f__Streptococcaceae;g__Streptococcus	0	238	0	426	108	46	400	101	38
d__Bacteria;p__Bacteroidota;c__Bacteroidia;o__Bacteroidales;f__Prevotellaceae;g__Prevotellaceae_UCG-003	0	0	0	9	33	72	0	0	0
d__Bacteria;p__Desulfobacterota;c__Desulfovibrionia;o__Desulfovibrionales;f__Desulfovibrionaceae;g__Desulfovibrio	0	0	0	0	21	0	6	0	66
d__Bacteria;p__Firmicutes;c__Clostridia;o__Oscillospirales;f__Oscillospiraceae;__	542	75	77	149	81	134	205	104	249
d__Bacteria;p__Firmicutes;c__Bacilli;o__Erysipelotrichales;f__Erysipelotrichaceae;g__Faecalibaculum	46	8	404	28	0	0	67	34	0

7DPT Fecal Genus Level Taxon Frequency Continued	AC-PO7d	AC-10mg/kg	AC-5mg/kg	anti-rsPilA	anti-tip-chimer	Ofloxacin	TS-30mg/kg	TS-15mg/kg	Saline
d__Bacteria;p__Actinobacteriota;c__Coriobacteriia;o__Coriobacteriales;f__Eggerthellaceae;g__uncultured	68	52	703	211	133	502	226	632	295
d__Bacteria;p__Firmicutes;c__Clostridia;o__Lachnospirales;f__Lachnospiraceae;g__Lachnospiraceae_NK4A136_group	1059	9	615	55	132	165	229	157	46
d__Bacteria;p__Firmicutes;c__Clostridia;o__Oscillospirales;f__Oscillospiraceae;g__UCG-005	541	72	601	695	204	608	429	385	1443
d__Bacteria;p__Firmicutes;c__Clostridia;o__Lachnospirales;f__Lachnospiraceae;g__Blautia	476	161	1311	257	18	510	156	381	255
d__Bacteria;p__Firmicutes;c__Clostridia;o__Lachnospirales;f__Lachnospiraceae;g__Lachnospiraceae_UCG-004	84	0	73	0	0	0	0	0	14
d__Bacteria;p__Actinobacteriota;c__Coriobacteriia;o__Coriobacteriales;f__Eggerthellaceae;__	0	11	56	121	190	43	469	205	52
d__Bacteria;p__Firmicutes;c__Clostridia;o__Clostridia;f__Hungateiclostridiaceae;g__Ruminiclostridium	91	27	78	19	9	0	8	8	0
d__Bacteria;p__Firmicutes;c__Clostridia;o__Oscillospirales;f__Ruminococcaceae;__	0	0	0	36	0	8	56	35	0
d__Bacteria;p__Firmicutes;c__Clostridia;o__Lachnospirales;f__Lachnospiraceae;g__Lachnoclostridium	861	0	323	232	0	358	45	414	565
d__Bacteria;p__Bacteroidota;c__Bacteroidia;o__Bacteroidales;f__Tannerellaceae;g__Parabacteroides	1365	492	539	0	124	136	126	47	133
d__Bacteria;p__Elusimicrobiota;c__Elusimicrobia;o__Elusimicrobiales;f__Elusimicrobiaceae;g__Elusimicrobium	0	0	180	26	24	0	103	25	295
d__Bacteria;p__Firmicutes;c__Clostridia;o__Lachnospirales;f__Defluviitaleaceae;g__Defluviitaleaceae_UCG-011	66	0	36	104	0	0	27	29	0
d__Bacteria;p__Firmicutes;c__Bacilli;o__Erysipelotrichales;f__Erysipelotrichaceae;g__Dubosiella	0	0	0	0	0	48	0	26	242
d__Bacteria;p__Firmicutes;c__Clostridia;o__Oscillospirales;f__Ruminococcaceae;g__Ruminococcaceae	0	0	0	0	0	27	22	0	0
d__Bacteria;p__Actinobacteriota;c__Actinobacteria;o__Bifidobacteriales;f__Bifidobacteriaceae;g__Bifidobacterium	124	0	0	457	138	0	632	1390	770
d__Bacteria;p__Firmicutes;c__Clostridia;o__Lachnospirales;f__Lachnospiraceae;g__Lachnospiraceae_UCG-001	0	0	0	0	168	14	0	0	0
d__Bacteria;p__Actinobacteriota;c__Coriobacteriia;o__Coriobacteriales;f__Eggerthellaceae;g__Paraeggerthella	0	466	1811	0	0	0	0	19	0
d__Bacteria;p__Desulfobacterota;c__Desulfovibrionia;o__Desulfovibrionales;f__Desulfovibrionaceae;g__Bilophila	2411	0	1284	0	0	0	88	22	117
d__Bacteria;p__Verrucomicrobiota;c__Verrucomicrobiae;o__Verrucomicrobiales;f__Akkermansiaceae;g__Akkermansia	0	3399	0	0	650	0	94	0	89
d__Bacteria;p__Firmicutes;c__Clostridia;o__Lachnospirales;f__Lachnospiraceae;g__Lachnospira	0	32	0	199	0	0	35	10	50
d__Bacteria;p__Bacteroidota;c__Bacteroidia;o__Bacteroidales;f__Muribaculaceae;g__Muribaculum	0	49	0	81	0	0	41	146	92
d__Bacteria;p__Actinobacteriota;c__Coriobacteriia;o__Coriobacteriales;f__Coriobacteriales_Incertae_Sedis;g__uncultured	16	96	134	0	29	9	32	6	6
d__Bacteria;p__Bacteroidota;c__Bacteroidia;o__Bacteroidales;f__Marinifilaceae;g__Odoribacter	44	0	25	0	0	0	4	14	22
d__Bacteria;p__Firmicutes;c__Clostridia;o__Lachnospirales;f__Lachnospiraceae;g__[Eubacterium]_ruminantium_group	0	36	0	32	83	156	32	307	45
d__Bacteria;p__Firmicutes;c__Clostridia;o__Peptostreptococcales-Tissierellales;f__Anaerovoracaceae;g__[Eubacterium]_brachy_group	0	0	0	53	0	0	24	16	33
d__Bacteria;p__Firmicutes;c__Clostridia;o__Oscillospirales;__;__	0	29	0	10	10	0	29	53	0
d__Bacteria;p__Firmicutes;c__Clostridia;o__Oscillospirales;f__Ruminococcaceae;g__UBA1819	3619	0	552	0	0	18	0	55	0
d__Bacteria;p__Actinobacteriota;c__Coriobacteriia;o__Coriobacteriales;f__Eggerthellaceae;g__Adlercreutzia	564	0	140	39	0	73	0	23	0
d__Bacteria;p__Firmicutes;c__Clostridia;o__Lachnospirales;f__Lachnospiraceae;g__Frisingicoccus	74	22	35	104	0	254	18	270	47
d__Bacteria;p__Actinobacteriota;c__Coriobacteriia;o__Coriobacteriales;f__Atopobiaceae;__	0	0	0	260	204	563	287	285	380
d__Bacteria;p__Firmicutes;c__Clostridia;o__Oscillospirales;f__Oscillospiraceae;g__Papillibacter	0	0	61	0	0	0	0	21	31
d__Bacteria;p__Bacteroidota;c__Bacteroidia;o__Bacteroidales;f__Rikenellaceae;g__dgA-11_gut_group	0	0	0	35	0	12	34	40	32
d__Bacteria;p__Proteobacteria;c__Gammaproteobacteria;o__Burkholderiales;f__Sutterellaceae;g__Parasutterella	871	0	237	54	9	44	306	115	80
d__Bacteria;p__Actinobacteriota;c__Coriobacteriia;o__Coriobacteriales;f__Coriobacteriaceae;g__Collinsella	4429	73	88	0	0	0	0	0	0
d__Bacteria;p__Firmicutes;c__Clostridia;o__Christensenellales;f__Christensenellaceae;__	144	0	62	0	0	0	0	53	0
d__Archaea;p__Euryarchaeota;c__Methanobacteria;o__Methanobacteriales;f__Methanobacteriaceae;g__Methanobrevibacter	8896	14	3624	1868	1761	5494	3509	3578	32
d__Bacteria;p__Firmicutes;c__Clostridia;o__Oscillospirales;f__Oscillospiraceae;g__Oscillibacter	0	0	85	0	20	0	53	0	44
d__Bacteria;p__Firmicutes;c__Clostridia;o__Peptostreptococcales-Tissierellales;f__Anaerovoracaceae;g__Mogibacterium	54	0	265	59	78	61	76	184	67
d__Bacteria;p__Fusobacteriota;c__Fusobacteriia;o__Fusobacteriales;f__Fusobacteriaceae;g__Fusobacterium	21100	134	2618	0	0	11	0	72	292
d__Bacteria;p__Firmicutes;c__Clostridia;o__Lachnospirales;f__Lachnospiraceae;g__Lachnospiraceae_UCG-010	0	0	0	0	21	0	0	0	0
d__Bacteria;p__Bacteroidota;c__Bacteroidia;o__Bacteroidales;f__Prevotellaceae;g__Prevotellaceae_NK3B31_group	0	0	256	20	312	174	337	41	0
d__Bacteria;p__Firmicutes;c__Clostridia;o__Peptostreptococcales-Tissierellales;f__Anaerovoracaceae;g__Family_XIII_AD3011_group	340	27	535	256	89	143	71	157	135
d__Bacteria;p__Firmicutes;c__Bacilli;o__Erysipelotrichales;f__Erysipelatoclostridiaceae;g__Erysipelatoclostridiaceae	271	14	361	0	0	81	0	0	0
d__Bacteria;p__Firmicutes;c__Clostridia;o__Lachnospirales;f__Lachnospiraceae;g__Dorea	60	17	19	45	0	47	45	0	0
d__Bacteria;p__Proteobacteria;c__Gammaproteobacteria;o__Enterobacterales;f__Enterobacteriaceae;g__Escherichia-Shigella	2399	531	180	0	12	0	146	715	184
d__Bacteria;p__Actinobacteriota;c__Coriobacteriia;o__Coriobacteriales;f__Atopobiaceae;g__uncultured	150	196	214	24	0	9	20	55	21
d__Bacteria;p__Actinobacteriota;c__Actinobacteria;o__Actinomycetales;f__Actinomycetaceae;g__Actinomycetaceae	503	390	56	0	0	0	0	0	0
d__Bacteria;p__Spirochaetota;c__Spirochaetia;o__Spirochaetales;f__Spirochaetaceae;g__Treponema	0	0	0	681	1385	247	588	7	21
d__Bacteria;p__Firmicutes;c__Clostridia;o__Oscillospirales;f__Ruminococcaceae;g__[Eubacterium]_siraeum_group	0	0	0	0	103	0	70	0	0
d__Bacteria;p__Firmicutes;c__Clostridia;o__Lachnospirales;f__Lachnospiraceae;g__[Ruminococcus]_torques_group	16	12	29	0	0	72	17	37	0
**Total Count/Treatment**	**84709**	**12792**	**36931**	**27798**	**33827**	**35666**	**47686**	**37559**	**20647**

Supplementary File 5
Relative Abundance Data

9DPT Fecal Genus Level Taxon Frequency	AC-PO7d	AC-10mg/kg	AC-5mg/kg	anti-rsPilA	anti-tip-chimer	Ofloxacin	TS-30mg/kg	TS-15mg/kg	Saline
d__Bacteria;p__Firmicutes;c__Clostridia;o__Oscillospirales;f__Oscillospiraceae;g__uncultured	335	80	1707	290	350	86	483	613	88
d__Bacteria;p__Firmicutes;c__Bacilli;o__Erysipelotrichales;f__Erysipelotrichaceae;__	124	492	55	2584	3079	3091	6299	7240	3808
d__Bacteria;p__Firmicutes;c__Clostridia;o__Christensenellales;f__Christensenellaceae;g__Christensenellaceae_R-7_group	1491	1037	1628	1311	1326	997	460	2535	261
d__Bacteria;p__Proteobacteria;c__Gammaproteobacteria;o__Pasteurellales;f__Pasteurellaceae;g__Haemophilus	586	0	0	120	73	0	0	0	0
d__Bacteria;p__Bacteroidota;c__Bacteroidia;o__Bacteroidales;__;__	150	36	62	141	45	57	92	289	66
d__Bacteria;p__Actinobacteriota;c__Actinobacteria;o__Actinomycetales;f__Actinomycetaceae;g__Actinomyces	0	233	0	76	15	0	19	14	0
d__Bacteria;p__Patescibacteria;c__Saccharimonadia;o__Saccharimonadales;f__Saccharimonadaceae;g__Candidatus_Saccharimonas	48	0	58	2203	1795	731	10819	4421	261
d__Bacteria;p__Bacteroidota;c__Bacteroidia;o__Bacteroidales;f__Muribaculaceae;g__Muribaculaceae	3775	1734	4114	7618	8065	5106	7759	9297	4000
d__Bacteria;p__Firmicutes;c__Clostridia;o__Lachnospirales;f__Lachnospiraceae;__	4473	1816	4806	808	3689	1189	455	1685	948
d__Bacteria;p__Actinobacteriota;c__Coriobacteriia;o__Coriobacteriales;f__Eggerthellaceae;g__Enterorhabdus	186	286	328	236	264	23	759	317	30
d__Bacteria;p__Firmicutes;c__Clostridia;o__Clostridia_UCG-014;f__Clostridia_UCG-014;g__Clostridia_UCG-014	194	0	76	164	372	306	383	315	79
d__Bacteria;p__Actinobacteriota;c__Coriobacteriia;o__Coriobacteriales;f__uncultured;g__uncultured	32	632	233	10	30	13	93	87	24
d__Bacteria;p__Firmicutes;c__Bacilli;o__Erysipelotrichales;f__Erysipelotrichaceae;g__Allobaculum	0	0	100	658	1470	732	731	442	49
d__Bacteria;p__Firmicutes;c__Clostridia;o__Oscillospirales;f__Ruminococcaceae;g__Incertae_Sedis	44	386	90	143	648	152	641	502	164
d__Bacteria;p__Firmicutes;c__Bacilli;o__Erysipelotrichales;f__Erysipelotrichaceae;g__uncultured	82	61	64	1004	1113	190	674	344	230
d__Bacteria;p__Bacteroidota;c__Bacteroidia;o__Bacteroidales;f__Marinifilaceae;g__Butyricimonas	252	0	26	16	4	71	0	59	0
d__Bacteria;p__Actinobacteriota;c__Coriobacteriia;o__Coriobacteriales;__;__	0	0	18	69	32	22	124	100	0
d__Bacteria;p__Firmicutes;c__Clostridia;o__Lachnospirales;f__Lachnospiraceae;g__Marvinbryantia	856	129	228	78	50	68	65	0	0
d__Bacteria;p__Firmicutes;c__Clostridia;o__Peptostreptococcales-Tissierellales;f__Anaerovoracaceae;g__[Eubacterium]_nodatum_group	24	0	289	38	112	108	0	14	0
d__Bacteria;p__Firmicutes;c__Clostridia;o__Oscillospirales;f__Oscillospiraceae;g__NK4A214_group	107	0	17	122	245	11	95	167	47
d__Bacteria;p__Bacteroidota;c__Bacteroidia;o__Bacteroidales;f__Rikenellaceae;g__Rikenellaceae_RC9_gut_group	51	0	0	8	0	62	55	25	43
d__Bacteria;p__Bacteroidota;c__Bacteroidia;o__Bacteroidales;f__Prevotellaceae;g__Prevotellaceae_UCG-001	619	249	0	412	861	1238	708	1091	353
d__Bacteria;p__Firmicutes;c__Clostridia;o__Oscillospirales;f__[Eubacterium]_coprostanoligenes_group;g__[Eubacterium]_coprostanoligenes_group	124	69	409	11	69	197	359	222	39
d__Bacteria;p__Bacteroidota;c__Bacteroidia;o__Bacteroidales;f__Prevotellaceae;g__Prevotella	857	532	197	241	738	582	677	442	761
d__Bacteria;p__Planctomycetota;c__Planctomycetes;o__Pirellulales;f__Pirellulaceae;g__p-1088-a5_gut_group	64	127	61	65	39	45	83	52	34
d__Bacteria;p__Firmicutes;c__Clostridia;o__Oscillospirales;f__Ruminococcaceae;g__Ruminococcus	3734	517	1342	306	1623	2075	781	1357	978
d__Bacteria;p__Bacteroidota;c__Bacteroidia;o__Bacteroidales;f__Prevotellaceae;g__Prevotellaceae_Ga6A1_group	0	0	0	38	117	69	185	118	32
d__Bacteria;p__Bacteroidota;c__Bacteroidia;o__Bacteroidales;f__Bacteroidaceae;g__Bacteroides	20507	1621	3600	320	169	1326	248	1103	142
d__Bacteria;p__Firmicutes;c__Bacilli;o__Erysipelotrichales;f__Erysipelotrichaceae;g__Ileibacterium	142	1104	747	1959	848	2918	876	798	246
d__Bacteria;p__Firmicutes;c__Clostridia;o__Monoglobales;f__Monoglobaceae;g__Monoglobus	59	875	364	183	427	146	840	15	70
d__Bacteria;p__Bacteroidota;c__Bacteroidia;o__Bacteroidales;f__Rikenellaceae;g__Alistipes	586	324	179	199	11	0	123	437	24
d__Bacteria;p__Firmicutes;c__Clostridia;o__Lachnospirales;f__Lachnospiraceae;g__Coprococcus	164	262	514	66	15	251	9	0	0
d__Bacteria;p__Firmicutes;c__Bacilli;o__Lactobacillales;f__Lactobacillaceae;g__Lactobacillus	0	986	26	254	193	0	151	402	1492
d__Bacteria;p__Bacteroidota;c__Bacteroidia;o__Bacteroidales;f__Muribaculaceae;__	5	218	0	104	0	0	94	0	0
d__Bacteria;p__Firmicutes;c__Bacilli;o__Erysipelotrichales;f__Erysipelatoclostridiaceae;__	0	0	28	59	0	58	0	15	18
d__Bacteria;p__Firmicutes;c__Negativicutes;o__Acidaminococcales;f__Acidaminococcaceae;g__Phascolarctobacterium	0	587	115	1190	139	187	235	593	126
d__Bacteria;p__Firmicutes;c__Clostridia;o__Lachnospirales;f__Lachnospiraceae;g__uncultured	0	0	22	0	59	0	106	0	0
d__Archaea;p__Euryarchaeota;c__Methanobacteria;o__Methanobacteriales;f__Methanobacteriaceae;g__Methanosphaera	7	0	201	0	0	0	20	147	0
d__Bacteria;p__Firmicutes;c__Clostridia;o__Oscillospirales;f__Butyricicoccaceae;g__UCG-008	167	211	51	101	142	0	13	0	142
d__Bacteria;p__Firmicutes;c__Bacilli;o__RF39;f__RF39;g__RF39	0	0	0	49	93	68	123	54	30
d__Bacteria;p__Firmicutes;c__Clostridia;o__Oscillospirales;f__Oscillospiraceae;g__Colidextribacter	768	155	184	87	299	119	73	209	0
d__Bacteria;p__Firmicutes;c__Clostridia;o__Eubacteriales;f__Eubacteriaceae;g__uncultured	0	398	869	1307	2250	1389	1636	2163	970
d__Bacteria;p__Firmicutes;c__Bacilli;o__Lactobacillales;f__Streptococcaceae;g__Streptococcus	23	238	28	1159	167	15	631	304	33
d__Bacteria;p__Bacteroidota;c__Bacteroidia;o__Bacteroidales;f__Prevotellaceae;g__Prevotellaceae_UCG-003	0	0	0	11	50	33	0	0	0
d__Bacteria;p__Desulfobacterota;c__Desulfovibrionia;o__Desulfovibrionales;f__Desulfovibrionaceae;g__Desulfovibrio	0	0	0	0	102	0	0	0	15
d__Bacteria;p__Firmicutes;c__Clostridia;o__Oscillospirales;f__Oscillospiraceae;__	499	89	225	55	217	87	220	182	127
d__Bacteria;p__Firmicutes;c__Bacilli;o__Erysipelotrichales;f__Erysipelotrichaceae;g__Faecalibaculum	34	570	354	39	0	0	100	25	33

Supplementary File 5
Relative Abundance Data

9DPT Fecal Genus Level Taxon Frequency Continued	AC-PO7d	AC-10mg/kg	AC-5mg/kg	anti-rsPilA	anti-tip-chimer	Ofloxacin	TS-30mg/kg	TS-15mg/kg	Saline
d__Bacteria;p__Actinobacteriota;c__Coriobacteriia;o__Coriobacteriales;f__Eggerthellaceae;g__uncultured	102	360	985	459	149	676	476	897	123
d__Bacteria;p__Firmicutes;c__Clostridia;o__Lachnospirales;f__Lachnospiraceae;g__Lachnospiraceae_NK4A136_group	1376	68	187	77	456	199	69	304	55
d__Bacteria;p__Firmicutes;c__Clostridia;o__Oscillospirales;f__Oscillospiraceae;g__UCG-005	1745	651	437	475	867	385	234	350	196
d__Bacteria;p__Firmicutes;c__Clostridia;o__Lachnospirales;f__Lachnospiraceae;g__Blautia	579	548	1914	98	62	188	78	223	91
d__Bacteria;p__Firmicutes;c__Clostridia;o__Lachnospirales;f__Lachnospiraceae;g__Lachnospiraceae_UCG-004	143	21	142	14	34	16	0	0	0
d__Bacteria;p__Actinobacteriota;c__Coriobacteriia;o__Coriobacteriales;f__Eggerthellaceae;__	0	36	9	302	197	57	506	462	33
d__Bacteria;p__Firmicutes;c__Clostridia;o__Clostridia;f__Hungateiclostridiaceae;g__Ruminiclostridium	107	30	78	0	0	64	0	45	0
d__Bacteria;p__Firmicutes;c__Clostridia;o__Oscillospirales;f__Ruminococcaceae;__	0	0	0	10	11	55	0	0	0
d__Bacteria;p__Firmicutes;c__Clostridia;o__Lachnospirales;f__Lachnospiraceae;g__Lachnoclostridium	859	121	576	222	184	286	247	345	134
d__Bacteria;p__Bacteroidota;c__Bacteroidia;o__Bacteroidales;f__Tannerellaceae;g__Parabacteroides	1679	243	1226	74	43	130	145	167	34
d__Bacteria;p__Elusimicrobiota;c__Elusimicrobia;o__Elusimicrobiales;f__Elusimicrobiaceae;g__Elusimicrobium	0	50	97	141	137	20	103	30	58
d__Bacteria;p__Firmicutes;c__Clostridia;o__Lachnospirales;f__Defluviitaleaceae;g__Defluviitaleaceae_UCG-011	59	46	20	244	75	0	40	130	0
d__Bacteria;p__Firmicutes;c__Bacilli;o__Erysipelotrichales;f__Erysipelotrichaceae;g__Dubosiella	0	0	0	0	0	54	0	149	0
d__Bacteria;p__Firmicutes;c__Clostridia;o__Oscillospirales;f__Ruminococcaceae;g__Ruminococcaceae	0	0	0	0	38	0	35	0	0
d__Bacteria;p__Actinobacteriota;c__Actinobacteria;o__Bifidobacteriales;f__Bifidobacteriaceae;g__Bifidobacterium	35	0	24	193	110	0	811	1073	316
d__Bacteria;p__Firmicutes;c__Clostridia;o__Lachnospirales;f__Lachnospiraceae;g__Lachnospiraceae_UCG-001	0	0	0	0	242	58	0	0	78
d__Bacteria;p__Actinobacteriota;c__Coriobacteriia;o__Coriobacteriales;f__Eggerthellaceae;g__Paraeggerthella	0	350	3178	0	0	92	0	0	0
d__Bacteria;p__Desulfobacterota;c__Desulfovibrionia;o__Desulfovibrionales;f__Desulfovibrionaceae;g__Bilophila	4404	0	1467	0	0	0	0	109	38
d__Bacteria;p__Verrucomicrobiota;c__Verrucomicrobiae;o__Verrucomicrobiales;f__Akkermansiaceae;g__Akkermansia	0	2915	0	0	357	1120	0	1792	0
d__Bacteria;p__Firmicutes;c__Clostridia;o__Lachnospirales;f__Lachnospiraceae;g__Lachnospira	0	163	93	0	0	59	0	22	0
d__Bacteria;p__Bacteroidota;c__Bacteroidia;o__Bacteroidales;f__Muribaculaceae;g__Muribaculum	0	32	0	31	22	28	11	195	0
d__Bacteria;p__Actinobacteriota;c__Coriobacteriia;o__Coriobacteriales;f__Coriobacteriales_Incertae_Sedis;g__uncultured	25	183	187	68	38	0	97	102	0
d__Bacteria;p__Bacteroidota;c__Bacteroidia;o__Bacteroidales;f__Marinifilaceae;g__Odoribacter	195	0	0	0	9	0	14	32	0
d__Bacteria;p__Firmicutes;c__Clostridia;o__Lachnospirales;f__Lachnospiraceae;g__[Eubacterium]_ruminantium_group	0	0	80	0	141	0	51	155	63
d__Bacteria;p__Firmicutes;c__Clostridia;o__Oscillospirales;__;__	23	257	0	25	54	51	63	87	0
d__Bacteria;p__Firmicutes;c__Clostridia;o__Oscillospirales;f__Ruminococcaceae;g__UBA1819	341	19	960	9	0	44	0	64	0
d__Bacteria;p__Actinobacteriota;c__Coriobacteriia;o__Coriobacteriales;f__Eggerthellaceae;g__Adlercreutzia	261	18	227	0	0	58	0	21	0
d__Bacteria;p__Firmicutes;c__Clostridia;o__Lachnospirales;f__Lachnospiraceae;g__Frisingicoccus	0	12	254	18	14	26	10	188	59
d__Bacteria;p__Actinobacteriota;c__Coriobacteriia;o__Coriobacteriales;f__Atopobiaceae;__	39	16	20	195	416	134	498	206	414
d__Bacteria;p__Firmicutes;c__Clostridia;o__Oscillospirales;f__Oscillospiraceae;g__Papillibacter	129	0	78	0	0	0	38	15	10
d__Bacteria;p__Bacteroidota;c__Bacteroidia;o__Bacteroidales;f__Rikenellaceae;g__dgA-11_gut_group	17	10	25	91	24	32	25	141	18
d__Bacteria;p__Proteobacteria;c__Gammaproteobacteria;o__Burkholderiales;f__Sutterellaceae;g__Parasutterella	986	121	693	126	64	93	149	122	147
d__Bacteria;p__Actinobacteriota;c__Coriobacteriia;o__Coriobacteriales;f__Coriobacteriaceae;g__Collinsella	0	20	136	0	0	8	0	0	0
d__Bacteria;p__Firmicutes;c__Clostridia;o__Christensenellales;f__Christensenellaceae;__	173	36	105	0	0	0	0	0	0
d__Archaea;p__Euryarchaeota;c__Methanobacteria;o__Methanobacteriales;f__Methanobacteriaceae;g__Methanobrevibacter	4206	4554	3398	4148	1604	5187	3138	2022	238
d__Bacteria;p__Firmicutes;c__Clostridia;o__Oscillospirales;f__Oscillospiraceae;g__Oscillibacter	95	11	39	0	0	0	24	0	0
d__Bacteria;p__Firmicutes;c__Clostridia;o__Peptostreptococcales-Tissierellales;f__Anaerovoracaceae;g__Mogibacterium	59	35	1179	65	53	49	101	95	6
d__Bacteria;p__Fusobacteriota;c__Fusobacteriia;o__Fusobacteriales;f__Fusobacteriaceae;g__Fusobacterium	9230	41	3075	0	0	53	0	100	0
d__Bacteria;p__Firmicutes;c__Clostridia;o__Lachnospirales;f__Lachnospiraceae;g__Lachnospiraceae_UCG-010	0	0	110	0	0	0	0	0	0
d__Bacteria;p__Bacteroidota;c__Bacteroidia;o__Bacteroidales;f__Prevotellaceae;g__Prevotellaceae_NK3B31_group	0	0	513	60	338	218	131	356	311
d__Bacteria;p__Firmicutes;c__Clostridia;o__Peptostreptococcales-Tissierellales;f__Anaerovoracaceae;g__Family_XIII_AD3011_group	71	77	1104	203	112	202	115	196	92
d__Bacteria;p__Firmicutes;c__Bacilli;o__Erysipelotrichales;f__Erysipelatoclostridiaceae;g__Erysipelatoclostridiaceae	67	37	148	0	5	68	0	75	0
d__Bacteria;p__Firmicutes;c__Clostridia;o__Lachnospirales;f__Lachnospiraceae;g__Dorea	83	46	45	0	18	43	19	0	0
d__Bacteria;p__Proteobacteria;c__Gammaproteobacteria;o__Enterobacterales;f__Enterobacteriaceae;g__Escherichia-Shigella	2174	56	154	23	342	8	32	758	12
d__Bacteria;p__Actinobacteriota;c__Coriobacteriia;o__Coriobacteriales;f__Atopobiaceae;g__uncultured	37	243	231	43	19	8	50	24	33
d__Bacteria;p__Actinobacteriota;c__Actinobacteria;o__Actinomycetales;f__Actinomycetaceae;g__Actinomycetaceae	103	105	57	0	0	66	0	0	0
d__Bacteria;p__Spirochaetota;c__Spirochaetia;o__Spirochaetales;f__Spirochaetaceae;g__Treponema	0	0	0	351	356	210	444	1399	0
d__Bacteria;p__Firmicutes;c__Clostridia;o__Oscillospirales;f__Ruminococcaceae;g__[Eubacterium]_siraeum_group	0	0	0	24	106	0	164	0	0
d__Bacteria;p__Firmicutes;c__Clostridia;o__Lachnospirales;f__Lachnospiraceae;g__[Ruminococcus]_torques_group	212	0	36	0	25	20	0	0	0
**Total Count/Treatment**	**70783**	**27585**	**46702**	**33701**	**38127**	**33883**	**46215**	**50646**	**18322**

Supplementary File 5
Relative Abundance Data

Baseline NPL Genus Level Taxon Frequency	AC-PO7d	AC-10mg/kg	AC-5mg/kg	anti-rsPilA	anti-tip-chimer	Ofloxacin	TS-30mg/kg	TS-15mg/kg	Saline
d__Bacteria;p__Actinobacteriota;c__Actinobacteria;o__Actinomycetales;f__Actinomycetaceae;g__Mobiluncus	3506	744	1208	1322	817	599	665	1362	1526
d__Bacteria;p__Actinobacteriota;c__Actinobacteria;o__Bifidobacteriales;f__Bifidobacteriaceae;g__Bifidobacterium	626	276	263	0	203	314	39	113	121
d__Bacteria;p__Campilobacterota;c__Campylobacteria;o__Campylobacterales;f__Campylobacteraceae;g__Campylobacter	7628	2688	3261	2526	3419	2725	4948	2879	2185
d__Bacteria;p__Bacteroidota;c__Bacteroidia;o__Bacteroidales;__;__	394	4194	7975	4280	4678	3556	307	4998	4600
d__Bacteria;p__Bacteroidota;c__Bacteroidia;o__Bacteroidales;f__Muribaculaceae;g__Muribaculaceae	1764	488	435	424	318	35	413	420	258
d__Bacteria;p__Actinobacteriota;c__Coriobacteriia;o__Coriobacteriales;f__Atopobiaceae;g__Olsenella	358	0	61	0	0	0	0	0	13
d__Bacteria;p__Actinobacteriota;c__Actinobacteria;o__Actinomycetales;f__Actinomycetaceae;__	1198	641	313	433	461	651	415	623	249
d__Bacteria;p__Firmicutes;c__Bacilli;o__Lactobacillales;f__Aerococcaceae;g__Aerococcus	287	0	0	107	73	86	216	0	40
d__Bacteria;p__Firmicutes;c__Bacilli;o__Staphylococcales;f__Staphylococcaceae;g__Staphylococcus	10355	542	299	940	441	290	1081	603	645
d__Bacteria;p__Fusobacteriota;c__Fusobacteriia;o__Fusobacteriales;f__Fusobacteriaceae;g__Fusobacterium	1305	902	574	440	516	1008	286	720	351
d__Bacteria;p__Proteobacteria;c__Gammaproteobacteria;o__Enterobacterales;f__Enterobacteriaceae;g__Escherichia-Shigella	795	0	0	0	31	332	162	0	0
d__Archaea;p__Euryarchaeota;c__Methanobacteria;o__Methanobacteriales;f__Methanobacteriaceae;g__Methanobrevibacter	630	0	17	129	0	0	58	35	0
d__Bacteria;p__Bacteroidota;c__Bacteroidia;o__Sphingobacteriales;f__Sphingobacteriaceae;__	478	482	588	426	989	653	3639	196	146
d__Bacteria;p__Firmicutes;c__Bacilli;o__Lactobacillales;f__Streptococcaceae;g__Streptococcus	88	0	0	259	0	0	152	27	0
d__Bacteria;p__Proteobacteria;c__Gammaproteobacteria;o__Enterobacterales;f__Enterobacteriaceae;__	399	0	290	468	189	0	726	0	229
d__Bacteria;p__Firmicutes;c__Bacilli;o__Erysipelotrichales;f__Erysipelotrichaceae;g__Ileibacterium	693	18	324	396	224	63	93	188	142
d__Bacteria;p__Proteobacteria;c__Gammaproteobacteria;o__Burkholderiales;__;__	383	632	275	666	3164	268	0	495	125
d__Bacteria;p__Bacteroidota;c__Bacteroidia;o__Chitinophagales;f__Chitinophagaceae;__	4748	766	721	1121	1068	1061	247	1249	558
d__Bacteria;p__Firmicutes;c__Bacilli;o__Erysipelotrichales;f__Erysipelotrichaceae;__	984	157	109	56	15	0	158	319	55
d__Bacteria;p__Patescibacteria;c__Saccharimonadia;o__Saccharimonadales;f__Saccharimonadales;g__Saccharimonadales	1288	256	425	283	902	151	377	219	65
d__Bacteria;p__Bacteroidota;c__Bacteroidia;o__Bacteroidales;f__Prevotellaceae;__	134	0	0	0	0	0	0	74	132
d__Bacteria;p__Fusobacteriota;c__Fusobacteriia;o__Fusobacteriales;f__Leptotrichiaceae;__	1291	0	0	111	563	385	300	314	185
d__Bacteria;p__Proteobacteria;c__Gammaproteobacteria;o__Pasteurellales;f__Pasteurellaceae;g__Actinobacillus	432	109	0	117	81	51	0	82	198
d__Bacteria;p__Firmicutes;c__Bacilli;o__Mycoplasmatales;f__Mycoplasmataceae;g__Mycoplasma	646	241	176	191	459	319	370	328	267
d__Bacteria;p__Actinobacteriota;c__Actinobacteria;o__Corynebacteriales;f__Corynebacteriaceae;g__Corynebacterium	0	0	72	0	73	0	315	86	0
d__Bacteria;p__Firmicutes;c__Clostridia;o__Eubacteriales;f__Eubacteriaceae;g__uncultured	597	172	100	0	15	11	109	0	0
d__Bacteria;p__Proteobacteria;c__Gammaproteobacteria;o__Xanthomonadales;f__Xanthomonadaceae;g__Xanthomonas	155	267	199	12	228	0	77	21	0
d__Bacteria;p__Bacteroidota;c__Bacteroidia;o__Bacteroidales;f__Bacteroidaceae;g__Bacteroides	275	0	0	0	0	115	187	0	0
d__Bacteria;p__Firmicutes;c__Clostridia;o__Lachnospirales;f__Lachnospiraceae;__	579	165	0	164	164	0	0	54	102
d__Bacteria;p__Actinobacteriota;c__Actinobacteria;o__Micrococcales;__;__	0	179	0	121	0	0	68	0	0
d__Bacteria;p__Firmicutes;c__Clostridia;o__Peptostreptococcales-Tissierellales;f__Peptostreptococcales-Tissierellales;g__Peptoniphilus	157	0	0	89	0	0	0	122	0
d__Bacteria;p__Bacteroidota;c__Bacteroidia;o__Bacteroidales;f__Prevotellaceae;g__Prevotellaceae_UCG-001	394	0	0	36	0	0	83	0	107
**Total Count/Treatment**	**42567**	**13919**	**17685**	**15117**	**19091**	**12673**	**15491**	**15527**	**12299**

2DPT NPL Genus Level Taxon Frequency	AC-PO7d	AC-10mg/kg	AC-5mg/kg	anti-rsPilA	anti-tip-chimer	Ofloxacin	TS-30mg/kg	TS-15mg/kg	Saline
d__Bacteria;p__Actinobacteriota;c__Actinobacteria;o__Actinomycetales;f__Actinomycetaceae;g__Mobiluncus	0	656	897	1103	988	822	609	1328	1543
d__Bacteria;p__Actinobacteriota;c__Actinobacteria;o__Bifidobacteriales;f__Bifidobacteriaceae;g__Bifidobacterium	182	212	300	270	154	466	0	83	521
d__Bacteria;p__Campilobacterota;c__Campylobacteria;o__Campylobacterales;f__Campylobacteraceae;g__Campylobacter	118	2991	3242	2623	3631	2131	5569	2561	2488
d__Bacteria;p__Bacteroidota;c__Bacteroidia;o__Bacteroidales;__;__	232	3080	10307	5407	4845	4669	117	4721	4519
d__Bacteria;p__Bacteroidota;c__Bacteroidia;o__Bacteroidales;f__Muribaculaceae;g__Muribaculaceae	34	301	218	207	317	25	206	200	502
d__Bacteria;p__Actinobacteriota;c__Coriobacteriia;o__Coriobacteriales;f__Atopobiaceae;g__Olsenella	0	0	61	0	58	0	0	0	11
d__Bacteria;p__Firmicutes;c__Clostridia;o__Peptostreptococcales-Tissierellales;f__Peptostreptococcales-Tissierellales;g__Peptoniphilus	128	129	24	0	37	0	9	265	116
d__Bacteria;p__Actinobacteriota;c__Actinobacteria;o__Actinomycetales;f__Actinomycetaceae;__	116	1098	685	590	472	715	465	521	278
d__Bacteria;p__Firmicutes;c__Bacilli;o__Lactobacillales;f__Aerococcaceae;g__Aerococcus	60	0	0	182	94	109	0	156	0
d__Bacteria;p__Firmicutes;c__Bacilli;o__Staphylococcales;f__Staphylococcaceae;g__Staphylococcus	0	316	751	568	1629	2002	613	327	2324
d__Bacteria;p__Fusobacteriota;c__Fusobacteriia;o__Fusobacteriales;f__Fusobacteriaceae;g__Fusobacterium	0	511	349	531	638	1097	246	673	482
d__Bacteria;p__Proteobacteria;c__Gammaproteobacteria;o__Enterobacterales;f__Enterobacteriaceae;g__Escherichia-Shigella	3445	30	141	0	41	234	220	0	0
d__Archaea;p__Euryarchaeota;c__Methanobacteria;o__Methanobacteriales;f__Methanobacteriaceae;g__Methanobrevibacter	2315	73	79	110	120	0	14	187	0
d__Bacteria;p__Bacteroidota;c__Bacteroidia;o__Sphingobacteriales;f__Sphingobacteriaceae;__	0	88	323	513	928	493	2682	218	195
d__Bacteria;p__Firmicutes;c__Bacilli;o__Lactobacillales;f__Streptococcaceae;g__Streptococcus	0	0	0	480	0	0	359	41	0
d__Bacteria;p__Proteobacteria;c__Gammaproteobacteria;o__Enterobacterales;f__Enterobacteriaceae;__	341	0	815	546	244	169	324	0	245
d__Bacteria;p__Firmicutes;c__Bacilli;o__Erysipelotrichales;f__Erysipelotrichaceae;g__Ileibacterium	0	137	374	160	313	0	41	377	174
d__Bacteria;p__Proteobacteria;c__Gammaproteobacteria;o__Burkholderiales;__;__	367	3294	600	1368	2643	462	0	584	102
d__Bacteria;p__Bacteroidota;c__Bacteroidia;o__Chitinophagales;f__Chitinophagaceae;__	45	985	596	833	854	71	137	235	839
d__Bacteria;p__Firmicutes;c__Bacilli;o__Erysipelotrichales;f__Erysipelotrichaceae;__	0	183	79	0	216	42	251	126	52
d__Bacteria;p__Patescibacteria;c__Saccharimonadia;o__Saccharimonadales;f__Saccharimonadales;g__Saccharimonadales	407	451	473	258	271	139	463	129	0
d__Bacteria;p__Bacteroidota;c__Bacteroidia;o__Bacteroidales;f__Prevotellaceae;__	0	0	0	0	47	0	0	0	30
d__Bacteria;p__Fusobacteriota;c__Fusobacteriia;o__Fusobacteriales;f__Leptotrichiaceae;__	34	16	0	198	611	355	114	279	215
d__Bacteria;p__Proteobacteria;c__Gammaproteobacteria;o__Pasteurellales;f__Pasteurellaceae;g__Actinobacillus	25	210	136	44	160	0	0	0	119
d__Bacteria;p__Firmicutes;c__Bacilli;o__Mycoplasmatales;f__Mycoplasmataceae;g__Mycoplasma	369	219	509	202	189	243	414	341	407
d__Bacteria;p__Actinobacteriota;c__Actinobacteria;o__Corynebacteriales;f__Corynebacteriaceae;g__Corynebacterium	0	0	0	0	0	0	489	0	0
d__Bacteria;p__Firmicutes;c__Clostridia;o__Eubacteriales;f__Eubacteriaceae;g__uncultured	0	226	0	0	0	0	41	154	90
d__Bacteria;p__Proteobacteria;c__Gammaproteobacteria;o__Xanthomonadales;f__Xanthomonadaceae;g__Xanthomonas	0	123	254	0	118	0	0	102	141
d__Bacteria;p__Bacteroidota;c__Bacteroidia;o__Bacteroidales;f__Bacteroidaceae;g__Bacteroides	2241	0	0	0	0	0	239	0	0
d__Bacteria;p__Firmicutes;c__Clostridia;o__Lachnospirales;f__Lachnospiraceae;__	11	0	37	0	40	208	0	304	0
d__Bacteria;p__Actinobacteriota;c__Actinobacteria;o__Micrococcales;__;__	0	0	0	0	0	0	87	49	0
d__Bacteria;p__Bacteroidota;c__Bacteroidia;o__Bacteroidales;f__Prevotellaceae;g__Prevotellaceae_UCG-001	201	0	0	0	0	0	64	0	0
**Total Count/Treatment**	**10671**	**15329**	**21250**	**16193**	**19658**	**14452**	**13773**	**13961**	**15393**

Supplementary File 5
Relative Abundance Data

5DPT NPL Genus Level Taxon Frequency	AC-PO7d	AC-10mg/kg	AC-5mg/kg	anti-rsPilA	anti-tip-chimer	Ofloxacin	TS-30mg/kg	TS-15mg/kg	Saline
d__Bacteria;p__Actinobacteriota;c__Actinobacteria;o__Actinomycetales;f__Actinomycetaceae;g__Mobiluncus	1324	284	724	769	878	745	812	1456	1568
d__Bacteria;p__Actinobacteriota;c__Actinobacteria;o__Bifidobacteriales;f__Bifidobacteriaceae;g__Bifidobacterium	181	180	422	75	158	345	0	0	289
d__Bacteria;p__Campilobacterota;c__Campylobacteria;o__Campylobacterales;f__Campylobacteraceae;g__Campylobacter	2524	1372	2582	2075	2902	2156	4061	3007	2437
d__Bacteria;p__Bacteroidota;c__Bacteroidia;o__Bacteroidales;__;__	635	2733	8038	4338	4512	4262	22	5759	3134
d__Bacteria;p__Bacteroidota;c__Bacteroidia;o__Bacteroidales;f__Muribaculaceae;g__Muribaculaceae	115	28	180	334	311	42	382	499	264
d__Bacteria;p__Actinobacteriota;c__Coriobacteriia;o__Coriobacteriales;f__Atopobiaceae;g__Olsenella	0	14	0	32	0	0	0	0	0
d__Bacteria;p__Firmicutes;c__Clostridia;o__Peptostreptococcales-Tissierellales;f__Peptostreptococcales-Tissierellales;g__Peptoniphilus	238	94	0	0	0	8	0	116	0
d__Bacteria;p__Actinobacteriota;c__Actinobacteria;o__Actinomycetales;f__Actinomycetaceae;__	181	724	385	0	156	466	486	413	269
d__Bacteria;p__Firmicutes;c__Bacilli;o__Lactobacillales;f__Aerococcaceae;g__Aerococcus	214	0	0	150	236	0	59	206	0
d__Bacteria;p__Firmicutes;c__Bacilli;o__Staphylococcales;f__Staphylococcaceae;g__Staphylococcus	1447	869	479	1422	735	843	300	872	1803
d__Bacteria;p__Fusobacteriota;c__Fusobacteriia;o__Fusobacteriales;f__Fusobacteriaceae;g__Fusobacterium	117	302	239	0	663	816	426	881	374
d__Bacteria;p__Proteobacteria;c__Gammaproteobacteria;o__Enterobacterales;f__Enterobacteriaceae;g__Escherichia-Shigella	25417	152	0	0	48	248	0	89	0
d__Archaea;p__Euryarchaeota;c__Methanobacteria;o__Methanobacteriales;f__Methanobacteriaceae;g__Methanobrevibacter	314	0	0	107	74	261	0	86	0
d__Bacteria;p__Bacteroidota;c__Bacteroidia;o__Sphingobacteriales;f__Sphingobacteriaceae;__	104	205	1529	175	1208	96	3118	408	187
d__Bacteria;p__Firmicutes;c__Bacilli;o__Lactobacillales;f__Streptococcaceae;g__Streptococcus	214	0	0	115	0	0	356	59	1017
d__Bacteria;p__Proteobacteria;c__Gammaproteobacteria;o__Enterobacterales;f__Enterobacteriaceae;__	721	0	719	330	0	140	849	0	252
d__Bacteria;p__Firmicutes;c__Bacilli;o__Erysipelotrichales;f__Erysipelotrichaceae;g__Ileibacterium	90	41	231	421	221	464	217	302	24
d__Bacteria;p__Proteobacteria;c__Gammaproteobacteria;o__Burkholderiales;__;__	1250	3743	1120	1222	2597	94	0	681	276
d__Bacteria;p__Bacteroidota;c__Bacteroidia;o__Chitinophagales;f__Chitinophagaceae;__	1312	608	613	440	732	414	0	449	775
d__Bacteria;p__Firmicutes;c__Bacilli;o__Erysipelotrichales;f__Erysipelotrichaceae;__	318	42	41	117	199	344	331	521	100
d__Bacteria;p__Patescibacteria;c__Saccharimonadia;o__Saccharimonadales;f__Saccharimonadales;g__Saccharimonadales	2777	292	561	0	408	245	824	0	0
d__Bacteria;p__Bacteroidota;c__Bacteroidia;o__Bacteroidales;f__Prevotellaceae;__	76	0	0	0	35	0	0	0	37
d__Bacteria;p__Fusobacteriota;c__Fusobacteriia;o__Fusobacteriales;f__Leptotrichiaceae;__	10	344	0	0	207	0	248	178	447
d__Bacteria;p__Proteobacteria;c__Gammaproteobacteria;o__Pasteurellales;f__Pasteurellaceae;g__Actinobacillus	0	0	50	0	0	0	0	0	0
d__Bacteria;p__Firmicutes;c__Bacilli;o__Mycoplasmatales;f__Mycoplasmataceae;g__Mycoplasma	332	0	448	21	315	293	476	211	160
d__Bacteria;p__Actinobacteriota;c__Actinobacteria;o__Corynebacteriales;f__Corynebacteriaceae;g__Corynebacterium	132	0	0	0	0	0	117	0	0
d__Bacteria;p__Firmicutes;c__Clostridia;o__Eubacteriales;f__Eubacteriaceae;g__uncultured	104	47	0	0	32	203	0	109	0
d__Bacteria;p__Proteobacteria;c__Gammaproteobacteria;o__Xanthomonadales;f__Xanthomonadaceae;g__Xanthomonas	86	0	189	0	246	0	0	210	143
d__Bacteria;p__Bacteroidota;c__Bacteroidia;o__Bacteroidales;f__Bacteroidaceae;g__Bacteroides	5568	0	0	0	0	0	332	0	0
d__Bacteria;p__Firmicutes;c__Clostridia;o__Lachnospirales;f__Lachnospiraceae;__	0	0	0	0	0	0	0	142	0
d__Bacteria;p__Actinobacteriota;c__Actinobacteria;o__Micrococcales;__;__	0	0	0	0	0	97	0	96	0
d__Bacteria;p__Bacteroidota;c__Bacteroidia;o__Bacteroidales;f__Prevotellaceae;g__Prevotellaceae_UCG-001	0	0	0	13	62	0	0	251	44
**Total Count/Treatment**	**45801**	**12074**	**18550**	**12156**	**16935**	**12582**	**13416**	**17001**	**13600**

7DPT NPL Genus Level Taxon Frequency	AC-PO7d	AC-10mg/kg	AC-5mg/kg	anti-rsPilA	anti-tip-chimer	Ofloxacin	TS-30mg/kg	TS-15mg/kg	Saline
d__Bacteria;p__Actinobacteriota;c__Actinobacteria;o__Actinomycetales;f__Actinomycetaceae;g__Mobiluncus	1180	0	878	799	865	926	400	1347	1191
d__Bacteria;p__Actinobacteriota;c__Actinobacteria;o__Bifidobacteriales;f__Bifidobacteriaceae;g__Bifidobacterium	675	0	481	0	265	381	0	106	132
d__Bacteria;p__Campilobacterota;c__Campylobacteria;o__Campylobacterales;f__Campylobacteraceae;g__Campylobacter	5177	628	4583	862	3185	2977	2238	3776	2068
d__Bacteria;p__Bacteroidota;c__Bacteroidia;o__Bacteroidales;__;__	7817	2919	8822	2035	4351	4019	42	6311	3396
d__Bacteria;p__Bacteroidota;c__Bacteroidia;o__Bacteroidales;f__Muribaculaceae;g__Muribaculaceae	10	35	114	633	315	27	413	633	361
d__Bacteria;p__Actinobacteriota;c__Coriobacteriia;o__Coriobacteriales;f__Atopobiaceae;g__Olsenella	0	0	24	47	0	0	0	0	0
d__Bacteria;p__Firmicutes;c__Clostridia;o__Peptostreptococcales-Tissierellales;f__Peptostreptococcales-Tissierellales;g__Peptoniphilus	285	0	0	0	0	0	0	75	0
d__Bacteria;p__Actinobacteriota;c__Actinobacteria;o__Actinomycetales;f__Actinomycetaceae;__	28	528	408	0	276	897	119	409	309
d__Bacteria;p__Firmicutes;c__Bacilli;o__Lactobacillales;f__Aerococcaceae;g__Aerococcus	0	0	0	167	148	0	65	203	0
d__Bacteria;p__Firmicutes;c__Bacilli;o__Staphylococcales;f__Staphylococcaceae;g__Staphylococcus	6293	1751	755	5051	1065	3949	205	1744	1290
d__Bacteria;p__Fusobacteriota;c__Fusobacteriia;o__Fusobacteriales;f__Fusobacteriaceae;g__Fusobacterium	0	474	482	0	488	1000	133	941	555
d__Bacteria;p__Proteobacteria;c__Gammaproteobacteria;o__Enterobacterales;f__Enterobacteriaceae;g__Escherichia-Shigella	21831	0	23	0	0	131	345	63	0
d__Archaea;p__Euryarchaeota;c__Methanobacteria;o__Methanobacteriales;f__Methanobacteriaceae;g__Methanobrevibacter	91	0	0	112	84	35	0	192	0
d__Bacteria;p__Bacteroidota;c__Bacteroidia;o__Sphingobacteriales;f__Sphingobacteriaceae;__	650	395	526	112	768	661	576	563	149
d__Bacteria;p__Firmicutes;c__Bacilli;o__Lactobacillales;f__Streptococcaceae;g__Streptococcus	0	0	0	122	15	0	453	0	1052
d__Bacteria;p__Proteobacteria;c__Gammaproteobacteria;o__Enterobacterales;f__Enterobacteriaceae;__	3350	225	529	394	0	0	698	0	181
d__Bacteria;p__Firmicutes;c__Bacilli;o__Erysipelotrichales;f__Erysipelotrichaceae;g__Ileibacterium	36	19	115	558	307	75	131	223	20
d__Bacteria;p__Proteobacteria;c__Gammaproteobacteria;o__Burkholderiales;__;__	5064	2730	677	1141	1523	191	0	536	218
d__Bacteria;p__Bacteroidota;c__Bacteroidia;o__Chitinophagales;f__Chitinophagaceae;__	1379	276	747	291	654	493	0	564	735
d__Bacteria;p__Firmicutes;c__Bacilli;o__Erysipelotrichales;f__Erysipelotrichaceae;__	0	0	0	348	103	0	320	53	0
d__Bacteria;p__Patescibacteria;c__Saccharimonadia;o__Saccharimonadales;f__Saccharimonadales;g__Saccharimonadales	2615	175	711	0	551	182	343	198	5
d__Bacteria;p__Bacteroidota;c__Bacteroidia;o__Bacteroidales;f__Prevotellaceae;__	265	0	0	0	0	0	0	0	0
d__Bacteria;p__Fusobacteriota;c__Fusobacteriia;o__Fusobacteriales;f__Leptotrichiaceae;__	0	342	0	0	277	196	73	195	387
d__Bacteria;p__Proteobacteria;c__Gammaproteobacteria;o__Pasteurellales;f__Pasteurellaceae;g__Actinobacillus	0	42	19	40	40	6	0	10	34
d__Bacteria;p__Firmicutes;c__Bacilli;o__Mycoplasmatales;f__Mycoplasmataceae;g__Mycoplasma	528	0	363	0	221	217	0	158	171
d__Bacteria;p__Actinobacteriota;c__Actinobacteria;o__Corynebacteriales;f__Corynebacteriaceae;g__Corynebacterium	97	0	0	0	0	0	0	0	0
d__Bacteria;p__Firmicutes;c__Clostridia;o__Eubacteriales;f__Eubacteriaceae;g__uncultured	0	9	0	107	0	0	146	112	118
d__Bacteria;p__Proteobacteria;c__Gammaproteobacteria;o__Xanthomonadales;f__Xanthomonadaceae;g__Xanthomonas	138	99	59	0	167	0	0	0	63
d__Bacteria;p__Bacteroidota;c__Bacteroidia;o__Bacteroidales;f__Bacteroidaceae;g__Bacteroides	2411	0	0	0	0	148	266	103	0
d__Bacteria;p__Actinobacteriota;c__Actinobacteria;o__Micrococcales;__;__	0	0	0	0	0	160	48	94	0
d__Bacteria;p__Bacteroidota;c__Bacteroidia;o__Bacteroidales;f__Prevotellaceae;g__Prevotellaceae_UCG-001	0	0	9	29	0	0	0	0	0
**Total Count/Treatment**	**59920**	**10647**	**20325**	**12848**	**15668**	**16671**	**7014**	**18609**	**12435**

Supplementary File 5
Relative Abundance Data

9DPT NPL Genus Level Taxon Frequency	AC-PO7d	AC-10mg/kg	AC-5mg/kg	anti-rsPilA	anti-tip-chimer	Ofloxacin	TS-30mg/kg	TS-15mg/kg	Saline
d__Bacteria;p__Actinobacteriota;c__Actinobacteria;o__Actinomycetales;f__Actinomycetaceae;g__Mobiluncus	1085	203	1000	1125	1076	584	764	1211	1122
d__Bacteria;p__Actinobacteriota;c__Actinobacteria;o__Bifidobacteriales;f__Bifidobacteriaceae;g__Bifidobacterium	526	93	309	166	292	220	0	0	322
d__Bacteria;p__Campilobacterota;c__Campylobacteria;o__Campylobacterales;f__Campylobacteraceae;g__Campylobacter	3360	740	4152	3029	3609	1699	3914	3206	2660
d__Bacteria;p__Bacteroidota;c__Bacteroidia;o__Bacteroidales;__;__	2884	2900	8823	6492	4271	2368	96	5540	3127
d__Bacteria;p__Bacteroidota;c__Bacteroidia;o__Bacteroidales;f__Muribaculaceae;g__Muribaculaceae	32	8	150	268	617	0	260	299	215
d__Bacteria;p__Actinobacteriota;c__Coriobacteriia;o__Coriobacteriales;f__Atopobiaceae;g__Olsenella	12	0	25	0	0	0	0	0	0
d__Bacteria;p__Firmicutes;c__Clostridia;o__Peptostreptococcales-Tissierellales;f__Peptostreptococcales-Tissierellales;g__Peptoniphilus	379	0	0	0	62	0	0	51	0
d__Bacteria;p__Actinobacteriota;c__Actinobacteria;o__Actinomycetales;f__Actinomycetaceae;__	63	316	492	0	150	352	376	258	251
d__Bacteria;p__Firmicutes;c__Bacilli;o__Lactobacillales;f__Aerococcaceae;g__Aerococcus	0	0	0	0	129	0	322	328	0
d__Bacteria;p__Firmicutes;c__Bacilli;o__Staphylococcales;f__Staphylococcaceae;g__Staphylococcus	43767	3869	501	3872	2221	4353	428	666	1187
d__Bacteria;p__Fusobacteriota;c__Fusobacteriia;o__Fusobacteriales;f__Fusobacteriaceae;g__Fusobacterium	0	256	729	0	625	701	255	886	481
d__Bacteria;p__Proteobacteria;c__Gammaproteobacteria;o__Enterobacterales;f__Enterobacteriaceae;g__Escherichia-Shigella	11592	0	0	0	0	211	414	140	0
d__Archaea;p__Euryarchaeota;c__Methanobacteria;o__Methanobacteriales;f__Methanobacteriaceae;g__Methanobrevibacter	0	8	0	0	42	0	0	15	0
d__Bacteria;p__Bacteroidota;c__Bacteroidia;o__Sphingobacteriales;f__Sphingobacteriaceae;__	456	607	600	199	1017	457	1422	551	66
d__Bacteria;p__Firmicutes;c__Bacilli;o__Lactobacillales;f__Streptococcaceae;g__Streptococcus	97	0	0	189	0	0	273	0	181
d__Bacteria;p__Proteobacteria;c__Gammaproteobacteria;o__Enterobacterales;f__Enterobacteriaceae;__	236	301	770	489	0	0	594	0	255
d__Bacteria;p__Firmicutes;c__Bacilli;o__Erysipelotrichales;f__Erysipelotrichaceae;g__Ileibacterium	15	0	101	287	447	52	36	444	0
d__Bacteria;p__Proteobacteria;c__Gammaproteobacteria;o__Burkholderiales;__;__	1516	2635	865	1623	2315	95	0	306	268
d__Bacteria;p__Bacteroidota;c__Bacteroidia;o__Chitinophagales;f__Chitinophagaceae;__	1185	221	803	498	705	382	5	578	825
d__Bacteria;p__Firmicutes;c__Bacilli;o__Erysipelotrichales;f__Erysipelotrichaceae;__	0	0	0	105	398	0	261	103	218
d__Bacteria;p__Patescibacteria;c__Saccharimonadia;o__Saccharimonadales;f__Saccharimonadales;g__Saccharimonadales	2718	75	634	0	742	0	648	328	0
d__Bacteria;p__Bacteroidota;c__Bacteroidia;o__Bacteroidales;f__Prevotellaceae;__	168	0	57	0	0	0	0	59	0
d__Bacteria;p__Fusobacteriota;c__Fusobacteriia;o__Fusobacteriales;f__Leptotrichiaceae;__	0	378	0	0	318	156	157	393	395
d__Bacteria;p__Proteobacteria;c__Gammaproteobacteria;o__Pasteurellales;f__Pasteurellaceae;g__Actinobacillus	28	0	137	0	190	0	8	44	0
d__Bacteria;p__Firmicutes;c__Bacilli;o__Mycoplasmatales;f__Mycoplasmataceae;g__Mycoplasma	326	25	343	0	350	159	213	354	143
d__Bacteria;p__Actinobacteriota;c__Actinobacteria;o__Corynebacteriales;f__Corynebacteriaceae;g__Corynebacterium	200	0	0	0	0	0	55	142	0
d__Bacteria;p__Firmicutes;c__Clostridia;o__Eubacteriales;f__Eubacteriaceae;g__uncultured	0	0	14	0	21	0	97	236	42
d__Bacteria;p__Proteobacteria;c__Gammaproteobacteria;o__Xanthomonadales;f__Xanthomonadaceae;g__Xanthomonas	0	249	113	0	399	19	60	36	57
d__Bacteria;p__Bacteroidota;c__Bacteroidia;o__Bacteroidales;f__Bacteroidaceae;g__Bacteroides	835	21	0	0	0	50	264	259	0
d__Bacteria;p__Firmicutes;c__Clostridia;o__Lachnospirales;f__Lachnospiraceae;__	0	0	0	0	139	0	0	0	0
d__Bacteria;p__Actinobacteriota;c__Actinobacteria;o__Micrococcales;__;__	0	0	0	0	67	0	0	84	0
d__Bacteria;p__Bacteroidota;c__Bacteroidia;o__Bacteroidales;f__Prevotellaceae;g__Prevotellaceae_UCG-001	0	0	0	0	0	0	0	130	15
**Total Count/Treatment**	**71480**	**12905**	**20618**	**18342**	**20202**	**11858**	**10922**	**16647**	**11830**

Supplementary File 5
Relative Abundance Data

Baseline Fecal Genus Level Taxon Relative Abundance	AC-PO7d	AC-10mg/kg	AC-5mg/kg	anti-rsPilA	anti-tip-chime	Ofloxacin	TS-30mg/kg	TS-15mg/kg	Saline
d__Archaea;p__Euryarchaeota;c__Methanobacteria;o__Methanobacteriales;f__Methanobacteriaceae;g__Methanobrevibacter	6.021%	10.409%	1.074%	3.986%	7.640%	17.860%	2.158%	3.244%	1.910%
d__Archaea;p__Euryarchaeota;c__Methanobacteria;o__Methanobacteriales;f__Methanobacteriaceae;g__Methanosphaera	1.018%	0.677%	1.476%	0.000%	0.007%	0.404%	0.190%	0.382%	0.759%
d__Bacteria;p__Actinobacteriota;c__Actinobacteria;o__Actinomycetales;f__Actinomycetaceae;g__Actinomyces	0.011%	0.543%	0.093%	0.093%	0.077%	0.024%	0.167%	0.233%	0.000%
d__Bacteria;p__Actinobacteriota;c__Actinobacteria;o__Actinomycetales;f__Actinomycetaceae;g__Actinomycetaceae	0.000%	0.000%	0.000%	0.000%	0.000%	0.000%	0.000%	0.000%	0.036%
d__Bacteria;p__Actinobacteriota;c__Actinobacteria;o__Bifidobacteriales;f__Bifidobacteriaceae;g__Bifidobacterium	2.651%	0.000%	2.196%	1.995%	0.024%	0.197%	0.650%	0.935%	4.449%
d__Bacteria;p__Actinobacteriota;c__Coriobacteriia;o__Coriobacteriales;__;__	0.213%	0.036%	0.324%	0.197%	0.309%	0.858%	0.113%	0.052%	0.039%
d__Bacteria;p__Actinobacteriota;c__Coriobacteriia;o__Coriobacteriales;f__Atopobiaceae;__	0.951%	1.347%	2.118%	0.928%	1.261%	0.568%	1.557%	1.827%	0.883%
d__Bacteria;p__Actinobacteriota;c__Coriobacteriia;o__Coriobacteriales;f__Atopobiaceae;g__uncultured	0.084%	0.234%	0.104%	0.176%	0.097%	0.057%	0.071%	0.045%	0.061%
d__Bacteria;p__Actinobacteriota;c__Coriobacteriia;o__Coriobacteriales;f__Coriobacteriaceae;g__Collinsella	0.000%	0.000%	0.000%	0.000%	0.000%	0.000%	0.000%	0.000%	0.036%
d__Bacteria;p__Actinobacteriota;c__Coriobacteriia;o__Coriobacteriales;f__Coriobacteriales_Incertae_Sedis;g__uncultured	0.104%	0.091%	0.039%	0.118%	0.000%	0.233%	0.145%	0.311%	0.293%
d__Bacteria;p__Actinobacteriota;c__Coriobacteriia;o__Coriobacteriales;f__Eggerthellaceae;__	0.238%	1.298%	0.140%	0.643%	0.399%	0.712%	0.170%	0.747%	0.262%
d__Bacteria;p__Actinobacteriota;c__Coriobacteriia;o__Coriobacteriales;f__Eggerthellaceae;g__Adlercreutzia	0.036%	0.000%	0.000%	0.113%	0.159%	0.000%	0.080%	0.000%	0.000%
d__Bacteria;p__Actinobacteriota;c__Coriobacteriia;o__Coriobacteriales;f__Eggerthellaceae;g__Enterorhabdus	0.744%	2.252%	0.725%	0.868%	1.177%	1.026%	0.737%	0.982%	0.486%
d__Bacteria;p__Actinobacteriota;c__Coriobacteriia;o__Coriobacteriales;f__Eggerthellaceae;g__uncultured	0.414%	1.493%	0.285%	2.854%	1.372%	0.888%	0.672%	1.091%	0.635%
d__Bacteria;p__Actinobacteriota;c__Coriobacteriia;o__Coriobacteriales;f__uncultured;g__uncultured	0.098%	0.098%	0.023%	0.030%	0.058%	0.197%	0.145%	0.337%	0.218%
d__Bacteria;p__Bacteroidota;c__Bacteroidia;o__Bacteroidales;__;__	0.789%	0.036%	0.243%	0.530%	0.244%	0.535%	0.618%	0.377%	0.240%
d__Bacteria;p__Bacteroidota;c__Bacteroidia;o__Bacteroidales;f__Bacteroidaceae;g__Bacteroides	1.714%	0.511%	0.816%	1.951%	0.621%	0.538%	1.135%	1.001%	0.742%
d__Bacteria;p__Bacteroidota;c__Bacteroidia;o__Bacteroidales;f__Marinifilaceae;g__Butyricimonas	0.058%	0.000%	0.039%	0.019%	0.041%	0.000%	0.016%	0.069%	0.052%
d__Bacteria;p__Bacteroidota;c__Bacteroidia;o__Bacteroidales;f__Marinifilaceae;g__Odoribacter	0.051%	0.000%	0.000%	0.000%	0.000%	0.000%	0.000%	0.183%	0.000%
d__Bacteria;p__Bacteroidota;c__Bacteroidia;o__Bacteroidales;f__Muribaculaceae;__	0.138%	1.285%	0.000%	0.343%	0.000%	0.000%	0.174%	0.000%	0.174%
d__Bacteria;p__Bacteroidota;c__Bacteroidia;o__Bacteroidales;f__Muribaculaceae;g__Muribaculaceae	25.141%	9.846%	13.901%	18.191%	20.307%	11.486%	25.572%	17.560%	16.246%
d__Bacteria;p__Bacteroidota;c__Bacteroidia;o__Bacteroidales;f__Muribaculaceae;g__Muribaculum	0.208%	0.000%	0.036%	0.062%	0.000%	0.036%	0.048%	0.220%	0.000%
d__Bacteria;p__Bacteroidota;c__Bacteroidia;o__Bacteroidales;f__Prevotellaceae;g__Prevotella	3.778%	1.435%	1.030%	0.643%	0.701%	2.222%	2.531%	0.524%	2.216%
d__Bacteria;p__Bacteroidota;c__Bacteroidia;o__Bacteroidales;f__Prevotellaceae;g__Prevotellaceae_Ga6A1_group	0.170%	0.000%	0.060%	0.088%	0.201%	0.173%	0.476%	0.054%	0.240%
d__Bacteria;p__Bacteroidota;c__Bacteroidia;o__Bacteroidales;f__Prevotellaceae;g__Prevotellaceae_NK3B31_group	0.387%	0.198%	0.023%	0.109%	0.072%	0.631%	0.264%	0.014%	0.160%
d__Bacteria;p__Bacteroidota;c__Bacteroidia;o__Bacteroidales;f__Prevotellaceae;g__Prevotellaceae_UCG-001	2.354%	2.408%	0.575%	0.333%	0.164%	2.112%	3.297%	2.722%	2.683%
d__Bacteria;p__Bacteroidota;c__Bacteroidia;o__Bacteroidales;f__Prevotellaceae;g__Prevotellaceae_UCG-003	0.085%	0.000%	0.057%	0.299%	0.145%	0.595%	0.000%	0.000%	0.000%
d__Bacteria;p__Bacteroidota;c__Bacteroidia;o__Bacteroidales;f__Rikenellaceae;g__Alistipes	0.176%	0.039%	0.166%	0.368%	0.259%	0.278%	0.457%	0.245%	0.177%
d__Bacteria;p__Bacteroidota;c__Bacteroidia;o__Bacteroidales;f__Rikenellaceae;g__dgA-11_gut_group	0.194%	0.046%	0.098%	0.012%	0.118%	0.039%	0.167%	0.050%	0.058%
d__Bacteria;p__Bacteroidota;c__Bacteroidia;o__Bacteroidales;f__Rikenellaceae;g__Rikenellaceae_RC9_gut_group	0.210%	0.010%	0.041%	0.000%	0.097%	0.054%	0.199%	0.086%	0.188%
d__Bacteria;p__Bacteroidota;c__Bacteroidia;o__Bacteroidales;f__Tannerellaceae;g__Parabacteroides	0.404%	0.104%	0.179%	0.252%	0.145%	0.404%	0.129%	0.304%	0.389%
d__Bacteria;p__Desulfobacterota;c__Desulfovibrionia;o__Desulfovibrionales;f__Desulfovibrionaceae;g__Bilophila	0.000%	0.000%	0.000%	0.000%	0.000%	0.000%	0.000%	0.199%	0.000%
d__Bacteria;p__Desulfobacterota;c__Desulfovibrionia;o__Desulfovibrionales;f__Desulfovibrionaceae;g__Desulfovibrio	0.097%	0.042%	0.202%	0.000%	0.000%	0.000%	0.000%	0.000%	0.204%
d__Bacteria;p__Elusimicrobiota;c__Elusimicrobia;o__Elusimicrobiales;f__Elusimicrobiaceae;g__Elusimicrobium	0.665%	0.000%	0.308%	0.532%	0.203%	0.239%	0.228%	0.175%	0.362%
d__Bacteria;p__Firmicutes;c__Bacilli;o__Erysipelotrichales;f__Erysipelatoclostridiaceae;__	0.064%	0.000%	0.000%	0.222%	0.072%	0.000%	0.174%	0.000%	0.000%
d__Bacteria;p__Firmicutes;c__Bacilli;o__Erysipelotrichales;f__Erysipelatoclostridiaceae;g__Erysipelatoclostridiaceae	0.027%	0.029%	0.000%	0.162%	0.082%	0.000%	0.000%	0.035%	0.000%
d__Bacteria;p__Firmicutes;c__Bacilli;o__Erysipelotrichales;f__Erysipelotrichaceae;__	7.153%	23.508%	8.575%	9.330%	8.522%	8.967%	10.467%	14.865%	8.603%
d__Bacteria;p__Firmicutes;c__Bacilli;o__Erysipelotrichales;f__Erysipelotrichaceae;g__Allobaculum	0.340%	0.420%	7.174%	1.099%	2.641%	0.296%	0.084%	0.709%	2.051%
d__Bacteria;p__Firmicutes;c__Bacilli;o__Erysipelotrichales;f__Erysipelotrichaceae;g__Dubosiella	0.194%	0.000%	0.000%	0.000%	0.000%	0.060%	0.000%	2.550%	0.756%
d__Bacteria;p__Firmicutes;c__Bacilli;o__Erysipelotrichales;f__Erysipelotrichaceae;g__Faecalibaculum	0.091%	0.081%	0.202%	0.134%	0.019%	0.024%	0.084%	0.000%	0.052%
d__Bacteria;p__Firmicutes;c__Bacilli;o__Erysipelotrichales;f__Erysipelotrichaceae;g__Ileibacterium	1.132%	0.813%	10.721%	3.164%	4.907%	1.421%	0.277%	3.447%	5.705%
d__Bacteria;p__Firmicutes;c__Bacilli;o__Erysipelotrichales;f__Erysipelotrichaceae;g__uncultured	2.157%	2.805%	11.146%	3.236%	3.431%	1.941%	1.058%	1.388%	3.627%
d__Bacteria;p__Firmicutes;c__Bacilli;o__Lactobacillales;f__Lactobacillaceae;g__Lactobacillus	0.281%	0.000%	1.235%	1.444%	0.181%	0.045%	0.000%	0.354%	2.393%
d__Bacteria;p__Firmicutes;c__Bacilli;o__Lactobacillales;f__Streptococcaceae;g__Streptococcus	0.397%	0.114%	0.194%	0.875%	0.507%	0.505%	0.193%	0.500%	1.510%
d__Bacteria;p__Firmicutes;c__Bacilli;o__RF39;f__RF39;g__RF39	0.147%	0.137%	0.041%	0.586%	0.430%	0.251%	0.550%	0.691%	0.433%
d__Bacteria;p__Firmicutes;c__Clostridia;o__Christensenellales;f__Christensenellaceae;__	0.008%	0.000%	0.000%	0.000%	0.000%	0.000%	0.000%	0.000%	0.000%

Supplementary File 5
Relative Abundance Data

Baseline Fecal Genus Level Taxon Relative Abundance Continued	AC-PO7d	AC-10mg/kg	AC-5mg/kg	anti-rsPilA	anti-tip-chime	Ofloxacin	TS-30mg/kg	TS-15mg/kg	Saline
d__Bacteria;p__Firmicutes;c__Clostridia;o__Christensenellales;f__Christensenellaceae;g__Christensenellaceae_R-7_group	2.688%	5.255%	1.012%	6.946%	3.518%	2.249%	1.129%	5.834%	1.893%
d__Bacteria;p__Firmicutes;c__Clostridia;o__Clostridia;f__Hungateiclostridiaceae;g__Ruminiclostridium	0.097%	0.068%	0.036%	0.035%	0.130%	0.000%	0.000%	0.119%	0.000%
d__Bacteria;p__Firmicutes;c__Clostridia;o__Clostridia_UCG-014;f__Clostridia_UCG-014;g__Clostridia_UCG-014	1.433%	1.659%	0.689%	1.044%	1.090%	1.134%	2.017%	0.911%	0.676%
d__Bacteria;p__Firmicutes;c__Clostridia;o__Eubacteriales;f__Eubacteriaceae;g__uncultured	2.951%	6.644%	4.075%	3.657%	4.540%	1.460%	1.653%	6.164%	6.337%
d__Bacteria;p__Firmicutes;c__Clostridia;o__Lachnospirales;f__Defluviitaleaceae;g__Defluviitaleaceae_UCG-011	0.140%	0.159%	0.280%	0.023%	0.193%	0.254%	0.199%	0.330%	0.190%
d__Bacteria;p__Firmicutes;c__Clostridia;o__Lachnospirales;f__Lachnospiraceae;__	9.138%	2.971%	2.434%	3.456%	9.389%	1.430%	2.776%	3.175%	3.301%
d__Bacteria;p__Firmicutes;c__Clostridia;o__Lachnospirales;f__Lachnospiraceae;g__[Eubacterium]_ruminantium_group	0.010%	0.000%	0.161%	0.391%	0.041%	0.981%	2.010%	1.200%	0.751%
d__Bacteria;p__Firmicutes;c__Clostridia;o__Lachnospirales;f__Lachnospiraceae;g__[Ruminococcus]_torques_group	0.008%	0.000%	0.000%	0.000%	0.162%	0.057%	0.100%	0.118%	0.000%
d__Bacteria;p__Firmicutes;c__Clostridia;o__Lachnospirales;f__Lachnospiraceae;g__Blautia	0.542%	0.241%	0.311%	0.861%	0.667%	0.081%	0.283%	0.465%	0.359%
d__Bacteria;p__Firmicutes;c__Clostridia;o__Lachnospirales;f__Lachnospiraceae;g__Coprococcus	0.064%	0.000%	0.150%	0.944%	0.080%	0.182%	0.093%	0.290%	0.050%
d__Bacteria;p__Firmicutes;c__Clostridia;o__Lachnospirales;f__Lachnospiraceae;g__Dorea	0.198%	0.046%	0.039%	0.407%	0.094%	0.000%	0.000%	0.045%	0.000%
d__Bacteria;p__Firmicutes;c__Clostridia;o__Lachnospirales;f__Lachnospiraceae;g__Frisingicoccus	0.000%	0.000%	0.000%	0.231%	0.169%	0.000%	0.000%	0.247%	0.047%
d__Bacteria;p__Firmicutes;c__Clostridia;o__Lachnospirales;f__Lachnospiraceae;g__Lachnoclostridium	0.232%	0.267%	0.251%	1.854%	0.553%	0.000%	0.675%	1.308%	1.237%
d__Bacteria;p__Firmicutes;c__Clostridia;o__Lachnospirales;f__Lachnospiraceae;g__Lachnospira	0.007%	0.000%	0.000%	0.484%	0.140%	0.000%	0.055%	0.000%	0.000%
d__Bacteria;p__Firmicutes;c__Clostridia;o__Lachnospirales;f__Lachnospiraceae;g__Lachnospiraceae_NK4A136_group	1.138%	0.456%	0.057%	0.380%	1.046%	0.000%	0.849%	0.493%	1.010%
d__Bacteria;p__Firmicutes;c__Clostridia;o__Lachnospirales;f__Lachnospiraceae;g__Lachnospiraceae_UCG-001	0.073%	0.000%	0.181%	0.225%	0.838%	0.042%	0.228%	0.000%	0.309%
d__Bacteria;p__Firmicutes;c__Clostridia;o__Lachnospirales;f__Lachnospiraceae;g__Lachnospiraceae_UCG-004	0.124%	0.000%	0.054%	0.000%	0.094%	0.000%	0.035%	0.000%	0.000%
d__Bacteria;p__Firmicutes;c__Clostridia;o__Lachnospirales;f__Lachnospiraceae;g__Lachnospiraceae_UCG-010	0.138%	0.000%	0.000%	0.354%	0.070%	0.000%	0.000%	0.052%	0.000%
d__Bacteria;p__Firmicutes;c__Clostridia;o__Lachnospirales;f__Lachnospiraceae;g__Marvinbryantia	0.180%	0.290%	0.000%	0.275%	0.249%	0.338%	0.000%	0.000%	0.000%
d__Bacteria;p__Firmicutes;c__Clostridia;o__Lachnospirales;f__Lachnospiraceae;g__uncultured	0.396%	0.033%	0.497%	0.285%	0.022%	0.054%	0.894%	0.283%	0.188%
d__Bacteria;p__Firmicutes;c__Clostridia;o__Monoglobales;f__Monoglobaceae;g__Monoglobus	0.863%	2.476%	0.585%	0.956%	0.913%	1.618%	1.193%	0.392%	0.442%
d__Bacteria;p__Firmicutes;c__Clostridia;o__Oscillospirales;__;__	0.088%	0.446%	0.000%	0.093%	0.019%	0.105%	0.187%	0.150%	0.152%
d__Bacteria;p__Firmicutes;c__Clostridia;o__Oscillospirales;f__[Eubacterium]_coprostanoligenes_group;g__[Eubacterium]_coprostanoligenes_group	0.324%	0.101%	0.140%	1.648%	0.829%	0.371%	0.466%	1.063%	0.229%
d__Bacteria;p__Firmicutes;c__Clostridia;o__Oscillospirales;f__Butyricicoccaceae;g__UCG-008	0.175%	0.120%	0.000%	0.354%	0.490%	0.000%	0.116%	0.031%	0.077%
d__Bacteria;p__Firmicutes;c__Clostridia;o__Oscillospirales;f__Oscillospiraceae;__	0.825%	0.745%	0.321%	0.435%	0.058%	0.840%	0.592%	0.757%	0.298%
d__Bacteria;p__Firmicutes;c__Clostridia;o__Oscillospirales;f__Oscillospiraceae;g__Colidextribacter	0.372%	0.296%	0.559%	0.456%	0.517%	0.117%	1.049%	0.368%	0.505%
d__Bacteria;p__Firmicutes;c__Clostridia;o__Oscillospirales;f__Oscillospiraceae;g__NK4A214_group	0.347%	0.130%	0.432%	0.421%	0.715%	0.278%	0.074%	0.494%	0.483%
d__Bacteria;p__Firmicutes;c__Clostridia;o__Oscillospirales;f__Oscillospiraceae;g__Oscillibacter	0.063%	0.000%	0.000%	0.069%	0.097%	0.048%	0.116%	0.040%	0.138%
d__Bacteria;p__Firmicutes;c__Clostridia;o__Oscillospirales;f__Oscillospiraceae;g__Papillibacter	0.102%	0.000%	0.000%	0.076%	0.176%	0.000%	0.138%	0.048%	0.000%
d__Bacteria;p__Firmicutes;c__Clostridia;o__Oscillospirales;f__Oscillospiraceae;g__UCG-005	2.506%	0.817%	0.546%	1.625%	1.911%	1.158%	2.355%	0.869%	1.350%
d__Bacteria;p__Firmicutes;c__Clostridia;o__Oscillospirales;f__Oscillospiraceae;g__uncultured	0.746%	1.070%	0.782%	3.581%	0.734%	0.787%	2.393%	1.561%	1.005%
d__Bacteria;p__Firmicutes;c__Clostridia;o__Oscillospirales;f__Ruminococcaceae;__	0.911%	0.042%	0.148%	0.139%	0.000%	0.242%	0.122%	0.131%	0.000%
d__Bacteria;p__Firmicutes;c__Clostridia;o__Oscillospirales;f__Ruminococcaceae;g__[Eubacterium]_siraeum_group	0.325%	0.000%	0.111%	0.000%	0.000%	0.000%	0.000%	0.000%	0.017%
d__Bacteria;p__Firmicutes;c__Clostridia;o__Oscillospirales;f__Ruminococcaceae;g__Incertae_Sedis	1.323%	1.503%	1.502%	2.060%	0.780%	2.154%	1.325%	2.012%	1.551%
d__Bacteria;p__Firmicutes;c__Clostridia;o__Oscillospirales;f__Ruminococcaceae;g__Ruminococcaceae	0.058%	0.179%	0.088%	0.000%	0.034%	0.275%	0.132%	0.000%	0.712%
d__Bacteria;p__Firmicutes;c__Clostridia;o__Oscillospirales;f__Ruminococcaceae;g__Ruminococcus	2.210%	2.883%	1.895%	1.778%	1.984%	2.713%	5.356%	2.843%	2.989%
d__Bacteria;p__Firmicutes;c__Clostridia;o__Oscillospirales;f__Ruminococcaceae;g__UBA1819	0.014%	0.000%	0.000%	0.025%	0.000%	0.000%	0.000%	0.000%	0.000%
d__Bacteria;p__Firmicutes;c__Clostridia;o__Peptostreptococcales-Tissierellales;f__Anaerovoracaceae;g__[Eubacterium]_brachy_group	0.072%	0.189%	0.000%	0.160%	0.039%	0.045%	0.000%	0.263%	0.091%
d__Bacteria;p__Firmicutes;c__Clostridia;o__Peptostreptococcales-Tissierellales;f__Anaerovoracaceae;g__[Eubacterium]_nodatum_group	0.238%	0.052%	0.243%	0.262%	0.507%	0.290%	0.051%	0.159%	0.000%
d__Bacteria;p__Firmicutes;c__Clostridia;o__Peptostreptococcales-Tissierellales;f__Anaerovoracaceae;g__Family_XIII_AD3011_group	0.164%	0.283%	0.243%	1.491%	0.478%	0.144%	0.061%	0.505%	0.130%
d__Bacteria;p__Firmicutes;c__Clostridia;o__Peptostreptococcales-Tissierellales;f__Anaerovoracaceae;g__Mogibacterium	0.185%	0.355%	0.106%	0.618%	0.536%	0.126%	0.122%	1.148%	0.182%
d__Bacteria;p__Firmicutes;c__Negativicutes;o__Acidaminococcales;f__Acidaminococcaceae;g__Phascolarctobacterium	0.832%	1.376%	0.593%	0.424%	0.348%	1.083%	0.457%	0.650%	0.836%
d__Bacteria;p__Fusobacteriota;c__Fusobacteriia;o__Fusobacteriales;f__Fusobacteriaceae;g__Fusobacterium	0.004%	0.000%	0.000%	0.000%	0.000%	0.000%	0.000%	0.847%	0.006%
d__Bacteria;p__Patescibacteria;c__Saccharimonadia;o__Saccharimonadales;f__Saccharimonadaceae;g__Candidatus_Saccharimonas	2.495%	5.170%	4.847%	3.916%	2.319%	19.612%	8.302%	4.492%	5.275%
d__Bacteria;p__Planctomycetota;c__Planctomycetes;o__Pirellulales;f__Pirellulaceae;g__p-1088-a5_gut_group	0.116%	0.381%	0.132%	0.079%	0.220%	0.242%	0.113%	0.423%	0.116%
d__Bacteria;p__Proteobacteria;c__Gammaproteobacteria;o__Burkholderiales;f__Sutterellaceae;g__Parasutterella	0.173%	0.221%	0.220%	0.329%	0.317%	0.072%	0.119%	0.180%	0.309%
d__Bacteria;p__Proteobacteria;c__Gammaproteobacteria;o__Enterobacterales;f__Enterobacteriaceae;g__Escherichia-Shigella	0.000%	0.000%	0.041%	0.056%	0.331%	0.000%	3.011%	0.451%	0.190%
d__Bacteria;p__Proteobacteria;c__Gammaproteobacteria;o__Pasteurellales;f__Pasteurellaceae;g__Haemophilus	0.000%	0.000%	0.000%	0.056%	0.232%	0.000%	0.000%	0.029%	0.000%
d__Bacteria;p__Spirochaetota;c__Spirochaetia;o__Spirochaetales;f__Spirochaetaceae;g__Treponema	3.720%	0.540%	3.014%	1.055%	0.802%	3.147%	4.101%	0.482%	0.210%
d__Bacteria;p__Verrucomicrobiota;c__Verrucomicrobiae;o__Verrucomicrobiales;f__Akkermansiaceae;g__Akkermansia	0.000%	0.192%	7.565%	0.000%	4.835%	0.395%	0.000%	0.067%	7.030%

Supplementary File 5
Relative Abundance Data

2DPT Fecal Genus Level Taxon Relative Abundance	AC-PO7d	AC-10mg/kg	AC-5mg/kg	anti-rsPil/	anti-tip-chime	Ofloxacin	TS-30mg/kg	TS-15mg/kg	Saline
d__Archaea;p__Euryarchaeota;c__Methanobacteria;o__Methanobacteriales;f__Methanobacteriaceae;g__Methanobrevibacter	6.748%	27.755%	11.152%	8.453%	9.116%	11.062%	4.181%	6.039%	0.284%
d__Archaea;p__Euryarchaeota;c__Methanobacteria;o__Methanobacteriales;f__Methanobacteriaceae;g__Methanosphaera	0.009%	1.634%	1.036%	0.000%	0.000%	0.969%	0.271%	0.190%	0.038%
d__Bacteria;p__Actinobacteriota;c__Actinobacteria;o__Actinomycetales;f__Actinomycetaceae;g__Actinomyces	0.000%	2.063%	0.102%	0.168%	0.018%	0.000%	0.103%	0.010%	0.000%
d__Bacteria;p__Actinobacteriota;c__Actinobacteria;o__Actinomycetales;f__Actinomycetaceae;g__Actinomycetaceae	0.114%	0.223%	0.000%	0.000%	0.000%	0.000%	0.000%	0.000%	0.041%
d__Bacteria;p__Actinobacteriota;c__Actinobacteria;o__Bifidobacteriales;f__Bifidobacteriaceae;g__Bifidobacterium	0.009%	0.435%	2.214%	1.446%	0.092%	1.763%	0.495%	3.294%	3.905%
d__Bacteria;p__Actinobacteriota;c__Coriobacteriia;o__Coriobacteriales;__;__	0.047%	0.133%	0.198%	0.153%	0.043%	0.156%	0.086%	0.079%	0.000%
d__Bacteria;p__Actinobacteriota;c__Coriobacteriia;o__Coriobacteriales;f__Atopobiaceae;__	0.047%	1.183%	3.046%	1.168%	0.819%	0.889%	1.737%	0.992%	0.894%
d__Bacteria;p__Actinobacteriota;c__Coriobacteriia;o__Coriobacteriales;f__Atopobiaceae;g__uncultured	0.000%	0.110%	0.237%	0.127%	0.071%	0.096%	0.074%	0.049%	0.248%
d__Bacteria;p__Actinobacteriota;c__Coriobacteriia;o__Coriobacteriales;f__Coriobacteriaceae;g__Collinsella	0.011%	0.585%	0.000%	0.000%	0.000%	0.000%	0.000%	0.000%	0.076%
d__Bacteria;p__Actinobacteriota;c__Coriobacteriia;o__Coriobacteriales;f__Coriobacteriales_Incertae_Sedis;g__uncultured	0.007%	0.678%	0.317%	0.136%	0.117%	0.233%	0.064%	0.032%	0.178%
d__Bacteria;p__Actinobacteriota;c__Coriobacteriia;o__Coriobacteriales;f__Eggerthellaceae;__	0.000%	0.452%	0.377%	0.321%	0.243%	0.252%	0.993%	0.539%	0.351%
d__Bacteria;p__Actinobacteriota;c__Coriobacteriia;o__Coriobacteriales;f__Eggerthellaceae;g__Adlercreutzia	0.000%	0.000%	0.000%	0.103%	0.117%	0.099%	0.121%	0.000%	0.000%
d__Bacteria;p__Actinobacteriota;c__Coriobacteriia;o__Coriobacteriales;f__Eggerthellaceae;g__Enterorhabdus	0.056%	1.857%	1.308%	0.814%	0.814%	0.526%	1.081%	0.411%	0.976%
d__Bacteria;p__Actinobacteriota;c__Coriobacteriia;o__Coriobacteriales;f__Eggerthellaceae;g__Paraeggerthella	0.000%	0.000%	0.044%	0.000%	0.000%	0.000%	0.000%	0.000%	0.000%
d__Bacteria;p__Actinobacteriota;c__Coriobacteriia;o__Coriobacteriales;f__Eggerthellaceae;g__uncultured	0.008%	0.183%	0.551%	4.010%	0.687%	1.285%	1.946%	0.821%	1.008%
d__Bacteria;p__Actinobacteriota;c__Coriobacteriia;o__Coriobacteriales;f__uncultured;g__uncultured	0.000%	0.256%	0.129%	0.013%	0.107%	0.000%	0.143%	0.148%	0.155%
d__Bacteria;p__Bacteroidota;c__Bacteroidia;o__Bacteroidales;__;__	0.029%	0.023%	0.000%	0.106%	0.202%	0.341%	0.224%	0.230%	0.418%
d__Bacteria;p__Bacteroidota;c__Bacteroidia;o__Bacteroidales;f__Bacteroidaceae;g__Bacteroides	36.428%	3.528%	2.090%	3.506%	1.070%	1.508%	1.308%	1.039%	1.172%
d__Bacteria;p__Bacteroidota;c__Bacteroidia;o__Bacteroidales;f__Marinifilaceae;g__Butyricimonas	0.103%	0.000%	0.000%	0.164%	0.000%	0.070%	0.000%	0.143%	0.082%
d__Bacteria;p__Bacteroidota;c__Bacteroidia;o__Bacteroidales;f__Marinifilaceae;g__Odoribacter	4.287%	0.000%	0.000%	0.000%	0.008%	0.000%	0.000%	0.000%	0.000%
d__Bacteria;p__Bacteroidota;c__Bacteroidia;o__Bacteroidales;f__Muribaculaceae;__	0.000%	0.000%	0.025%	0.231%	0.000%	0.000%	0.190%	0.000%	0.000%
d__Bacteria;p__Bacteroidota;c__Bacteroidia;o__Bacteroidales;f__Muribaculaceae;g__Muribaculaceae	0.144%	2.269%	5.181%	15.093%	17.834%	14.339%	10.714%	12.455%	16.805%
d__Bacteria;p__Bacteroidota;c__Bacteroidia;o__Bacteroidales;f__Muribaculaceae;g__Muribaculum	0.000%	0.000%	0.000%	0.058%	0.000%	0.000%	0.000%	0.000%	0.000%
d__Bacteria;p__Bacteroidota;c__Bacteroidia;o__Bacteroidales;f__Prevotellaceae;g__Prevotella	0.000%	0.113%	0.543%	0.224%	1.455%	1.431%	4.543%	1.608%	2.417%
d__Bacteria;p__Bacteroidota;c__Bacteroidia;o__Bacteroidales;f__Prevotellaceae;g__Prevotellaceae_Ga6A1_group	0.000%	0.000%	0.000%	0.000%	0.294%	0.147%	0.576%	0.134%	0.865%
d__Bacteria;p__Bacteroidota;c__Bacteroidia;o__Bacteroidales;f__Prevotellaceae;g__Prevotellaceae_NK3B31_group	0.000%	0.106%	0.000%	0.024%	0.189%	0.357%	0.049%	0.092%	0.254%
d__Bacteria;p__Bacteroidota;c__Bacteroidia;o__Bacteroidales;f__Prevotellaceae;g__Prevotellaceae_UCG-001	0.000%	0.123%	0.187%	0.179%	3.970%	4.842%	4.560%	0.994%	4.627%
d__Bacteria;p__Bacteroidota;c__Bacteroidia;o__Bacteroidales;f__Prevotellaceae;g__Prevotellaceae_UCG-003	0.000%	0.000%	0.000%	0.013%	0.092%	0.179%	0.000%	0.000%	0.000%
d__Bacteria;p__Bacteroidota;c__Bacteroidia;o__Bacteroidales;f__Rikenellaceae;g__Alistipes	0.189%	0.126%	0.019%	0.136%	0.202%	0.000%	0.362%	0.415%	0.313%
d__Bacteria;p__Bacteroidota;c__Bacteroidia;o__Bacteroidales;f__Rikenellaceae;g__dgA-11_gut_group	0.000%	0.017%	0.000%	0.000%	0.036%	0.000%	0.254%	0.168%	0.096%
d__Bacteria;p__Bacteroidota;c__Bacteroidia;o__Bacteroidales;f__Rikenellaceae;g__Rikenellaceae_RC9_gut_group	0.000%	0.000%	0.000%	0.000%	0.360%	0.000%	0.338%	0.000%	0.479%
d__Bacteria;p__Bacteroidota;c__Bacteroidia;o__Bacteroidales;f__Tannerellaceae;g__Parabacteroides	2.194%	1.116%	1.776%	0.946%	0.153%	0.210%	0.439%	0.223%	0.298%
d__Bacteria;p__Desulfobacterota;c__Desulfovibrionia;o__Desulfovibrionales;f__Desulfovibrionaceae;g__Bilophila	2.768%	0.043%	0.000%	0.000%	0.000%	0.010%	0.052%	0.040%	0.091%
d__Bacteria;p__Desulfobacterota;c__Desulfovibrionia;o__Desulfovibrionales;f__Desulfovibrionaceae;g__Desulfovibrio	0.000%	0.000%	0.000%	0.000%	0.036%	0.000%	0.000%	0.000%	0.129%
d__Bacteria;p__Elusimicrobiota;c__Elusimicrobia;o__Elusimicrobiales;f__Elusimicrobiaceae;g__Elusimicrobium	0.000%	0.073%	0.030%	0.050%	0.087%	0.083%	0.409%	0.007%	0.088%
d__Bacteria;p__Firmicutes;c__Bacilli;o__Erysipelotrichales;f__Erysipelatoclostridiaceae;__	0.000%	0.000%	0.000%	0.323%	0.000%	0.000%	0.128%	0.000%	0.134%
d__Bacteria;p__Firmicutes;c__Bacilli;o__Erysipelotrichales;f__Erysipelatoclostridiaceae;g__Erysipelatoclostridiaceae	0.000%	0.000%	0.000%	0.211%	0.112%	0.038%	0.121%	0.084%	0.000%
d__Bacteria;p__Firmicutes;c__Bacilli;o__Erysipelotrichales;f__Erysipelotrichaceae;__	0.408%	15.165%	7.778%	9.493%	7.814%	6.742%	20.598%	16.290%	8.414%
d__Bacteria;p__Firmicutes;c__Bacilli;o__Erysipelotrichales;f__Erysipelotrichaceae;g__Allobaculum	0.000%	0.389%	6.814%	1.381%	3.605%	2.161%	1.150%	6.081%	3.516%
d__Bacteria;p__Firmicutes;c__Bacilli;o__Erysipelotrichales;f__Erysipelotrichaceae;g__Dubosiella	0.000%	0.000%	0.000%	0.000%	0.000%	2.480%	0.000%	0.628%	2.514%
d__Bacteria;p__Firmicutes;c__Bacilli;o__Erysipelotrichales;f__Erysipelotrichaceae;g__Faecalibaculum	0.017%	1.226%	0.284%	0.080%	0.036%	0.000%	0.047%	0.124%	0.067%
d__Bacteria;p__Firmicutes;c__Bacilli;o__Erysipelotrichales;f__Erysipelotrichaceae;g__Ileibacterium	0.198%	1.644%	14.129%	4.227%	5.504%	6.799%	0.692%	4.081%	4.331%
d__Bacteria;p__Firmicutes;c__Bacilli;o__Erysipelotrichales;f__Erysipelotrichaceae;g__uncultured	0.013%	4.568%	10.987%	3.641%	4.090%	3.570%	1.892%	0.702%	3.542%
d__Bacteria;p__Firmicutes;c__Bacilli;o__Lactobacillales;f__Lactobacillaceae;g__Lactobacillus	0.038%	10.135%	0.578%	1.159%	0.000%	1.441%	0.049%	0.406%	0.780%
d__Bacteria;p__Firmicutes;c__Bacilli;o__Lactobacillales;f__Streptococcaceae;g__Streptococcus	0.043%	0.299%	0.432%	1.187%	0.199%	0.112%	1.229%	0.317%	1.339%
d__Bacteria;p__Firmicutes;c__Bacilli;o__RF39;f__RF39;g__RF39	0.000%	0.000%	0.212%	0.196%	0.342%	0.446%	0.017%	0.025%	0.044%

Supplementary File 5
Relative Abundance Data

2DPT Fecal Genus Level Taxon Relative Abundance Continued	AC-PO7d	AC-10mg/kg	AC-5mg/kg	anti-rsPilA	anti-tip-chime	Ofloxacin	TS-30mg/kg	TS-15mg/kg	Saline
d__Bacteria;p__Firmicutes;c__Clostridia;o__Christensenellales;f__Christensenellaceae;__	0.183%	1.226%	0.000%	0.000%	0.000%	0.000%	0.000%	0.121%	0.000%
d__Bacteria;p__Firmicutes;c__Clostridia;o__Christensenellales;f__Christensenellaceae;g__Christensenellaceae_R-7_group	0.000%	1.080%	1.352%	6.289%	4.031%	4.673%	3.185%	10.095%	5.673%
d__Bacteria;p__Firmicutes;c__Clostridia;o__Clostridia;f__Hungateiclostridiaceae;g__Ruminiclostridium	0.000%	0.000%	0.000%	0.060%	0.000%	0.000%	0.059%	0.042%	0.000%
d__Bacteria;p__Firmicutes;c__Clostridia;o__Clostridia_UCG-014;f__Clostridia_UCG-014;g__Clostridia_UCG-014	0.000%	0.043%	0.584%	0.825%	0.952%	0.615%	0.673%	1.640%	0.272%
d__Bacteria;p__Firmicutes;c__Clostridia;o__Eubacteriales;f__Eubacteriaceae;g__uncultured	0.021%	1.585%	4.051%	5.705%	6.658%	10.921%	4.025%	5.364%	13.316%
d__Bacteria;p__Firmicutes;c__Clostridia;o__Lachnospirales;f__Defluviitaleaceae;g__Defluviitaleaceae_UCG-011	0.000%	0.000%	0.138%	0.226%	0.028%	0.105%	0.549%	0.151%	0.108%
d__Bacteria;p__Firmicutes;c__Clostridia;o__Lachnospirales;f__Lachnospiraceae;__	0.022%	1.242%	3.801%	3.693%	10.135%	2.215%	3.496%	3.964%	3.817%
d__Bacteria;p__Firmicutes;c__Clostridia;o__Lachnospirales;f__Lachnospiraceae;g__[Eubacterium]_ruminantium_group	0.000%	1.747%	0.099%	0.366%	0.000%	0.583%	0.781%	0.618%	0.760%
d__Bacteria;p__Firmicutes;c__Clostridia;o__Lachnospirales;f__Lachnospiraceae;g__[Ruminococcus]_torques_group	0.000%	0.000%	0.066%	0.000%	0.130%	0.096%	0.111%	0.198%	0.000%
d__Bacteria;p__Firmicutes;c__Clostridia;o__Lachnospirales;f__Lachnospiraceae;g__Blautia	0.241%	0.229%	0.777%	0.907%	0.204%	0.398%	0.559%	0.967%	0.786%
d__Bacteria;p__Firmicutes;c__Clostridia;o__Lachnospirales;f__Lachnospiraceae;g__Coprococcus	0.000%	0.053%	0.887%	0.985%	0.064%	0.121%	0.224%	0.082%	0.023%
d__Bacteria;p__Firmicutes;c__Clostridia;o__Lachnospirales;f__Lachnospiraceae;g__Dorea	0.000%	0.000%	0.107%	0.078%	0.000%	0.000%	0.670%	0.143%	0.000%
d__Bacteria;p__Firmicutes;c__Clostridia;o__Lachnospirales;f__Lachnospiraceae;g__Frisingicoccus	0.000%	0.000%	0.000%	1.112%	0.000%	0.067%	0.081%	0.571%	0.061%
d__Bacteria;p__Firmicutes;c__Clostridia;o__Lachnospirales;f__Lachnospiraceae;g__Lachnoclostridium	0.216%	0.000%	1.834%	3.176%	0.082%	1.431%	1.838%	1.781%	1.061%
d__Bacteria;p__Firmicutes;c__Clostridia;o__Lachnospirales;f__Lachnospiraceae;g__Lachnospira	0.000%	0.000%	0.000%	0.131%	0.000%	0.054%	0.000%	0.022%	0.000%
d__Bacteria;p__Firmicutes;c__Clostridia;o__Lachnospirales;f__Lachnospiraceae;g__Lachnospiraceae_NK4A136_group	0.000%	0.156%	0.000%	0.289%	0.472%	0.000%	0.929%	1.321%	0.801%
d__Bacteria;p__Firmicutes;c__Clostridia;o__Lachnospirales;f__Lachnospiraceae;g__Lachnospiraceae_UCG-001	0.000%	0.000%	0.110%	0.000%	0.000%	0.000%	0.000%	0.000%	0.000%
d__Bacteria;p__Firmicutes;c__Clostridia;o__Lachnospirales;f__Lachnospiraceae;g__Lachnospiraceae_UCG-004	0.000%	0.000%	0.000%	0.024%	0.051%	0.089%	0.000%	0.069%	0.079%
d__Bacteria;p__Firmicutes;c__Clostridia;o__Lachnospirales;f__Lachnospiraceae;g__Lachnospiraceae_UCG-010	0.000%	0.000%	0.000%	0.392%	0.051%	0.096%	0.000%	0.324%	0.000%
d__Bacteria;p__Firmicutes;c__Clostridia;o__Lachnospirales;f__Lachnospiraceae;g__Marvinbryantia	0.008%	0.149%	0.650%	0.084%	0.064%	0.000%	0.251%	0.148%	0.000%
d__Bacteria;p__Firmicutes;c__Clostridia;o__Lachnospirales;f__Lachnospiraceae;g__uncultured	0.000%	0.000%	0.000%	0.000%	0.000%	0.000%	0.012%	0.322%	0.105%
d__Bacteria;p__Firmicutes;c__Clostridia;o__Monoglobales;f__Monoglobaceae;g__Monoglobus	0.027%	0.159%	0.435%	0.771%	0.449%	0.430%	0.983%	0.193%	0.471%
d__Bacteria;p__Firmicutes;c__Clostridia;o__Oscillospirales;__;__	0.000%	0.000%	0.000%	0.149%	0.263%	0.163%	0.187%	0.129%	0.243%
d__Bacteria;p__Firmicutes;c__Clostridia;o__Oscillospirales;f__[Eubacterium]_coprostanoligenes_group;g__[Eubacterium]_coprostanoligenes_group	0.000%	0.056%	0.240%	1.342%	0.431%	0.548%	0.554%	0.848%	0.058%
d__Bacteria;p__Firmicutes;c__Clostridia;o__Oscillospirales;f__Butyricicoccaceae;g__UCG-008	0.020%	0.804%	0.000%	0.386%	0.214%	0.249%	0.000%	0.000%	0.085%
d__Bacteria;p__Firmicutes;c__Clostridia;o__Oscillospirales;f__Oscillospiraceae;__	0.085%	0.060%	0.000%	1.482%	0.217%	0.000%	0.404%	0.047%	0.696%
d__Bacteria;p__Firmicutes;c__Clostridia;o__Oscillospirales;f__Oscillospiraceae;g__Colidextribacter	0.018%	0.000%	0.000%	0.213%	0.250%	0.131%	0.717%	0.279%	0.617%
d__Bacteria;p__Firmicutes;c__Clostridia;o__Oscillospirales;f__Oscillospiraceae;g__NK4A214_group	0.000%	0.000%	0.099%	0.452%	0.424%	0.609%	0.493%	0.228%	0.438%
d__Bacteria;p__Firmicutes;c__Clostridia;o__Oscillospirales;f__Oscillospiraceae;g__Oscillibacter	0.000%	0.000%	0.000%	0.082%	0.138%	0.000%	0.121%	0.020%	0.044%
d__Bacteria;p__Firmicutes;c__Clostridia;o__Oscillospirales;f__Oscillospiraceae;g__Papillibacter	0.000%	0.000%	0.000%	0.060%	0.278%	0.000%	0.000%	0.067%	0.108%
d__Bacteria;p__Firmicutes;c__Clostridia;o__Oscillospirales;f__Oscillospiraceae;g__UCG-005	0.310%	0.445%	0.600%	1.060%	2.111%	1.651%	1.784%	0.581%	2.303%
d__Bacteria;p__Firmicutes;c__Clostridia;o__Oscillospirales;f__Oscillospiraceae;g__uncultured	0.094%	0.090%	0.595%	2.103%	0.477%	0.963%	1.843%	3.522%	0.438%
d__Bacteria;p__Firmicutes;c__Clostridia;o__Oscillospirales;f__Ruminococcaceae;__	0.000%	0.000%	0.074%	0.043%	0.059%	0.077%	0.340%	0.069%	0.000%
d__Bacteria;p__Firmicutes;c__Clostridia;o__Oscillospirales;f__Ruminococcaceae;g__[Eubacterium]_siraeum_group	0.000%	0.000%	0.000%	0.000%	0.317%	0.000%	0.032%	0.000%	0.000%
d__Bacteria;p__Firmicutes;c__Clostridia;o__Oscillospirales;f__Ruminococcaceae;g__Incertae_Sedis	0.000%	0.718%	1.008%	1.213%	0.666%	0.386%	1.150%	2.411%	0.409%
d__Bacteria;p__Firmicutes;c__Clostridia;o__Oscillospirales;f__Ruminococcaceae;g__Ruminococcaceae	0.000%	0.000%	0.000%	0.000%	0.000%	0.000%	0.175%	0.000%	0.000%
d__Bacteria;p__Firmicutes;c__Clostridia;o__Oscillospirales;f__Ruminococcaceae;g__Ruminococcus	0.000%	0.967%	1.085%	1.493%	3.204%	1.600%	3.543%	1.847%	2.429%
d__Bacteria;p__Firmicutes;c__Clostridia;o__Oscillospirales;f__Ruminococcaceae;g__UBA1819	0.117%	0.080%	0.019%	0.500%	0.000%	0.000%	0.000%	0.124%	0.047%
d__Bacteria;p__Firmicutes;c__Clostridia;o__Peptostreptococcales-Tissierellales;f__Anaerovoracaceae;g__[Eubacterium]_brachy_group	0.011%	0.063%	0.055%	0.140%	0.000%	0.051%	0.059%	0.074%	0.050%
d__Bacteria;p__Firmicutes;c__Clostridia;o__Peptostreptococcales-Tissierellales;f__Anaerovoracaceae;g__[Eubacterium]_nodatum_group	0.000%	0.000%	0.300%	0.211%	0.066%	0.096%	0.000%	0.047%	0.000%
d__Bacteria;p__Firmicutes;c__Clostridia;o__Peptostreptococcales-Tissierellales;f__Anaerovoracaceae;g__Family_XIII_AD3011_group	0.061%	0.345%	0.865%	0.987%	0.462%	0.599%	0.099%	0.067%	0.567%
d__Bacteria;p__Firmicutes;c__Clostridia;o__Peptostreptococcales-Tissierellales;f__Anaerovoracaceae;g__Mogibacterium	0.000%	0.797%	0.647%	0.396%	0.281%	0.354%	0.288%	0.660%	0.099%
d__Bacteria;p__Firmicutes;c__Negativicutes;o__Acidaminococcales;f__Acidaminococcaceae;g__Phascolarctobacterium	0.000%	0.349%	0.262%	0.347%	0.855%	0.316%	0.416%	0.235%	0.570%
d__Bacteria;p__Fusobacteriota;c__Fusobacteriia;o__Fusobacteriales;f__Fusobacteriaceae;g__Fusobacterium	34.984%	3.329%	0.000%	0.000%	0.000%	0.029%	0.000%	0.000%	0.029%
d__Bacteria;p__Patescibacteria;c__Saccharimonadia;o__Saccharimonadales;f__Saccharimonadaceae;g__Candidatus_Saccharimonas	0.136%	1.372%	1.969%	1.879%	3.474%	2.531%	4.072%	0.433%	1.444%
d__Bacteria;p__Planctomycetota;c__Planctomycetes;o__Pirellulales;f__Pirellulaceae;g__p-1088-a5_gut_group	0.012%	0.442%	0.102%	0.069%	0.054%	0.252%	0.680%	0.025%	0.035%
d__Bacteria;p__Proteobacteria;c__Gammaproteobacteria;o__Burkholderiales;f__Sutterellaceae;g__Parasutterella	0.093%	0.644%	0.242%	0.595%	0.207%	0.281%	0.111%	0.225%	0.412%
d__Bacteria;p__Proteobacteria;c__Gammaproteobacteria;o__Enterobacterales;f__Enterobacteriaceae;g__Escherichia-Shigella	8.750%	2.136%	0.017%	0.000%	0.176%	0.673%	0.000%	0.000%	0.079%
d__Bacteria;p__Proteobacteria;c__Gammaproteobacteria;o__Pasteurellales;f__Pasteurellaceae;g__Haemophilus	0.669%	1.086%	3.740%	0.058%	0.551%	0.000%	0.000%	0.000%	0.000%
d__Bacteria;p__Spirochaetota;c__Spirochaetia;o__Spirochaetales;f__Spirochaetaceae;g__Treponema	0.000%	0.040%	0.094%	0.086%	0.544%	1.868%	0.052%	1.036%	0.222%
d__Bacteria;p__Verrucomicrobiota;c__Verrucomicrobiae;o__Verrucomicrobiales;f__Akkermansiaceae;g__Akkermansia	0.004%	0.000%	1.317%	0.000%	0.978%	0.045%	3.217%	0.000%	0.275%

Supplementary File 5
Relative Abundance Data

5DPT Fecal Genus Level Taxon Relative Abundance	AC-PO7d	AC-10mg/kg	AC-5mg/kg	anti-rsPilA	anti-tip-chime	Ofloxacin	TS-30mg/kg	TS-15mg/kg	Saline
d__Archaea;p__Euryarchaeota;c__Methanobacteria;o__Methanobacteriales;f__Methanobacteriaceae;g__Methanobrevibacter	2.426%	0.095%	10.674%	12.504%	4.728%	12.463%	4.539%	9.106%	0.242%
d__Archaea;p__Euryarchaeota;c__Methanobacteria;o__Methanobacteriales;f__Methanobacteriaceae;g__Methanosphaera	0.078%	0.022%	0.314%	0.000%	0.000%	0.025%	0.306%	0.287%	0.163%
d__Bacteria;p__Actinobacteriota;c__Actinobacteria;o__Actinomycetales;f__Actinomycetaceae;g__Actinomyces	0.000%	0.000%	0.000%	0.116%	0.000%	0.000%	0.043%	0.056%	0.163%
d__Bacteria;p__Actinobacteriota;c__Actinobacteria;o__Actinomycetales;f__Actinomycetaceae;g__Actinomycetaceae	0.000%	4.470%	0.618%	0.000%	0.000%	0.000%	0.000%	0.000%	0.023%
d__Bacteria;p__Actinobacteriota;c__Actinobacteria;o__Bifidobacteriales;f__Bifidobacteriaceae;g__Bifidobacterium	1.054%	0.000%	0.066%	0.855%	0.326%	0.014%	1.568%	2.521%	3.305%
d__Bacteria;p__Actinobacteriota;c__Coriobacteriia;o__Coriobacteriales;__;__	0.049%	0.000%	0.041%	0.451%	0.075%	0.152%	0.066%	0.063%	0.048%
d__Bacteria;p__Actinobacteriota;c__Coriobacteriia;o__Coriobacteriales;f__Atopobiaceae;__	0.175%	0.000%	0.098%	2.075%	0.888%	1.405%	1.845%	0.497%	1.677%
d__Bacteria;p__Actinobacteriota;c__Coriobacteriia;o__Coriobacteriales;f__Atopobiaceae;g__uncultured	0.057%	0.045%	0.027%	0.069%	0.000%	0.045%	0.082%	0.109%	0.235%
d__Bacteria;p__Actinobacteriota;c__Coriobacteriia;o__Coriobacteriales;f__Coriobacteriaceae;g__Collinsella	0.486%	2.460%	0.238%	0.000%	0.000%	0.000%	0.000%	0.000%	0.072%
d__Bacteria;p__Actinobacteriota;c__Coriobacteriia;o__Coriobacteriales;f__Coriobacteriales_Incertae_Sedis;g__uncultured	0.144%	0.000%	0.044%	0.129%	0.083%	0.079%	0.118%	0.099%	0.149%
d__Bacteria;p__Actinobacteriota;c__Coriobacteriia;o__Coriobacteriales;f__Eggerthellaceae;__	0.000%	0.000%	0.090%	0.309%	0.255%	0.268%	0.647%	0.874%	0.266%
d__Bacteria;p__Actinobacteriota;c__Coriobacteriia;o__Coriobacteriales;f__Eggerthellaceae;g__Adlercreutzia	0.553%	0.000%	0.068%	0.000%	0.000%	0.152%	0.000%	0.000%	0.048%
d__Bacteria;p__Actinobacteriota;c__Coriobacteriia;o__Coriobacteriales;f__Eggerthellaceae;g__Enterorhabdus	0.255%	0.000%	0.033%	0.718%	0.490%	0.150%	0.940%	0.608%	1.134%
d__Bacteria;p__Actinobacteriota;c__Coriobacteriia;o__Coriobacteriales;f__Eggerthellaceae;g__Paraeggerthella	0.000%	3.719%	5.441%	0.000%	0.000%	0.000%	0.000%	0.241%	0.000%
d__Bacteria;p__Actinobacteriota;c__Coriobacteriia;o__Coriobacteriales;f__Eggerthellaceae;g__uncultured	0.000%	0.000%	0.421%	0.361%	0.360%	2.043%	0.844%	1.746%	1.462%
d__Bacteria;p__Actinobacteriota;c__Coriobacteriia;o__Coriobacteriales;f__uncultured;g__uncultured	0.047%	0.056%	0.052%	0.000%	0.033%	0.023%	0.306%	0.360%	0.045%
d__Bacteria;p__Bacteroidota;c__Bacteroidia;o__Bacteroidales;__;__	0.160%	0.000%	0.019%	0.421%	0.127%	0.206%	0.125%	0.384%	0.428%
d__Bacteria;p__Bacteroidota;c__Bacteroidia;o__Bacteroidales;f__Bacteroidaceae;g__Bacteroides	12.593%	17.546%	14.596%	0.640%	1.170%	1.891%	0.958%	1.301%	0.932%
d__Bacteria;p__Bacteroidota;c__Bacteroidia;o__Bacteroidales;f__Marinifilaceae;g__Butyricimonas	0.121%	0.724%	0.618%	0.026%	0.047%	0.279%	0.000%	0.046%	0.116%
d__Bacteria;p__Bacteroidota;c__Bacteroidia;o__Bacteroidales;f__Marinifilaceae;g__Odoribacter	0.355%	0.401%	1.558%	0.000%	0.061%	0.011%	0.000%	0.041%	0.000%
d__Bacteria;p__Bacteroidota;c__Bacteroidia;o__Bacteroidales;f__Muribaculaceae;__	0.000%	0.000%	0.000%	0.125%	0.000%	0.000%	0.150%	0.000%	0.000%
d__Bacteria;p__Bacteroidota;c__Bacteroidia;o__Bacteroidales;f__Muribaculaceae;g__Muribaculaceae	17.002%	2.444%	6.015%	20.557%	18.058%	15.496%	10.308%	14.324%	######
d__Bacteria;p__Bacteroidota;c__Bacteroidia;o__Bacteroidales;f__Muribaculaceae;g__Muribaculum	0.015%	0.367%	0.000%	0.082%	0.000%	0.102%	0.000%	0.106%	0.211%
d__Bacteria;p__Bacteroidota;c__Bacteroidia;o__Bacteroidales;f__Prevotellaceae;g__Prevotella	2.021%	0.000%	0.000%	1.096%	3.193%	2.551%	5.267%	0.275%	0.725%
d__Bacteria;p__Bacteroidota;c__Bacteroidia;o__Bacteroidales;f__Prevotellaceae;g__Prevotellaceae_Ga6A1_group	0.142%	0.000%	0.000%	0.021%	0.160%	0.209%	0.159%	0.080%	0.419%
d__Bacteria;p__Bacteroidota;c__Bacteroidia;o__Bacteroidales;f__Prevotellaceae;g__Prevotellaceae_NK3B31_group	0.038%	0.000%	0.891%	0.950%	0.401%	0.373%	0.111%	0.094%	0.219%
d__Bacteria;p__Bacteroidota;c__Bacteroidia;o__Bacteroidales;f__Prevotellaceae;g__Prevotellaceae_UCG-001	0.338%	0.000%	0.000%	0.477%	3.339%	3.034%	4.378%	0.357%	3.166%
d__Bacteria;p__Bacteroidota;c__Bacteroidia;o__Bacteroidales;f__Prevotellaceae;g__Prevotellaceae_UCG-003	0.000%	0.000%	0.000%	0.649%	0.036%	0.144%	0.000%	0.000%	0.000%
d__Bacteria;p__Bacteroidota;c__Bacteroidia;o__Bacteroidales;f__Rikenellaceae;g__Alistipes	1.025%	0.629%	0.159%	0.146%	0.235%	0.000%	0.213%	0.558%	0.279%
d__Bacteria;p__Bacteroidota;c__Bacteroidia;o__Bacteroidales;f__Rikenellaceae;g__dgA-11_gut_group	0.101%	0.000%	0.000%	0.202%	0.069%	0.085%	0.107%	0.065%	0.085%
d__Bacteria;p__Bacteroidota;c__Bacteroidia;o__Bacteroidales;f__Rikenellaceae;g__Rikenellaceae_RC9_gut_group	0.183%	0.000%	0.000%	0.000%	0.097%	0.000%	0.182%	0.000%	0.539%
d__Bacteria;p__Bacteroidota;c__Bacteroidia;o__Bacteroidales;f__Tannerellaceae;g__Parabacteroides	1.489%	1.687%	2.815%	0.352%	0.116%	0.223%	0.413%	0.164%	0.364%
d__Bacteria;p__Desulfobacterota;c__Desulfovibrionia;o__Desulfovibrionales;f__Desulfovibrionaceae;g__Bilophila	0.623%	0.000%	3.801%	0.000%	0.000%	0.000%	0.036%	0.019%	0.227%
d__Bacteria;p__Desulfobacterota;c__Desulfovibrionia;o__Desulfovibrionales;f__Desulfovibrionaceae;g__Desulfovibrio	0.000%	0.000%	0.000%	0.052%	0.111%	0.000%	0.000%	0.000%	0.293%
d__Bacteria;p__Elusimicrobiota;c__Elusimicrobia;o__Elusimicrobiales;f__Elusimicrobiaceae;g__Elusimicrobium	0.049%	0.000%	0.186%	0.950%	0.053%	0.000%	0.048%	0.080%	0.238%
d__Bacteria;p__Firmicutes;c__Bacilli;o__Erysipelotrichales;f__Erysipelatoclostridiaceae;__	0.098%	0.000%	0.424%	0.125%	0.000%	0.000%	0.129%	0.000%	0.091%
d__Bacteria;p__Firmicutes;c__Bacilli;o__Erysipelotrichales;f__Erysipelatoclostridiaceae;g__Erysipelatoclostridiaceae	0.069%	2.349%	1.224%	0.000%	0.047%	0.209%	0.014%	0.092%	0.019%
d__Bacteria;p__Firmicutes;c__Bacilli;o__Erysipelotrichales;f__Erysipelotrichaceae;__	2.800%	0.000%	0.175%	9.346%	6.352%	10.484%	19.814%	17.151%	######
d__Bacteria;p__Firmicutes;c__Bacilli;o__Erysipelotrichales;f__Erysipelotrichaceae;g__Allobaculum	0.118%	0.000%	0.372%	0.834%	4.892%	1.166%	0.846%	5.100%	2.351%
d__Bacteria;p__Firmicutes;c__Bacilli;o__Erysipelotrichales;f__Erysipelotrichaceae;g__Dubosiella	0.201%	0.000%	0.000%	0.000%	0.000%	0.468%	0.000%	0.372%	1.471%
d__Bacteria;p__Firmicutes;c__Bacilli;o__Erysipelotrichales;f__Erysipelotrichaceae;g__Faecalibaculum	0.000%	0.184%	0.834%	0.000%	0.000%	0.000%	0.093%	0.068%	0.041%
d__Bacteria;p__Firmicutes;c__Bacilli;o__Erysipelotrichales;f__Erysipelotrichaceae;g__Ileibacterium	0.258%	0.022%	1.153%	3.004%	6.527%	5.074%	0.806%	2.436%	2.931%
d__Bacteria;p__Firmicutes;c__Bacilli;o__Erysipelotrichales;f__Erysipelotrichaceae;g__uncultured	0.602%	0.000%	0.036%	0.958%	4.302%	1.027%	1.797%	0.676%	2.844%
d__Bacteria;p__Firmicutes;c__Bacilli;o__Lactobacillales;f__Lactobacillaceae;g__Lactobacillus	0.896%	0.456%	0.249%	2.028%	0.086%	0.000%	0.054%	0.292%	1.419%
d__Bacteria;p__Firmicutes;c__Bacilli;o__Lactobacillales;f__Streptococcaceae;g__Streptococcus	0.095%	0.256%	0.038%	3.867%	0.241%	0.040%	1.033%	0.307%	0.742%
d__Bacteria;p__Firmicutes;c__Bacilli;o__RF39;f__RF39;g__RF39	0.116%	0.000%	0.000%	1.345%	0.307%	0.542%	0.075%	0.270%	0.306%

Supplementary File 5
Relative Abundance Data

5DPT Fecal Genus Level Taxon Relative Abundance Continued	AC-PO7d	AC-10mg/kg	AC-5mg/kg	anti-rsPilA	anti-tip-chime	Ofloxacin	TS-30mg/kg	TS-15mg/kg	Saline
d__Bacteria;p__Firmicutes;c__Clostridia;o__Christensenellales;f__Christensenellaceae;__	0.052%	0.150%	0.063%	0.000%	0.000%	0.000%	0.000%	0.212%	0.000%
d__Bacteria;p__Firmicutes;c__Clostridia;o__Christensenellales;f__Christensenellaceae;g__Christensenellaceae_R-7_group	1.146%	0.084%	0.380%	4.254%	2.728%	5.404%	2.392%	6.107%	3.813%
d__Bacteria;p__Firmicutes;c__Clostridia;o__Clostridia;f__Hungateiclostridiaceae;g__Ruminiclostridium	0.118%	0.100%	0.044%	0.219%	0.075%	0.071%	0.000%	0.039%	0.000%
d__Bacteria;p__Firmicutes;c__Clostridia;o__Clostridia_UCG-014;f__Clostridia_UCG-014;g__Clostridia_UCG-014	0.319%	0.000%	0.197%	0.477%	0.955%	1.642%	0.284%	0.517%	1.254%
d__Bacteria;p__Firmicutes;c__Clostridia;o__Eubacteriales;f__Eubacteriaceae;g__uncultured	0.713%	0.000%	0.011%	2.849%	10.126%	4.936%	3.804%	5.411%	9.790%
d__Bacteria;p__Firmicutes;c__Clostridia;o__Lachnospirales;f__Defluviitaleaceae;g__Defluviitaleaceae_UCG-011	0.139%	0.000%	0.044%	0.000%	0.083%	0.000%	0.424%	0.111%	0.109%
d__Bacteria;p__Firmicutes;c__Clostridia;o__Lachnospirales;f__Lachnospiraceae;__	11.187%	6.057%	5.160%	2.093%	9.454%	1.885%	2.521%	4.477%	6.004%
d__Bacteria;p__Firmicutes;c__Clostridia;o__Lachnospirales;f__Lachnospiraceae;g__[Eubacterium]_ruminantium_group	0.000%	0.000%	0.000%	0.000%	0.000%	0.875%	0.735%	0.896%	0.231%
d__Bacteria;p__Firmicutes;c__Clostridia;o__Lachnospirales;f__Lachnospiraceae;g__[Ruminococcus]_torques_group	0.000%	0.000%	0.000%	0.086%	0.000%	0.000%	0.100%	0.109%	0.000%
d__Bacteria;p__Firmicutes;c__Clostridia;o__Lachnospirales;f__Lachnospiraceae;g__Blautia	0.190%	0.501%	1.547%	0.503%	0.415%	1.058%	0.343%	1.338%	0.855%
d__Bacteria;p__Firmicutes;c__Clostridia;o__Lachnospirales;f__Lachnospiraceae;g__Coprococcus	0.302%	0.061%	0.096%	0.662%	0.000%	0.085%	0.104%	0.048%	0.050%
d__Bacteria;p__Firmicutes;c__Clostridia;o__Lachnospirales;f__Lachnospiraceae;g__Dorea	0.000%	0.228%	0.000%	0.000%	0.000%	0.102%	0.424%	0.140%	0.099%
d__Bacteria;p__Firmicutes;c__Clostridia;o__Lachnospirales;f__Lachnospiraceae;g__Frisingicoccus	0.000%	0.128%	0.000%	0.150%	0.000%	1.411%	0.000%	1.157%	0.068%
d__Bacteria;p__Firmicutes;c__Clostridia;o__Lachnospirales;f__Lachnospiraceae;g__Lachnoclostridium	0.636%	1.915%	2.932%	1.263%	0.528%	1.956%	0.940%	1.574%	1.830%
d__Bacteria;p__Firmicutes;c__Clostridia;o__Lachnospirales;f__Lachnospiraceae;g__Lachnospiraceae_NK4A136_group	1.891%	0.000%	0.847%	0.546%	0.971%	0.240%	0.994%	0.657%	0.514%
d__Bacteria;p__Firmicutes;c__Clostridia;o__Lachnospirales;f__Lachnospiraceae;g__Lachnospiraceae_UCG-001	0.000%	0.000%	0.000%	0.000%	0.138%	0.068%	0.050%	0.000%	0.000%
d__Bacteria;p__Firmicutes;c__Clostridia;o__Lachnospirales;f__Lachnospiraceae;g__Lachnospiraceae_UCG-004	0.046%	0.122%	0.273%	0.112%	0.000%	0.000%	0.039%	0.000%	0.029%
d__Bacteria;p__Firmicutes;c__Clostridia;o__Lachnospirales;f__Lachnospiraceae;g__Lachnospiraceae_UCG-010	0.054%	0.156%	0.000%	0.000%	0.000%	0.048%	0.000%	0.147%	0.000%
d__Bacteria;p__Firmicutes;c__Clostridia;o__Lachnospirales;f__Lachnospiraceae;g__Marvinbryantia	0.469%	0.000%	0.085%	0.000%	0.000%	0.000%	0.059%	0.000%	0.114%
d__Bacteria;p__Firmicutes;c__Clostridia;o__Lachnospirales;f__Lachnospiraceae;g__uncultured	0.041%	0.000%	0.000%	0.060%	0.266%	0.107%	0.100%	0.126%	0.068%
d__Bacteria;p__Firmicutes;c__Clostridia;o__Monoglobales;f__Monoglobaceae;g__Monoglobus	0.351%	0.779%	0.000%	1.006%	0.454%	1.445%	1.486%	0.336%	0.279%
d__Bacteria;p__Firmicutes;c__Clostridia;o__Oscillospirales;__;__	0.000%	0.000%	0.000%	0.146%	0.138%	0.000%	0.066%	0.070%	0.157%
d__Bacteria;p__Firmicutes;c__Clostridia;o__Oscillospirales;f__[Eubacterium]_coprostanoligenes_group;g__[Eubacterium]_coprostanoligenes_group	0.067%	0.072%	0.033%	0.653%	0.116%	0.960%	0.477%	0.430%	0.314%
d__Bacteria;p__Firmicutes;c__Clostridia;o__Oscillospirales;f__Butyricicoccaceae;g__UCG-008	0.288%	0.000%	0.016%	0.318%	0.108%	0.144%	0.045%	0.000%	0.192%
d__Bacteria;p__Firmicutes;c__Clostridia;o__Oscillospirales;f__Oscillospiraceae;__	0.226%	0.000%	0.219%	0.438%	0.789%	0.121%	0.034%	0.220%	0.595%
d__Bacteria;p__Firmicutes;c__Clostridia;o__Oscillospirales;f__Oscillospiraceae;g__Colidextribacter	0.392%	0.000%	0.388%	0.000%	0.462%	0.339%	0.529%	0.157%	0.254%
d__Bacteria;p__Firmicutes;c__Clostridia;o__Oscillospirales;f__Oscillospiraceae;g__NK4A214_group	0.033%	0.000%	0.000%	0.413%	0.523%	0.124%	0.161%	0.171%	0.461%
d__Bacteria;p__Firmicutes;c__Clostridia;o__Oscillospirales;f__Oscillospiraceae;g__Oscillibacter	0.057%	0.000%	0.000%	0.000%	0.028%	0.000%	0.191%	0.000%	0.000%
d__Bacteria;p__Firmicutes;c__Clostridia;o__Oscillospirales;f__Oscillospiraceae;g__Papillibacter	0.129%	0.000%	0.000%	0.000%	0.000%	0.135%	0.000%	0.000%	0.074%
d__Bacteria;p__Firmicutes;c__Clostridia;o__Oscillospirales;f__Oscillospiraceae;g__UCG-005	2.460%	0.857%	0.741%	1.641%	1.433%	1.383%	1.602%	0.681%	2.369%
d__Bacteria;p__Firmicutes;c__Clostridia;o__Oscillospirales;f__Oscillospiraceae;g__uncultured	3.314%	0.145%	0.273%	1.358%	1.314%	0.923%	1.289%	1.309%	0.549%
d__Bacteria;p__Firmicutes;c__Clostridia;o__Oscillospirales;f__Ruminococcaceae;__	0.731%	0.000%	0.000%	0.133%	0.042%	0.056%	0.334%	0.082%	0.000%
d__Bacteria;p__Firmicutes;c__Clostridia;o__Oscillospirales;f__Ruminococcaceae;g__[Eubacterium]_siraeum_group	0.131%	0.000%	0.000%	0.000%	0.246%	0.000%	0.000%	0.000%	0.000%
d__Bacteria;p__Firmicutes;c__Clostridia;o__Oscillospirales;f__Ruminococcaceae;g__Incertae_Sedis	0.566%	0.000%	0.000%	2.630%	1.613%	0.508%	1.480%	2.893%	0.390%
d__Bacteria;p__Firmicutes;c__Clostridia;o__Oscillospirales;f__Ruminococcaceae;g__Ruminococcaceae	0.029%	0.000%	0.000%	0.000%	0.160%	0.093%	0.152%	0.000%	0.000%
d__Bacteria;p__Firmicutes;c__Clostridia;o__Oscillospirales;f__Ruminococcaceae;g__Ruminococcus	2.604%	1.742%	1.757%	1.663%	3.184%	5.255%	2.671%	0.671%	1.758%
d__Bacteria;p__Firmicutes;c__Clostridia;o__Oscillospirales;f__Ruminococcaceae;g__UBA1819	1.774%	5.177%	0.834%	0.000%	0.000%	0.000%	0.000%	0.220%	0.029%
d__Bacteria;p__Firmicutes;c__Clostridia;o__Peptostreptococcales-Tissierellales;f__Anaerovoracaceae;g__[Eubacterium]_brachy_group	0.144%	0.000%	0.000%	0.138%	0.000%	0.000%	0.066%	0.116%	0.031%
d__Bacteria;p__Firmicutes;c__Clostridia;o__Peptostreptococcales-Tissierellales;f__Anaerovoracaceae;g__[Eubacterium]_nodatum_group	0.113%	0.000%	0.232%	0.142%	0.183%	0.257%	0.000%	0.099%	0.091%
d__Bacteria;p__Firmicutes;c__Clostridia;o__Peptostreptococcales-Tissierellales;f__Anaerovoracaceae;g__Family_XIII_AD3011_group	0.139%	0.039%	0.484%	0.593%	0.282%	0.344%	0.084%	0.051%	0.419%
d__Bacteria;p__Firmicutes;c__Clostridia;o__Peptostreptococcales-Tissierellales;f__Anaerovoracaceae;g__Mogibacterium	0.000%	0.000%	0.148%	0.516%	0.213%	0.031%	0.243%	0.531%	0.240%
d__Bacteria;p__Firmicutes;c__Negativicutes;o__Acidaminococcales;f__Acidaminococcaceae;g__Phascolarctobacterium	0.131%	0.000%	0.071%	0.984%	0.833%	0.779%	0.433%	0.698%	0.483%
d__Bacteria;p__Fusobacteriota;c__Fusobacteriia;o__Fusobacteriales;f__Fusobacteriaceae;g__Fusobacterium	16.129%	10.532%	20.775%	0.000%	0.000%	0.000%	0.000%	0.094%	0.014%
d__Bacteria;p__Patescibacteria;c__Saccharimonadia;o__Saccharimonadales;f__Saccharimonadaceae;g__Candidatus_Saccharimonas	0.817%	0.000%	0.199%	7.232%	3.135%	4.857%	8.079%	2.687%	2.361%
d__Bacteria;p__Planctomycetota;c__Planctomycetes;o__Pirellulales;f__Pirellulaceae;g__p-1088-a5_gut_group	0.105%	0.050%	0.134%	0.030%	0.089%	0.099%	0.202%	0.104%	0.035%
d__Bacteria;p__Proteobacteria;c__Gammaproteobacteria;o__Burkholderiales;f__Sutterellaceae;g__Parasutterella	0.211%	0.056%	0.538%	0.133%	0.208%	0.158%	0.141%	0.220%	0.446%
d__Bacteria;p__Proteobacteria;c__Gammaproteobacteria;o__Enterobacterales;f__Enterobacteriaceae;g__Escherichia-Shigella	3.842%	0.579%	4.785%	0.000%	0.562%	0.000%	0.023%	0.444%	0.101%
d__Bacteria;p__Proteobacteria;c__Gammaproteobacteria;o__Pasteurellales;f__Pasteurellaceae;g__Haemophilus	0.000%	5.700%	3.309%	0.017%	0.158%	0.000%	0.000%	0.000%	0.000%
d__Bacteria;p__Spirochaetota;c__Spirochaetia;o__Spirochaetales;f__Spirochaetaceae;g__Treponema	1.084%	0.000%	0.000%	0.705%	0.102%	1.668%	1.344%	3.426%	0.014%
d__Bacteria;p__Verrucomicrobiota;c__Verrucomicrobiae;o__Verrucomicrobiales;f__Akkermansiaceae;g__Akkermansia	0.000%	26.837%	0.000%	0.000%	0.623%	0.000%	7.137%	0.000%	1.677%

Supplementary File 5
Relative Abundance Data

7DPT Fecal Genus Level Taxon Relative Abundance	AC-PO7d	AC-10mg/kg	AC-5mg/kg	anti-rsPilA	anti-tip-chime	Ofloxacin	TS-30mg/kg	TS-15mg/kg	Saline
d__Archaea;p__Euryarchaeota;c__Methanobacteria;o__Methanobacteriales;f__Methanobacteriaceae;g__Methanobrevibacter	10.502%	0.109%	9.813%	6.720%	5.206%	15.404%	7.359%	9.526%	0.155%
d__Archaea;p__Euryarchaeota;c__Methanobacteria;o__Methanobacteriales;f__Methanobacteriaceae;g__Methanosphaera	0.019%	0.023%	0.181%	0.000%	0.000%	0.000%	0.046%	0.141%	0.015%
d__Bacteria;p__Actinobacteriota;c__Actinobacteria;o__Actinomycetales;f__Actinomycetaceae;g__Actinomyces	0.000%	1.493%	0.000%	0.147%	0.086%	0.000%	0.078%	0.000%	0.044%
d__Bacteria;p__Actinobacteriota;c__Actinobacteria;o__Actinomycetales;f__Actinomycetaceae;g__Actinomycetaceae	0.594%	3.049%	0.152%	0.000%	0.000%	0.000%	0.000%	0.000%	0.000%
d__Bacteria;p__Actinobacteriota;c__Actinobacteria;o__Bifidobacteriales;f__Bifidobacteriaceae;g__Bifidobacterium	0.146%	0.000%	0.000%	1.644%	0.408%	0.000%	1.325%	3.701%	3.729%
d__Bacteria;p__Actinobacteriota;c__Coriobacteriia;o__Coriobacteriales;__;__	0.000%	0.000%	0.000%	0.806%	0.278%	0.076%	0.086%	0.027%	0.000%
d__Bacteria;p__Actinobacteriota;c__Coriobacteriia;o__Coriobacteriales;f__Atopobiaceae;__	0.000%	0.000%	0.000%	0.935%	0.603%	1.579%	0.602%	0.759%	1.840%
d__Bacteria;p__Actinobacteriota;c__Coriobacteriia;o__Coriobacteriales;f__Atopobiaceae;g__uncultured	0.177%	1.532%	0.579%	0.086%	0.000%	0.025%	0.042%	0.146%	0.102%
d__Bacteria;p__Actinobacteriota;c__Coriobacteriia;o__Coriobacteriales;f__Coriobacteriaceae;g__Collinsella	5.228%	0.571%	0.238%	0.000%	0.000%	0.000%	0.000%	0.000%	0.000%
d__Bacteria;p__Actinobacteriota;c__Coriobacteriia;o__Coriobacteriales;f__Coriobacteriales_Incertae_Sedis;g__uncultured	0.019%	0.750%	0.363%	0.000%	0.086%	0.025%	0.067%	0.016%	0.029%
d__Bacteria;p__Actinobacteriota;c__Coriobacteriia;o__Coriobacteriales;f__Eggerthellaceae;__	0.000%	0.086%	0.152%	0.435%	0.562%	0.121%	0.984%	0.546%	0.252%
d__Bacteria;p__Actinobacteriota;c__Coriobacteriia;o__Coriobacteriales;f__Eggerthellaceae;g__Adlercreutzia	0.666%	0.000%	0.379%	0.140%	0.000%	0.205%	0.000%	0.061%	0.000%
d__Bacteria;p__Actinobacteriota;c__Coriobacteriia;o__Coriobacteriales;f__Eggerthellaceae;g__Enterorhabdus	0.028%	0.242%	0.607%	1.101%	0.970%	0.157%	1.323%	0.250%	0.804%
d__Bacteria;p__Actinobacteriota;c__Coriobacteriia;o__Coriobacteriales;f__Eggerthellaceae;g__Paraeggerthella	0.000%	3.643%	4.904%	0.000%	0.000%	0.000%	0.000%	0.051%	0.000%
d__Bacteria;p__Actinobacteriota;c__Coriobacteriia;o__Coriobacteriales;f__Eggerthellaceae;g__uncultured	0.080%	0.407%	1.904%	0.759%	0.393%	1.408%	0.474%	1.683%	1.429%
d__Bacteria;p__Actinobacteriota;c__Coriobacteriia;o__Coriobacteriales;f__uncultured;g__uncultured	0.076%	1.775%	0.501%	0.000%	0.148%	0.028%	0.317%	0.248%	0.334%
d__Bacteria;p__Bacteroidota;c__Bacteroidia;o__Bacteroidales;__;__	0.073%	0.000%	0.024%	0.263%	0.145%	0.107%	0.197%	0.335%	1.782%
d__Bacteria;p__Bacteroidota;c__Bacteroidia;o__Bacteroidales;f__Bacteroidaceae;g__Bacteroides	12.116%	5.073%	12.309%	0.637%	0.449%	3.704%	0.883%	2.492%	2.746%
d__Bacteria;p__Bacteroidota;c__Bacteroidia;o__Bacteroidales;f__Marinifilaceae;g__Butyricimonas	0.000%	0.000%	0.208%	0.000%	0.033%	0.684%	0.000%	0.101%	0.320%
d__Bacteria;p__Bacteroidota;c__Bacteroidia;o__Bacteroidales;f__Marinifilaceae;g__Odoribacter	0.052%	0.000%	0.068%	0.000%	0.000%	0.000%	0.008%	0.037%	0.107%
d__Bacteria;p__Bacteroidota;c__Bacteroidia;o__Bacteroidales;f__Muribaculaceae;__	0.000%	0.000%	0.000%	0.504%	0.000%	0.000%	0.136%	0.000%	0.000%
d__Bacteria;p__Bacteroidota;c__Bacteroidia;o__Bacteroidales;f__Muribaculaceae;g__Muribaculaceae	11.962%	5.566%	13.666%	20.984%	21.521%	17.311%	19.630%	18.177%	20.255%
d__Bacteria;p__Bacteroidota;c__Bacteroidia;o__Bacteroidales;f__Muribaculaceae;g__Muribaculum	0.000%	0.383%	0.000%	0.291%	0.000%	0.000%	0.086%	0.389%	0.446%
d__Bacteria;p__Bacteroidota;c__Bacteroidia;o__Bacteroidales;f__Prevotellaceae;g__Prevotella	0.495%	0.000%	0.815%	1.025%	1.647%	1.752%	1.529%	0.170%	0.644%
d__Bacteria;p__Bacteroidota;c__Bacteroidia;o__Bacteroidales;f__Prevotellaceae;g__Prevotellaceae_Ga6A1_group	0.000%	0.000%	0.000%	0.115%	0.228%	0.286%	0.271%	0.000%	0.232%
d__Bacteria;p__Bacteroidota;c__Bacteroidia;o__Bacteroidales;f__Prevotellaceae;g__Prevotellaceae_NK3B31_group	0.000%	0.000%	0.693%	0.072%	0.922%	0.488%	0.707%	0.109%	0.000%
d__Bacteria;p__Bacteroidota;c__Bacteroidia;o__Bacteroidales;f__Prevotellaceae;g__Prevotellaceae_UCG-001	0.092%	0.000%	0.000%	0.468%	0.822%	2.983%	1.992%	1.869%	0.620%
d__Bacteria;p__Bacteroidota;c__Bacteroidia;o__Bacteroidales;f__Prevotellaceae;g__Prevotellaceae_UCG-003	0.000%	0.000%	0.000%	0.032%	0.098%	0.202%	0.000%	0.000%	0.000%
d__Bacteria;p__Bacteroidota;c__Bacteroidia;o__Bacteroidales;f__Rikenellaceae;g__Alistipes	3.041%	1.008%	0.571%	0.104%	0.092%	0.000%	0.195%	0.085%	0.872%
d__Bacteria;p__Bacteroidota;c__Bacteroidia;o__Bacteroidales;f__Rikenellaceae;g__dgA-11_gut_group	0.000%	0.000%	0.000%	0.126%	0.000%	0.034%	0.071%	0.106%	0.155%
d__Bacteria;p__Bacteroidota;c__Bacteroidia;o__Bacteroidales;f__Rikenellaceae;g__Rikenellaceae_RC9_gut_group	0.000%	0.000%	0.000%	0.000%	0.012%	0.087%	0.354%	0.000%	0.145%
d__Bacteria;p__Bacteroidota;c__Bacteroidia;o__Bacteroidales;f__Tannerellaceae;g__Parabacteroides	1.611%	3.846%	1.459%	0.000%	0.367%	0.381%	0.264%	0.125%	0.644%
d__Bacteria;p__Desulfobacterota;c__Desulfovibrionia;o__Desulfovibrionales;f__Desulfovibrionaceae;g__Bilophila	2.846%	0.000%	3.477%	0.000%	0.000%	0.000%	0.185%	0.059%	0.567%
d__Bacteria;p__Desulfobacterota;c__Desulfovibrionia;o__Desulfovibrionales;f__Desulfovibrionaceae;g__Desulfovibrio	0.000%	0.000%	0.000%	0.000%	0.062%	0.000%	0.013%	0.000%	0.320%
d__Bacteria;p__Elusimicrobiota;c__Elusimicrobia;o__Elusimicrobiales;f__Elusimicrobiaceae;g__Elusimicrobium	0.000%	0.000%	0.487%	0.094%	0.071%	0.000%	0.216%	0.067%	1.429%
d__Bacteria;p__Firmicutes;c__Bacilli;o__Erysipelotrichales;f__Erysipelatoclostridiaceae;__	0.000%	0.000%	0.477%	0.198%	0.000%	0.059%	0.000%	0.000%	0.068%
d__Bacteria;p__Firmicutes;c__Bacilli;o__Erysipelotrichales;f__Erysipelatoclostridiaceae;g__Erysipelatoclostridiaceae	0.320%	0.109%	0.977%	0.000%	0.000%	0.227%	0.000%	0.000%	0.000%
d__Bacteria;p__Firmicutes;c__Bacilli;o__Erysipelotrichales;f__Erysipelotrichaceae;__	0.058%	0.000%	0.011%	8.051%	8.771%	7.800%	17.080%	17.980%	9.280%
d__Bacteria;p__Firmicutes;c__Bacilli;o__Erysipelotrichales;f__Erysipelotrichaceae;g__Allobaculum	0.000%	0.000%	0.130%	2.205%	5.085%	1.534%	1.273%	1.395%	0.828%
d__Bacteria;p__Firmicutes;c__Bacilli;o__Erysipelotrichales;f__Erysipelotrichaceae;g__Dubosiella	0.000%	0.000%	0.000%	0.000%	0.000%	0.135%	0.000%	0.069%	1.172%
d__Bacteria;p__Firmicutes;c__Bacilli;o__Erysipelotrichales;f__Erysipelotrichaceae;g__Faecalibaculum	0.054%	0.063%	1.094%	0.101%	0.000%	0.000%	0.141%	0.091%	0.000%
d__Bacteria;p__Firmicutes;c__Bacilli;o__Erysipelotrichales;f__Erysipelotrichaceae;g__Ileibacterium	0.012%	0.571%	0.338%	4.655%	3.905%	5.995%	2.607%	2.804%	2.441%
d__Bacteria;p__Firmicutes;c__Bacilli;o__Erysipelotrichales;f__Erysipelotrichaceae;g__uncultured	0.085%	0.000%	0.162%	2.676%	3.846%	0.858%	0.948%	0.964%	2.417%
d__Bacteria;p__Firmicutes;c__Bacilli;o__Lactobacillales;f__Lactobacillaceae;g__Lactobacillus	0.000%	5.801%	0.000%	2.015%	0.106%	0.020%	0.086%	0.218%	0.044%
d__Bacteria;p__Firmicutes;c__Bacilli;o__Lactobacillales;f__Streptococcaceae;g__Streptococcus	0.000%	1.861%	0.000%	1.532%	0.319%	0.129%	0.839%	0.269%	0.184%
d__Bacteria;p__Firmicutes;c__Bacilli;o__RF39;f__RF39;g__RF39	0.000%	0.000%	0.000%	0.201%	0.171%	0.390%	0.285%	0.035%	0.378%

Supplementary File 5
Relative Abundance Data

7DPT Fecal Genus Level Taxon Relative Abundance Continued	AC-PO7d	AC-10mg/kg	AC-5mg/kg	anti-rsPilA	anti-tip-chime	Ofloxacin	TS-30mg/kg	TS-15mg/kg	Saline
d__Bacteria;p__Firmicutes;c__Clostridia;o__Christensenellales;f__Christensenellaceae;__	0.170%	0.000%	0.168%	0.000%	0.000%	0.000%	0.000%	0.141%	0.000%
d__Bacteria;p__Firmicutes;c__Clostridia;o__Christensenellales;f__Christensenellaceae;g__Christensenellaceae_R-7_group	0.868%	3.275%	1.692%	3.471%	1.981%	5.033%	1.076%	5.149%	2.015%
d__Bacteria;p__Firmicutes;c__Clostridia;o__Clostridia;f__Hungateiclostridiaceae;g__Ruminiclostridium	0.107%	0.211%	0.211%	0.068%	0.027%	0.000%	0.017%	0.021%	0.000%
d__Bacteria;p__Firmicutes;c__Clostridia;o__Clostridia_UCG-014;f__Clostridia_UCG-014;g__Clostridia_UCG-014	0.034%	0.000%	0.192%	2.626%	0.302%	1.772%	1.126%	0.357%	0.809%
d__Bacteria;p__Firmicutes;c__Clostridia;o__Eubacteriales;f__Eubacteriaceae;g__uncultured	0.000%	0.000%	0.106%	3.212%	8.742%	4.354%	3.127%	5.530%	6.083%
d__Bacteria;p__Firmicutes;c__Clostridia;o__Lachnospirales;f__Defluviitaleaceae;g__Defluviitaleaceae_UCG-011	0.078%	0.000%	0.097%	0.374%	0.000%	0.000%	0.057%	0.077%	0.000%
d__Bacteria;p__Firmicutes;c__Clostridia;o__Lachnospirales;f__Lachnospiraceae;__	6.087%	7.997%	11.998%	4.061%	3.009%	5.308%	3.288%	6.856%	4.064%
d__Bacteria;p__Firmicutes;c__Clostridia;o__Lachnospirales;f__Lachnospiraceae;g__[Eubacterium]_ruminantium_group	0.000%	0.281%	0.000%	0.115%	0.245%	0.437%	0.067%	0.817%	0.218%
d__Bacteria;p__Firmicutes;c__Clostridia;o__Lachnospirales;f__Lachnospiraceae;g__[Ruminococcus]_torques_group	0.019%	0.094%	0.079%	0.000%	0.000%	0.202%	0.036%	0.099%	0.000%
d__Bacteria;p__Firmicutes;c__Clostridia;o__Lachnospirales;f__Lachnospiraceae;g__Blautia	0.562%	1.259%	3.550%	0.925%	0.053%	1.430%	0.327%	1.014%	1.235%
d__Bacteria;p__Firmicutes;c__Clostridia;o__Lachnospirales;f__Lachnospiraceae;g__Coprococcus	0.370%	1.337%	0.403%	0.896%	0.000%	0.364%	0.000%	0.029%	0.097%
d__Bacteria;p__Firmicutes;c__Clostridia;o__Lachnospirales;f__Lachnospiraceae;g__Dorea	0.071%	0.133%	0.051%	0.162%	0.000%	0.132%	0.094%	0.000%	0.000%
d__Bacteria;p__Firmicutes;c__Clostridia;o__Lachnospirales;f__Lachnospiraceae;g__Frisingicoccus	0.087%	0.172%	0.095%	0.374%	0.000%	0.712%	0.038%	0.719%	0.228%
d__Bacteria;p__Firmicutes;c__Clostridia;o__Lachnospirales;f__Lachnospiraceae;g__Lachnoclostridium	1.016%	0.000%	0.875%	0.835%	0.000%	1.004%	0.094%	1.102%	2.736%
d__Bacteria;p__Firmicutes;c__Clostridia;o__Lachnospirales;f__Lachnospiraceae;g__Lachnospira	0.000%	0.250%	0.000%	0.716%	0.000%	0.000%	0.073%	0.027%	0.242%
d__Bacteria;p__Firmicutes;c__Clostridia;o__Lachnospirales;f__Lachnospiraceae;g__Lachnospiraceae_NK4A136_group	1.250%	0.070%	1.665%	0.198%	0.390%	0.463%	0.480%	0.418%	0.223%
d__Bacteria;p__Firmicutes;c__Clostridia;o__Lachnospirales;f__Lachnospiraceae;g__Lachnospiraceae_UCG-001	0.000%	0.000%	0.000%	0.000%	0.497%	0.039%	0.000%	0.000%	0.000%
d__Bacteria;p__Firmicutes;c__Clostridia;o__Lachnospirales;f__Lachnospiraceae;g__Lachnospiraceae_UCG-004	0.099%	0.000%	0.198%	0.000%	0.000%	0.000%	0.000%	0.000%	0.068%
d__Bacteria;p__Firmicutes;c__Clostridia;o__Lachnospirales;f__Lachnospiraceae;g__Lachnospiraceae_UCG-010	0.000%	0.000%	0.000%	0.000%	0.062%	0.000%	0.000%	0.000%	0.000%
d__Bacteria;p__Firmicutes;c__Clostridia;o__Lachnospirales;f__Lachnospiraceae;g__Marvinbryantia	0.590%	0.000%	0.233%	0.227%	0.000%	0.000%	0.099%	0.000%	0.349%
d__Bacteria;p__Firmicutes;c__Clostridia;o__Lachnospirales;f__Lachnospiraceae;g__uncultured	0.000%	0.000%	0.000%	0.144%	0.000%	0.098%	0.174%	0.000%	0.000%
d__Bacteria;p__Firmicutes;c__Clostridia;o__Monoglobales;f__Monoglobaceae;g__Monoglobus	0.045%	3.760%	0.579%	0.989%	0.656%	0.816%	0.990%	0.394%	1.046%
d__Bacteria;p__Firmicutes;c__Clostridia;o__Oscillospirales;__;__	0.000%	0.227%	0.000%	0.036%	0.030%	0.000%	0.061%	0.141%	0.000%
d__Bacteria;p__Firmicutes;c__Clostridia;o__Oscillospirales;f__[Eubacterium]_coprostanoligenes_group;g__[Eubacterium]_coprostanoligenes_group	0.162%	0.680%	0.815%	0.633%	0.030%	1.511%	0.522%	0.317%	0.247%
d__Bacteria;p__Firmicutes;c__Clostridia;o__Oscillospirales;f__Butyricicoccaceae;g__UCG-008	0.150%	0.321%	0.000%	0.626%	0.204%	0.000%	0.113%	0.000%	0.092%
d__Bacteria;p__Firmicutes;c__Clostridia;o__Oscillospirales;f__Oscillospiraceae;__	0.640%	0.586%	0.208%	0.536%	0.239%	0.376%	0.430%	0.277%	1.206%
d__Bacteria;p__Firmicutes;c__Clostridia;o__Oscillospirales;f__Oscillospiraceae;g__Colidextribacter	0.131%	0.907%	0.996%	0.201%	0.597%	0.000%	0.161%	0.088%	0.000%
d__Bacteria;p__Firmicutes;c__Clostridia;o__Oscillospirales;f__Oscillospiraceae;g__NK4A214_group	0.000%	0.000%	0.165%	0.640%	0.245%	0.118%	0.105%	0.226%	0.605%
d__Bacteria;p__Firmicutes;c__Clostridia;o__Oscillospirales;f__Oscillospiraceae;g__Oscillibacter	0.000%	0.000%	0.230%	0.000%	0.059%	0.000%	0.111%	0.000%	0.213%
d__Bacteria;p__Firmicutes;c__Clostridia;o__Oscillospirales;f__Oscillospiraceae;g__Papillibacter	0.000%	0.000%	0.165%	0.000%	0.000%	0.000%	0.000%	0.056%	0.150%
d__Bacteria;p__Firmicutes;c__Clostridia;o__Oscillospirales;f__Oscillospiraceae;g__UCG-005	0.639%	0.563%	1.627%	2.500%	0.603%	1.705%	0.900%	1.025%	6.989%
d__Bacteria;p__Firmicutes;c__Clostridia;o__Oscillospirales;f__Oscillospiraceae;g__uncultured	2.291%	1.188%	1.200%	1.079%	0.627%	0.684%	0.673%	0.274%	0.751%
d__Bacteria;p__Firmicutes;c__Clostridia;o__Oscillospirales;f__Ruminococcaceae;__	0.000%	0.000%	0.000%	0.130%	0.000%	0.022%	0.117%	0.093%	0.000%
d__Bacteria;p__Firmicutes;c__Clostridia;o__Oscillospirales;f__Ruminococcaceae;g__[Eubacterium]_siraeum_group	0.000%	0.000%	0.000%	0.000%	0.304%	0.000%	0.147%	0.000%	0.000%
d__Bacteria;p__Firmicutes;c__Clostridia;o__Oscillospirales;f__Ruminococcaceae;g__Incertae_Sedis	0.000%	0.735%	0.087%	2.860%	1.239%	0.451%	1.428%	1.299%	0.489%
d__Bacteria;p__Firmicutes;c__Clostridia;o__Oscillospirales;f__Ruminococcaceae;g__Ruminococcaceae	0.000%	0.000%	0.000%	0.000%	0.000%	0.076%	0.046%	0.000%	0.000%
d__Bacteria;p__Firmicutes;c__Clostridia;o__Oscillospirales;f__Ruminococcaceae;g__Ruminococcus	0.438%	2.619%	3.114%	1.658%	0.928%	4.455%	2.542%	2.441%	1.637%
d__Bacteria;p__Firmicutes;c__Clostridia;o__Oscillospirales;f__Ruminococcaceae;g__UBA1819	4.272%	0.000%	1.495%	0.000%	0.000%	0.050%	0.000%	0.146%	0.000%
d__Bacteria;p__Firmicutes;c__Clostridia;o__Peptostreptococcales-Tissierellales;f__Anaerovoracaceae;g__[Eubacterium]_brachy_group	0.000%	0.000%	0.000%	0.191%	0.000%	0.000%	0.050%	0.043%	0.160%
d__Bacteria;p__Firmicutes;c__Clostridia;o__Peptostreptococcales-Tissierellales;f__Anaerovoracaceae;g__[Eubacterium]_nodatum_group	0.042%	0.000%	0.095%	0.471%	0.222%	0.258%	0.000%	0.130%	0.000%
d__Bacteria;p__Firmicutes;c__Clostridia;o__Peptostreptococcales-Tissierellales;f__Anaerovoracaceae;g__Family_XIII_AD3011_group	0.401%	0.211%	1.449%	0.921%	0.263%	0.401%	0.149%	0.418%	0.654%
d__Bacteria;p__Firmicutes;c__Clostridia;o__Peptostreptococcales-Tissierellales;f__Anaerovoracaceae;g__Mogibacterium	0.064%	0.000%	0.718%	0.212%	0.231%	0.171%	0.159%	0.490%	0.325%
d__Bacteria;p__Firmicutes;c__Negativicutes;o__Acidaminococcales;f__Acidaminococcaceae;g__Phascolarctobacterium	0.000%	2.064%	0.338%	0.651%	2.625%	0.353%	0.380%	0.525%	0.465%
d__Bacteria;p__Fusobacteriota;c__Fusobacteriia;o__Fusobacteriales;f__Fusobacteriaceae;g__Fusobacterium	24.909%	1.048%	7.089%	0.000%	0.000%	0.031%	0.000%	0.192%	1.414%
d__Bacteria;p__Patescibacteria;c__Saccharimonadia;o__Saccharimonadales;f__Saccharimonadaceae;g__Candidatus_Saccharimonas	0.022%	0.000%	0.024%	5.335%	11.630%	1.654%	15.522%	1.608%	0.877%
d__Bacteria;p__Planctomycetota;c__Planctomycetes;o__Pirellulales;f__Pirellulaceae;g__p-1088-a5_gut_group	0.172%	1.329%	0.122%	0.158%	0.381%	0.258%	0.145%	0.130%	0.063%
d__Bacteria;p__Proteobacteria;c__Gammaproteobacteria;o__Burkholderiales;f__Sutterellaceae;g__Parasutterella	1.028%	0.000%	0.642%	0.194%	0.027%	0.123%	0.642%	0.306%	0.387%
d__Bacteria;p__Proteobacteria;c__Gammaproteobacteria;o__Enterobacterales;f__Enterobacteriaceae;g__Escherichia-Shigella	2.832%	4.151%	0.487%	0.000%	0.035%	0.000%	0.306%	1.904%	0.891%
d__Bacteria;p__Proteobacteria;c__Gammaproteobacteria;o__Pasteurellales;f__Pasteurellaceae;g__Haemophilus	0.000%	0.000%	0.000%	0.000%	0.092%	0.255%	0.000%	0.000%	5.613%
d__Bacteria;p__Spirochaetota;c__Spirochaetia;o__Spirochaetales;f__Spirochaetaceae;g__Treponema	0.000%	0.000%	0.000%	2.450%	4.094%	0.693%	1.233%	0.019%	0.102%
d__Bacteria;p__Verrucomicrobiota;c__Verrucomicrobiae;o__Verrucomicrobiales;f__Akkermansiaceae;g__Akkermansia	0.000%	26.571%	0.000%	0.000%	1.922%	0.000%	0.197%	0.000%	0.431%

Supplementary File 5
Relative Abundance Data

9DPT Fecal Genus Level Taxon Relative Abundance	AC-PO7d	AC-10mg/kg	AC-5mg/kg	anti-rsPilA	anti-tip-chimer	Ofloxacin	TS-30mg/kg	TS-15mg/kg	Saline
d__Archaea;p__Euryarchaeota;c__Methanobacteria;o__Methanobacteriales;f__Methanobacteriaceae;g__Methanobrevibacter	5.942%	16.509%	7.276%	12.308%	4.207%	15.309%	6.790%	3.992%	1.299%
d__Archaea;p__Euryarchaeota;c__Methanobacteria;o__Methanobacteriales;f__Methanobacteriaceae;g__Methanosphaera	0.010%	0.000%	0.430%	0.000%	0.000%	0.000%	0.043%	0.290%	0.000%
d__Bacteria;p__Actinobacteriota;c__Actinobacteria;o__Actinomycetales;f__Actinomycetaceae;g__Actinomyces	0.000%	0.845%	0.000%	0.226%	0.039%	0.000%	0.041%	0.028%	0.000%
d__Bacteria;p__Actinobacteriota;c__Actinobacteria;o__Actinomycetales;f__Actinomycetaceae;g__Actinomycetaceae	0.146%	0.381%	0.122%	0.000%	0.000%	0.195%	0.000%	0.000%	0.000%
d__Bacteria;p__Actinobacteriota;c__Actinobacteria;o__Bifidobacteriales;f__Bifidobacteriaceae;g__Bifidobacterium	0.049%	0.000%	0.051%	0.573%	0.289%	0.000%	1.755%	2.119%	1.725%
d__Bacteria;p__Actinobacteriota;c__Coriobacteriia;o__Coriobacteriales;__;__	0.000%	0.000%	0.039%	0.205%	0.084%	0.065%	0.268%	0.197%	0.000%
d__Bacteria;p__Actinobacteriota;c__Coriobacteriia;o__Coriobacteriales;f__Atopobiaceae;__	0.055%	0.058%	0.043%	0.579%	1.091%	0.395%	1.078%	0.407%	2.260%
d__Bacteria;p__Actinobacteriota;c__Coriobacteriia;o__Coriobacteriales;f__Atopobiaceae;g__uncultured	0.052%	0.881%	0.495%	0.128%	0.050%	0.024%	0.108%	0.047%	0.180%
d__Bacteria;p__Actinobacteriota;c__Coriobacteriia;o__Coriobacteriales;f__Coriobacteriaceae;g__Collinsella	0.000%	0.073%	0.291%	0.000%	0.000%	0.024%	0.000%	0.000%	0.000%
d__Bacteria;p__Actinobacteriota;c__Coriobacteriia;o__Coriobacteriales;f__Coriobacteriales_Incertae_Sedis;g__uncultured	0.035%	0.663%	0.400%	0.202%	0.100%	0.000%	0.210%	0.201%	0.000%
d__Bacteria;p__Actinobacteriota;c__Coriobacteriia;o__Coriobacteriales;f__Eggerthellaceae;__	0.000%	0.131%	0.019%	0.896%	0.517%	0.168%	1.095%	0.912%	0.180%
d__Bacteria;p__Actinobacteriota;c__Coriobacteriia;o__Coriobacteriales;f__Eggerthellaceae;g__Adlercreutzia	0.369%	0.065%	0.486%	0.000%	0.000%	0.171%	0.000%	0.041%	0.000%
d__Bacteria;p__Actinobacteriota;c__Coriobacteriia;o__Coriobacteriales;f__Eggerthellaceae;g__Enterorhabdus	0.263%	1.037%	0.702%	0.700%	0.692%	0.068%	1.642%	0.626%	0.164%
d__Bacteria;p__Actinobacteriota;c__Coriobacteriia;o__Coriobacteriales;f__Eggerthellaceae;g__Paraeggerthella	0.000%	1.269%	6.805%	0.000%	0.000%	0.272%	0.000%	0.000%	0.000%
d__Bacteria;p__Actinobacteriota;c__Coriobacteriia;o__Coriobacteriales;f__Eggerthellaceae;g__uncultured	0.144%	1.305%	2.109%	1.362%	0.391%	1.995%	1.030%	1.771%	0.671%
d__Bacteria;p__Actinobacteriota;c__Coriobacteriia;o__Coriobacteriales;f__uncultured;g__uncultured	0.045%	2.291%	0.499%	0.030%	0.079%	0.038%	0.201%	0.172%	0.131%
d__Bacteria;p__Bacteroidota;c__Bacteroidia;o__Bacteroidales;__;__	0.212%	0.131%	0.133%	0.418%	0.118%	0.168%	0.199%	0.571%	0.360%
d__Bacteria;p__Bacteroidota;c__Bacteroidia;o__Bacteroidales;f__Bacteroidaceae;g__Bacteroides	28.972%	5.876%	7.708%	0.950%	0.443%	3.913%	0.537%	2.178%	0.775%
d__Bacteria;p__Bacteroidota;c__Bacteroidia;o__Bacteroidales;f__Marinifilaceae;g__Butyricimonas	0.356%	0.000%	0.056%	0.047%	0.010%	0.210%	0.000%	0.116%	0.000%
d__Bacteria;p__Bacteroidota;c__Bacteroidia;o__Bacteroidales;f__Marinifilaceae;g__Odoribacter	0.275%	0.000%	0.000%	0.000%	0.024%	0.000%	0.030%	0.063%	0.000%
d__Bacteria;p__Bacteroidota;c__Bacteroidia;o__Bacteroidales;f__Muribaculaceae;__	0.007%	0.790%	0.000%	0.309%	0.000%	0.000%	0.203%	0.000%	0.000%
d__Bacteria;p__Bacteroidota;c__Bacteroidia;o__Bacteroidales;f__Muribaculaceae;g__Muribaculaceae	5.333%	6.286%	8.809%	22.605%	21.153%	15.070%	16.789%	18.357%	21.832%
d__Bacteria;p__Bacteroidota;c__Bacteroidia;o__Bacteroidales;f__Muribaculaceae;g__Muribaculum	0.000%	0.116%	0.000%	0.092%	0.058%	0.083%	0.024%	0.385%	0.000%
d__Bacteria;p__Bacteroidota;c__Bacteroidia;o__Bacteroidales;f__Prevotellaceae;g__Prevotella	1.211%	1.929%	0.422%	0.715%	1.936%	1.718%	1.465%	0.873%	4.153%
d__Bacteria;p__Bacteroidota;c__Bacteroidia;o__Bacteroidales;f__Prevotellaceae;g__Prevotellaceae_Ga6A1_group	0.000%	0.000%	0.000%	0.113%	0.307%	0.204%	0.400%	0.233%	0.175%
d__Bacteria;p__Bacteroidota;c__Bacteroidia;o__Bacteroidales;f__Prevotellaceae;g__Prevotellaceae_NK3B31_group	0.000%	0.000%	1.098%	0.178%	0.887%	0.643%	0.283%	0.703%	1.697%
d__Bacteria;p__Bacteroidota;c__Bacteroidia;o__Bacteroidales;f__Prevotellaceae;g__Prevotellaceae_UCG-001	0.875%	0.903%	0.000%	1.223%	2.258%	3.654%	1.532%	2.154%	1.927%
d__Bacteria;p__Bacteroidota;c__Bacteroidia;o__Bacteroidales;f__Prevotellaceae;g__Prevotellaceae_UCG-003	0.000%	0.000%	0.000%	0.033%	0.131%	0.097%	0.000%	0.000%	0.000%
d__Bacteria;p__Bacteroidota;c__Bacteroidia;o__Bacteroidales;f__Rikenellaceae;g__Alistipes	0.828%	1.175%	0.383%	0.590%	0.029%	0.000%	0.266%	0.863%	0.131%
d__Bacteria;p__Bacteroidota;c__Bacteroidia;o__Bacteroidales;f__Rikenellaceae;g__dgA-11_gut_group	0.024%	0.036%	0.054%	0.270%	0.063%	0.094%	0.054%	0.278%	0.098%
d__Bacteria;p__Bacteroidota;c__Bacteroidia;o__Bacteroidales;f__Rikenellaceae;g__Rikenellaceae_RC9_gut_group	0.072%	0.000%	0.000%	0.024%	0.000%	0.183%	0.119%	0.049%	0.235%
d__Bacteria;p__Bacteroidota;c__Bacteroidia;o__Bacteroidales;f__Tannerellaceae;g__Parabacteroides	2.372%	0.881%	2.625%	0.220%	0.113%	0.384%	0.314%	0.330%	0.186%
d__Bacteria;p__Desulfobacterota;c__Desulfovibrionia;o__Desulfovibrionales;f__Desulfovibrionaceae;g__Bilophila	6.222%	0.000%	3.141%	0.000%	0.000%	0.000%	0.000%	0.215%	0.207%
d__Bacteria;p__Desulfobacterota;c__Desulfovibrionia;o__Desulfovibrionales;f__Desulfovibrionaceae;g__Desulfovibrio	0.000%	0.000%	0.000%	0.000%	0.268%	0.000%	0.000%	0.000%	0.082%
d__Bacteria;p__Elusimicrobiota;c__Elusimicrobia;o__Elusimicrobiales;f__Elusimicrobiaceae;g__Elusimicrobium	0.000%	0.181%	0.208%	0.418%	0.359%	0.059%	0.223%	0.059%	0.317%
d__Bacteria;p__Firmicutes;c__Bacilli;o__Erysipelotrichales;f__Erysipelatoclostridiaceae;__	0.000%	0.000%	0.060%	0.175%	0.000%	0.171%	0.000%	0.030%	0.098%
d__Bacteria;p__Firmicutes;c__Bacilli;o__Erysipelotrichales;f__Erysipelatoclostridiaceae;g__Erysipelatoclostridiaceae	0.095%	0.134%	0.317%	0.000%	0.013%	0.201%	0.000%	0.148%	0.000%
d__Bacteria;p__Firmicutes;c__Bacilli;o__Erysipelotrichales;f__Erysipelotrichaceae;__	0.175%	1.784%	0.118%	7.667%	8.076%	9.123%	13.630%	14.295%	20.784%
d__Bacteria;p__Firmicutes;c__Bacilli;o__Erysipelotrichales;f__Erysipelotrichaceae;g__Allobaculum	0.000%	0.000%	0.214%	1.952%	3.856%	2.160%	1.582%	0.873%	0.267%
d__Bacteria;p__Firmicutes;c__Bacilli;o__Erysipelotrichales;f__Erysipelotrichaceae;g__Dubosiella	0.000%	0.000%	0.000%	0.000%	0.000%	0.159%	0.000%	0.294%	0.000%
d__Bacteria;p__Firmicutes;c__Bacilli;o__Erysipelotrichales;f__Erysipelotrichaceae;g__Faecalibaculum	0.048%	2.066%	0.758%	0.116%	0.000%	0.000%	0.216%	0.049%	0.180%
d__Bacteria;p__Firmicutes;c__Bacilli;o__Erysipelotrichales;f__Erysipelotrichaceae;g__Ileibacterium	0.201%	4.002%	1.600%	5.813%	2.224%	8.612%	1.895%	1.576%	1.343%
d__Bacteria;p__Firmicutes;c__Bacilli;o__Erysipelotrichales;f__Erysipelotrichaceae;g__uncultured	0.116%	0.221%	0.137%	2.979%	2.919%	0.561%	1.458%	0.679%	1.255%
d__Bacteria;p__Firmicutes;c__Bacilli;o__Lactobacillales;f__Lactobacillaceae;g__Lactobacillus	0.000%	3.574%	0.056%	0.754%	0.506%	0.000%	0.327%	0.794%	8.143%
d__Bacteria;p__Firmicutes;c__Bacilli;o__Lactobacillales;f__Streptococcaceae;g__Streptococcus	0.032%	0.863%	0.060%	3.439%	0.438%	0.044%	1.365%	0.600%	0.180%
d__Bacteria;p__Firmicutes;c__Bacilli;o__RF39;f__RF39;g__RF39	0.000%	0.000%	0.000%	0.145%	0.244%	0.201%	0.266%	0.107%	0.164%

Supplementary File 5
Relative Abundance Data

9DPT Fecal Genus Level Taxon Relative Abundance Continued	AC-PO7d	AC-10mg/kg	AC-5mg/kg	anti-rsPilA	anti-tip-chimer	Ofloxacin	TS-30mg/kg	TS-15mg/kg	Saline
d__Bacteria;p__Firmicutes;c__Clostridia;o__Christensenellales;f__Christensenellaceae;__	0.244%	0.131%	0.225%	0.000%	0.000%	0.000%	0.000%	0.000%	0.000%
d__Bacteria;p__Firmicutes;c__Clostridia;o__Christensenellales;f__Christensenellaceae;g__Christensenellaceae_R-7_group	2.106%	3.759%	3.486%	3.890%	3.478%	2.942%	0.995%	5.005%	1.425%
d__Bacteria;p__Firmicutes;c__Clostridia;o__Clostridia;f__Hungateiclostridiaceae;g__Ruminiclostridium	0.151%	0.109%	0.167%	0.000%	0.000%	0.189%	0.000%	0.089%	0.000%
d__Bacteria;p__Firmicutes;c__Clostridia;o__Clostridia_UCG-014;f__Clostridia_UCG-014;g__Clostridia_UCG-014	0.274%	0.000%	0.163%	0.487%	0.976%	0.903%	0.829%	0.622%	0.431%
d__Bacteria;p__Firmicutes;c__Clostridia;o__Eubacteriales;f__Eubacteriaceae;g__uncultured	0.000%	1.443%	1.861%	3.878%	5.901%	4.099%	3.540%	4.271%	5.294%
d__Bacteria;p__Firmicutes;c__Clostridia;o__Lachnospirales;f__Defluviitaleaceae;g__Defluviitaleaceae_UCG-011	0.083%	0.167%	0.043%	0.724%	0.197%	0.000%	0.087%	0.257%	0.000%
d__Bacteria;p__Firmicutes;c__Clostridia;o__Lachnospirales;f__Lachnospiraceae;__	6.319%	6.583%	10.291%	2.398%	9.676%	3.509%	0.985%	3.327%	5.174%
d__Bacteria;p__Firmicutes;c__Clostridia;o__Lachnospirales;f__Lachnospiraceae;g__[Eubacterium]_ruminantium_group	0.000%	0.000%	0.171%	0.000%	0.370%	0.000%	0.110%	0.306%	0.344%
d__Bacteria;p__Firmicutes;c__Clostridia;o__Lachnospirales;f__Lachnospiraceae;g__[Ruminococcus]_torques_group	0.300%	0.000%	0.077%	0.000%	0.066%	0.059%	0.000%	0.000%	0.000%
d__Bacteria;p__Firmicutes;c__Clostridia;o__Lachnospirales;f__Lachnospiraceae;g__Blautia	0.818%	1.987%	4.098%	0.291%	0.163%	0.555%	0.169%	0.440%	0.497%
d__Bacteria;p__Firmicutes;c__Clostridia;o__Lachnospirales;f__Lachnospiraceae;g__Coprococcus	0.232%	0.950%	1.101%	0.196%	0.039%	0.741%	0.019%	0.000%	0.000%
d__Bacteria;p__Firmicutes;c__Clostridia;o__Lachnospirales;f__Lachnospiraceae;g__Dorea	0.117%	0.167%	0.096%	0.000%	0.047%	0.127%	0.041%	0.000%	0.000%
d__Bacteria;p__Firmicutes;c__Clostridia;o__Lachnospirales;f__Lachnospiraceae;g__Frisingicoccus	0.000%	0.044%	0.544%	0.053%	0.037%	0.077%	0.022%	0.371%	0.322%
d__Bacteria;p__Firmicutes;c__Clostridia;o__Lachnospirales;f__Lachnospiraceae;g__Lachnoclostridium	1.214%	0.439%	1.233%	0.659%	0.483%	0.844%	0.534%	0.681%	0.731%
d__Bacteria;p__Firmicutes;c__Clostridia;o__Lachnospirales;f__Lachnospiraceae;g__Lachnospira	0.000%	0.591%	0.199%	0.000%	0.000%	0.174%	0.000%	0.043%	0.000%
d__Bacteria;p__Firmicutes;c__Clostridia;o__Lachnospirales;f__Lachnospiraceae;g__Lachnospiraceae_NK4A136_group	1.944%	0.247%	0.400%	0.228%	1.196%	0.587%	0.149%	0.600%	0.300%
d__Bacteria;p__Firmicutes;c__Clostridia;o__Lachnospirales;f__Lachnospiraceae;g__Lachnospiraceae_UCG-001	0.000%	0.000%	0.000%	0.000%	0.635%	0.171%	0.000%	0.000%	0.426%
d__Bacteria;p__Firmicutes;c__Clostridia;o__Lachnospirales;f__Lachnospiraceae;g__Lachnospiraceae_UCG-004	0.202%	0.076%	0.304%	0.042%	0.089%	0.047%	0.000%	0.000%	0.000%
d__Bacteria;p__Firmicutes;c__Clostridia;o__Lachnospirales;f__Lachnospiraceae;g__Lachnospiraceae_UCG-010	0.000%	0.000%	0.236%	0.000%	0.000%	0.000%	0.000%	0.000%	0.000%
d__Bacteria;p__Firmicutes;c__Clostridia;o__Lachnospirales;f__Lachnospiraceae;g__Marvinbryantia	1.209%	0.468%	0.488%	0.231%	0.131%	0.201%	0.141%	0.000%	0.000%
d__Bacteria;p__Firmicutes;c__Clostridia;o__Lachnospirales;f__Lachnospiraceae;g__uncultured	0.000%	0.000%	0.047%	0.000%	0.155%	0.000%	0.229%	0.000%	0.000%
d__Bacteria;p__Firmicutes;c__Clostridia;o__Monoglobales;f__Monoglobaceae;g__Monoglobus	0.083%	3.172%	0.779%	0.543%	1.120%	0.431%	1.818%	0.030%	0.382%
d__Bacteria;p__Firmicutes;c__Clostridia;o__Oscillospirales;__;__	0.032%	0.932%	0.000%	0.074%	0.142%	0.151%	0.136%	0.172%	0.000%
d__Bacteria;p__Firmicutes;c__Clostridia;o__Oscillospirales;f__[Eubacterium]_coprostanoligenes_group;g__[Eubacterium]_coprostanoligenes_group	0.175%	0.250%	0.876%	0.033%	0.181%	0.581%	0.777%	0.438%	0.213%
d__Bacteria;p__Firmicutes;c__Clostridia;o__Oscillospirales;f__Butyricicoccaceae;g__UCG-008	0.236%	0.765%	0.109%	0.300%	0.372%	0.000%	0.028%	0.000%	0.775%
d__Bacteria;p__Firmicutes;c__Clostridia;o__Oscillospirales;f__Oscillospiraceae;__	0.705%	0.323%	0.482%	0.163%	0.569%	0.257%	0.476%	0.359%	0.693%
d__Bacteria;p__Firmicutes;c__Clostridia;o__Oscillospirales;f__Oscillospiraceae;g__Colidextribacter	1.085%	0.562%	0.394%	0.258%	0.784%	0.351%	0.158%	0.413%	0.000%
d__Bacteria;p__Firmicutes;c__Clostridia;o__Oscillospirales;f__Oscillospiraceae;g__NK4A214_group	0.151%	0.000%	0.036%	0.362%	0.643%	0.032%	0.206%	0.330%	0.257%
d__Bacteria;p__Firmicutes;c__Clostridia;o__Oscillospirales;f__Oscillospiraceae;g__Oscillibacter	0.134%	0.040%	0.084%	0.000%	0.000%	0.000%	0.052%	0.000%	0.000%
d__Bacteria;p__Firmicutes;c__Clostridia;o__Oscillospirales;f__Oscillospiraceae;g__Papillibacter	0.182%	0.000%	0.167%	0.000%	0.000%	0.000%	0.082%	0.030%	0.055%
d__Bacteria;p__Firmicutes;c__Clostridia;o__Oscillospirales;f__Oscillospiraceae;g__UCG-005	2.465%	2.360%	0.936%	1.409%	2.274%	1.136%	0.506%	0.691%	1.070%
d__Bacteria;p__Firmicutes;c__Clostridia;o__Oscillospirales;f__Oscillospiraceae;g__uncultured	0.473%	0.290%	3.655%	0.861%	0.918%	0.254%	1.045%	1.210%	0.480%
d__Bacteria;p__Firmicutes;c__Clostridia;o__Oscillospirales;f__Ruminococcaceae;__	0.000%	0.000%	0.000%	0.030%	0.029%	0.162%	0.000%	0.000%	0.000%
d__Bacteria;p__Firmicutes;c__Clostridia;o__Oscillospirales;f__Ruminococcaceae;g__[Eubacterium]_siraeum_group	0.000%	0.000%	0.000%	0.071%	0.278%	0.000%	0.355%	0.000%	0.000%
d__Bacteria;p__Firmicutes;c__Clostridia;o__Oscillospirales;f__Ruminococcaceae;g__Incertae_Sedis	0.062%	1.399%	0.193%	0.424%	1.700%	0.449%	1.387%	0.991%	0.895%
d__Bacteria;p__Firmicutes;c__Clostridia;o__Oscillospirales;f__Ruminococcaceae;g__Ruminococcaceae	0.000%	0.000%	0.000%	0.000%	0.100%	0.000%	0.076%	0.000%	0.000%
d__Bacteria;p__Firmicutes;c__Clostridia;o__Oscillospirales;f__Ruminococcaceae;g__Ruminococcus	5.275%	1.874%	2.874%	0.908%	4.257%	6.124%	1.690%	2.679%	5.338%
d__Bacteria;p__Firmicutes;c__Clostridia;o__Oscillospirales;f__Ruminococcaceae;g__UBA1819	0.482%	0.069%	2.056%	0.027%	0.000%	0.130%	0.000%	0.126%	0.000%
d__Bacteria;p__Firmicutes;c__Clostridia;o__Peptostreptococcales-Tissierellales;f__Anaerovoracaceae;g__[Eubacterium]_nodatum_group	0.034%	0.000%	0.619%	0.113%	0.294%	0.319%	0.000%	0.028%	0.000%
d__Bacteria;p__Firmicutes;c__Clostridia;o__Peptostreptococcales-Tissierellales;f__Anaerovoracaceae;g__Family_XIII_AD3011_group	0.100%	0.279%	2.364%	0.602%	0.294%	0.596%	0.249%	0.387%	0.502%
d__Bacteria;p__Firmicutes;c__Clostridia;o__Peptostreptococcales-Tissierellales;f__Anaerovoracaceae;g__Mogibacterium	0.083%	0.127%	2.525%	0.193%	0.139%	0.145%	0.219%	0.188%	0.033%
d__Bacteria;p__Firmicutes;c__Negativicutes;o__Acidaminococcales;f__Acidaminococcaceae;g__Phascolarctobacterium	0.000%	2.128%	0.246%	3.531%	0.365%	0.552%	0.508%	1.171%	0.688%
d__Bacteria;p__Fusobacteriota;c__Fusobacteriia;o__Fusobacteriales;f__Fusobacteriaceae;g__Fusobacterium	13.040%	0.149%	6.584%	0.000%	0.000%	0.156%	0.000%	0.197%	0.000%
d__Bacteria;p__Patescibacteria;c__Saccharimonadia;o__Saccharimonadales;f__Saccharimonadaceae;g__Candidatus_Saccharimonas	0.068%	0.000%	0.124%	6.537%	4.708%	2.157%	23.410%	8.729%	1.425%
d__Bacteria;p__Planctomycetota;c__Planctomycetes;o__Pirellulales;f__Pirellulaceae;g__p-1088-a5_gut_group	0.090%	0.460%	0.131%	0.193%	0.102%	0.133%	0.180%	0.103%	0.186%
d__Bacteria;p__Proteobacteria;c__Gammaproteobacteria;o__Burkholderiales;f__Sutterellaceae;g__Parasutterella	1.393%	0.439%	1.484%	0.374%	0.168%	0.274%	0.322%	0.241%	0.802%
d__Bacteria;p__Proteobacteria;c__Gammaproteobacteria;o__Enterobacterales;f__Enterobacteriaceae;g__Escherichia-Shigella	3.071%	0.203%	0.330%	0.068%	0.897%	0.024%	0.069%	1.497%	0.065%
d__Bacteria;p__Proteobacteria;c__Gammaproteobacteria;o__Pasteurellales;f__Pasteurellaceae;g__Haemophilus	0.828%	0.000%	0.000%	0.356%	0.191%	0.000%	0.000%	0.000%	0.000%
d__Bacteria;p__Spirochaetota;c__Spirochaetia;o__Spirochaetales;f__Spirochaetaceae;g__Treponema	0.000%	0.000%	0.000%	1.042%	0.934%	0.620%	0.961%	2.762%	0.000%
d__Bacteria;p__Verrucomicrobiota;c__Verrucomicrobiae;o__Verrucomicrobiales;f__Akkermansiaceae;g__Akkermansia	0.000%	10.567%	0.000%	0.000%	0.936%	3.305%	0.000%	3.538%	0.000%

Supplementary File 5
Relative Abundance Data

**Baseline NPL Genus Level Taxon Relative Abundance**	**AC-PO7d**	**AC-10mg/kg**	**AC-5mg/kg**	**anti-rsPilA**	**anti-tip-chime**	**Ofloxacin**	**TS-30mg/kg**	**TS-15mg/kg**	**Saline**
d__Archaea;p__Euryarchaeota;c__Methanobacteria;o__Methanobacteriales;f__Methanobacteriaceae;g__Methanobrevibacter	1.480%	0.000%	0.096%	0.853%	0.000%	0.000%	0.374%	0.225%	0.000%
d__Bacteria;p__Actinobacteriota;c__Actinobacteria;o__Actinomycetales;f__Actinomycetaceae;__	2.814%	4.605%	1.770%	2.864%	2.415%	5.137%	2.679%	4.012%	2.025%
d__Bacteria;p__Actinobacteriota;c__Actinobacteria;o__Actinomycetales;f__Actinomycetaceae;g__Mobiluncus	8.236%	5.345%	6.831%	8.745%	4.280%	4.727%	4.293%	8.772%	12.408%
d__Bacteria;p__Actinobacteriota;c__Actinobacteria;o__Bifidobacteriales;f__Bifidobacteriaceae;g__Bifidobacterium	1.471%	1.983%	1.487%	0.000%	1.063%	2.478%	0.252%	0.728%	0.984%
d__Bacteria;p__Actinobacteriota;c__Actinobacteria;o__Corynebacteriales;f__Corynebacteriaceae;g__Corynebacterium	0.000%	0.000%	0.407%	0.000%	0.382%	0.000%	2.033%	0.554%	0.000%
d__Bacteria;p__Actinobacteriota;c__Actinobacteria;o__Micrococcales;__;__	0.000%	1.286%	0.000%	0.800%	0.000%	0.000%	0.439%	0.000%	0.000%
d__Bacteria;p__Actinobacteriota;c__Coriobacteriia;o__Coriobacteriales;f__Atopobiaceae;g__Olsenella	0.841%	0.000%	0.345%	0.000%	0.000%	0.000%	0.000%	0.000%	0.106%
d__Bacteria;p__Bacteroidota;c__Bacteroidia;o__Bacteroidales;__;__	0.926%	30.131%	45.095%	28.312%	24.504%	28.060%	1.982%	32.189%	37.401%
d__Bacteria;p__Bacteroidota;c__Bacteroidia;o__Bacteroidales;f__Bacteroidaceae;g__Bacteroides	0.646%	0.000%	0.000%	0.000%	0.000%	0.907%	1.207%	0.000%	0.000%
d__Bacteria;p__Bacteroidota;c__Bacteroidia;o__Bacteroidales;f__Muribaculaceae;g__Muribaculaceae	4.144%	3.506%	2.460%	2.805%	1.666%	0.276%	2.666%	2.705%	2.098%
d__Bacteria;p__Bacteroidota;c__Bacteroidia;o__Bacteroidales;f__Prevotellaceae;__	0.315%	0.000%	0.000%	0.000%	0.000%	0.000%	0.000%	0.477%	1.073%
d__Bacteria;p__Bacteroidota;c__Bacteroidia;o__Bacteroidales;f__Prevotellaceae;g__Prevotellaceae_UCG-001	0.926%	0.000%	0.000%	0.238%	0.000%	0.000%	0.536%	0.000%	0.870%
d__Bacteria;p__Bacteroidota;c__Bacteroidia;o__Chitinophagales;f__Chitinophagaceae;__	11.154%	5.503%	4.077%	7.415%	5.594%	8.372%	1.594%	8.044%	4.537%
d__Bacteria;p__Bacteroidota;c__Bacteroidia;o__Sphingobacteriales;f__Sphingobacteriaceae;__	1.123%	3.463%	3.325%	2.818%	5.180%	5.153%	23.491%	1.262%	1.187%
d__Bacteria;p__Campilobacterota;c__Campylobacteria;o__Campylobacterales;f__Campylobacteraceae;g__Campylobacter	17.920%	19.312%	18.439%	16.710%	17.909%	21.502%	31.941%	18.542%	17.766%
d__Bacteria;p__Firmicutes;c__Bacilli;o__Erysipelotrichales;f__Erysipelotrichaceae;__	2.312%	1.128%	0.616%	0.370%	0.079%	0.000%	1.020%	2.054%	0.447%
d__Bacteria;p__Firmicutes;c__Bacilli;o__Erysipelotrichales;f__Erysipelotrichaceae;g__Ileibacterium	1.628%	0.129%	1.832%	2.620%	1.173%	0.497%	0.600%	1.211%	1.155%
d__Bacteria;p__Firmicutes;c__Bacilli;o__Lactobacillales;f__Aerococcaceae;g__Aerococcus	0.674%	0.000%	0.000%	0.708%	0.382%	0.679%	1.394%	0.000%	0.325%
d__Bacteria;p__Firmicutes;c__Bacilli;o__Lactobacillales;f__Streptococcaceae;g__Streptococcus	0.207%	0.000%	0.000%	1.713%	0.000%	0.000%	0.981%	0.174%	0.000%
d__Bacteria;p__Firmicutes;c__Bacilli;o__Mycoplasmatales;f__Mycoplasmataceae;g__Mycoplasma	1.518%	1.731%	0.995%	1.263%	2.404%	2.517%	2.388%	2.112%	2.171%
d__Bacteria;p__Firmicutes;c__Bacilli;o__Staphylococcales;f__Staphylococcaceae;g__Staphylococcus	24.326%	3.894%	1.691%	6.218%	2.310%	2.288%	6.978%	3.884%	5.244%
d__Bacteria;p__Firmicutes;c__Clostridia;o__Eubacteriales;f__Eubacteriaceae;g__uncultured	1.402%	1.236%	0.565%	0.000%	0.079%	0.087%	0.704%	0.000%	0.000%
d__Bacteria;p__Firmicutes;c__Clostridia;o__Lachnospirales;f__Lachnospiraceae;__	1.360%	1.185%	0.000%	1.085%	0.859%	0.000%	0.000%	0.348%	0.829%
d__Bacteria;p__Firmicutes;c__Clostridia;o__Peptostreptococcales-Tissierellales;f__Peptostreptococcales-Tissierellales;g__Peptoniphilus	0.369%	0.000%	0.000%	0.589%	0.000%	0.000%	0.000%	0.786%	0.000%
d__Bacteria;p__Fusobacteriota;c__Fusobacteriia;o__Fusobacteriales;f__Fusobacteriaceae;g__Fusobacterium	3.066%	6.480%	3.246%	2.911%	2.703%	7.954%	1.846%	4.637%	2.854%
d__Bacteria;p__Fusobacteriota;c__Fusobacteriia;o__Fusobacteriales;f__Leptotrichiaceae;__	3.033%	0.000%	0.000%	0.734%	2.949%	3.038%	1.937%	2.022%	1.504%
d__Bacteria;p__Patescibacteria;c__Saccharimonadia;o__Saccharimonadales;f__Saccharimonadales;g__Saccharimonadales	3.026%	1.839%	2.403%	1.872%	4.725%	1.192%	2.434%	1.410%	0.528%
d__Bacteria;p__Proteobacteria;c__Gammaproteobacteria;o__Burkholderiales;__;__	0.900%	4.541%	1.555%	4.406%	16.573%	2.115%	0.000%	3.188%	1.016%
d__Bacteria;p__Proteobacteria;c__Gammaproteobacteria;o__Enterobacterales;f__Enterobacteriaceae;__	0.937%	0.000%	1.640%	3.096%	0.990%	0.000%	4.687%	0.000%	1.862%
d__Bacteria;p__Proteobacteria;c__Gammaproteobacteria;o__Enterobacterales;f__Enterobacteriaceae;g__Escherichia-Shigella	1.868%	0.000%	0.000%	0.000%	0.162%	2.620%	1.046%	0.000%	0.000%
d__Bacteria;p__Proteobacteria;c__Gammaproteobacteria;o__Pasteurellales;f__Pasteurellaceae;g__Actinobacillus	1.015%	0.783%	0.000%	0.774%	0.424%	0.402%	0.000%	0.528%	1.610%
d__Bacteria;p__Proteobacteria;c__Gammaproteobacteria;o__Xanthomonadales;f__Xanthomonadaceae;g__Xanthomonas	0.364%	1.918%	1.125%	0.079%	1.194%	0.000%	0.497%	0.135%	0.000%

**2DPT NPL Genus Level Taxon Relative Abundance**	**AC-PO7d**	**AC-10mg/kg**	**AC-5mg/kg**	**anti-rsPilA**	**anti-tip-chime**	**Ofloxacin**	**TS-30mg/kg**	**TS-15mg/kg**	**Saline**
d__Archaea;p__Euryarchaeota;c__Methanobacteria;o__Methanobacteriales;f__Methanobacteriaceae;g__Methanobrevibacter	21.694%	0.476%	0.372%	0.679%	0.610%	0.000%	0.102%	1.339%	0.000%
d__Bacteria;p__Actinobacteriota;c__Actinobacteria;o__Actinomycetales;f__Actinomycetaceae;__	1.087%	7.163%	3.224%	3.644%	2.401%	4.947%	3.376%	3.732%	1.806%
d__Bacteria;p__Actinobacteriota;c__Actinobacteria;o__Actinomycetales;f__Actinomycetaceae;g__Mobiluncus	0.000%	4.279%	4.221%	6.812%	5.026%	5.688%	4.422%	9.512%	10.024%
d__Bacteria;p__Actinobacteriota;c__Actinobacteria;o__Bifidobacteriales;f__Bifidobacteriaceae;g__Bifidobacterium	1.706%	1.383%	1.412%	1.667%	0.783%	3.224%	0.000%	0.595%	3.385%
d__Bacteria;p__Actinobacteriota;c__Actinobacteria;o__Corynebacteriales;f__Corynebacteriaceae;g__Corynebacterium	0.000%	0.000%	0.000%	0.000%	0.000%	0.000%	3.550%	0.000%	0.000%
d__Bacteria;p__Actinobacteriota;c__Actinobacteria;o__Micrococcales;__;__	0.000%	0.000%	0.000%	0.000%	0.000%	0.000%	0.632%	0.351%	0.000%
d__Bacteria;p__Actinobacteriota;c__Coriobacteriia;o__Coriobacteriales;f__Atopobiaceae;g__Olsenella	0.000%	0.000%	0.287%	0.000%	0.295%	0.000%	0.000%	0.000%	0.071%
d__Bacteria;p__Bacteroidota;c__Bacteroidia;o__Bacteroidales;__;__	2.174%	20.093%	48.504%	33.391%	24.646%	32.307%	0.849%	33.816%	29.358%
d__Bacteria;p__Bacteroidota;c__Bacteroidia;o__Bacteroidales;f__Bacteroidaceae;g__Bacteroides	21.001%	0.000%	0.000%	0.000%	0.000%	0.000%	1.735%	0.000%	0.000%
d__Bacteria;p__Bacteroidota;c__Bacteroidia;o__Bacteroidales;f__Muribaculaceae;g__Muribaculaceae	0.319%	1.964%	1.026%	1.278%	1.613%	0.173%	1.496%	1.433%	3.261%
d__Bacteria;p__Bacteroidota;c__Bacteroidia;o__Bacteroidales;f__Prevotellaceae;__	0.000%	0.000%	0.000%	0.000%	0.239%	0.000%	0.000%	0.000%	0.195%
d__Bacteria;p__Bacteroidota;c__Bacteroidia;o__Bacteroidales;f__Prevotellaceae;g__Prevotellaceae_UCG-001	1.884%	0.000%	0.000%	0.000%	0.000%	0.000%	0.465%	0.000%	0.000%
d__Bacteria;p__Bacteroidota;c__Bacteroidia;o__Chitinophagales;f__Chitinophagaceae;__	0.422%	6.426%	2.805%	5.144%	4.344%	0.491%	0.995%	1.683%	5.451%
d__Bacteria;p__Bacteroidota;c__Bacteroidia;o__Sphingobacteriales;f__Sphingobacteriaceae;__	0.000%	0.574%	1.520%	3.168%	4.721%	3.411%	19.473%	1.561%	1.267%
d__Bacteria;p__Campilobacterota;c__Campylobacteria;o__Campylobacterales;f__Campylobacteraceae;g__Campylobacter	1.106%	19.512%	15.256%	16.198%	18.471%	14.745%	40.434%	18.344%	16.163%
d__Bacteria;p__Firmicutes;c__Bacilli;o__Erysipelotrichales;f__Erysipelotrichaceae;__	0.000%	1.194%	0.372%	0.000%	1.099%	0.291%	1.822%	0.903%	0.338%
d__Bacteria;p__Firmicutes;c__Bacilli;o__Erysipelotrichales;f__Erysipelotrichaceae;g__Ileibacterium	0.000%	0.894%	1.760%	0.988%	1.592%	0.000%	0.298%	2.700%	1.130%
d__Bacteria;p__Firmicutes;c__Bacilli;o__Lactobacillales;f__Aerococcaceae;g__Aerococcus	0.562%	0.000%	0.000%	1.124%	0.478%	0.754%	0.000%	1.117%	0.000%
d__Bacteria;p__Firmicutes;c__Bacilli;o__Lactobacillales;f__Streptococcaceae;g__Streptococcus	0.000%	0.000%	0.000%	2.964%	0.000%	0.000%	2.607%	0.294%	0.000%
d__Bacteria;p__Firmicutes;c__Bacilli;o__Mycoplasmatales;f__Mycoplasmataceae;g__Mycoplasma	3.458%	1.429%	2.395%	1.247%	0.961%	1.681%	3.006%	2.443%	2.644%
d__Bacteria;p__Firmicutes;c__Bacilli;o__Staphylococcales;f__Staphylococcaceae;g__Staphylococcus	0.000%	2.061%	3.534%	3.508%	8.287%	13.853%	4.451%	2.342%	15.098%
d__Bacteria;p__Firmicutes;c__Clostridia;o__Eubacteriales;f__Eubacteriaceae;g__uncultured	0.000%	1.474%	0.000%	0.000%	0.000%	0.000%	0.298%	1.103%	0.585%
d__Bacteria;p__Firmicutes;c__Clostridia;o__Lachnospirales;f__Lachnospiraceae;__	0.103%	0.000%	0.174%	0.000%	0.203%	1.439%	0.000%	2.177%	0.000%
d__Bacteria;p__Firmicutes;c__Clostridia;o__Peptostreptococcales-Tissierellales;f__Peptostreptococcales-Tissierellales;g__Peptoniphilus	1.200%	0.842%	0.113%	0.000%	0.188%	0.000%	0.065%	1.898%	0.754%
d__Bacteria;p__Fusobacteriota;c__Fusobacteriia;o__Fusobacteriales;f__Fusobacteriaceae;g__Fusobacterium	0.000%	3.334%	1.642%	3.279%	3.245%	7.591%	1.786%	4.821%	3.131%
d__Bacteria;p__Fusobacteriota;c__Fusobacteriia;o__Fusobacteriales;f__Leptotrichiaceae;__	0.319%	0.104%	0.000%	1.223%	3.108%	2.456%	0.828%	1.998%	1.397%
d__Bacteria;p__Patescibacteria;c__Saccharimonadia;o__Saccharimonadales;f__Saccharimonadales;g__Saccharimonadales	3.814%	2.942%	2.226%	1.593%	1.379%	0.962%	3.362%	0.924%	0.000%
d__Bacteria;p__Proteobacteria;c__Gammaproteobacteria;o__Burkholderiales;__;__	3.439%	21.489%	2.824%	8.448%	13.445%	3.197%	0.000%	4.183%	0.663%
d__Bacteria;p__Proteobacteria;c__Gammaproteobacteria;o__Enterobacterales;f__Enterobacteriaceae;__	3.196%	0.000%	3.835%	3.372%	1.241%	1.169%	2.352%	0.000%	1.592%
d__Bacteria;p__Proteobacteria;c__Gammaproteobacteria;o__Enterobacterales;f__Enterobacteriaceae;g__Escherichia-Shigella	32.284%	0.196%	0.664%	0.000%	0.209%	1.619%	1.597%	0.000%	0.000%
d__Bacteria;p__Proteobacteria;c__Gammaproteobacteria;o__Pasteurellales;f__Pasteurellaceae;g__Actinobacillus	0.234%	1.370%	0.640%	0.272%	0.814%	0.000%	0.000%	0.000%	0.773%
d__Bacteria;p__Proteobacteria;c__Gammaproteobacteria;o__Xanthomonadales;f__Xanthomonadaceae;g__Xanthomonas	0.000%	0.802%	1.195%	0.000%	0.600%	0.000%	0.000%	0.731%	0.916%

Supplementary File 5
Relative Abundance Data

**5DPT NPL Genus Level Taxon Relative Abundance**	**AC-PO7d**	**AC-10mg/kç**	**AC-5mg/kç**	**anti-rsPil/**	**anti-tip-chime**	**Ofloxacin**	**TS-30mg/kg**	**TS-15mg/kg**	**Saline**
d__Archaea;p__Euryarchaeota;c__Methanobacteria;o__Methanobacteriales;f__Methanobacteriaceae;g__Methanobrevibacter	0.686%	0.000%	0.000%	0.880%	0.437%	2.074%	0.000%	0.506%	0.000%
d__Bacteria;p__Actinobacteriota;c__Actinobacteria;o__Actinomycetales;f__Actinomycetaceae;__	0.395%	5.996%	2.075%	0.000%	0.921%	3.704%	3.623%	2.429%	1.978%
d__Bacteria;p__Actinobacteriota;c__Actinobacteria;o__Actinomycetales;f__Actinomycetaceae;g__Mobiluncus	2.891%	2.352%	3.903%	6.326%	5.185%	5.921%	6.052%	8.564%	11.529%
d__Bacteria;p__Actinobacteriota;c__Actinobacteria;o__Bifidobacteriales;f__Bifidobacteriaceae;g__Bifidobacterium	0.395%	1.491%	2.275%	0.617%	0.933%	2.742%	0.000%	0.000%	2.125%
d__Bacteria;p__Actinobacteriota;c__Actinobacteria;o__Corynebacteriales;f__Corynebacteriaceae;g__Corynebacterium	0.288%	0.000%	0.000%	0.000%	0.000%	0.000%	0.872%	0.000%	0.000%
d__Bacteria;p__Actinobacteriota;c__Actinobacteria;o__Micrococcales;__;__	0.000%	0.000%	0.000%	0.000%	0.000%	0.771%	0.000%	0.565%	0.000%
d__Bacteria;p__Actinobacteriota;c__Coriobacteriia;o__Coriobacteriales;f__Atopobiaceae;g__Olsenella	0.000%	0.116%	0.000%	0.263%	0.000%	0.000%	0.000%	0.000%	0.000%
d__Bacteria;p__Bacteroidota;c__Bacteroidia;o__Bacteroidales;__;__	1.386%	22.635%	43.332%	35.686%	26.643%	33.874%	0.164%	33.874%	23.044%
d__Bacteria;p__Bacteroidota;c__Bacteroidia;o__Bacteroidales;f__Bacteroidaceae;g__Bacteroides	12.157%	0.000%	0.000%	0.000%	0.000%	0.000%	2.475%	0.000%	0.000%
d__Bacteria;p__Bacteroidota;c__Bacteroidia;o__Bacteroidales;f__Muribaculaceae;g__Muribaculaceae	0.251%	0.232%	0.970%	2.748%	1.836%	0.334%	2.847%	2.935%	1.941%
d__Bacteria;p__Bacteroidota;c__Bacteroidia;o__Bacteroidales;f__Prevotellaceae;__	0.166%	0.000%	0.000%	0.000%	0.207%	0.000%	0.000%	0.000%	0.272%
d__Bacteria;p__Bacteroidota;c__Bacteroidia;o__Bacteroidales;f__Prevotellaceae;g__Prevotellaceae_UCG-001	0.000%	0.000%	0.000%	0.107%	0.366%	0.000%	0.000%	1.476%	0.324%
d__Bacteria;p__Bacteroidota;c__Bacteroidia;o__Chitinophagales;f__Chitinophagaceae;__	2.865%	5.036%	3.305%	3.620%	4.322%	3.290%	0.000%	2.641%	5.699%
d__Bacteria;p__Bacteroidota;c__Bacteroidia;o__Sphingobacteriales;f__Sphingobacteriaceae;__	0.227%	1.698%	8.243%	1.440%	7.133%	0.763%	23.241%	2.400%	1.375%
d__Bacteria;p__Campilobacterota;c__Campylobacteria;o__Campylobacterales;f__Campylobacteraceae;g__Campylobacter	5.511%	11.363%	13.919%	17.070%	17.136%	17.136%	30.270%	17.687%	17.919%
d__Bacteria;p__Firmicutes;c__Bacilli;o__Erysipelotrichales;f__Erysipelotrichaceae;__	0.694%	0.348%	0.221%	0.962%	1.175%	2.734%	2.467%	3.065%	0.735%
d__Bacteria;p__Firmicutes;c__Bacilli;o__Erysipelotrichales;f__Erysipelotrichaceae;g__Ileibacterium	0.197%	0.340%	1.245%	3.463%	1.305%	3.688%	1.617%	1.776%	0.176%
d__Bacteria;p__Firmicutes;c__Bacilli;o__Lactobacillales;f__Aerococcaceae;g__Aerococcus	0.467%	0.000%	0.000%	1.234%	1.394%	0.000%	0.440%	1.212%	0.000%
d__Bacteria;p__Firmicutes;c__Bacilli;o__Lactobacillales;f__Streptococcaceae;g__Streptococcus	0.467%	0.000%	0.000%	0.946%	0.000%	0.000%	2.654%	0.347%	7.478%
d__Bacteria;p__Firmicutes;c__Bacilli;o__Mycoplasmatales;f__Mycoplasmataceae;g__Mycoplasma	0.725%	0.000%	2.415%	0.173%	1.860%	2.329%	3.548%	1.241%	1.176%
d__Bacteria;p__Firmicutes;c__Bacilli;o__Staphylococcales;f__Staphylococcaceae;g__Staphylococcus	3.159%	7.197%	2.582%	11.698%	4.340%	6.700%	2.236%	5.129%	13.257%
d__Bacteria;p__Firmicutes;c__Clostridia;o__Eubacteriales;f__Eubacteriaceae;g__uncultured	0.227%	0.389%	0.000%	0.000%	0.189%	1.613%	0.000%	0.641%	0.000%
d__Bacteria;p__Firmicutes;c__Clostridia;o__Lachnospirales;f__Lachnospiraceae;__	0.000%	0.000%	0.000%	0.000%	0.000%	0.000%	0.000%	0.835%	0.000%
d__Bacteria;p__Firmicutes;c__Clostridia;o__Peptostreptococcales-Tissierellales;f__Peptostreptococcales-Tissierellales;g__Peptoniphilus	0.520%	0.779%	0.000%	0.000%	0.000%	0.064%	0.000%	0.682%	0.000%
d__Bacteria;p__Fusobacteriota;c__Fusobacteriia;o__Fusobacteriales;f__Fusobacteriaceae;g__Fusobacterium	0.255%	2.501%	1.288%	0.000%	3.915%	6.485%	3.175%	5.182%	2.750%
d__Bacteria;p__Fusobacteriota;c__Fusobacteriia;o__Fusobacteriales;f__Leptotrichiaceae;__	0.022%	2.849%	0.000%	0.000%	1.222%	0.000%	1.849%	1.047%	3.287%
d__Bacteria;p__Patescibacteria;c__Saccharimonadia;o__Saccharimonadales;f__Saccharimonadales;g__Saccharimonadales	6.063%	2.418%	3.024%	0.000%	2.409%	1.947%	6.142%	0.000%	0.000%
d__Bacteria;p__Proteobacteria;c__Gammaproteobacteria;o__Burkholderiales;__;__	2.729%	31.000%	6.038%	10.053%	15.335%	0.747%	0.000%	4.006%	2.029%
d__Bacteria;p__Proteobacteria;c__Gammaproteobacteria;o__Enterobacterales;f__Enterobacteriaceae;__	1.574%	0.000%	3.876%	2.715%	0.000%	1.113%	6.328%	0.000%	1.853%
d__Bacteria;p__Proteobacteria;c__Gammaproteobacteria;o__Enterobacterales;f__Enterobacteriaceae;g__Escherichia-Shigella	55.494%	1.259%	0.000%	0.000%	0.283%	1.971%	0.000%	0.523%	0.000%
d__Bacteria;p__Proteobacteria;c__Gammaproteobacteria;o__Pasteurellales;f__Pasteurellaceae;g__Actinobacillus	0.000%	0.000%	0.270%	0.000%	0.000%	0.000%	0.000%	0.000%	0.000%
d__Bacteria;p__Proteobacteria;c__Gammaproteobacteria;o__Xanthomonadales;f__Xanthomonadaceae;g__Xanthomonas	0.188%	0.000%	1.019%	0.000%	1.453%	0.000%	0.000%	1.235%	1.051%

**7DPT NPL Genus Level Taxon Relative Abundance**	**AC-PO7d**	**AC-10mg/kç**	**AC-5mg/kç**	**anti-rsPil/**	**anti-tip-chime**	**Ofloxacin**	**TS-30mg/kg**	**TS-15mg/kg**	**Saline**
d__Archaea;p__Euryarchaeota;c__Methanobacteria;o__Methanobacteriales;f__Methanobacteriaceae;g__Methanobrevibacter	0.152%	0.000%	0.000%	0.872%	0.536%	0.210%	0.000%	1.032%	0.000%
d__Bacteria;p__Actinobacteriota;c__Actinobacteria;o__Actinomycetales;f__Actinomycetaceae;__	0.047%	4.959%	2.007%	0.000%	1.762%	5.381%	1.697%	2.198%	2.485%
d__Bacteria;p__Actinobacteriota;c__Actinobacteria;o__Actinomycetales;f__Actinomycetaceae;g__Mobiluncus	1.969%	0.000%	4.320%	6.219%	5.521%	5.555%	5.703%	7.238%	9.578%
d__Bacteria;p__Actinobacteriota;c__Actinobacteria;o__Bifidobacteriales;f__Bifidobacteriaceae;g__Bifidobacterium	1.127%	0.000%	2.367%	0.000%	1.691%	2.285%	0.000%	0.570%	1.062%
d__Bacteria;p__Actinobacteriota;c__Actinobacteria;o__Corynebacteriales;f__Corynebacteriaceae;g__Corynebacterium	0.162%	0.000%	0.000%	0.000%	0.000%	0.000%	0.000%	0.000%	0.000%
d__Bacteria;p__Actinobacteriota;c__Actinobacteria;o__Micrococcales;__;__	0.000%	0.000%	0.000%	0.000%	0.000%	0.960%	0.684%	0.505%	0.000%
d__Bacteria;p__Actinobacteriota;c__Coriobacteriia;o__Coriobacteriales;f__Atopobiaceae;g__Olsenella	0.000%	0.000%	0.118%	0.366%	0.000%	0.000%	0.000%	0.000%	0.000%
d__Bacteria;p__Bacteroidota;c__Bacteroidia;o__Bacteroidales;__;__	13.046%	27.416%	43.405%	15.839%	27.770%	24.108%	0.599%	33.914%	27.310%
d__Bacteria;p__Bacteroidota;c__Bacteroidia;o__Bacteroidales;f__Bacteroidaceae;g__Bacteroides	4.024%	0.000%	0.000%	0.000%	0.000%	0.888%	3.792%	0.553%	0.000%
d__Bacteria;p__Bacteroidota;c__Bacteroidia;o__Bacteroidales;f__Muribaculaceae;g__Muribaculaceae	0.017%	0.329%	0.561%	4.927%	2.010%	0.162%	5.888%	3.402%	2.903%
d__Bacteria;p__Bacteroidota;c__Bacteroidia;o__Bacteroidales;f__Prevotellaceae;__	0.442%	0.000%	0.000%	0.000%	0.000%	0.000%	0.000%	0.000%	0.000%
d__Bacteria;p__Bacteroidota;c__Bacteroidia;o__Bacteroidales;f__Prevotellaceae;g__Prevotellaceae_UCG-001	0.000%	0.000%	0.044%	0.226%	0.000%	0.000%	0.000%	0.000%	0.000%
d__Bacteria;p__Bacteroidota;c__Bacteroidia;o__Chitinophagales;f__Chitinophagaceae;__	2.301%	2.592%	3.675%	2.265%	4.174%	2.957%	0.000%	3.031%	5.911%
d__Bacteria;p__Bacteroidota;c__Bacteroidia;o__Sphingobacteriales;f__Sphingobacteriaceae;__	1.085%	3.710%	2.588%	0.872%	4.902%	3.965%	8.212%	3.025%	1.198%
d__Bacteria;p__Campilobacterota;c__Campylobacteria;o__Campylobacterales;f__Campylobacteraceae;g__Campylobacter	8.640%	5.898%	22.549%	6.709%	20.328%	17.857%	31.908%	20.291%	16.630%
d__Bacteria;p__Firmicutes;c__Bacilli;o__Erysipelotrichales;f__Erysipelotrichaceae;__	0.000%	0.000%	0.000%	2.709%	0.657%	0.000%	4.562%	0.285%	0.000%
d__Bacteria;p__Firmicutes;c__Bacilli;o__Erysipelotrichales;f__Erysipelotrichaceae;g__Ileibacterium	0.060%	0.178%	0.566%	4.343%	1.959%	0.450%	1.868%	1.198%	0.161%
d__Bacteria;p__Firmicutes;c__Bacilli;o__Lactobacillales;f__Aerococcaceae;g__Aerococcus	0.000%	0.000%	0.000%	1.300%	0.945%	0.000%	0.927%	1.091%	0.000%
d__Bacteria;p__Firmicutes;c__Bacilli;o__Lactobacillales;f__Streptococcaceae;g__Streptococcus	0.000%	0.000%	0.000%	0.950%	0.096%	0.000%	6.459%	0.000%	8.460%
d__Bacteria;p__Firmicutes;c__Bacilli;o__Mycoplasmatales;f__Mycoplasmataceae;g__Mycoplasma	0.881%	0.000%	1.786%	0.000%	1.411%	1.302%	0.000%	0.849%	1.375%
d__Bacteria;p__Firmicutes;c__Bacilli;o__Staphylococcales;f__Staphylococcaceae;g__Staphylococcus	10.502%	16.446%	3.715%	39.314%	6.797%	23.688%	2.923%	9.372%	10.374%
d__Bacteria;p__Firmicutes;c__Clostridia;o__Eubacteriales;f__Eubacteriaceae;g__uncultured	0.000%	0.085%	0.000%	0.833%	0.000%	0.000%	2.082%	0.602%	0.949%
d__Bacteria;p__Firmicutes;c__Clostridia;o__Peptostreptococcales-Tissierellales;f__Peptostreptococcales-Tissierellales;g__Peptoniphilus	0.476%	0.000%	0.000%	0.000%	0.000%	0.000%	0.000%	0.403%	0.000%
d__Bacteria;p__Fusobacteriota;c__Fusobacteriia;o__Fusobacteriales;f__Fusobacteriaceae;g__Fusobacterium	0.000%	4.452%	2.371%	0.000%	3.115%	5.998%	1.896%	5.057%	4.463%
d__Bacteria;p__Fusobacteriota;c__Fusobacteriia;o__Fusobacteriales;f__Leptotrichiaceae;__	0.000%	3.212%	0.000%	0.000%	1.768%	1.176%	1.041%	1.048%	3.112%
d__Bacteria;p__Patescibacteria;c__Saccharimonadia;o__Saccharimonadales;f__Saccharimonadales;g__Saccharimonadales	4.364%	1.644%	3.498%	0.000%	3.517%	1.092%	4.890%	1.064%	0.040%
d__Bacteria;p__Proteobacteria;c__Gammaproteobacteria;o__Burkholderiales;__;__	8.451%	25.641%	3.331%	8.881%	9.720%	1.146%	0.000%	2.880%	1.753%
d__Bacteria;p__Proteobacteria;c__Gammaproteobacteria;o__Enterobacterales;f__Enterobacteriaceae;__	5.591%	2.113%	2.603%	3.067%	0.000%	0.000%	9.952%	0.000%	1.456%
d__Bacteria;p__Proteobacteria;c__Gammaproteobacteria;o__Enterobacterales;f__Enterobacteriaceae;g__Escherichia-Shigella	36.434%	0.000%	0.113%	0.000%	0.000%	0.786%	4.919%	0.339%	0.000%
d__Bacteria;p__Proteobacteria;c__Gammaproteobacteria;o__Pasteurellales;f__Pasteurellaceae;g__Actinobacillus	0.000%	0.394%	0.093%	0.311%	0.255%	0.036%	0.000%	0.054%	0.273%
d__Bacteria;p__Proteobacteria;c__Gammaproteobacteria;o__Xanthomonadales;f__Xanthomonadaceae;g__Xanthomonas	0.230%	0.930%	0.290%	0.000%	1.066%	0.000%	0.000%	0.000%	0.507%

Supplementary File 5
Relative Abundance Data

9DPT NPL Genus Level Taxon Abundance	AC-PO7d	AC-10mg/kg	AC-5mg/kg	anti-rsPilA	anti-tip-chime	Ofloxacin	TS-30mg/kg	TS-15mg/kg	Saline
d__Archaea;p__Euryarchaeota;c__Methanobacteria;o__Methanobacteriales;f__Methanobacteriaceae;g__Methanobrevibacter	0.000%	0.062%	0.000%	0.000%	0.208%	0.000%	0.000%	0.090%	0.000%
d__Bacteria;p__Actinobacteriota;c__Actinobacteria;o__Actinomycetales;f__Actinomycetaceae;__	0.088%	2.449%	2.386%	0.000%	0.743%	2.968%	3.443%	1.550%	2.122%
d__Bacteria;p__Actinobacteriota;c__Actinobacteria;o__Actinomycetales;f__Actinomycetaceae;g__Mobiluncus	1.518%	1.573%	4.850%	6.133%	5.326%	4.925%	6.995%	7.275%	9.484%
d__Bacteria;p__Actinobacteriota;c__Actinobacteria;o__Bifidobacteriales;f__Bifidobacteriaceae;g__Bifidobacterium	0.736%	0.721%	1.499%	0.905%	1.445%	1.855%	0.000%	0.000%	2.722%
d__Bacteria;p__Actinobacteriota;c__Actinobacteria;o__Corynebacteriales;f__Corynebacteriaceae;g__Corynebacterium	0.280%	0.000%	0.000%	0.000%	0.000%	0.000%	0.504%	0.853%	0.000%
d__Bacteria;p__Actinobacteriota;c__Actinobacteria;o__Micrococcales;__;__	0.000%	0.000%	0.000%	0.000%	0.332%	0.000%	0.000%	0.505%	0.000%
d__Bacteria;p__Actinobacteriota;c__Coriobacteriia;o__Coriobacteriales;f__Atopobiaceae;g__Olsenella	0.017%	0.000%	0.121%	0.000%	0.000%	0.000%	0.000%	0.000%	0.000%
d__Bacteria;p__Bacteroidota;c__Bacteroidia;o__Bacteroidales;__;__	4.035%	22.472%	42.793%	35.394%	21.141%	19.970%	0.879%	33.279%	26.433%
d__Bacteria;p__Bacteroidota;c__Bacteroidia;o__Bacteroidales;f__Bacteroidaceae;g__Bacteroides	1.168%	0.163%	0.000%	0.000%	0.000%	0.422%	2.417%	1.556%	0.000%
d__Bacteria;p__Bacteroidota;c__Bacteroidia;o__Bacteroidales;f__Muribaculaceae;g__Muribaculaceae	0.045%	0.062%	0.728%	1.461%	3.054%	0.000%	2.381%	1.796%	1.817%
d__Bacteria;p__Bacteroidota;c__Bacteroidia;o__Bacteroidales;f__Prevotellaceae;__	0.235%	0.000%	0.276%	0.000%	0.000%	0.000%	0.000%	0.354%	0.000%
d__Bacteria;p__Bacteroidota;c__Bacteroidia;o__Bacteroidales;f__Prevotellaceae;g__Prevotellaceae_UCG-001	0.000%	0.000%	0.000%	0.000%	0.000%	0.000%	0.000%	0.781%	0.127%
d__Bacteria;p__Bacteroidota;c__Bacteroidia;o__Chitinophagales;f__Chitinophagaceae;__	1.658%	1.713%	3.895%	2.715%	3.490%	3.221%	0.046%	3.472%	6.974%
d__Bacteria;p__Bacteroidota;c__Bacteroidia;o__Sphingobacteriales;f__Sphingobacteriaceae;__	0.638%	4.704%	2.910%	1.085%	5.034%	3.854%	13.020%	3.310%	0.558%
d__Bacteria;p__Campilobacterota;c__Campylobacteria;o__Campylobacterales;f__Campylobacteraceae;g__Campylobacter	4.701%	5.734%	20.138%	16.514%	17.865%	14.328%	35.836%	19.259%	22.485%
d__Bacteria;p__Firmicutes;c__Bacilli;o__Erysipelotrichales;f__Erysipelotrichaceae;__	0.000%	0.000%	0.000%	0.572%	1.970%	0.000%	2.390%	0.619%	1.843%
d__Bacteria;p__Firmicutes;c__Bacilli;o__Erysipelotrichales;f__Erysipelotrichaceae;g__Ileibacterium	0.021%	0.000%	0.490%	1.565%	2.213%	0.439%	0.330%	2.667%	0.000%
d__Bacteria;p__Firmicutes;c__Bacilli;o__Lactobacillales;f__Aerococcaceae;g__Aerococcus	0.000%	0.000%	0.000%	0.000%	0.639%	0.000%	2.948%	1.970%	0.000%
d__Bacteria;p__Firmicutes;c__Bacilli;o__Lactobacillales;f__Streptococcaceae;g__Streptococcus	0.136%	0.000%	0.000%	1.030%	0.000%	0.000%	2.500%	0.000%	1.530%
d__Bacteria;p__Firmicutes;c__Bacilli;o__Mycoplasmatales;f__Mycoplasmataceae;g__Mycoplasma	0.456%	0.194%	1.664%	0.000%	1.733%	1.341%	1.950%	2.127%	1.209%
d__Bacteria;p__Firmicutes;c__Bacilli;o__Staphylococcales;f__Staphylococcaceae;g__Staphylococcus	61.230%	29.981%	2.430%	21.110%	10.994%	36.709%	3.919%	4.001%	10.034%
d__Bacteria;p__Firmicutes;c__Clostridia;o__Eubacteriales;f__Eubacteriaceae;g__uncultured	0.000%	0.000%	0.068%	0.000%	0.104%	0.000%	0.888%	1.418%	0.355%
d__Bacteria;p__Firmicutes;c__Clostridia;o__Lachnospirales;f__Lachnospiraceae;__	0.000%	0.000%	0.000%	0.000%	0.688%	0.000%	0.000%	0.000%	0.000%
d__Bacteria;p__Firmicutes;c__Clostridia;o__Peptostreptococcales-Tissierellales;f__Peptostreptococcales-Tissierellales;g__Peptoniphilus	0.530%	0.000%	0.000%	0.000%	0.307%	0.000%	0.000%	0.306%	0.000%
d__Bacteria;p__Fusobacteriota;c__Fusobacteriia;o__Fusobacteriales;f__Fusobacteriaceae;g__Fusobacterium	0.000%	1.984%	3.536%	0.000%	3.094%	5.912%	2.335%	5.322%	4.066%
d__Bacteria;p__Fusobacteriota;c__Fusobacteriia;o__Fusobacteriales;f__Leptotrichiaceae;__	0.000%	2.929%	0.000%	0.000%	1.574%	1.316%	1.437%	2.361%	3.339%
d__Bacteria;p__Patescibacteria;c__Saccharimonadia;o__Saccharimonadales;f__Saccharimonadales;g__Saccharimonadales	3.802%	0.581%	3.075%	0.000%	3.673%	0.000%	5.933%	1.970%	0.000%
d__Bacteria;p__Proteobacteria;c__Gammaproteobacteria;o__Burkholderiales;__;__	2.121%	20.418%	4.195%	8.849%	11.459%	0.801%	0.000%	1.838%	2.265%
d__Bacteria;p__Proteobacteria;c__Gammaproteobacteria;o__Enterobacterales;f__Enterobacteriaceae;__	0.330%	2.332%	3.735%	2.666%	0.000%	0.000%	5.439%	0.000%	2.156%
d__Bacteria;p__Proteobacteria;c__Gammaproteobacteria;o__Enterobacterales;f__Enterobacteriaceae;g__Escherichia-Shigella	16.217%	0.000%	0.000%	0.000%	0.000%	1.779%	3.791%	0.841%	0.000%
d__Bacteria;p__Proteobacteria;c__Gammaproteobacteria;o__Pasteurellales;f__Pasteurellaceae;g__Actinobacillus	0.039%	0.000%	0.664%	0.000%	0.941%	0.000%	0.073%	0.264%	0.000%
d__Bacteria;p__Proteobacteria;c__Gammaproteobacteria;o__Xanthomonadales;f__Xanthomonadaceae;g__Xanthomonas	0.000%	1.929%	0.548%	0.000%	1.975%	0.160%	0.549%	0.216%	0.482%

Supplementary File 5
Relative Abundance Data

Fecal: Significant Baseline Genera
None

**Fecal: Significant 2DPT Genera**	**AC-PO7d**	**AC-10mg/kg**	**AC-5mg/kg**	**anti-rsPilA**	**anti-tip-chimer**	**Ofloxacin**	**TS-30mg/kg**	**TS-15mg/kg**	**Saline**
Fusobacterium	34.98%	3.33%	0.00%	0.00%	0.00%	0.03%	0.00%	0.00%	0.03%

**Fecal: Significant 5DPT Genera**	**AC-PO7d**	**AC-10mg/kg**	**AC-5mg/kg**	**anti-rsPilA**	**anti-tip-chimer**	**Ofloxacin**	**TS-30mg/kg**	**TS-15mg/kg**	**Saline**
Fusobacterium	16.129%	10.532%	20.775%	0.000%	0.000%	0.000%	0.000%	0.094%	0.014%
Paraeggerthella	0.000%	3.719%	5.441%	0.000%	0.000%	0.000%	0.000%	0.241%	0.000%
Haemophilus	0.000%	5.700%	3.309%	0.017%	0.158%	0.000%	0.000%	0.000%	0.000%
Undefined genus of the family *Actinomycetaceae*	0.000%	4.470%	0.618%	0.000%	0.000%	0.000%	0.000%	0.000%	0.023%
Undefined genus of Erysipelatoclostridiaceae	0.069%	2.349%	1.224%	0.000%	0.047%	0.209%	0.014%	0.092%	0.019%
Odoribacter	0.355%	0.401%	1.558%	0.000%	0.061%	0.011%	0.000%	0.041%	0.000%
(Ruminococcaceae family) *UBA1819*	1.774%	5.177%	0.834%	0.000%	0.000%	0.000%	0.000%	0.220%	0.029%
Collinsella	0.486%	2.460%	0.238%	0.000%	0.000%	0.000%	0.000%	0.000%	0.072%
(Lachnospiraceae family) *[Eubacterium] ruminantium group*	0.000%	0.000%	0.000%	0.000%	0.000%	0.875%	0.735%	0.896%	0.231%
Actinomyces	0.000%	0.000%	0.000%	0.116%	0.000%	0.000%	0.043%	0.056%	0.163%
(Ruminococcaceae family) *[Eubacterium] siraeum group*	0.131%	0.000%	0.000%	0.000%	0.246%	0.000%	0.000%	0.000%	0.000%
Adlercreutzia	0.553%	0.000%	0.068%	0.000%	0.000%	0.152%	0.000%	0.000%	0.048%
Marvinbryantia	0.469%	0.000%	0.085%	0.000%	0.000%	0.000%	0.059%	0.000%	0.114%
Undefined genus of the family Erysipelatoclostridiaceae	0.098%	0.000%	0.424%	0.125%	0.000%	0.000%	0.129%	0.000%	0.091%
Parabacteroides	1.489%	1.687%	2.815%	0.352%	0.116%	0.223%	0.413%	0.164%	0.364%
Bacteroides	12.593%	17.546%	14.596%	0.640%	1.170%	1.891%	0.958%	1.301%	0.932%
Christensenellaceae R-7 group	1.146%	0.084%	0.380%	4.254%	2.728%	5.404%	2.392%	6.107%	3.813%
Undefined genus of the family Erysipelotrichaceae	2.800%	0.000%	0.175%	9.346%	6.352%	10.484%	19.814%	17.151%	11.416%
Uncultured genus of the family Eubacteriaceae	0.713%	0.000%	0.011%	2.849%	10.126%	4.936%	3.804%	5.411%	9.790%
Uncultured genus of the family Eggerthellaceae	0.000%	0.000%	0.421%	0.361%	0.360%	2.043%	0.844%	1.746%	1.462%
Undefined genus of the family Eggerthellaceae	0.000%	0.000%	0.090%	0.309%	0.255%	0.268%	0.647%	0.874%	0.266%
Mogibacterium	0.000%	0.000%	0.148%	0.516%	0.213%	0.031%	0.243%	0.531%	0.240%
Undefined genus of the family Atopobiaceae	0.175%	0.000%	0.098%	2.075%	0.888%	1.405%	1.845%	0.497%	1.677%
Enterorhabdus	0.255%	0.000%	0.033%	0.718%	0.490%	0.150%	0.940%	0.608%	1.134%
Undefined genus of the order Bacteroidales	0.160%	0.000%	0.019%	0.421%	0.127%	0.206%	0.125%	0.384%	0.428%
(Ruminococcaceae family) Incertae Sedis	0.566%	0.000%	0.000%	2.630%	1.613%	0.508%	1.480%	2.893%	0.390%
Prevotella	2.021%	0.000%	0.000%	1.096%	3.193%	2.551%	5.267%	0.275%	0.725%
Prevotellaceae UCG-001	0.338%	0.000%	0.000%	0.477%	3.339%	3.034%	4.378%	0.357%	3.166%

**Fecal: Significant 7DPT Genera**	**AC-PO7d**	**AC-10mg/kg**	**AC-5mg/kg**	**anti-rsPilA**	**anti-tip-chimer**	**Ofloxacin**	**TS-30mg/kg**	**TS-15mg/kg**	**Saline**
Uncultured genus of the family Eubacteriaceae	0.00%	0.00%	0.11%	3.21%	8.74%	4.35%	3.13%	5.53%	6.08%
Undefined genus of the family Erysipelotrichaceae	0.06%	0.00%	0.01%	8.05%	8.77%	7.80%	17.08%	17.98%	9.28%

Fecal: Significant 9DPT Genera
None

NPL: Significant Baseline Genera
None

**NPL: Significant 2 DPT Genera**	**AC-PO7d**	**AC-10mg/kg**	**AC-5mg/kg**	**anti-rsPilA**	**anti-tip-chimer**	**Ofloxacin**	**TS-30mg/kg**	**TS-15mg/kg**	**Saline**
Methanobrevibacter	21.69%	0.48%	0.37%	0.68%	0.61%	0.00%	0.10%	1.34%	0.00%
Streptococcus	0.00%	0.00%	0.00%	2.96%	0.00%	0.00%	2.61%	0.29%	0.00%
Corynebacterium	0.00%	0.00%	0.00%	0.00%	0.00%	0.00%	3.55%	0.00%	0.00%
Mobiluncus	0.00%	4.28%	4.22%	6.81%	5.03%	5.69%	4.42%	9.51%	10.02%
Bacteroides	21.00%	0.00%	0.00%	0.00%	0.00%	0.00%	1.74%	0.00%	0.00%

**NPL: Significant 5 DPT Genera**	**AC-PO7d**	**AC-10mg/kg**	**AC-5mg/kg**	**anti-rsPilA**	**anti-tip-chimer**	**Ofloxacin**	**TS-30mg/kg**	**TS-15mg/kg**	**Saline**
Mobiluncus	2.891%	2.352%	3.903%	6.326%	5.185%	5.921%	6.052%	8.564%	11.529%
Bacteroides	12.157%	0.000%	0.000%	0.000%	0.000%	0.000%	2.475%	0.000%	0.000%
Undefined genus of the family Lachnospiraceae	0.000%	0.000%	0.000%	0.000%	0.000%	0.000%	0.000%	0.835%	0.000%
Actinobacillus	0.000%	0.000%	0.270%	0.000%	0.000%	0.000%	0.000%	0.000%	0.000%
Olsenella	0.000%	0.116%	0.000%	0.263%	0.000%	0.000%	0.000%	0.000%	0.000%

NPL: Significant 7 DPT Genera
None

NPL: Significant 9 DPT Genera
None
